# Diversity Dynamics of Silurian–Early Carboniferous Land Plants in South China

**DOI:** 10.1371/journal.pone.0075706

**Published:** 2013-09-20

**Authors:** Conghui Xiong, Deming Wang, Qi Wang, Michael J. Benton, Jinzhuang Xue, Meicen Meng, Qi Zhao, Jing Zhang

**Affiliations:** 1 Key Laboratory of Orogenic Belts and Crustal Evolution, Ministry of Education, Department of Geology, Peking University, Beijing, China; 2 State Key Laboratory of Systematic and Evolutionary Botany, Institute of Botany, Chinese Academy of Sciences, Beijing, China; 3 School of Earth Sciences, University of Bristol, Bristol, United Kingdom; 4 Key Laboratory of Evolutionary Systematics of Vertebrates, Institute of Vertebrate Paleontology and Paleoanthropology, Chinese Academy of Sciences, Beijing, China; New York State Museum, United States of America

## Abstract

New megafossil and microfossil data indicate four episodes in the diversification of Silurian–Early Carboniferous land plants of South China, a relatively continuous regional record. Plant diversity increased throughout, but the rising curve was punctuated by three major falls. There were peaks of origination in the Ludlow–Pragian, Givetian, late Famennian and Visean and peaks of extinction in the Pragian–Emsian, Givetian and early Tournaisian. Speciation and extinction rates were highest in the Lochkovian–Pragian and became progressively lower in subsequent stages. High correlation coefficients indicate that these events are associated with the availability of land habitat contingent on eustatic variations and increasing numbers of cosmopolitan genera. Meanwhile, proportions of endemic genera declined gradually. Due to less endemism and more migrations, both speciation and species extinction rates reduced. The changes of diversity and the timing of the three extinctions of land plants in South China are similar to those known already from Laurussia. However, the largest events in the Lochkovian–Pragian and subsequent smaller ones have not been seen in the global pattern of plant evolution. These land plant events do not correspond well temporally with those affecting land vertebrates or marine invertebrates. In South China, the diversity curve of land plants is generally opposite to that of marine faunas, showing a strong effect of eustatic variations. The increasing diversity of both land vertebrates and plants was punctuated above the Devonian–Carboniferous boundary, known as Romer's Gap, implying common underlying constraints on macroevolution of land animals and plants.

## Introduction

The Silurian–Early Carboniferous interval was characterized by a series of eustatic variations as well as changes of atmospheric CO_2_ level and temperature [1]–[4]. During this time, the Frasnian–Famennian (F–F) biocrisis occurred in marine faunas, and was one of the “big five” mass extinctions of the Phanerozoic [5]. However, terrestrial extinctions do not correspond closely in timing or magnitude with marine events [6]–[9]. Land plants diversified in the Early Devonian [10], [11]. They had their highest speciation rates and three extinction events in the Devonian and Early Carboniferous, which are different from either land vertebrates or marine invertebrates [12]. Although the regional diversity patterns of early land plants and their constraints have been discussed before [13], [14], only a few studies have focused on early land plant speciation and extinction events, and their implications (e.g., underlying driving factors) are still not fully understood.

As far as land tetrapods are concerned, regional endemism, occupation of ecospace, and specialization were suggested as three fundamental drivers of species richness [15]. The Devonian land plants and benthic animals of South China include not only high proportions of endemic genera but also some cosmopolitan elements [16], [17]. However, it remains unclear whether these driving factors have the same or similar impacts on land plants, and several key questions are still waiting for answers. For example, are the regional speciation and extinction patterns of land plants unique? To what extent has the abundance of endemic plants influenced the speciation and extinction events in South China? Prior to the present study, relatively little was known about the diversity of early land plants in South China. Although Wang et al. [18] summarized the diversity curves of megafossils in general terms, they did not deliver an integrated data list and elaborated analysis. In this paper, we aim to (1) study the diversity dynamics and speciation and extinction events of the Silurian–Early Carboniferous land plants in South China; and (2) explore what key factors drove the shifts in speciation and extinction rates.

## Data and Methods

For six years, we have collected data on the Silurian–Early Carboniferous land plants of South China. The data include locality and section information, geological formations and species of megafossils and microfossils (i.e., spores and pollen) from the published literature (see Text S1), with some sections and plants studied by ourselves. In former years, such a study might have been rendered difficult by the risk of unrecognized synonymy caused by differing nomenclatures both between research groups in China, and between Chinese paleobotanists and those from other parts of the world. However, our species lists have been substantially revised, with comparisons of taxa in particular families between research groups and collections. Further, international collaboration and comparison is now substantial, and we have endeavored to make all identifications of taxa with due regard to worldwide comparison.

**Figure 1:**
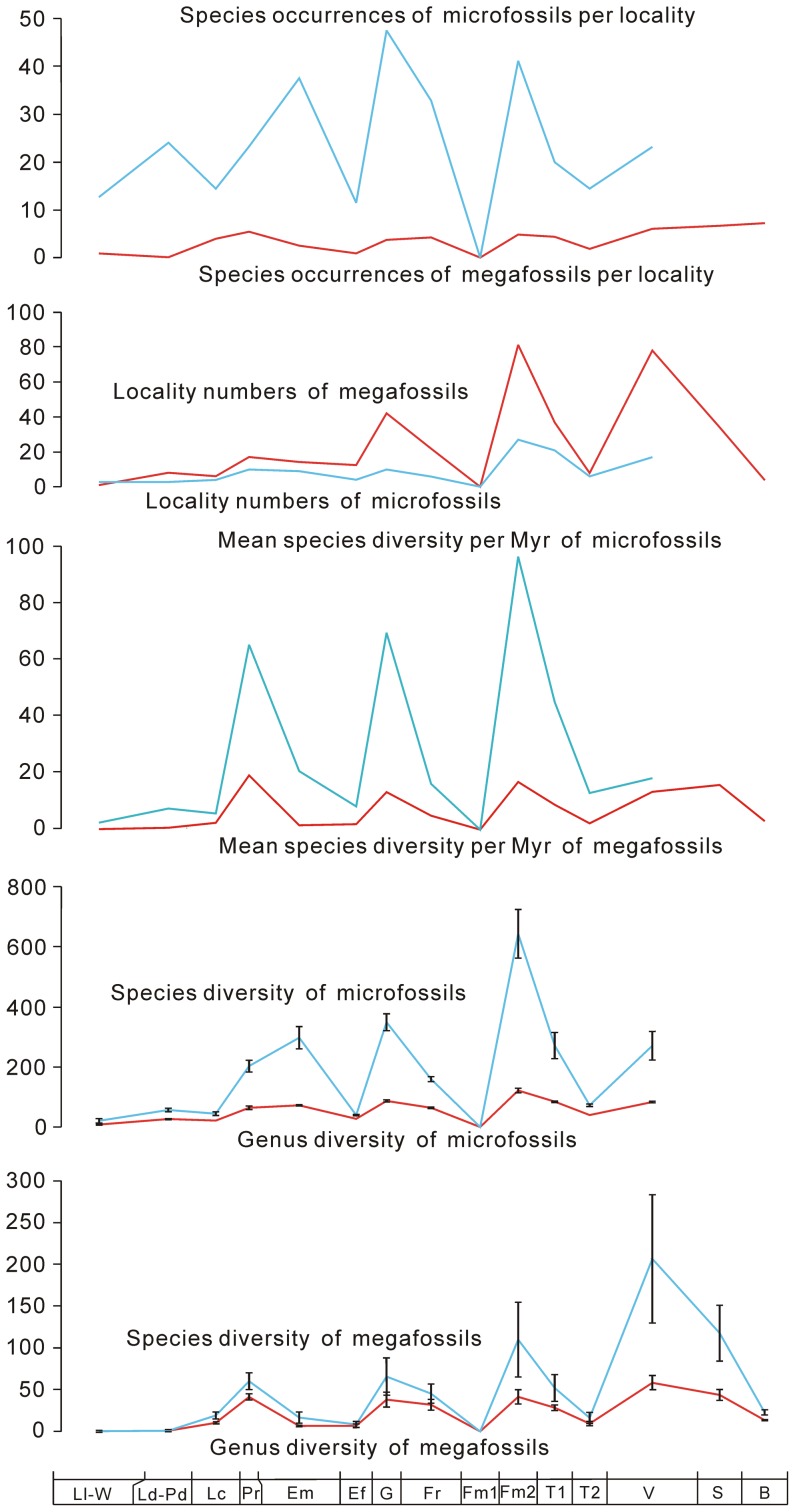
Genus and species diversity, mean species diversity per million years (Myr), locality numbers, and species occurrences per locality of Silurian–Early Carboniferous land plants in South China. Confidence intervals are shown on the curves of the genus and species diversity of land plants. The abbreviations of geological stages are: Llandovery–Wenlock (Ll–W), Ludlow–Pridoli (Ld–Pd), Lochkovian (Lc), Pragian (Pr), Emsian (Em), Eifelian (Ef), Givetian (G), Frasnian (Fr), early Famennian (Fm1), late Famennian (Fm2), early Tournaisian (T1), late Tournaisian (T2), Visean (V), Serpukhovian (S), and Bashkirian (B).

**Figure 2:**
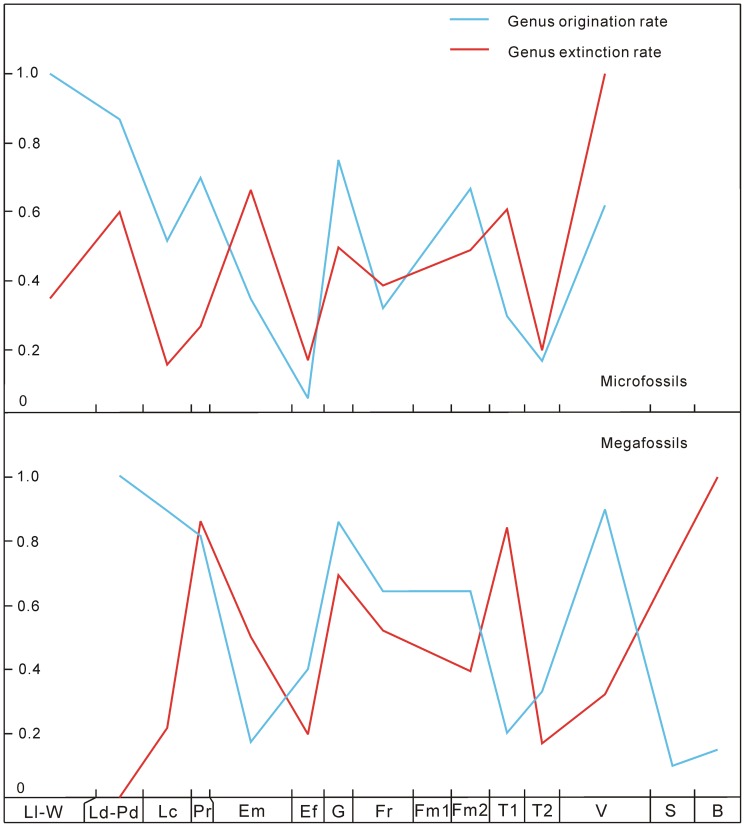
Genus origination and extinction rates of Silurian–Early Carboniferous land plants in South China. The abbreviations of geological stages are the same as in Figure 1.

**Figure 3:**
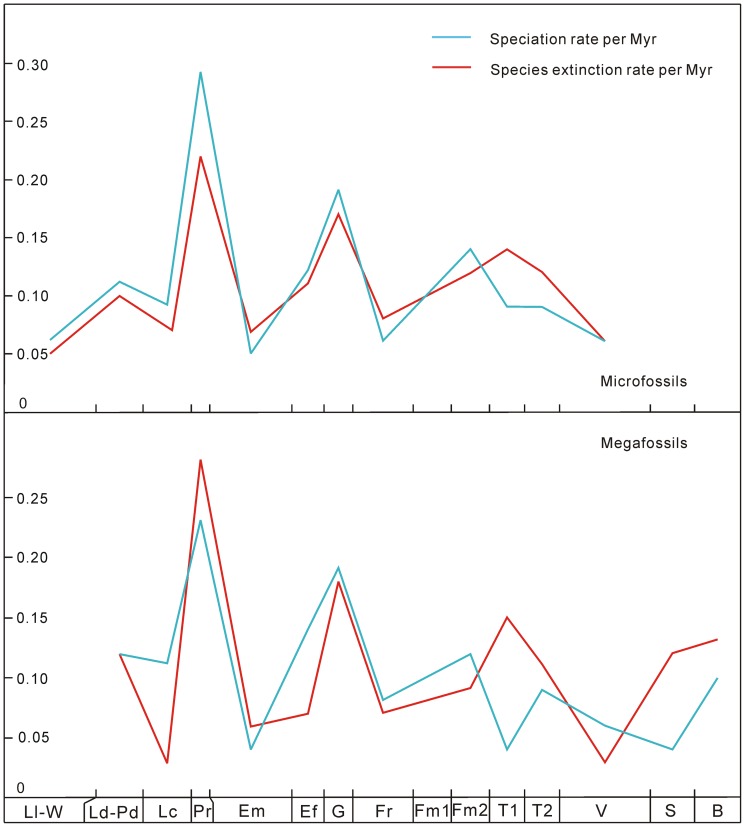
Speciation and species extinction rates per Myr of Silurian–Early Carboniferous land plants in South China. The abbreviations of geological stages are the same as in Figure 1.

**Figure 4:**
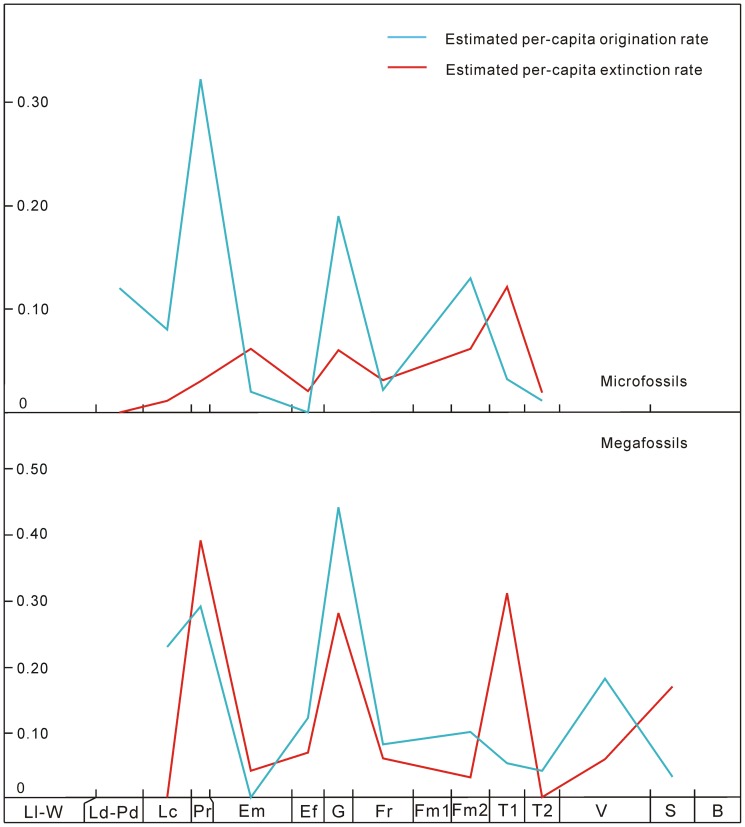
The estimated per-capita origination and extinction rates of Silurian–Early Carboniferous land plants in South China. The abbreviations of geological stages are the same as in Figure 1.

**Figure 5:**
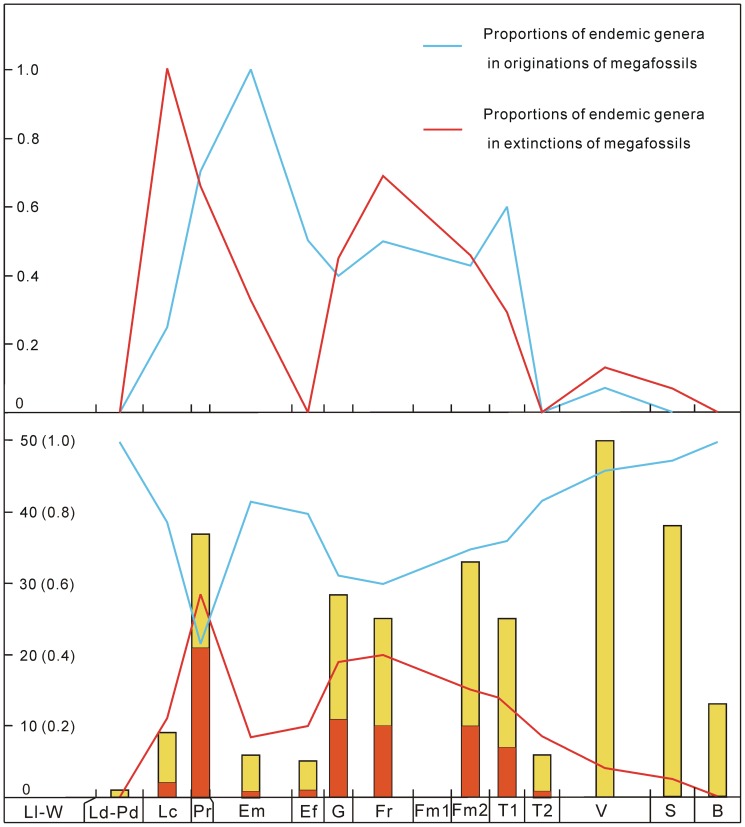
Silurian–Early Carboniferous endemic and cosmopolitan genus numbers of megafossils and their proportions in South China. The abbreviations of geological stages are the same as in Figure 1. Lower part: The red columns and line represent endemic genera and their proportions in the lowest genus diversity of megafossils (see the vertical ordinate); the yellow columns and the blue line represent cosmopolitan genera and their proportions in the lowest genus diversity of megafossils.

The genera of megafossils are assigned to higher groups and identified as endemic or not, depending on whether they occur only in South China or more widely (see Table S1). The ages of all formations yielding land plants in South China are based on regional stratigraphic studies chiefly using biostratigraphic index fossils, and are correlated with the standard subdivisions of the International Stratigraphic Chart [19] (see Table S2). The duration of stages is based on the International Stratigraphic Chart (2013) (http://www.stratigraphy.org/ICSchart/ChronostratChart2013-01.pdf) (see Table S3). South China lacks continental sediments and plant records of the early Famennian and Late Carboniferous (after Bashkirian), and the studied period is divided into 15 chronological stages (see Figs. 1–5, 7).

**Figure 6:**
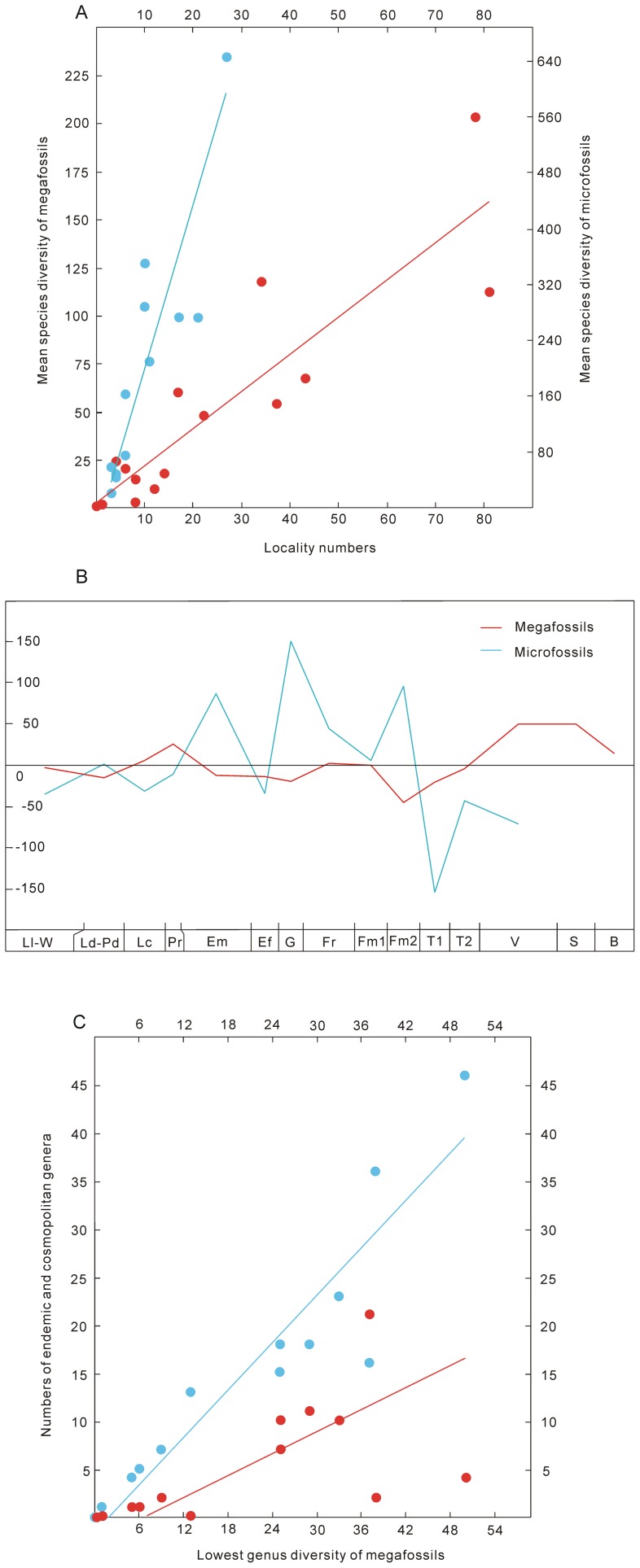
Correlations between Silurian–Early Carboniferous land plant diversity and other variables, and residuals between observed and expected species diversity in South China. A: The red line and dots represent the correlation between mean species diversity and locality numbers of megafossils (*r^2^* = 0.8, *p*<0.001); the blue line and dots represent the correlation between mean species diversity and locality numbers of microfossils (*r^2^* = 0.8, *p*<0.001). B: Residuals between observed and expected mean species diversity of megafossils and microfossils. C: The red line and dots represent the correlation between the lowest genus diversity and numbers of endemic genera (*r^2^* = 0.37, *p* = 0.02); The blue line and dots represent the correlation between the lowest genus diversity and numbers of cosmopolitan genera (*r^2^* = 0.87, *p*<0.001).

The diversity patterns are analyzed by counting the numbers of species and genera of megafossils and microfossils in all localities and formations of each stage. Since the fossil records of some genera and species, i.e., “Lazarus” [20], [21] and other problematic taxa, are uncertain or difficult to identify, we exclude and include such uncertain records; the ranges between lowest (definite) diversity and highest (unfiltered) diversity provide confidence intervals (see Fig. 1, Table S1 and S3). The mean species diversity per million years (Myr) is calculated, locality numbers are counted and species occurrences per locality are measured (see Fig. 1 and Table S3). According to Foote [22], four fundamental categories of taxa (i.e., confined to interval, only bottom boundary crossed, only top boundary crossed, and both boundaries crossed) are applied, and based on this, numbers of originations and extinctions are counted to calculate the genus origination and extinction rates, speciation rates per Myr, species extinction rates per Myr, and estimated per-capita origination and extinction rates (see Figs. 2–4 and Table S3). We calculate the diversity of both endemic and (more or less) cosmopolitan genera as well as their proportions (against the lowest genus diversity of megafossils) in each stage. We also investigate the contributions of endemic genera to originations and extinctions (see Fig. 5 and Table S3). We evaluate the correlations between diversity and several variables (i.e., locality numbers, and endemic and cosmopolitan genera), and the residuals between observed and expected species diversity are analyzed (see Fig. 6).

## Results

### Diversity rise punctuated by three falls

The diversity of Silurian–Early Carboniferous land plants in South China shows progressive increase, punctuated by three major falls, in the Eifelian, early Famennian and late Tournaisian. Hence, we subdivide the plant diversity time series into four episodes, each of which shows a cycle of scarcity–abundance–scarcity in genus and species numbers (Fig. 1). The first episode began with the terrestrialization of plants in the Silurian and ended with decreased diversity in the Eifelian. The diversity of megafossils was very low in the Silurian and rapidly increased in the Lochkovian and Pragian, and then dramatically declined in the Emsian and Eifelian. Compared to megafossils, microfossils had a higher diversity in the Silurian, indicating the likely faster evolution and proliferation of early plants; microfossils were more abundant in the Emsian, exhibiting a continuous rise after the radiation in the Pragian. In the second episode, from Eifelian to early Famennian, the genus diversity of megafossils and microfossils showed a high level in the Givetian, and a slight decrease or equilibrium in the Frasnian. The species diversity of these fossils however dropped significantly from Givetian to Frasnian. In the early Famennian, presently there is no plant record in South China. As to the third episode, genus and species diversity (especially the latter) reached a zenith in the late Famennian and declined in the Tournaisian. Plants were rarely documented in the late Tournaisian. In the last episode, microfossil data were insufficient, and the diversity of megafossils peaked in the Visean and decreased gradually from Serpukhovian to Bashkirian.

Different from the progressively increasing species diversity during the Silurian–Early Carboniferous, the four peaks (Pragian, Givetian, late Famennian, and Visean) of mean species diversity per Myr of megafossils demonstrated no obvious changes in values (Fig. 1). When we estimated minimum numbers of localities in all stages, the species occurrences of megafossils and microfossils showed the same pattern of troughs and peaks as the above diversity (Fig. 1).

### Shifts in origination (speciation) and extinction rates

The values of genus origination and extinction rates of megafossils and microfossils varied extensively from the Silurian to Early Carboniferous (Fig. 2). Origination rates were higher in the Ludlow–Pridoli, Pragian, Givetian, late Famennian and Visean, but lower in the Lochkovian, Emsian–Eifelian, Frasnian and Tournaisian. The extinction rates showed a similar tendency as the origination rates in the Silurian and Devonian, but a different trend in the Early Carboniferous. The peak values of origination and extinction rates appeared to be stable. The peaks and troughs of speciation rates per Myr, species extinction rates per Myr (Fig. 3), and the estimated per-capita origination and extinction rates (Fig. 4) coincided temporally with the genus origination and extinction rates. However, land plants had their highest speciation and species extinction rates per Myr in the Lochkovian–Pragian. These rates became progressively lower in the Givetian, late Famennian and Tournaisian.

### Decreasing diversity of endemic genera

The diversity of endemic genera greatly increased after the Silurian and attained a peak in the Pragian, when these genera exceeded the proportions of cosmopolitan genera (Fig. 5). The diversity of endemic genera dropped noticeably as the whole diversity of megafossils decreased in the Emsian and Eifelian; it became higher in the Givetian, Frasnian, late Famennian and early Tournaisian, and then lower in later stages. In all stages except for the Pragian, endemic genera were fewer than cosmopolitan genera. In general, the proportions of endemic genera gradually declined from the Early Devonian to Early Carboniferous, whereas cosmopolitan genera followed an opposite pattern. Nearly, or over 50% genera of originations and extinctions were endemic in the Early Devonian. The contributions of endemic genera to originations and extinctions decreased strikingly in the Middle–Late Devonian and were insignificant in the Early Carboniferous.

### Correlation analyses

The mean species diversities of megafossils and microfossils show high correlation coefficients (*r^2^* = 0.8, *p*<0.001) with their locality numbers (Fig. 6A). The residuals between observed and expected species diversity are higher in the Pragian−Emsian, Givetian, late Famennian and Visean, and lower in the Eifelian, early Famennian and early Tournaisian (Fig. 6B). The number of endemic genera has a lower correlation coefficient (*r^2^* = 0.37, *p* = 0.02) with the genus diversity of megafossils, whereas the number of cosmopolitan genera has a much higher coefficient (*r^2^* = 0.87, *p*<0.001) (Fig. 6C).

## Discussion

### The effect of sampling on diversity of land plants

The high correlation coefficients between diversity and locality numbers could provide evidence for a strong sampling bias, or that both measures are redundant with each other [23], [24]. It is noteworthy that the curves of species occurrences per locality show similar troughs and peaks to those of genus and species diversity. The present study shows that land plant diversity in South China was high in the Pragian, Givetian and late Famennian, and low in the Emsian–Eifelian and early Famennian. Such changes of diversity roughly correspond with those in Laurussia, the Acadian terrain and southern Siberia [13], [14]. The congruence in diversity patterns with well-studied areas, and the similar patterns between two variables (i.e., species occurrences per locality, residuals) and total diversity in all localities indicate that sampling bias does not have an immense effect on the early land plant diversity in South China.

### Intensive speciation and extinction events of land plants

There are four speciation events (Ludlow–Pragian, Givetian, late Famennian, and Visean) and three extinction events (Pragian–Emsian, Givetian, and early Tournaisian) recognized in the megafossil and microfossil records of Silurian–Early Carboniferous land plants in South China. These regional extinction events coincide temporally with all (land and marine) organisms [25] or land plants [12] on a global scale. In South China, however, the largest speciation and extinction events occurred in the Lochkovian–Pragian and the later events became progressively minor. This pattern has not been shown in the global scale of plant evolution as indicated by Niklas [12].

**Figure 7:**
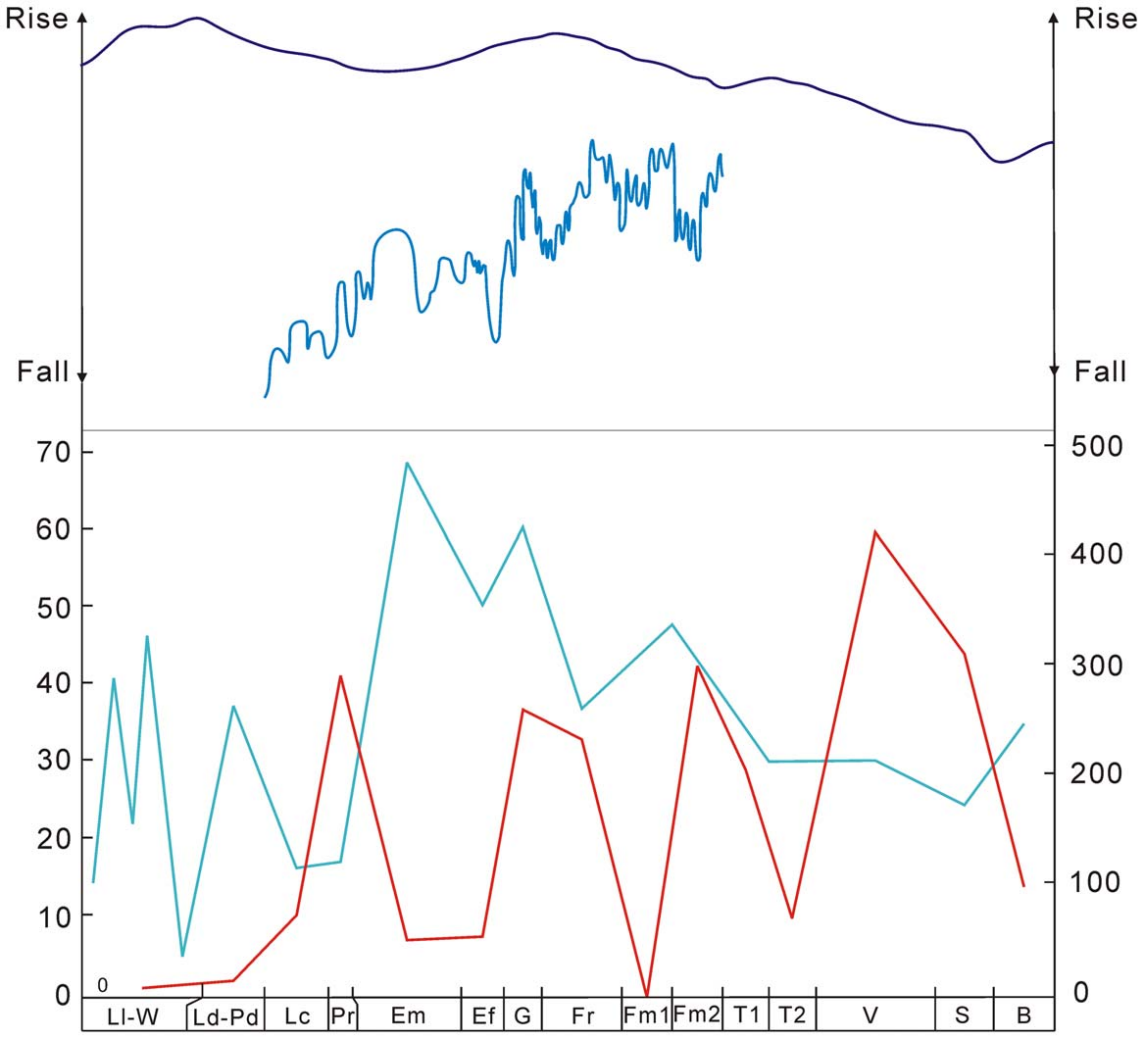
Comparisons between genus diversity of land plants (see the left vertical ordinate and red line; this study) and marine faunas (see the right vertical ordinate and bule line; modified from [**31**]) in South China (the lower part), and sea-level changes (the upper part; the blue line is modified from [**33**]; the purple line is modified from [**37**]). The abbreviations of geological stages are the same as in Figure 1.

As for land vertebrates, the increasing diversity trend was reversed around the Devonian–Carboniferous boundary by a diversity drop, which is known as Romer's Gap, indicating that either fossils were missing because of non-preservation [26], [27] and collection failure [28] or a genuine dip in diversity associated with low atmospheric oxygen levels [29]. In contrast, land plants underwent more intensive events, among which the early Tournaisian event in South China corresponds temporally with Romer's Gap. This implies common underlying constraints on macroevolution of land animals and plants at the Devonian–Carboniferous boundary. Comparisons between land plants and animals in South China are difficult because of absence of data on the latter. Marine faunas suffered stepwise crises near the F–F boundary (F–F mass extinction), and about 50% of genera became extinct [2], [30]–[32]. The land plants of South China experienced three events, with the biggest one in the Lochkovian–Pragian. They experienced a similar magnitude of extinction in the Givetian as marine faunas did near the F–F boundary, and continuing loss of diversity from Givetian to early Famennian. The diversity of marine faunas in South China was high in the Silurian, much lower in the Lochkovian–Pragian, rose to peaks in the Emsian–Eifelian–Givetian, decreased from Givetian to Famennian, and remained low in the Tournaisian–Visean (Fig. 7) [33]. The coeval land plants and marine faunas of South China are generally opposite in their diversity dynamics (Fig. 7).

### Eustatic variations greatly controlling speciation and extinction

These generally opposite diversity patterns of land plants and marine faunas in South China imply that available living space was a crucial factor in the speciation and extinction of organisms during the Silurian and Early Carboniferous. This is reinforced by the general congruence and high correlation coefficients between locality numbers and mean species diversity of land plants. Through geological time, habitat size for organisms was greatly affected by eustatic variations, which might have resulted from climate and ice-volume changes [34]. Sea-level change in South China is represented by two large transgressive–regressive cycles, i.e., Lochkovian to Eifelian, and late Eifelian to late Famennian, which reached their highstands in the middle Emsian and late Frasnian (Fig. 7) [35]. As in Laurussia [36], generally sea level rose in the Givetian and continued high through most of the Frasnian, and then a regression occurred in the late Famennian. Thus, in South China, the lower diversity of marine faunas in the Lochkovian–Pragian results directly from sea level fall, which corresponds with more adequate space for the diversification of land plants [18]; locality numbers and plant diversity showed lower values in the Emsian–Eifelian, Frasnian–early Famennian, but higher values in the Givetian and late Famennian. Although sea level rose in the Frasnian, marine faunas had lower diversity [33]. They seem to have been susceptible to anoxic events and/or global climatic changes in the late Frasnian [37], [38]. Continental sediments and plants of the early Famennian have not been recorded in South China, but existed in Laurussia, the Acadian terrain and southern Siberia [13], [14]. This suggests that sea-level change may have had different effects in different regions according to their distinctive geography (e.g., size, position, and geomorphology). Global sea level rose in the Tournaisian and dropped in the Visean (Fig. 7) [39], which perhaps led to the early Tournaisian extinction and Visean radiation of land plants in South China.

### Endemism and migration greatly controlling speciation and extinction

**Figure 8:**
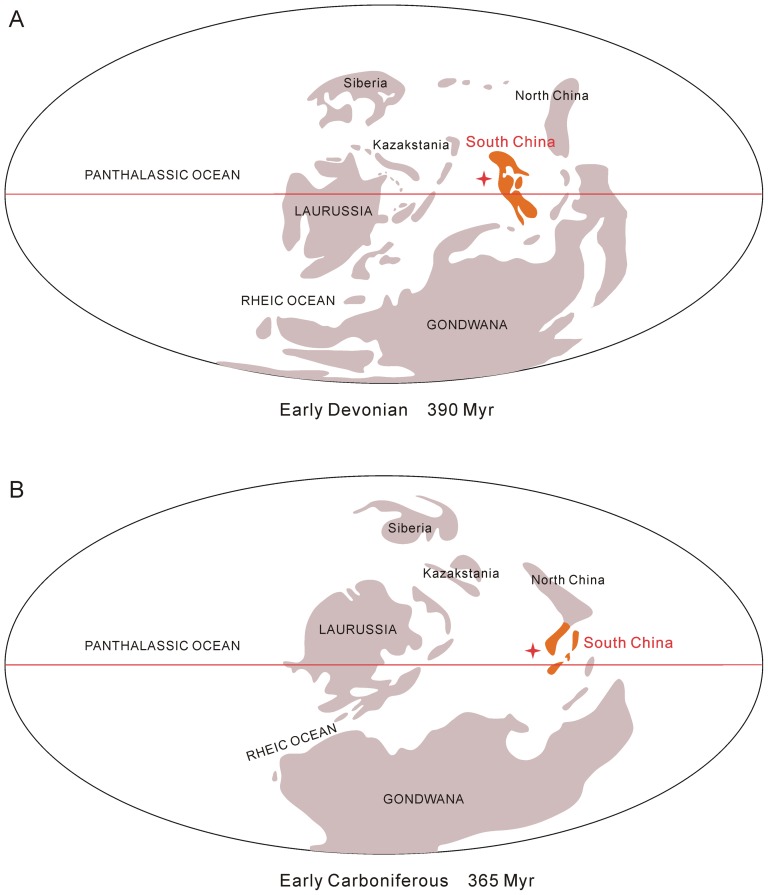
The geographical position of South China during the Devonian and Early Carboniferous (modified from [**40**]).

The number of cosmopolitan genera has a high correlation coefficient with the genus diversity of megafossils, suggesting that cosmopolitan elements were important components of total diversity. As endemism reduced through time, and cosmopolitan taxa proliferated to compensate the deficiency, the magnitude of speciation and extinction events decreased gradually (Figs. 3 and 5). Endemic genera were especially sparse in the Early Carboniferous, indicating that the frequency of migration or interaction between South China and other regions increased greatly after the Devonian. As Raymond [40] and Raymond et al. [41] explained, numerous equatorial genera of the Carboniferous expanded their ranges northward and reduced the global endemic plant diversity because of the climatic amelioration in the north middle–high latitudes caused by the collision of Laurussia and Gondwana. The South China Block was situated in the equatorial region during the Devonian–Early Carboniferous (Figs. 8A and B) [42], so plant migrations reduced not only the speciation rate but also the species extinction rate in the wake of the late Famennian sea level rise in South China and the climatic amelioration in the north middle–high latitudes. These two rates declined at similar magnitudes, and so maintained a roughly stable peak diversity throughout early land plant histories.

## Supporting Information

Table S1 **Silurian–Early Carboniferous genera and species of megafossils in South China. Plants in shadow area can not be identified, or their occurrences are not doubtless. Thus they are not included to calculate the lowest (definite) diversity.**
(PDF)

Table S2 **Silurian–Early Carboniferous geological strata in South China (main references: Regional geology of each province of South China; Cai Chongyang and Li Xingxue, 1982; Cai Chongyang, 2000; Wu Xiuyuan and Zhu Huaicheng, 2000; Wang Deming et al., 2002; Ma Xueping et al., 2009; Gonez et al., 2012).**
(PDF)

Table S3 **Silurian–Early Carboniferous diversity values and other mentioned variables (the abbreviations of geological stages are the same as in** **Figure 1** **).**
(PDF)

Text S1 **The Original Silurian–Early Carboniferous paleobotanical data of South China.**
(PDF)
