# Supplementary material for: Diversity Dynamics of Silurian–Early Carboniferous Land Plants in South China
Source: PLoS One. 2013 Sep 20;8(9):e75706. doi: 10.1371/journal.pone.0075706 (PMC3779156; doi:10.1371/journal.pone.0075706)
Supplement: Table S1 — Silurian–Early Carboniferous genera and species of megafossils in South China. Plants in shadow area can not be identified, or their occurrences are not doubtless. Thus they are not included to calculate the lowest (definite) diversity. (PDF) [file pone.0075706.s001.pdf]

Llandovery–Wenlock				
Class	Order/Family/Group	Genus	Species	Endemic genus or not
Incertae Sedis	Incertae Sedis	*Pinnatiramosus*	*Pinnatiramosus qianensis*	
**Ludlow–Pridoli**				
Class	Order/Family/Group	Genus	Species	Endemic genus or not
Zosterophyllopsida	Zosterophyllales	*Zosterophyllum*	*Zosterophyllum qujingense*	No
			Zosterophyllum? indet.	
Incertae Sedis	Incertae Sedis	*Aberlemnia*?	*Aberlemnia*? sp.	
**Lochkovian**				
Class	Order/Family/Group	Genus	Species	Endemic genus or not
Zosterophyllopsida	Zosterophyllales	*Hicklingia*	*Hicklingia* cf. *edwardii*	No
Zosterophyllopsida	Zosterophyllales	*Zosterophyllum*	*Zosterophyllum longa*	No
Zosterophyllopsida	Zosterophyllales	*Zosterophyllum*	*Zosterophyllum minorstachyum*	
Zosterophyllopsida	Zosterophyllales	*Zosterophyllum*	*Zosterophyllum myretonianum*	
Zosterophyllopsida	Zosterophyllales	*Zosterophyllum*	*Zosterophyllum shengfengense*	
Zosterophyllopsida	Zosterophyllales	*Zosterophyllum*	*Zosterophyllum sichuanense*	
Zosterophyllopsida	Zosterophyllales	*Zosterophyllum*	*Zosterophyllum* sp.	
Zosterophyllopsida	Zosterophyllales	*Zosterophyllum*	*Zosterophyllum* sp.	
Zosterophyllopsida	Zosterophyllales	*Zosterophyllum*	*Zosterophyllum* sp.2	
Zosterophyllopsida	Zosterophyllales	*Zosterophyllum*	*Zosterophyllum xishanense*	
Zosterophyllopsida	Zosterophyllales	*Zosterophyllum*	*Zosterophyllum yunnanicum*	
Zosterophyllopsida	Gosslingiales	*Oricilla*	*Oricilla unilateralis*	No
Zosterophyllopsida	Incertae Sedis	*Xitunia*	*Xitunia spinitheca*	Yes
Lycopsida	Drepanophycales	*Drepanophycus*	*Drepanophycus spinaeformis*	No
Lycopsida	Drepanophycales	*Drepanophycus*	*Drepanophycus spinosus*	
Lycopsida	Drepanophycales	*Drepanophycus*?	*Drepanophycus*? *ramificanalis*	
Lycopsida	Drepanophycales	*Drepanophycus*?	*Drepanophycus*? sp.	
Lycopsida	Protolepidodendrales	A protolepidodendrid lycopsids		
Rhyniopsida	Rhyniales	*Eogaspsiea*	*Eogaspsiea gracilis*	No
Rhyniopsida	Rhyniales	*Uskiella*	*Uskiella* sp.	No
Pteropsida	Psilophytales	*Psilophyton*?	*Psilophyton*? sp.	
Incertae Sedis	Incertae Sedis	*Amplectosprangium*	*Amplectosprangium jiangyouense*	Yes
**Pragian**				
Class	Order/Family/Group	Genus	Species	Endemic genus or not
Zosterophyllopsida	Zosterophyllales	*Distichophytum*	*Distichophytum* sp.	No
Zosterophyllopsida	Zosterophyllales	*Gumuia*	*Gumuia zyzzata*	Yes
Zosterophyllopsida	Zosterophyllales	*Hicklingia*	*Hicklingia* cf. *edwardii*	No
Zosterophyllopsida	Zosterophyllales	cf. *Hicklingia*	cf. *Hicklingia* sp.	
Zosterophyllopsida	Zosterophyllales	*Ramoferis*	*Ramoferis amalia*	Yes
Zosterophyllopsida	Zosterophyllales	*Wenshania*	*Wenshania zhichangensis*	Yes
Zosterophyllopsida	Zosterophyllales	*Zosterophyllum*	*Zosterophyllum australianum*	No

Zosterophyllopsida	Zosterophyllales	*Zosterophyllum*	*Zosterophyllum longa*	
Zosterophyllopsida	Zosterophyllales	*Zosterophyllum*	*Zosterophyllum minifertillum*	
Zosterophyllopsida	Zosterophyllales	*Zosterophyllum*	*Zosterophyllum myretonianum*	
Zosterophyllopsida	Zosterophyllales	*Zosterophyllum*	*Zosterophyllum ramosum*	
Zosterophyllopsida	Zosterophyllales	*Zosterophyllum*	*Zosterophyllum sichuanense*	
Zosterophyllopsida	Zosterophyllales	*Zosterophyllum*	*Zosterophyllum sinense*	
Zosterophyllopsida	Zosterophyllales	*Zosterophyllum*	*Zosterophyllum tenerum*	
Zosterophyllopsida	Zosterophyllales	*Zosterophyllum*	*Zosterophyllum yunnanicum*	
Zosterophyllopsida	Zosterophyllales	*Zosterophyllum*	*Zosterophyllum* sp.	
Zosterophyllopsida	Zosterophyllales	*Zosterophyllum*	*Zosterophyllum* sp.	
Zosterophyllopsida	Zosterophyllales	*Zosterophyllum*	*Zosterophyllum* sp.	
Zosterophyllopsida	Zosterophyllales	*Zosterophyllum*	*Zosterophyllum* sp.2	
Zosterophyllopsida	Zosterophyllales	*Zosterophyllum*	*Zosterophyllum* sp.4	
Zosterophyllopsida	Zosterophyllales	*Zosterophyllum*	*Zosterophyllum* sp.6	
Zosterophyllopsida	Zosterophyllales	*Zosterophyllum*	*Zosterophyllum* sp.7	
Zosterophyllopsida	Zosterophyllales	*Zosterophyllum*	*Zosterophyllum yunnanicum*?	
Zosterophyllopsida	Zosterophyllales	*Zosterophyllum*?	*Zosterophyllum*? *myretonianum*	
Zosterophyllopsida	Zosterophyllales	*Zosterophyllum*?	*Zosterophyllum*? sp.	
Zosterophyllopsida	Gosslingiales	*Discalis*	*Discalis longistipa*	Yes
Zosterophyllopsida	Gosslingiales	*Oricilla*	*Oricilla* sp.	No
Zosterophyllopsida	Gosslingiales	*Oricilla*	*Oricilla unilateralis*	
Zosterophyllopsida	Adoketophytales	*Adoketophyton*	*Adoketophyton parvulum*	Yes
Zosterophyllopsida	Adoketophytales	*Adoketophyton*	*Adoketophyton* sp.	
Zosterophyllopsida	Adoketophytales	*Adoketophyton*	*Adoketophyton subverticillatum*	
Zosterophyllopsida	Incertae Sedis	*Guangnania*	*Guangnania cuneata*	No
Zosterophyllopsida	Incertae Sedis	*Yunia*	*Yunia dichotoma*	Yes
Lycopsida	Drepanophycales	*Baragwanathia*	*Baragwanathia* sp.	No
Lycopsida	Drepanophycales	*Baragwanathia?*	*Baragwanathia*? sp.	
Lycopsida	Drepanophycales	*Baragwanathia?*	*Baragwanathia? yunnanensis*	
Lycopsida	Drepanophycales	*Drepanophycus*	*Drepanophycus qujingensis*	No
Lycopsida	Drepanophycales	*Drepanophycus*	*Drepanophycus spinaeformis*	
Lycopsida	Drepanophycales	*Drepanophycus*	*Drepanophycus spinosus*	
Lycopsida	Drepanophycales	*Drepanophycus?*	*Drepanophycus*? sp.	
Lycopsida	Drepanophycales	*Halleophyton*	*Halleophyton zhichangense*	Yes
Lycopsida	Drepanophycales?	*Hueberia*	*Hueberia zhichangensis*	Yes
Lycopsida	Protolepidodendrales	A protolepidodendrid lycopsids		
Lycopsida	Protolepidodendrales	*Zhenglia*	*Zhenglia radiate*	Yes
Rhyniopsida	Rhyniales	*Eogaspsiea*	*Eogaspsiea gracilis*	No
Rhyniopsida	Rhyniales	*Uskiella*	*Uskiella* sp.	No
Rhyniopsida?	Incertae Sedis	*Huia*	*Huia gracilis*	No
Rhyniopsida?	Incertae Sedis	*Huia*	*Huia recurvata*	

Pteropsida	Psilophytales	*Psilophytites*	*Psilophytites* sp.	No
Pteropsida	Psilophytales	*Psilophytites?*	*Psilophytites*? sp.	
Pteropsida	Psilophytales	*Psilophyton*	*Psilophyton primitivum*	No
Pteropsida	Psilophytales	*Psilophyton?*	*Psilophyton*? sp.	
Pteropsida	Psilophytales	*Pauthecophyton*	*Pauthecophyton gracile*	Yes
Sphenopsida?		*Cervicornus*	*Cervicornus wenshanensis*	Yes
Sphenopsida?		*Estinnophyton*	*Estinnophyton yunnanense*	No
Progymnospermopsida?	Incertae Sedis	*Hedeia*	*Hedeia sinica*	No
Progymnospermopsida?	Incertae Sedis	*Polythecophyton*	*Polythecophyton demissum*	Yes
gymnosperms?	Incertae Sedis	*Celatheca*	*Celatheca beckii*	Yes
Eophyllophytopsida	Eophyllophytales	*Eophyllophyton*	*Eophyllophyton bellum*	Yes
Stachyophytopsida	Stachyophytales	*Stachyophyton*	*Stachyophyton yunnanense*	Yes
Incertae Sedis	Taeniocradaceae	*Taeniocrada*	*Taeniocrada decheniana*	No
Incertae Sedis	Taeniocradaceae	*Taeniocrada*?	*Taeniocrada*? cf. *dubia*	
Incertae Sedis	Taeniocradaceae	*Taeniocrada*?	*Taeniocrada*? *langii*	
Incertae Sedis	Incertae Sedis	*Amplectosprangium*	*Amplectosprangium jiangyouense*	Yes
Incertae Sedis	Incertae Sedis	*Bracteophyton*	*Bracteophyton variatum*	Yes
Incertae Sedis	Incertae Sedis	*Catenalis*	*Catenalis digitata*	Yes
Incertae Sedis	Incertae Sedis	*Changwuia*	*Changwuia schweitzeri*	Yes
Incertae Sedis	Incertae Sedis	*Demersatheca*	*Demersatheca contigua*	Yes
Incertae Sedis	Incertae Sedis	*Dibracophyton*	*Dibracophyton acrovatum*	Yes
**Emsian**				
Class	Order/Family/Group	Genus	Species	Endemic genus or not
Zosterophyllopsida	Zosterophyllales	*Zosterophyllum*	*Zosterophyllum bifurcatum*	No
Zosterophyllopsida	Zosterophyllales	*Zosterophyllum*	*Zosterophyllum* cf. *yunnanicum*	
Zosterophyllopsida	Zosterophyllales	*Zosterophyllum*	*Zosterophyllum dushanense*	
Zosterophyllopsida	Zosterophyllales	*Zosterophyllum*	*Zosterophyllum* sp.1	
Zosterophyllopsida	Zosterophyllales	*Zosterophyllum*	*Zosterophyllum* sp.3	
Zosterophyllopsida	Zosterophyllales	*Zosterophyllum*	*Zosterophyllum* sp.5	
Zosterophyllopsida	Zosterophyllales	*Zosterophyllum*	*Zosterophyllum* sp.a	
Zosterophyllopsida	Zosterophyllales	*Zosterophyllum*	*Zosterophyllum* sp.A	
Zosterophyllopsida	Zosterophyllales	*Zosterophyllum*	*Zosterophyllum* sp.B	
Zosterophyllopsida	Zosterophyllales	*Zosterophyllum*	*Zosterophyllum* sp.b	
Zosterophyllopsida	Zosterophyllales	*Zosterophyllum*	*Zosterophyllum* sp.C	
Zosterophyllopsida	Zosterophyllales	*Zosterophyllum*	*Zosterophyllum spathulatum*	
Zosterophyllopsida	Zosterophyllales	*Zosterophyllum*	*Zosterophyllum yunnanicum*	
Zosterophyllopsida	Zosterophyllales	*Zosterophyllum?*	*Zosterophyllum*? sp.	
Zosterophyllopsida	Zosterophyllales	Zosterophyllum?	Zosterophyllum? sp.	
Lycopsida	Drepanophycales	*Drepanophycus*	*Drepanophycus qujingensis*	No
Lycopsida	Drepanophycales	*Drepanophycus*	*Drepanophycus spinaeformis*	
Rhyniopsida?	Incertae Sedis	*Hsüa*	*Hsüa deflexa*	Yes
Rhyniopsida?	Incertae Sedis	*Hsüa*	*Hsüa robusta*	

Pteropsida	Psilophytales	*Psilophytites*	*Psilophytites* sp.	No
Pteropsida	Psilophytales	*Psilophyton*	*Psilophyton* cf. *goldschmidtii*	No
Incertae Sedis	Taeniocradaceae	*Taeniocrada*	*Taeniocrada decheniana*	No
Incertae Sedis	Taeniocradaceae	*Taeniocrada*	*Taeniocrada* sp.	
“Lazarus taxa”			*Psilophytites* sp.	
**Eifelian**				
Class	Order/Family/Group	Genus	Species	Endemic genus or not
Lycopsida	Protolepidodendrales	*Minarodendron*	*Minarodendron cathaysiense*	Yes
Lycopsida	Incertae Sedis	*Lepidodendropsis*?	*Lepidodendropsis*? sp.	
Lycopsida	Incertae Sedis	*Aspidiaria*?	*Aspidiaria*? sp.	
Lycopsida	Incertae Sedis	*Dictyoxylon*?	*Dictyoxylon*? sp.	
Pteropsida	Psilophytales	*Psilophytites*	*Psilophytites* sp.	No
Pteropsida	Psilophytales	*Psilophytites*	*Psilophytites* sp.	
Pteropsida	Psilophytales	*Psilophyton*	*Psilophyton* sp.	No
Pteropsida	Psilophytales	*Psilophyton*	*Psilophyton* sp.A	
Pteropsida	Psilophytales	*Thursophyton*	*Thursophyton* sp.	No
Incertae Sedis	Taeniocradaceae	*Taeniocrada*	*Taeniocrada* sp.	No
“Lazarus taxa”		*Drepanophycus*	*Drepanophycus spinosus*	
“Lazarus taxa”		*Drepanophycus*	*Drepanophycus qujingensis*	
“Lazarus taxa”			*Taeniocrada decheniana*	
**Givetian**				
Class	Order/Family/Group	Genus	Species	Endemic genus or not
Lycopsida	Drepanophycales	*Drepanophycus*	*Drepanophycus qujingensis*	No
Lycopsida	Drepanophycales	*Drepanophycus*	*Drepanophycus spinaeformis*	
Lycopsida	Protolepidodendrales	*Colpodexylon*	*Colpodexylon variabile*	No
Lycopsida	Protolepidodendrales	*Colpodexylon*?	*Colpodexylon*? sp.	
Lycopsida	Protolepidodendrales	*Lepidosigillaria?*	*Lepidosigillaria? cycloformis*	
Lycopsida	Protolepidodendrales	*Minarodendron*	*Minarodendron cathaysiense*	Yes
Lycopsida	Protolepidodendrales	*Minarodendron*	*Minarodendron* cf. *cathaysiense*	
Lycopsida	Protolepidodendrales	*Prelepidodendron?*	*Prelepidodendron? beichuanensis*	
Lycopsida	Protolepidodendrales	*Prelepidodendron?*	*Prelepidodendron?* cf. *jinbaoshiensis*	
Lycopsida	Protolepidodendrales	*Prelepidodendron?*	*Prelepidodendron? jinbaoshiensis*	
Lycopsida	Protolepidodendrales	*Prelepidodendron?*	*Prelepidodendron? sichuanensis*	
Lycopsida	Protolepidodendrales	*Prelepidodendron?*	*Prelepidodendron?* sp.	
Lycopsida	Protolepidodendrales	*Protolepidodendron?*	*Protolepidodendron? arborecens*	
Lycopsida	Protolepidodendrales	*Protolepidodendron?*	*Protolepidodendron? lixianense*	
Lycopsida	Protolepidodendrales	*Protolepidodendron?*	*Protolepidodendron? minutum*	
Lycopsida	Protolepidodendrales	*Protolepidodendron?*	*Protolepidodendron?* sp.	
Lycopsida	Protolepidodendrales	*Protolepidodendron?*	*Protolepidodendron?* sp.	
Lycopsida	Incertae Sedis	*Longostachys*	*Longostachys latisporophyllus*	Yes
Lycopsida	Isoëtales *sensu lato*	*Cyclostigma*	*Cyclostigma* sp.	No
Lycopsida	Isoëtales *sensu lato*	*Cyclostigma?*	*Cyclostigma? kiltorkens*	

Lycopsida	Isoëtales *sensu lato*	*Knorria*	*Knorria* sp.	No
Lycopsida	Incertae Sedis	*Lepidodendropsis*	*Lepidodendropsis arborescens*	No
Lycopsida	Incertae Sedis	*Lepidodendropsis*	*Lepidodendropsis* cf. *arborescens*	
Lycopsida	Incertae Sedis	*Lepidodendropsis*	*Lepidodendropsis* cf. *hirmeri*	
Lycopsida	Incertae Sedis	*Lepidodendropsis*	*Lepidodendropsis* cf. *sinensis*	
Lycopsida	Incertae Sedis	*Lepidodendropsis*	*Lepidodendropsis* cf. *wutubulakensis*	
Lycopsida	Incertae Sedis	*Lepidodendropsis*	*Lepidodendropsis guanzhuangensis*	
Lycopsida	Incertae Sedis	*Lepidodendropsis*	*Lepidodendropsis kazachstanica*	
Lycopsida	Incertae Sedis	*Lepidodendropsis*	*Lepidodendropsis niuewanensis*	
Lycopsida	Incertae Sedis	*Lepidodendropsis*	*Lepidodendropsis* sp.	
Lycopsida	Incertae Sedis	*Lepidodendropsis*	*Lepidodendropsis* sp.	
Lycopsida	Incertae Sedis	*Lepidodendropsis*	*Lepidodendropsis theodori*	
Lycopsida	Incertae Sedis	*Lepidodendropsis*	*Lepidodendropsis tiaomaensis*	
Lycopsida	Incertae Sedis	*Lepidodendropsis?*	*Lepidodendropsis? sinensis*	
Lycopsida	Isoëtales *sensu lato*	*Lepidostrobus?*	*Lepidostrobus? dentatus*	
Lycopsida	Isoëtales *sensu lato*	*Lepidostrobus?*	*Lepidostrobus?* sp.	
Lycopsida	Isoëtales *sensu lato*	*Sublepidodendron*	*Sublepidodendron* sp.	No
Lycopsida	Isoëtales *sensu lato*	*Syringodendron*	*Syringodendron* sp.3	No
Lycopsida	Isoëtales *sensu lato*	*Syringodendron*	*Syringodendron* sp.4	
Lycopsida	Incertae Sedis	*Yuguangia*	*Yuguangia ordinata*	Yes
Lycopsida?	Incertae Sedis	*Haplostigma*	*Haplostigma* sp.	No
Pteropsida	Psilophytales	*Barsassia*	*Barsassia sibirica*	No
Pteropsida	Psilophytales	*Psilophytites*	*Psilophytites* sp.	No
Pteropsida	Psilophytales	*Psilophytites*	*Psilophytites* sp.	
Pteropsida	Psilophytales	*Psilophytites*	*Psilophytites* sp.	
Pteropsida	Psilophytales	*Psilophytites*	*Psilophytites* sp.1	
Pteropsida	Psilophytales	*Psilophytites*	*Psilophytites* sp.2	
Pteropsida	Psilophytales	cf. *Psilophytites*	cf. *Psilophytites* sp.	
Pteropsida	Psilophytales	*Psilophytites*	*Psilophytites?* sp.	
Pteropsida	Psilophytales	*Psilophytites*	*Psilophytites?* sp.	
Pteropsida	Psilophytales	*Psilophyton*	*Psilophyton bellum*	No
Pteropsida	Psilophytales	*Psilophyton*	*Psilophyton* sp.	
Pteropsida	Psilophytales	*Psilophyton*	*Psilophyton* sp.	
Pteropsida	Psilophytales	*Psilophyton*	*Psilophyton* sp.B	
Pteropsida	Psilophytales	*Psilophyton?*	*Psilophyton? jiangxiensis*	
Pteropsida	Psilophytales	*Psilophyton?*	*Psilophyton? striatum*	
Pteropsida	Incertae Sedis	*Eocladoxylon*	*Eocladoxylon minutum*	Yes
Pteropsida	Incertae Sedis	*Kunia*	*Kunia vernusta*	Yes
Pteropsida	Incertae Sedis	*Protopteridophyton*	*Protopteridophyton devonicum*	Yes
Pteropsida	Incertae Sedis	*Tauritheca*	*Tauritheca cornuta*	Yes
Pteropsida	Incertae Sedis	*Tsaia*	*Tsaia denticulata*	Yes
Pteropsida	Cladoxylopsida	*Panxia*	*Panxia gabata*	Yes

Pteropsida	Cladoxylopsida	*Rhipidophyton*	*Rhipidophyton acanthum*	Yes
Pteropsida	Cladoxylopsida	*Rhipidophyton*	*Rhipidophyton* sp.	
Pteropsida	Cladoxylopsida	*Pseudosporochnus?*	*Pseudosporochnus? heteroramis*	
Pteropsida	Cladoxylopsida	*Pseudosporochnus?*	*Pseudosporochnus?* sp.	
Sphenopsida		*Xihuphyllum*	*Xihuphyllum* sp.	Yes
foliage	Incertae Sedis	*Platyphyllum*	*Platyphyllum* cf. *fuellingii*	No
Progymnospermopsida	Aneurophytales	*Protopteridium*	*Protopteridium scharyanum*	No
Progymnospermopsida	Aneurophytales	*Protopteridium*	*Protopteridium thomsonii*	
Progymnospermopsida	Aneurophytales	*Protopteridium?*	*Protopteridium? minutum*	
Progymnospermopsida	Archaeopteridales	*Archaeopteris*	*Archaeopteris* sp.	No
gymnosperms	Ginkgoaceae	*Baiera?*	*Baiera? minor*	
gymnosperms	Ginkgoaceae	*Sphenobaiera?*	*Sphenobaiera?* sp.	
gymnosperms	Czekanowskiales	*Czekanowskia?*	*Czekanowskia? zhoupingensis*	
gymnosperms	Incertae Sedis	*Archaeosperma*	*Archaeosperma* sp.	No
Incertae Sedis	Taeniocradaceae	*Taeniocrada*	*Taeniocrada decheniana*	No
Incertae Sedis	Taeniocradaceae	*Taeniocrada*	*Taeniocrada* sp.	
Incertae Sedis	Taeniocradaceae	*Taeniocrada*	*Taeniocrada* sp.	
Incertae Sedis	Taeniocradaceae	*Taeniocrada?*	*Taeniocrada? decheniana*	
Incertae Sedis	Barrandeinaceae	*Barrandeina*	*Barrandeina* cf. *dusliana*	No
Incertae Sedis	Barrandeinaceae	*Barrandeina*	*Barrandeina contigua*	
Incertae Sedis	Barrandeinaceae	*Barrandeina*	*Barrandeina dusliana*	
Incertae Sedis	Barrandeinaceae	*Barrandeina*	*Barrandeina laxa*	
Incertae Sedis	Barrandeinaceae	*Barrandeina*	*Barrandeina lixianensis*	
Incertae Sedis	Barrandeinaceae	*Barrandeina*	*Barrandeina multistriata*	
Incertae Sedis	Incertae Sedis	*Amplectosporangium?*	*Amplectosporangium? jiangyouense*	
"Lazarus taxa"			*Drepanophycus spinosus*	
**Frasnian**				
Class	Order/Family/Group	Genus	Species	Endemic genus or not
Lycopsida	Protolepidodendrales	*Minarodendron*	*Minarodendron* cf. *cathaysiense*	Yes
Lycopsida	Isoëtales *sensu lato*	*Chamaedendron*	*Chamaedendron multisporangiatum*	Yes
Lycopsida	Incertae Sedis	cf. *Longostachys*	cf. *Longostachys* sp.	
Lycopsida	Isoëtales *sensu lato*	*Aspidiaria*	*Aspidiaria* sp.	No
Lycopsida	Isoëtales *sensu lato*	*Knorria*	*Knorria* sp.	No
Lycopsida	Isoëtales *sensu lato*	*Knorria?*	*Knorria?* sp.	
Lycopsida	Isoëtales *sensu lato*	*Cyclostigma*	*Cyclostigma kiltorkense*	No
Lycopsida	Incertae Sedis	*Lepidodendropsis*	*Lepidodendropsis arborescens*	No
Lycopsida	Incertae Sedis	*Lepidodendropsis*	*Lepidodendropsis* cf. *theodori*	
Lycopsida	Incertae Sedis	*Lepidodendropsis*	*Lepidodendropsis hirmeri*	
Lycopsida	Incertae Sedis	*Lepidodendropsis*	*Lepidodendropsis* sp.	
Lycopsida	Incertae Sedis	*Lepidodendropsis*	*Lepidodendropsis theodori*	
Lycopsida	Incertae Sedis	*Lepidodendropsis*	*Lepidodendropsis yangtziensis*	
Lycopsida	Incertae Sedis	*Lepidodendropsis?*	*Lepidodendropsis?* sp.	
Lycopsida	Incertae Sedis	*Lepidodendropsis?*	*Lepidodendropsis?* sp.	

Lycopsida	Isoëtales *sensu lato*	*Lepidosigillaria?*	*Lepidosigillaria? sichuanensis*	
Lycopsida	Isoëtales *sensu lato*	*Lepidostrobus?*	*Lepidostrobus?* sp.	
Lycopsida	Isoëtales *sensu lato*	*Leptophloeum*	*Leptophloeum rhombicum*	No
Lycopsida	Isoëtales *sensu lato*	*Leptophloeum*	*Leptophloeum suzhoense*	
Lycopsida	Isoëtales *sensu lato*	*Stigmaria*	*Stigmaria ficoides*	No
Lycopsida	Isoëtales *sensu lato*	*Sublepidodendron*	*Sublepidodendron grabaui*	No
Lycopsida	Isoëtales *sensu lato*	*Sublepidodendron*	*Sublepidodendron mirabile*	
Lycopsida	Isoëtales *sensu lato*	*Sublepidodendron*	*Sublepidodendron songziense*	
Lycopsida	Isoëtales *sensu lato*	*Sublepidodendron*	*Sublepidodendron* sp.	
Lycopsida	Isoëtales *sensu lato*	*Sublepidodendron*	*Sublepidodendron wuhanense*	
Lycopsida	Isoëtales *sensu lato*	*Syringodendron*	*Syringodendron hanyangense*	No
Lycopsida	Isoëtales *sensu lato*	*Syringodendron*	*Syringodendron* sp.1	
Lycopsida	Isoëtales *sensu lato*	*Syringodendron*	*Syringodendron* sp.2	
Lycopsida	Archaeosigillariaceae	*Gilboaphyton?*	*Gilboaphyton? changyangense*	
Lycopsida	Archaeosigillariaceae	*Gilboaphyton?*	*Gilboaphyton? goldringiae*	
Lycopsida	Incertae Sedis	*Cyperites?*	*Cyperites?* sp.	
Lycopsida	Incertae Sedis	*Sphinxiocarpon*	*Sphinxiocarpon wuhanium*	Yes
Pteropsida	Cladoxylopsida	*Metacladophyton*	*Metacladophyton tetraxylum*	Yes
Pteropsida	Cladoxylopsida	*Metacladophyton*	*Metacladophyton ziguinum*	
Pteropsida	Cladoxylopsida	cf. *Metacladophyton*	cf. *Metacladophyton* sp.	
Pteropsida	Cladoxylopsida	*Polypetalophyton*	*Polypetalophyton wufengensis*	Yes
Pteropsida	Incertae Sedis	*Denglongia*	*Denglongia hubeiensis*	Yes
Pteropsida	Incertae Sedis	*Protopteridophyton*	*Protopteridophyton devonicum*	Yes
Pteropsida	Incertae Sedis	*Rhacophyton*	*Rhacophyton* sp.	No
Pteropsida	Incertae Sedis	*Rhacophyton?*	*Rhacophyton? ceratangium*	
Pteropsida	Incertae Sedis	*Tenuisa*	*Tenuisa frasniana*	Yes
Sphenopsida		*Archaeocalamites*	*Archaeocalamites radiates*	No
Sphenopsida		*Archaeocalamites?*	*Archaeocalamites? longiternodus*	
Sphenopsida		*Archaeocalamites?*	*Archaeocalamites?* sp.	
Sphenopsida		*Asterocalamites*	*Asterocalamites* sp.	No
Sphenopsida		*Hamatophyton*	*Hamatophyton verticillatum*	Yes
Sphenopsida		*Sphenophyllum?*	*Sphenophyllum?* sp.	
foliage	Sphenopterids	*Sphenopteridium*	*Sphenopteridium* sp.	No
foliage	Sphenopterids	*Sphenopteris*	*Sphenopteris recurva?*	No
foliage	Sphenopterids	*Sphenopteris?*	*Sphenopteris? recurva*	
foliage	Incertae Sedis	*Platyphyllum*	*Platyphyllum* cf. *williamsonii*	No
foliage	Incertae Sedis	*Platyphyllum*	*Platyphyllum ginkgophylloides*	
Progymnospermopsida	Archaeopteridales	*Archaeopteris*	*Archaeopteris macilenta*	No
gymnosperms	Cordaitales	*Cordaites?*	*Cordaites?* sp.	
Incertae Sedis	Incertae Sedis	*Aphyllopteris?*	*Aphyllopteris?* sp.	
Incertae Sedis	Incertae Sedis	*Changyanophyton*	*Changyanophyton hupiense*	Yes
"Lazarus taxa"		*Drepanophycus*	*Drepanophycus qujingensis*	
"Lazarus taxa"		*Drepanophycus*	*Drepanophycus spinosus*	

"Lazarus taxa"			*Lepidodendropsis* cf. *hirmeri*	
"Lazarus taxa"			*Lepidodendropsis guanzhuangensis*	
**Famennian**				
Class	Order/Family/Group	Genus	Species	Endemic genus or not
Zosterophyllopsida	Barinophytaceae	*Barinophyton*	*Barinophyton citrulliformie*?	
Lycopsida	Drepanophycales	*Drepanophycus*	*Drepanophycus spinaeformis*	No
Lycopsida	Drepanophycales	*Drepanophycus*	*Drepanophycus spinosus*	
Lycopsida	Protolepidodendrales	*Archaeosigillaria?*	*Archaeosigillaria?* sp.	
Lycopsida	Protolepidodendrales	*Archaeosigillaria?*	*Archaeosigillaria? vanuxemi*	
Lycopsida	Protolepidodendrales	*Eolepidodendron*	*Eolepidodendron* cf. *wusihense*	Yes
Lycopsida	Protolepidodendrales	*Eolepidodendron*	*Eolepidodendron densata?*	
Lycopsida	Protolepidodendrales	*Eolepidodendron*	*Eolepidodendron hushanense*	
Lycopsida	Protolepidodendrales	*Eolepidodendron*	*Eolepidodendron* sp.	
Lycopsida	Protolepidodendrales	*Eolepidodendron*	*Eolepidodendron timenense*?	
Lycopsida	Protolepidodendrales	*Eolepidodendron*	*Eolepidodendron volidum?*	
Lycopsida	Protolepidodendrales	*Eolepidodendron*	*Eolepidodendron wusihense*	
Lycopsida	Protolepidodendrales	*Hubeiia*	*Hubeiia dicrofollia*	Yes
Lycopsida	Protolepidodendrales	*Protolepidodendron?*	*Protolepidodendron*? sp.	
Lycopsida	Incertae Sedis	*Monilistrobus*	*Monilistrobus yixingensis*	Yes
Lycopsida	Incertae Sedis	*Wuxia*	*Wuxia bistrobilata*	Yes
Lycopsida	Isoëtales *sensu lato*	*Bothrodendron*	*Bothrodendron (Cyclostigma) anhuiense*	No
Lycopsida	Isoëtales *sensu lato*	*Bothrodendron*	*Bothrodendron (Cyclostigma)* cf. *kiltorkense*	
Lycopsida	Isoëtales *sensu lato*	*Bothrodendron*	*Bothrodendron (Cyclostigma)* sp.1	
Lycopsida	Isoëtales *sensu lato*	*Bothrodendron*	*Bothrodendron (Cyclostigma)* sp.2	
Lycopsida	Isoëtales *sensu lato*	*Cyclostigma*	*Cyclostigma chongyiense*	No
Lycopsida	Isoëtales *sensu lato*	*Cyclostigma*	*Cyclostigma hunanense*	
Lycopsida	Isoëtales *sensu lato*	*Cyclostigma*	*Cyclostigma kiltorkense*	
Lycopsida	Isoëtales *sensu lato*	*Cyclostigma?*	*Cyclostigma*? *yiduense*	
Lycopsida	Isoëtales *sensu lato*	*Knorria*	*Knorria* sp.	No
Lycopsida	Isoëtales *sensu lato*	*Knorria?*	*Knorria*? sp.	
Lycopsida	Isoëtales *sensu lato*	*Lepidodendron*	*Lepidodendron hirmeri*	No
Lycopsida	Isoëtales *sensu lato*	*Lepidodendron*	*Lepidodendron procurrens*	
Lycopsida	Isoëtales *sensu lato*	*Lepidodendron*	*Lepidodendron* sp.	
Lycopsida	Isoëtales *sensu lato*	*Lepidodendron*	*Lepidodendron* sp.	
Lycopsida	Isoëtales *sensu lato*	*Lepidodendron*	*Lepidodendron* sp.	
Lycopsida	Isoëtales *sensu lato*	*Lepidodendron*	*Lepidodendron* sp.	
Lycopsida	Isoëtales *sensu lato*	*Lepidodendron?*	*Lepidodendron*? sp.	
Lycopsida	Incertae Sedis	*Lepidodendropsis*	*Lepidodendropsis* cf. *hirmeri*	No
Lycopsida	Incertae Sedis	*Lepidodendropsis*	*Lepidodendropsis* cf. *scobiniformis*	
Lycopsida	Incertae Sedis	*Lepidodendropsis*	*Lepidodendropsis cyclostigmatoides*	
Lycopsida	Incertae Sedis	*Lepidodendropsis*	*Lepidodendropsis guanzhuangensis*	

Lycopsida	Incertae Sedis	*Lepidodendropsis*	*Lepidodendropsis hirmeri*	
Lycopsida	Incertae Sedis	*Lepidodendropsis*	*Lepidodendropsis scobiniformis*	
Lycopsida	Incertae Sedis	*Lepidodendropsis*	*Lepidodendropsis* sp.	
Lycopsida	Incertae Sedis	*Lepidodendropsis*	*Lepidodendropsis* sp.	
Lycopsida	Incertae Sedis	*Lepidodendropsis*	*Lepidodendropsis* sp.	
Lycopsida	Incertae Sedis	*Lepidodendropsis*	*Lepidodendropsis* sp.	
Lycopsida	Incertae Sedis	*Lepidodendropsis*	*Lepidodendropsis* sp.	
Lycopsida	Incertae Sedis	*Lepidodendropsis*	*Lepidodendropsis* sp.	
Lycopsida	Incertae Sedis	*Lepidodendropsis*	*Lepidodendropsis theodori*	
Lycopsida	Isoëtales *sensu lato*	*Lepidophyllum*	*Lepidophyllum (Cantheliophorus)* cf. *mirabile*	No
Lycopsida	Isoëtales *sensu lato*	*Lepidophyllum*	*Lepidophyllum* sp.	
Lycopsida	Isoëtales *sensu lato*	*Lepidophyllum*	*Lepidophyllum xiphidium*	
Lycopsida	Isoëtales *sensu lato*	*Lepidophyllum?*	*Lepidophyllum*? sp.	
Lycopsida	Isoëtales *sensu lato*	*Lepidostrobophyllum*	*Lepidostrobophyllum* sp.	No
Lycopsida	Isoëtales *sensu lato*	*Lepidostrobophyllum*	*Lepidostrobophyllum* sp.	
Lycopsida	Isoëtales *sensu lato*	*Lepidostrobophyllum*	*Lepidostrobophyllum* sp.	
Lycopsida	Isoëtales *sensu lato*	*Lepidostrobophyllum*	*Lepidostrobophyllum xiphidium*	
Lycopsida	Isoëtales *sensu lato*	*Lepidostrobus*	*Lepidostrobus* aff. *ugulatus*	No
Lycopsida	Isoëtales *sensu lato*	*Lepidostrobus*	*Lepidostrobus hineri*	
Lycopsida	Isoëtales *sensu lato*	*Lepidostrobus*	*Lepidostrobus* sp.	
Lycopsida	Isoëtales *sensu lato*	*Lepidostrobus*	*Lepidostrobus* sp.	
Lycopsida	Isoëtales *sensu lato*	*Lepidostrobus*	*Lepidostrobus* sp.	
Lycopsida	Isoëtales *sensu lato*	*Lepidostrobus*	*Lepidostrobus* sp.	
Lycopsida	Isoëtales *sensu lato*	*Lepidostrobus*	*Lepidostrobus ungulatus*	
Lycopsida	Isoëtales *sensu lato*	*Lepidostrobus*	*Lepidostrobus wufengensis*	
Lycopsida	Isoëtales *sensu lato*	*Leptophloeum*	*Leptophloeum rhombicum*	No
Lycopsida	Isoëtales *sensu lato*	*Leptophloeum*	*Leptophloeum suzhouense*	
Lycopsida	Isoëtales *sensu lato*	*Minostrobus*	*Minostrobus chaohuensis*	Yes
Lycopsida	Isoëtales *sensu lato*	*Stigmaria*	*Stigmaria* cf. *radiato-punctata*	No
Lycopsida	Isoëtales *sensu lato*	*Stigmaria*	*Stigmaria ficoides*	
Lycopsida	Isoëtales *sensu lato*	*Stigmaria*	*Stigmaria radiato-punctata*	
Lycopsida	Isoëtales *sensu lato*	*Stigmaria*	*Stigmaria rugulosa*	
Lycopsida	Isoëtales *sensu lato*	*Stigmaria*	*Stigmaria* sp.	
Lycopsida	Isoëtales *sensu lato*	*Sublepidodendron*	*Sublepidodendron* cf. *grabaui*	No
Lycopsida	Isoëtales *sensu lato*	*Sublepidodendron*	*Sublepidodendron* cf. *mirabile*	
Lycopsida	Isoëtales *sensu lato*	*Sublepidodendron*	*Sublepidodendron* cf. *xinjiangense*	
Lycopsida	Isoëtales *sensu lato*	*Sublepidodendron*	*Sublepidodendron grabaui*	
Lycopsida	Isoëtales *sensu lato*	*Sublepidodendron*	*Sublepidodendron mirabile*	
Lycopsida	Isoëtales *sensu lato*	*Sublepidodendron*	*Sublepidodendron shimenense*	
Lycopsida	Isoëtales *sensu lato*	*Sublepidodendron*	*Sublepidodendron songziense*	
Lycopsida	Isoëtales *sensu lato*	*Sublepidodendron*	*Sublepidodendron* sp.	
Lycopsida	Isoëtales *sensu lato*	*Sublepidodendron*	*Sublepidodendron* sp.	

Lycopsida	Isoëtales *sensu lato*	*Sublepidodendron*	*Sublepidodendron* sp.	
Lycopsida	Isoëtales *sensu lato*	*Sublepidodendron*	*Sublepidodendron* sp.	
Lycopsida	Isoëtales *sensu lato*	*Sublepidodendron*	*Sublepidodendron* sp.	
Lycopsida	Isoëtales *sensu lato*	*Sublepidodendron*	*Sublepidodendron* sp.	
Lycopsida	Isoëtales *sensu lato*	*Sublepidodendron*	*Sublepidodendron* sp.	
Lycopsida	Isoëtales *sensu lato*	*Sublepidodendron*	*Sublepidodendron* sp.	
Lycopsida	Isoëtales *sensu lato*	*Sublepidodendron*	*Sublepidodendron* sp.	
Lycopsida	Isoëtales *sensu lato*	*Sublepidodendron*	*Sublepidodendron* sp.?	
Lycopsida	Isoëtales *sensu lato*	*Sublepidodendron*	*Sublepidodendron* sp.1	
Lycopsida	Isoëtales *sensu lato*	*Sublepidodendron*	*Sublepidodendron* sp.2	
Lycopsida	Isoëtales *sensu lato*	*Sublepidodendron*	*Sublepidodendron* sp.3	
Lycopsida	Isoëtales *sensu lato*	*Sublepidodendron*	*Sublepidodendron* spp.	
Lycopsida	Isoëtales *sensu lato*	*Sublepidodendron*	*Sublepidodendron taihuensis*	
Lycopsida	Isoëtales *sensu lato*	*Sublepidodendron*	*Sublepidodendron xiaoshanense*	
Lycopsida	Isoëtales *sensu lato*	*Sublepidodendron*	*Sublepidodendron yichangense*	
Lycopsida	Isoëtales *sensu lato*	*Sublepidodendron?*	*Sublepidodendron*? *grabaui*	
Lycopsida	Isoëtales *sensu lato*	*Sublepidodendron?*	*Sublepidodendron*? sp.	
Lycopsida	Isoëtales *sensu lato*	*Sublepidodendron?*	*Sublepidodendron*? sp.	
Lycopsida	Incertae Sedis	*Spinolepidodendron*	*Spinolepidodendron hangzhouense*	Yes
Lycopsida	Incertae Sedis	*Spinolepidodendron*	*Spinolepidodendron* sp.	
Lycopsida	Incertae Sedis	*Spinolepidodendron*	*Spinolepidodendron xiaoshanense*	
Pteropsida	Incertae Sedis	*Rhacophyton?*	*Rhacophyton*? sp.	
Sphenopsida		*Archaeocalamites*	*Archaeocalamites* sp.	No
Sphenopsida		*Asterocalamites*	*Asterocalamites* aff. *scrobiculatus*	No
Sphenopsida		*Calamostachys*	*Calamostachys hunanensis?*	No
Sphenopsida		*Calamostachys*	*Calamostachys* sp.	
Sphenopsida		*Eviostachya*	*Eviostachya hoegii*	No
Sphenopsida		*Hamatophyton*	*Hamatophyton* sp.	Yes
Sphenopsida		*Hamatophyton*	*Hamatophyton* sp.	
Sphenopsida		*Hamatophyton*	*Hamatophyton verticillatum*	
Sphenopsida		*Palaeostachya*	*Palaeostachya shimenensis*	No
Sphenopsida		*Rotafolia*	*Rotafolia songziensis*	Yes
Sphenopsida		*Sphenophyllum*	*Sphenophyllum guangzhouensis*	No
Sphenopsida		*Sphenophyllum*	*Sphenophyllum lungtanense*	
Sphenopsida		*Sphenophyllum*	*Sphenophyllum megalofolium*	
Sphenopsida		*Sphenophyllum*	*Sphenophyllum pseudotenerrimum*	
Sphenopsida		*Sphenophyllum*	*Sphenophyllum* sp.	
Sphenopsida		*Sphenophyllum?*	*Sphenophyllum*? *changshaense*	
Sphenopsida		*Xihuphyllum*	*Xihuphyllum elongatum*	Yes
Sphenopsida		*Xihuphyllum*	*Xihuphyllum megalofolium*	
foliage	Sphenopterids	*Sphenopteridium?*	*Sphenopteridium*? sp.a	
foliage	Sphenopterids	*Sphenopteridium?*	*Sphenopteridium*? sp.b	
foliage	Sphenopterids	*Sphenopteris*	*Sphenopteris* cf. *taihuensis*	No

foliage	Sphenopterids	*Sphenopteris*	*Sphenopteris* sp.	
foliage	Sphenopterids	*Sphenopteris*	*Sphenopteris* sp.	
foliage	Sphenopterids	*Sphenopteris*	*Sphenopteris* sp.	
foliage	Sphenopterids	*Sphenopteris*	*Sphenopteris taihuensis*	
foliage	Sphenopterids	*Sphenopteris?*	*Sphenopteris*? *recurva*	
foliage	Incertae Sedis	*Platyphyllum*	*Platyphyllum* sp.	No
foliage	Incertae Sedis	*Platyphyllum*	*Platyphyllum* sp.	
foliage	Incertae Sedis	*Platyphyllum*	*Platyphyllum* sp.	
foliage	Incertae Sedis	*Platyphyllum*	*Platyphyllum subreiculatum*	
foliage	Incertae Sedis	*Platyphyllum?*	*Platyphyllum*? cf. *williamsonii*	
Progymnospermopsida	Aneurophytales	*Protopteridium?*	*Protopteridium*? *minutum*	
Progymnospermopsida	Archaeopteridales	*Archaeopteris*	*Archaeopteris* cf. *macilenta*	No
Progymnospermopsida	Archaeopteridales	*Archaeopteris*	*Archaeopteris* cf. *sphenophyllifolia*	
Progymnospermopsida	Archaeopteridales	*Archaeopteris*	*Archaeopteris halliana*	
Progymnospermopsida	Archaeopteridales	*Archaeopteris*	*Archaeopteris macillenta*	
Progymnospermopsida	Archaeopteridales	*Archaeopteris*	*Archaeopteris mutatoformis*	
Progymnospermopsida	Archaeopteridales	*Archaeopteris*	*Archaeopteris roemeriana*	
Progymnospermopsida	Archaeopteridales	*Archaeopteris*	*Archaeopteris* sp.	
Progymnospermopsida	Archaeopteridales	*Archaeopteris*	*Archaeopteris* sp.	
Progymnospermopsida	Archaeopteridales	*Archaeopteris*	*Archaeopteris* sp.	
Progymnospermopsida	Archaeopteridales	*Archaeopteris*	*Archaeopteris* sp.	
Progymnospermopsida	Archaeopteridales	*Archaeopteris*	*Archaeopteris* sp.	
Progymnospermopsida	Archaeopteridales	*Archaeopteris*	*Archaeopteris* sp.1	
Progymnospermopsida	Archaeopteridales	*Archaeopteris*	*Archaeopteris* sp.2	
Progymnospermopsida	Archaeopteridales	*Archaeopteris*	*Archaeopteris tonglingiana*	
Progymnospermopsida	Archaeopteridales	*Archaeopteris*	*Archaeopteris zhongmingiana*	
gymnosperms	Cordaitales	*Cordaites*	*Cordaites* sp.	No
gymnosperms	Incertae Sedis	*Carpolithus*	*Carpolithus* sp.	No
gymnosperms	Incertae Sedis	*Carpolithus*	*Carpolithus* sp.	
gymnosperms	Incertae Sedis	*Sphenophyllostachys?*	*Sphenophyllostachys*? sp.	
Incertae Sedis	Taeniocradaceae	*Taeniocrada?*	*Taeniocrada*? sp.	
Incertae Sedis	Incertae Sedis	*Kongshania*	*Kongshania synangioides*	Yes
Incertae Sedis	Incertae Sedis	*Yichangophyton?*	*Yichangophyton*? *guanzuangense*	
"Lazarus taxa"			*Archaeopteris macilenta*	
"Lazarus taxa"		*Aspidiaria*	*Aspidiaria* sp.	
"Lazarus taxa"			*Lepidodendropsis* cf. *hirmeri*	
**Tournaisian1**				
Class	Order/Family/Group	Genus	Species	Endemic genus or not
Lycopsida	Protolepidodendrales	*Eolepidodendron*	*Eolepidodendron* cf. *wusihense*	Yes
Lycopsida	Protolepidodendrales	*Eolepidodendron*	*Eolepidodendron hushanense*	
Lycopsida	Protolepidodendrales	*Eolepidodendron*	*Eolepidodendron wusihense*	
Lycopsida	Protolepidodendrales	*Protolepidodendron?*	*Protolepidodendron*? sp.	
Lycopsida	Isoëtales *sensu lato*	*Cyclostigma*	*Cyclostigma kiltorkense*	No

Lycopsida	Isoëtales *sensu lato*	*Knorria?*	*Knorria*? sp.	
Lycopsida	Isoëtales *sensu lato*	*Lepidodendron*	*Lepidodendron procurrens*	No
Lycopsida	Isoëtales *sensu lato*	*Lepidodendron*	*Lepidodendron* sp.	
Lycopsida	Isoëtales *sensu lato*	*Lepidodendron*	*Lepidodendron* sp.	
Lycopsida	Incertae Sedis	*Lepidodendropsis*	*Lepidodendropsis* cf. *hirmeri*	No
Lycopsida	Incertae Sedis	*Lepidodendropsis*	*Lepidodendropsis hirmeri*	
Lycopsida	Incertae Sedis	*Lepidodendropsis*	*Lepidodendropsis scobiniformis*	
Lycopsida	Incertae Sedis	*Lepidodendropsis*	*Lepidodendropsis* sp.	
Lycopsida	Incertae Sedis	*Lepidodendropsis*	*Lepidodendropsis* sp.	
Lycopsida	Isoëtales *sensu lato*	*Lepidophyllum*	*Lepidophyllum (Cantheliophorus)* cf. *mirabile*	No
Lycopsida	Isoëtales *sensu lato*	*Lepidophyllum*	*Lepidophyllum xiphidium*	
Lycopsida	Isoëtales *sensu lato*	*Lepidostrobophyllum*	*Lepidostrobophyllum* sp.	No
Lycopsida	Isoëtales *sensu lato*	*Lepidostrobophyllum*	*Lepidostrobophyllum* sp.	
Lycopsida	Isoëtales *sensu lato*	*Lepidostrobophyllum*	*Lepidostrobophyllum xiphidium*	
Lycopsida	Isoëtales *sensu lato*	*Lepidostrobus*	*Lepidostrobus* sp.	No
Lycopsida	Isoëtales *sensu lato*	*Lepidostrobus*	*Lepidostrobus ungulatus*	
Lycopsida	Isoëtales *sensu lato*	*Leptophloeum*	*Leptophloeum rhombicum*	No
Lycopsida	Isoëtales *sensu lato*	*Leptophloeum*	*Leptophloeum suzhouense*	
Lycopsida	Isoëtales *sensu lato*	*Stigmaria*	*Stigmaria ficoides*	No
Lycopsida	Isoëtales *sensu lato*	*Stigmaria*	*Stigmaria radiatopunctata*	
Lycopsida	Isoëtales *sensu lato*	*Stigmaria*	*Stigmaria rugulosa*	
Lycopsida	Isoëtales *sensu lato*	*Stigmaria*	*Stigmaria* sp.	
Lycopsida	Isoëtales *sensu lato*	*Sublepidodendron*	*Sublepidodendron* cf. *grabaui*	No
Lycopsida	Isoëtales *sensu lato*	*Sublepidodendron*	*Sublepidodendron* cf. *mirabile*	
Lycopsida	Isoëtales *sensu lato*	*Sublepidodendron*	*Sublepidodendron grabaui*	
Lycopsida	Isoëtales *sensu lato*	*Sublepidodendron*	*Sublepidodendron mirabile*	
Lycopsida	Isoëtales *sensu lato*	*Sublepidodendron*	*Sublepidodendron songziense*	
Lycopsida	Isoëtales *sensu lato*	*Sublepidodendron*	*Sublepidodendron* sp.	
Lycopsida	Isoëtales *sensu lato*	*Sublepidodendron*	*Sublepidodendron* sp.	
Lycopsida	Isoëtales *sensu lato*	*Sublepidodendron*	*Sublepidodendron* sp.	
Lycopsida	Isoëtales *sensu lato*	*Sublepidodendron*	*Sublepidodendron* sp.	
Lycopsida	Isoëtales *sensu lato*	*Sublepidodendron*	*Sublepidodendron* sp.?	
Lycopsida	Isoëtales *sensu lato*	*Sublepidodendron*	*Sublepidodendron xiaoshanense*	
Lycopsida	Isoëtales *sensu lato*	*Sublepidodendron?*	*Sublepidodendron*? *grabaui*	
Lycopsida	Incertae Sedis	*Spinolepidodendron*	*Spinolepidodendron hangzhouense*	Yes
Lycopsida	Incertae Sedis	*Spinolepidodendron*	*Spinolepidodendron* sp.	
Lycopsida	Incertae Sedis	*Spinolepidodendron*	*Spinolepidodendron xiaoshanense*	
Pteropsida	Stauropteridales	*Multifurcatus*	*Multifurcatus tenellus*	Yes
Pteropsida	Incertae Sedis	*Coenosophyton*	*Coenosophyton tristichus*	Yes
Pteropsida	Incertae Sedis	*Helicophyton*	*Helicophyton dichotomum*	Yes
Pteropsida	Incertae Sedis	*Rhacophyton*	*Rhacophyton* sp.	No

Sphenopsida		*Archaeocalamites*	*Archaeocalamites scrobiculatus*	No
Sphenopsida		*Archaeocalamites*	*Archaeocalamites* sp.	
Sphenopsida		*Eviostachya*	*Eviostachya hoegii*	No
Sphenopsida		*Hamatophyton*	*Hamatophyton* sp.	Yes
Sphenopsida		*Hamatophyton*	*Hamatophyton verticillatum*	
Sphenopsida		*Sphenophyllum*	*Sphenophyllum lungtanense*	No
Sphenopsida		*Sphenophyllum*	*Sphenophyllum pseudotenerrimum*	
Sphenopsida		*Sphenophyllum*	*Sphenophyllum* sp.	
Sphenopsida		*Sphenophyllum*	*Sphenophyllum* sp.	
Sphenopsida		*Xihuphyllum*	*Xihuphyllum elongatum*	Yes
Sphenopsida		*Xihuphyllum*	*Xihuphyllum megalofolium*	
foliage	Sphenopterids	*Rhodeopteridium*	*Rhodeopteridium* cf. *hsianghsiangense*	No
foliage	Sphenopterids	*Rhodeopteridium*	*Rhodeopteridium* sp.	
foliage	Sphenopterids	*Sphenopteridium?*	*Sphenopteridium*? sp.	
foliage	Sphenopterids	*Sphenopteris*	*Sphenopteris* sp.	No
foliage	Sphenopterids	*Sphenopteris*	*Sphenopteris taihuensis*	
foliage	Incertae Sedis	*Platyphyllum*	*Platyphyllum* sp.	No
foliage	Incertae Sedis	*Platyphyllum?*	*Platyphyllum*? cf. *williamsonii*	
Progymnospermopsida	Archaeopteridales	*Archaeopteris*	*Archaeopteris macilenta*	No
gymnosperms	Incertae Sedis	*Carpolithus*	*Carpolithus* sp.	No
gymnosperms	Incertae Sedis	*Carpolithus*	*Carpolithus* sp.	
gymnosperms	Incertae Sedis	*Sphenophyllostachys?*	*Sphenophyllostachys*? sp.	
"Lazarus taxa"		*Aspidiaria*	*Aspidiaria* sp.	
"Lazarus taxa"			*Lepidodendropsis* cf. *hirmeri*	
**Tournaisian2**				
Class	Order/Family/Group	Genus	Species	Endemic genus or not
Lycopsida	Protolepidodendrales	*Eolepidodendron*	*Eolepidodendron* cf. *wusihense*	Yes
Lycopsida	Protolepidodendrales	*Eolepidodendron*	*Eolepidodendron wusihense*	
Lycopsida	Isoëtales *sensu lato*	*Bothrodendron*	*Bothrodendron fuyangense*	No
Lycopsida	Incertae Sedis	*Lepidodendropsis*	*Lepidodendropsis* cf. *hirmeri*	No
Lycopsida	Incertae Sedis	*Lepidodendropsis*	*Lepidodendropsis* sp.	
Lycopsida	Isoëtales *sensu lato*	*Sublepidodendron*	*Sublepidodendron grabaui*	No
Lycopsida	Isoëtales *sensu lato*	*Sublepidodendron*	*Sublepidodendron mirabile*	
Lycopsida	Isoëtales *sensu lato*	*Sublepidodendron*	*Sublepidodendron* sp.	
foliage	Sphenopterids	*Rhodeopteridium*	*Rhodeopteridium* sp.	No
foliage	Sphenopterids	*Sphenopteridium*	*Sphenopteridium* sp.	No
"Lazarus taxa"		*Archaeocalamites*	*Archaeocalamites scrobiculatus*	
"Lazarus taxa"		*Aspidiaria*	*Aspidiaria* sp.	
"Lazarus taxa"		*Carpolithus*	*Carpolithus* sp.	
"Lazarus taxa"		*Hamatophyton*	*Hamatophyton verticillatum*	
"Lazarus taxa"		*Knorria*	*Knorria* sp.	

"Lazarus taxa"			*Lepidodendropsis hirmeri*	
"Lazarus taxa"		*Sphenophyllum*	*Sphenophyllum pseudotenerrimum*	
"Lazarus taxa"		*Stigmaria*	*Stigmaria ficoides*	
"Lazarus taxa"		*Stigmaria*	*Stigmaria rugulosa*	
"Lazarus taxa"			*Sublepidodendron songziense*	
**Visean**				
Class	Order/Family/Group	Genus	Species	Endemic genus or not
Lycopsida	Protolepidodendrales	*Eolepidodendron*	*Eolepidodendron* cf. *wusihense*	Yes
Lycopsida	Protolepidodendrales	*Eolepidodendron*	*Eolepidodendron changyangense?*	
Lycopsida	Protolepidodendrales	*Eolepidodendron*	*Eolepidodendron jurongense*	
Lycopsida	Protolepidodendrales	*Eolepidodendron*	*Eolepidodendron nathorsti*	
Lycopsida	Protolepidodendrales	*Eolepidodendron*	*Eolepidodendron* sp.	
Lycopsida	Isoëtales *sensu lato*	*Bothrodendron*	*Bothrodendron circulare*	No
Lycopsida	Isoëtales *sensu lato*	*Bothrodendron*	*Bothrodendron flabellatum*	
Lycopsida	Isoëtales *sensu lato*	*Bothrodendron*	*Bothrodendron ruchengense*	
Lycopsida	Isoëtales *sensu lato*	*Bothrodendron*	*Bothrodendron* sp.	
Lycopsida	Isoëtales *sensu lato*	*Bothrodendron*	*Bothrodendron* sp.a	
Lycopsida	Isoëtales *sensu lato*	*Bothrodendron*	*Bothrodendron* sp.b	
Lycopsida	Isoëtales *sensu lato*	*Bothrodendron?*	*Bothrodendron*? *yangshanense*	
Lycopsida	Isoëtales *sensu lato*	*Cathaysiodendron*	*Cathaysiodendron gushiense*	Yes
Lycopsida	Isoëtales *sensu lato*	*Cathaysiodendron*	*Cathaysiodendron yangshanense*	
Lycopsida	Isoëtales *sensu lato*	*Cathaysiodendron?*	*Cathaysiodendron*? sp.	
Lycopsida	Isoëtales *sensu lato*	*Knorria*	*Knorria* sp.	No
Lycopsida	Isoëtales *sensu lato*	*Knorria*	*Knorria* sp.	
Lycopsida	Isoëtales *sensu lato*	*Lepidodendron*	*Lepidodendron* aff. *aolungpylukense*	No
Lycopsida	Isoëtales *sensu lato*	*Lepidodendron*	*Lepidodendron* cf. *aolungpylukense*	
Lycopsida	Isoëtales *sensu lato*	*Lepidodendron*	*Lepidodendron* cf. *canobianum*	
Lycopsida	Isoëtales *sensu lato*	*Lepidodendron*	*Lepidodendron* cf. *robertii*	
Lycopsida	Isoëtales *sensu lato*	*Lepidodendron*	*Lepidodendron* cf. *shanyangense*	
Lycopsida	Isoëtales *sensu lato*	*Lepidodendron*	*Lepidodendron* cf. *subrhombicum*	
Lycopsida	Isoëtales *sensu lato*	*Lepidodendron*	*Lepidodendron* cf. *volkmannianum*	
Lycopsida	Isoëtales *sensu lato*	*Lepidodendron*	*Lepidodendron* cf. *worthenii*	
Lycopsida	Isoëtales *sensu lato*	*Lepidodendron*	*Lepidodendron dabieshanense*	
Lycopsida	Isoëtales *sensu lato*	*Lepidodendron*	*Lepidodendron gaolishanense*	
Lycopsida	Isoëtales *sensu lato*	*Lepidodendron*	*Lepidodendron huashanlingense*	
Lycopsida	Isoëtales *sensu lato*	*Lepidodendron*	*Lepidodendron hunanense*	
Lycopsida	Isoëtales *sensu lato*	*Lepidodendron*	*Lepidodendron jiandeense*	
Lycopsida	Isoëtales *sensu lato*	*Lepidodendron*	*Lepidodendron lengshuijiangense*	
Lycopsida	Isoëtales *sensu lato*	*Lepidodendron*	*Lepidodendron machalaensis*	
Lycopsida	Isoëtales *sensu lato*	*Lepidodendron*	*Lepidodendron quadratum*	
Lycopsida	Isoëtales *sensu lato*	*Lepidodendron*	*Lepidodendron rhodeanum*	
Lycopsida	Isoëtales *sensu lato*	*Lepidodendron*	*Lepidodendron rimosum*	
Lycopsida	Isoëtales *sensu lato*	*Lepidodendron*	*Lepidodendron shanyangense*	

Lycopsida	Isoëtales *sensu lato*	*Lepidodendron*	*Lepidodendron sophoroides*?	
Lycopsida	Isoëtales *sensu lato*	*Lepidodendron*	*Lepidodendron* sp.	
Lycopsida	Isoëtales *sensu lato*	*Lepidodendron*	*Lepidodendron* sp.	
Lycopsida	Isoëtales *sensu lato*	*Lepidodendron*	*Lepidodendron* sp.	
Lycopsida	Isoëtales *sensu lato*	*Lepidodendron*	*Lepidodendron* sp.	
Lycopsida	Isoëtales *sensu lato*	*Lepidodendron*	*Lepidodendron* sp.	
Lycopsida	Isoëtales *sensu lato*	*Lepidodendron*	*Lepidodendron* sp.	
Lycopsida	Isoëtales *sensu lato*	*Lepidodendron*	*Lepidodendron* sp. cf. *quadratum*	
Lycopsida	Isoëtales *sensu lato*	*Lepidodendron*	*Lepidodendron* sp.1	
Lycopsida	Isoëtales *sensu lato*	*Lepidodendron*	*Lepidodendron* sp.2	
Lycopsida	Isoëtales *sensu lato*	*Lepidodendron*	*Lepidodendron* sp.a	
Lycopsida	Isoëtales *sensu lato*	*Lepidodendron*	*Lepidodendron* sp.b	
Lycopsida	Isoëtales *sensu lato*	*Lepidodendron*	*Lepidodendron taoshuiense*	
Lycopsida	Isoëtales *sensu lato*	*Lepidodendron*	*Lepidodendron wengyuanense*	
Lycopsida	Isoëtales *sensu lato*	*Lepidodendron*	*Lepidodendron yuduense*	
Lycopsida	Isoëtales *sensu lato*	*Lepidodendron?*	*Lepidodendron*? sp.	
Lycopsida	Incertae Sedis	*Lepidodendropsis*	*Lepidodendropsis hirmeri*	No
Lycopsida	Incertae Sedis	*Lepidodendropsis*	*Lepidodendropsis shaoyangensis?*	
Lycopsida	Incertae Sedis	*Lepidodendropsis*	*Lepidodendropsis* sp.	
Lycopsida	Incertae Sedis	*Lepidodendropsis*	*Lepidodendropsis* sp.	
Lycopsida	Incertae Sedis	*Lepidodendropsis*	*Lepidodendropsis taoshanensis?*	
Lycopsida	Isoëtales *sensu lato*	*Lepidophyllum*	*Lepidophyllum* sp.	No
Lycopsida	Isoëtales *sensu lato*	*Lepidostrobophyllum*	*Lepidostrobophyllum* cf. *hastatum*	No
Lycopsida	Isoëtales *sensu lato*	*Lepidostrobophyllum*	*Lepidostrobophyllum* cf. *lanceolatum*	
Lycopsida	Isoëtales *sensu lato*	*Lepidostrobophyllum*	*Lepidostrobophyllum* cf. *ovatifolium*	
Lycopsida	Isoëtales *sensu lato*	*Lepidostrobophyllum*	*Lepidostrobophyllum* sp.	
Lycopsida	Isoëtales *sensu lato*	*Lepidostrobophyllum*	*Lepidostrobophyllum* sp.	
Lycopsida	Isoëtales *sensu lato*	*Lepidostrobophyllum*	*Lepidostrobophyllum* sp.1	
Lycopsida	Isoëtales *sensu lato*	*Lepidostrobophyllum*	*Lepidostrobophyllum* sp.2	
Lycopsida	Isoëtales *sensu lato*	*Lepidostrobophyllum*	*Lepidostrobophyllum* sp.3	
Lycopsida	Isoëtales *sensu lato*	*Lepidostrobophyllum*	*Lepidostrobophyllum* spp.	
Lycopsida	Isoëtales *sensu lato*	*Lepidostrobus*	*Lepidostrobus* sp.	No
Lycopsida	Isoëtales *sensu lato*	*Lepidostrobus*	*Lepidostrobus* sp.	
Lycopsida	Isoëtales *sensu lato*	*Lepidostrobus?*	*Lepidostrobus*? sp.	
Lycopsida	Isoëtales *sensu lato*	*Sigillaria*	*Sigillaria brardii*	No
Lycopsida	Isoëtales *sensu lato*	*Stigmaria*	*Stigmaria ficoides*	No
Lycopsida	Isoëtales *sensu lato*	*Stigmaria*	*Stigmaria rugulosa*	
Lycopsida	Isoëtales *sensu lato*	*Stigmaria*	*Stigmaria* sp.	
Lycopsida	Isoëtales *sensu lato*	*Stigmaria*	*Stigmaria* sp.	
Lycopsida	Isoëtales *sensu lato*	*Sublepidodendron*	*Sublepidodendron* cf. *mirabile*	No
Lycopsida	Isoëtales *sensu lato*	*Sublepidodendron*	*Sublepidodendron* cf. *robertii*	
Lycopsida	Isoëtales *sensu lato*	*Sublepidodendron*	*Sublepidodendron changduense*	

Lycopsida	Isoëtales *sensu lato*	*Sublepidodendron*	*Sublepidodendron changyangense?*	
Lycopsida	Isoëtales *sensu lato*	*Sublepidodendron*	*Sublepidodendron grabaui*	
Lycopsida	Isoëtales *sensu lato*	*Sublepidodendron*	*Sublepidodendron mirabile*	
Lycopsida	Isoëtales *sensu lato*	*Sublepidodendron*	*Sublepidodendron songziense*	
Lycopsida	Isoëtales *sensu lato*	*Sublepidodendron*	*Sublepidodendron* sp.	
Lycopsida	Isoëtales *sensu lato*	*Sublepidodendron*	*Sublepidodendron* sp.	
Lycopsida	Isoëtales *sensu lato*	*Sublepidodendron*	*Sublepidodendron tangshanense*	
Lycopsida	Isoëtales *sensu lato*	*Sublepidodendron*	*Sublepidodendron taoshanense?*	
Lycopsida	Isoëtales *sensu lato*	*Sublepidodendron*	*Sublepidodendron wengtanense?*	
Lycopsida	Lycopodiales	*Cantheliophorus*	*Cantheliophorus* sp.	No
Sphenopsida		*Archaeocalamites*	*Archaeocalamites prolixus*	No
Sphenopsida		*Archaeocalamites*	*Archaeocalamites scrobiculatus*	
Sphenopsida		*Archaeocalamites*	*Archaeocalamites* sp.	
Sphenopsida		*Archaeocalamites*	*Archaeocalamites* sp.	
Sphenopsida		*Archaeocalamites*	*Archaeocalamites* sp.	
Sphenopsida		*Archaeocalamites*	*Archaeocalamites* sp.	
Sphenopsida		*Archaeocalamites*	*Archaeocalamites* sp.	
Sphenopsida		*Archaeocalamites*	*Archaeocalamites* sp.	
Sphenopsida		*Asterocalamites*	*Asterocalamites scrobiculatus*	No
Sphenopsida		*Asterocalamites*	*Asterocalamites* sp.	
Sphenopsida		*Asterophyllites*	*Asterophyllites longifolius*	No
Sphenopsida		*Calamites*	*Calamites (?Mesocalamites)* sp.	No
Sphenopsida		*Calamites*	*Calamites* sp.	
Sphenopsida		*Calamostachys?*	*Calamostachys*? sp.	
Sphenopsida		*Hamatophyton*	*Hamatophyton verticillatum*	Yes
Sphenopsida		*Mesocalamites*	*Mesocalamites* sp.	No
Sphenopsida		*Mesocalamites*	*Mesocalamites* sp.	
Sphenopsida		*Mesocalamites*	*Mesocalamites* sp.	
Sphenopsida		*Mesocalamites*	*Mesocalamites* sp.	
Sphenopsida		*Mesocalamites*	*Mesocalamites* sp.	
Sphenopsida		*Mesocalamites*	*Mesocalamites* sp.	
Sphenopsida		*Mesocalamites*	*Mesocalamites* sp.	
Sphenopsida		*Sphenophyllum*	*Sphenophyllum cuneifolium*	No
Sphenopsida		*Sphenophyllum*	*Sphenophyllum geigense?*	
Sphenopsida		*Sphenophyllum*	*Sphenophyllum pseudotenerrimum*	
Sphenopsida		*Sphenophyllum*	*Sphenophyllum* sp.	
Sphenopsida		*Sphenophyllum*	*Sphenophyllum* sp.	
Sphenopsida		*Sphenophyllum*	*Sphenophyllum tenertimum*	
Sphenopsida		*Sphenophyllum*	*Sphenophyllum yuduense*	
Sphenopsida		*Sphenophyllostachys*	*Sphenophyllostachys tenerrimus*	No
Sphenopsida		*Pothocites*	*Pothocites* sp.	No
foliage	Alethopterids	*Lopinopteris*	*Lopinopteris intercalata*	Yes
foliage	Alethopterids	*Psygmophyllum?*	*Psygmophyllum*? sp.	

foliage	Cardiopterids	*Cardiopteris*	*Cardiopteris frongdosa*	No
foliage	Cardiopterids	*Cardiopteris*	*Cardiopteris sp.*	
foliage	Cardiopterids	*Cardiopteris?*	*Cardiopteris*? sp.	
foliage	Cardiopterids	*Cardiopteris?*	*Cardiopteris*? spp.	
foliage	Mariopterids	*Mariopteris*	*Mariopteris acuta*	No
foliage	Mariopterids	*Mariopteris?*	*Mariopteris*? sp.	
foliage	Mariopterids	*Mariopteris?*	*Mariopteris*? sp.	
foliage	Neuropterids	*Neuropteris*	*Neuropteris (Mizoneura)* sp.	No
foliage	Neuropterids	*Neuropteris*	*Neuropteris* cf. *antecedens*	
foliage	Neuropterids	*Neuropteris*	*Neuropteris* cf. *gigantea*	
foliage	Neuropterids	*Neuropteris*	*Neuropteris* cf. *pseudogigantea*	
foliage	Neuropterids	*Neuropteris*	*Neuropteris gigantea*	
foliage	Neuropterids	*Neuropteris*	*Neuropteris jiangxiensis*	
foliage	Neuropterids	*Neuropteris*	*Neuropteris kaipingiana*	
foliage	Neuropterids	*Neuropteris*	*Neuropteris otozamioides*	
foliage	Neuropterids	*Neuropteris*	*Neuropteris pseudogigantea*	
foliage	Neuropterids	*Neuropteris*	*Neuropteris shaoguanensis?*	
foliage	Neuropterids	*Neuropteris*	*Neuropteris* sp.	
foliage	Neuropterids	*Neuropteris*	*Neuropteris* sp.	
foliage	Neuropterids	*Neuropteris*	*Neuropteris* sp.	
foliage	Neuropterids	*Neuropteris*	*Neuropteris* sp.	
foliage	Neuropterids	*Neuropteris*	*Neuropteris* sp.	
foliage	Neuropterids	*Neuropteris*	*Neuropteris* sp.	
foliage	Neuropterids	*Neuropteris*	*Neuropteris* sp.1	
foliage	Neuropterids	*Neuropteris*	*Neuropteris* sp.1	
foliage	Neuropterids	*Neuropteris*	*Neuropteris* sp.1	
foliage	Neuropterids	*Neuropteris*	*Neuropteris* sp.1	
foliage	Neuropterids	*Neuropteris*	*Neuropteris* sp.2	
foliage	Neuropterids	*Neuropteris*	*Neuropteris* sp.2	
foliage	Neuropterids	*Neuropteris*	*Neuropteris* sp.3	
foliage	Neuropterids	*Neuropteris*	*Neuropteris* sp.4	
foliage	Neuropterids	*Neuropteris*	*Neuropteris* sp.5	
foliage	Neuropterids	*Paripteris*	*Paripteris antecedens*	No
foliage	Neuropterids	*Paripteris*	*Paripteris cardiopteroides*	
foliage	Neuropterids	*Paripteris*	*Paripteris* cf. *pseudogigantea*	
foliage	Neuropterids	*Paripteris*	*Paripteris gigantea*	
foliage	Neuropterids	*Paripteris*	*Paripteris*? sp.	
foliage	Odontopterids	*Odontopteris?*	*Odontopteris*? *lopingensis*	
foliage	Pecopterids	*Pecopteris*	*Pecopteris* sp.	No
foliage	Pecopterids	*Pecopteris*	*Pecopteris* sp.1	
foliage	Pecopterids	*Pecopteris*	*Pecopteris* sp.2	
foliage	Sphenopterids	*Anisopteris*	*Anisopteris* cf. *transitionis*	No
foliage	Sphenopterids	*Archaeopteridium*	*Archaeopteridium orientale*	No

foliage	Sphenopterids	*Archaeopteridium*	*Archaeopteridium shaoguanense?*	
foliage	Sphenopterids	*Eusphenopteris*	*Eusphenopteris* cf. *scribanii*	No
foliage	Sphenopterids	*Eusphenopteris*	*Eusphenopteris* sp. cf. *foliata*	
foliage	Sphenopterids	*Rhacopteris*	*Rhacopteris angusta?*	No
foliage	Sphenopterids	*Rhacopteris*	*Rhacopteris gannanensis*	
foliage	Sphenopterids	*Rhacopteris*	*Rhacopteris kaihuaensis*	
foliage	Sphenopterids	*Rhacopteris*	*Rhacopteris ovata*	
foliage	Sphenopterids	*Rhacopteris*	*Rhacopteris plumosa*	
foliage	Sphenopterids	*Rhacopteris*	*Rhacopteris* sp.	
foliage	Sphenopterids	*Rhacopteris?*	*Rhacopteris*? *zishanensis*	
foliage	Sphenopterids	*Rhodeites?*	*Rhodeites? lanceolata*	
foliage	Sphenopterids	*Rhodeopteridium*	*Rhodeopteridium* cf. *gigantea*	No
foliage	Sphenopterids	*Rhodeopteridium*	*Rhodeopteridium* cf. *bifidum*	
foliage	Sphenopterids	*Rhodeopteridium*	*Rhodeopteridium* cf. *chunanense*	
foliage	Sphenopterids	*Rhodeopteridium*	*Rhodeopteridium* cf. *hsianghsiangensis*	
foliage	Sphenopterids	*Rhodeopteridium*	*Rhodeopteridium* cf. *nematophyllum*	
foliage	Sphenopterids	*Rhodeopteridium*	*Rhodeopteridium* cf. *parasparsum*	
foliage	Sphenopterids	*Rhodeopteridium*	*Rhodeopteridium chunanense*	
foliage	Sphenopterids	*Rhodeopteridium*	*Rhodeopteridium hsianghsiangensis*	
foliage	Sphenopterids	*Rhodeopteridium*	*Rhodeopteridium jiangningense*	
foliage	Sphenopterids	*Rhodeopteridium*	*Rhodeopteridium lianpingense*	
foliage	Sphenopterids	*Rhodeopteridium*	*Rhodeopteridium lipoldi*	
foliage	Sphenopterids	*Rhodeopteridium*	*Rhodeopteridium parasparsa*	
foliage	Sphenopterids	*Rhodeopteridium*	*Rhodeopteridium* sp.	
foliage	Sphenopterids	*Rhodeopteridium*	*Rhodeopteridium* sp.	
foliage	Sphenopterids	*Rhodeopteridium*	*Rhodeopteridium* sp.	
foliage	Sphenopterids	*Rhodeopteridium*	*Rhodeopteridium* sp.	
foliage	Sphenopterids	*Rhodeopteridium*	*Rhodeopteridium* sp.	
foliage	Sphenopterids	*Rhodeopteridium*	*Rhodeopteridium* sp. cf. *patentissimum*	
foliage	Sphenopterids	*Rhodeopteridium*	*Rhodeopteridium* sp.1	
foliage	Sphenopterids	*Rhodeopteridium*	*Rhodeopteridium* sp.2	
foliage	Sphenopterids	*Rhodeopteridium*	*Rhodeopteridium tenuis*	
foliage	Sphenopterids	*Rhodeopteridium*	*Rhodeopteridium yingdeense*	
foliage	Sphenopterids	*Rhodeopteridium?*	*Rhodeopteridium*? sp.	
foliage	Sphenopterids	*Rhodeopteridium?*	*Rhodeopteridium*? sp.	
foliage	Sphenopterids	*Rhodeopteridium?*	*Rhodeopteridium*? sp.	
foliage	Sphenopterids	*Sphenopteris Sphenopteris (?Lyginopteris)*	*Sphenopteris (?Lyginopteris) leei*	
foliage	Sphenopterids	*Sphenopteris*	*Sphenopteris* aff. *leei*	No
foliage	Sphenopterids	*Sphenopteris*	*Sphenopteris affinis*	

foliage	Sphenopterids	*Sphenopteris*	*Sphenopteris* cf. *gracilis*	
foliage	Sphenopterids	*Sphenopteris*	*Sphenopteris* cf. *obtusiloba*	
foliage	Sphenopterids	*Sphenopteris*	*Sphenopteris* cf. *scribanii*	
foliage	Sphenopterids	*Sphenopteris*	*Sphenopteris changduensis*	
foliage	Sphenopterids	*Sphenopteris*	*Sphenopteris leei*	
foliage	Sphenopterids	*Sphenopteris*	*Sphenopteris obtustloba*	
foliage	Sphenopterids	*Sphenopteris*	*Sphenopteris* sp.	
foliage	Sphenopterids	*Sphenopteris*	*Sphenopteris* sp.	
foliage	Sphenopterids	*Sphenopteris*	*Sphenopteris* sp.	
foliage	Sphenopterids	*Sphenopteris*	*Sphenopteris* sp.	
foliage	Sphenopterids	*Sphenopteris*	*Sphenopteris* sp. cf. *launoiti*	
foliage	Sphenopterids	*Sphenopteris*	*Sphenopteris* sp. cf. *praecedens*	
foliage	Sphenopterids	*Sphenopteris*	*Sphenopteris* sp.1	
foliage	Sphenopterids	*Sphenopteris*	*Sphenopteris* sp.1	
foliage	Sphenopterids	*Sphenopteris*	*Sphenopteris* sp.1	
foliage	Sphenopterids	*Sphenopteris*	*Sphenopteris* sp.2	
foliage	Sphenopterids	*Sphenopteris*	*Sphenopteris* sp.2	
foliage	Sphenopterids	*Sphenopteris*	*Sphenopteris subsulcata*	
foliage	Sphenopterids	*Sphenopteris*	*Sphenopteris tseishuiensis*	
foliage	Sphenopterids	*Triphyllopteris*	*Triphyllopteris collombiana*	No
foliage	Sphenopterids	*Triphyllopteris*	*Triphyllopteris gushiensis*	
foliage	Sphenopterids	*Triphyllopteris*	*Triphyllopteris* sp.	
foliage	Sphenopterids	*Triphyllopteris*	*Triphyllopteris* sp.	
foliage	Sphenopterids	*Triphyllopteris*	*Triphyllopteris* sp.	
foliage	Sphenopterids	*Triphyllopteris*	*Triphyllopteris* sp.	
foliage	Sphenopterids	*Triphyllopteris?*	*Triphyllopteris*? sp.2	
foliage	Incertae Sedis	*Adiantites*	*Adiantites* cf. *gothani*	No
foliage	Incertae Sedis	*Adiantites*	*Adiantites gothani*	
foliage	Incertae Sedis	*Adiantites*	*Adiantites lianpingensis?*	
foliage	Incertae Sedis	*Adiantites*	*Adiantites matouensis*	
foliage	Incertae Sedis	*Adiantites*	*Adiantites* sp.	
foliage	Incertae Sedis	*Adiantites*	*Adiantites* sp.	
foliage	Incertae Sedis	*Adiantites*	*Adiantites* sp.	
foliage	Incertae Sedis	*Adiantites*	*Adiantites* sp.	
foliage	Incertae Sedis	*Adiantites*	*Adiantites* sp.	
foliage	Incertae Sedis	*Adiantites*	*Adiantites* sp.	
foliage	Incertae Sedis	*Adiantites*	*Adiantites* sp.2	
foliage	Incertae Sedis	*Adiantites*	*Adiantites ungeri*	
foliage	Incertae Sedis	*Aneimites*	*Aneimites dichotomous*	No
foliage	Incertae Sedis	*Aneimites*	*Aneimites* sp.	
foliage	Incertae Sedis	*Aneimites*	*Aneimites szei*	
foliage	Incertae Sedis	*Aphlebia*	*Aphlebia* sp.	No
foliage	Incertae Sedis	*Cardiopteridium*	*Cardiopteridium* cf. *spetsbergerse*	No

foliage	Incertae Sedis	*Cardiopteridium*	*Cardiopteridium nanum*	
foliage	Incertae Sedis	*Cardiopteridium*	*Cardiopteridium podozamioides*	
foliage	Incertae Sedis	*Cardiopteridium*	*Cardiopteridium* sp.	
foliage	Incertae Sedis	*Cardiopteridium*	*Cardiopteridium* sp.	
foliage	Incertae Sedis	*Cardiopteridium*	*Cardiopteridium spetsbergense*	
foliage	Incertae Sedis	*Diplotmema*	*Diplotmema adiantoides*	No
foliage	Incertae Sedis	*Platyphyllum?*	*Platyphyllum*? sp.	
Progymnospermopsida	Archaeopteridales	*Archaeopteris?*	*Archaeopteris*? *gothani*	
gymnosperms	Medullosales	*Palmatopteris*	*Palmatopteris subgeniculata*	No
gymnosperms	Incertae Sedis	*Potoniea*	*Potoniea racemicarpa*	No
gymnosperms	Incertae Sedis	*Potoniea*	*Potoniea* sp.	
gymnosperms	Incertae Sedis	*Potoniea*	*Potoniea turbinata*	
gymnosperms	Incertae Sedis	*Telangium*	*Telangium* sp.	No
gymnosperms	Incertae Sedis	*Telangium*	*Telangium* sp.	
gymnosperms	Incertae Sedis	*Trigonocarpus*	*Trigonocarpus ellipticus*	No
gymnosperms	Incertae Sedis	*Trigonocarpus*	*Trigonocarpus schultzianus*	
gymnosperms	Incertae Sedis	*Trigonocarpus*	*Trigonocarpus* sp.	
gymnosperms	Incertae Sedis	*Trigonocarpus*	*Trigonocarpus* sp.	
gymnosperms	Incertae Sedis	*Carpolithus*	*Carpolithus perpusillus*	No
gymnosperms	Incertae Sedis	*Carpolithus*	*Carpolithus* sp.	
gymnosperms	Incertae Sedis	*Carpolithus*	*Carpolithus* sp.	
gymnosperms	Incertae Sedis	*Hexagonocarpus*	*Hexagonocarpus ellipticus*	No
gymnosperms	Incertae Sedis	*Hexagonocarpus*	*Hexagonocarpus* sp.	
gymnosperms	Cordaitales	*Cardiocarpus*	*Cardiocarpus changyangensis?*	No
gymnosperms	Cordaitales	*Cardiocarpus*	*Cardiocarpus cordai*	
gymnosperms	Cordaitales	*Cardiocarpus*	*Cardiocarpus* sp.	
gymnosperms	Cordaitales	*Cordaites*	*Cordaites schenkii*	No
gymnosperms	Cordaitales	*Cordaites*	*Cordaites* sp.	
gymnosperms	Cordaitales	*Cordaites*	*Cordaites* sp.	
gymnosperms	Cordaitales	*Cordaites*	*Cordaites* sp.	
gymnosperms	Cordaitales	*Samaropsis*	*Samaropsis* sp.	No
Incertae Sedis	Taeniocradaceae	*Taeniocrada?*	*Taeniocrada*? sp.	
Incertae Sedis	Incertae Sedis	*Codonospermum*	*Codonospermum* sp.	No
“Lazarus taxa”		*Aspidiaria*	*Aspidiaria* sp.	
**Serpukhovian**				
Class	Order/Family/Group	Genus	Species	Endemic genus or not
Lycopsida	Isoëtales *sensu lato*	*Bothrodendron*	*Bothrodendron circulare*	No
Lycopsida	Isoëtales *sensu lato*	*Bothrodendron*	*Bothrodendron ellipticum*	
Lycopsida	Isoëtales *sensu lato*	*Bothrodendron*	*Bothrodendron flabellatum*	
Lycopsida	Isoëtales *sensu lato*	*Bothrodendron*	*Bothrodendron* sp.a	
Lycopsida	Isoëtales *sensu lato*	*Bothrodendron*	*Bothrodendron* sp.b	
Lycopsida	Isoëtales *sensu lato*	*Bothrodendron?*	*Bothrodendron*? *yangshanense*	
Lycopsida	Isoëtales *sensu lato*	*Cathaysiodendron*	*Cathaysiodendron gushiense*	Yes

Lycopsida	Isoëtales *sensu lato*	*Cathaysiodendron*	*Cathaysiodendron yangshanense*	
Lycopsida	Isoëtales *sensu lato*	*Cathaysiodendron?*	*Cathaysiodendron*? sp.	
Lycopsida	Isoëtales *sensu lato*	*Lepidodendron*	*Lepidodendron* aff. *aolungpylukense*	No
Lycopsida	Isoëtales *sensu lato*	*Lepidodendron*	*Lepidodendron* cf. *aolungpylukense*	
Lycopsida	Isoëtales *sensu lato*	*Lepidodendron*	*Lepidodendron* cf. *shanyangense*	
Lycopsida	Isoëtales *sensu lato*	*Lepidodendron*	*Lepidodendron* cf. *subrhombicum*	
Lycopsida	Isoëtales *sensu lato*	*Lepidodendron*	*Lepidodendron* cf. *volkmannianum*	
Lycopsida	Isoëtales *sensu lato*	*Lepidodendron*	*Lepidodendron* cf. *worthenii*	
Lycopsida	Isoëtales *sensu lato*	*Lepidodendron*	*Lepidodendron dabieshanense*	
Lycopsida	Isoëtales *sensu lato*	*Lepidodendron*	*Lepidodendron huashanlingense*	
Lycopsida	Isoëtales *sensu lato*	*Lepidodendron*	*Lepidodendron jiandeense*	
Lycopsida	Isoëtales *sensu lato*	*Lepidodendron*	*Lepidodendron quadratum*	
Lycopsida	Isoëtales *sensu lato*	*Lepidodendron*	*Lepidodendron rhodeanum*	
Lycopsida	Isoëtales *sensu lato*	*Lepidodendron*	*Lepidodendron shanyangense*	
Lycopsida	Isoëtales *sensu lato*	*Lepidodendron*	*Lepidodendron* sp.	
Lycopsida	Isoëtales *sensu lato*	*Lepidodendron*	*Lepidodendron* sp.	
Lycopsida	Isoëtales *sensu lato*	*Lepidodendron*	*Lepidodendron* sp.	
Lycopsida	Isoëtales *sensu lato*	*Lepidodendron*	*Lepidodendron* sp.a	
Lycopsida	Isoëtales *sensu lato*	*Lepidodendron*	*Lepidodendron* sp.b	
Lycopsida	Isoëtales *sensu lato*	*Lepidodendron*	*Lepidodendron tangjiaense*	
Lycopsida	Isoëtales *sensu lato*	*Lepidodendron*	*Lepidodendron yuduense*	
Lycopsida	Isoëtales *sensu lato*	*Lepidostrobophyllum*	*Lepidostrobophyllum* cf. *hastatum*	No
Lycopsida	Isoëtales *sensu lato*	*Lepidostrobophyllum*	*Lepidostrobophyllum* cf. *ovatifolium*	
Lycopsida	Isoëtales *sensu lato*	*Lepidostrobophyllum*	*Lepidostrobophyllum* sp.	
Lycopsida	Isoëtales *sensu lato*	*Lepidostrobus?*	*Lepidostrobus*? sp.	
Lycopsida	Isoëtales *sensu lato*	*Sigillaria*	*Sigillaria brardii*	No
Lycopsida	Isoëtales *sensu lato*	*Sigillaria*	*Sigillaria* sp.	
Lycopsida	Isoëtales *sensu lato*	*Sigillaria?*	*Sigillaria*? sp.	
Lycopsida	Isoëtales *sensu lato*	*Stigmaria*	*Stigmaria ficoides*	No
Lycopsida	Isoëtales *sensu lato*	*Stigmaria*	*Stigmaria* sp.	
Lycopsida	Incertae Sedis	*Aspidiaria*	*Aspidiaria* sp.	No
Sphenopsida		*Archaeocalamites*	*Archaeocalamites scrobiculatus*	No
Sphenopsida		*Asterocalamites*	*Asterocalamites* cf. *scrobiculatus*	No
Sphenopsida		*Asterophyllites*	*Asterophyllites longifolius*	No
Sphenopsida		*Calamites*	*Calamites* sp.	No
Sphenopsida		*Calamostachys?*	*Calamostachys*? sp.	
Sphenopsida		*Mesocalamites*	*Mesocalamites jiangshanensis*	No
Sphenopsida		*Mesocalamites*	*Mesocalamites* sp.	
Sphenopsida		*Mesocalamites*	*Mesocalamites* sp.	
Sphenopsida		*Mesocalamites*	*Mesocalamites* sp.	
Sphenopsida		*Pothocites*	*Pothocites* sp.	No
Sphenopsida		*Sphenophyllostachys*	*Sphenophyllostachys tenerrimus*	No
Sphenopsida		*Sphenophyllum*	*Sphenophyllum pseudotenerrimum*	No

Sphenopsida		*Sphenophyllum*	*Sphenophyllum* sp.	
Sphenopsida		*Sphenophyllum*	*Sphenophyllum tenertimum*	
Sphenopsida		*Sphenophyllum*	*Sphenophyllum yuduense*	
foliage	Sphenopterids	*Eusphenopteris*	*Eusphenopteris* cf. *scribanii*	No
foliage	Sphenopterids	*Rhacopteris*	*Rhacopteris gannanensis*	No
foliage	Sphenopterids	*Rhacopteris*	*Rhacopteris plumosa*	
foliage	Sphenopterids	*Rhacopteris*	*Rhacopteris* sp.	
foliage	Sphenopterids	*Rhacopteris?*	*Rhacopteris*? *zishanensis*	
foliage	Sphenopterids	*Rhodeopteridium*	*Rhodeopteridium* sp.	No
foliage	Sphenopterids	*Rhodeopteridium*	*Rhodeopteridium* cf. *hsianghsiangense*	
foliage	Sphenopterids	*Rhodeopteridium*	*Rhodeopteridium* cf. *lipoldi*	
foliage	Sphenopterids	*Rhodeopteridium*	*Rhodeopteridium* cf. *nematophyllum*	
foliage	Sphenopterids	*Rhodeopteridium*	*Rhodeopteridium chunanense*	
foliage	Sphenopterids	*Rhodeopteridium*	*Rhodeopteridium hsianghsiangense*	
foliage	Sphenopterids	*Rhodeopteridium*	*Rhodeopteridium machanekii*	
foliage	Sphenopterids	*Rhodeopteridium*	*Rhodeopteridium multibifidum*	
foliage	Sphenopterids	*Rhodeopteridium*	*Rhodeopteridium parasparsa*	
foliage	Sphenopterids	*Rhodeopteridium*	*Rhodeopteridium quxianense*	
foliage	Sphenopterids	*Rhodeopteridium*	*Rhodeopteridium* sp.	
foliage	Sphenopterids	*Rhodeopteridium*	*Rhodeopteridium* sp.	
foliage	Sphenopterids	*Rhodeopteridium*	*Rhodeopteridium tenuis*	
foliage	Sphenopterids	*Rhodeopteridium?*	*Rhodeopteridium*? sp.	
foliage	Sphenopterids	*Rhodeopteridium?*	*Rhodeopteridium*? sp.	
foliage	Sphenopterids	*Sphenopteris*	*Sphenopteris (Palmatopteris)* sp.	No
foliage	Sphenopterids	*Sphenopteris*	*Sphenopteris* aff. *leei*	
foliage	Sphenopterids	*Sphenopteris*	*Sphenopteris* cf. *gracilis*	
foliage	Sphenopterids	*Sphenopteris*	*Sphenopteris* cf. *schatzlarensis*	
foliage	Sphenopterids	*Sphenopteris*	*Sphenopteris* cf. *scribanii*	
foliage	Sphenopterids	*Sphenopteris*	*Sphenopteris leei*	
foliage	Sphenopterids	*Sphenopteris*	*Sphenopteris obtusiloba*	
foliage	Sphenopterids	*Sphenopteris*	*Sphenopteris* sp.	
foliage	Sphenopterids	*Sphenopteris*	*Sphenopteris* sp.	
foliage	Sphenopterids	*Sphenopteris*	*Sphenopteris* sp.	
foliage	Sphenopterids	*Sphenopteris*	*Sphenopteris* sp.	
foliage	Sphenopterids	*Sphenopteris*	*Sphenopteris* sp.	
foliage	Sphenopterids	*Triphyllopteris*	*Triphyllopteris collombiana*	No
foliage	Sphenopterids	*Triphyllopteris*	*Triphyllopteris gushiensis*	
foliage	Sphenopterids	*Triphyllopteris*	*Triphyllopteris* sp.	
foliage	Sphenopterids	*Triphyllopteris*	*Triphyllopteris* sp.	
foliage	Sphenopterids	*Triphyllopteris*	*Triphyllopteris* sp.	
foliage	Alethopterids	*Lopinopteris*	*Lopinopteris intercalata*	Yes
foliage	Cardiopterids	*Cardiopteris*	*Cardiopteris frongdosa*	No

foliage	Cardiopterids	*Cardiopteris*	*Cardiopteris* sp.	
foliage	Cardiopterids	*Cardiopteris*	*Cardiopteris* sp.	
foliage	Cardiopterids	*Cardiopteris?*	*Cardiopteris*? spp.	
foliage	Mariopterids	*Mariopteris*	*Mariopteris acuta*	No
foliage	Mariopterids	*Mariopteris*	*Mariopteris acuta* f. *obtusa*	
foliage	Mariopterids	*Mariopteris*	*Mariopteris* sp.	
foliage	Neuropterids	*Linopteris*	*Linopteris* sp.	No
foliage	Neuropterids	*Linopteris*	*Linopteris* sp.	
foliage	Neuropterids	*Neuropteris*	*Neuropteris* cf. *kaipingiana*	No
foliage	Neuropterids	*Neuropteris*	*Neuropteris* cf. *pseudogigantea*	
foliage	Neuropterids	*Neuropteris*	*Neuropteris gigantea*	
foliage	Neuropterids	*Neuropteris*	*Neuropteris jiangxiensis*	
foliage	Neuropterids	*Neuropteris*	*Neuropteris kaipingiana*	
foliage	Neuropterids	*Neuropteris*	*Neuropteris otozamioides*	
foliage	Neuropterids	*Neuropteris*	*Neuropteris schlehani*	
foliage	Neuropterids	*Neuropteris*	*Neuropteris* sp.	
foliage	Neuropterids	*Neuropteris*	*Neuropteris* sp.	
foliage	Neuropterids	*Neuropteris*	*Neuropteris* sp.	
foliage	Neuropterids	*Neuropteris*	*Neuropteris* sp.	
foliage	Neuropterids	*Neuropteris*	*Neuropteris* sp.	
foliage	Neuropterids	*Neuropteris*	*Neuropteris* sp.	
foliage	Neuropterids	*Neuropteris*	*Neuropteris* sp.	
foliage	Neuropterids	*Neuropteris*	*Neuropteris zhejiangensis*	
foliage	Neuropterids	*Paripteris*	*Paripteris* cf. *pseudogigantea*	No
foliage	Neuropterids	*Paripteris*	*Paripteris gigantea*	
foliage	Neuropterids	*Paripteris*	*Paripteris*? sp.	
foliage	Odontopterids	*Odontopteris?*	*Odontopteris*? *lopingensis*	
foliage	Incertae Sedis	*Adiantites*	*Adiantites* cf. *gothani*	No
foliage	Incertae Sedis	*Adiantites*	*Adiantites gothani*	
foliage	Incertae Sedis	*Adiantites*	*Adiantites* sp.	
foliage	Incertae Sedis	*Adiantites*	*Adiantites* sp.	
foliage	Incertae Sedis	*Adiantites*	*Adiantites* sp.	
foliage	Incertae Sedis	*Adiantites*	*Adiantites* sp.	
foliage	Incertae Sedis	*Adiantites*	*Adiantites* sp.1	
foliage	Incertae Sedis	*Aneimites*	*Aneimites dichotomous*	No
foliage	Incertae Sedis	*Aneimites*	*Aneimites* sp.	
foliage	Incertae Sedis	*Aphlebia*	*Aphlebia* sp.	No
foliage	Incertae Sedis	*Cardiopteridium*	*Cardiopteridium nanum*	No
foliage	Incertae Sedis	*Cardiopteridium*	*Cardiopteridium podozamioides*	
foliage	Incertae Sedis	*Cardiopteridium*	*Cardiopteridium spetsbergense*	
foliage	Incertae Sedis	*Cyclopteris*	*Cyclopteris* sp.1	No
foliage	Incertae Sedis	*Cyclopteris?*	*Cyclopteris*? sp.2	
foliage	Incertae Sedis	*Karinopteris*	*Karinopteris acuta*	No

foliage	Incertae Sedis	*Platyphyllum?*	*Platyphyllum*? sp.	
gymnosperms	Incertae Sedis	*Telangium*	*Telangium* sp.	No
gymnosperms	Incertae Sedis	*Trigonocarpus*	*Trigonocarpus ellipticus*	No
gymnosperms	Incertae Sedis	*Trigonocarpus*	*Trigonocarpus schultzianus*	
gymnosperms	Incertae Sedis	*Trigonocarpus*	*Trigonocarpus* sp.	
gymnosperms	Incertae Sedis	*Trigonocarpus*	*Trigonocarpus* sp.	
gymnosperms	Incertae Sedis	*Trigonocarpus*	*Trigonocarpus* sp.1	
gymnosperms	Incertae Sedis	*Trigonocarpus*	*Trigonocarpus* sp.2	
gymnosperms	Incertae Sedis	*Carpolithus*	*Carpolithus* sp.	No
gymnosperms	Incertae Sedis	*Hexagonocarpus*	*Hexagonocarpus ellipticus*	No
gymnosperms	Cordaitales	*Cardiocarpus*	*Cardiocarpus cordai*	No
gymnosperms	Cordaitales	*Cordaites*	*Cordaites schenkii*	No
gymnosperms	Cordaitales	*Cordaites*	*Cordaites* sp.	
gymnosperms	Cordaitales	*Cordaites*	*Cordaites* sp.	
"Lazarus taxa"			*Rhodeopteridium lipoldi*	
"Lazarus taxa"			*Neuropteris pseudogigantea*	
**Bashkirian**				
Class	Order/Family/Group	Genus	Species	Endemic genus or not
Lycopsida	Isoëtales *sensu lato*	*Bothrodendron*	*Bothrodendron* sp.	No
Lycopsida	Isoëtales *sensu lato*	*Lepidodendron*	*Lepidodendron* sp.	No
Pteropsida	Leptosporangiopsida	*Senftenbergia*	*Senftenbergia plumosa*	No
Sphenopsida		*Calamites*	*Calamites* sp.	No
Sphenopsida		*Mesocalamites*	*Mesocalamites* sp.	No
Sphenopsida		*Sphenophyllum*	*Sphenophyllum tenerrimum*	No
foliage	Sphenopterids	*Anisopteris*	*Anisopteris petiolata*	No
foliage	Sphenopterids	*Rhodeopteridium*	*Rhodeopteridium* cf. *sublipoldi*	No
foliage	Sphenopterids	*Rhodeopteridium*	*Rhodeopteridium fengxianense*	
foliage	Sphenopterids	*Rhodeopteridium*	*Rhodeopteridium lipoldi*	
foliage	Sphenopterids	*Rhodeopteridium*	*Rhodeopteridium parasparsum*	
foliage	Sphenopterids	*Sphenopteris*	*Sphenopteris (Cyclosphenopteris) schillingsi*	No
foliage	Sphenopterids	*Sphenopteris*	*Sphenopteris* (*Lyginopteris*) *parabaeumleri*	
foliage	Sphenopterids	*Sphenopteris*	*Sphenopteris leei*	
foliage	Sphenopterids	*Sphenopteris*	*Sphenopteris obtusiloba*	
foliage	Sphenopterids	*Sphenopteris*	*Sphenopteris parabaeumleri*	
foliage	Neuropterids	*Linopteris*	*Linopteris* cf. *oblique*	No
foliage	Neuropterids	*Linopteris*	*Linopteris neuropteroides*	
foliage	Neuropterids	*Neuropteris*	*Neuropteris* cf. *scheuchzeri*	No
foliage	Neuropterids	*Neuropteris*	*Neuropteris gigantea*	
foliage	Neuropterids	*Neuropteris*	*Neuropteris longifolia*	
foliage	Neuropterids	*Neuropteris*	*Neuropteris pseudogigantea*	
foliage	Neuropterids	*Neuropteris*	*Neuropteris* sp.	

foliage	Neuropterids	*Paripteris*	*Paripteris pseudogigantea*	No
foliage	Incertae Sedis	*Cyclopteris*	*Cyclopteris* sp.	No
foliage	Incertae Sedis	*Diplotmema*?	*Diplotmema*? *subgeniculata*	
