# Supplementary material for: Diversity Dynamics of Silurian–Early Carboniferous Land Plants in South China
Source: PLoS One. 2013 Sep 20;8(9):e75706. doi: 10.1371/journal.pone.0075706 (PMC3779156; doi:10.1371/journal.pone.0075706)
Supplement: Table S2 — Silurian–Early Carboniferous geological strata in South China (main references: Regional geology of each province of South China; Cai Chongyang and Li Xingxue, 1982; Cai Chongyang, 2000; Wu Xiuyuan and Zhu Huaicheng, 2000; Wang Deming et al., 2002; Ma Xueping et al., 2009; Gonez et al., 2012). (PDF) [file pone.0075706.s002.pdf]

			Yunnan, China				Hainan, China
			Kunming-Qujing	Wenshan	Zhaotong	western Yunnan	
	Pennsylvanian	Bashkirian					
**Carboniferous**	Mississippian	Serpukhovian					
		Visean					
			Shangsi Formation				Sanlengshan Formation
			Jiusi Formation				
			Wanshoushan Formation				
		Tournaisian	Upper Tangbagou Formation				
			Lower Tangbagou Formation				
**Devonian**	Upper	late Famennian					
		Frasnian	Yidade Formation				
	Middle	Givetian		Donggangling Formation			
			Haikou Formation				
		Eifelian	Chuandong Formation				
	Lower	Emsian					
			Upper Xujiachong Formation	Pojiao Formation		Banmandaodi Formation	
		Pragian	Lower-Middle Xujiachong Formation	Posongchong Formation		Wangjiacun Formation	
			Guijiatun Formation				
		Lochkovian	Xitun Formation			Xiangyangsi Formation	
			Xiaxishan Formation				
**Silurian**	Pridoli		Yulongsi Formation				
	Ludlow						
	Wenlock						
	Llandovery						

			Guangxi, China	Guangdong, China			
				western Guangdong	northern Guangdong	central Guangdong	northeastern Guangdong
	Pennsylvanian	Bashkirian					
**Carboniferous**	Mississippian	Serpukhovian					
		Visean					
			Simen Formation	Ceshui Formation			Zhongxin/Furongshan Formation
				Shidengzi Formation			Daohu Formation
		Tournaisian		Upper Liujiatang Formation			
				Lower Liujiatang Formation		Longjiang Formation	Maozifeng Formation
**Devonian**	Upper	late Famennian		Menggong'ao Formation			
				Shaodong Formation			
				Maozifeng Formation		Shashuigang Formation	
		Frasnian					
	Middle	Givetian	Donggangling Formation		Qiziqiao Formation		
		Eifelian	Xindu Formation	Xindu Formation	Guitou Formation		
	Lower	Emsian					
			Upper Shiqiao Formation				
		Pragian	Nagaoling Formation				
			Lianhuashan Formation				
		Lochkovian					
**Silurian**	Pridoli						
	Ludlow						
	Wenlock						
	Llandovery						

			Guizhou, China				Sichuan, China
			Dushan	Kaili-Sandu	mid-southern Guizhou	Fenggang	eastern Sichuan
	Pennsylvanian	Bashkirian					
**Carboniferous**	Mississippian	Serpukhovian					
		Visean					
			Shangsi Formation				
			Jiusi Formation				
			Xiangbai Formation				
		Tournaisian	Upper Tangbagou Formation		the bottom of Dawuba Formation		
			Lower Tangbagou Formation				
**Devonian**	Upper	late Famennian	Gelaohe Formation		Wangyou Formation		
			Zhewang Formation				
		Frasnian					Shawozi Formation
	Middle	Givetian					Guanwushan Formation
							Jinbaoshi Formation
		Eifelian	Longdongshui Formation				
	Lower	Emsian	Shujiaping Formation	Lower-Middle Mangshan Group			
			Middle-Upper Danlin Formation				
							Bailiuping Formation
		Pragian	Lower Danlin Formation				Guanshanpo Formation
							Guanyinmiao Formation
							Mu'erchang Formation
		Lochkovian					Guixi Formation
**Silurian**	Pridoli						
	Ludlow						
	Wenlock		Wengxiang Group				
	Llandovery					Hanjiadian Formation	

			Hunan, China		Hubei, China		Fujian, China
			mid-southern Hunan	northwestern Hunan	Western Hubei	Eastern Hubei	northeastern Fujian
	Pennsylvanian	Bashkirian					
**Carboniferous**	Mississippian	Serpukhovian					Lindi Group
		Visean			Hezhou Formation		
			Ceshui Formation				
					Gaolishan Formation		
			Shidengzi Formation				
		Tournaisian	Upper Liujiatang Formation		Jinling Formation		
			Lower Liujiatang Formation		Changyang Formation		
**Devonian**	Upper	late Famennian	Menggong'ao Formation				
			Shaodong Formation				
			Xikuangshan Formation/Yuelushan Formation	Tizikou Formation			
		Frasnian	Yunlugong Formation	Huangjiadeng Formation		Wutung Formation	
	Middle	Givetian	Qiziqiao Formation	Yuntaiguan Formation			
			Tiaomajian Formation				
		Eifelian	Banshan Formation				
	Lower	Emsian					
			Yuankou Formation				
		Pragian					
		Lochkovian					
**Silurian**	Pridoli			Xiaoxi Formation			
	Ludlow						
	Wenlock						
	Llandovery						

			Zhejiang, China		Anhui, China	Jiangsu, China
			Jiangshan-Hangzhou	Chanahua-Changxing	Yangtse Area	Southern Jiangsu
	Pennsylvanian	Bashkirian				
Carboniferous	Mississippian	Serpukhovian	Yejiatang Group			
		Visean			Hezhou Formation	
				Gaolishan Formation		
		Tournaisian	Zhucangwu Formation			
			Xihu Formation	Upper Leigutai Member of Wutung Formation/the bottom of Jinling Formation/Chenjiabian Formation		
Devonian	Upper	late Famennian		Guanshan Member and Lower-Middle Leigutai Member of Wutung Formation		
		Frasnian				
	Middle	Givetian				
		Eifelian				
	Lower	Emsian				
		Pragian				
		Lochkovian				
Silurian	Pridoli					
	Ludlow					
	Wenlock				Maoshan Group	
	Llandovery					

			Jiangxi, China				Henan, China	Shaanxi, China	Vietnam
			Chongyi-Longnan	Lianhua-Shanggao	Yudu	Northern Jiangxi	Gushi	Shanyang and Fengxian	
	Pennsylvanian	Bashkirian						Caoliangyi Formation	
**Carboniferous**	Mississippian	Serpukhovian			Zishan Gourp	Foling Formation	Yangshan Formation	Eryuhe Formation	
		Visean				Zhanggongmiao Formation			
			Ceshui Formation						
			Shidengzi Formation						
		Tournaisian	Yanguan Formation	Upper Liujiatang Formation					
				Lower Liujiatang Formation		Wutung Formation			
**Devonian**	Upper	late Famennian							
			Xikuangshan Formation		Sanmentan Formation				
		Frasnian			Zhongpeng Formation				
	Middle	Givetian		Qiziqiao Formation	Yunshan Formation				Duong Dong Formation (age: Emsian?—Givetian?)
				Tiaomajian Formation					
		Eifelian							
	Lower	Emsian							
		Pragian							
		Lochkovian							
**Silurian**	Pridoli								Van Canh Formation
	Ludlow								
	Wenlock								
	Llandovery								
