# Supplementary material for: Diversity Dynamics of Silurian–Early Carboniferous Land Plants in South China
Source: PLoS One. 2013 Sep 20;8(9):e75706. doi: 10.1371/journal.pone.0075706 (PMC3779156; doi:10.1371/journal.pone.0075706)
Supplement: Table S3 — Silurian–Early Carboniferous diversity values and other mentioned variables (the abbreviations of geological stages are the same as in Figure 1 ). (PDF) [file pone.0075706.s003.pdf]

	Genus lowest diversity of megafossils	Genus highest diversity of megafossils	Species lowest diversity of megafossils	Species highest diversity of megafossils	Genus lowest diversity of microfossils	Genus highest diversity of microfossils	Species lowest diversity of microfossils	Species highest diversity of microfossils
**Ll–W**	0	1	0	1	6	10	13	28
**Ld–Pd**	1	2	1	3	26	27	52	63
**Lc**	9	11	16	22	22	22	40	51
**Pr**	37	44	49	69	61	67	183	234
**Em**	6	7	11	24	73	76	242	330
**Ef**	5	9	5	13	27	27	40	43
**G**	28	45	43	87	85	92	321	378
**Fr**	25	40	34	60	63	64	152	169
**Fm2**	33	51	65	158	114	130	564	726
**T1**	25	32	36	70	83	86	229	315
**T2**	6	13	7	20	40	40	70	78
**V**	50	69	126	280	81	86	225	319
**S**	38	50	83	151	0	0	0	0
**B**	13	14	20	26	0	0	0	0

	Genera confined to interval of megafossils	Species confined to interval of megafossils	Genera confined to interval of microfossils	Species confined to interval of microfossils	Only bottom boundary crossed genera of megafossils	Only bottom boundary crossed species of megafossils	Only bottom boundary crossed genera of microfossils	Only bottom boundary crossed speices of microfossils
**Ll–W**	0	0	2	10	0	0	0	0
**Ld–Pd**	0	1	15	42	0	0	0	0
**Lc**	2	4	2	23	0	0	1	1
**Pr**	27	34	12	120	5	10	2	6
**Em**	1	6	18	176	2	4	29	52
**Ef**	0	2	1	17	1	0	3	7
**G**	16	36	35	252	3	3	6	15
**Fr**	9	22	12	83	4	2	11	37
**Fm2**	11	35	40	434	2	2	14	26
**T1**	4	9	18	114	17	24	31	84
**T2**	1	4	4	33	0	1	3	18
**V**	13	64	48	206	3	2	33	19
**S**	2	22	0	0	25	57	0	0
**B**	2	16	0	0	11	4	0	0

	Only top boundary crossed genera of megafossils	Only top boundary crossed species of megafossils	Only top boundary crossed genera of microfossils	Only top boundary crossed species of microfossils	Both boundaries crossed genera of megafossils	Both boundaries crossed species of megafossils	Both boundaries crossed genera of microfossils	Both boundaries crossed species of microfossils
**Ll–W**	0	0	4	3	0	0	0	0
**Ld–Pd**	1	0	7	7	0	0	4	3
**Lc**	6	12	9	7	1	0	10	9
**Pr**	3	3	30	47	2	2	17	10
**Em**	0	0	7	8	3	1	19	6
**Ef**	2	2	0	9	2	1	23	7
**G**	8	4	27	53	1	0	17	1
**Fr**	7	8	7	16	5	2	33	16
**Fm2**	10	19	34	97	10	9	26	7
**T1**	1	0	5	12	3	3	29	19
**T2**	1	0	2	6	4	2	31	13
**V**	32	60	0	0	2	0	0	0
**S**	2	1	0	0	9	3	0	0
**B**	0	0	0	0	0	0	0	0

	Number of genus originations of megafossils	Number of species originations of megafossils	Number of genus originations of microfossils	Number of species originations of microfossils	Number of genus extinctions of megafossils	Number of species extinctions of megafossils	Number of genus extinctions of microfossils	Number of species extinctions of microfossils
**Ll–W**	0	0	6	13	0	0	2	10
**Ld–Pd**	1	1	22	49	0	1	15	42
**Lc**	8	16	11	30	2	4	3	24
**Pr**	30	37	42	167	32	44	14	126
**Em**	1	6	25	184	3	10	47	228
**Ef**	2	4	1	26	1	2	4	24
**G**	24	40	62	305	19	39	41	267
**Fr**	16	30	19	99	13	24	23	120
**Fm2**	21	54	74	531	13	37	54	460
**T1**	5	9	23	126	21	33	49	198
**T2**	2	4	6	39	1	5	7	51
**V**	45	124	48	206	16	66	81	225
**S**	4	23	0	0	27	79	0	0
**B**	2	16	0	0	13	20	0	0

	Estimated genera per-capita origination rate of megafossils	Estimated genera per-capita extinction rate of megafossils	Estimated genera per-capita origination rate of microfossils	Estimated genera per-capita extinction rate of microfossils	Estimated species per-capita origination rate of megafossils	Estimated species per-capita extinction rate of megafossils	Estimated species per-capita origination rate of microfossils	Estimated species per-capita extinction rate of microfossils
**Ll–W**	-	-	-	-	-	-	-	-
**Ld–Pd**	-	-	0.12	0	-	-	0.15	0
**Lc**	0.23	0.00	0.08	0.01	-	-	0.07	0.01
**Pr**	0.29	0.39	0.32	0.03	0.29	0.56	0.54	0.15
**Em**	0	0.04	0.02	0.06	0	0.11	0.06	0.16
**Ef**	0.12	0.07	0	0.02	0.2	0	0.15	0.12
**G**	0.44	0.28	0.19	0.06	-	-	0.8	0.55
**Fr**	0.08	0.06	0.02	0.03	0.15	0.07	0.07	0.11
**Fm2**	0.10	0.03	0.13	0.06	0.17	0.03	0.41	0.23
**T1**	0.05	0.31	0.03	0.12	0	0.36	0.08	0.28
**T2**	0.04	0.00	0.01	0.02	0	0.07	0.06	0.14
**V**	0.18	0.06	-	-	-	-	-	-
**S**	0.03	0.17	-	-	0.04	0.39	-	-
**B**	-	-	-	-	-	-	-	-

	Locality numbers of megafossils	Locality numbers of microfossils	Duration of chronological stages	Mean species diversity per Myr of megafossils	Mean species diversity per Myr of microfossils	Species occurrences of megafossils per locality	Species occurrences of micofossils per locality
**Ll–W**	1	3	16	0.03	1.28	1.00	12.67
**Ld–Pd**	8	3	8.2	0.24	7.01	0.63	24.00
**Lc**	6	4	8.4	2.26	5.42	3.83	14.50
**Pr**	17	11	3.2	18.44	65.16	5.41	23.82
**Em**	14	10	14.3	1.22	20.00	2.50	36.7
**Ef**	12	4	5.6	1.61	7.41	1.00	11.50
**G**	43	10	5	13	69.90	3.47	47.40
**Fr**	22	6	10.5	4.48	15.29	4.27	32.83
**Fm2**	81	27	6.65	16.77	96.99	4.78	40.96
**T1**	37	21	6.1	8.69	44.59	4.35	19.86
**T2**	8	6	6.1	2.21	12.13	1.75	14.50
**V**	78	17	15.8	12.85	17.22	5.96	23.06
**S**	34	0	7.7	15.2	0	6.65	0
**B**	4	0	8	2.88	0	7.25	0

	Genus origination rate of megafossils	Genus extinction rate of megafossils	Genus origination rate of microfossils	Genus extinction rate of microfossils	Speciation rate per Myr of megafossils	Species extinction rate per Myr of megafossils	Speciation rate per Myr of microfossils	Species extinction rate per Myr of microfossils
**Ll–W**	-	-	1	0.33	-	-	0.06	0.05
**Ld–Pd**	1	0	0.85	0.58	0.12	0.12	0.11	0.1
**Lc**	0.89	0.22	0.5	0.14	0.11	0.03	0.09	0.07
**Pr**	0.81	0.86	0.69	0.23	0.23	0.28	0.29	0.22
**Em**	0.17	0.5	0.34	0.64	0.04	0.06	0.05	0.07
**Ef**	0.4	0.2	0.04	0.15	0.14	0.07	0.12	0.11
**G**	0.86	0.68	0.73	0.48	0.19	0.18	0.19	0.17
**Fr**	0.64	0.52	0.3	0.37	0.08	0.07	0.06	0.08
**Fm2**	0.64	0.39	0.65	0.47	0.12	0.09	0.14	0.12
**T1**	0.2	0.84	0.28	0.59	0.04	0.15	0.09	0.14
**T2**	0.33	0.17	0.15	0.18	0.09	0.11	0.09	0.12
**V**	0.9	0.32	0.6	1	0.06	0.03	0.06	0.06
**S**	0.11	0.71	-	-	0.04	0.12	-	-
**B**	0.15	1	-	-	0.1	0.13	-	-

	Number of endemic genera	Number of cosmopolitan genera	Proportion of endemic genera in megafossils	Proportion of cosmopolitan genera in megafossils	Proportion of endemic genera in originations of megafossils	Proportion of endemic genera in extinctions of megafossils
**Ll–W**	0	0	-	-	-	-
**Ld–Pd**	0	1	0	1	0	-
**Lc**	2	7	0.22	0.78	0.25	1
**Pr**	21	16	0.57	0.43	0.7	0.66
**Em**	1	5	0.17	0.83	1	0.33
**Ef**	1	4	0.2	0.8	0.5	0
**G**	11	17	0.39	0.61	0.4	0.45
**Fr**	10	15	0.4	0.6	0.5	0.69
**Fm2**	10	23	0.3	0.7	0.43	0.46
**T1**	7	18	0.28	0.7	0.6	0.29
**T2**	1	5	0.17	0.83	0	0
**V**	4	46	0.08	0.92	0.07	0.13
**S**	2	36	0.05	0.95	0	0.07
**B**	0	13	0	1	0	0
