# Supplementary material for: Diversity Dynamics of Silurian–Early Carboniferous Land Plants in South China
Source: PLoS One. 2013 Sep 20;8(9):e75706. doi: 10.1371/journal.pone.0075706 (PMC3779156; doi:10.1371/journal.pone.0075706)
Supplement: Text S1 — The Original Silurian–Early Carboniferous paleobotanical data of South China. (PDF) [file pone.0075706.s004.pdf]

Megafossil Plants of South China (Pp. 1–85)
Llandovery (Silurian)

Guizhou

Dongkala Section, in Fenggang County: Hanjiadian or Liangshan Formation

Pinnatiramosus Geng, 1986

Pinnatiramosus qianensis Geng, 1986 1986 Geng Baoyin, pl. I-VI; 1995 Cai Chongyang et Li Xingxue, pl. I, 1-5; 1996 Cai Chongyang et al., fig. 2; 2007 Wang Yi et Cai Chongyang, pl.1-9; 2013 Wang Yi et al., fig.2A, B [Note: fig.2C indicating that this megafossil plant was also collected from the base of Liangshan Formation (Lower Permian), Shiqian, Guizhou]

Ludlow–Pridoli (Silurian)

Sichuan

Xuanhe, Guangyuan City: upper part of the Jintaiguan Formation to the lower part of the Chejiaba Formation

The rock samples were collected from Xuanhe, about 10 km north of Chaotianyi, Guangyuan County , Sichuan Province

Type I, II, III 2004 Wang Y et al., pl. I, 1-10

Hunan

Yutan Village Section, Jiashihe Section and an unnamed section near Geopark of Zhangjiajie, Zhangjiajie City: Xiaoxi Formation

Type I, II, III 2010 Wang Y et al., pl. IV, 4, 5; IV, 1-3; IV, 6

Yunnan

Xiaxishan Reservior, Qujing City: Upper Yulongsi Formation

Zosterophyllum Penhallow, 1892

Zosterophyllum qujingense Hao et al., 2007 2007a Hao et al., figs.3, 5a-d

Vietnam

Locality 1, South China Plate: Van Canh Formation

Plant fragments 2012 Gonez et al., tab. 1.

Locality 2 and 3, South China Plate: Van Canh Formation

Aberlemnia-type 2012 Gonez et al., tab. 1.

Zosterophylls indet. 2012 Gonez et al., tab. 1.

Lochkovian (Early Devonian)

Yunnan

1. Xiaxishan Reservior, Qujing City: Xiaxishancun Formation

Zosterophyllum Penhallow, 1892
Zosterophyllum xishanense Hao et al., 2007 2007a Hao et al., figs.4, 5e-g, 6-8
Zosterophyllum sp. 2 1977 Li Xingxue et Cai Chongyang, pl.I, 9, 18, 18a

2. Cuifeng Mountain Section, Qujing City: Xiaxishancun Formation
Drepanophycus Göppert, 1852
?*Drepanophycus ramificanalis* Li, 1985 2001 Li Daiyun et Ge Hongru, pl. 29, 1-2; 30, 1-9
Zosterophyllum Penhallow, 1892
Zosterophyllum sp. 1978 Li Xingxue et Cai Chongyang, p. 3

3. Huaguoshan Section, Shengfeng District, Qujing City: Xitun Formation
Xitunia Xue, 2009
Xitunia spinitheca Xue, 2009 2009 Xue Jinzhuang, pl.I, 1-5; fig.1
Zosterophyllum Penhallow, 1892
Zosterophyllum minorstachyum Xue, 2009 2009 Xue Jinzhuang, pl.I, 6-12; fig.2
Zosterophyllum shengfengense Hao, Xue, Guo et Wang DM 2010 Hao et al., figs. 2-5

4. Wenquan Commune, Changning County: The fifth layer of Lower Devonian
Zosterophyllum Penhallow, 1892
Zosterophyllum sp. 1983 Li Daiyun et Yang Jiawen, p. 37.

Sichuan

1. Yanmenba Section, Jiangyou City: Pingyipu Formation (Lochkovian–Pragian)
Amplectosporangium Geng, 1992
Amplectosporangium jiangyouense Geng 1992 1992b Geng Baoyin, pl.I, 1
Drepanophycus Göppert, 1852
Drepanophycus spinaeformis Göppert, 1852 1992a Geng Baoyin, pl.6, 45-47
Drepanophycus spinosus Kräusel et Weyland 1933 1992a Geng Baoyin, pl.6, 48, 49
Drepanophycus? sp. 1992a Geng Baoyin, pl.6, 50-52
Eogaspsiea Daber, 1960
Eogaspsiea gracilis Daber, 1960 1992a Geng Baoyin, pl.5, 36-40; pl.7, 61
Hicklingia Kidston et Lang, 1923
Hicklingia cf. *edwardii* Kidston et Lang, 1923 1992a Geng Baoyin, pl.3, 17-21; pl.7, 59-60
Oricilla Gensel, 1982
Oricilla unilateralis Geng, 1992 1992a Geng Baoyin, pl.4, 28-35; pl.7, 58
Psilophyton (Dawson) Hueber et Banks, 1967
Psilophyton? sp. 1992a Geng Baoyin, pl.5, 43-44
Sporogonites Halle
Sporogonites xichuanensis Li et Cai, 1978 1978 Li Xingxue et Cai Chongyang, pl. I, 21, 21a.
Uskiella Shute et Edwards, 1989
Uskiella sp. 1992a Geng Baoyin, pl.5, 41-42
Zosterophyllum Penhallow, 1892
Zosterophyllum sp. 1977 Li Xingxue et Cai Chongyang, pl. V, 14

Zosterophyllum myretonianum Penhallow, 1892 1992a Geng Baoyin, pl.1, 1-9

Zosterophyllum sichuanense Geng, 1992 1992a Geng Baoyin, pl.2, 10-14

Zosterophyllum yunnanicum Hsü, 1966 1992a Geng Baoyin, pl.2, 15-16

Zosterophyllum longa Wang DM, 2007 2007 Wang Deming, pl. I, 1-11

An unnamed protolepidodendrid lycopsids (Emendation to *Leclercqia complexa* Banks, Bonamo et Geierson 1972) 1992a Geng Baoyin, pl.7, 62-64; pl.8, 65-71; 2009 Xu et Wang, pls. I-II.

2. Ganxi Section, Beichuan County, Mianyang City: Upper Pingyipu Formation

Zosterophyllum Penhallow, 1892

Zosterophyllum sp. 2 1977 Li Xingxue et Cai Chongyang, pl.V, 15

Pragian (Early Devonian)

Yunnan

1. Guijiatun Section, Qujing City: Guijiatun Formation

Zosterophyllum Penhallow, 1892

?*Zosterophyllum myretonianum* Penhallow 2001 Li Daiyun et Ge Hongru, pl. 10, 1-5, 7, 8, 10

2. Xujiachong Section, Qujing City: Guijiatun Formation

Taeniocrada White, 1902

?*Taeniocrada langii* Stockmans 1985 Li Daiyun, pl.36, 4, 5; 2001 Li Daiyun et Ge Hongru, pl. 5, 4, 8

Xujiachong Section, Qujing City: Xujiachong Formation (1-6 layer)

Bracteophyton Wang et Hao, 2004

Bracteophyton variatum Wang et Hao, 2004 2004 Wang et Hao, figs.1-5

Drepanophycus Göppert, 1852

Drepanophycus qujingensis Li et Edwards, 1995 1966 Hsü, p. 51; 1995 Li Chengsen et Edwards, figs.2-74 ; 2002 Wang DM et al., fig. 1

Guangnania Wang et Hao, 2002

Guangnania cuneata Wang et Hao, 2002 2002 Wang DM et al., fig. 1

Hedeia Cookson, 1935

Hedeia sinica Hao et Gensel, 1998 2002 Wang DM et al., fig. 1

Huia Geng, 1985

Huia gracilis Wang et Hao, 2001 2001 Wang et Hao, pls.1-5; figs.1-4; tab.1; 2002 Wang DM et al., fig. 1

Zosterophyllum Penhallow, 1892

Zosterophyllum australianum Lang et Cookson, 1930 emend. Hao and Gensel, 1998 1992 Hao Shougang, figs. 3-33; 2002 Wang DM et al., fig. 1

Zosterophyllum yunnanicum Hsü, 1966 1966 Hsü, pl. I, 3-8; II, 1-6; fig. 3; 1974 Gu et Zhi, pl. I, 6-12; 1977 Li Xingxue et Cai Chongyang, pl.II, 1-7, 10; 1995 Cai Chongyang et Li Xingxue, pl. III, 1-8; 2002 Wang DM et al., fig. 1

Zosterophyllum sp.4 1977 Li Xingxue et Cai Chongyang, pl.II, 13, 15

Zosterophyllum sp.6 1977 Li Xingxue et Cai Chongyang, pl.II, 18, 19

Zosterophyllum sp.7 1977 Li Xingxue et Cai Chongyang, pl.II, 20, 20a

3. Cuifeng Mountain Section, Qujing City: Guijiatun Formation

Zosterophyllum Penhallow, 1892

Zosterophyllum myretonianum Penhallow 1977 Li Xingxue et Cai Chongyang, pl.I, 1-7, 8, 8a; fig.41; 1985 Li Daiyun, pl.33, 5, 7, 11; pl.34, 6

4. Zhichang Section, Wenshan City: Posongchong Formation

Adoketophyton Li et Edwards, 1992

Adoketophyton parvulum Zhu et al., 2011 2011 Zhu et al., pls. I-III

Adoketophyton subverticillatum (Li et Cai 1977) Li et Edwards, 1992 1992 Li et Edwards, pl.1-4; figs.2; 2001 Li Daiyun et Ge Hongru, pls. 34, 1-6; 35, 1-6; 36, 1-4; 2003 Hao Shougang et al., pls. I-III; figs. 1-2

Adoketophyton sp. 2013 Hao et Xue, p. 184

Catenalis Hao et Beck, 1991

Catenalis digitata Hao et Beck, 1991 2001 Li Daiyun et Ge Hongru, pl. 3, 1-4

Celatheca Hao et Gensel, 1995

Celatheca beckii Hao et Gensel, 1995 2013 Hao et Xue, p. 184

Cervicornus Li et Hueber, 2000

Cervicornus wenshanensis Li et Hueber, 2000 2000 Li et Hueber, pl.I; fig.1; 2001 Li Daiyun et Ge Hongru, pl. 31, 6, 7; 2013 Hao et Xue, p. 184

Demersatheca Li et Edwards, 1996

Demersatheca contigua (Li and Cai, 1977) Li et Edwards, 1996 1977 Li et Cai, pl.III, 4, 5, 7, 9; fig. 7; 1996 Li et Edwards, pl.I-IV, figs.1, 2; 2001 Li Daiyun et Ge Hongru, pls. 14, 1-7; 15, 1-7; 2013 Hao et Xue, p. 184

Discalis Hao, 1989

Discalis longistipa Hao, 1989 1989a Hao, pls. I-IV; 2001 Li Daiyun et Ge Hongru, pl. 16, 3

Dibracophyton Hao, Xue, Zhu et Wang, 2012

Dibracophyton acrovatum Hao, Xue, Zhu et Wang, 2012 2012 Hao Shougang et al., pls. I-III, figs. 1-3

Distichophytum Mägdefrau, 1938

Distichophytum sp. 2013 Hao et Xue, p. 184

Eophyllophyton Hao, 1988

Eophyllophyton bellum Hao, 1988 1988 Hao Shougang, pls. I-III; 1993 Hao Shougang et Beck, pls. I-VI; 1995 Cai Chongyang et Li Xingxue, pl. II, 1, 2; 2001 Li Daiyun et Ge Hongru, pls. 23, 1; 24, 1-5

Estinnophyton Hao, Wang DM et Wang Q, 2004

Estinnophyton yunnanense Hao, Wang DM et Wang Q, 2004 2004 Hao, Wang DM et Wang Q, figs.1-34

Guangnania Wang et Hao, 2002

Guangnania cuneata Wang et Hao, 2002 2013 Hao et Xue, p. 184

Gumuia Hao, 1989

Gumuia zyzzata Hao, 1989 1989b Hao Shougang, pl.I, 1-3; pl.II, 1-11; 2001 Li Daiyun et Ge Hongru, pls. 16, 1, 2, 4-6; 17, 1-4, 6

Halleophyton Li et Edwards, 1997

Halleophyton zhichangense Li et Edwards, 1997 1997 Li Chengsen et Edwards D, pl.1-29; 2001 Li Daiyun et Ge Hongru, pls. 32, 1-6; 33, 1-4; 2013 Hao et Xue, p. 184

Hicklingia Kidston et Lang, 1923

cf. *Hicklingia* sp. 2013 Hao et Xue, p. 184

Huia Geng, 1985

Huia recurvata Geng, 1985 1985 Geng Baoyin, pl.I; pl.II; 2001 Li Daiyun et Ge Hongru, pl. 6, 1-9; 2013 Hao et Xue, p. 184

Hueberia Yang et Li, 2009

Hueberia zhichangensis Yang et Li, 2009 2009 Yang Nan et Li Chengsen, pls. I, 1-5; II, 1-4; 2013 Hao et Xue, p. 184

Oricilla Gensel, 1982

Oricilla sp. 2013 Hao et Xue, p. 184

Polythecophyton Hao, Gensel et Wang, 2001

Polythecophyton demissum Hao, Gensel et Wang, 2001 2001 Hao, Gensel et Wang, pls.1-3; figs.1-3

Psilophyton (Dawson) Hueber et Banks, 1967

Psilophyton primitivum Hao, 1998 2013 Hao et Xue, p. 184

Ramoferis Hao et Xue, 2011

Ramoferis amalia Hao et Xue, 2011 2013 Hao et Xue, p. 184

Stachyophyton Geng, 1983

Stachyophyton yunnanense Geng, 1983 1983 Geng, fig.1; 1996 Wang et Cai, pl.I, 1-5; pl.II, 1-9; pl.III, 1, 2; pl.IV, 1-4; figs.1-3; 2001 Li Daiyun et Ge Hongru, pl. 25, 1-9; 2013 Hao et Xue, p. 184

Taeniocrada White, 1902

?*Taeniocrada* cf. *dubia* Kräusel et Weyland 2001 Li Daiyun et Ge Hongru, pl. 26, 5

Pauthecophyton Xue et al., 2012

Pauthecophyton gracile Xue et al., 2012 2012 Xue et al., pls. I-IV; figs. 1, 2; 2013 Hao et Xue, p. 184

Wenshania Zhu et Kenrick, 1999

Wenshania zhichangensis Zhu et Kenrick, 1999 1999 Zhu et Kenrick, pl.I, 1-5; figs.1-3; 2001 Li Daiyun et Ge Hongru, pl. 18, 1-6; 2013 Hao et Xue, p. 184

Yunia Hao et Beck, 1991

Yunia dichotoma Hao et Beck, 1991 1991b Hao et Beck, pls.I-IV; figs. 1-3; 2001 Li Daiyun et Ge Hongru, pl. 22, 1-6

Zhenglia Hao et al., 2006

Zhenglia radiata Hao et al. 2006 2006 Hao et al., pl.1, 1-12; fig.2

Zosterophyllum Penhallow, 1892

Zosterophyllum australianum Lang et Cookson, 1930 emend. Hao and Gensel, 1998 1977 Li et Cai, pl.III, 6, 8, 10, 11, 13, 13a; 1998 Hao et Gensel, fig. 2, f, g; fig.6; 2001 Li Daiyun et Ge Hongru, pls. 9, 1-8; 21, 1

Zosterophyllum minifertillum 2013 Hao et Xue, p. 184

Zosterophyllum ramosum Hao et Wang, 2000 2000 Hao et Wang, pl.3, 1-7; pl.4, 1-10; fig.2, A-F

Zosterophyllum tenerum Hao et Xue, 2013 2013 Hao et Xue, p. 184

Zosterophyllum yunnanicum? Hsü 2001 Li Daiyun et Ge Hongru, pls. 11, 4, 6, 11; 12, 1-8; 13, 8, 11, 12, 15, 16

5. Changputang Section, Wenshan City: Posongchong Formation

Catenalis Hao et Beck, 1991

Catenalis digitata Hao et Beck, 1991 1991a Hao et Beck, figs.1-31; 1998 Hao Shougang et Gensel, p.6; 2001 Li Daiyun et Ge Hongru, pl. 3, 1-4; 2001 Li Daiyun et Ge Hongru, pls. 19, 1-4; 20, 1-5

Celatheca Hao et Gensel, 1995

Celatheca beckii Hao et Gensel, 1995 1995 Hao et Gensel, figs. 1-35; 1998 Hao Shougang et Gensel, p.6

Eophyllophyton Hao, 1988

Eophyllophyton bellum Hao, 1988 1998 Hao Shougang et Gensel, p.6

Gumuia Hao, 1989

Gumuia zyzzata Hao, 1989 1998 Hao Shougang et Gensel, p.6

Hedeia Cookson, 1935

Hedeia sinica Hao et Gensel, 1998 1998 Hao Shougang et Gensel, fig.2, d; fig.3; 2001 Li Daiyun et Ge Hongru, pl. 5, 3

Huia Geng, 1985

Huia recurvata Geng, 1985 1998 Hao Shougang et Gensel, p.6

Psilophyton Dawson, 1859 emend. Hueber et Banks, 1967

Psilophyton primitivum Hao et Gensel 1998 1998 Hao Shougang et Gensel, fig.2, f, j; fig.4; 2001 Li Daiyun et Ge Hongru, pl. 21, 2

Ramoferis Hao et Xue, 2011

Ramoferis amalia Hao et Xue, 20112011 Hao et Xue, pls. I-III; fig. 2

Zhenglia Hao et al., 2006

Zhenglia radiata Hao et al., 2006 2006 Hao et al., pl.1, 1-12; fig.2

Zosterophyllum Penhallow, 1892

Zosterophyllum australianum Lang et Cookson, 1930 emend. Hao and Gensel, 1998 2000 Hao et Wang, pl.1, 1-5; pl.2, 1-6; fig.1, A-H

Zosterophyllum ramosum Hao et Wang, 2000 1998 Hao Shougang et Gensel, fig.2, h, i

6. Gegu train station Section, Mengzi District: Posongchong Formation

Adoketophyton Li et Edwards, 1992

Adoketophyton subverticillatum (Li et Cai, 1977) Li et Edwards, 1992 1998 Hao Shougang et Gensel, p.6

Baragwanathia Lang and Cookson, 1935

Baragwanathia sp. 1998 Hao Shougang et Gensel, fig.2, a-c

Eophyllophyton Hao, 1988

Eophyllophyton bellum Hao, 1988 1998 Hao Shougang et Gensel, p.6

Huia Geng, 1985

Huia recurvata Geng, 1985 1998 Hao Shougang et Gensel, p.7

Zosterophyllum Penhallow, 1892

Zosterophyllum australianum Lang et Cookson, 1930 1998 Hao Shougang et Gensel, p.7

7. Datianjiao Section, Mengzi District: Posongchong Formation

Baragwanathia Lang et Cookson, 1935

?*Baragwanathia yunnanensis* Li 2001 Li Daiyun et Ge Hongru, pl. 26, 2-3

?*Baragwanathia* sp. 2001 Li Daiyun et Ge Hongru, pl. 26, 1, 4, 6, 7

8. Daliantang Section, Guangnan County: Posongchong Formation

Adoketophyton Li et Edwards, 1992

Adoketophyton subverticillatum (Li et Cai, 1977) Li et Edwards, 1992 1977 Li Xingxue et Cai Chongyang, pl.III, 1-3, 3a; fig.8; 2003 Hao Shougang et al., pls. I-III; figs. 1-2

Guangnania Wang et Hao, 2002

Guangnania cuneata Wang et Hao, 2002 2002 Wang et Hao, pls.I-III; figs.1-3

Zosterophyllum Penhallow, 1892

Zosterophyllum australianum Lang et Cookson, 1930 emend. Hao and Gensel, 1998 1977 Li Xingxue et Cai Chongyang, pl.III, 6, 8, 10, 11, 13, 13a; fig.6; 2001 Li Daiyun et Ge Hongru, pls. 9, 1-8; 21, 1

Zosterophyllum? sp. 1977 Li Xingxue et Cai Chongyang, pl.III, 12

9. Zhaotong: Xujiachong Formation

Psilophytites Høeg, 1952

?*Psilophytites* sp. 2001 Li Daiyun et Ge Hongru, pl. 5, 2

10. Wenquan Commune, Changning County: The tenth layer of Lower Devonian

Psilophytites Høeg, 1952

Psilophytites sp. 1983 Li Daiyun et Yang Jiawen, p. 37.

Zosterophyllum Penhallow, 1892

Zosterophyllum sp. 1983 Li Daiyun et Yang Jiawen, p. 37.

11. Mojiang and Baoshan: Longhuashan Formation?

Only plant fragments 1982 Cai Chongyang et Li Xingxue

Guizhou

Lishan Section, Dushan County: Lower Bangzhai Formation

Zosterophyllum Penhallow, 1892

Zosterophyllum sp. 1982 Cai Chongyang et Li Xingxue, p. 110

Guangxi

1. Shiqiao Section, Cangwu County: Lower–Middle Cangwu Group

Changwuia Hilton et Li, 2000

Changwuia schweitzeri Hilton et Li, 2000 2000 Hilton et Li, pl. I, 1-6; fig. 1.

Taeniocrada White, 1902

Taeniocrada decheniana (Göppert) Kräusel and Weyland 1982 Cai et Li, p. 111

Zosterophyllum Penhallow, 1892

Zosterophyllum sinense Li and Cai, 1977 1977 Li Xingxue et Cai Chongyang, pl.IV, 1, 3-8; pl.V, 1-6; fig.9; 1995 Cai Chongyang et Li Xingxue, pl. II, 3

2. Longshanwei, Guizhou: Lianhuashan Formation

No plant record

Sichuan

1. Yanmenba Section, Jiangyou City: Pingyipu Formation (Lochkovian–Pragian)

Amplectosprangium Geng, 1992

Amplectosprangium jiangyouense Geng, 1992 1992b Geng Baoyin, pl.I, 1

Drepanophycus Göppert, 1852

Drepanophycus spinaeformis Göppert, 1852 1992a Geng Baoyin, pl.6, 45-47

Drepanophycus spinosus Kräusel et Weyland, 1933 1992a Geng Baoyin, pl.6, 48, 49

Drepanophycus? sp. 1992a Geng Baoyin, pl.6, 50-52

Eogaspsiea Daber, 1960

Eogaspsiea gracilis Daber, 1960 1992a Geng Baoyin, pl.5, 36-40; pl.7, 61

Hicklingia Kidston et Lang, 1923

Hicklingia cf. *edwardii* Kidston et Lang, 1923 1992a Geng Baoyin, pl.3, 17-21; pl.7, 59-60

Oricilla Gensel, 1982

Oricilla unilateralis Geng, 1992 1992a Geng Baoyin, pl.4, 28-35; pl.7, 58

<u>*Psilophyton* Dawson, 1859 emend. Hueber et Banks, 1967</u>

Psilophyton? sp. 1992a Geng Baoyin, pl.5, 43-44

<u>*Sporogonites* Halle</u>

Sporogonites xichuanensis Li et Cai, 1978 1978 Li Xingxue et Cai Chongyang, pl. I, 21, 21a.

<u>*Uskiella* Shute et Edwards, 1989</u>

Uskiella sp. 1992a Geng Baoyin, pl.5, 41-42

<u>*Zosterophyllum* Penhallow, 1892</u>

Zosterophyllum sp. 1977 Li Xingxue et Cai Chongyang, pl.V, 14

Zosterophyllum myretonianum Penhallow, 1892 1992a Geng Baoyin, pl.1, 1-9

Zosterophyllum sichuanense Geng, 1992 1992a Geng Baoyin, pl.2, 10-14

Zosterophyllum yunnanicum Hsü, 1966 1992a Geng Baoyin, pl.2, 15-16

Zosterophyllum longa Wang DM, 2007 2007 Wang Deming, pl. I, 1-11

An unnamed protolepidodendrid lycopsids (Emendation to *Leclercqia complexa* Banks, Bonamo et Geierson 1972) 1992a Geng Baoyin, pl.7, 62-64; pl.8, 65-71; 2009 Xu et Wang, pls. I-II.

2. Ganxi Section, Beichuan County, Mianyang City: Upper Pingyipu Formation

<u>*Zosterophyllum* Penhallow, 1892</u>

Zosterophyllum sp. 2 1977 Li Xingxue et Cai Chongyang, pl.V, 15

Emsian (Middle Devonian)

Yunnan

1. Xujiachong Section, Qujing City: Xujiachong Formation (7–8th layer)

<u>*Drepanophycus* Göppert, 1852</u>

Drepanophycus qujingensis Li et Edwards, 1995 1966 Hsü, p. 51; 1995 Li Chengsen et Edwards, figs.2-74 ; 2002 Wang DM et al., fig. 1

<u>*Hsüa* Li, 1982</u>

Hsüa deflexa Wang DM, Hao et Wang Q, 2003 2003a Wang DM, Hao et Wang Q, figs.1-52; 2003b Wang DM, Hao et Wang Q, figs.1-6

Hsüa robusta (Li et Cai) Li, 1982 1978 Li Xingxue et Cai Chongyang, pl.II, 7-14

<u>*Zosterophyllum* Penhallow, 1892</u>

Zosterophyllum spathulatum Li et Cai, 1977 1977 Li Xingxue et Cai Chongyang, pl.II, 16, 16a; fig.2

Zosterophyllum yunnanicum Hsü, 1966 1978 Li Xingxue et Cai Chongyang, p. 2

2. Longhuashan Section, Qujing City: Longhuashan Formation

<u>*Drepanophycus* Göppert, 1852</u>

Drepanophycus qujingensis Li et Edwards 1995 Cai Chongyang et Li Xingxue, pl. II, 4

<u>*Hsüa* Li, 1982</u>

Hsüa robusta (Li et Cai) Li, 1982 1978 Li Xingxue et Cai Chongyang, pl.II, 6-14; 1992 Li Chengsen, pls.I-V; figs.1-4

<u>*Zosterophyllum* Penhallow, 1892</u>

Zosterophyllum bifurcatum Li et Cai, 19771977 Li Xingxue et Cai Chongyang, pl.I, 16, 17; pl. II, 17; fig.3

Zosterophyllum yunnanicum Hsü, 1977 Li Xingxue et Cai Chongyang i, pl.II, 1-7, 10; 1992 Hao Shougang, pls. I-II; fig. 1; 2007 Wang DM, pls. II-III

Zosterophyllum cf. *yunnanicum* Hsü 1977 Li Xingxue et Cai Chongyang, pl.II, 8, 8a, 9
Zosterophyllum sp. 1 1977 Li Xingxue et Cai Chongyang, pl.I, 10-15; pl.V, 9, 9a
Zosterophyllum sp. 3 1977 Li Xingxue et Cai Chongyang, pl.II, 12; fig.5
Zosterophyllum sp. 5 1977 Li Xingxue et Cai Chongyang, pl.II, 11, 14

3. Dacaozi Section, Ninglang County: Longhuashan Formation
Drepanophycus Göppert, 1852
Drepanophycus spinaeformis Göppert, 1852 1982 Cai et Li, p. 110
Taeniocrada White, 1902
Taeniocrada sp. 1982 Cai et Li, p. 110

4. Mingyin, Lijiang County: Banmandaodi Formation
Zosterophyllum Penhallow, 1892
Zosterophyllum? sp. 1977 Li Xingxue et Cai Chongyang, pl.IV, 17, 18
Taeniocrada White, 1902
Taeniocrada sp. 1982 Cai et Li, p. 111
Psilophytites Høeg, 1952
Psilophytites sp. 1982 Cai et Li, p. 112

5. Mojiang and Baoshan: Longhuashan Formation?
Only plant fragments 1982 Cai Chongyang et Li Xingxue

Guangxi

1. Shiqiao Section, Cangwu County: Upper Cangwu Group
Zosterophyllum Penhallow, 1892
Zosterophyllum cf. *yunnanicum* Hsü, 1966 1982 Cai et Li, p. 111
Zosterophyllum sp. a 1977 Li Xingxue et Cai Chongyang, pl.IV, 2, 2a

2. Huangguan Section, Guanyang County: Middle–Upper Shiqiao Group
Zosterophyllum Penhallow, 1892
Zosterophyllum cf. *yunnanicum* Hsü 1966 1982 Cai et Li, p. 111
Zosterophyllum sp. b 1977 Li Xingxue et Cai Chongyang, pl.V, 7, 7a
Zosterophyllum? sp. c 1977 Li Xingxue et Cai Chongyang, pl.V, 8

Guizhou

1. Lishan Section, Dushan County: Upper Bangzhai Formation
Drepanophycus Göppert, 1852
Drepanophycus spinaeformis Göppert, 1852 1982 Cai et Li, p. 110
Psilophyton Dawson, 1859 emend. Hueber et Banks, 1967
Psilophyton cf. *goldschmidtii* Halle, 1916 1982 Cai et Li, p. 110
Taeniocrada White, 1902
Taeniocrada decheniana (Göppert) Kräusel and Weyland 1982 Cai et Li, p. 110

Zosterophyllum Penhallow, 1892
Zosterophyllum dushanense Li et Cai, 1977 1977 Li Xingxue et Cai Chongyang, pl.V, 10, 10a; fig.10
Zosterophyllum yunnanicum Hsü 1977 Li Xingxue et Cai Chongyang, pl.V, 11, 11a
Zosterophyllum sp. A 1977 Li Xingxue et Cai Chongyang, pl.V, 13, 13a; fig.11
Zosterophyllum sp. B 1977 Li Xingxue et Cai Chongyang, pl.V, 12, 12a

2. Banzhuang Section, Duyun County: Lower–Middle Mangshan Group
Drepanophycus Göppert, 1852
Drepanophycus spinaeformis Göppert, 1852 1982 Cai et Li, p. 111
Taeniocrada White, 1902
Taeniocrada sp. 1982 Cai et Li, p. 111
Zosterophyllum Penhallow, 1892
Zosterophyllum sp. C 1977 Li Xingxue et Cai Chongyang, pl.V, 16

3. Pingzhai Section, Sandu County: Middle Danlin Formation
Drepanophycus Göppert, 1852
Drepanophycus spinaeformis Göppert, 1852 1994 Geng Baoyin et Zhu Weiqing, pl.1-2

Hunan

Yuankou Section, Jiangyong County: Yuankou Formation
Zosterophyllum Penhallow, 1892
Zosterophyllum cf. *yunnanicum* Hsü 1982 Cheng, pl. 323, 3, 3a, 7; 1982 Cai et Li, p. 111

Vietnam

2 localities, South China Plate: Duong Dong Formation (Emsian?)
Some basal euphyllophytes 2012 Gonez et al., tab. 1.

Eifelian (Middle Devonian)

Guizhou

Huaxi Section, Guiyang City: Lower Hou'ershan Formation
Aspidiaria Presl, 1838
Aspidiaria? sp. 1982 Cai et Li, p. 112
Dictyoxylon Brongniart ex Renault, 1872
Dictyoxylon? sp. 1982 Cai et Li, p. 112
Lepidodendropsis Lutz, 1933
Lepidodendropsis? sp. 1982 Cai et Li, p. 112

Hunan

1. Youtingwei Section, Lingling County: Lower Middle Devonian

Dawsonites Halle, 1916

Dawsonites(*Psilophyton*) sp. 1982 Cheng, pl. 323, 1-2, 5

2. An uncertain Section: Xindu Formation

No plant record 1982 Cai Chongyang et Li Xingxue

3. Ningyuan: Banshan Formation

Only plant fragments 1982 Cai Chongyang et Li Xingxue

Yunnan

1. Xichong Section, Zhanyi County: Chuandong Formation

Taeniocrada White, 1902

Taeniocrada sp. 1982 Cai et Li, p. 122

2. Yuanjiang: Liji Formation

Taeniocrada White, 1902

Taeniocrada sp. 1982 Cai et Li, p. 122

3. Kunming

Thursophyton Nathorst, 1914-1915

Thursophyton sp. 1974 Gu et Zhi, pl. I, 13-15.

Guangxi

1. Xindu Section, He County: Xindu Formation

Dawsonites Halle, 1916

Dawsonites(*Psilophyton*) sp.A 1987 Schweitzer et Cai, pl. XX, 1

Minarodendron Li, 1990

Minarodendron cathaysiense (Schweitzer et Cai) Li 1990 1982 Cai et Li, p. 112

Psilophytites Høeg, 1952

Psilophytites sp. 1982 Cai et Li, p. 112

2. Beiliu County: Eifelian?

Psilophytites Høeg, 1952

Psilophytites sp. 1977 Feng et al., pl. 230, 1

3. Xiuren County: Eifelian?

Minarodendron Li, 1990

Minarodendron cathaysiense (Schweitzer et Cai) Li, 1990 1977 Feng Shaonan et al., pl.230, 7, 8

4. Lipu County: Eifelian?

Minarodendron Li, 1990

Minarodendron cathaysiense (Schweitzer et Cai) Li, 1990 1977 Feng Shaonan et al., pl.230, 7, 8

Vietnam

2 localities, South China Plate: Duong Dong Formation (Eifelian?)
Some basal euphyllophytes 2012 Gonez et al., tab. 1.

Givetian (Middle Devonian)

Guizhou

Gaopochang, Huaxi District, Guiyang City: Upper Dushan Formation
Lepidodendropsis Lutz, 1933
Lepidodendropsis arborescens 1982 Cai et Li, p. 113
"Protopteridium" minutum [possibly *Eocladoxylon minutum* (Halle) Koidzumi emend. Berry et Wang, 2006] 1982 Cai et Li, p. 113

Hubei

Huaibaoshi Section, Zigui County: Upper Yuntaiguan Fomation
Amplectosporangium Geng, 1992
Amplectosporangium? *jiangyouense* Geng 1999 Feng Shaonan et Zhang Renjie, pl.IV-1; pl.V-4; pl.III-1-6
Archaeosperma Pettitt et Beck, 1968
Archaeosperma sp. 1999 Feng Shaonan et Zhang Renjie, pl.VI-3
Baiera Braun, 1843 emend. Florin, 1936
Baiera? *minor* Feng and Zhang, 1999 1999 Feng Shaonan et Zhang Renjie, pl.I-4
Barrandeina (Krejči) Stur, 1882
Barrandeina dusliana (Krejči) Stur 1999 Feng Shaonan et Zhang Renjie, pl.I-1; pl.II-1-3
Barsassia Zalessky, 1933
Barsassia sibirica (Krysht.) Zalessky, emend. Dou et Sun 1999 Feng Shaonan et Zhang Renjie, pl.II-5
Czekanowskia Heer, 1876
Czekanowskia? *zhoupingensis* Feng et Zhang, 1999 1999 Feng Shaonan et Zhang Renjie, pl.IV-4-5
Longostachys Zhu, Hu et Feng 1983
Longostachys latisporophyllus Zhu, Hu et Feng, 1983 1999 Feng Shaonan et Zhang Renjie, p. 36
Minarodendron Li, 1990
Minarodendron cathaysiense (Schweitzer et Cai) Li, 1990 1999 Feng Shaonan et Zhang Renjie, pl.I-2-3
Protopteridium Krejči, 1880
"Protopteridium" minutum Halle [possibly *Eocladoxylon minutum* (Halle) Koidzumi emend. Berry et Wang, 2006] 1999 Feng Shaonan et Zhang Renjie, pl.VI-3; pl. VI-1
Protopteridium scharyanum Krejči 1999 Feng Shaonan et Zhang Renjie, p. 35
Psilophytites Høeg, 1952
Psilophytites? sp. 1999 Feng Shaonan et Zhang Renjie, p. 36
Psilophyton Dawson, 1859
Psilophyton sp. 1999 Feng Shaonan et Zhang Renjie, p. 36
Sphenobaiera Florin, 1936

Sphenobaiera? sp. 1999 Feng Shaonan et Zhang Renjie, pl.II-6
Taeniocrada White, 1902
Taeniocrada sp. 1999 Feng Shaonan et Zhang Renjie, p. 36
Xihuphyllum Chen, 1988
Xihuphyllum sp. 1999 Feng Shaonan et Zhang Renjie, pl.V, 1-3

Hunan

1. Gugang Section, Liuyang County: Tiaomajian Formation
Minarodendron Li, 1990
Minarodendron cathaysiense (Schweitzer et Cai) Li, 1990 1987 Schweitzer et Cai, pls.I-II; fig.4a-d; 1995 Cai Chongyang et Li Xingxue, pl. IV, 1-5

2. Guankou Section, Liuyang County: Tiaomajian Formation
Knorria Sternberg, 1825
Knorria sp. 1982 Cheng Lizhu, pl.326, 1, 1a
Lepidodendropsis Lutz, 1933
Lepidodendropsis tiaomaensis Feng, 1977 1982 Cheng Lizhu, pl.324, 5, 6
Minarodendron Li, 1990
Minarodendron cathaysiense (Schweitzer et Cai) Li, 1990 1982 Cheng Lizhu, pl.323, 8,8a

3. Tiaomajian Section, Changsha City: Tiaomajian Formation
Lepidodendropsis Lutz, 1933
Lepidodendropsis arborescens (Sze) Sze 1982 Cheng Lizhu, pl.324, 1
Lepidodendropsis cf. *arborescens* (Sze) Sze 2003 Wang Hongfeng, pl.1, 31
Lepidodendropsis tiaomaensis Feng, 1977 1977 Feng Shaonan et al., pl.230, 11; 1982 Cheng Lizhu, pl.324, 5, 6

4. Qilichong Section, Yiyang County: Tiaomajian Formation
Minarodendron Li, 1990
Minarodendron cathaysiense (Schweitzer et Cai) Li, 1990 1977 Feng Shaonan et al., pl.230, 7, 8; 1974 Gu et Zhi, pl.3, 7-11; fig.22-24

5. Zhuzhou: Tiaomajian Formation
Lepidodendropsis Lutz, 1933
Lepidodendropsis tiaomaensis Feng, 1977 1977 Feng Shaonan et al., pl.230, 11; 1982 Cheng Lizhu, pl.324, 5, 6
Lepidodendropsis guanzhuangensis Feng et Meng 1977 Feng Shaonan et al., pl.231, 3-6; 1982 Cheng Lizhu, pl.324, 10, 10a

6. Hengyang: Tiaomajian Formation
Lepidodendropsis Lutz, 1933
Lepidodendropsis tiaomaensis Feng, 1977 1977 Feng Shaonan et al., pl.230, 11

7. Niu’egou Section, Yiyang County: Tiaomajian Formation
Lepidodendropsis Lutz, 1933
Lepidodendropsis niuewanensis Zhang, 1977 1977 Feng Shaonan et al., pl.230, 9-10; 1982 Cheng Lizhu, pl.324, 11, 12

<u>*Minarodendron* Li, 1990</u>

Minarodendron cathaysiense (Schweitzer et Cai) Li, 1990 1982 Cheng Lizhu, pl.323, 8,8a; 1977 Feng Shaonan et al., pl.230, 7, 8

8. Changsha County: Tiaomajian Formation

<u>*Lepidodendropsis* Lutz, 1933</u>

Lepidodendropsis arborescens (Sze) Sze 1954 Sze et Hsü, pl. 8, 2-4; 1974 Gu et Zhi, pl.5, 3-4; 1977 Feng Shaonan et al., pl.231, 13

<u>*Protopteridophyton* Li et Hsü, 1987</u>

Protopteridophyton devonicum Li et Hsü, 1987 1987 Li et Hsü, pls.1-16; figs.1-5

9. Huangnitang Section, Xintian County: Qiziqiao Formation

<u>*Platyphyllum* Dawson, 1888</u>

Platyphyllum cf. *fuellingii* Kräusel et Weyland 1982 Cheng Lizhu, pl.333, 7

10. Shanmen Reservoir, Li County: Yuntaiguan Formation

<u>*Barrandeina* (Krejči) Stur, 1882</u>

Barrandeina contigua Zhu, 1983 1983 Zhu Jianan et al., pl.I, 14

Barrandeina dusliana (Krejči) Stur 1984 Feng Shaonan, pl.46, 12

Barrandeina laxa Zhu, 1983 1983 Zhu Jianan et al., pl.I, 5, 6

Barrandeina lixianensis Zhu, 1983 1983 Zhu Jianan et al., pl.I, 13

Barrandeina multistriata Zhu, 1983 1983 Zhu Jianan et al., pl.I, 11-12

<u>*Longostachys* Zhu, Hu et Feng, 1983</u>

Longostachys latisporophyllus Zhu, Hu et Feng emend. Cai et Cheng, 1996 1983 Zhu Jianan et al., pl.I, 1; 1996 Cai Chongyang et Cheng Lizhu, pl.1-20; figs.1-16

<u>*Minarodendron* Li, 1990</u>

Minarodendron cathaysiense (Schweitzer et Cai) Li, 1990 1984 Feng Shaonan, pl.46, 6; 1988 Feng Shaonan et Ma Jie, pl.II, 1

<u>*Protolepidodendron* Krejči, 1880 ex Gothan, 1921</u>

Protolepidodendron? *lixianense* Feng et Ma, 1988 1988 Feng Shaonan et Ma Jie, pl.I, 1, 5; fig.2

"Protolepidodendron" minutum Halle [possibly *Eocladoxylon minutum* (Halle) Koidzumi emend. Berry et Wang, 2006] 1984 Feng Shaonan, pl.46, 10

<u>*Psilophytites* Høeg, 1952</u>

Psilophytites sp. 1984 Feng Shaonan, pl.46, 11

11. Central Hunan: Tiaomajian Formation

<u>*Protolepidodendron* Krejči, 1880 ex Gothan, 1921</u>

Protolepidodendron? *arborecens* Sze 1937 Sze, pl.I; figs.7, 8

<u>*Psilophytites* Høeg, 1952</u>

Psilophytites sp. 1937 Sze, pl.I; figs.1, 2

cf. *Psilophytites* sp. 1937 Sze, pl.I; figs.3-5

Psilophytites? sp. 1937 Sze, pl.I; figs.6

12. Frontiers of Hunan and Hubei: Yuntaiguan Formation

<u>*Barrandeina* (Krejči) Stur, 1882</u>

Barrandeina dusliana (Krejči) Stur 1985 Feng Shaonan, p.40

Barrandeina contigua Zhu, 1983 1985 Feng Shaonan, p.40
Barrandeina lixianensis Zhu, 1983 1985 Feng Shaonan, p.40
Barrandeina laxa Zhu, 1983 1985 Feng Shaonan, p.40
Barrandeina multistriata Zhu, 1983 1985 Feng Shaonan, p.40
Drepanophycus Göppert, 1852
Drepanophycus spinaeformis Göppert, 1852 1985 Feng Shaonan, p.40
Longostachys Zhu, Hu et Feng, 1983
Longostachys latisporophyllus Zhu, Hu et Feng emend. Cai et Cheng, 1996 1985 Feng Shaonan, p.40
Minarodendron Li, 1990
Minarodendron cathaysiense (Schweitzer et Cai) Li, 1990 1985 Feng Shaonan, p.40
Protopteridium Krejči 1880
"Protopteridium" minutum Halle [possibly *Eocladoxylon minutum* (Halle) Koidzumi emend. Berry et Wang, 2006] 1985 Feng Shaonan, p.40
Protopteridium thomsonii 1985 Feng Shaonan, p.40
Psilophytites Høeg, 1952
Psilophytites sp. 1985 Feng Shaonan, p.40

13. Hongjiayu Section, Sangzhi County: Lower Yuntaiguan Formation
Only plant fragments 1982 Cai Chongyang et Li Xingxue

Jiangxi

1. Xiashan Section, Ganzhou City: Yunshan Formation
Barrandeina Stur, 1882
Barrandeina dusliana (Krejči) Stur 1982 Li Hanmin et al., pl.131, 9
Dawsonites Halle, 1916
Dawsonites(*Psilophyton*)? *jiangxiensis* Lee, 1982 1982 Li Hanmin et al., pl.137, 3
Lepidodendropsis Lutz, 1933
Lepidodendropsis arborescens (Sze) Sze 1987 Schweitzer et Cai, pls. VI, 1-10; VII, 1-3; 1995 Cai Chongyang et Li Xingxue, pl. IV, 8
Lepidodendropsis cf. *arborescens* (Sze) Sze 2003 Wang Hongfeng, pl.1, 31
Minarodendron Li, 1990
Minarodendron cathaysiense (Schweitzer et Cai) Li, 1990 1982 Li Hanmin et al., pl.130, 4-5

2. Jinggangshan: Tiaomajian Formation
Minarodendron Li, 1990
Minarodendron cathaysiense (Schweitzer et Cai) Li, 1990 1982 Li Hanmin et al., pl.130, 4-5
Taeniocrada White, 1902
Taeniocrada sp. 1982 Li Hanmin et al., pl.129, 5

3. Doushui Section, Shangyou County: Tiaomajian Formation
Minarodendron Li, 1990
Minarodendron cathaysiense (Schweitzer et Cai) Li, 1990 1982 Li Hanmin et al., pl.130, 4-5

Yunnan

1. Maliaohe Section, Luquan County: Haikou Formation

Cyclostigma Haughton ex Heer, 1871

Cyclostigma? *kiltorkens* 1997 Quan Biao et Han Dexin, p.92

Drepanophycus Göppert, 1852

Drepanophycus spinaeformis Göppert, 1852 1997 Quan Biao et Han Dexin, p.92

Lepidodendropsis Lutz, 1933

Lepidodendropsis arborescens (Sze) Sze 1997 Quan Biao et Han Dexin, p.92

Lepidodendropsis cf. *sinensis* 1997 Quan Biao et Han Dexin, p.92

Taeniocrada White, 1902

Taeniocrada? *decheniana* (Göppert) Kräusel et Weyland 1997 Quan Biao et Han Dexin, p.92

Sublepidodendron (Nathorst) Hirmer, 1927 emend. Wang Q, Hao, Wang DM, Wang Y et Thomas Denk, 2003

Sublepidodendron sp. 1997 Quan Biao et Han Dexin, p.92

2. Wujian Village, Maoshan Town, Luquan County: Upper Haikou Formation

Eocladoxylon (Koidzumi) Berry and Wang, 2006

Eocladoxylon minutum (Halle) Koidzumi emend. Berry et Wang, 2006 2007 Sun Dewei et al., p. 356

Lepidodendropsis Lutz, 1933

Lepidodendropsis arborescens (Sze) Sze 2007 Sun Dewei et al., p. 356

Minarodendron Li, 1990

Minarodendron cathaysiense (Schweitzer et Cai) Li, 1990 2007 Sun Dewei et al., p. 356

Panxia Wang et Berry, 2006

Panxia gabata Wang et Berry, 2006 2007 Sun Dewei et al., p. 356

3. Liukongqiao Section, Luquan County: Xichong Formation

Lepidodendropsis Lutz, 1933

Lepidodendropsis? *sinensis* (Hsü) Gu et Zhi 1987 Schweitzer et Cai, pl. VIII, 1-4

4. Longhuashan Section, Zhanyi County: Haikou Formation

Drepanophycus Göppert, 1852

Drepanophycus qujingensis Li et Edwards, 1995 1966 Hsü, p. 228

Eocladoxylon Koidzumi emend. Berry et Wang, 2006

Eocladoxylon minutum (Halle) Koidzumi emend. Berry et Wang, 2006 1966 Hsü, pl.III, 1-4; pl. IV, 2-7; pl.V, 2-9; pl.VI, 5-8; fig.6-12; 1974 Gu et Zhi, pls. II, 8-14; III, 1; 1974 Li Xingxue et Deng Longhua, pl.127, 1-3

Minarodendron Li, 1990

Minarodendron cathaysiense (Schweitzer et Cai) Li, 1990 1966 Hsü, pl. III, 1; 1974 Gu et Zhi, pl. III, 7-11; 1974 Li Xingxue et Deng Longhua, pl.127, 6, 7; 1990 Li, pls.1-11; figs.1-8

Psilophytites Høeg, 1952

Psilophytites sp.2 1974 Gu et Zhi, pl. II, 6-7

Sporogonites Halle, 1916

Sporogonites yunnanense Hsü, 1966 1966 Hsü, pls. III, 5-6; IV, 1; VI, 1-4; Figs. 13-14; 1974 Gu et Zhi, pl. I, 1-3

5. Yuguang Section, Zhanyi County: Haikou Formation

Lepidodendropsis Lutz, 1933

Lepidodendropsis sp. 2007b Hao et al., fig. 1

Minarodendron Li, 1990

Minarodendron cathaysiense (Schweitzer et Cai) Li, 1990 2007b Hao et al., fig. 1

Yuguangia Hao et al., 2007

Yuguangia ordinata Hao et al., 2007 2007b Hao et al., figs.2-8

6. Huaning: Haikou Formation

Minarodendron Li, 1990

Minarodendron cathaysiense (Schweitzer et Cai) Li, 1990 1974 Gu et Zhi, pl. III, 7-11

7. Zhaotong City: Haikou Formation

Psilophytites Høeg, 1952

Psilophytites sp. 1974 Li Xingxue et Deng Longhua, pl.127, 8

Psilophytites sp.1 1974 Gu et Zhi, pl. II, 4-5

8. Tongdi: Haikou Formation

Drepanophycus Göppert, 1852

Drepanophycus spinaeformis Göppert, 1852 1974 Gu et Zhi, pl. III, 2-6

9. Kunming: Haikou Formation

Minarodendron Li, 1990

Minarodendron cathaysiense (Schweitzer et Cai) Li, 1990 1974 Gu et Zhi, pl. III, 7-11

10. Wangjiazhuang Reservoir, Erjie Town, Jinning County, Kunming City: Haikou Formation

Kunia Xiong et al., 2012

Kunia vernusta Xiong et al., 2012 2012 Xiong et al., figs. 1-5

11. Longhuashan Section, Zhanyi County: Xichong Formation

Lepidodendropsis Lutz, 1933

Lepidodendropsis kazachstanica Senkevitsch 1987 Schweitzer et Cai, pls.VII, 4-10; X, 1; 1995 Cai Chongyang et Li Xingxue, pl. IV, 7

Minarodendron Li, 1990

Minarodendron cathaysiense (Schweitzer et Cai) Li, 1990 1987 Schweitzer et Cai, pls.I-II; fig.4a-d; 1995 Cai Chongyang et Li Xingxue, pl. IV, 1-5

12. Yuguang Section, Zhanyi County: Xichong Formation

Panxia Wang et Berry, 2006

Panxia gabata Wang et Berry, 2006 2006 Wang et Berry, figs.3-7

13. Xichong Section, Zhanyi County: Xichong Formation

Barrandeina Stur, 1882

Barrandeina cf. *dusliana* Stur 1987 Schweitzer et Cai, pls.XVI, 1-10; XVII, 1-10; XVIII, 1-4; tab.XIX, 1-2

Eocladoxylon Koidzumi emend. Berry et Wang, 2006

Eocladoxylon minutum (Halle) Koidzumi emend. Berry et Wang, 2006 1987 Schweitzer et Cai, pl. XII, 1-4; 2006b Berry et Wang, figs.1-14

Lepidostrobus Brongniart, 1828 emend. Brack-Hanes et Thomas, 1983

Lepidostrobus? *dentatus* Schweitzer et Cai 1987 1987 Schweitzer et Cai, pls. VIII, 5-8; IX, 1-4

Minarodendron Li, 1990

Minarodendron cathaysiense (Schweitzer et Cai) Li, 1990 2006a Berry et Wang, p. 64

Psilophyton Dawson, 1859

Psilophyton? *striatum*, 2001 2001b Wang et Berry, pl. I, 1-13; fig.3

Rhipidophyton Berry et Wang, 2006

Rhipidophyton acanthum Berry et Wang, 2006 2006a Berry et Wang, pls.1-3; figs.2-6

14. Shijiapo Section, Zhanyi County: Xichong Formation

Barrandeina Stur, 1882

Barrandeina cf. *dusliana* Stur 1982 Cai et Li, p. 113

Colpodexylon Schweitzer et Cai, 1987

Colpodexylon variabile Schweitzer et Cai, 1987 1987 Schweitzer et Cai, pls. III-IV; V, 1-3; 1995 Cai Chongyang et Li Xingxue, pl. V, 1-3

Minarodendron Li, 1990

Minarodendron cathaysiense (Schweitzer et Cai) Li, 1990 1982 Cai et Li, p. 113; 1995 Cai Chongyang et Li Xingxue, pl. IV, 1-5

15. Taoyuan Section, Zhanyi County: Xichong Formation

Barrandeina Stur, 1882

Barrandeina cf. *dusliana* Stur 1982 Cai et Li, p. 113

Lepidosigillaria Kräusel et Weyland, 1949

Lepidosigillaria? *Cycloformis*, 1987 1987 Schweitzer et Cai, pl.X, 2-8; 1995 Cai Chongyang et Li Xingxue, pl. VI, 6, 7

Minarodendron Li 1990

Minarodendron cathaysiense (Schweitzer et Cai) Li, 1990 1982 Cai et Li, p. 113; 1995 Cai Chongyang et Li Xingxue, pl. IV, 1-5

16. Yangliuhe Section, Wuding County: Xichong Formation

Rhipidophyton Berry et Wang, 2006

Rhipidophyton sp. 2006a Berry et Wang, p. 64

17. Xinsun Village, near Yangliuhe, Wuding County: Xichong Formation

Dawsonites Halle, 1916

Dawsonites(*Psilophyton*) sp. B 1987 Schweitzer et Cai, pl.XX, 2-4

Eocladoxylon Koidzumi Berry et Wang, 2006

Eocladoxylon minutum (Halle) Koidzumi emend. Berry et Wang, 2006 2003 Wang et Berry, p. 439

Lepidodendropsis Lutz, 1933

Lepidodendropsis arborescens (Sze) Sze 2003 Wang et Berry, p. 439

Lepidodendropsis kazachstanica Senkevitsch 1987 Schweitzer et Cai, pls.VII, 4-10; X, 1

Minarodendron Li, 1990

Minarodendron cathaysiense (Schweitzer et Cai) Li, 1990 2003 Wang et Berry, p. 439

<u>*Protolepidodendron* Krejči ex Gothan, 1921</u>

Protolepidodendron? sp. 2003 Wang et Berry, p. 439

<u>*Rhipidophyton* Berry et Wang, 2006</u>

Rhipidophyton sp. 2006a Berry et Wang, p. 65

<u>*Tauritheca* Wang et Berry, 2003</u>

Tauritheca cornuta (Schweitzer et Cai) Wang et Berry, 2003 1987 Schweitzer et Cai, pl.XIX, 3-6; 1995 Cai Chongyang et Li Xingxue, pl. IV, 6; 2003 Wang et Berry, figs.1-7

<u>*Tsaia* Wang et Berry, 2001</u>

Tsaia denticulata Wang et Berry, 2001 2001a Wang et Berry, pl.I-II, figs.2-5

18. Mile County: Huaning Formation

Pseudosporochnus? sp. (possibly *Rhipidophyton acanthum*) 1987 Schweitzer et Cai, pl. XIII, 1

Pseudosporochnus? *heteroramis* (possibly *Rhipidophyton acanthum*) 1987 Schweitzer et Cai, pls.XIII, 2-8; XIV, 1-3

19. Panxi Section, Huaning County: Huaning Formation

<u>*Barrandeina* Stur, 1882</u>

Barrandeina cf. *dusliana* Stur 1982 Cai et Li, p. 113

<u>*Eocladoxylon* Koidzumi emend. Berry et Wang, 2006</u>

Eocladoxylon minutum (Halle) Koidzumi emend. Berry et Wang, 2006 1987 Schweitzer et Cai, pl. XV, 2-7

<u>*Minarodendron* Li, 1990</u>

Minarodendron cathaysiense (Schweitzer et Cai) Li, 1990 1982 Cai et Li, p. 113

<u>*Panxia* Wang et Berry, 2006</u>

Panxia gabata Wang et Berry, 2006 1987 Schweitzer et Cai, pls.XIV, 4-6;.XV, 1; 2006 Wang et Berry, figs.3-7

Sichuan

1. Ganxi Section, Beichuan County: Jinbaoshi Formation

<u>*Archaeopteris* (Dawson) Stur, 1875</u>

Archaeopteris sp. 2003 Wang Hongfeng, pl.2, 12-13, 15-16

<u>*Colpodexylon* Schweitzer et Cai, 1987</u>

?*Colpodexylon* sp. 2003 Wang Hongfeng, pl.1, 20

<u>*Cyclostigma* Haughton ex Heer, 1871</u>

Cyclostigma sp. 2003 Wang Hongfeng, pl.1, 26

<u>*Eocladoxylon*Koidzumi emend. Berry et Wang, 2006</u>

Eocladoxylon minutum (Halle) Koidzumi emend. Berry et Wang, 2006 2003 Wang Hongfeng, pl.1, 13-19

<u>*Lepidodendropsis* Lutz, 1933</u>

Lepidodendropsis cf. *arborescens* (Sze) Sze 2003 Wang Hongfeng, pl.1, 31

Lepidodendropsis cf. *hirmeri* Lutz 2003 Wang Hongfeng, pl.1, 29-30

Lepidodendropsis theodori (Zalessky) Jongmans 2003 Wang Hongfeng, pl.1, 27-28

Lepidodendropsis cf. *wutubulakensis* Dou et Sun 2003 Wang Hongfeng, pl.1, 32-35

<u>*Minarodendron* Li, 1990</u>

Minarodendron cf. *cathaysiense* (Schweitzer et Cai) Li, 1990 2003 Wang Hongfeng, pl.1, 21-25

<u>*Prelepidodendron* Danzé-Corsin, 1958</u>

Prelepidodendron? *beichuanensis* Wang, 2003 2003 Wang Hongfeng, pl.2, 24-26

Prelepidodendron? *jinbaoshiensis* Wang, 2003 2003 Wang Hongfeng, pl.2, 7-9
Prelepidodendron? cf. *jinbaoshiensis* Wang, 2003 2003 Wang Hongfeng, pl.2, 1-3
Prelepidodendron? *sichuanensis* Wang, 2003 2003 Wang Hongfeng, pl.2,10-11
Prelepidodendron? sp. 2003 Wang Hongfeng, pl.2, 4-6
Psilophyton Dawson, 1859
Psilophyton bellum (Tschirkova) Dou 2003 Wang Hongfeng, pl.1, 11-12; 2010 Sun et al., pl. 21, 8, 9
Psilophyton sp. 2003 Wang Hongfeng, pl.1, 7-10
Syringodendron Sternberg, 1820
Syringodendron sp. 3 2003 Wang Hongfeng, pl.2, 18-19
Syringodendron sp. 4 2003 Wang Hongfeng, pl.2, 20 ?
Taeniocrada White, 1902
Taeniocrada decheniana (Göppert) Kräusel et Weyland 2003 Wang Hongfeng, pl.1, 1-6

2. Longmenshan, northwestern Sichuan: Guanwushan Formation
Lepidodendropsis Lutz, 1933
Lepidodendropsis sp. 1982 Cai et Li, p. 113
Minarodendron Li, 1990
Minarodendron cathaysiense (Schweitzer et Cai) Li, 1990 1982 Cai et Li, p. 113

3. Shangsi Section, Guangyuan City: Guanwushan Formation
Lepidodendropsis Lutz, 1933
Lepidodendropsis sp. 1982 Cai et Li, p. 114
Lepidostrobus Brongniart, 1828 emend. Brack-Hanes et Thomas, 1983
Lepidostrobus? sp. 1982 Cai et Li, p. 114

Guangxi

1. Quanzhou: Lower Donggangling Formation
Lepidodendropsis Lutz, 1933
Lepidodendropsis sp. 1982 Cai et Li, p. 114

2. Luzhai: Lower Donggangling (Tungkangling) Formation
Barrandeina Stur, 1882
Barrandeina dusliana Krejči ex Stur 1995 Cai Chongyang et Li Xingxue, pl. V, 5

3. Luocheng: Upper Middle Devonian
Haplostigma Seward, 1932
Haplostigma sp. 1982 Cai et Li, p. 114
Lepidodendropsis Lutz, 1933
Lepidodendropsis sp. 1982 Cai et Li, p. 114
Minarodendron Li, 1990
Minarodendron cathaysiense (Schweitzer et Cai) Li, 1990 1982 Cai et Li, p. 114

Guangdong

Ruyuan: Upper Guitou Formation

Lepidodendropsis Lutz, 1933

Lepidodendropsis sp. 1982 Cai et Li, p. 114

Protolepidodendron Krejči, 1880 ex Gothan, 1921

Protolepidodendron? sp. 1982 Cai et Li, p. 114

Tibet

Tuoba, Changdu, and Mangkang: Dingzonglong Formation

No land-plant record 1982 Cai Chongyang et Li Xingxue

Vietnam

2 localities, South China Plate: Duong Dong Formation (Givetian?)

Some basal euphyllophytes 2012 Gonez et al., tab. 1.

Frasnian (Late Devonian)

Hubei

1. Miliangshan Section, Wuhan City: Luojia Series, layer 1–5

Chamaedendron Schweitzer et Li, 1996

Chamaedendron multisporangiatum Schweitzer et Li, 1996 1996 Schweitzer et Li, pls.1-4; 2000 Li Chengsen, fig. 3

Cyclostigma Haughton ex Heer, 1871

Cyclostigma kiltorkense Haughton ex Heer, 18711974 Gu et Zhi, pl.3, 12-13; pl.4, 1; 2000 Li Chengsen, fig. 3

Lepidostrobus Brongniart, 1828 emend. Brack-Hanes et Thomas, 1983

Lepidostrobus? sp. 2000 Li Chengsen, fig. 3

Leptophloeum Dawson, 1861 emend. Li, Dou et Sun, 1986

Leptophloeum rhombicum Dawson 2000 Li Chengsen, fig. 3

Protopteridophyton Li et Hsü, 1987

Protopteridophyton devonicum Li et Hsü, 1987 1987 Li et Hsü, pls.1-16; figs.1-5

Sphinxiocarpon Wang, Xue et Prestianni, 2007

Sphinxiocarpon wuhanium (Li, Hilton et Hemsley) Wang, Xue et Prestianni 1997 Li Chengsen et al., figs.1-24; 2000 Li Chengsen, fig. 3; 2007 Wang Q et al., p. 393; 2008 Wang et al., p. 301

Miliangshan Section, Wuhan City: Luojia Series, layer 6

Asterocalamites Schimper ex Zeiller, 1879

Asterocalamites sp. 2000 Li Chengsen, fig. 3

Cyclostigma Haughton ex Heer, 1871

Cyclostigma kiltorkense Haughton ex Heer, 1871 2000 Li Chengsen, fig. 3

Leptophloeum Dawson, 1861 emend. Li, Dou et Sun, 1986

Leptophloeum rhombicum Dawson 2000 Li Chengsen, fig. 3

Sphenopteris (Brongniart) Sternberg, 1825

Sphenopteris recurva? 2000 Li Chengsen, fig. 3

Sphenophyllum Brongniart, 1828

Sphenophyllum? sp. 2000 Li Chengsen, fig. 3

Sublepidodendron (Nathorst) Hirmer, 1927 emend. Wang Q, Hao, Wang DM, Wang Y et Thomas Denk, 2003

Sublepidodendron grabaui (Sze) Wang et Xu 2000 Li Chengsen, fig. 3

Miliangshan Section, Wuhan City: Wutung Formation

Cyclostigma Haughton ex Heer, 1871

Cyclostigma kiltorkense Haughton ex Heer, 1871 1977 Feng Shaonan et al., pl.230,2-4; 1984 Chen Gongxin, pl. 218, 8

Lepidodendropsis Lutz, 1933

Lepidodendropsis yangtziensis Chen, 1977 1977 Feng Shaonan et al., pl.233, 4; 1984 Chen Gongxin, pl. 217, 4

Leptophloeum Dawson, 1861 emend. Li, Dou et Sun, 1986

Leptophloeum rhombicum Dawson 1977 Feng Shaonan et al., pl.230, 15; 1984 Chen Gongxin, pl. 216, 4-6

Sublepidodendron (Nathorst) Hirmer, 1927 emend. Wang Q, Hao, Wang DM, Wang Y et Thomas Denk, 2003

Sublepidodendron mirabile (Nathorst) Hirmer, 1927 1984 Chen Gongxin, pl. 218, 7

Sublepidodendron wuhanense Chen, 1977 1977 Feng Shaonan et al., pl.231, 15; 1984 Chen Gongxin, pl. 217, 6

Syringodendron Sternberg 1820

Syringodendron hanyangense Chen, 1977 1977 Feng Shaonan et al., pl.234, 1-3; 1984 Chen Gongxin, pl. 217, 5

2. Xiannüshan Quarry, near Wuhan City: Wutung Formation

Lepidodendropsis Lutz, 1933

Lepidodendropsis hirmeri Lutz 2012 Xu Honghe et al., p. 131

Leptophloeum Dawson, 1861 emend. Li, Dou et Sun, 1986

Leptophloeum rhombicum Dawson 2012 Xu Honghe et al., p. 131

Sphinxiocarpon Wang, Xue et Prestianni, 2007

Sphinxiocarpon wuhanium (Li, Hilton et Hemsley) Wang, Xue et Prestianni 2012 Xu Honghe et al., fig. 3, A-F

Sublepidodendron (Nathorst) Hirmer, 1927 emend. Wang Q, Hao, Wang DM, Wang Y et Thomas Denk, 2003

Sublepidodendron grabaui (Sze) Wang et Xu 2012 Xu Honghe et al., p. 131

Sublepidodendron songziense Chen ex Wang Q, Hao, Wang DM, Wang Y, et Thomas Denk. 2012 Xu Honghe et al., p. 131

3. Guishan and Sheshan, Wuhan City: Wutung Formation

Leptophloeum Dawson, 1861 emend. Li, Dou et Sun, 1986

Leptophloeum rhombicum Dawson 1977 Feng Shaonan et al., pl.230, 15

4. Lan'ganya Section, Changyang County: Huangjiadeng Fomation

Archaeopteris (Dawson) Stur, 1875

Archaeopteris macilenta (Lesquereux) Carluccio, Hueber et Banks, 1966 1981 Cai Chongyang, pl. I, 1-7; 1984 Chen Gongxin, pls. 217, 8-10; 218, 6; 1984 Feng Shaonan, pl.46, 1-3; 4-5; 8; 13-19; 1987 Hao Shougang et Mei Shilong, pls. I, 1-8; II, 1-11; 1995 Cai Chongyang et Li Xingxue, pl. VII, 3, 4; 2011 Guo Yun and Wang Deming, figs. 2-7

Cyclostigma Haughton ex Heer, 1871

Cyclostigma kiltorkense Haughton ex Heer, 1871 1984 Feng , pl.47, 1-3

Denglongia Xue et Hao, 2008

Denglongia hubeiensis Xue et Hao, 2008 2008 Xue et Hao, figs.1-14; 2010 Xue Jinzhuang et al., figs. 1-6

Gilboaphyton Arnold, 1937

Gilboaphyton? *changyangense*, Feng 1984 1984 Feng Shaonan, pl.47, 5

Leptophloeum Dawson, 1861 emend. Li, Dou et Sun, 1986

Leptophloeum rhombicum Dawson 1984 Feng , pl.46, 9

Longostachys Zhu, Hu et Feng, 1983

cf. *Longostachys* sp. 2007 Xue jinzhuang et Hao Shougang, text-figs.1-5; 2008 Xue et Hao, p. 1315

Metacladophyton Wang et Lin, 2007

cf. *Metacladophyton* sp. 2010 Xue Jinzhuang et al., p. 108; 2008 Xue et Hao, p. 1315

Sphinxiocarpon Wang, Xue and Prestianni

Sphinxiocarpon wuhanium (Li, Hilton and Hemsley) Wang, Xue and Prestianni 2010 Xue, figs. 1-4

5. Huangjiadeng Section, Changyang County: Huangjiadeng Fomation

Aphyllopteris Arnold, 1939

Aphyllopteris? sp. 1952 Sze, pl. VI, 5, 6

Cordaites Unger, 1850

Cordaites? sp. 1952 Sze, pl. VIII, 13-15

Cyclostigma Haughton ex Heer, 1871

Cyclostigma kiltorkense Haughton 1952 Sze, pls. IV, 6, 6a; VI, 2, 2a; 1984 Chen Gongxin, pl. 218, 8

Lepidodendropsis Lutz, 1933

Lepidodendropsis? sp. 1952 Sze, pls. IV, 7; VI, 3; VII, 2, 2a

Leptophloeum Dawson, 1861 emend. Li, Dou et Sun, 1986

Leptophloeum rhombicum Dawson 1952 Sze, pls. I, 1-6; II, 1-3; III, 1-4; IV, 3-5; 1984 Chen Gongxin, pl. 216, 4-6

Rhacophyton Crépin, 1875

Rhacophyton sp. 1952 Sze, pl. VIII, 9

Sphenopteris (Brongniart) Sternberg, 1825

Sphenopteris? *recurva* Dawson 1952 Sze, pl. VIII, 1-7

Sublepidodendron (Nathorst) Hirmer, 1927 emend. Wang Q, Hao, Wang DM, Wang Y et Thomas Denk, 2003

Sublepidodendron grabaui (Sze) Wang et Xu 1984 Chen Gongxin, pl. 217, 1

6. Yingfengnao Section, Changyang County: Huangjiadeng Fomation

Cyperites Lindley et Hutton, 1832

Cyperites? sp. 1952 Sze, pls. VII, 4, 5; VIII, 12

7. Near Changyang County: Huangjiadeng Formation

Changyanophyton Sze

Changyanophyton hupiense Sze 1954 Sze et Hsü, pl. 2, 4-8

Lepidodendropsis Lutz, 1933

Lepidodendropsis arborescens (Sze) Sze 1984 Chen Gongxin, pl. 216, 1, 2

Leptophloeum Dawson, 1861 emend. Li, Dou et Sun, 1986

Leptophloeum rhombicum Dawson 1954 Sze et Hsü, pl. 1, 10-11; 1977 Feng et al., pl.230, 15

Sphenopteris (Brongniart) Sternberg, 1825

Sphenopteris? *recurva* Dawson 1974 Gu et Zhi, pl.51, 1-2; 1984 Chen Gongxin, pl. 218, 9

Sublepidodendron (Nathorst) Hirmer, 1927 emend. Wang Q, Hao, Wang DM, Wang Y et Thomas Denk, 2003

Sublepidodendron grabaui (Sze) Wang et Xu 2005 1974 Gu et Zhi, pl.16, 7-8; 1977 Feng Shaonan et al., pl.234, 12-13

8. Guanzhuang, Yichang City: Huangjiadeng Fomation

Cyclostigma Haughton ex Heer, 1871

Cyclostigma kiltorkense Haughton ex Heer, 1871 2010 Peng et al., pl. II, 6-8

Lepidodendropsis Lutz, 1933

Lepidodendropsis arborescens (Sze) Sze 2010 Peng et al., pl. II, 1-3

Leptophloeum Dawson, 1861 emend. Li, Dou et Sun, 1986

Leptophloeum rhombicum Dawson 2010 Peng et al., pls. I, 1-2; II, 10

Minarodendron Li, 1990

Minarodendron cf. *cathaysiense* (Schweitzer et Cai) Li, 1990 2010 Peng et al., pl. II, 9

Stigmaria Brongniart, 1822

Stigmaria ficoides (Sternberg) Brongniart 2010 Peng et al., pl. II, 5

Sublepidodendron (Nathorst) Hirmer, 1927 emend. Wang Q, Hao, Wang DM, Wang Y et Thomas Denk, 2003

Sublepidodendron sp. 2010 Peng et al., pl. II, 4

9. Lijiawan Section, Wufeng County: Huangjiadeng Fomation

Polypetalophyton Geng ex Hilton, Geng et Kenrick, 2003

Polypetalophyton wufengensis Geng ex Hilton, Geng et Kenrick, 2003 2003 Hilton J et al., figs.1-10

10. Liuping Section, Changyang County: Huangjiadeng Fomation

Metacladophyton Wang Z et Geng, 1997

Metacladophyton tetraxylum Wang Z et Geng, 1997 1997 Wang Zhong et Geng Baoyin, pl.1-11; figs.1-7

11. Huaibaoshi Section, Zigui County: Huangjiadeng Fomation

Archaeocalamites Stur, 1875

Archaeocalamites radiates Brongniart 2000 Feng Shaonan et Zhang Renjie, p. 36

Archaeopteris (Dawson) Stur, 1875

Archaeopteris macilenta (Lesquereux) Carluccio, Hueber et Banks, 1966 2000 Feng Shaonan et Zhang Renjie, p. 36

Metacladophyton Wang et Lin, 2007

Metacladophyton tetraxylum Wang Z et Geng, 1997 2000 Feng Shaonan et Zhang Renjie, p. 36

Metacladophyton ziguinum Wang et Lin, 2007 2007 Wang DM et Lin, figs.1-14

12. Meitangou Section, Songzi County: Huangjiadeng Fomation

Lepidodendropsis Lutz, 1933

Lepidodendropsis arborescens (Sze) Sze 1952 Sze, pl.VI, 1, 1a; 1954 Sze et Hsü, pl.2, 3; pl.8, 2-4; pl.9, 2-3

13. Frontiers of Hunan and Hubei: Huangjiadeng Formation

Archaeopteris (Dawson) Stur, 1875
Archaeopteris macilenta (Lesquereux) Carluccio, Hueber et Banks, 1966 1985 Feng Shaonan, p.41
Cyclostigma Haughton ex Heer, 1871
Cyclostigma kiltorkense Haughton ex Heer, 18711985 Feng Shaonan, p.41
Gilboaphyton Arnold, 1937
?*Gilboaphyton goldringiae* Arnold 1985 Feng Shaonan, p.41
Leptophloeum Dawson, 1861 emend. Li, Dou et Sun, 1986
Leptophloeum rhombicum Dawson 1985 Feng Shaonan, p.41
Rhacophyton Crépin, 1875
?*Rhacophyton ceratangium* Anderws et Phillips 1985 Feng Shaonan, p.41

Hunan

1. Pingshan Section, Changsha City: Yunlugong Formation
Leptophloeum Dawson, 1861 emend. Li, Dou et Sun, 1986
Leptophloeum rhombicum Dawson 1982 Cai et Li, p. 115
Tenuisa Wang, 2007
Tenuisa frasniana Wang, 2007 2007 Wang Deming, figs.3-5

2. Yuelushan Section, Changsha City: Yunlugong Formation
Hamatophyton Gu et Zhi, 1974 emend. Li, Cai and Wang, 1995
Hamatophyton verticillatum Gu et Zhi, 1974 emend. Li, Cai et Wang, 1995 1982 Cai et Li, p. 115
Lepidodendropsis Lutz, 1933
Lepidodendropsis sp. 1982 Cai et Li, p. 115
Platyphyllum Dawson, 1888
Platyphyllum ginkgophylloides (Lee) Gu et Zhi 1974 Gu et Zhi, pl. 126, 1-2; 1982 Cheng Lizhu, pl.333, 9; 1982 Cai et Li, p. 115
Platyphyllum cf. *williamsonii* (Nathorst) Høeg 1974 Gu et Zhi, pl. 126, 3; 1982 Cheng Lizhu, pl.333, 3; 1982 Cai et Li, p. 115

3. Taiqingshan Section, Li County: Huangjiadeng Formation
Platyphyllum Dawson, 1888
Platyphyllum cf. *williamsonii* (Nathorst) Høeg 1982 Cheng Lizhu, pl.333, 3

Jiangxi

Taihe, Wan'an, Quannan, Xunwu: Zhongpeng Formation
Cyclostigma Haughton ex Heer, 1871
Cyclostigma kiltorkense 1978 Zhou Dianchao et Zhu Zhenggang, p. 185
Leptophloeum Dawson, 1861 emend. Li, Dou et Sun, 1986
Leptophloeum rhombicum Dawson 1978 Zhou Dianchao et Zhu Zhenggang, p. 185
Leptophloeum suzhouense 1978 Zhou Dianchao et Zhu Zhenggang, p. 185
Platyphyllum Dawson, 1888
Platyphyllum cf. *williamsonii* (Nathorst) Høeg 1978 Zhou Dianchao et Zhu Zhenggang, p. 185
Sphenopteridium Schimper, 1874
Sphenopteridium sp. 1978 Zhou Dianchao et Zhu Zhenggang, p. 185

Sichuan

1. Shangsi Village, Guangyuan City: Shawozi Formation

Archaeocalamites Stur, 1875

Archaeocalamites? *longiternodus* Li et Wang, 1982 1982 Li Xingxue et Wang, pl.1, 1-4

Archaeocalamites? sp. 1982 Li et Wang, pl.1, 5

Aspidiaria Presl, 1838

Aspidiaria sp. 1982 Li et Wang, pl.1, 10

Cyclostigma Haughton ex Heer, 1871

Cyclostigma kiltorkense 2009 Zhang et Gong, fig. 4, 2

Lepidodendropsis Lutz, 1933

Lepidodendropsis hirmeri Lutz 2009 Zhang et Gong, fig. 4, 1

Lepidodendropsis theodori (Zalessky) Jongmans 1982 Li Xingxue et Wang, pl.2, 3-4

Lepidodendropsis cf. *theodori* (Zalessky) Jongmans 1982 Li Xingxue et Wang, pl.2, 5

Lepidodendropsis? sp. 1982 Li Xingxue et Wang, pl.1, 9

Lepidosigillaria Kräusel et Weyland, 1949

Lepidosigillaria? *sichuanensis* 1982 1982 Li Xingxue et Wang, pl.2, 1-2

Leptophloeum Dawson, 1861 emend. Li, Dou et Sun, 1986

Leptophloeum rhombicum Dawson? 1982 Li Xingxue et Wang, pl.2, 7

Syringodendron Sternberg, 1820

Syringodendron sp.2 1982 Li Xingxue et Wang, pl.1, 8

2. Mao'ertang Section, Guangyuan City: Shawozi Formation

Syringodendron Sternberg, 1820

Syringodendron sp.1 1982 Li Xingxue et Wang, pl.1, 6-7

3. Yangquan Section, Zhuyuan Town, Guangyuan City: Shawozi Formation

Knorria Sternberg, 1825

Knorria sp. 1982 Li Xingxue et Wang, pl.2, 8

Knorria? sp. 1982 Li Xingxue et Wang, pl.1, 11

Late Famennian

Guangdong

1. Hashan, Muzhou Town, Xinhui County, Jiangmen City: Dawushi Formation

Archaeopteris (Dawson) Stur, 1875

Archaeopteris roemeriana (Göppert) Lesquereux 1977 Feng et al., pls. 239, 2; 253, 5; 1987 Cai et al., pl.1, 1-4a; pl.2, 1-2; 1995 Cai Chongyang et Li Xingxue, pl. VII, 1, 2

Carpolithus Brongniart, 1822

Carpolithus sp. 1987 Cai et al., pl.2, 5

Lepidostrobophyllum Hirmer, 1927

Lepidostrobophyllum sp. 1987 Cai et al., pl.2, 3, 3a

Sphenopteris (Brongniart) Sternberg, 1825

Sphenopteris sp. 1987 Cai et al., pl.2, 4, 4a

Stigmaria Brongniart, 1822

Stigmaria rugulosa Gothan 1987 Cai et al., pl.3, 5

Sublepidodendron (Nathorst) Hirmer, 1927 emend. Wang Q, Hao, Wang DM, Wang Y et Thomas Denk, 2003

Sublepidodendron grabaui (Sze) Wang et Xu, 2005 1987 Cai et al., pl.3, 3-4

Sublepidodendron cf. *grabaui* (Sze) Wang et Xu, 2005 1987 Cai et al., pl.3, 2

2. Dashacun Section, Guangzhou City: Dawushi Formation

Cyclostigma Haughton ex Heer, 1871

Cyclostigma kiltorkense Haughton ex Heer, 1871 1965 Fang Ruilian, pl. I, 9

Leptophloeum Dawson, 1861 emend. Li, Dou et Sun, 1986

Leptophloeum rhombicum Dawson 1965 Fang Ruilian, pl. I, 8

Sublepidodendron (Nathorst) Hirmer, 1927 emend. Wang Q, Hao, Wang DM, Wang Y et Thomas Denk, 2003

Sublepidodendron mirabile (Nathorst) Hirmer, 1927 1965 Fang Ruilian, pl. I, 10

Sublepidodendron cf. *mirabile* (Nathorst) Hirmer, 1927 1965 Fang Ruilian, pl. II, 11-14

3. Longweigang Section, Guangzhou City: Dawushi Formation

*Leptophloeum*Dawson, 1861 emend. Li, Dou et Sun, 1986

Leptophloeum rhombicum Dawson 1965 Fang Ruilian, pl. I, 8

4. Shashuigang Section, Guangzhou City: Dawushi Formation

Lepidodendron Sternberg, 1820

Lepidodendron sp. 1965 Fang Ruilian, pl. II, 15-17

Lepidostrobus Brongniart, 1828 emend. Brack-Hanes and Thomas, 1983

Lepidostrobus sp. 1965 Fang Ruilian, pl. III, 18-23

Sphenopteris (Brongniart) Sternberg, 1825

Sphenopteris sp. 1965 Fang Ruilian, pl. IV, 26-28

Shashuigang Section, Guangzhou City: Shashuigang Formation

Archaeocalamites Stur, 1875

Archaeocalamites sp. 1993 Wu Qijun et Dong Lingling, p. 112; 2001 Jin Jianhua et Wu Qijun, p. 167

Archaeopteris (Dawson) Stur, 1875

Archaeopteris sp. 2000 Jin Jianhua et al., p. 116

Cyclostigma Haughton ex Heer, 1871

Cyclostigma kiltorkense Haughton 1993 Wu Qijun et Dong Lingling, p. 112; 2000 Jin Jianhua et al., p. 116

Eolepidodendron Wu et Zhao, 1981

Eolepidodendron wusihense (Sze) Wu et Zhao, 1981 1993 Wu Qijun et Dong Lingling, p. 112; 2000 Jin Jianhua et al., p. 116

Hamatophyton Gu et Zhi, 1974 emend. Li, Cai et Wang, 1995

Hamatophyton verticillatum Gu et Zhi, 1974 emend. Li, Cai and Wang, 1995 1993 Wu Qijun et Dong Lingling, p. 112; 2000 Jin Jianhua et al., p. 116

Lepidodendropsis Lutz, 1933
Lepidodendropsis hirmeri Lutz 2000 Jin Jianhua et al., p. 116
Lepidodendropsis sp. 2000 Jin Jianhua et al., p. 116
Leptophloeum Dawson, 1861 emend.Li, Dou et Sun, 1986
Leptophloeum rhombicum Dawson 1993 Wu Qijun et Dong Lingling, p. 112; 2000 Jin Jianhua et al., p. 116
Sphenophyllum Brongniart, 1828
Sphenophyllum guangzhouensis 1993 Wu Qijun et Dong Lingling, p. 112; 2000 Jin Jianhua et al., p. 116
Sphenophyllum pseudotenerrimum (Sze) Li, 1984 1993 Wu Qijun et Dong Lingling, p. 112; 2000 Jin Jianhua et al., p. 116
Stigmaria Brongniart, 1822
Stigmaria ficoides (Sternberg) Brongniart 1993 Wu Qijun et Dong Lingling, p. 112; 2000 Jin Jianhua et al., p. 116
Sublepidodendron (Nathorst) Hirmer, 1927 emend. Wang Q, Hao, Wang DM, Wang Y et Thomas Denk, 2003
Sublepidodendron grabaui (Sze) Wang Y et Xu, 2005 1993 Wu Qijun et Dong Lingling, p. 112; 2000 Jin Jianhua et al., p. 116
Sublepidodendron mirabile (Nathorst) Hirmer, 1927 1993 Wu Qijun et Dong Lingling, p. 112; 2000 Jin Jianhua et al., p. 116
Sublepidodendron songziense Chen ex Wang Q, Hao, Wang DM, Wang Y, et Thomas Denk. 1993 Wu Qijun et Dong Lingling, p. 112
Sublepidodendron sp. 1 1993 Wu Qijun et Dong Lingling, p. 112; 2000 Jin Jianhua et al., p. 116

5. Chushanhu Section, Guangzhou City: Dawushi Formation

Lepidophyllum Brongniart, 1828
Lepidophyllum? sp. 1965 Fang Ruilian, pl. III, 24-25

6. Niugang Section, Guangzhou City: Dawushi Formation

Sphenopteris (Brongniart) Sternberg, 1825
Sphenopteris sp. 1965 Fang Ruilian, pl. IV, 26-28

7. Shangmiaobei, Wujiang District, Shaoguan City: Maozifeng Formation (late Famennian)

Hamatophyton Gu et Zhi, 1974 emend. Li, Cai and Wang, 1995
Hamatophyton sp. 1990 Zhao Ruxuan et Qin Guorong, tab. 2
Eolepidodendron Wu et Zhao, 1981
Eolepidodendron wusihense (Sze) Wu et Zhao, 1981 1990 Zhao Ruxuan et Qin Guorong, tab. 2
Lepidodendropsis Lutz, 1933
Lepidodendropsis hirmeri 1990 Zhao Ruxuan et Qin Guorong, tab. 2
Leptophloeum Dawson, 1861 emend. Li, Dou et Sun, 1986
Leptophloeum rhombicum Dawson 1977 Feng et al., pl.230, 15
Sublepidodendron (Nathorst) Hirmer, 1927 emend. Wang Q, Hao, Wang DM, Wang Y et Thomas Denk, 2003
Sublepidodendron mirabile (Nathorst) Hirmer, 1927 1990 Zhao Ruxuan et Qin Guorong, tab. 2

8. Shiliting, Zhenjiang District, Shaoguan City: Maozifeng Formation (late Famennian)

Hamatophyton Gu et Zhi, 1974 emend. Li, Cai and Wang, 1995
Hamatophyton sp. 1990 Zhao Ruxuan et Qin Guorong, tab. 2
Sublepidodendron (Nathorst) Hirmer, 1927 emend. Wang Q, Hao, Wang DM, Wang Y et Thomas Denk, 2003

Sublepidodendron sp. 1990 Zhao Ruxuan et Qin Guorong, tab. 2

9. Shengpinglu, Zhenjiang District, Shaoguan City: Maozifeng Formation (late Famennian)

Leptophloeum Dawson, 1861 emend. Li, Dou et Sun, 1986

Leptophloeum rhombicum Dawson 1977 Feng et al., pl.230, 15

Sublepidodendron (Nathorst) Hirmer, 1927 emend. Wang Q, Hao, Wang DM, Wang Y et Thomas Denk, 2003

Sublepidodendron sp. 1990 Zhao Ruxuan et Qin Guorong, tab. 2

10. Shijiaoju, Shaoguan City: Maozifeng Formation (late Famennian)

Leptophloeum Dawson, 1861 emend. Li, Dou et Sun, 1986

Leptophloeum rhombicum Dawson 1977 Feng et al., pl.230, 15

11. Chishijing, Renhua County, Shaoguan City: Maozifeng Formation (Tournaisian)

Leptophloeum Dawson, 1861 emend. Li, Dou et Sun, 1986

Leptophloeum rhombicum Dawson 1990 Zhao Ruxuan et Qin Guorong, tab. 2

Sublepidodendron (Nathorst) Hirmer, 1927 emend. Wang Q, Hao, Wang DM, Wang Y et Thomas Denk, 2003

Sublepidodendron sp. 1990 Zhao Ruxuan et Qin Guorong, tab. 2

12. Fankou, Renhua County, Shaoguan City: Maozifeng Formation (Tournaisian)

Leptophloeum Dawson, 1861 emend. Li, Dou et Sun, 1986

Leptophloeum rhombicum Dawson 1990 Zhao Ruxuan et Qin Guorong, tab. 2

Protolepidodendron Krejči, 1880 ex Gothan, 1921

Protolepidodendron? sp. 1990 Zhao Ruxuan et Qin Guorong, tab. 2

13. Xianrenmiao, Qujiang District, Shaoguan City: Maozifeng Formation (late Famennian)

Only plant fragments 1982 Cai Chongyang et Li Xingxue

14. Huangshaping, Qujiang District, Shaoguan City: Maozifeng Formation (late Famennian)

Only plant fragments 1982 Cai Chongyang et Li Xingxue

15. Bianshan, Ruyuan County, Shaoguan City: Maozifeng Formation (late Famennian)

Only plant fragments 1982 Cai Chongyang et Li Xingxue

Hunan

1. Wushidu Section, Tiaoma Town, Changsha County: Xikuangshan Formation

Lepidodendropsis Lutz, 1933

Lepidodendropsis hirmeri Lutz 1982 Cheng Lizhu, pl.324, 4

2. Tongmu Section, Tiaoma Town, Changsha County: Xikuangshan Formation

Lepidodendropsis Lutz, 1933

Lepidodendropsis hirmeri Lutz 1982 Cheng Lizhu, pl.324, 4

3. Chahuamiao, Lianyuan County: Xikuangshan Formation

Lepidodendropsis Lutz, 1933

Lepidodendropsis hirmeri Lutz 1982 Cheng Lizhu, pl.324, 4

4. Xiaduanchong, Liling County: Xikuangshan Formation

Sublepidodendron (Nathorst) Hirmer, 1927 emend. Wang Q, Hao, Wang DM, Wang Y et Thomas Denk, 2003

Sublepidodendron sp. 1982 Cheng Lizhu, pl.325, 1, 1a

5. Shuangjiangqiao, Shaodong County: Xikuangshan Formation

Sublepidodendron (Nathorst) Hirmer, 1927 emend. Wang Q, Hao, Wang DM, Wang Y et Thomas Denk, 2003

Sublepidodendron grabaui (Sze) Wang et Xu, 2005 1982 Cheng Lizhu, pl.327, 2, 3

6. Niu'ewan, Yiyang County: Xikuangshan Formation

Hamatophyton Gu et Zhi 1974 emend. Li, Cai and Wang, 1995

Hamatophyton verticillatum Gu et Zhi, 1974 emend. Li, Cai and Wang, 1995 1982 Cheng Lizhu, pl.327, 7; pl.328, 4; 1995 Li Xingxue et al., pls. I, 3; III, 2-4, 6-10, 12; VI-VIII

7. Nantang, Zhuzhou County: Xikuangshan Formation

Hamatophyton Gu et Zhi, 1974 emend. Li, Cai and Wang, 1995

Hamatophyton verticillatum Gu et Zhi, 1974 emend. Li, Cai and Wang, 1995 1982 Cheng Lizhu, pl.327, 7; pl.328, 4

8. Taipingchong, Matian District, Changsha County: Xikuangshan Formation

Lepidodendron Sternberg, 1820

Lepidodendron sp. 1963 Ao Zhenkuan, p. 610

Lepidophyllum Brongniart, 1828

Lepidophyllum sp. 1963 Ao Zhenkuan, p. 610

Sphenophyllum Brongniart, 1828

Sphenophyllum? *changshaense* Ao 1963 Ao Zhenkuan, fig. 1; 1982 Cheng Lizhu, pl.326, 2

Stigmaria Brongniart, 1822

Stigmaria ficoides (Sternberg) Brongniart 1963 Ao Zhenkuan, p. 610

Taeniocrada White, 1902

Taeniocrada? sp. 1963 Ao Zhenkuan, p. 610

9. Lianhuaqiao, Changsha County: Xikuangshan Formation

Platyphyllum Dawson, 1888

Platyphyllum subreiculatum Zhang, 1977 1982 Cheng Lizhu, pl.333, 4

10. Xikuangshan Section, Xinhua County: Xikuangshan Formation

Cyclostigma Haughton ex Heer, 1871

Cyclostigma kiltorkense Haughtom 1978 Zhao et al., p. 95

Hamatophyton Gu et Zhi, 1974 emend. Li, Cai and Wang, 1995

Hamatophyton verticillatum Gu et Zhi, 1974 emend. Li, Cai and Wang, 1995 1978 Zhao et al., p. 95; 1982 Cheng Lizhu, pl.327, 7; pl.328, 4; 1995 Cai Chongyang et Li Xingxue, pl. VII, 5

Knorria Sternberg, 1825

Knorria sp. 1978 Zhao et al., p. 95

Lepidodendropsis Lutz, 1933

Lepidodendropsis sp. 1978 Zhao et al., p. 95

Lepidodendropsis hirmeri Lutz 1978 Zhao et al., p. 95

Sublepidodendron (Nathorst) Hirmer, 1927 emend. Wang Q, Hao, Wang DM, Wang Y et Thomas Denk, 2003

Sublepidodendron grabaui (Sze) Wang et Xu, 2005 1978 Zhao et al., p. 95

Sublepidodendron mirabile (Nathorst) Hirmer, 1927 1978 Zhao et al., p. 95

11. Pingshan Section, Changsha City

Lepidodendropsis Lutz, 1933

Lepidodendropsis sp. 1982 Cai et Li, p. 115

Platyphyllum Dawson, 1888

Platyphyllum sp. 1982 Cai et Li, p. 115

Sublepidodendron (Nathorst) Hirmer, 1927 emend. Wang Q, Hao, Wang DM, Wang Y et Thomas Denk, 2003

Sublepidodendron sp. 1982 Cai et Li, p. 115

12. Wujiafang Section, Taojiang County: Yuelushan Formation

*Hamatophyton*Gu et Zhi, 1974 emend. Li, Cai and Wang, 1995

Hamatophyton verticillatum Gu et Zhi, 1974 emend. Li, Cai et Wang, 1995 2006 Wang DM et al., figs. 1-7

13. Yungaisi, Changsha City: Menggong'ao Formation

Lepidodendropsis Lutz, 1933

Lepidodendropsis hirmeri Lutz 1982 Cheng Lizhu, pl.324, 4

14. Majiagou Section, Sansheng Commune, Shimen County: Xiejingsi Formation

Archaeosigillaria (Kidston) Lacey, 1962

Archaeosigillaria? sp. 1977 Feng Shaonan et al., pl.233, 7; 1975 Feng Shaonan et Meng Fansong, pl.I, 16-17

Calamostachys Schimper, 1869

Calamostachys hunanensis? Feng, 1977 1982 Cheng, pl.329, 8; 1977 Feng et al., pl.237, 8-9

Calamostachys sp. 1975 Feng Shaonan et Meng Fansong, pl. I, 18

Cyclostigma Haughton ex Heer, 1871

Cyclostigma hunanense Feng et Meng, 1975 1977 Feng Shaonan et al., pl.230, 5, 6; 1975 Feng Shaonan et Meng Fansong, pl.III, 8-12

Lepidodendropsis Lutz, 1933

Lepidodendropsis cyclostigmatoides Jongmans, Gothan et Darrah 1937 1982 Cheng Lizhu, pl.324, 9; 1977 Feng Shaonan et al., pl.230, 12-14; 1975 Feng Shaonan et Meng Fansong, pl.II, 12; pl.III, 3-7

Palaeostachya Weiss, 1876

Palaeostachya shimenensis Feng, 1977 1982 Cheng Lizhu, pl.328, 3; 1977 Feng Shaonan et al., pl.237, 10

Sublepidodendron (Nathorst) Hirmer, 1927 emend. Wang Q, Hao, Wang DM, Wang Y et Thomas Denk, 2003

Sublepidodendron grabaui (Sze) Wang et Xu, 2005 1982 Cheng, pl.327, 2, 3; 1977 Feng et al., pl.234, 12-13; 1975 Feng et Meng, pl.I, 12-14

Sublepidodendron mirabile (Nathorst) Hirmer, 1927 1975 Feng Shaonan et Meng Fansong, pls. I, 15; II, 5-7; 1977 Feng Shaonan et al., pl.231, 14; 1982 Cheng Lizhu, pl.324, 7-8

Sublepidodendron shimenense Feng et Meng, 1975 1982 Cheng Lizhu, pl.325, 4, 4a; 1977 Feng Shaonan et al., pl.231, 16; 1975 Feng Shaonan et Meng Fansong, pl.I, 7-8

15. Tianmenya Village, Shimen County: Tizikou Formation (Strunian)

Archaeosigillaria (Kidoston) Lacey, 1962

Archaeosigillaria? *vanuxemi* (Göppert) Kidston 1985 Feng Shaonan, p.41

Barinophyton White, 1905

Barinophyton citrulliformie? 1985 Feng Shaonan, p.41

Cyclostigma Haughton ex Heer, 1871

Cyclostigma kiltorkense Haughton ex Heer, 1871 1985 Feng Shaonan, p.41

Cyclostigma? *yiduense* Feng, 1984 1985 Feng Shaonan, p.41

Drepanophycus Göppert, 1852

Drepanophycus spinaeformis Göppert, 1852 1985 Feng Shaonan, p.41

Drepanophycus spinosus 1985 Feng Shaonan, p.41

Eolepidodendron Wu et Zhao, 1981

Eolepidodendron densata? Feng et Meng 1985 Feng Shaonan, p.41

Eolepidodendron timenense? 1985 Feng Shaonan, p.41

Eolepidodendron wusihense (Sze) Wu et Zhao 1981 1984 Feng Shaonan, pl.49, 7; 1985 Feng Shaonan, p.41

Eolepidodendron volidum? Feng et Meng 1985 Feng Shaonan, p.41

Hamatophyton Gu et Zhi, 1974 emend. Li, Cai et Wang, 1995

Hamatophyton verticillatum Gu et Zhi, 1974 emend. Li, Cai et Wang, 1995 1985 Feng Shaonan, p.41

Lepidostrobophyllum Hirmer, 1927

Lepidostrobophyllum xiphidium (Gothan et Sze) Gu et Zhi, 1974 1985 Feng Shaonan, p.41

Lepidodendropsis Lutz, 1933

Lepidodendropsis hirmeri Lutz 1984 Feng Shaonan, pl.49, 11; 1985 Feng Shaonan, p.41

Lepidodendropsis theodori Jongmans 1985 Feng Shaonan, p.41

Leptophloeum Dawson emend. Li, Dou et Sun, 1986

Leptophloeum rhombicum Dawson 1985 Feng Shaonan, p.41

Platyphyllum Dawson, 1888

Platyphyllum cf. *williamsonii* (Nathorst) Höeg 1985 Feng Shaonan, p.41

Sphenopteris (Brongniart) Sternberg, 1825

Sphenopteris? *recurva* 1985 Feng Shaonan, p.41

Sublepidodendron (Nathorst) Hirmer, 1927 emend. Wang Q, Hao, Wang DM, Wang Y et Thomas Denk, 2003

Sublepidodendron grabaui (Sze) Wang et Xu 1984 Feng Shaonan, pl.49, 3-5, 9; 1985 Feng Shaonan, p.41

Sublepidodendron mirabile (Nathorst) Hirmer, 1927 1985 Feng Shaonan, p.41

Sublepidodendron cf. *mirabile* (Nathorst) Hirmer, 1927 1984 Feng Shaonan, pl.49, 2; 1985 Feng Shaonan, p.41

Sublepidodendron songziense Chen ex Wang Q, Hao, Wang DM, Wang Y, et Thomas Denk. 1985 Feng Shaonan, p.41

Sublepidodendron sp. 1985 Feng Shaonan, p.42
Yichangophyton Feng, 1984
Yichangophyton? *guanzuangense* (Feng et Meng) Feng, 1984 1985 Feng Shaonan, p.41

Jiangxi

1. **Near Pingxiang City: Xikuangshan Formation**

Hamatophyton Gu et Zhi, 1974 emend. Li, Cai et Wang, 1995
Hamatophyton verticillatum Gu et Zhi, 1974 emend. Li, Cai et Wang, 1995 1982 Cai et Li, p. 116
Lepidodendropsis Lutz, 1933
Lepidodendropsis hirmeri Lutz 1982 Cai et Li, p. 116
Leptophloeum Dawson, 1861 emend. Li, Dou et Sun, 1986
Leptophloeum rhombicum Dawson 1982 Cai et Li, p. 116
Sublepidodendron (Nathorst) Hirmer, 1927 emend. Wang Q, Hao, Wang DM, Wang Y et Thomas Denk, 2003
Sublepidodendron sp. 1982 Cai et Li, p. 116

2. **Yanghu Section, Yongxin City: Xikuangshan Formation**

Hamatophyton Gu et Zhi, 1974 emend. Li, Cai et Wang, 1995
Hamatophyton verticillatum Gu et Zhi, 1974 emend. Li, Cai et Wang, 1995 1982 Cai et Li, p. 116
Lepidodendropsis Lutz, 1933
Lepidodendropsis hirmeri Lutz 1982 Cai et Li, p. 116
Leptophloeum Dawson, 1861 emend. Li, Dou et Sun, 1986
Leptophloeum rhombicum Dawson 1982 Cai et Li, p. 116
Sublepidodendron (Nathorst) Hirmer, 1927 emend. Wang Q, Hao, Wang DM, Wang Y et Thomas Denk, 2003
Sublepidodendron sp. 1982 Cai et Li, p. 116

3. **Xinfeng: Sanmentan Formation**

Leptophloeum Dawson 1861 emend. Li, Dou et Sun, 1986
Leptophloeum rhombicum Dawson 1978 Zhou Dianchao et Zhu Zhenggang, p. 185
Sublepidodendron (Nathorst) Hirmer, 1927 emend. Wang Q, Hao, Wang DM, Wang Y et Thomas Denk, 2003
Sublepidodendron grabaui (Sze) Wang et Xu, 2005 1978 Zhou Dianchao et Zhu Zhenggang, p. 185
Sublepidodendron mirabile (Nathorst) Hirmer, 1927 1978 Zhou Dianchao et Zhu Zhenggang, p. 185

4. **Northwestern suburb of Chongyi County to Banqiaokeng, Chongyi County: Chongyi Formation (late Famennian)**

Cyclostigma Haughton ex Heer, 1871
Cyclostigma chongyiense Chang, 1978 1978 Zhang Zhongying, pl. I, 1-4
Hamatophyton Gu et Zhi, 1974 emend. Li, Cai et Wang, 1995
Hamatophyton verticillatum Gu et Zhi, 1974 emend. Li, Cai et Wang, 19951978 Zhang Zhongying, p. 73
Lepidodendron Sternberg, 1820
Lepidodendron? sp. 1978 Zhang Zhongying, p. 73

Lepidostrobophyllum Hirmer, 1927
Lepidostrobophyllum xiphidium (Gothan et Sze) Gu et Zhi, 1974 1978 Zhang Zhongying, p. 73
Lepidostrobophyllum sp. 1978 Zhang Zhongying, p. 73
Lepidostrobus Brongniart, 1828 emend. Brack-Hanes and Thomas, 1983
Lepidostrobus sp. 1978 Zhang Zhongying, p. 72
Leptophloeum Dawson, 1861 emend. Li, Dou et Sun, 1986
Leptophloeum rhombicum Dawson 1978 Zhang Zhongying, p. 72
Platyphyllum Dawson, 1888
Platyphyllum sp. 1978 Zhang Zhongying, p. 73
Stigmaria Brongniart, 1822
Stigmaria rugulosa Gothan 1978 Zhang Zhongying, p. 72
Stigmaria cf. *radiato-punctata* Gothan et Sze 1978 Zhang Zhongying, p. 72
Sublepidodendron (Nathorst) Hirmer, 1927 emend. Wang Q, Hao, Wang DM, Wang Y et Thomas Denk, 2003
Sublepidodendron? *grabaui* (Sze) Wang et Xu 1978 Zhang Zhongying, p. 73
Sublepidodendron mirabile (Nathorst) Hirmer, 1927 1978 Zhang Zhongying, p. 72
Sublepidodendron sp. 1978 Zhang Zhongying, p. 73
Sublepidodendron? sp. 1978 Zhang Zhongying, p. 73
Sublepidodendron spp. 1978 Zhang Zhongying, p. 73

Anhui

1. Dongzhi Section, Chizhou City: Wutung Formation
Archaeopteris (Dawson) Stur, 1875
Archaeopteris halliana 2009 Guo Yun et Wang Deming, pl. I-V

2. Banshanli Section, Tongling County, Tongling City: Wutung Formation
Archaeopteris (Dawson) Stur, 1875
Archaeopteris sp. 1984 Li Hanmin et al., p. 153
Eolepidodendron Wu et Zhao, 1981
Eolepidodendron wusihense (Sze) Wu et Zhao, 1981 1984 Li Hanmin et al., p. 153, 154
Sublepidodendron (Nathorst) Hirmer, 1927 emend. Wang Q, Hao, Wang DM, Wang Y et Thomas Denk, 2003
Sublepidodendron mirabile (Nathorst) Hirmer 1984 Li Hanmin et al., p. 153, 154

3. Dachucun Section, Xinqiao Town, Tongling County, Tongling City: Wutung Formation
Eolepidodendron Wu et Zhao, 1981
Eolepidodendron wusihense (Sze) Wu et Zhao, 1981 1984 Li Hanmin et al., p. 154
Hamatophyton Gu et Zhi 1974 emend. Li, Cai et Wang, 1995
Hamatophyton verticillatum Gu et Zhi, 1974 emend. Li, Cai et Wang, 1995 1984 Li Hanmin et al., p. 154
Sublepidodendron (Nathorst) Hirmer, 1927 emend. Wang Q, Hao, Wang DM, Wang Y et Thomas Denk, 2003
Sublepidodendron mirabile (Nathorst) Hirmer 1984 Li Hanmin et al., p. 154
Sublepidodendron taihuensis Sze 1984 Li Hanmin et al., p. 154

4. Yeshanchong Section, Zhongming, Tongling County, Tongling City: Wutung Formation

Archaeopteris (Dawson) Stur, 1875

Archaeopteris sp. 1984 Li Hanmin et al., p. 154

Eolepidodendron Wu et Zhao, 1981

Eolepidodendron wusihense (Sze) Wu et Zhao, 1981 1984 Li Hanmin et al., p. 155

Sphenophyllum Brongniart, 1828

Sphenophyllum pseudotenerrimum (Sze) Li, 1984 1984 Li Hanmin et al., pls. III, 5-6; II, 11

Sublepidodendron (Nathorst) Hirmer, 1927 emend. Wang Q, Hao, Wang DM, Wang Y et Thomas Denk, 2003

Sublepidodendron mirabile (Nathorst) Hirmer 1984 Li Hanmin et al., p. 155

5. Limaoding Section, Jinlang Town, Tongling County, Tongling City: Wutung Formation

Archaeopteris (Dawson) Stur, 1875

Archaeopteris tonglingiana Li et al., 1984 1984 Li Hanmin et al., pl. I, 1-4

Archaeopteris zhongmingiana Li et al., 1984 1984 Li Hanmin et al., pl. II, 17

Archaeopteris mutatoformis Wu 1984 Li Hanmin et al., pl. II, 9-10

Archaeopteris cf. *sphenophyllifolia* Lesquereux 1984 Li Hanmin et al., p. 156

Eolepidodendron Wu et Zhao, 1981

Eolepidodendron wusihense (Sze) Wu et Zhao, 1981 1984 Li Hanmin et al., p. 156

Hamatophyton Gu et Zhi, 1974 emend. Li, Cai and Wang, 1995

Hamatophyton verticillatum Gu et Zhi, 1974 emend. Li, Cai and Wang, 1995 1984 Li Hanmin et al., pl. III, 7-10

Lepidodendropsis Lutz, 1933

Lepidodendropsis hirmeri 1984 Li Hanmin et al., p. 156

Protopteridium minutum Halle [possibly *Eocladoxylon minutum* (Halle) Koidzumi emend. Berry et Wang, 2006] 1984 Li Hanmin et al., pl. 3, 1-4

Sphenophyllum Brongniart, 1828

Sphenophyllum lungtanense Gothan et Sze emend. Gu et Zhi 1984 Li Hanmin et al., pl. I, 6-7

Stigmaria Brongniart, 1822

Stigmaria ficoides (Sternberg) Brongniart 1984 Li Hanmin et al., p. 156

Sublepidodendron (Nathorst) Hirmer, 1927 emend. Wang Q, Hao, Wang DM, Wang Y et Thomas Denk, 2003

Sublepidodendron mirabile (Nathorst) Hirmer 1984 Li Hanmin et al., p. 156

Sublepidodendron grabaui (Sze) Wang et Xu 1984 Li Hanmin et al., p. 156

6. Qishan Section, Chaohu City: Wutung Formation

Hamatophyton Gu et Zhi, 1974 emend. Li, Cai et Wang, 1995

Hamatophyton verticillatum Gu et Zhi, 1974 emend. Li, Cai et Wang, 1995 1984 Li Hanmin et Lan Shanxian, p.223; 2009 Wang Deming et Guo Yun, pl.I-III

Lepidodendropsis Lutz, 1933

Lepidodendropsis hirmeri Lutz 1984 Li Hanmin et Lan Shanxian, p.223

Sphenophyllum Brongniart, 1828

Sphenophyllum? *changshaense* Ao 1984 Li Hanmin et Lan Shanxian, pl.I, 3-6; 1995 Cai Chongyang et Wang Yi, p. 43

Stigmaria Brongniart, 1822

Stigmaria ficoides (Sternberg) Brongniart 1984 Li Hanmin et Lan Shanxian, p.223

Sublepidodendron (Nathorst) Hirmer, 1927 emend. Wang Q, Hao, Wang DM, Wang Y et Thomas Denk, 2003

Sublepidodendron songziense Chen ex Wang Q, Hao, Wang DM, Wang Y, et Thomas Denk. 1984 Li Hanmin et Lan Shanxian, pl.I, 1, 1a, 1b, 2, 2a, 7, 7a, 8

Sublepidodendron grabaui (Sze) Wang et Xu, 2005 1984 Li Hanmin et Lan Shanxian, p.223

Sublepidodendron mirabile (Northorst) Hirmer, 1927 1984 Li Hanmin et Lan Shanxian, p.223

7. Shizikou Section, Chaohu City: Wutung Formation

Archaeopteris (Dawson) Stur, 1875

Archaeopteris sp. 1994 Cai Chongyang et Wu Xiuyuan, p. 75

Archaeopteris macillenta Lesquereux 2001 Wang, p. 440; 2003 Wang Qi et al., p. 280

Bothrodendron Lindley et Hutton, 1833

Bothrodendron (Cyclostigma) anhuiense Cai et Wu, 1994 1994 Cai Chongyang et Wu Xiuyuan, pl.I, 1, 1a, 2, 2a; pl.II, 1

Bothrodendron (Cyclostigma) cf. *kiltorkense* Haughton 1994 Cai Chongyang et Wu Xiuyuan, pl.I, 3, 3a; 2001 Wang, p. 440

Bothrodendron (Cyclostigma) sp.1 1994 Cai Chongyang et Wu Xiuyuan, pl.II, 2, 2a, 2b

Bothrodendron (Cyclostigma) sp.2 1994 Cai Chongyang et Wu Xiuyuan, pl.II, 3, 3a

Cyclostigma Haughton ex Heer, 1870

Cyclostigma kiltorkense Haughton ex Heer 18712003 Wang Qi et al., p. 279; 2012 Wang Yi et al., p. 22.

Hamatophyton Gu et Zhi, 1974 emend. Li, Cai et Wang, 1995

Hamatophyton verticillatum Gu et Zhi, 1974 emend. Li, Cai and Wang, 1995 1994 Cai Chongyang et Wu Xiuyuan, p. 75; 1995 Li Xingxue et al., pl. III, 11; 2001 Wang, p. 440

Lepidodendropsis Lutz, 1933

Lepidodendropsis hirmeri Lutz, 1933 2001 Wang, p. 440

Lepidodendropsis cf. *scobiniformis* (Meek) Read 1994 Cai Chongyang et Wu Xiuyuan, p. 76

Leptophloeum Dawson, 1861 emend. Li, Dou et Sun, 1986

Leptophloeum rhombicum Dawson 2001 Wang Yi, p. 439; 2003 Wang Qi et al., p. 279

Minostrobus Wang Yi, 2001 emend Wang Yi, Xu Honghe et Wang Qi

Minostrobus chaohuensis WangYi emend. Meng Meiceng et al., 2013 2001 Wang Yi, pls.3-41; 2012 Wang Yi et al., figs. 1-3; 2013 Meng Meicen et al., pls. 1-10, in press

Sphenophyllum Brongniart, 1828

Sphenophyllum lungtanense Gothan et Sze emend. Gu et Zhi 1994 Cai Chongyang et Wu Xiuyuan, p. 75; 2001 Wang, p. 440; 2008 Wang DM et al., figs. 1-3

Stigmaria Brongniart, 1822

Stigmaria radiato-punctata Gothan and Sze 1994 Cai Chongyang et Wu Xiuyuan, p. 76

Sublepidodendron (Nathorst) Hirmer, 1927 emend. Wang Q, Hao, Wang DM, Wang Y et Thomas Denk, 2003

Sublepidodendron grabaui (Sze) Wang et Xu, 2005 1994 Cai Chongyang et Wu Xiuyuan, p. 76; 2001 Wang, p. 440; 2005 Wang Yi et Xu, figs.2-57

Sublepidodendron mirabile (Nathorst) Hirmer 1927 1994 Cai Chongyang et Wu Xiuyuan, p. 75; 2001 Wang, p. 439

Sublepidodendron songziense Chen ex Wang Q, Hao, Wang DM, Wang Y, et Thomas Denk. 2003 Wang Qi et al., pls. I-VI; figs. 4-6

Jiangsu

1. Dachaoshan, Dingshu Town, Yixing City: Wutung Formation

Lepidodendron Sternberg, 1820

Lepidodendron hirmeri Lutz. 1979 Wu Chongzhang et al., p. 53

Sphenophyllum Brongniart, 1828

Sphenophyllum megalofolium Wu, 1979 1979 Wu Chongzhang et al., pl.1-5; fig.a, b

Sphenophyllum pseudotenerrimum Sze 1979 Wu Chongzhang et al., p. 53

Sublepidodendron (Nathorst) Hirmer, 1927 emend. Wang Q, Hao, Wang DM, Wang Y et Thomas Denk, 2003

Sublepidodendron grabaui (Sze) Wang et Xu, 20051979 Wu Chongzhang et al., p. 53

Sublepidodendron mirabile (Nathorst) Hirmer, 1927 1979 Wu Chongzhang et al., p. 53

2. Dachaoshan, Dingshu Town, Yixing City: Middle Wutung Formation

Sublepidodendron (Nathorst) Hirmer, 1927 emend. Wang Q, Hao, Wang DM, Wang Y et Thomas Denk, 2003

Sublepidodendron cf. *xinjiangense* Sun 1996 Du Meili, pl. I, 1-9

3. Nanshan, Dingshu Town, Yixing City: Wutung Formation

Hamatophyton Gu et Zhi, 1974 emend. Li, Cai et Wang, 1995

Hamatophyton verticillatum Gu et Zhi, 1974 emend. Li, Cai et Wang, 1995 1995 Li Xingxue et al., pls. I, 2, 4-7; II, 1-11; III, 1, 5, 13, 15; IV, 1-15

Eviostachya Stockmans, 1948

Eviostachya hoegii Stockmans emend. Wang, 1993 1993 Wang Yi, pls. I-IV; V, 1-6, 10-11; VI; 1995 Cai Chongyang et Li Xingxue, pl. VIII, 10

Leptophloeum Dawson, 1861 emend. Li, Dou et Sun, 1986

Leptophloeum rhombicum Dawson 2003 Wang Yi et Berry, p. 1297

Lepidodendropsis Lutz, 1933

Lepidodendropsis hirmeri Lutz 2003 Wang Yi et Berry, p. 1297

Monilistrobus Wang et Berry, 2003

Monilistrobus yixingensis Wang et Berry, 2003 2003 Wang Yi et Berry, pls.1-3; figs.2-5

Sphenophyllum Brongniart 1828

Sphenophyllum lungtanense Gothan et Sze emend. Gu et Zhi 2003 Wang Yi et Berry, p. 1297

Sphenopteris (Brongniart) Sternberg, 1825

Sphenopteris taihuense Sze 2003 Wang Yi et Berry, p. 1297

Stigmaria Brongniart, 1822

Stigmaria sp. 1988 Cai Chongyang et al., p. 182

Sublepidodendron (Nathorst) Hirmer, 1927 emend. Wang Q, Hao, Wang DM, Wang Y et Thomas Denk, 2003

Sublepidodendron grabaui (Sze) Wang et Xu, 2005 2005 Wang Yi et Xu, figs.2-57

Sublepidodendron mirabile (Nathorst) Hirmer, 1927 2003 Wang Yi et Berry, p. 1297

4. Qinhuangshan, Jiangyin City: Wutung Formation

Hamatophyton Gu et Zhi, 1974 emend. Li, Cai et Wang, 1995

Hamatophyton verticillatum Gu et Zhi, 1974 emend. Li, Cai et Wang, 1995 1974 Gu et Zhi, pl.19, 3-5; pl.20, 1-4; 1982 Li Hanmin et al., pl.137, 1-2; 1995 Li Xingxue et al., pls. VI, 16; V, 1-9

5. Xiaohuoshan quarry, Jilongshan, Wuxi City: Wutung Formation

Wuxia Berry, Wang et Cai, 2003

Wuxia bistrobilata Berry, Wang et Cai, 2003 2003 Berry C M et al., pl.2-6

6. Cishan, Nanjing City: Leigutai Member of Wutung Formation (late Famennian)

Lepidodendropsis Lutz, 1933

Lepidodendropsis hirmeri Lutz 1987 Li Hanmin et al., p. 118

Sphenopteris (Brongniart) Sternberg, 1825

Sphenopteris taihuensis Sze 1987 Li Hanmin et al., p. 118

Stigmaria Brongniart, 1822

Stigmaria ficoides (Sternberg) Brongniart 1987 Li Hanmin et al., p. 118

Sublepidodendron (Nathorst) Hirmer, 1927 emend. Wang Q, Hao, Wang DM, Wang Y et Thomas Denk, 2003

Sublepidodendron grabaui (Sze) Wang et Xu 1987 Li Hanmin et al., p. 118

7. Kongshan, Nanjing City: Wutung Formation

Archaeopteris (Dawson) Stur, 1875

Archaeopteris macilenta (Lesquereux) Carluccio, Hueber et Banks, 1966 1982 Wu Chongzhang et al., pl. I, 1-3; fig. 1

Archaeopteris sp. 1 1982 Wu Chongzhang et al., pl.I, 4, 5

Archaeopteris sp. 2 1982 Wu Chongzhang et al., pl.I, 6; fig.2

Eviostachya Stockmans, 1948

Eviostachya hoegii Stockmans emend. Wang 1993 1993 Wang Yi, pl. V, 8-10

Hamatophyton Gu et Zhi, 1974 emend. Li, Cai et Wang, 1995

Hamatophyton verticillatum Gu et Zhi, 1974 emend. Li, Cai et Wang, 1995 1988 Cai Chongyang et al., p. 178

Kongshania Wang, 2000

Kongshania synangioides Wang, 2000 2000 Wang Yi, pls.1-4

Lepidostrobus Brongniart, 1828 emend. Brack-Hanes and Thomas, 1983

Lepidostrobus sp. 1988 Cai Chongyang et al., p. 178

Leptophloeum Dawson, 1861 emend. Li, Dou et Sun, 1986

Leptophloeum rhombicum Dawson 1982 Wu Chongzhang et al., p. 292

Rhacophyton Crépin, 1875

Rhacophyton? sp. 1988 Cai Chongyang et al., p. 178

Sphenophyllum Brongniart, 1828

Sphenophyllum lungtanense Gothan et Sze emend. Gu et Zhi 1988 Cai Chongyang et al., p. 178

Sphenopteris (Brongniart) Sternberg, 1825

Sphenopteris sp. 1988 Cai Chongyang et al., p. 178

Sublepidodendron (Nathorst) Hirmer, 1927 emend. Wang Q, Hao, Wang DM, Wang Y et Thomas Denk, 2003

Sublepidodendron? *grabaui* (Sze) Wang et Xu 1982 Wu Chongzhang et al., p. 292

Sublepidodendron mirabile (Nathorst) Hirmer 1927 1982 Wu Chongzhang et al., p. 292

8. Fentou Village, Jiangning District, Nanjing City: Wutung Formation

Cyclostigma Haughton ex Heer, 1871

Cyclostigma kiltorkense Haughton ex Heer, 1871 1982 Li Hanmin et al., pl.131, 10-11

9. Ma'anshan Section, Shangfang Town, Jiangning District, Nanjing City: Lower–Mid Wutung Formation (late Famennian)

Archaeopteris (Dawson) Stur, 1875

Archaeopteris cf. *macilenta* (Lesquereux) Carluccio, Hueber et Banks, 1966 2005 Yang Songsheng, pl. I, 1-7; fig. 2.

Archaeopteris sp. 2005 Yang Songsheng, p. 80

Cordaites Unger, 1850

Cordaites sp. 2005 Yang Songsheng, p. 80

Cyclostigma Haughton ex Heer, 1871

Cyclostigma kiltorkense Haughton ex Heer, 1871 2005 Yang Songsheng, p. 80

Eolepidodendron Wu et Zhao, 1981

Eolepidodendron wusihense (Sze) Wu et Zhao, 1981 2005 Yang Songsheng, p. 80

Eolepidodendron Wu et Zhao, 1981

Eolepidodendron sp. 2005 Yang Songsheng, p. 80

Hamatophyton Gu et Zhi, 1974 emend. Li, Cai et Wang, 1995

Hamatophyton sp. 2005 Yang Songsheng, p. 80

Lepidodendropsis Lutz, 1933

Lepidodendropsis hirmeri Lutz 2005 Yang Songsheng, p. 80

Lepidodendron Sternberg, 1820

Lepidodendron sp. 2005 Yang Songsheng, p. 80

Lepidostrobus Brongniart, 1828 emend. Brack-Hanes and Thomas, 1983

Lepidostrobus aff. *ugulatus* 2005 Yang Songsheng, p. 80

Lepidostrobus sp. 2005 Yang Songsheng, p. 80

Sublepidodendron (Nathorst) Hirmer, 1927 emend. Wang Q, Hao, Wang DM, Wang Y et Thomas Denk, 2003

Sublepidodendron grabaui (Sze) Wang et Xu 2005 Yang Songsheng, p. 80

Sublepidodendron mirabile (Nathorst) Hirmer, 1927 2005 Yang Songsheng, p. 80

Sublepidodendron sp. 1 2005 Yang Songsheng, p. 80

Sublepidodendron sp. 2 2005 Yang Songsheng, p. 80

Sublepidodendron sp. 3 2005 Yang Songsheng, p. 80

10. Guanshan Section, Longtan Town, Nanjing City: Lower–Middle Leigutai Member of Wutung Formation

Archaeocalamites Stur, 1875

Asterocalamites aff. *scrobiculatus* (Schlotheim) Zeiller 1988 Cai Chongyang et al., p. 173

Hamatophyton Gu et Zhi, 1974 emend. Li, Cai et Wang, 1995

Hamatophyton verticillatum Gu et Zhi, 1974 emend. Li, Cai et Wang, 1995 1988 Cai Chongyang et al., p. 173

Rhacophyton Crépin, 1875

Rhacophyton? sp. 1988 Cai Chongyang et al., p. 173

Sphenopteridium Schimper, 1874

Sphenopteridium? sp. a 1988 Cai Chongyang et al., p. 173

Sphenopteridium? sp. b 1988 Cai Chongyang et al., p. 173

Sphenopteris (Brongniart) Sternberg, 1825

Sphenopteris cf. *taihuensis* 1988 Cai Chongyang et al., p. 173

Stigmaria Brongniart, 1822

Stigmaria sp. 1988 Cai Chongyang et al., p. 173, 174

Sublepidodendron (Nathorst) Hirmer, 1927 emend. Wang Q, Hao, Wang DM, Wang Y et Thomas Denk, 2003

"Sublepidodendron" grabaui (Sze) Wang et Xu 1988 Cai Chongyang et al., p. 173

Sublepidodendron sp. 1988 Cai Chongyang et al., p. 173

Guanshan Section, Longtan Town, Nanjing City: Guanshan Member of Wutung Formation

Sublepidodendron (Nathorst) Hirmer, 1927 emend. Wang Q, Hao, Wang DM, Wang Y et Thomas Denk, 2003

Sublepidodendron? sp. 1988 Cai Chongyang et al., p. 174

11. Leigutai Section, Longtan Town, Nanjing City: Middle Leigutai Member of Wutung Formation

Lepidodendropsis Lutz, 1933

Lepidodendropsis hirmeri 1988 Cai Chongyang et al., p. 175

Lepidodendropsis sp. 1988 Cai Chongyang et al., p. 175

Leptophloeum Dawson, 1861 emend. Li, Dou et Sun, 1986

Leptophloeum rhombicum Dawson 1988 Cai Chongyang et al., p. 175

Sublepidodendron (Nathorst) Hirmer, 1927 emend. Wang Q, Hao, Wang DM, Wang Y et Thomas Denk, 2003

"Sublepidodendron" grabaui (Sze) Wang et Xu 1988 Cai Chongyang et al., p. 175

Sublepidodendron mirabile (Nathorst) Hirmer, 1927 1988 Cai Chongyang et al., p. 175

Sublepidodendron? sp. 1988 Cai Chongyang et al., p. 175

12. Yixing: Middle Wutung Formation

Xihuphyllum Chen, 1988

Xihuphyllum megalofolium (Wu) Chen, 1988 1995 Cai Chongyang et Li Xingxue, pl. VI, 1-3

Hubei

1. Lan'ganya Section, Changyang County: Xiejingsi Fomation

Archaeopteris (Dawson) Stur, 1875

Archaeopteris sp. 2005 Xue et al., p. 520

Hubeiia Xue et al., 2005

Hubeiia dicrofollia Xue et al., 2005 2005 Xue et al., figs.2, 4-6

2. Near Changyang County: Xiejingsi Formation

Leptophloeum Dawson, 1861 emend. Li, Dou et Sun, 1986

Leptophloeum rhombicum Dawson 1977 Feng et al., pl.230, 15

Sublepidodendron (Nathorst) Hirmer, 1927 emend. Wang Q, Hao, Wang DM, Wang Y et Thomas Denk, 2003

Sublepidodendron mirabile (Nathorst) Hirmer 1927 1977 Feng Shaonan et al., pl.231, 14; 1984 Chen Gongxin, pl. 218, 7

3. Guanzhuang, Yichang City: Xiejingsi Formation

Cyclostigma Haughton ex Heer, 1871

Cyclostigma hunanense Feng et Meng 1984 Chen Gongxin, pl. 216, 8

Lepidodendropsis Lutz, 1933

Lepidodendropsis guanzhuangensis Feng et Meng 1984 Chen Gongxin, pl. 216, 3

Lepidostrobophyllum Hirmer, 1927

Lepidostrobophyllum xiphidium (Gothan et Sze) Gu et Zhi, 1974 1984 Chen Gongxin, pl. 218, 5

Sublepidodendron (Nathorst) Hirmer, 1927 emend. Wang Q, Hao, Wang DM, Wang Y et Thomas Denk, 2003

Sublepidodendron yichangense Feng et Meng 1984 Chen Gongxin, pl. 216, 7

Guanzhuang, Yichang City: Tizikou Fomation (Strunian)

Cyclostigma Haughton ex Heer, 1871

Cyclostigma kiltorkense Haughton 1984 Feng Shaonan, pl.47, 1-3

Lepidodendropsis Lutz, 1933

Lepidodendropsis theodori (Zalessky) Jongmans 1984 Feng Shaonan, pl.47, 7

Lepidostrobophyllum Hirmer, 1927

Lepidostrobophyllum xiphidium (Gothan et Sze) Gu et Zhi, 1974 1984 Feng Shaonan, pl.47, 14

Yichangophyton Feng, 1984

Yichangophyton? *guanzuangense* (Feng et Meng) Feng, 1984 1984 Feng Shaonan, pl.48, 5-6

4. Maohushang Section, Yidu County: Xiejingsi Formation

Lepidodendropsis Lutz, 1933

Lepidodendropsis hirmeri Lutz 1977 Feng Shaonan et al., pl.231, 8-9

Lepidodendropsis scobiniformis (Meek) Read 1977 Feng Shaonan et al., pl.231, 10-11

Rotafolia Wang DM, Hao et Wang Q, 2005

Rotafolia songziensis Wang DM, Hao et Wang Q, 2005 1977 Feng Shaonan et al., pl.235, 1-4

Sublepidodendron (Nathorst) Hirmer, 1927 emend. Wang Q, Hao, Wang DM, Wang Y et Thomas Denk, 2003

Sublepidodendron songziense Chen ex Wang Q, Hao, Wang DM, Wang Y, et Thomas Denk. 1977 Feng Shaonan et al., pl.231, 1-2; 1984 Chen Gongxin, pl. 217, 7

Tizikou Section, Yidu County: Tizikou Formation (Strunian)

Cyclostigma Haughton ex Heer, 1871

Cyclostigma? *yiduense* Feng 1984 1984 Feng Shaonan, pl.48, 13

Rotafolia Wang DM, Hao et Wang Q, 2005

Rotafolia songziensis Wang DM, 2005 1984 Feng Shaonan, pl.48, 9, 11, 12; 1984 Chen Gongxin, pl. 218, 1-4; 2005 Wang DM et al., figs.2-42; 2006 Wang DM et al., fig. 1-6

Sublepidodendron (Nathorst) Hirmer, 1927 emend. Wang Q, Hao, Wang DM, Wang Y et Thomas Denk, 2003

Sublepidodendron songziense Chen ex Wang Q, Hao, Wang DM, Wang Y, et Thomas Denk. emend 1984 Feng Shaonan, pls. 47, 13; 48, 8, 10

Sublepidodendron sp. 1984 Feng Shaonan, pl.48, 3

5. Maozhuang Village, Wufeng County: Xiejingsi Formation

Lepidodendropsis Lutz, 1933

Lepidodendropsis cyclostigmatoides Jongmans, Gothan et Darrah, 1937 1975 Feng et Meng, pls. II, 12; III, 3-7; 1977 Feng Shaonan et al., pl.230, 12-14

Lepidostrobus Brongniart, 1828 emend. Brack-Hanes and Thomas, 1983

Lepidostrobus wufengensis Feng et Meng 1977 Feng et al., pl.234, 8-9; 1975 Feng et Meng, pl.I, 9-11; 1984 Chen Gongxin, pl. 217, 2

Leptophloeum Dawson, 1861 emend. Li, Dou et Sun, 1986

Leptophloeum rhombicum Dawson 1975 Feng et Meng , pl. II, 1-4

Stigmaria Brongniart, 1822

Stigmaria ficoides (Sternberg) Brongniart 1975 Feng et Meng , pl. II, 11; 1984 Chen Gongxin, pl. 216, 9

Sublepidodendron (Nathorst) Hirmer, 1927 emend. Wang Q, Hao, Wang DM, Wang Y et Thomas Denk, 2003

Sublepidodendron mirabile (Nathorst) Hirmer 1927 1977 Feng Shaonan et al., pl.231, 14; 1975 Feng et Meng, pl.I, 15; pl.II, 5-7; 1984 Chen Gongxin, pl. 218, 7

6. Huangkuang Section, Liujiachang Town, Songzi County: Xiejingsi Formation

Lepidodendropsis Lutz, 1933

Lepidodendropsis guanzhuangensis Feng et Meng 1984 Chen Gongxin, pl. 216, 3

Lepidodendropsis hirmeri Lutz 1984 Chen Gongxin, pl. 217, 3

Lepidodendropsis scobiniformis (Meek) Read 1977 Feng Shaonan et al., pl.231, 10-11

Rotafolia Wang DM, Hao et Wang Q, 2005

Rotafolia songziensis Wang DM, Hao et Wang Q, 2005 2005 Wang DM et al., figs.2-42; 2006 Wang DM et al., fig. 1-6

Sublepidodendron (Nathorst) Hirmer, 1927 emend. Wang Q, Hao, Wang DM, Wang Y et Thomas Denk, 2003

Sublepidodendron grabaui (Sze) Wang et Xu, 2005 1977 Feng Shaonan et al., pl.234, 12-13; 1984 Chen Gongxin, pl. 217, 1

Sublepidodendron shimenense Feng et Meng, 1975 1977 Feng Shaonan et al., pl.231, 16

Sublepidodendron songziense Chen ex Wang Q, Hao, Wang DM, Wang Y, et Thomas Denk. 1977 Feng Shaonan et al., pl.231, 1-2; 1984 Chen Gongxin, pl. 217, 7; 2002 Wang Qi et al., figs. 1-30; 2003 Wang Qi et al., pls. I-VI; figs. 4-6

Huangkuang Section, Liujiachang Town, Songzi County: Tizikou Formation (Strunian)

Archaeosigillaria (Kidston) Lacey, 1962

Archaeosigillaria? *vanuxemi* (Göppert) Kidston 1984 Feng Shaonan, pl.47, 12

Barinophyton White, 1905

Barinophyton citrulliforme Arnold 1984 Feng Shaonan, pl.48, 1-2

Cyclostigma Haughton ex Heer, 1871

Cyclostigma kiltorkense Haughton ex Heer 1871 1984 Feng Shaonan, pl.47, 1-3

Drepanophycus Göppert, 1825

Drepanophycus spinaeformis Göppert, 1852 1984 Feng Shaonan, pl.46, 7; pl.47, 6

Drepanophycus spinosus (Krejci) Kräusel et Weyland 1984 Feng Shaonan, pl.47, 10

Lepidodendropsis Lutz, 1933

Lepidodendropsis hirmeri Lutz 1984 Feng Shaonan, pl.49, 11

Rotafolia Wang DM, Hao et Wang Q, 2005

Rotafolia songziensis Wang DM, 2005 1984 Feng Shaonan, pl.48, 4, 9, 11, 12; 1991 Feng shaonan et Ma Jie, pls. I-II

Sublepidodendron (Nathorst) Hirmer, 1927 emend. Wang Q, Hao, Wang DM, Wang Y et Thomas Denk,

<u>2003</u>

Sublepidodendron songziense Chen ex Wang Q, Hao, Wang DM, Wang Y, et Thomas Denk. 1984 Feng Shaonan, pls.47, 9; 49, 12

Late Famennian to Early Tournaisian

Anhui

Lintou, Chaohu City: Wutung Formation

<u>*Eolepidodendron* Wu et Zhao, 1981</u>

Eolepidodendron wusihense (Sze) Wu et Zhao, 1981 1982 Li Hanmin et al., pl.131, 4-5

<u>*Lepidodendropsis* Lutz, 1933</u>

Lepidodendropsis hirmeri Lutz 1982 Li Hanmin et al., pl.129, 1-4

Jiangsu

1. Nanjing: Wutung Formation

<u>*Lepidodendropsis* Lutz, 1933</u>

Lepidodendropsis hirmeri Lutz 1982 Li Hanmin et al., pl.129, 1-4

<u>*Leptophloeum* Dawson, 1861 emend. Li, Dou et Sun, 1986</u>

Leptophloeum rhombicum Dawson 1982 Li Hanmin et al., pl.130, 8

2. Longtan, Nanjing; Taihu, Wuxi City: Wutung Formation

<u>*Lepidodendron* Sternberg, 1820</u>

Lepidodendron procurrens Gothen et Sze 1953 Sze, pl.5, 1-2

Lepidodendron sp. 1953 Sze, pl.7, 6

<u>*Lepidodendropsis* Lutz, 1933</u>

Lepidodendropsis hirmeri Lutz 1956 Sze, pl.1; fig.1-3; 1974 Gu et Zhi, pl. V, 1-2

Lepidodendropsis scobiniformis (Meek) Read 1965a Zhang Qianshen, pl.I, 1, 1a

<u>*Lepidophyllum* Brongniart, 1828</u>

Lepidophyllum xiphidium Gothan et Sze 1953 Sze, pl.5, 8; 1954 Sze et Hsü, pl.4, 3

Lepidophyllum (*Cantheliophorus*) cf. *mirabile* Nathorst 1953 Sze, pl.5, 9; 1954 Sze et Hsü, pl.4, 2

<u>*Lepidostrobus* Brongniart, 1828 emend. Brack-Hanes and Thomas, 1983</u>

Lepidostrobus ungulatus Gothan et Sze 1953 Sze, pl.7, 7-8

<u>*Lepidostrobophyllum* Hirmer, 1927</u>

Lepidostrobophyllum xiphidium (Gothan et Sze) Gu et Zhi, 1974 1974 Gu et Zhi, pl.17, 3

Lepidostrobophyllum sp. 1974 Gu et Zhi, pl.17, 15

<u>*Sphenophyllum* Brongniart 1828</u>

Sphenophyllum lungtanense Gothan et Sze emend. Gu et Zhi 1953 Sze, pl.5, 5-6; 1974 Gu et Zhi, pl.22, 1-4; 1982 Li Hanmin et al., pl.137, 6-7

Sphenophyllum pseudotenerrimum Sze 1953 Sze, pl.12, 1-4; pl.15, 7; 1954 Sze et Hsü, pl.6, 1-3; 1974 Gu et Zhi, pl.21, 2-4

Sphenophyllum sp. 1953 Sze, pl.5, 7

<u>*Sphenopteris* (Brongniart) Sternberg, 1825</u>

Sphenopteris taihuensis Sze 1953 Sze, pl.22, 3-4

<u>*Stigmaria* Brongniart, 1822</u>

Stigmaria ficoides (Sternberg) Brongniart 1953 Sze, pl.5, 4

Stigmaria radiato-punctata Gothan et Sze1953 Sze, pl.5, 3; 1954 Sze et Hsü, pl.3, 1; 1974 Gu et Zhi, pl.20, 5

Stigmaria rugulosa Gothan 1974 Gu et Zhi, pl.20, 6-7

Sublepidodendron (Nathorst) Hirmer, 1927 emend. Wang Q, Hao, Wang DM, Wang Y et Thomas Denk, 2003

Sublepidodendron grabaui (Sze) Wang et Xu, 2005 1953 Sze, pls.6, 1-7; 8, 2-3; 1954 Sze et Hsü, pl.3, 3-5; 1974 Gu et Zhi, pl.16, 1-4, 7-8

Sublepidodendron mirabile (Nathorst) Hirmer, 1927 1953 Sze, pl.7, 1-5; 1954 Sze et Hsü, pl.4, 1; pl.3, 6-7; 1974 Gu et Zhi, pl.5, 9-11

3. Mituoshan, Guangfu Town, Suzhou City: Wutung Formation

Leptophloeum Dawson, 1861 emend. Li, Dou et Sun, 1986

Leptophloeum rhombicum Dawson 1974 Gu et Zhi, pl.4, 2-5; fig.25

Leptophloeum suzhouense Chang, 1965 1965a Zhang Qianshen, pl.I, 2-3

4. Wuxi: Wutung Formation

Sublepidodendron (Nathorst) Hirmer, 1927 emend. Wang Q, Hao, Wang DM, Wang Y et Thomas Denk, 2003

Sublepidodendron grabaui (Sze) Wang et Xu, 2005 1982 Li Hanmin et al., pl.142, 6

5. Wuxi: Upper Wutung Formation

Sphenopteris (Brongniart) Sternberg, 1825

Sphenopteris taihuensis Sze 1982 Li Hanmin et al., pl.142, 7-10

6. Yixing: Wutung Formation

Platyphyllum Dawson, 1888

Platyphyllum? cf. *williamsonii* (Nathorst) Hœg 1982 Li Hanmin et al., pl.142, 1-2

Zhejiang

1. Baitashan, Hangzhou City: Xihu Formation (late Famennian to early Tournaisian)

Leptophloeum Dawson, 1861 emend. Li, Dou et Sun, 1986

Leptophloeum rhombicum Dawson 1999 Chen Qishi, p. 15

Sublepidodendron (Nathorst) Hirmer, 1927 emend. Wang Q, Hao, Wang DM, Wang Y et Thomas Denk, 2003

Sublepidodendron grabaui (Sze) Wang et Xu, 2005 1999 Chen Qishi, p. 15

Sublepidodendron cf. *mirabile* (Nathorst) Hirmer, 1927 1999 Chen Qishi, p. 15

2. Changshantou Village, Xiaoshan City: Xihu Formation (late Famennian to early Tournaisian)

Eolepidodendron Wu et Zhao, 1981

Eolepidodendron hushanense Chen 1999 Chen Qishi, p. 15

Eolepidodendron cf. *wusihense* (Sze) Wu et Zhao, 1981 1999 Chen Qishi, p. 15

Lepidodendropsis Lutz, 1933

Lepidodendropsis sp. 1999 Chen Qishi, p. 15

Leptophloeum Dawson, 1861 emend. Li, Dou et Sun, 1986

Leptophloeum rhombicum Dawson 1999 Chen Qishi, p. 15

Spinolepidodendron Chen, 1999

Spinolepidodendron hangzhouense Chen 1999 Chen Qishi, p. 15

Spinolepidodendron xiaoshanense Chen 1999 Chen Qishi, p. 15

Spinolepidodendron sp. 1999 Chen Qishi, p. 15

Sublepidodendron (Nathorst) Hirmer, 1927 emend. Wang Q, Hao, Wang DM, Wang Y et Thomas Denk, 2003

Sublepidodendron cf. *mirabile* (Nathorst) Hirmer 1927 1999 Chen Qishi, p. 15

3. Maowan, Fuyang City: Xihu Formation (late Famennian to early Tournaisian)

Lepidodendropsis Lutz, 1933

Lepidodendropsis cf. *hirmeri* Lutz 1999 Chen Qishi, p. 15

Sublepidodendron (Nathorst) Hirmer, 1927 emend. Wang Q, Hao, Wang DM, Wang Y et Thomas Denk, 2003

Sublepidodendron cf. *grabaui* (Sze) Wang et Xu 1999 Chen Qishi, p. 15

Sublepidodendron sp. 1999 Chen Qishi, p. 15

4. Dalaowukou, Tonglu County: Xihu Formation (late Famennian to early Tournaisian)

Sublepidodendron (Nathorst) Hirmer, 1927 emend. Wang Q, Hao, Wang DM, Wang Y et Thomas Denk, 2003

Sublepidodendron sp. 1999 Chen Qishi, p. 15

5. Xin'anjiangba, Jiande City: Xihu Formation (late Famennian to early Tournaisian)

Leptophloeum Dawson, 1861 emend. Li, Dou et Sun, 1986

Leptophloeum rhombicum Dawson 1999 Chen Qishi, p. 15

6. Dongshansi, Jiande City: Xihu Formation (late Famennian to early Tournaisian)

Sublepidodendron (Nathorst) Hirmer, 1927 emend. Wang Q, Hao, Wang DM, Wang Y et Thomas Denk, 2003

Sublepidodendron grabaui (Sze) Wang et Xu, 2005 1999 Chen Qishi, p. 16

Sublepidodendron mirabile (Nathorst) Hirmer, 1927 1999 Chen Qishi, p. 16

7. Xiangudong Section, Chun'an County: Xihu Formation (late Famennian to early Tournaisian)

Leptophloeum Dawson, 1861 emend. Li, Dou et Sun, 1986

Leptophloeum rhombicum Dawson 1999 Chen Qishi, p. 16

8. Daliyuan Village, Shangfang Town, Quxian County: Xihu Formation (late Famennian to early Tournaisian)

Leptophloeum Dawson, 1861 emend. Li, Dou et Sun, 1986

Leptophloeum rhombicum Dawson 1999 Chen Qishi, pl. I, 1-9; fig. 1

9. Hushan, Xiaoshan City: Xihu Formation (late Famennian to early Tournaisian)

Eolepidodendron Wu et Zhao, 1981

Eolepidodendron hushanense Chen 1999 1999 Chen Qishi, pl. II, 8; fig. 2

Knorria Sternberg, 1825

Knorria? sp. 1999 Chen Qishi, pl. V, 7-8

Lepidodendropsis Lutz, 1933

Lepidodendropsis sp. 1999 Chen Qishi, pl. V, 4-6

Leptophloeum Dawson, 1861 emend. Li, Dou et Sun, 1986

Leptophloeum rhombicum Dawson 1999 Chen Qishi, pl. I, 1-9; fig. 1

Sphenophyllum Brongniart, 1828

Sphenophyllum lungtanense Gothan et Sze emend. Gu et Zhi 1988 Chen, pl. IV, 1-6; fig. 5

Sphenophyllum pseudotenerrimum Sze 1988 Chen, pl. III, 7-11

Spinolepidodendron Chen, 1999

Spinolepidodendron hangzhouense Chen, 1999 1999 Chen Qishi, pls. II, 1-7b; III, 1-1a

Sublepidodendron (Nathorst) Hirmer, 1927 emend. Wang Q, Hao, Wang DM, Wang Y et Thomas Denk, 2003

Sublepidodendron xiaoshanense Chen, 1999 1999 Chen Qishi, pls I, 11; III, 2-3a; IV, 1, 2a; V, 1-2a; figs. 3-4

Sublepidodendron sp.? 1999 Chen Qishi, pl. V, 2b

Sublepidodendron sp. 1999 Chen Qishi, pl. V, 3

Xihuphyllum Chen, 1988

Xihuphyllum elongatum Chen 1988 1988 Chen, pl.4, 8-11; fig.4

Xihuphyllum megalofolium (Wu) Chen, 1988 1988 Chen, pls.1-2; pl.3, 1-6; pl.4, 7; pl.5, 1-5; figs.1-3; 1995 Cai Chongyang et Li Xingxue, pl. VI, 1-3

10. Shiji Village, Si'an Town, Changxing City: Wutung Formation

Eolepidodendron Wu et Zhao, 1981

Eolepidodendron cf. *wusihense* (Sze) Wu et Zhao, 1981 1999 Chen Qishi, pl. I, 10

Eviostachya Stockmans, 1948

Eviostachya hoegii Stockmans 2001 Chen Qishi, pls. I, 1A-5; II, 3a-3c

Hamatophyton Gu et Zhi, 1974 emend. Li, Cai et Wang, 1995

Hamatophyton verticillatum Gu et Zhi, 1974 emend. Li, Cai et Wang, 1995 2001 Chen Qishi, pls. II, 4-8, 11; III, 1-11

Lepidodendropsis Lutz, 1933

Lepidodendropsis sp. 1999 Chen Qishi, pl. V, 4-6

Leptophloeum Dawson, 1861 emend. Li, Dou et Sun, 1986

Leptophloeum rhombicum Dawson 1999 Chen Qishi, pl. I, 1-9; fig. 1

Sphenophyllostachys Seward

Sphenophyllostachys? sp. 2001 Chen Qishi, pl. I, 6-8

Sphenophyllum Brongniart, 1928

Sphenophyllum pseudotenerrimum Sze 2001 Chen Qishi, pl. II,9-10

Sublepidodendron (Nathorst) Hirmer, 1927 emend. Wang Q, Hao, Wang DM, Wang Y et Thomas Denk, 2003

Sublepidodendron grabaui (Sze) Wang et Xu, 2005 1999 Chen Qishi, p. 16

Sublepidodendron xiaoshanense Chen, 1999 1999 Chen Qishi, pls I, 11; III, 2-3a; IV, 1, 2a; V, 1-2a; figs. 3-4

11. Chanling Village, Changxing City: Wutung Formation

Stigmaria Brongniart, 1822

Stigmaria rugulosa Gothan 1999 Chen Qishi, p. 16
Sublepidodendron (Nathorst) Hirmer, 1927 emend. Wang Q, Hao, Wang DM, Wang Y et Thomas Denk, 2003
Sublepidodendron grabaui (Sze) Wang et Xu, 2005 1999 Chen Qishi, p. 16
Sublepidodendron mirabile (Nathorst) Hirmer, 1927 1999 Chen Qishi, p. 16
Sublepidodendron sp. 1999 Chen Qishi, p. 16

12. Xushan, Jiashan County: Wutung Formation
Sublepidodendron (Nathorst) Hirmer, 1927 emend. Wang Q, Hao, Wang DM, Wang Y et Thomas Denk, 2003
Sublepidodendron cf. *grabaui* (Sze) Wang et Xu 1999 Chen Qishi, p. 16
Sublepidodendron cf. *mirabile* (Nathorst) Hirmer, 1927 1999 Chen Qishi, p. 16

Jiangxi

1. Near Xiushui: Upper Wutung Formation
Leptophloeum Dawson, 1861 emend. Li, Dou et Sun, 1986
Leptophloeum rhombicum Dawson 1978 Zhou Dianchao et Zhu Zhenggang, p. 186
Sublepidodendron (Nathorst) Hirmer, 1927 emend. Wang Q, Hao, Wang DM, Wang Y et Thomas Denk, 2003
Sublepidodendron grabaui (Sze) Wang et Xu, 2005 1978 Zhou Dianchao et Zhu Zhenggang, p. 186
Sublepidodendron mirabile (Nathorst) Hirmer, 1927 1978 Zhou Dianchao et Zhu Zhenggang, p. 186

2. Near Wuning: Upper Wutung Formation
Leptophloeum Dawson, 1861 emend. Li, Dou et Sun, 1986
Leptophloeum rhombicum Dawson 1978 Zhou Dianchao et Zhu Zhenggang, p. 186
Sublepidodendron (Nathorst) Hirmer, 1927 emend. Wang Q, Hao, Wang DM, Wang Y et Thomas Denk, 2003
Sublepidodendron grabaui (Sze) Wang et Xu, 2005 1978 Zhou Dianchao et Zhu Zhenggang, p. 186
Sublepidodendron mirabile (Nathorst) Hirmer, 1927 1978 Zhou Dianchao et Zhu Zhenggang, p. 186

3. Near Duchang: Upper Wutung Formation
Leptophloeum Dawson, 1861 emend. Li, Dou et Sun, 1986
Leptophloeum rhombicum Dawson 1978 Zhou Dianchao et Zhu Zhenggang, p. 186
Sublepidodendron (Nathorst) Hirmer, 1927 emend. Wang Q, Hao, Wang DM, Wang Y et Thomas Denk, 2003
Sublepidodendron grabaui (Sze) Wang et Xu, 2005 1978 Zhou Dianchao et Zhu Zhenggang, p. 186
Sublepidodendron mirabile (Nathorst) Hirmer, 1927 1978 Zhou Dianchao et Zhu Zhenggang, p. 186

4. Near Yushan: Upper Wutung Formation
Leptophloeum Dawson, 1861 emend. Li, Dou et Sun, 1986
Leptophloeum rhombicum Dawson 1978 Zhou Dianchao et Zhu Zhenggang, p. 186
Sublepidodendron (Nathorst) Hirmer, 1927 emend. Wang Q, Hao, Wang DM, Wang Y et Thomas Denk, 2003

Sublepidodendron grabaui (Sze) Wang et Xu, 2005 1978 Zhou Dianchao et Zhu Zhenggang, p. 186
Sublepidodendron mirabile (Nathorst) Hirmer, 1927 1978 Zhou Dianchao et Zhu Zhenggang, p. 186

5. Wutung Formation
Sublepidodendron (Nathorst) Hirmer, 1927 emend. Wang Q, Hao, Wang DM, Wang Y et Thomas Denk, 2003
Sublepidodendron mirabile (Nathorst) Hirmer, 1927 1943 Sze, pl.1; figs.1, 2

6. Pangushan, Anyuan County
Leptophloeum Dawson, 1861 emend. Li, Dou et Sun, 1986
Leptophloeum rhombicum Dawson 1952 Sze, pl. IV, 1

7. Wushiling, Yongxin County: Wutung Formation
Hamatophyton Gu et Zhi, 1974 emend. Li, Cai and Wang, 1995
Hamatophyton verticillatum Gu et Zhi, 1974 emend. Li, Cai and Wang, 1995 1974 Gu et Zhi, pl.19, 3-5; pl.20, 1-4

Guangdong

1. Kaiping
Cyclostigma Haughton ex Heer, 1871
Cyclostigma kiltorkense Haughton 1982 Cai et Li, p. 117
Leptophloeum Dawson, 1861 emend. Li, Dou et Sun, 1986
Leptophloeum rhombicum Dawson 1982 Cai et Li, p. 117
Platyphyllum Dawson, 1888
Platyphyllum sp. 1982 Cai et Li, p. 117
Sphenophyllum Brongniart, 1928
Sphenophyllum pseudotenerrimum 1982 Cai et Li, p. 117
Sublepidodendron (Nathorst) Hirmer, 1927 emend. Wang Q, Hao, Wang DM, Wang Y et Thomas Denk, 2003
Sublepidodendron sp. 1982 Cai et Li, p. 117

2. Taishan
Leptophloeum Dawson, 1861 emend. Li, Dou et Sun, 1986
Leptophloeum rhombicum Dawson 1982 Cai et Li, p. 117
Sublepidodendron (Nathorst) Hirmer, 1927 emend. Wang Q, Hao, Wang DM, Wang Y et Thomas Denk, 2003
Sublepidodendron? *grabaui* (Sze) Wang et Xu 1982 Cai et Li, p. 117
Sublepidodendron mirabile 1982 Cai et Li, p. 117

3. Lianjiang
Carpolithus Brongniart, 1822
Carpolithus sp. 1982 Cai et Li, p. 117
Leptophloeum Dawson, 1861 emend. Li, Dou et Sun, 1986
Leptophloeum rhombicum Dawson 1982 Cai et Li, p. 117

Sublepidodendron (Nathorst) Hirmer, 1927 emend. Wang Q, Hao, Wang DM, Wang Y et Thomas Denk, 2003

Sublepidodendron? *grabaui* (Sze) Wang et Xu 1982 Cai et Li, p. 117

4. Daguling, Huadu District, Guangzhou City

Leptophloeum Dawson, 1861 emend. Li, Dou et Sun, 1986

Leptophloeum rhombicum Dawson 1952 Sze, pl. I, 7

Early Tournaisian (Early Carboniferous)

Guangdong

1. Shashuigang Section, Guangzhou City: Longjiang Formation

Archaeocalamites Stur, 1875

Archaeocalamites sp. 1993 Wu Qijun et Dong Lingling, p. 112; 2000 Jin Jianhua et al., p. 116

Eolepidodendron Wu et Zhao, 1981

Eolepidodendron wusihense (Sze) Wu et Zhao, 1981 1993 Wu Qijun et Dong Lingling, p. 112; 2000 Jin Jianhua et al., p. 116

Hamatophyton Gu et Zhi, 1974 emend. Li, Cai and Wang, 1995

Hamatophyton verticillatum Gu et Zhi, 1974 emend. Li, Cai and Wang, 1995 emend. 1993 Wu Qijun et Dong Lingling, p. 112; 2000 Jin Jianhua et al., p. 116

Rhodeopteridium (Presl) Zimmermann, 1959

Rhodeopteridium cf. *hsianghsiangense* (Sze) 2000 Jin Jianhua et al., p. 116

Stigmaria Brongniart, 1822

Stigmaria ficoides (Sternberg) Brongniart 1993 Wu Qijun et Dong Lingling, p. 112; 2000 Jin Jianhua et al., p. 116

Sublepidodendron (Nathorst) Hirmer, 1927 emend. Wang Q, Hao, Wang DM, Wang Y et Thomas Denk, 2003

Sublepidodendron mirabile (Nathorst) Hirmer, 1927 1993 Wu Qijun et Dong Lingling, p. 112; 2000 Jin Jianhua et al., p. 116

Sublepidodendron cf. *mirabile* (Nathorst) Hirmer, 1927 1993 Wu Qijun et Dong Lingling, p. 112; 2000 Jin Jianhua et al., p. 116

Sublepidodendron sp. 2 1993 Wu Qijun et Dong Lingling, p. 112; 2000 Jin Jianhua et al., p. 116

2. Hukeng, Renhua County, Shaoguan City: Maozifeng Formation (Tournaisian)

Archaeocalamites Stur, 1875

Archaeocalamites scrobiculatus Schlotheim ex Seward 1990 Zhao Ruxuan et Qin Guorong, tab. 2

Eolepidodendron Wu et Zhao, 1981

Eolepidodendron wusihense (Sze) Wu et Zhao, 1981 1990 Zhao Ruxuan et Qin Guorong, tab. 2

Hamatophyton Gu et Zhi, 1974 emend. Li, Cai et Wang, 1995

Hamatophyton sp. 1990 Zhao Ruxuan et Qin Guorong, tab. 2

Lepidodendron Sternberg, 1820

Lepidodendron sp. 1990 Zhao Ruxuan et Qin Guorong, tab. 2

Stigmaria Brongniart, 1822

Stigmaria rugolosa 1990 Zhao Ruxuan et Qin Guorong, tab. 2

Sublepidodendron (Nathorst) Hirmer, 1927 emend. Wang Q, Hao, Wang DM, Wang Y et Thomas Denk, 2003

Sublepidodendron mirabile (Nathorst) Hirmer, 1927 1990 Zhao Ruxuan et Qin Guorong, tab. 2

Sublepidodendron songziense Chen ex Wang Q, Hao, Wang DM, Wang Y, et Thomas Denk. 1990 Zhao Ruxuan et Qin Guorong, tab. 2

3. Qingshuitang, Shixing County, Shaoguan City: Maozifeng Formation (Tournaisian)

Archaeocalamites Stur, 1875

Archaeocalamites scrobiculatus Schlotheim ex Seward 1990 Zhao Ruxuan et Qin Guorong, tab. 2

Eolepidodendron Wu et Zhao, 1981

Eolepidodendron wusihense (Sze) Wu et Zhao, 1981 1990 Zhao Ruxuan et Qin Guorong, tab. 2

Protolepidodendron Krejči ex Gothan, 1921

Protolepidodendron? sp. 1990 Zhao Ruxuan et Qin Guorong, tab. 2

Jiangsu

1. Huanglongshan Mountain, Longtan Town, Nanjing City: Middle–Upper Wutung Formation (early Tournaisian)

Lepidodendropsis Lutz, 1933

Lepidodendropsis hirmeri Lutz 1959 He Xilin, pl. I, 1

2. Leigutai Section, Longtan Town, Nanjing City: Upper Leigutai Member of Wutung Formation

Carpolithus Brongniart, 1822

Carpolithus sp. 1988 Cai Chongyang et al., p. 175

Hamatophyton Gu et Zhi, 1974 emend. Li, Cai et Wang, 1995

Hamatophyton verticillatum Gu et Zhi, 1974 emend. Li, Cai et Wang, 1995 1988 Cai Chongyang et al., p. 175

Lepidostrobophyllum Hirmer, 1927

Lepidostrobophyllum sp. 1988 Cai Chongyang et al., p. 175

Sphenophyllum Brongniart, 1828

Sphenophyllum lungtanense Gothan et Sze emend. Gu et Zhi 1988 Cai Chongyang et al., p. 175

Sphenophyllum sp. 1988 Cai Chongyang et al., p. 175

Sphenopteridium Schimper, 1874

Sphenopteridium? sp. 1988 Cai Chongyang et al., p. 175

Sphenopteris (Brongniart) Sternberg, 1825

Sphenopteris sp. 1988 Cai Chongyang et al., p. 175

Sublepidodendron (Nathorst) Hirmer, 1927 emend. Wang Q, Hao, Wang DM, Wang Y et Thomas Denk, 2003

Sublepidodendron sp. 1988 Cai Chongyang et al., p. 175

Sublepidodendron? *grabaui* (Sze) Wang et Xu, 2005 1988 Cai Chongyang et al., p. 175

Sublepidodendron mirabile (Nathorst) Hirmer, 1927 1988 Cai Chongyang et al., p. 175

3. Cishan, Nanjing City: Cishan Formation (early Tournaisian)

Eolepidodendron Wu et Zhao, 1981

Eolepidodendron wusihense (Sze) Wu et Zhao, 1981 1987 Li Hanmin et al., p. 118

Rhacophyton Crépin, 1875

Rhacophyton sp. 1987 Li Hanmin et al., p. 118

Stigmaria Brongniart, 1822

Stigmaria ficoides (Sternberg) Brongniart 1987 Li Hanmin et al., p. 118

Sublepidodendron (Nathorst) Hirmer, 1927 emend. Wang Q, Hao, Wang DM, Wang Y et Thomas Denk,

2003

Sublepidodendron mirabile (Nathorst) Hirmer, 1927 1987 Li Hanmin et al., p. 118

4. Kongshan, Nanjing City: Wutung Formation

Helicophyton Wang et Xu, 2002

Helicophyton dichotomum Wang et Xu, 2002 2002 Wang Yi et Xu, fig. 1-6

Multifurcatus Wang, 2003

Multifurcatus tenellus Wang, 2003 2003 Wang Yi, figs. 1-8

5. A cement factory, Dingshu Town, Yixing City: Wutung Formation

Hamatophyton Gu et Zhi, 1974 emend. Li, Cai et Wang, 1995

Hamatophyton verticillatum Gu et Zhi, 1974 emend. Li, Cai et Wang, 1995 1988 Cai Chongyang et al., p. 183

Lepidostrobus Brongniart, 1828 emend. Brack-Hanes and Thomas, 1983

Lepidostrobus sp. 1988 Cai Chongyang et al., p. 183

Stigmaria Brongniart, 1822

Stigmaria sp. 1988 Cai Chongyang et al., p. 183

Sublepidodendron (Nathorst) Hirmer, 1927 emend. Wang Q, Hao, Wang DM, Wang Y et Thomas Denk, 2003

Sublepidodendron grabaui (Sze) Wang et Xu, 2005 1988 Cai Chongyang et al., p. 183

Sublepidodendron mirabile (Nathorst) Hirmer, 1927 1988 Cai Chongyang et al., p. 183

6. Wuxi: Top of Wutung Formation

Archaeopteris (Dawson) Stur, 1875

Archaeopteris macilenta (Lesquereux) Carluccio, Hueber et Banks, 1966 1987 Yang Zhaorong, p.285

Anhui

Shizikou Section, Chaohu City: Wutung Formation

Coenosophyton Wang et Xu

Coenosophyton tristichus Wang et Xu, 2003 2003 Wang Yi et Xu, Figs. 2-7

Hunan

1. Xuefeng Mountain: Yan'guan Stage

Rhodeopteridium (Presl) Zimmermann, 1959

Rhodeopteridium sp. 1982 Cheng Lizhu, pl.330, 4, 5

2. Guankou, Liuyang City: Yan'guan Stage

Sublepidodendron (Nathorst) Hirmer, 1927 emend. Wang Q, Hao, Wang DM, Wang Y et Thomas Denk, 2003

Sublepidodendron grabaui (Sze) Wang et Xu 1982 Cheng Lizhu, pl.327, 2, 3

Late Tournaisian

Zhejiang

1. Gaoshan Village, Tonglu County: Zhucangwu Formation

Sphenopteridium Schimper, 1874

Sphenopteridium sp. 1987 Chen Qishi, pl.4, 4

Sublepidodendron (Nathorst) Hirmer, 1927 emend. Wang Q, Hao, Wang DM, Wang Y et Thomas Denk, 2003

Sublepidodendron mirabile (Nathorst) Hirmer, 1927 1986 Zhao Xiuhu et al., pl.I, 1-3, 1a, 2a

2. Xintong Town, Fuyang County: Zhucangwu Formation

Eolepidodendron Wu et Zhao, 1981

Eolepidodendron wusihense (Sze) Wu et Zhao, 1981 1986 Zhao Xiuhu et al., p. 6

Lepidodendropsis Lutz, 1933

Lepidodendropsis sp. 1986 Zhao Xiuhu et al., p. 5

Sublepidodendron (Nathorst) Hirmer, 1927 emend. Wang Q, Hao, Wang DM, Wang Y et Thomas Denk, 2003

Sublepidodendron mirabile (Nathorst) Hirmer, 1927 1986 Zhao Xiuhu et al., pl.I, 1-3, 1a, 2a

Sublepidodendron sp. 1986 Zhao Xiuhu et al., pl.I, 5, 5a

3. Maowan, Fuyang County: Zhucangwu Formation

Bothrodendron Lindley et Hutton, 1833

Bothrodendron fuyangense Chen, 1986 1986 Zhao Xiuhu et al., pl.IV, 1, 1a, 1b, 1c

Eolepidodendron Wu et Zhao, 1981

Eolepidodendron cf. *wusihense* (Sze) Wu et Zhao, 1981 1986 Zhao Xiuhu et al., pl.I, 4, 4a

Lepidodendropsis Lutz, 1933

Lepidodendropsis cf. *hirmeri* Lutz 1986 Zhao Xiuhu et al., p. 6

4. Youshuwu, Fuyang County: Zhucangwu Formation

Bothrodendron Lindley et Hutton, 1833

Bothrodendron fuyangense Chen, 1986 1987 Chen Qishi, pl.4, 1

Eolepidodendron Wu et Zhao, 1981

Eolepidodendron cf. *wusihense* (Sze) Wu et Zhao, 1981 1987 Chen Qishi, pl.4, 2

5. Meidongwu, Fuyang County: Zhucangwu Formation

Sublepidodendron (Nathorst) Hirmer, 1927 emend. Wang Q, Hao, Wang DM, Wang Y et Thomas Denk, 2003

Sublepidodendron mirabile (Nathorst) Hirmer, 1927 1986 Zhao Xiuhu et al., pl.I, 1-3, 1a, 2a; 1987 Chen Qishi, pl.4, 3

6. Zhucangwu Section, Linghu Village, Changshan County: Zhucangwu Formation

Hunan

1. Xuefeng Mountain: Yan’guan Stage

Rhodeopteridium (Presl) Zimmermann, 1959

Rhodeopteridium sp. 1982 Cheng Lizhu, pl.330, 4, 5

2. Guankou, Liuyang City: Yan'guan Stage

Sublepidodendron (Nathorst) Hirmer, 1927 emend. Wang Q, Hao, Wang DM, Wang Y et Thomas Denk, 2003

Sublepidodendron grabaui (Sze) Wang et Xu 1982 Cheng Lizhu, pl.327, 2, 3

Visean

Hubei

1. Xiangxi Village, Ziqiu Town, Changyang County: lower Gaolishan Formation

Lepidostrobus Brongniart, 1828 emend. Brack-Hanes and Thomas, 1983

Lepidostrobus sp. 2008 Cui Li et Wang Deming, fig.2, 4

Stigmaria Brongniart, 1822

Stigmaria ficoides (Sternberg) Brongniart 2008 Cui Li et Wang Deming, fig.2

Sublepidodendron (Nathorst) Hirmer, 1927 emend. Wang Q, Hao, Wang DM, Wang Y et Thomas Denk, 2003

Sublepidodendron mirabile (Nathorst) Hirmer, 1927 2008 Cui Li et Wang Deming, fig.2-3

Sublepidodendron songziense Chen ex Wang Q, Hao, Wang DM, Wang Y, et Thomas Denk. 2008 Cui Li et Wang Deming, fig.2-3

2. Xiangxi Village, Ziqiu Town, Changyang County: Ziqiu Formation

Cardiocarpus Brongniart, 1881

Cardiocarpus changyangensis? Feng 1984 1984 Feng Shaonan, pl.47, 8

Eolepidodendron Wu et Zhao, 1981

Eolepidodendron changyangense? Feng 1984 1984 Feng Shaonan, pl.49, 8

Lepidodendropsis Lutz, 1933

Lepidodendropsis taoshanensis? Feng, 1984 1984 Feng Shaonan, pl.49, 13

Sphenophyllum Brongniart, 1828

Sphenophyllum geigense? Kräusel et Weyland 1984 Feng Shaonan, pl.48, 7

Sublepidodendron (Nathorst) Hirmer, 1927 emend. Wang Q, Hao, Wang DM, Wang Y et Thomas Denk, 2003

Sublepidodendron changyangense? Feng, 1984 1984 Feng Shaonan, pl.49, 6

Sublepidodendron mirabile (Nathorst) Hirmer, 1927 1984 Feng Shaonan, pl.49, 1

Sublepidodendron taoshanense? Feng, 1984 1984 Feng Shaonan, pl.49, 10

Hunan

1. Ceshui, Yongfeng Town, Shuangfeng County: Ceshui Formation

Psygmophyllum Schimper, 1870

Psygmophyllum? sp. 1956 Ao Zhenkuan, p.34

Sphenopteris (Brongniart) Sternberg, 1825

Sphenopteris tseishuiensis Ngo, 1956 1956 Ao Zhenkuan, p.34

2. Wantou Village, Aotou Mountain, Shuangfeng County: Ceshui Formation

Adiantites Göppert, 1836

Adiantites gothani (Sze) Zhang, Zhao et Wu, 1980 1980 Zhang Shanzhen et al., p. 220

Archaeocalamites Stur, 1875

Archaeocalamites prolixus Zhang, Zhao et Wu, 1980 1977 Feng Shaonan et al., pl.236, 11-12; 1980 Zhang Shanzhen et al., p. 220

Cardiopteridium Nathorst, 1914

Cardiopteridium spetsbergense Nathorst 1980 Zhang Shanzhen et al., p. 220; 1977 Feng et al., pl.239, 3-5

Lepidodendron Sternberg, 1820

Lepidodendron cf. *robertii* Nathorst 1980 Zhang Shanzhen et al., pl.III, 3-5, 3a, 4a

Lepidodendron sp. (? sp. nov) 1980 Zhang Shanzhen et al., pl.III, 1-2

Lepidostrobophyllum Hirmer, 1927

Lepidostrobophyllum cf. *lanceolatum* Lindely et Hutton 1974 Gu et Zhi, pl.17, 4-5

Lepidostrobophyllum spp. 1980 Zhang Shanzhen et al., pl.I, 12; pl.II, 6-7

Lepidostrobus Brongniart, 1828 emend. Brack-Hanes and Thomas, 1983

Lepidostrobus sp. 1980 Zhang Shanzhen et al., pl.IV, 2-3, 3a

Rhodeopteridium (Presl) Zimmermann, 1959

Rhodeopteridium cf. *hsianghsiangense* Sze 1980 Zhang Shanzhen et al., p. 220

Stigmaria Brongniart, 1822

Stigmaria ficoides (Sternberg) Brongniart 1980 Zhang Shanzhen et al., p. 220

Stigmaria rugulosa Gothan 1980 Zhang Shanzhen et al., p. 220

Triphyllopteris Schimper, 1869

Triphyllopteris collombiana Schimper 1980 Zhang Shanzhen et al., p. 220

3. Wantou Village, Shuangfeng County: Ceshui Formation

Adiantites Göppert, 1836

Adiantites gothani (Sze) Zhang, Zhao et Wu, 1980 1982 Cheng, pl.329, 1

Archaeocalamites Stur, 1875

Archaeocalamites sp. 1982 Cheng, pl. 327, 8

Stigmaria Brongniart, 1822

Stigmaria ficoides (Sternberg) Brongniart 1982 Cheng Lizhu, pl.326, 4

Triphyllopteris Schimper, 1869

Triphyllopteris collombiana Schimper 1982 Cheng, pl. 330, 2, 2a

4. Aotou Mountain, Shuangfeng County: Ceshui Formation

Archaeocalamites Stur, 1875

Archaeocalamites prolixus Zhang, Zhao et Wu, 1980 1982 Cheng, pl.327, 9

Cardiopteridium Nathorst, 1914

Cardiopteridium spetsbergense Nathorst 1982 Cheng, pl. 329, 2-7

Stigmaria Brongniart, 1822

Stigmaria rugulosa Gothan 1982 Cheng Lizhu, pl.326, 6

5. Near Shuangfeng County: Ceshui Formation

Archaeopteris (Dawson) Stur, 1875

Archaeopteris? *gothani* Sze 1977 Feng Shaonan et al., pl.238, 6-7

Lepidostrobophyllum Hirmer, 1927

Lepidostrobophyllum cf. *lanceolatum* Lindely et Hutton 1977 Feng Shaonan et al., pl. 234, 10-11

Stigmaria Brongniart, 1822

Stigmaria ficoides (Sternberg) Brongniart 1977 Feng Shaonan et al., pl.234, 4

Sublepidodendron (Nathorst) Hirmer, 1927 emend. Wang Q, Hao, Wang DM, Wang Y et Thomas Denk, 2003

Sublepidodendron mirabile (Nathorst) Hirmer, 1927 1977 Feng Shaonan et al., pl.231, 14

Triphyllopteris Schimper, 1869

Triphyllopteris collombiana Schimper 1977 Feng Shaonan et al., pl.239, 6-7

6. Jinshazhou Village, Huishan'gang Town, Taojiang County: Ceshui Formation

Cardiopteridium Nathorst, 1914

Cardiopteridium spetsbergense Nathorst 1982 Cheng, pl. 329, 2-7

Cordaites Unger, 1850

Cordaites schenkii Halle 1982 Cheng Lizhu, pl.333, 6

7. Xinhua: Ceshui Formation

Bothrodendron Lindley et Hutton, 1833

Bothrodendron sp. 1982a Zhao Xiuhu et Wu Xiuyuan, pl.IV, 5, 5a, 5b; pl.V, 1, 1a; fig.2

Cardiocarpus Brongniart, 1881

Cardiocarpus sp. 1982a Zhao Xiuhu et Wu Xiuyuan, pl.XI, 5; pl.XII, 4A, 4Aa

Cardiopteridium Nathorst, 1914

Cardiopteridium podozamioides (Sze) Zhao et Wu, 1982 1982a Zhao Xiuhu et Wu Xiuyuan, pl.VI, 3-14, 7a; ?20-21; pl.VII, 2; fig.4

Cardiopteridium spetsbergense Nathorst 1974 Gu et Zhi, pl.41, 13-20; fig.59; 1977 Feng et al., pl.239, 3-5

Carpolithus Brongniart, 1822

Carpolithus sp. 1982a Zhao Xiuhu et Wu Xiuyuan, pl.XII, 1-3, 20, 1a, 3a

Lepidodendron Sternberg, 1820

Lepidodendron shanyangense Wu et He 1982a Zhao et Wu , pl.II, 4-6, 4a, 6a; pl.III, 1-5, 2a-5a; pl.IV, 4, 4a

Lepidodendron sp. 1 1982a Zhao Xiuhu et Wu Xiuyuan, pl.III, 6, 6a

Sublepidodendron (Nathorst) Hirmer, 1927 emend. Wang Q, Hao, Wang DM, Wang Y et Thomas Denk, 2003

Sublepidodendron mirabile (Nathorst) Hirmer, 1927 1977 Feng Shaonan et al., pl.231, 14

Trigonocarpus Brongniart, 1828

Trigonocarpus sp. 1982a Zhao Xiuhu et Wu Xiuyuan, pl.XI, 12; pl.XII, 4B, 4Ba

8. Lengshuijiang: Ceshui Formation

Archaeocalamites Stur, 1875

Archaeocalamites scrobiculatus Schlotheim ex Seward 1982a Zhao Xiuhu et Wu Xiuyuan, pl. V, 4

Cardiopteridium Nathorst, 1914

Cardiopteridium podozamioides (Sze) Zhao et Wu, 1982 1982a Zhao Xiuhu et Wu Xiuyuan, pl.VI, 3-14, 7a; ?20-21; pl.VII, 2; fig.4

Cardiopteridium spetsbergense Nathorst 1982a Zhao Xiuhu et Wu Xiuyuan, pl. VI, 15-19

<u>*Lepidodendron* Sternberg, 1820</u>
Lepidodendron lengshuijiangense, 1982 1982a Zhao Xiuhu et Wu Xiuyuan, pl.II, 2-3, 3a; fig.1
Lepidodendron shanyangense Wu et He 1982a Zhao et Wu , pl.II, 4-6, 4a, 6a; pl.III, 1-5, 2a-5a; pl.IV, 4, 4a
?*Lepidodendron* sp. 2 1982a Zhao Xiuhu et Wu Xiuyuan, pl.II, 1, 1a
<u>*Rhodeopteridium* (Presl) Zimmermann, 1959</u>
Rhodeopteridium sp.2 1982a Zhao Xiuhu et Wu Xiuyuan, pl.XII, 7, 7a
<u>*Sublepidodendron* (Nathorst) Hirmer, 1927 emend. Wang Q, Hao, Wang DM, Wang Y et Thomas Denk, 2003</u>
Sublepidodendron mirabile (Nathorst) Hirmer, 1927 1982a Zhao Xiuhu et Wu Xiuyuan, pl. I, 1-4
Sublepidodendron cf. *robertii* (Nathorst) Chaloner et Boureau 1982a Zhao Xiuhu et Wu Xiuyuan, pl.I, 5, 5a

9. Rucheng: Ceshui Formation
<u>*Archaeocalamites* Stur, 1875</u>
Archaeocalamites sp. 1982a Zhao Xiuhu et Wu Xiuyuan, pl. V, 3B
<u>*Bothrodendron* Lindley et Hutton, 1833</u>
Bothrodendron ruchengense Zhao et Wu emend. 1982a Zhao et Wu, pl.V, 2, 2a, 2b, 3A, 3Aa
<u>*Cardiopteridium* Nathorst, 1914</u>
Cardiopteridium spitsbergense Nathorst 1982a Zhao Xiuhu et Wu Xiuyuan, pl. VI, 15-19
<u>*Sphenophyllum* Brongniart, 1828</u>
Sphenophyllum tenerrimum Ettinghausen 1982a Zhao Xiuhu et Wu Xiuyuan, pl.V, 7, 8
<u>*Trigonocarpus* Brongniart, 1828</u>
Trigonocarpus sp. 1982a Zhao Xiuhu et Wu Xiuyuan, pl.XI, 12; pl.XII, 4B, 4Ba

10. Lixian: Ceshui Formation
<u>*Cardiopteridium* Nathorst, 1914</u>
Cardiopteridium podozamioides (Sze) Zhao et Wu, 1982 1982a Zhao Xiuhu et Wu Xiuyuan, pl.VI, 3-14, 7a; ?20-21; pl.VII, 2; fig.4
Cardiopteridium spitsbergense Nathorst 1974 Gu et Zhi, pl.41, 13-20; fig.59; 1982a Zhao Xiuhu et Wu Xiuyuan, pl. VI, 15-19
<u>*Neuropteris* (Brongniart) Sternberg, 1825</u>
Neuropteris gigantea Sternberg 1982a Zhao Xiuhu et Wu Xiuyuan, pl. XII, 8-15
Neuropteris sp.1 1982a Zhao Xiuhu et Wu Xiuyuan, pl.XII, 16-18
<u>*Rhodeopteridium* (Presl) Zimmermann, 1959</u>
Rhodeopteridium cf. *hsianghsiangense* Sze 1982 Cheng, pl.330, 6; 1980 Zhang et al., pl.II, 1-3, 1a, 2a

11. Yujiang Coalmine, Lixian County: Ceshui Formation
<u>*Neuropteris* (Brongniart) Sternberg, 1825</u>
Neuropteris gigantea Sternberg 1982 Cheng Lizhu, pl.331, 5
Neuropteris cf. *gigantea* Sternberg 1982 Cheng Lizhu, pl.331, 6

12. Xiangxiang: Ceshui Formation
<u>*Lepidophyllum* Brongniart, 1828</u>
Lepidophyllum sp. 1953 Sze, pl.9, 12-13
<u>*Rhodeopteridium* (Presl) Zimmermann, 1959</u>
Rhodeopteridium cf. *gigantea* Stur 1953 Sze, pl.9, 10-11

Rhodeopteridium hsianghsiangensis Sze 1954 Sze et Hsü, pl. 4, 9-12; 1963 Zhou, pl. 66, 1, 2; 1974 Gu et Zhi, pl.52, 1-3; 1977 Feng Shaonan et al., pl.240, 6

Rhodeopteridium (Presl) Zimmermann, 1959

Rhodeopteridium hsianghsiangense Sze 1982 Cheng Lizhu, pl.330, 1

Sublepidodendron (Nathorst) Hirmer, 1927 emend. Wang Q, Hao, Wang DM, Wang Y et Thomas Denk, 2003

Sublepidodendron mirabile (Nathorst) Hirmer 1927 1977 Feng Shaonan et al., pl.231, 14; 1982 Cheng Lizhu, pl.324, 7-8

13. Shuangfeng: Ceshui Formation

Sublepidodendron (Nathorst) Hirmer, 1927 emend. Wang Q, Hao, Wang DM, Wang Y et Thomas Denk, 2003

Sublepidodendron mirabile (Nathorst) Hirmer 1927 1982 Cheng Lizhu, pl.324, 7-8

14. Lianyuan and Lengshuijiang: Ceshui Formation

Aneimites (Dawson) Schimper, 1874

Aneimites szei Wu, Lu et Yang, 1986 1986 Wu Xiuyuan et al., pl.II, 2-4

Bothrodendron Lindley et Hutton, 1833

Bothrodendron ruchengense Zhao et Wu emend. 1986 Wu Xiuyuan et al., pl. I, 3-7

Lepidodendropsis Lutz, 1933

Lepidodendropsis sp. 1986 Wu Xiuyuan et al., pl.II, 1

Sphenopteris (Brongniart) Sternberg, 1825

Sphenopteris affinis Lindley et Hutton 1986 Wu Xiuyuan et al., pl.II, 6

Sphenopteris cf. *obtusiloba* Brongniart 1986 Wu Xiuyuan et al., pl.II, 5

15. Shaping Coalmine, Daqiao Commune, Lianyuan County: Ceshui Formation

Mesocalamites Hirmer, 1927

Mesocalamites sp. 1982 Cheng Lizhu, pl.327, 4

16. Qingtangpu Town, Anhua County: Ceshui Formation

Lepidodendropsis Lutz, 1933

Lepidodendropsis hirmeri Lutz 1982 Cheng Lizhu, pl.324, 4

Triphyllopteris Schimper, 1869

Triphyllopteris collombiana Schimper 1982 Cheng, pl. 330, 2, 2a

17. Shaoyang: Ceshui Formation

Lepidodendropsis Lutz, 1933

Lepidodendropsis shaoyangensis? Feng, 1977 1977 Feng Shaonan et al., pl.231, 7; pl.253, 3

18. Ningxiang: Ceshui Formation

Triphyllopteris Schimper, 1869

Triphyllopteris collombiana Schimper 1977 Feng Shaonan et al., pl.239, 6-7

19. Majiaqiao Village, Shitan Town, Xiangtan County: Ceshui Formation

Lepidodendron Sternberg, 1820

Lepidodendron hunanense Cheng, 1982 1982 Cheng Lizhu, pl.325, 8

20. Taoshui Coalmine, Youxian County: Ceshui Formation

Lepidodendron Sternberg, 1820

Lepidodendron taoshuiense Cheng, 1982 1982 Cheng Lizhu, pl.325, 3, 3a; fig.5

21. Shihuichong Coalmine, Youxian County, Zhuzhou City: Ceshui Formation

Cardiopteridium Nathorst, 1914

Cardiopteridium spitsbergense Nathorst 1982 Cheng, pl. 329, 2-7

22. Southern suburb of Zhuzhou City: Ceshui Formation

Stigmaria Brongniart, 1822

Stigmaria ficoides (Sternberg) Brongniart 1982 Cheng Lizhu, pl.326, 4

23. Meitanba Town, Ningxiang County: Ceshui Formation

Cardiopteridium Nathorst, 1914

Cardiopteridium spitsbergense Nathorst 1982 Cheng, pl. 329, 2-7

Rhodeopteridium (Presl) Zimmermann, 1959

Rhodeopteridium sp. 1982 Cheng Lizhu, pl.330, 4, 5

Triphyllopteris Schimper, 1869

Triphyllopteris collombiana Schimper 1982 Cheng, pl. 330, 2, 2a

Guangxi

1. Liucheng: Simen Formation

Mesocalamites Hirmer, 1927

Mesocalamites sp. 1982a Zhao Xiuhu et Wu Xiuyuan, pl.V, 5, 6

2. Liucheng: Yanzi Group

Cardiopteridium Nathorst, 1914

Cardiopteridium spitsbergense Nathorst 1974 Gu et Zhi, pl.41, 13-20; fig.59

Mesocalamites Hirmer, 1927

Mesocalamites sp. 1974 Gu et Zhi, pl.27, 5

Neuropteris (Brongniart) Sternberg, 1825

Neuropteris sp. 1 1974 Gu et Zhi, fig.92

Sphenopteris (Brongniart) Sternberg, 1825

Sphenopteris leei Sze 1977 Feng Shaonan et al., pl.243, 8; 1974 Gu et Zhi, pl.43, 8-10; pl.44, 1-2

3. Dapu Town, Liucheng County: Yanzi Group

Sphenopteris (Brongniart) Sternberg, 1825

Sphenopteris (?Lyginopteris) leei Sze 1953 Sze, pl. 22, 5-6

4. Luocheng: Simen Formation

Calamites Brongniart, 1828

Calamites (?*Mesocalamites*) sp. 1982 Wu et Zhao, p. 142
Neuropteris (Brongniart) Sternberg, 1825
Neuropteris (*Mizoneura*) sp. 1982 Wu et Zhao, p. 142
Sphenopteris (Brongniart) Sternberg, 1825
Sphenopteris(?*Lyginopteris*) *leei* Sze 1982 Wu et Zhao, p. 142

Guangdong

1. **Xiling School and Xiling Factory, Xiaobu Village, Huashan Town, Huadu City: Ceshui Formation**

Archaeocalamites Stur, 1875
Archaeocalamites sp. 1992 Laveine JP et al., pl.2, fig.9-10 ; pl.3, fig.1-2
Bothrodendron Lindley et Hutton, 1833
Bothrodendron circulare Sze 1992 Laveine JP et al., pl.2, fig.6
Cantheliophorus Bassler, 1919
Cantheliophorus sp. 1992 Laveine JP et al., pl.1, fig.9-11
Cardiopteridium Nathorst, 1914
Cardiopteridium cf. *spetsbergerse* Nathorst 1992 Laveine JP et al., pl.7, fig.4-11, 13-14
Eusphenopteris Gothan ex Simson-Scharold, 1934
Eusphenopteris sp. cf. *foliata* Stur 1992 Laveine JP et al., pl.7, fig.2-3
Lepidodendron Sternberg, 1820
Lepidodendron sp. cf. *quadratum* Zhao et Wu 1992 Laveine JP et al., pl.2, fig.1-3
Lepidodendron sp. 1 1992 Laveine JP et al., pl.1, fig.3
Lepidodendron sp. 2 1992 Laveine JP et al., pl.1, fig.5
Lepidostrobophyllum Hirmer, 1927
Lepidostrobophyllum sp. 1 1992 Laveine JP et al., pl.1, fig.6
Lepidostrobophyllum sp. 2 1992 Laveine JP et al., pl.1, fig.7
Lepidostrobophyllum sp. 3 1992 Laveine JP et al., pl.1, fig.8
Palmatopteris Potonié
Palmatopteris subgeniculata (Stur) 1992 Laveine JP et al., pl.6, fig.1-2
Paripteris Gothan, 1941
Paripteris gigantea (Sternberg) 1992 Laveine JP et al., pl.7, fig.12 ; pl.8, fig.3-9 ; pl.9-10
Rhodeopteridium (Presl) Zimmermann, 1959
Rhodeopteridium cf. *chunanense* Wu 1992 Laveine JP et al., pl.4, fig.8
Rhodeopteridium hsianghsiangense Sze 1982 Feng Shaonan et al., p.380
Rhodeopteridium lipoldi Göppert 1992 Laveine JP et al., pl.5, fig.3-6
Rhodeopteridium cf. *parasparsum* Sze 1992 Laveine JP et al., pl.4, fig.5-7
Rhodeopteridium sp. cf. *patentissimum* Ettingshausen 1992 Laveine JP et al., pl.5, fig.2
Rhodeopteridium sp. 1992 Laveine JP et al., pl.4, fig.9-10
Sphenopteris (Brongniart) Sternberg, 1825
Sphenopteris sp. cf. *launoiti* Stockmans et Williere 1992 Laveine JP et al., pl.4, fig.2
Sphenopteris sp. cf. *praecedens* Gothan 1992 Laveine JP et al., pl.4, fig.3-4
Sphenopteris sp. 1 1992 Laveine JP et al., pl.3, fig.5-7
Sphenopteris sp. 2 1992 Laveine JP et al., pl.3, fig.8 ; pl.4, fig.1

Stigmaria Brongniart, 1822

Stigmaria ficoides (Sternberg) Brongniart 1992 Laveine JP et al., pl.1, fig.1

Triphyllopteris Schimper, 1869

?*Triphyllopteris* sp.2 1992 Laveine JP et al., pl.6, fig.7 ; pl.7, fig.1

2. Jiuhu Section, Huadong Town, Huadu City: Ceshui Formation

Archaeocalamites Stur, 1875

Archaeocalamites scrobiculatus Schlotheim ex Seward 2001 Jin Jianhua et Wu Qijun, p. 171

Cardiopteridium Nathorst, 1914

Cardiopteridium spetsbergense Nathorst 2001 Jin Jianhua et Wu Qijun, p. 171

Cordaites Unger, 1850

Cordaites sp. 2001 Jin Jianhua et Wu Qijun, p. 171

Hexagonocarpus Renault, 1890

Hexagonocarpus sp. 2001 Jin Jianhua et Wu Qijun, p. 171

Paripteris Gothan, 1941

Paripteris antecedens 2001 Jin Jianhua et Wu Qijun, p. 171

Paripteris gigantea Sternberg 1992 Laveine JP et al., pl.7, fig.12 ; pl.8, fig.3-9 ; pl.9-10

Potoniea Zeiller, 1899

Potoniea sp. 2001 Jin Jianhua et Wu Qijun, p. 171

Sphenophyllum Brongniart, 1828

Sphenophyllum tenerrimum Ettingshausen 1992 Laveine JP et al., pl.3, fig.3

Stigmaria Brongniart, 1822

Stigmaria ficoides (Sternberg) Brongniart 2001 Jin Jianhua et Wu Qijun, p. 171

Sublepidodendron (Nathorst) Hirmer, 1927 emend. Wang Q, Hao, Wang DM, Wang Y et Thomas Denk, 2003

Sublepidodendron grabaui (Sze) Wang et Xu, 2005 2001 Jin Jianhua et Wu Qijun, p. 171

Triphyllopteris Schimper, 1869

Triphyllopteris sp. 2001 Jin Jianhua et Wu Qijun, p. 171

3. Shaoguan: Furongshan Formation

Cardiopteridium Nathorst, 1914

Cardiopteridium spitsbergense Nathorst 1982a Zhao Xiuhu et Wu Xiuyuan, pl.VI, 15-19; fig.3

Cardiopteridium podozamioides (Sze) Zhao et Wu, 1982 1982a Zhao Xiuhu et Wu Xiuyuan, pl. VI, 3-14, 7a; ?20-21; pl. VII, 2; fig.4

Carpolithus Brongniart, 1822

Carpolithus perpusillus Lesquereux 1982a Zhao Xiuhu et Wu Xiuyuan, pl. XI, 13-14, 14a

Carpolithus sp. 1982a Zhao Xiuhu et Wu Xiuyuan, pl. XII, 1-3, 20, 1a, 3a

Codonospermum Brongniart

Codonospermum sp. 1982a Zhao Xiuhu et Wu Xiuyuan, pl. XI, 11, 11a; 1992 Li Xingxue et al., pl. I, 12

Potoniea Zeiller, 1899

Potoniea racemicarpa Feng et al. 1992 Li Xingxue et al., pl. III, 9, 10

Sublepidodendron (Nathorst) Hirmer, 1927 emend. Wang Q, Hao, Wang DM, Wang Y et Thomas Denk, 2003

Sublepidodendron mirabile (Nathorst) Hirmer 1927 1977 Feng Shaonan et al., pl.231, 14

4. Shaoguan: Furongshan Formation

Cardiopteridium Nathorst, 1914

Cardiopteridium spetsbergense Nathorst 1982a Zhao Xiuhu et Wu Xiuyuan, pl.VI, 15-19; fig.3

Mesocalamites Hirmer, 1927

Mesocalamites sp. 1982a Zhao Xiuhu et Wu Xiuyuan, pl.V, 5, 6

Neuropteris (Brongniart) Sternberg, 1825

Neuropteris cf. *antecedens* Stur 1982a Zhao Xiuhu et Wu Xiuyuan, pl.VII, 5-9, 9a; fig.6

Rhodeopteridium (Presl) Zimmermann, 1959

Rhodeopteridium hsianghsiangense Sze 1982a Zhao Xiuhu et Wu Xiuyuan, pl.IX, 5; pl.X, 1

Sphenopteris (Brongniart) Sternberg, 1825

Sphenopteris leei Sze 1982a Zhao Xiuhu et Wu Xiuyuan, pl.XI, 1, 1a, ?2-3, 2a-3a

5. Shaoguan: Ceshui Formation

Adiantites Göppert, 1836

Adiantites sp. 1982 Feng Shaonan et al., p.380

Diplotmema adiantoides Schlotheim ex Gothan 1982 Feng Shaonan et al., p.380

Rhodeopteridium (Presl) Zimmermann, 1959

Rhodeopteridium hsianghsiangense Sze 1982 Feng Shaonan et al., p.380

Stigmaria Brongniart, 1822

Stigmaria rugulosa 1982 Feng Shaonan et al., p.380

6. Shaoguan: Ceshui Formation

Archaeocalamites Stur, 1875

Archaeocalamites scrobiculatus Schlotheim ex Seward 1982 Feng Shaonan et al., p.380

Archaeopteridium Kidston, 1923

Archaeopteridium shaoguanense? Feng et al., 1982 1982 Feng Shaonan et al., pl.II, 8-10

Lepidodendron Sternberg, 1820

Lepidodendron sp. 1982 Feng Shaonan et al., p.380

Lepidodendron sophoroides? Feng et al., 1982 1982 Feng Shaonan et al., pl.I, 2

Neuropteris (Brongniart) Sternberg, 1825

Neuropteris kaipingiana Sze 1982 Feng Shaonan et al., p.380

Neuropteris shaoguanensis? Feng et al., 1982 1982 Feng Shaonan et al., pl.I, 3

Neuropteris sp.4 1982 Feng Shaonan et al., p.380

Neuropteris sp.5 1982 Feng Shaonan et al., p.380

Potoniea Zeiller, 1899

Potoniea racemicarpa Feng et al., 1982 1982 Feng Shaonan et al., pl.II, 1a, 1b

Rhacopteris Schimper, 1869

Rhacopteris sp. 1982 Feng Shaonan et al., p.380

Rhodeopteridium (Presl) Zimmermann, 1959

Rhodeopteridium hsianghsiangense Sze 1982 Feng Shaonan et al., p.380

Taeniopteris Brongniart, 1828

Taeniopteris? sp. 1982 Feng Shaonan et al., p.380

7. Shaoguan: Middle Zhongxin Formation

Sphenopteris (Brongniart) Sternberg, 1825

Sphenopteris obtustloba 1982 Feng Shaonan et al., p.381

8. Qujiang District, Shaoguan City: Ceshui Formation

Rhodeopteridium (Presl) Zimmermann, 1959

Rhodeopteridium hsianghsiangensis Sze 1963 Zhou, pl. 66, 1, 2; 1977 Feng Shaonan et al., pl.240, 6

Sphenopteris (Brongniart) Sternberg, 1825

Sphenopteris obtustloba 1977 Feng Shaonan et al., pl. 241, 3

9. Yingde: Datang Stage

Lepidodendron Sternberg, 1820

Lepidodendron shanyangense Wu et He 1982a Zhao et Wu, pl.II, 4-6, 4a, 6a; pl.III, 1-5, 2a-5a; pl.IV, 4, 4a

Lepidodendron wengyuanense Zhao et Wu 1982 1982a Zhao Xiuhu et Wu Xiuyuan, pl.IV, 3, 3a

Neuropteris (Brongniart) Sternberg, 1825

Neuropteris sp.1 1982a Zhao Xiuhu et Wu Xiuyuan, pl.XII, 16-18

Rhodeopteridium (Presl) Zimmermann, 1959

Rhodeopteridium yingdeense Zhao et Wu, 1982 1982a Zhao Xiuhu et Wu Xiuyuan, pl.IX, 4; pl. X, 2-6

Rhodeopteridium sp.1 1982a Zhao Xiuhu et Wu Xiuyuan, pl.X, 11

Sphenopteris (Brongniart) Sternberg, 1825

Sphenopteris sp.1 1982a Zhao Xiuhu et Wu Xiuyuan, pl.XI, 4

Sphenopteris sp.2 1982a Zhao Xiuhu et Wu Xiuyuan, pl.XI, 10

10. Lianping County, Heyuan City: Upper Zhongxin Formation

Adiantites Göppert, 1836

Adiantites lianpingensis? Feng et al., 1982 1982 Feng Shaonan et al., pl.I, 4

Archaeopteridium Kidston, 1923

Archaeopteridium orientale Zhao et Wu, 1982 1982a Zhao Xiuhu et Wu Xiuyuan, pl.VIII, 1-5; pl.IX, 1-3, 3a

Cardiopteridium Nathorst, 1914

Cardiopteridium podozamioides (Sze) Zhao et Wu, 1982 1982a Zhao Xiuhu et Wu Xiuyuan, pl.VI, 3-14, 7a; ?20-21; pl.VII, 2; fig.4

Cardiopteridium spitsbergense Nathorst 1982a Zhao Xiuhu et Wu Xiuyuan, pl.VI, 15-19; fig.3

Mariopteris Zeiller, 1879

Mariopteris acuta cf. *obtusa* 1982 Feng Shaonan et al., p.381

Neuropteris (Brongniart) Sternberg, 1825

Neuropteris gigantea Sternberg 1982a Zhao Xiuhu et Wu Xiuyuan, pl.XII, 8-15; 1982 Feng Shaonan et al., p.381

Neuropteris sp.2 1982a Zhao Xiuhu et Wu Xiuyuan, pl.XII, 19; 1982 Feng Shaonan et al., p.381

Potoniea Zeiller, 1899

Potoniea turbinata Feng et al. 1982 1982 Feng Shaonan et al., pl.II, 3a, 3b

Rhodeopteridium (Presl) Zimmermann, 1959

Rhodeopteridium cf. *bifidum* (L. et H.) Oberste Brink 1982a Zhao Xiuhu et Wu Xiuyuan, pl.XII, 5-6

Rhodeopteridium hsianghsiangense Sze 1982a Zhao Xiuhu et Wu Xiuyuan, pl.IX, 5; pl.X, 1

Rhodeopteridium lianpingense 1982 1982a Zhao Xiuhu et Wu Xiuyuan, pl.X, 7-10

Sphenopteris (Brongniart) Sternberg, 1825

Sphenopteris subsulcata Zhao et Wu, 1982 1982a Zhao Xiuhu et Wu Xiuyuan, pl.XI, 6-9, 6a-8a

Paripteris Gothan, 1941

Paripteris cardiopteroides Bohlin 1992 Li Xingxue et al., pl. II, 5-7

Samaropsis Göppert, 1864

Samaropsis sp. 1982 Feng Shaonan et al., p.381

Sphenopteris (Brongniart) Sternberg, 1825

Sphenopteris leei Sze 1982 Feng Shaonan et al., p.381

Sphenopteris obtustloba 1982 Feng Shaonan et al., p.381

11. Lianping County, Heyuan City: Middle Zhongxin Formation

Potoniea Zeiller, 1899

Potoniea turbinata Feng et al. 1992 Li Xingxue et al., pl. II, 17, 18

12. Lianping County, Heyuan City: Ceshui Formation

*Pecopteris (*Brongniart) Sternberg, 1825

Pecopteris sp.1 1982 Feng Shaonan et al., p.380

Sphenopteris (Brongniart) Sternberg, 1825

Sphenopteris obtustloba 1982 Feng Shaonan et al., p.380

13. Heyuan City: Ceshui Formation

Adiantites Göppert, 1836

Adiantites sp. 1977 Feng Shaonan et al., pl.241, 4

14. Lianping County, Heyuan City: Dahu Formation

Archaeocalamites Stur, 1875

Archaeocalamites sp. 1982 Feng Shaonan et al., p.380

Lepidodendropsis Lutz, 1933

Lepidodendropsis sp. 1982 Feng Shaonan et al., p.380

Sublepidodendron (Nathorst) Hirmer, 1927 emend. Wang Q, Hao, Wang DM, Wang Y et Thomas Denk, 2003

Sublepidodendron mirabile (Nathorst) Hirmer 1927 1982 Feng Shaonan et al., p.380

15. Zhaoqing: Ceshui Formation

Adiantites Göppert, 1836

Adiantites gothani 1982 Feng Shaonan et al., p.380

Cardiopteris Schimper, 1869

Cardiopteris? sp. 1982 Feng Shaonan et al., p.380

Rhacopteris Schimper, 1869

Rhacopteris ovata (McCoy) Walkom, 1934 1982 Feng Shaonan et al., p.380

16. Zhaoqing: Ceshui Formation

Rhodeites Nemejc, 1936

Rhodeites? *lanceolata* Feng et al., 1982 1982 Feng Shaonan et al., pl.I, 7

17. Xinfengjiang River: Ceshui Formation

Adiantites Göppert, 1836

Adiantites matouensis Feng et al., 1982 1982 Feng Shaonan et al., pl.I, 9a

Mariopteris Zeiller, 1879

Mariopteris? sp. 1982 Feng Shaonan et al., p.380

18. Wengyuan: Ceshui Formation

Rhodeopteridium (Presl) Zimmermann, 1959

Rhodeopteridium hsianghsiangense Sze 1982 Feng Shaonan et al., p.380

19. Wengyuan: Ceshui Formation

Neuropteris (Brongniart) Sternberg, 1825

Neuropteris pseudogigantea Potonié 1982 Feng Shaonan et al., p.380

Neuropteris sp.1 1982 Feng Shaonan et al., p.380

Neuropteris sp.3 1982 Feng Shaonan et al., p.380

Rhacopteris Schimper, 1869

Rhacopteris angusta? Feng et al., 1982 1982 Feng Shaonan et al., pl.II, 2

20. Wengyuan: Dahu Formation

Sublepidodendron (Nathorst) Hirmer, 1927 emend. Wang Q, Hao, Wang DM, Wang Y et Thomas Denk, 2003

Sublepidodendron wengtanense? Feng et al. 1982 1982 Feng Shaonan et al., pl.I, 1

21. Ruyuan: Ceshui Formation

Pecopteris (Brongniart) Sternberg, 1825

Pecopteris sp.2 1982 Feng Shaonan et al., p.380

22. North Guangdong: Ceshui Formation

Sphenopteris (Brongniart) Sternberg, 1825

Sphenopteris sp.1 1953 Sze, pl. 22, 1-2

23. Caochanggang–Gangwei Section, Nanhai City: Lower Ceshui Formation

Adiantites Göppert, 1836

Adiantites gothani (Sze) Zhang, Zhao et Wu 2001 Jin Jianhua et Wu Qijun, p. 169

Archaeocalamites Stur, 1875

Archaeocalamites scrobiculatus Schlotheim ex Seward 2001 Jin Jianhua et Wu Qijun, p. 169

Cardiopteridium Nathorst, 1914

Cardiopteridium spitsbergense Nathorst 2001 Jin Jianhua et Wu Qijun, p. 169

Mesocalamites Hirmer, 1927

Mesocalamites sp. 2001 Jin Jianhua et Wu Qijun, p. 169

Rhodeopteridium (Presl) Zimmermann, 1959

Rhodeopteridium hsianghsiangense Sze 2001 Jin Jianhua et Wu Qijun, p. 168

Sphenopteris (Brongniart) Sternberg, 1825

Sphenopteris obtusiloba Brongniart 2001 Jin Jianhua et Wu Qijun, p. 169

Sphenopteris leei Sze 2001 Jin Jianhua et Wu Qijun, p. 169
Stigmaria Brongniart, 1822
Stigmaria ficoides (Sternberg) Brongniart 2001 Jin Jianhua et Wu Qijun, p. 169
Triphyllopteris Schimper, 1869
Triphyllopteris collombiana Schimper 2001 Jin Jianhua et Wu Qijun, p. 169

24. Shima Section, Guangzhou City: Upper Ceshui Formation
Archaeocalamites Stur, 1875
Archaeocalamites scrobiculatus Schlotheim ex Seward 2001 Jin Jianhua et Wu Qijun, p. 169
Cardiopteridium Nathorst, 1914
Cardiopteridium podozamioides (Sze) Zhao et Wu, 1982 2001 Jin Jianhua et Wu Qijun, p. 169
Cardiopteridium spitsbergense Nathorst 2001 Jin Jianhua et Wu Qijun, p. 169
Cordaites Unger, 1850
Cordaites sp. 2001 Jin Jianhua et Wu Qijun, p. 169
Rhodeopteridium (Presl) Zimmermann, 1959
Rhodeopteridium hsianghsiangense Sze 2001 Jin Jianhua et Wu Qijun, p. 169
Rhodeopteridium sp. 2001 Jin Jianhua et Wu Qijun, p. 169
Paripteris Gothan, 1941
Paripteris gigantea (Sternberg) Gothan 2001 Jin Jianhua et Wu Qijun, p. 169
Paripteris antecedens 2001 Jin Jianhua et Wu Qijun, p. 169
Sphenopteris (Brongniart) Sternberg, 1825
Sphenopteris affinis Lindley et Hutton 2001 Jin Jianhua et Wu Qijun, p. 169

25. Fenggang Section, Guangzhou City: Upper Ceshui Formation
Cardiopteridium Nathorst, 1914
Cardiopteridium podozamioides (Sze) Zhao et Wu, 1982 2001 Jin Jianhua et Wu Qijun, p. 170
Cardiopteridium spitsbergense Nathorst 2001 Jin Jianhua et Wu Qijun, p. 170
Cordaites Unger, 1850
Cordaites sp. 2001 Jin Jianhua et Wu Qijun, p. 170
Lepidodendron Sternberg, 1820
Lepidodendron shanyangense Wu et He 2001 Jin Jianhua et Wu Qijun, p. 170
Paripteris Gothan, 1941
Paripteris gigantea (Sternberg) Gothan 2001 Jin Jianhua et Wu Qijun, p. 170
Triphyllopteris Schimper, 1869
Triphyllopteris collombiana Schimper 2001 Jin Jianhua et Wu Qijun, p. 169

26. Near Chini Cement Factory, Huadu City: Ceshui Formation
Archaeocalamites Stur, 1875
Archaeocalamites scrobiculatus Schlotheim ex Seward 2001 Jin Jianhua et Wu Qijun, p. 169
Cardiopteridium Nathorst, 1914
Cardiopteridium spetsbergense Nathorst 2001 Jin Jianhua et Wu Qijun, p. 170
Lepidodendron Sternberg, 1820
Lepidodendron jiandeense Zhao et Chen, 1986 2001 Jin Jianhua et Wu Qijun, p. 170
Lepidodendron quadratum Zhao et Wu, 1982 2001 Jin Jianhua et Wu Qijun, p. 170

Lepidodendron shanyangense Wu et He 2001 Jin Jianhua et Wu Qijun, p. 170

Lepidodendron sp. 2001 Jin Jianhua et Wu Qijun, p. 170

Paripteris Gothan, 1941

Paripteris gigantea (Sternberg) Gothan 2001 Jin Jianhua et Wu Qijun, p. 170

Rhodeopteridium (Presl) Zimmermann, 1959

Rhodeopteridium hsianghsiangense Sze 2001 Jin Jianhua et Wu Qijun, p. 169

27. The 2nd Mine of Chini Feishuyan, Huadu City: Ceshui Formation

No plant record

Guizhou

1. Guiyang: Datang Stage

Anisopteris (Oberste-Brink) Hirmer

Anisopteris cf. *transitionis* (Stur) Hirmer 1982a Zhao Xiuhu et Wu Xiuyuan, pl.VIII, 6, 6a

2. Shuiyan Town, Dushan County: Jiusi Formation

Rhodeopteridium (Presl) Zimmermann, 1959

Rhodeopteridium tenuis Gothan 1978 Zhang Jihui, pl.153, 1

Sublepidodendron (Nathorst) Hirmer, 1927 emend. Wang Q, Hao, Wang DM, Wang Y et Thomas Denk, 2003

Sublepidodendron cf. *mirabile* (Nathorst) Hirmer, 1927 1978 Zhang Jihui, pl.150, 5

3. Shanping Coalmine, Shanping Village, Huaxi District, Guiyang City: Jiusi Formation

Lepidodendron Sternberg, 1820

Lepidodendron cf. *canobianum* Crookall 1978 Zhang Jihui, pl.150, 9

4. Western–Middle Guizhou: Wanshoushan Formation

Lepidodendron Sternberg, 1820

Lepidodendron rimosum Sternberg 1982 Wu et Zhao, p. 141

Lepidodendron shanyangense Wu et He 1982 Wu et Zhao, p. 141

Lepidostrobophyllum Hirmer, 1927

Lepidostrobophyllum sp. 1982 Wu et Zhao, p. 141

Sublepidodendron (Nathorst) Hirmer, 1927 emend. Wang Q, Hao, Wang DM, Wang Y et Thomas Denk, 2003

Sublepidodendron cf. *mirabile* (Nathorst) Hirmer, 1927 1982 Wu et Zhao, p. 141

Zhejiang

1. Changxing: Gaolishan Formation or/and Hezhou Formation

Sphenophyllum Brongniart, 1828

Sphenophyllum sp. 1982 Wu et Zhao, p. 140

Stigmaria Brongniart, 1822

Stigmaria sp. 1982 Wu et Zhao, p. 140

<u>*Sublepidodendron* (Nathorst) Hirmer, 1927 emend. Wang Q, Hao, Wang DM, Wang Y et Thomas Denk, 2003</u>

Sublepidodendron mirabile (Nathorst) Hirmer, 1927 1982 Wu et Zhao, p. 140

2. Xiangudong, Lijia Town, Jiande County: lower Yejiatang Group

<u>*Archaeocalamites* Stur, 1875</u>

Archaeocalamites scrobiculatus Schlotheim ex Seward 1987 Chen Qishi, pl.4, 5

<u>*Hexagonocarpus* Renault, 1890</u>

Hexagonocarpus ellipticus Zhao et al., 1986 1986 Zhao Xiuhu et al., pl.XV, 5, 6

<u>*Neuropteris* (Brongniart) Sternberg, 1825</u>

Neuropteris sp. 1986 Zhao Xiuhu et al., p. 4

<u>*Sphenopteris* (Brongniart) Sternberg, 1825</u>

Sphenopteris sp. 1986 Zhao Xiuhu et al., p. 4

3. Shimatou Coal Mine, Xin'anjiang Town, Jiande County: lower Yejiatang Group

<u>*Sphenophyllum* Brongniart, 1828</u>

Sphenophyllum tenerrimum Ettingshausen 1986 Zhao Xiuhu et al., pl. V, 3-5

4. Tianfan Village, Datong Town, Jiande County: lower Yejiatang Group

<u>*Adiantites* Göppert, 1836</u>

Adiantites gothani (Sze) Zhang, Zhao et Wu 1986 Zhao Xiuhu et al., pl.VI, 3, 4, 3a, 4a

5. Qiuchuan Town, Changshan County: lower Yejiatang Group

<u>*Anisopteris* (Oberste-Brink) Hirmer</u>

Anisopteris cf. *transitionis* (Stur) Hirmer 1986 Zhao Xiuhu et al., pl.VI, 2, 2a

<u>*Neuropteris* (Brongniart) Sternberg, 1825</u>

Neuropteris otozamioides Sze et Lee 1986 Zhao Xiuhu et al., pl.XIII, 7, 8, 7a

<u>*Sphenophyllum* Brongniart, 1828</u>

Sphenophyllum tenerrimum Ettingshausen 1986 Zhao Xiuhu et al., pl. V, 3-5

<u>*Sublepidodendron* (Nathorst) Hirmer, 1927 emend. Wang Q, Hao, Wang DM, Wang Y et Thomas Denk, 2003</u>

Sublepidodendron sp. 1987 Chen Qishi, pl.5, 2

6. Baima Town, Chun'an County, Hangzhou City: lower Yejiatang Group

<u>*Adiantites* Göppert, 1836</u>

Adiantites sp. 1986 Zhao Xiuhu et al., p. 5

<u>*Aneimites* (Dawson) Schimper, 1874</u>

Aneimites dichotomous Chen 1982 1982 Li Hanmin et al., pl.134, 7; 1987 Chen, pl. V, 8

<u>*Neuropteris* (Brongniart) Sternberg, 1825</u>

Neuropteris sp. 1986 Zhao Xiuhu et al., p. 5

<u>*Rhodeopteridium* (Presl) Zimmermann, 1959</u>

Rhodeopteridium chunanense Wu 1986 1987 Chen, pl. IV, 11

Rhodeopteridium cf. *hsianghsiangense* Sze 1986 Zhao Xiuhu et al., pl.VIII, 1, 2

<u>*Sphenophyllum* Brongniart, 1828</u>

Sphenophyllum tenerrimum Ettingshausen 1986 Zhao Xiuhu et al., pl. V, 3-5

7. Yejiatang Section, Fengjia Town, Kaihua County: lower Yejiatang Group

Adiantites Göppert, 1836

Adiantites gothani (Sze) Zhang, Zhao et Wu 1986 Zhao Xiuhu et al., pl.VI, 3, 4, 3a, 4a; 1987 Chen, pl. IV, 7

Adiantites sp.2 1986 Zhao Xiuhu et al., pl.VII, 5

Archaeocalamites Stur, 1875

Archaeocalamites scrobiculatus Schlotheim ex Seward 1986 Zhao Xiuhu et al., pl. IV, 5, 5a

Cardiopteridium Nathorst, 1914

Cardiopteridium sp. 1987 Chen, pl. IV, 8-9

Neuropteris (Brongniart) Sternberg, 1825

Neuropteris sp. 1986 Zhao Xiuhu et al., p. 4

Rhacopteris Schimper, 1869

Rhacopteris kaihuaensis Zhao 1986 Zhao Xiuhu et al., pl. VI, 5, 5a; 1987 Chen Qishi, pl.4, 6

Rhodeopteridium (Presl) Zimmermann, 1959

Rhodeopteridium chunanense Wu, 1986 1987 Chen, pl. IV, 11

Rhodeopteridium cf. *hsianghsiangense* Sze 1986 Zhao Xiuhu et al., pl.VIII, 1, 2

Rhodeopteridium tenuis Gothan 1986 Zhao Xiuhu et al., pl.VIII, 4, 5

Rhodeopteridium cf. *nematophyllum* Daber. 1986 Zhao Xiuhu et al., pl.VIII, 3; pl. XI, 4

Sphenophyllum Brongniart, 1828

Sphenophyllum tenerrimum Ettingshausen 1986 Zhao Xiuhu et al., pl. V, 3-5; 1987 Chen, pl. IV, 10

8. Hejiashan Town, Jiangshan County: lower Yejiatang Group

Hexagonocarpus Renault, 1890

Hexagonocarpus ellipticus Zhao et al., 1986 1986 Zhao Xiuhu et al., pl.XV, 5, 6

9. Zhucangwu Section, Linghu Village, Changshan County: Lower Yejiatang Group

Jiangxi

1. Tizikou Section, Maohushang Village, Yidu County: Gaolishan Formation

Sphenopteris (Brongniart) Sternberg, 1825

Sphenopteris obtusiloba Brongniart 1982 Li Hanmin et al., pl.138, 5

2. Folingwei Section, Fengcheng City: Zhanggongmiao Formation

Archaeocalamites Stur, 1875

Archaeocalamites scrobiculatus (Schlotheim) Seward 1982 Wu et Zhao, p. 142

Asterocalamites Schimper ex Zeiller, 1879

Asterocalamites sp. 1964 Zhang Caifan et Liu Yaguang, p. 372

Cardiopteridium Nathorst, 1914

Cardiopteridium spitsbergense Nathorst 1982 Wu et Zhao, p. 142

Cordaites Unger, 1850

Cordaites sp. 1964 Zhang Caifan et Liu Yaguang, p. 372

Knorria Sternberg, 1825

Knorria sp. 1964 Zhang Caifan et Liu Yaguang, p. 372

Rhodeopteridium (Presl) Zimmermann, 1959

Rhodeopteridium sp. 1964 Zhang Caifan et Liu Yaguang, p. 372

Rhodeopteridium (Presl) Zimmermann, 1959

Rhodeopteridium lipoldi (Stur) Patteisky 1982 Wu et Zhao, p. 142

Rhodeopteridium tenuis (Gothan) Zimmermann 1982 Wu et Zhao, p. 142

Rhodeopteridium sp. 1982 Wu et Zhao, p. 142

Rhodeopteridium cf. *hsianghsiangense* Sze 1982 Wu et Zhao, p. 142

Sphenophyllum Brongniart, 1828

Sphenophyllum cuneifolium Sze 1982 Wu et Zhao, p. 142

Sublepidodendron sp. 1964 Zhang Caifan et Liu Yaguang, p. 372

3. Huashanling Section, Fengcheng City: Zhanggongmiao Formation

Adiantites Göppert, 1836

Adiantites sp. 1964 Zhang Caifan et Liuyaguang, p. 373

Calamites Brongniart, 1828

Calamites sp. 1964 Zhang Caifan et Liuyaguang, p. 373

Mesocalamites Hirmer, 1927

Mesocalamites sp. 1964 Zhang Caifan et Liuyaguang, p. 373

Rhodeopteridium (Presl) Zimmermann, 1959

Rhodeopteridium cf. *hsianghsiangensis* Sze 1964 Zhang Caifan et Liuyaguang, p. 373

Sphenopteris (Brongniart) Sternberg, 1825

Sphenopteris sp. 1964 Zhang Caifan et Liuyaguang, p. 373

4. Yuyi Section, Fuzhou City: Zhanggongmiao Formation

Asterocalamites Schimper ex Zeiller, 1879

Asterocalamites scrobiculatus Schlotheim ex Zeiller 1964 Zhang Caifan et Liuyaguang, p. 373

Cardiopteridium Nathorst, 1914

Cardiopteridium sp. 1964 Zhang Caifan et Liuyaguang, p. 373

Cordaites Unger, 1850

Cordaites sp. 1964 Zhang Caifan et Liuyaguang, p. 373

Neuropteris (Brongniart) Sternberg, 1825

Neuropteris gigantea Sternberg 1964 Zhang Caifan et Liuyaguang, p. 373

5. Da'ao Section, Yundu County: Zhanggongmiao Formation

Adiantites Göppert, 1836

Adiantites ungeri Reed 1964 Zhang Caifan et Liuyaguang, p. 375

Cardiopteridium Nathorst, 1914

Cardiopteridium cf. *spitsbergense* Nathorst 1964 Zhang Caifan et Liuyaguang, p. 375

Yunnan

1. Xintun Village, Yanfang Town, Zhanyi County

Lepidodendron Sternberg, 1820

Lepidodendron sp. 1927 Halle T G, pl.1, fig.4-5

2. Longhuashan, Zhanyi County

Lepidodendron Sternberg, 1820

Lepidodendron sp. 1927 Halle T G, pl.1, fig.4-5

3. Eastern Yunnan: Wanshoushan Formation

Lepidodendron Sternberg, 1820

Lepidodendron rimosum Sternberg 1982 Wu et Zhao, p. 141

Lepidodendron shanyangense 1982 Wu et Zhao, p. 141

Lepidostrobophyllum Hirmer, 1927

Lepidostrobophyllum sp. 1982 Wu et Zhao, p. 141

Sublepidodendron (Nathorst) Hirmer, 1927 emend. Wang Q, Hao, Wang DM, Wang Y et Thomas Denk, 2003

Sublepidodendron cf. *mirabile* (Nathorst) Hirmer, 1927 1982 Wu et Zhao, p. 141

Tibet (Xizang)

Machala Section, Leiwuqi County, Changdu: Machala Formation

Archaeocalamites Stur, 1875

Archaeocalamites scrobiculatus Schlotheim ex Seward 1982 Wu et Zhao, p. 140

Cardiopteridium Nathorst, 1914

Cardiopteridium spitsbergense Nathorst 1978 Deng Longhua, pl. I, 9-12; 1982 Wu et Zhao, p. 140

Lepidodendron Sternberg, 1820

Lepidodendron machalaensis Deng 1978 1978 Deng Longhua, pl. I, 1-4; 1982 Wu et Zhao, p. 140

Rhodeopteridium (Presl) Zimmermann, 1959

Rhodeopteridium tenuis Gothan 1978 Deng Longhua, pl. I, 14-17; 1982 Wu et Zhao, p. 140

Sphenophyllum Brongniart, 1828

Sphenophyllum tenerrimum Ettingshausen 1982 Wu et Zhao, p. 140

Sphenopteris (Brongniart) Sternberg, 1825

Sphenopteris changduensis Gu et Zhi 1974 Gu et Zhi, pl. 50, 4-8; 1978 Deng Longhua, pl. I, 5-8, 13; 1982 Wu et Zhao, p. 140

Stigmaria Brongniart, 1822

Stigmaria sp. 1978 Deng Longhua, pl. I, 18; 1982 Wu et Zhao, p. 140

Sublepidodendron (Nathorst) Hirmer, 1927 emend. Wang Q, Hao, Wang DM, Wang Y et Thomas Denk, 2003

Sublepidodendron changduense Gu et Zhi 1974 Gu et Zhi, pl. 6, 5-8

Triphyllopteris Schimper, 1869

Triphyllopteris collombiana Schimper 1982 Wu et Zhao, p. 140

Jiangsu

1. Yixing: Gaolishan Formation

Eolepidodendron Wu et Zhao, 1981

Eolepidodendron nathorsti (Kidston) Wu 1982 Li Hanmin et al., pl.129, 6-7

<u>*Sublepidodendron* (Nathorst) Hirmer, 1927 emend. Wang Q, Hao, Wang DM, Wang Y et Thomas Denk, 2003</u>
Sublepidodendron tangshanense Wu, 1982 1982 Li Hanmin et al., pl.131, 1-3

2. Jurong: Gaolishan Formation
<u>*Archaeocalamites* Stur, 1875</u>
Archaeocalamites sp. 1982 Li Hanmin et al., pl.132, 2
<u>*Knorria* Sternberg, 1825</u>
Knorria sp. 1987 Yan Youyin, p. 14
<u>*Lepidodendron* Sternberg, 1820</u>
Lepidodendron gaolishanense Wu et Zhao, 1981 192 Li et al., pl. 134, 2-3
<u>*Lopinopteris* Sze, 1958</u>
Lopinopteris intercalata (Sze) Gu et Zhi 1982 Li Hanmin et al., pl.152, 1-4
<u>*Stigmaria* Brongniart, 1822</u>
Stigmaria ficoides (Sternberg) Brongniart 1987 Yan Youyin, p. 14
Stigmaria rugulosa Gothan 1987 Yan Youyin, p. 14
<u>*Sublepidodendron* (Nathorst) Hirmer, 1927 emend. Wang Q, Hao, Wang DM, Wang Y et Thomas Denk, 2003</u>
Sublepidodendron mirabile (Nathorst) Hirmer, 1927 1982 Li Hanmin et al., pl.131, 6-7

3. Gaolishan Section, Gaozi Town, Jurong County: Gaolishan Formation
<u>*Archaeocalamites* Stur, 1875</u>
Archaeocalamites sp. 1981 Wu Xiuyuan et Zhao Xiuhu, pl.II，1-3
<u>*Eolepidodendron* Wu et Zhao, 1981</u>
Eolepidodendron jurongense Wu et Zhao, 1981 1981 Wu et Zhao, pl.I, 5, 5a
Eolepidodendron cf. *wusihense* (Sze) Wu et Zhao, 1981 1981 Wu Xiuyuan et Zhao Xiuhu, pl.I, 7
Eolepidodendron sp. 1981 Wu Xiuyuan et Zhao Xiuhu, pl.I, 6
<u>*Hamatophyton* Gu et Zhi, 1974 emend. Li, Cai et Wang, 1995</u>
Hamatophyton verticillatum Gu et Zhi, 1974 emend. Li, Cai et Wang, 1995 1981 Wu Xiuyuan et Zhao Xiuhu, pl.II, 4-7, 7a
<u>*Lepidodendron* Sternberg, 1820</u>
Lepidodendron gaolishanense Wu et Zhao, 1981 1981 Wu et Zhao, pl.I, 8, 8a, 9, 10, 10a, 11, 12
<u>*Rhodeopteridium* (Presl) Zimmermann, 1959</u>
Rhodeopteridium cf. *hsianghsiangense* Sze 1981 Wu Xiuyuan et Zhao Xiuhu, pl.II, 8, 8a, 9, 10
Rhodeopteridium sp. 1981 Wu Xiuyuan et Zhao Xiuhu, pl.II, 11, 12; pl.III, 1, 1a
<u>*Sublepidodendron* (Nathorst) Hirmer, 1927 emend. Wang Q, Hao, Wang DM, Wang Y et Thomas Denk, 2003</u>
Sublepidodendron cf. *mirabile* (Nathorst) Hirmer, 1927 1981 Wu Xiuyuan et Zhao Xiuhu, pl.I, 3, 4
<u>*Telangium* Benson, 1904</u>
Telangium sp. 1981 Wu Xiuyuan et Zhao Xiuhu, pl. III, 2-5, 2a-5a

4. Jiangning: Gaolishan Formation
<u>*Rhodeopteridium* (Presl) Zimmermann, 1959</u>
Rhodeopteridium jiangningense Zhao et Wu, 1982 1982 Li Hanmin et al., pl.141, 2-3

Sublepidodendron (Nathorst) Hirmer, 1927 emend. Wang Q, Hao, Wang DM, Wang Y et Thomas Denk, 2003

Sublepidodendron tangshanense Wu, 1982 1982 Li Hanmin et al., pl.131, 1-3

5. Longtan: Gaolishan Formation

Sublepidodendron (Nathorst) Hirmer, 1927 emend. Wang Q, Hao, Wang DM, Wang Y et Thomas Denk, 2003

Sublepidodendron mirabile (Nathorst) Hirmer, 1927 1974 Gu et Zhi, pl.5, 9-11

Anhui

1. Chaoxian: Gaolishan Formation

Cardiopteridium Nathorst, 1914

Cardiopteridium spitsbergense Nathorst 1982 Wu et Zhao, p. 139

2. Guangde: Gaolishan Formation (or, and Hezhou Formation)

Sphenophyllum Brongniart, 1828

Sphenophyllum sp. 1982 Wu et Zhao, p. 140

Stigmaria Brongniart, 1822

Stigmaria sp. 1982 Wu et Zhao, p. 140

Sublepidodendron (Nathorst) Hirmer, 1927 emend. Wang Q, Hao, Wang DM, Wang Y et Thomas Denk, 2003

Sublepidodendron mirabile (Nathorst) Hirmer, 1927 1982 Wu et Zhao, p. 140

Hainan

Shilu Iron Mine, Changjiang County: Sanlengshan Formation

Cordaites Unger, 1850

Cordaites sp. 1992 Feng Shaonan, pl. 7, 5

Mariopteris Zeiller, 1879

Mariopteris acuta Brongniart f. *obtusa* Gothan 1992 Feng Shaonan, pl. 7, 1a

?*Mariopteris* sp. 1982 Wu et Zhao, p. 143

Neuropteris (Brongniart) Sternberg, 1825

Neuropteris sp. 1992 Feng Shaonan, pl. 7, 6

Neuropteris sp. 1 1992 Feng Shaonan, pl. 7, 1b-4a

Neuropteris sp. 2 1992 Feng Shaonan, pl. 7, 4b

Neuropteris sp. 1982 Wu et Zhao, p. 143

Pecopteris (Brongniart) Sternberg, 1825

Pecopteris sp. 1982 Wu et Zhao, p. 143

Rhodeopteridium (Presl) Zimmermann, 1959

?*Rhodeopteridium* sp. 1982 Wu et Zhao, p. 143

Sphenopteris (Brongniart) Sternberg, 1825

Sphenopteris cf. *obtusiloba* Brongniart 1982 Wu et Zhao, p. 143

Sphenopteris sp. 1982 Wu et Zhao, p. 143

Visean–Serpukhovian

Zhejiang

1. Shangfang Town, Quxian County, Quzhou City: Yejiatang Group

Asterophyllites Brongniart, 1822

Asterophyllites longifolius (Sternberg) Brongniart 1982 Li Hanmin et al., pl.138, 6-7

Rhodeopteridium (Presl) Zimmermann, 1959

Rhodeopteridium hsianghsiangense Sze 1982 Li Hanmin et al., pl.141, 1

Rhodeopteridium parasparsa Sze 1982 Li Hanmin et al., pl.141, 16-17

2. Xiangudong, Lijia Town, Jiande County: Yejiatang Group

Adiantites Göppert, 1836

Adiantites sp. 1982 Li Hanmin et al., pl.134, 5-6

Lepidodendron Sternberg, 1820

Lepidodendron rhodeanum Sternberg 1982 Li Hanmin et al., pl.133, 8-10

Lepidostrobophyllum Hirmer, 1927

Lepidostrobophyllum cf. *hastatum* (Lesquereux) Chaloner et Boureau 1982 Li Hanmin et al., pl.133, 4-5

3. Tangjia Town, Jinhua County: Yejiatang Group

Lepidodendron Sternberg, 1820

Lepidodendron cf. *worthenii* Lesquereux 1982 Li Hanmin et al., pl.133, 1-2

Lepidodendron cf. *volkmannianum* Sternberg 1982 Li Hanmin et al., pl.134, 2-3

4. Qiuchuan Town, Changshan County: Yejiatang Group

Rhacopteris Schimper, 1869

Rhacopteris plumosa Li HM, 1982 1982 Li Hanmin et al., pl.141, 4-9

5. Baima Town, Chun'an County, Hangzhou City: Yejiatang Group

Aneimites (Dawson) Schimper, 1874

Aneimites dichotomous Chen, 1982 1982 Li Hanmin et al., pl.134, 7

Triphyllopteris Schimper, 1869

Triphyllopteris sp. 1982 Li Hanmin et al., pl.134, 4

6. Hetang Village, Jiangshan County: Yejiatang Group

Cardiopteris Schimper, 1869

Cardiopteris frongdosa (Göppert) Schimper 1976 Chen Qishi, fig.1

Cardiopteris Schimper, 1869

Cardiopteris sp. 1982 Li Hanmin et al., pl.133, 3

Neuropteris (Brongniart) Sternberg, 1825

Neuropteris gigantea Sternberg 1976 Chen Qishi, p.192

Neuropteris sp. 1976 Chen Qishi, p.192

Sphenopteris (Brongniart) Sternberg, 1825

Sphenopteris aff. *leei* Sze 1976 Chen Qishi, p.192

7. Hejiashan Town, Jiangshan County: Yejiatang Group

Mesocalamites Hirmer, 1927

Mesocalamites sp. 1982 Li Hanmin et al., pl.133, 6-7

Jiangxi

1. Fengmenli, Leping City: Zishan Group

Lopinopteris Sze, 1958

Lopinopteris intercalata (Sze) Gu et Zhi 1982 Li Hanmin et al., pl.152, 1-4

Neuropteris (Brongniart) Sternberg, 1825

Neuropteris gigantea Sternberg 1982 Li Hanmin et al., pl.151, 3-4

Odontopteris Brongniart, 1822

Odontopteris? *lopingensis* Li HM, 1982 1982 Li et al., pl. 138, 15-17

2. Huashanling Section, Fengcheng City: Zishan Group

Bothrodendron Lindley et Hutton, 1833

Bothrodendron circulare Sze 1981 Li Hanmin, pl. 2, 8-9

Cardiopteridium Nathorst, 1914

Cardiopteridium spitsbergense Nathorst 1981 Li Hanmin, pl. 1, 1-6; 1982 Li Hanmin et al., pl.138, 9-14

Lepidodendron Sternberg, 1820

Lepidodendron huashanlingense Li, 1981 1981 Li Hanmin, pl.1, 9

Lepidodendron shanyangense Wu et He 1981 Li Hanmin, pl.1, 7-8; 1982 Li et al., pl. 136, 1

3. Fengcheng: Zishan Formatio

Mariopteris Zeiller, 1879

Mariopteris acuta Brongn. forma *obtusa* Gothan 1982 Li et al., pl. 152, 5-6

4. Sanmentan Section, Yudu County: Zishan Group

Adiantites Göppert, 1836

Adiantites gothani (Sze) Zhang, Zhao et Wu 1982b Zhao Xiuhu et Wu Xiuyuan, pl.III, 1, 1a, 2

Aphlebia Presl, 1838

Aphlebia sp. 1981 Li Hanmin, pl. 4, 11

Cardiopteridium Nathorst, 1914

Cardiopteridium spitsbergense Nathorst 1981 Li Hanmin, pl. 1, 1-6; 1982 Li Hanmin et al., pl.138, 9-14

Lepidodendron Sternberg, 1820

Lepidodendron quadratum Zhao et Wu, 1982 1982b Zhao Xiuhu et Wu Xiuyuan, pl.I, 1-3

Neuropteris (Brongniart) Sternberg, 1825

Neuropteris gigantea Sternberg 1982b Zhao Xiuhu et Wu Xiuyuan, pl.III, 4, 4a, 5, 8, 9

Neuropteris jiangxiensis Li, 1981 1981 Li Hanmin, pl. 4, 1-2; 1982 Li Hanmin et al., pl.151, 5-6

Neuropteris kaipingiana Sze 1981 Li Hanmin, pl. 4, 3-4

Neuropteris cf. *pseudogigantea* Potonié 1982b Zhao Xiuhu et Wu Xiuyuan, pl.III, 6, 6a
Neuropteris sp. 1982b Zhao Xiuhu et Wu Xiuyuan, pl.III, 7
Neuropteris sp. 1981 Li Hanmin, pl. 4, 5-6
<u>*Rhacopteris* Schimper, 1869</u>
Rhacopteris gannanensis Li, 1981 1981 Li Hanmin, pl. 2, 5-6; 1982 Li Hanmin et al., pl.141, 10-11
Rhacopteris? *zishanensis* Li, 1982 1982 Li Hanmin et al., pl.141, 12-15
<u>*Rhodeopteridium* (Presl) Zimmermann, 1959</u>
Rhodeopteridium hsianghsiangense Sze 1981 Li Hanmin, pl. 2, 16
Rhodeopteridium? sp. 1981 Li Hanmin, pl. 2, 1-4
<u>*Sigillaria* Brongniart, 1822</u>
Sigillaria brardii Brongniart 1982b Zhao Xiuhu et Wu Xiuyuan , pl.II, 1, 2
<u>*Sphenophyllostachys* Seward, 1898</u>
Sphenophyllostachys tenerrimus (Ettingshausen) Stockmans et Williere, 1953 1982b Zhao Xiuhu et Wu Xiuyuan, pl.II, 8, 8a, 9
<u>*Sphenophyllum* Brongniart, 1828</u>
Sphenophyllum tenertimum Ettingshausen 1981 Li Hanmin, pl. 2, 7; 1982 Li Hanmin et al., pl.138, 8
Sphenophyllum pseudotenerrimum Sze 1981 Li Hanmin, pl. 2, 14-15
Sphenophyllum yuduense Li, 1981 1981 Li Hanmin, pl. 2, 10-13; 1982 Li Hanmin et al., pl.138, 1-4
<u>*Trigonocarpus* Brongniart, 1828</u>
Trigonocarpus schultzianus Göppert et Berg. 1981 Li Hanmin, pl. 4, 7-10; 1982 Li et al., pl. 151, 7-10
Trigonocarpus sp. 1982b Zhao Xiuhu et Wu Xiuyuan, p. 700

5. Zishanwei Section, Yudu County: Zishan Group

<u>*Adiantites* Göppert, 1836</u>
Adiantites gothani (Sze) Zhang, Zhao et Wu 1982b Zhao Xiuhu et Wu Xiuyuan, pl.III, 1, 1a, 2
<u>*Lepidodendron* Sternberg, 1820</u>
Lepidodendron cf. *shanyangense* Wu et He 1982b Zhao Xiuhu et Wu Xiuyuan, pl. I, 5, 5a
Lepidodendron yuduense Zhao et Wu, 1982 1982b Zhao Xiuhu et Wu Xiuyuan, pl.I, 4
<u>*Lepidostrobophyllum* Hirmer, 1927</u>
Lepidostrobophyllum cf. *ovatifolium* (Lesquereux) Chaloner et Boureau 1982b Zhao Xiuhu et Wu Xiuyuan, pl.II, 4, 5
<u>*Mesocalamites* Hirmer, 1927</u>
Mesocalamites sp. 1982b Zhao Xiuhu et Wu Xiuyuan, p. 699
<u>*Neuropteris* (Brongniart) Sternberg, 1825</u>
Neuropteris gigantea Sternberg 1982b Zhao Xiuhu et Wu Xiuyuan, pl.III, 4, 4a, 5, 8, 9
Neuropteris sp. 1982b Zhao Xiuhu et Wu Xiuyuan, p. 699
<u>*Platyphyllum* Dawson, 1888</u>
?*Platyphyllum* sp. 1982b Zhao Xiuhu et Wu Xiuyuan, p. 699
<u>*Sigillaria* Brongniart, 1822</u>
Sigillaria brardii Brongniart 1982b Zhao Xiuhu et Wu Xiuyuan , pl.II, 1, 2
<u>*Sphenophyllum* Brongniart, 1828</u>
Sphenophyllum tenerrimum Ettingshausen 1982b Zhao Xiuhu et Wu Xiuyuan, p. 699
<u>*Sphenopteris* (Brongniart) Sternberg, 1825</u>
Sphenopteris sp. 1982b Zhao Xiuhu et Wu Xiuyuan, pl.III, 3
<u>*Stigmaria* Brongniart, 1822</u>

Stigmaria ficoides (Sternberg) Brongniart 1982b Zhao Xiuhu et Wu Xiuyuan, p. 699

Trigonocarpus Brongniart, 1828

Trigonocarpus ellipticus Zhao et Wu, 1982 1982b Zhao Xiuhu et Wu Xiuyuan, pl.II, 10-12

Trigonocarpus sp. 1982b Zhao Xiuhu et Wu Xiuyuan, pl.II, 13, 13a

6. Guyuan Village, Zishan Town, Yudu County: Zishan Group

Calamostachys Schimper, 1869

Calamostachys? sp. 1981 Li Hanmin, pl. 4, 12A-13

Rhodeopteridium (Presl) Zimmermann, 1959

Rhodeopteridium? sp. 1981 Li Hanmin, pl. 2, 12B

Sigillaria Brongniart, 1822

Sigillaria brardii Brongniart 1982 Li et al., pl.132, 4-5

Tibet

Machala Section, Leiwuqi County, Changdu: Zishan Group

Archaeocalamites Stur, 1875

Archaeocalamites scrobiculatus Schlotheim ex Seward 1974 Gu et Zhi, pl.27, 1-2; fig.41

Rhodeopteridium (Presl) Zimmermann, 1959

Rhodeopteridium tenuis Gothan 1974 Gu et Zhi, pl.51, 9-14

Sphenophyllum Brongniart, 1828

Sphenophyllum tenerrimum Ettingshausen 1974 Gu et Zhi, pl. 21, 5-7

Henan

1. Shangcheng and Gushi: Yangshan Formation

Archaeocalamites Stur, 1875

Archaeocalamites scrobiculatus Schlotheim ex Seward 1996 Chen et Sun, pl.II, 4; pl.III, 1, 2

Bothrodendron Lindley et Hutton, 1833

Bothrodendron sp. a 1996 Chen Fen et Sun Keqin, pl.I, 7

Bothrodendron sp. b 1996 Chen Fen et Sun Keqin, p.315

Cardiocarpus Brongniart, 1881

Cardiocarpus cordai (Geinitz) Gu et Zhi 1996 Chen Fen et Sun Keqin, p.315

Cardiopteridium Nathorst, 1914

Cardiopteridium spitsbergense Nathorst 1996 Chen Fen et Sun Keqin, p.315

Carpolithus Brongniart, 1822

Carpolithus sp. 1996 Chen Fen et Sun Keqin, p.315

Cordaites Unger, 1850

Cordaites schenkii Halle 1996 Chen Fen et Sun Keqin, p.315

Lepidodendron Sternberg, 1820

Lepidodendron cf. *aolungpylukense* Sze 1996 Chen Fen et Sun Keqin, p.315

Lepidodendron cf. *subrhombicum* Gu et Zhi 1996 Chen Fen et Sun Keqin, p.315

Lepidodendron shanyangense Wu et He 1996 Chen Fen et Sun Keqin, pl.I, 1, 1a, 2

Lepidodendron sp. a 1996 Chen Fen et Sun Keqin, p.315

Lepidodendron sp. b 1996 Chen Fen et Sun Keqin, p.315

Lepidostrobus Brongniart, 1828

Lepidostrobus? sp. 1996 Chen Fen et Sun Keqin, p.315

Paripteris Gothan, 1941

Paripteris cf. *pseudogigantea* (Potonié) Gothan 1996 Chen Fen et Sun Keqin, p.315

Paripteris? sp. 1996 Chen Fen et Sun Keqin, p.315

Rhodeopteridium (Presl) Zimmermann, 1959

Rhodeopteridium hsianghsiangense (Sze) Zhang, Zhao et Wu 1996 Chen Fen et Sun Keqin, pl.III, 4

Stigmaria Brongniart, 1822

Stigmaria ficoides (Sternberg) Brongniart 1996 Chen Fen et Sun Keqin, p.315

Triphyllopteris Schimper, 1869

Triphyllopteris sp. 1996 Chen Fen et Sun Keqin, p.315

2. Yangshan Coal Mine, Gushi County: Yangshan Formation

Adiantites Göppert, 1836

Adiantites cf. *gothani* (Sze) 1992 Wu Xiuyuan, pl.IV, 9; pl.VI, 6, 7b

Aneimites Ettingshausen, 1865

Aneimites sp. 1992 Wu Xiuyuan, pl.VI, 2-3a

Archaeocalamites Stur, 1875

Archaeocalamites scrobiculatus Schlotheim ex Seward 1992 Wu Xiuyuan, pl.IV, 1-5, 1a, 4a; pl.V, 1-3; fig.3,4

Bothrodendron Lindley et Hutton, 1833

Bothrodendron flabellatum Wu, 1992 1992 Wu Xiuyuan, pl.III, 5-6b

Bothrodendron? *yangshanense* Wu, 1992 1992 Wu Xiuyuan, pl.III, 4-4b; fig.2

Cardiopteridium Nathorst, 1914

Cardiopteridium podozamioides Sze 1992 Wu Xiuyuan, pl.IV, 6-8

Cardiopteridium spitsbergense Nathorst 1992 Wu Xiuyuan, p.565

Cathaysiodendron Lee, 1963

Cathaysiodendron gushiense (Wu) Zhang et al., 2006 1992 Wu Xiuyuan, pl.III, 1, 1; 1997 Wu et Liu, pl. I, 2, 3; 2006 Zhang et al., p. 266

Cathaysiodendron yangshanense Zhang et al., 2006 2006 Zhang et al., fig. 1, a, b, c

Cathaysiodendron? sp. 1992 Wu Xiuyuan, pl.III, 3, 3a; fig.1; 1997 Wu Xiuyuan et Liu Lujun, pl.4, 2

Eusphenopteris Gothan ex Simson-Scharold, 1934

Eusphenopteris cf. *scribanii* Amerom 1992 Wu Xiuyuan, pl.VI, 8, 8a; fig.6

Lepidodendron Sternberg, 1820

Lepidodendron aff. *aolungpylukense* Sze 1992 Wu Xiuyuan, pl.I, I-3, 2a

Lepidodendron dabieshanense Wu, 1992 1992 Wu Xiuyuan, pl.II, 7-7b

Lepidodendron cf. *shanyangense* Wu et He 1992 Wu Xiuyuan, pl.II, 2-5

Lepidodendron cf. *worthenii* Lesqureux 1992 Wu Xiuyuan, pl.I, 4-6a; pl.II, 1

Lepidodendron sp. 1992 Wu Xiuyuan, pl.III, 2, 2a

Lepidostrobophyllum Hirmer, 1927

Lepidostrobophyllum sp. 1992 Wu Xiuyuan, p.565

Neuropteris Brongniart, 1825

Neuropteris sp. 1992 Wu Xiuyuan, p.565

Pothocites Paterson, 1844

Pothocites sp. 1992 Wu Xiuyuan, p.565

Rhodeopteridium (Presl) Zimmermann, 1959

Rhodeopteridium hsianghsiangense Sze 1992 Wu Xiuyuan, pl.VI, 4-5a

Sphenophyllum Brongniart, 1828

Sphenophyllum sp. 1992 Wu Xiuyuan, p.565

Sphenopteris (Brongniart) Sternberg, 1825

Sphenopteris cf. *gracilis* Brongniart 1992 Wu Xiuyuan, pl.V, 8-9; fig.5

Sphenopteris cf. *scribanii* Amerom 1992 Wu Xiuyuan, p.565

Stigmaria Brongniart, 1822

Stigmaria ficoides (Sternberg) Brongniart 1992 Wu Xiuyuan, p.565

Telangium Benson, 1904

Telangium sp. 1992 Wu Xiuyuan, p.565

Triphyllopteris Schimper, 1869

Triphyllopteris gushiensis Wu, 1992 1992 Wu Xiuyuan, pl.V, 4, 4a

Triphyllopteris sp. 1992 Wu Xiuyuan, pl.V, 5-7

Shanxi

Eryuhe Section, Shanyang County: Eryuhe Formation

Adiantites Göppert, 1836

Adiantites sp. 1978 Deng Bao, pl.I, 13, 21

Archaeocalamites Stur, 1875

Archaeocalamites scrobiculatus Schlotheim ex Seward 1978 Deng Bao, pl.I, 10; 1983 Liu Zijin et Shen Guanglong, pl.160, 9

Cardiopteridium Nathorst, 1914

Cardiopteridium spitsbergense Nathorst 1978 Deng Bao, p. 15; 1983 Liu Zijin et Shen Guanglong, pl.164, 4-9

Cardiopteridium nanum (Eichwald) Nathorst 1978 Deng Bao, pl.I, 10

Cardiopteris Schimper, 1869

Cardiopteris? spp. 1978 Deng Bao, pl.I, 11, 12

Lepidodendron Sternberg, 1820

Lepidodendron shanyangense Wu et He, 1978 1978 Deng Bao, pl.I, 1-5

Rhodeopteridium (Presl) Zimmermann, 1959

Rhodeopteridium hsianghsiangensis Sze 1978 Deng Bao, pl.I, 10; 1983 Liu Zijin et Shen Guanglong, pl.165, 6

Triphyllopteris Schimper, 1869

Triphyllopteris collombiana Schimper 1978 Deng Bao, pl.I, 10; 1983 Liu Zijin et Shen Guanglong, pl.164, 10-11

Serpukhovian

Fujian

Xiangliao Village, Chishui Town, Zhangping City: Upper Lindi Group

Cordaites Unger, 1850

Cordaites sp. 1982 Wu et Zhao, p. 143

Neuropteris (Brongniart) Sternberg, 1825

Neuropteris gigantea Sternberg 1982 Wu et Zhao, p. 143

Neuropteris sp. 1982 Wu et Zhao, p. 143

Rhodeopteridium (Presl) Zimmermann, 1959

Rhodeopteridium sp. 1982 Wu et Zhao, p. 143

Sigillaria Brongniart, 1822

Sigillaria sp. 1982 Wu et Zhao, p. 143

Zhejiang

1. Xiangudong, Lijia Town, Jiande City: Middle Yejiatang Group

Adiantites Göppert, 1836

Adiantites sp.1 1986 Zhao Xiuhu et al., pl.VII, 1-4

Hexagonocarpus Renault, 1890

Hexagonocarpus ellipticus Zhao, 1986 1986 Zhao Xiuhu et al., pl.XV, 5, 6

Lepidodendron Sternberg, 1820

Lepidodendron jiandeense Zhao et Chen, 1986 1986 Zhao et al., pl.III, 1, 1a; pl.IV, 3, 4

Lepidodendron quadratum Zhao et Wu 1986 Zhao Xiuhu et al., pl.II, 1-4, 2a

Lepidostrobophyllum Hirmer, 1927

Lepidostrobophyllum cf. *ovatifolium* (Lesquereux) Chaloner et Boureau 1986 Zhao Xiuhu et al., pl.III, 4, 5; pl. IV, 8

Rhodeopteridium (Presl) Zimmermann, 1959

Rhodeopteridium sp. 1986 Zhao Xiuhu et al., p. 4

Stigmaria Brongniart, 1822

Stigmaria ficoides (Sternberg) Brongniart 1986 Zhao Xiuhu et al., p. 4

2. Shimatou Coal Mine, Xin’anjiang Town, Jiande County: Middle Yejiatang Group

Linopteris Presl, 1838

Linopteris sp. 1986 Zhao Xiuhu et al., pl.XIV, 6, 7, 6a, 7a

Sphenophyllum Brongniart, 1828

Sphenophyllum tenerrimum Ettingshausen 1986 Zhao Xiuhu et al., pl. V, 3-5

3. Tangjia Town, Jinhua City: Middle Yejiatang Group

Lepidodendron Sternberg, 1820

Lepidodendron quadratum Zhao et Wu 1987 Chen Qishi, pl.5, 6; 1986 Zhao Xiuhu et al., pl.II, 1-4, 2a

Lepidodendron tangjiaense Chen, 1987(= *Lepidodendron* cf. *worthenii* Lesquereux) 1987 Chen Qishi, pl.5, 5

Lepidodendron cf. *worthenii* Lesquereux 1986 Zhao Xiuhu et al., pl.II, 5, 5a

Lepidodendron sp. 1986 Zhao Xiuhu et al., pl.III, 2, 3

4. Qiuchuan Town, Changshan County: Middle Yejiatang Group

Bothrodendron Lindley et Hutton, 1833

Bothrodendron ellipticum Zhao, 1986 1986 Zhao Xiuhu et al., pl.IV, 2, 2a

Cyclopteris Brongniart, 1828

Cyclopteris sp.1 1986 Zhao Xiuhu et al., pl.XIV, 8; pl.XV, 1-3

Lepidostrobophyllum Hirmer, 1927

Lepidostrobophyllum cf. *ovatifolium* (Lesquereux) Chaloner et Boureau 1986 Zhao Xiuhu et al., pl.III, 4, 5; pl. IV, 8

Mariopteris Zeiller, 1879

Mariopteris acuta Brongniart cf. *obtusa* Gothan 1986 Zhao Xiuhu et al., pl.XII, 4, 5, 4a; pl.XIII, 15; 1987 Chen, pl. V, 7, 15

Neuropteris (Brongniart) Sternberg, 1825

Neuropteris gigantea Sternberg 1986 Zhao Xiuhu et al., pl.XIII, 9-13, 14?

Sphenophyllum Brongniart, 1828

Sphenophyllum tenerrimum Ettingshausen 1986 Zhao Xiuhu et al., pl. V, 3-5

Sphenopteris (Brongniart) Sternberg, 1825

Sphenopteris leei Sze 1986 Zhao Xiuhu et al., pl. VII, 6, 6a

Sphenopteris sp. 1986 Zhao Xiuhu et al., pl.VII, 8-11, 8a-11a; 1987 Chen, pl. V, 14

Stigmaria Brongniart, 1822

Stigmaria sp. 1986 Zhao Xiuhu et al., pl.II, 6, 6a

Trigonocarpus Brongniart, 1828

Trigonocarpus sp.2 1986 Zhao Xiuhu et al., pl.XV, 12-14

5. Shangfang Town, Quxian County, Quzhou City: Middle Yejiatang Group

Cyclopteris Brongniart, 1828

Cyclopteris sp.1 1986 Zhao Xiuhu et al., pl.XIV, 8; pl.XV, 1-3

Linopteris Presl, 1838

Linopteris sp. 1986 Zhao Xiuhu et al., pl.XIV, 6, 7, 6a, 7a; 1992 Li Xingxue et al., pl. II, 8, 9

Rhodeopteridium (Presl) Zimmermann, 1959

Rhodeopteridium chunanense Wu, 1986 1986 Zhao Xiuhu et al., pl.IX, 1, 2; pl. XI, 2, 3

Rhodeopteridium cf. *lipoldi* (Stur) Patteisky 1986 Zhao Xiuhu et al., pl.X, 4; pl.XII, 1-3

Rhodeopteridium machanekii Ettingshausen 1986 Zhao Xiuhu et al., pl.V, 8

Rhodeopteridium multibifidum Wu, 1986 1986 Zhao Xiuhu et al., pl.X, 1-3; pl.XI, 1

Rhodeopteridium quxianense Wu, 1986 1986 Zhao Xiuhu et al., pl.IX, 3, 4

Sphenopteris (Brongniart) Sternberg, 1825

Sphenopteris (*Palmatopteris*) sp. 1986 Zhao Xiuhu et al., pl.V, 6, 7

6. Baima Town, Chun'an County, Hangzhou City: Middle Yejiatang Group

Lepidostrobophyllum Hirmer, 1927

Lepidostrobophyllum cf. *ovatifolium* (Lesquereux) Chaloner et Boureau 1986 Zhao Xiuhu et al., pl.III, 4, 5; pl. IV, 8

Rhodeopteridium (Presl) Zimmermann, 1959

Rhodeopteridium chunanense wu, 1986 1986 Zhao Xiuhu et al., pl.IX, 1, 2; pl. XI, 2, 3

7. Xiangudong, Chun'an County: Middle Yejiatang Group

Adiantites Göppert, 1836

Adiantites sp. 1987 Chen, pl. V, 9

Lepidodendron Sternberg, 1820

Lepidodendron jiandeense Zhao et Chen, 1986 1987 Chen, pl.5, 3-4

8. Yejiatang Section, Fengjia Town, Kaihua County: Middle Yejiatang Group

Adiantites Göppert, 1836

Adiantites sp. 1986 Zhao Xiuhu et al., p. 3

Mariopteris Zeiller, 1879

Mariopteris acuta Brongniart cf. *obtusa* Gothan 1986 Zhao Xiuhu et al., pl.XII, 4, 5, 4a; pl.XIII, 15

Neuropteris (Brongniart) Sternberg, 1825
Neuropteris sp. 1986 Zhao Xiuhu et al., p. 3
Neuropteris gigantea Sternberg 1986 Zhao Xiuhu et al., pl.XIII, 9-13, 14?
Neuropteris otozamioides Sze et Lee 1986 Zhao Xiuhu et al., pl.XIII, 7, 8, 7a
Rhodeopteridium (Presl) Zimmermann, 1959
Rhodeopteridium cf. *hsianghsiangense* Sze 1986 Zhao Xiuhu et al., p. 3
Rhodeopteridium cf. *nematophyllum* Daber 1986 Zhao Xiuhu et al., p. 4
Sigillaria Brongniart, 1822
?*Sigillaria* sp. 1986 Zhao Xiuhu et al., pl.III, 6
Sphenophyllum Brongniart, 1828
Sphenophyllum tenerrimum Ettingshausen 1986 Zhao Xiuhu et al., pl. V, 3-5
Sphenopteris (Brongniart) Sternberg, 1825
Sphenopteris cf. *schatzlarensis* Kidston 1986 Zhao Xiuhu et al., pl.VII, 7; 1987 Chen, pl. V, 13

9. Hetang Village, Jiangshan County: Middle Yejiatang Group

Cyclopteris Brongniart, 1828
?*Cyclopteris* sp.2 1986 Zhao Xiuhu et al., pl.XV, 4
Cardiopteris Schimper, 1869
Cardiopteris sp. 1987 Chen Qishi, pl.5, 10
Mesocalamites Hirmer, 1927
Mesocalamites jiangshanensis Chen, 1986 1986 Zhao Xiuhu et al., pl. IV, 6, 7; 1987 Chen, pl. V, 1
Neuropteris (Brongniart) Sternberg, 1825
Neuropteris gigantea Sternberg 1986 Zhao Xiuhu et al., pl.XIII, 9-13, 14?
Neuropteris schlehani Stur. cf. *rectinervis* Kidston 1986 Zhao et al., pl.XIII, 1-5, 1a, 3a, 5a; 1992 Li Xingxue et al., pl. I, 7, 8
Neuropteris zhejiangensis Chen, 1986 1986 Zhao Xiuhu et al., pl.XIV, 1-3, 3a
Trigonocarpus Brongniart, 1828
Trigonocarpus sp.1 1986 Zhao Xiuhu et al., pl.XV, 8-11

10. Hejiashan Town, Jiangshan County: Mid-Upper Yejiatang Group

Cyclopteris Brongniart, 1828
Cyclopteris sp.1 1986 Zhao Xiuhu et al., pl.XIV, 8; pl.XV, 1-3
Hexagonocarpus Renault, 1890
Hexagonocarpus ellipticus Zhao, 1986 1986 Zhao Xiuhu et al., pl.XV, 5, 6
Mesocalamites Hirmer, 1927
Mesocalamites sp. 1986 Zhao Xiuhu et al., p. 3
Neuropteris (Brongniart) Sternberg, 1825
Neuropteris gigantea Sternberg 1986 Zhao Xiuhu et al., p. 3
Neuropteris cf. *kaipingiana* Sze 1986 Zhao Xiuhu et al., pl.XIII, 6, 6a
Neuropteris schlehani Stur. cf. *rectinervis* Kidston 1986 Zhao et al., pl.XIII, 1-5, 1a, 3a, 5a; 1987 Chen, pl. V, 12b; 1992 Li Xingxue et al., pl. I, 9, 10
Neuropteris sp. (*Neuropteris* cf. *rarinervis* Bunburg) 1986 Zhao Xiuhu et al., pl.XIV, 4, 5, 4a, 5a
Rhacopteris Schimper, 1869
Rhacopteris plumosa Li, 1982 1987 Chen, pl. V, 11
Rhacopteris sp. 1986 Zhao Xiuhu et al., pl.VI, 1, 1a

Sphenopteris (Brongniart) Sternberg, 1825

Sphenopteris sp. 1986 Zhao Xiuhu et al., p. 3

Trigonocarpus Brongniart, 1828

Trigonocarpus sp. 1986 Zhao Xiuhu et al., p. 3

11. Zhucangwu Section, Linghu Village, Changshan County: Upper Yejiatang Group

No land plant record

Jiangxi

1. Leiping: Upper Zishan Group

Lopinopteris Sze, 1959

Lopinopteris intercalata (Sze) Gu et Zhi 1963 Zhou, pl. 68, 3; 1974 Gu et Zhi, pl. 81, 4-10; 1997 Wu Xiuyuan et Liu Lujun, pl.1, 4

Paripteris Gothan, 1941

Paripteris gigantea (Sternberg) Gothan 1997 Wu Xiuyuan et Liu Lujun, pl.1, 7

Sphenopteris (Brongniart) Sternberg, 1825

Sphenopteris obtusiloba Brongniart 1982 Li Hanmin et al., pl.138, 5

2. Fengcheng: Upper Zishan Group

Karinopteris Boersma, 1972

Karinopteris acuta Brongniart forma *obtusa* Gothan 1997 Wu Xiuyuan et Liu Lujun, pl.1, 5, 6

Mariopteris Zeiller, 1879

Mariopteris acuta Brongniart forma *obtusa* Gothan 1974 Gu et Zhi, pl. 84, 9

3. Yudu: Upper Zishan Group

Lepidodendron Sternberg, 1820

Lepidodendron quadratum Zhao et Wu, 1982 1997 Wu et Liu, pl.1, 1

4. Folingwei Section, Fengcheng City: Foling Formation

Aspidiaria Presl, 1838

Aspidiaria sp. 1964 Zhang Caifan et Liuyaguang, p. 372

Asterocalamites Schimper ex Zeiller, 1879

Asterocalamites cf. *scrobiculatus* (Schlotheim) Zeiller 1964 Zhang Caifan et Liuyaguang, p. 372

Calamites Brongniart, 1828

Calamites sp. 1964 Zhang Caifan et Liuyaguang, p. 372

Cordaites Unger, 1850

Cordaites sp. 1964 Zhang Caifan et Liuyaguang, p. 372

Lepidodendron Sternberg, 1820

Lepidodendron sp. 1964 Zhang Caifan et Liuyaguang, p. 372

Mariopteris Zeiller, 1879

Mariopteris acuta f. *obtusa* 1982 Wu et Zhao, p. 142; 1964 Zhang Caifan et Liuyaguang, p. 372

Mariopteris sp. 1964 Zhang Caifan et Liuyaguang, p. 372

Neuropteris (Brongniart) Sternberg, 1825

Neuropteris gigantea Sternberg 1982 Wu et Zhao, p. 142; 1964 Zhang Caifan et Liuyaguang, p. 372

Rhodeopteridium (Presl) Zimmermann, 1959
Rhodeopteridium sp. 1964 Zhang Caifan et Liuyaguang, p. 372
Rhodeopteridium cf. *hsianghsiangensis* Sze 1964 Zhang Caifan et Liuyaguang, p. 372
Sphenopteris (Brongniart) Sternberg, 1825
Sphenopteris obtusiloba Brongniart 1982 Wu et Zhao, p. 142; 1964 Zhang Caifan et Liuyaguang, p. 372
Stigmaria Brongniart, 1822
Stigmaria ficoides (Sternberg) Brongniart 1964 Zhang Caifan et Liuyaguang, p. 372

5. Huashanling Section, Fengcheng City: Foling Formation

Asterocalamites Schimper ex Zeiller, 1879
Asterocalamites cf. *scrobiculatus* (Schlotheim) Zeiller 1964 Zhang Caifan et Liuyaguang, p. 372
Cordaites Unger, 1850
Cordaites sp. 1964 Zhang Caifan et Liuyaguang, p. 372
Neuropteris (Brongniart) Sternberg, 1825
Neuropteris gigantea Sternberg 1964 Zhang Caifan et Liuyaguang, p. 372

6. Chayuanshan Section, Fuzhou City: Foling Formation

Neuropteris (Brongniart) Sternberg, 1825
Neuropteris sp. 1964 Zhang Caifan et Liuyaguang, p. 374
Sphenopteris (Brongniart) Sternberg, 1825
Sphenopteris sp. 1964 Zhang Caifan et Liuyaguang, p. 374

7. Bajiaoling Section, Yingtan City: Foling Formation

Neuropteris (Brongniart) Sternberg 1825
Neuropteris gigantea Sternberg 1964 Zhang Caifan et Liuyaguang, p. 374
Neuropteris sp. 1964 Zhang Caifan et Liuyaguang, p. 374

8. Da'ao Section, Yundu County: Foling Formation

Neuropteris (Brongniart) Sternberg, 1825
Neuropteris gigantea Sternberg 1964 Zhang Caifan et Liuyaguang, p. 375
Neuropteris sp. 1964 Zhang Caifan et Liuyaguang, p. 375

9. Gongshui River: Zishan Group

Neuropteris (Brongniart) Sternberg, 1825
Neuropteris gigantea Sternberg 1942 Sze et Chen, p. 150

10. Wangbashan Mountain, Leping County: Zishan Group

Lopinopteris Sze, 1958
Lopinopteris intercalata Sze 1958 1958 Sze, pl.II, 1-4; pl.III, 4-6; 1963 Zhou, pl.68, 3
Neuropteris (Brongniart) Sternberg, 1825
Neuropteris gigantea Sternberg 1958 Sze, pl.I, 1-6, 6a
Paripteris Gothan, 1941
Paripteris gigantea (Sternberg) Gothan 1992 Li Xingxue et al., pl.1, 1
Rhodeopteridium (Presl) Zimmermann, 1959

Rhodeopteridium sp. (cf. *Rhodeopteridium subpetiolata* Potonié) 1958 Sze, pl.I, 7-8
Sphenopteris (Brongniart) Sternberg, 1825
Sphenopteris obtusiloba Brongniart 1958 Sze, pl.III, 1-3

11. Raojiashan Section, Fengcheng City: Upper Zishan Group
Mariopteris Zeiller, 1879
Mariopteris acuta Brongn. forma *obtusa* Gothan 1964 Li Xingxue, pl.II, 1-6

12. 11 localities: Zishan Group
Neuropteris (Brongniart) Sternberg, 1825
Neuropteris gigantea Sternberg 1943 Sze et Chen, p.150
Sphenopteris (Brongniart) Sternberg, 1825
Sphenopteris sp. 1943 Sze et Chen Guoda, p.150
Stigmaria Brongniart, 1822
Stigmaria ficoides (Sternberg) Brongniart 1943 Sze et Chen Guoda, p.150

Bashkirian

Shaanxi

1. Zhugou Section, Caoliangyi Village, Fengxian County: Caoliangyi Formation
Paripteris Gothan, 1941
Paripteris pseudogigantea (Potonié) Gothan 1997 Wu Xiuyuan et Liu Lujun, pl.4, 12, 13
Sphenopteris (Brongniart) Sternberg, 1825
Sphenopteris parabaeumleri Sze, 1953 1953b Sze, pl.I, 1-6; pl.II, 1-5
Sphenopteris (*Lyginopteris*) *parabaeumleri* Sze 1953a Sze, pl.8, 4-7; pl.9, 1-5

2. Meitangou Section, Caoliangyi Village, Fengxian County: Caoliangyi Formation
Neuropteris Brongniart, 1828
Neuropteris gigantea Sternberg 1956 Wang Dewen, p.39

3. Luobo'an Section, Fengxian County: Caoliangyi Formation
Anisopteris (Oberste Brink) Hirmer
Anisopteris petiolata (Göppert) Hirmer 1983 Wu Xiuyuan et Deng Bao, pl.I, 8, 9; pl.II, 7
Diplotmema? *subgeniculata* Stur 1983 Wu Xiuyuan et Deng Bao, pl.II, 5
Mesocalamites Hirmer, 1927
Mesocalamites sp. 1983 Wu Xiuyuan et Deng Bao, pl.I, 1
Neuropteris Brongniart, 1828
Neuropteris longifolia Wu et Deng, 1983 1983 Wu Xiuyuan et Deng Bao, pl.III, 2
Sphenophyllum Brongniart, 1828
Sphenophyllum tenerrimum Ettingshausen 1983 Wu Xiuyuan et Deng Bao, p. 184
Sphenopteris (Brongniart) Sternberg, 1825
Sphenopteris parabaeumleri Sze 1983 Wu Xiuyuan et Deng Bao, p. 184

4. Caotangou Section, Fengxian County: Caoliangyi Formation

Bothrodendron Lindley et Hutton, 1833

Bothrodendron sp. 1983 Wu Xiuyuan et Deng Bao, pl. II, 1

Calamites Brongniart, 1828

Calamites sp. 1983 Wu Xiuyuan et Deng Bao, p. 184

Cyclopteris Brongniart, 1828

Cyclopteris sp. 1983 Wu Xiuyuan et Deng Bao, p. 184

Lepidodendron Sternberg, 1820

Lepidodendron sp. 1983 Wu Xiuyuan et Deng Bao, p. 184

Linopteris Presl, 1838

Linopteris neuropteroides Potoni é 1983 Wu Xiuyuan et Deng Bao, pl.III, 8, 9

Linopteris cf. *oblique* Brongniart 1983 Wu Xiuyuan et Deng Bao, pl.III, 10, 11

Neuropteris Brongniart, 1828

Neuropteris sp. 1983 Wu Xiuyuan et Deng Bao, pl. I, 12

Neuropteris gigantea Sternberg 1983 Wu Xiuyuan et Deng Bao, p. 184

Neuropteris pseudogigantea Potoni é 1983 Wu Xiuyuan et Deng Bao, p. 184

Neuropteris cf. *scheuchzeri* Hoffmann 1983 Wu Xiuyuan et Deng Bao, pl.I, 13

Rhodeopteridium (Presl) Zimmermann

Rhodeopteridium lipoldi Stur 1983 Wu Xiuyuan et Deng Bao, pl.III, 1

Rhodeopteridium cf. *sublipoldi* Stockmans et Williere 1983 Wu Xiuyuan et Deng Bao, pl.I, 7

Rhodeopteridium fengxianense Wu et Deng 1983 1983 Wu Xiuyuan et Deng Bao, pl.I, 5, 6

Rhodeopteridium parasparsum Sze 1983 Wu Xiuyuan et Deng Bao, pl. I, 4

Senftenbergia Corda, 1845

Senftenbergia plumosa (Artis) Stur 1983 Wu Xiuyuan et Deng Bao, pl.IV, 4-7

Sphenopteris (Brongniart) Sternberg, 1825

Sphenopteris leei Sze 1983 Wu Xiuyuan et Deng Bao, p. 184

Sphenopteris obtusiloba Brongniart 1983 Wu Xiuyuan et Deng Bao, p. 184

Sphenopteris parabaeumleri Sze 1983 Wu Xiuyuan et Deng Bao, p. 184

Sphenopteris (Cyclosphenopteris) schillingsi Andrae 1983 Wu Xiuyuan et Deng Bao, pl.II, 3, 4

Microfossil Plants of South China (Pp. 86–177)

Llandovery

Guizhou

Dongkala Section, Fenggang County: Hanjiadian Formation

Ambitisporites avitus Hoffmeister 1995 Cai Chongyang, pl.3, 1
Ambitisporites avitus Hoffmeister 1997 Wang Yi et Ouyang Shu, pl.I, 5, 8
Ambitisporites cf. *dilutus* (Hoffmeister) Richardson et Lister 1997 Wang Yi et Ouyang Shu, pl.I, 1-3
Ambitisporites cf. *dilutus* (Hoffmeister) Richardson et Lister 1995 Cai Chongyang, pl.3, 2
Ambitisporites dilutus (Hoffmeister) Richardson et Lister 1995 Cai Chongyang, pl.3, 4
Ambitisporites dilutus (Hoffmeister) Richardson et Lister 1997 Wang Yi et Ouyang Shu, pl.I, 4
Apiculiretusispora sp. 1995 Cai Chongyang, pl.3, 6
Apiculiretusispora sparsa Wang et Ouyang, 1997 1997 Wang Yi et Ouyang Shu, pl.I, 9
Apiculiretusispora spicula Richardson et Lister 1997 Wang Yi et Ouyang Shu, pl.I, 10
Apiculiretusispora? sp. 1995 Cai Chongyang, p.8
Leiotriletes sp. 1997 Wang Yi et Ouyang Shu, p.219
?*Nodosphaera oyleri* Strother et Traverse 1995 Cai Chongyang, pl.3, 8
Punctatisporites sp. 1995 Cai Chongyang et al., p.8
Punctatisporites sp. 1997 Wang Yi et Ouyang Shu, p.219
Retusotriletes cf. *abundo* Rodriguoz 1997 Wang Yi et Ouyang Shu, pl.I, 14
Retusotriletes cf. *triangulatus* (Streel) Streel 1995 Cai Chongyang, pl.3, 10
Retusotriletes cf. *triangulatus* (Streel) Streel 1997 Wang Yi et Ouyang Shu, pl.I, 6, 7
Retusotriletes cf. *warringtonii* Richardson et Lister 1995 Cai Chongyang, pl.3, 3
Retusotriletes cf. *warringtonii* Richardson et Lister 1997 Wang Yi et Ouyang Shu, pl.I, 13
Retusotriletes minor Kedo 1997 Wang Yi et Ouyang Shu, pl.I, 11
Retusotriletes warringtonii Richardson et Lister 1995 Cai Chongyang, pl.3, 5
Retusotriletes warringtonii Richardson et Lister 1997 Wang Yi et Ouyang Shu, pl.I, 12
Tetrahedraletes? sp. 1995 Cai Chongyang, pl.3, 7

Llandovery–Wenlock

Guizhou

Hou'ershan Section, Dushan County: Upper Wengxiang Group

Ambitisporites avitus Hoffmeister, 1959 1993 Gao Lianda, pl.1, 8
Ambitisporites dilutus (Hoffmeister) Richardson et Lister, 1969 1993 Gao Lianda, pl.1, 7
Ambitisporites sp. 1981 Gao Lianda, p. 11
Apiculiretusispora sp. 1981 Gao Lianda, p. 11
Apiculiretusispora sp. A 1993 Gao Lianda, pl.1, 2-3
Apiculiretusispora sp. B 1993 Gao Lianda, pl.1, 4
Apiculiretusispora spicula Richardson et Lister, 1969 1993 Gao Lianda, pl.1, 5
Archaeozonotriletes chulus var. *chulus* Richardson et Lister, 1969 1993 Gao Lianda, pl.1, 9
Emphanisporites? sp. 1993 Gao Lianda, pl.1, 6
Leiotriletes sp. 1981 Gao Lianda, p. 11
Punctatisporites sp. 1981 Gao Lianda, p. 11

Retusotriletes sp. 1981 Gao Lianda, p. 11

Retusotriletes warringtonii Richardson et Lister, 1969 1993 Gao Lianda, pl.1, 1

Wenlock

Anhui

Daijiahui Reservior, Nanling County: Maoshan Group

Archaeozonotriletes sp. 1982 Hou Jingpeng, pl. II, 34

Punctatisporites sp. 1982 Hou Jingpeng, pl. II, 33

Ludlow–Pridoli

Yunnan

Xiangyangsi Section, Shidian County: Niushiping Formation

Achulus var. *nanus* 1996 Gao Lianda, p.106

Ambitisporites avitus Hoffmeister 1959 1996 Gao Lianda, pl.1, 2

Ambitisporites sp. 1996 Gao Lianda, pl.1, 5

Ambitisporites sp. B Richardson et Ioannides 1973 1996 Gao Lianda, pl.1, 3

Amicosporites (*Synorisporites*) cf. *lobatus* (Rodriguez) Steemans, 1989 1996 Gao Lianda, pl.1, 8

Amicosporites (*Synorisporites*) *lobatus* (Rodriguez) Steemans, 1989 1996 Gao Lianda, pl.1, 9

Amicosporites jonkers (Riegel) Steemans, 1989 1996 Gao Lianda, pl.1, 6, 11

Amicosporites lebatus 1996 Gao Lianda, p.106

Amicosporites miserabilis Cramer, 1966 1996 Gao Lianda, pl.1, 13

Apiculiretusispora spicula Richardson et Lister, 1969 1996 Gao Lianda, pl.1, 21

Archaeozonotriletes chulus (Cramer) var. *chulus* Richardson et Lister, 1969 1996 Gao Lianda, pl.1, 22

Brochotriletes rarus Arkhangelskaya, 1978 1996 Gao Lianda, pl.1, 36

Brochotriletes? *foveotatus* Naumova, 1953 1996 Gao Lianda, pl.1, 37

Chelimospora cassicula 1996 Gao Lianda, p.105

Chelimospora retorrida Toenau, 1986 1996 Gao Lianda, pl.1, 34

Clivorispora verrucata McGregor var. *verrucata* McGregor, 1973 1996 Gao Lianda, pl.1, 27

Cymbosporites downtonensis 1996 Gao Lianda, p.106

Cymbosporites echinatus Richardson et Lister, 1969 1996 Gao Lianda, pl.1, 30

Cymbosporites rarispinosus Steemans, 1989 1996 Gao Lianda, pl.1, 32

Dictyotriletes richardosnii Steemans, 1989 1996 Gao Lianda, pl.1, 38

Emphanisporites neglectus 1996 Gao Lianda, p.105

Iberospora glabella Cremer, 1966 1996 Gao Lianda, pl.1, 42

Iberospora mariae 1996 Gao Lianda, p.106

Iberospora nonispissatosa Steemans, 1989 1996 Gao Lianda, pl.2, 1

Retusotriletes cf. *goensis* Lele et Streel, 1969 1996 Gao Lianda, pl.2, 7

Retusotriletes dittonensis 1996 Gao Lianda, p.105

Retusotriletes downtonensis 1996 Gao Lianda, p.105

Streelispora granulata Richardson et Lister, 1969 1996 Gao Lianda, pl.2, 13-14

Streelispora newportensis (Chaloner et Streel) Richardson et Lister, 1969 1996 Gao Lianda, pl.2, 15

Synorisporites dittomensis 1996 Gao Lianda, p.105
Synorisporites libycus 1996 Gao Lianda, p.105
Synorisporites verrucatus Richardson et Lister, 1969 1996 Gao Lianda, pl.2, 9, 11

Sichuan

10 km north of Chaotianyi, Guangyuan City: Upper Jintaiguan Formation to Lower Chejiaba Formation
Ambitisporites dilutus (Hoffmeister) Richardson et Lister, 1969 2005 Wang Yi et al., pl.2, 9, 12
Aneurospora sp. 2005 Wang Yi et al., pl.2, 10-11
Artemopyra brevicosta Burgess et Richardson, 1991 2005 Wang Yi et al., pl.1, 9-11
Dactylofusa cabotti (Cramer) Fensome et al., 1990 2005 Wang Yi et al., pl.2, 13
Dyadospora murusattenuata Strother et Traverse, emend. Burgess et Richardson, 1991 2005 Wang Yi et al., pl.1, 4-6
Dyadospora murusdensa Strother et traverse, emend. Burgess et Richardson, 1991 2005 Wang Yi et al., pl.1, 12
Hispanaediscus verrucatus Cramer, emend. Burgess et Richardson, 1991 2005 Wang Yi et al., pl.1, 15
Laevitubulus plicatus Burgess et Edwards, 1991 2005 Wang Yi et al., pl.2, 14
Laevolancis divellomedia (Chibrikova) Burgess et Richardson, 1991 2005 Wang Yi et al., pl.1, 7-8; pl.2, 6
Pseudodyadospora laevigata Johnson, 1985 2005 Wang Yi et al., pl.1, 13
Pseudodyadospora petasus Wellman et Richardson, 1993 2005 Wang Yi et al., pl.1, 14
Retusotriletes cf. *minor* Kedo, 1963 2005 Wang Yi et al., pl.2, 7
Retusotriletes cf. *warringtonii* Richardson et Lister, 1969 2005 Wang Yi et al., pl.2, 2
Retusotriletes sp. A. 2005 Wang Yi et al., pl.2, 3
Retusotriletes sp. C. 2005 Wang Yi et al., pl.2, 8
Retusotriletes sp.B 2005 Wang Yi et al., pl.2, 4
Scyalospora downiei Bugress et Richardson, 1995 2005 Wang Yi et al., pl.2, 1
Scyalospora scripta Burgess et Richardson, 1995 2005 Wang Yi et al., pl.2, 5
Velatitetras laevigata Burgess, 1991 2005 Wang Yi et al., pl.1, 1-3

Pridoli

Yunnan

Qujing: Yulongsi Formation
Ambitisporites avitus 1994 Fang et al., p. 82; 1995 Cai Chongyang et al., p. 13
Ambitisporites dilutes 1994 Fang et al., p. 82; 1995 Cai Chongyang et al., p. 13
Ambitisporites sp. B “Richardson and Ioannides” 1994 Fang et al., p. 82; 1995 Cai Chongyang et al., p. 13
Apiculiretusispora specula 1994 Fang et al., p. 82; 1995 Cai Chongyang et al., p. 13
Apiculiretusispora synorea 1994 Fang et al., p. 82; 1995 Cai Chongyang et al., p. 13
Archaeozonotriletes chulus var. *chulus* 1994 Fang et al., p. 82; 1995 Cai Chongyang et al., p. 13
Archaeozonotriletes splendidus 1994 Fang et al., p. 82; 1995 Cai Chongyang et al., p. 13
Archaeozonotriletes suberatara 1994 Fang et al., p. 82; 1995 Cai Chongyang et al., p. 13
Brochotriletes rarus 1994 Fang et al., p. 82; 1995 Cai Chongyang et al., p. 13
Coronospora mariae 1994 Fang et al., p. 82; 1995 Cai Chongyang et al., p. 14

Cymbosporites catilius 1994 Fang et al., p. 82; 1995 Cai Chongyang et al., p. 14
Cymbosporites dittonensis 1994 Fang et al., p. 82; 1995 Cai Chongyang et al., p. 14
Emphanisporites neglectus 1994 Fang et al., p. 82; 1995 Cai Chongyang et al., p. 13
Retusotriletes abundo 1994 Fang et al., p. 82; 1995 Cai Chongyang et al., p. 13
Retusotriletes aureoladus 1994 Fang et al., p. 82; 1995 Cai Chongyang et al., p. 13
Retusotriletes dittonensis 1994 Fang et al., p. 82; 1995 Cai Chongyang et al., p. 13
Retusotriletes dubiosus 1994 Fang et al., p. 82; 1995 Cai Chongyang et al., p. 13
Retusotriletes minor 1994 Fang et al., p. 82; 1995 Cai Chongyang et al., p. 13
Retusotriletes warringtonii 1994 Fang et al., p. 82; 1995 Cai Chongyang et al., p. 13
Streelispora granulata 1994 Fang et al., p. 82; 1995 Cai Chongyang et al., p. 13
Streelispora newportensis 1994 Fang et al., p. 82; 1995 Cai Chongyang et al., p. 13
Synorisporites verrucatus 1994 Fang et al., p. 82; 1995 Cai Chongyang et al., p. 13

Lochkovian

Yunnan

1. Qujing: Xiaxishancun Formation

Ambitisporites avitus 1994 Fang Zongjie et al., p.86
Ambitisporites cf. *dilutus* 1994 Fang Zongjie et al., p.85
Ambitisporites dilutus 1994 Fang Zongjie et al., p.86
Anapiculatisporites petilus 1994 Fang Zongjie et al., p.85
Apiculiretusispora plicata 1994 Fang Zongjie et al., p.85
Apiculiretusispora qujingensis Gao, 1983 1983b Gao Lianda, pl.107, 14
Apiculiretusispora sp. 1994 Fang Zongjie et al., p.85
Apiculiretusispora spicula 1994 Fang Zongjie et al., p.85
Archaeozonotriletes chulus var. *chulus* 1994 Fang Zongjie et al., p.85
Archaeozonotriletes chulus var. *nanus* 1994 Fang Zongjie et al., p.86
Brochotriletes sanpetrensis 1994 Fang Zongjie et al., p.86
Brochotriletes sp. 1994 Fang Zongjie et al., p.85
Brochotriletes xishanensis Gao, 1983 1983b Gao Lianda, pl.110, 2
Camptozonotriletes sp. 1994 Fang Zongjie et al., p.86
Chelinospora cassicula 1994 Fang Zongjie et al., p.85
Clivosisporites verrucata var. *verrucata* 1994 Fang Zongjie et al., p.85
Cymbosporites dittonensis 1994 Fang Zongjie et al., p.85
Cymbosporites proteus 1994 Fang Zongjie et al., p.85
Cymbosporites verrucatus 1994 Fang Zongjie et al., p.85
Emphanisporites neglectus 1994 Fang Zongjie et al., p.86
Emphanisporites sp. 1994 Fang Zongjie et al., p.85
Leiotriletes furcatus Naumova, 1953 1983b Gao Lianda, pl.106, 3
Punctatisporites regosus Gao, 1983 1983b Gao Lianda, pl.106, 11
Punctatisporites sp. 1 1983b Gao Lianda, pl.106, 10
Punctatisporites sp. 3 1983b Gao Lianda, pl.106, 15
Retusotriletes cf. *warringronii* 1994 Fang Zongjie et al., p.85

Retusotriletes dittonensis 1994 Fang Zongjie et al., p.85
Retusotriletes laevis 1994 Fang Zongjie et al., p.85
Retusotriletes minor 1994 Fang Zongjie et al., p.85
Retusotriletes triangulates 1994 Fang Zongjie et al., p.85
Stenozonotriletes extensus Naumova, 1953 1983b Gao Lianda, pl.110, 16
Streelispora newportensis 1994 Fang Zongjie et al., p.85
Synorisporites cf. *verrucatus* 1994 Fang Zongjie et al., p.85
Synorisporites labutus 1994 Fang Zongjie et al., p.85

2. Qujing: Xitun Formation
Punctatisporites subminor (Naumova) Gao and Hou 1981 Gao Lianda, pl. I, 3

3. Xiangyangsi Section, Shidian County: Xiangyangsi Formation
Apiculatasporites microconus 1996 Gao Lianda, p.106
Ambitisporites dilutus (Hoffmeister) Richardson et Lister, 1969 1996 Gao Lianda, pl.1, 1
Amicosporites sp. A 1996 Gao Lianda, pl.1, 7
Amicosporites sp. B 1996 Gao Lianda, pl.1, 10
Anapiculatisporites picatus Cramer, 1966 1996 Gao Lianda, pl.1, 14
Apiculiretusispora plicata (Allen) Streel, 1967 1996 Gao Lianda, pl.1, 20
Apiculiretusispora pygmaea McGregor, 1973 1996 Gao Lianda, pl.1, 17-18
Archaeozonotriletes chulus var. *chulus* 1996 Gao Lianda, p.106
Archaeozonotriletes chulus var. *nanus* 1996 Gao Lianda, p.106
Breconisporites breconensis Richardson et al. 1996 Gao Lianda, pl.1, 25
Brochotriletes cf. *foveolatus* 1996 Gao Lianda, p.106
Cymbosporites mixtornatus Steemans, 1989 1996 Gao Lianda, pl.1, 33
Dibolisporites sp. 1996 Gao Lianda, pl.1, 35
Emphanisporites neglectus 1996 Gao Lianda, p.106
Retusotriletes cf. *warringtonii* Richardson et Lister, 1969 1996 Gao Lianda, pl.2, 6
Retusotriletes downtonensis Richardson Lister, 1969 1996 Gao Lianda, pl.2, 3
Retusotriletes sp. 1996 Gao Lianda, pl.2, 4
Retusotriletes warringtonii Richardson Lister, 1969 1996 Gao Lianda, pl.2, 2
Streelispora newportensis (Chaloner et Streel) Richardson et Lister, 1969 1996 Gao Lianda, pl.2, 15
Streetiapora granulate 1996 Gao Lianda, p.106
Synorisporites verrucatus Richardson et Lister, 1969 1996 Gao Lianda, pl.2, 9, 11

Sichuan

Ganxi Section, Beichuan County, Mianyang City: Guixi Formation; Mu'erchang Formation; Guanyinmiao Formation
Dictyotriletes contortus Gao, 1988 1988 Gao, pl. 145, 19-22, 29
Synorisporites similverrucatus Gao, 1988 1988 Gao, pl. 147, 13, 14

Pragian

Sichuan

Ganxi Section, Beichuan County, Mianyang City: Guixi Formation; Mu'erchang Formation; Guanyinmiao Formation

Amocosporites glomeratus Gao, 1988 1988 Gao, pl. 150, 5, 6
Anapiculatisporites hispidus Gao, 1988 1988 Gao, pl. 143, 15
Anapiculatisporites minutus Gao, 1988 1988 Gao, pl. 143, 17, 18
Anapiculatisporites mistusus Gao, 1988 1988 Gao, pl. 143, 14
Anapiculatisporites subsidiaries (Chibrikova) Gao, 1988 1988 Gao, pl. 143, 19
Apiculatisporis papillatus Gao, 1988 1988 Gao, pl. 143, 21, 23
Apiculiretusispora micromanifesta var. *limbata* (Naumova) Gao, 1988 1988 Gao, pl. 143, 4, 5, 9
Apiculiretusispora pachylosa Gao, 1988 1988 Gao, pl. 143, 10
Apiculiretusispora visendus var. *ciliolate* (Chibrikova) Gao, 1988 1988 Gao, pl. 143, 8, 13
Brochotriletes cavernus Gao, 1988 1988 Gao, pl. 146, 5, 6
Brochotriletes favosus (McGregor and Camfield) Gao, 1988 1988 Gao, pl. 146, 11
Chelinospora reclinata Gao, 1988 1988 Gao, pl. 147, 6, 7
Chelinospora simiconvoluta Gao, 1988 1988 Gao, pl. 147, 2, 3, 4
Convolutispora longmenshanensis Gao 1987 1988 Gao, pl. 145, 4, 10
Cymbosporites notans Gao, 1988 1988 Gao, pl. 148, 22
Cymbosporites spissatus Gao, 1988 1988 Gao, pl. 149, 1, 2
Cymbosporites stimulates Gao, 1988 1988 Gao, pl. 149, 3
Dibolisporites ancylosus Gao, 1988 1988 Gao, pl. 144, 19
Dibolisporites dumetornus Gao, 1988 1988 Gao, pl. 144, 10
Dibolisporites erectus Gao, 1988 1988 Gao, pl. 144, 12, 13
Dibolisporites horridus Gao, 1988 1988 Gao, pl. 144, 15
Dictyotriletes camutus Gao, 1988 1988 Gao, pl. 145, 15, 16, 18
Dictyotriletes contortus Gao, 1988 1988 Gao, pl. 145, 19-22, 29
Lophozonotriletes inflatus Gao, 1988 1988 Gao, pl. 147, 25
Raistrickia laciniata Gao, 1988 1988 Gao, pl. 147, 15
Synorisporites convolutus Gao, 1988 1988 Gao, pls. 147, 16, 17; 153, 6
Synorisporites longmenshanensis Gao, 1988 1988 Gao, pl. 148, 9, 10, 11
Synorisporites purus Gao, 1988 1988 Gao, pl. 148, 7, 8
Tholisporites minutus Gao, 1988 1988 Gao, pl. 149, 17, 18, 19
Thymospora commutate (Chibrikova) Gao, 1988 1988 Gao, pl. 150, 21
Thymospora microtuberculata (Chibrikova) Gao, 1988 1988 Gao, pl. 150, 20
Verrucosisporites cirratusa Gao, 1988 1988 Gao, pls. 144, 22; 145, 2
Verrucosisporites concaternatus Gao, 1988 1988 Gao, pl. 144, 7
Verrucosisporites evlanensis (Naumova) Gao, 1988 1988 Gao, pl. 144, 2, 6

Yunnan

1. Xiangyangsi Section, Shidian County: Lower Wangjiacun Formation

Amicosporites sp. C 1996 Gao Lianda, pl.1, 12
Apicularetusispora plicata 1996 Gao Lianda, p.106
Apiculatasporites microconus 1996 Gao Lianda, p.106

Archaeozonotriletes chulus var. *chulus* 1996 Gao Lianda, p.106
Archaeozonotriletes sp. 1996 Gao Lianda, pl.1, 23
Breconisporites breconensis 1996 Gao Lianda, p.106
Cymbosporites downtonensis Richardson et Lister, 1969 1996 Gao Lianda, pl.1, 28-29
Dibolisporites sp. 1996 Gao Lianda, p.106
Retusotriletes dittonensis 1996 Gao Lianda, p.106
Retusotriletes downtonensis Richardson Lister, 1969 1996 Gao Lianda, pl.2, 3
Retusotriletes warringtonii 1996 Gao Lianda, p.106
Synorisporites libycus Richardson et Lister, 1969 1996 Gao Lianda, pl.2, 12
Synorisporites verrucatus Richardson et Lister, 1969 1996 Gao Lianda, pl.2, 10

2. Gumu Section, Wenshan City: Posongchong Formation

Apiculiretusispora plicata (Allen) Streel, 1967 1994 Wang Yi, pl.II, 1-3
Apiculiretusispora pygmaea McGregor, 1973 1994 Wang Yi, pl.II, 19-21
Apiculiretusispora wenshanensis 1994 1994 Wang Yi, pl.II, 14, 15, 17
Brochotriletes sp. B. McGregor, 1973 1994 Wang Yi, p.321
Brochotriletes? *foveolatus* Naumova, 1953 1994 Wang Yi, p.321
Calamospora cf. *microrugsa* (Ibr.) S. W. et B., 1944 1994 Wang Yi, pl.I, 1-2
Calamospora cf. *panuncea* Richardson, 1965 1994 Wang Yi, pl.I, 4-5
Camptozonotriletes cf. *caperatus* McGregor, 1973 1994 Wang Yi, pl.II, 4
Crissisporites guangxiensis Gao, 1978 1994 Wang Yi, pl.I, 19
Cyclogranisporites sp. 1994 Wang Yi, p.321
Cymbosporites echinatus Richardson et Lister, 1969 1994 Wang Yi, p.321
Cymbosporites raistrickiaeformis (Schultz) Steemens 1982 1994 Wang Yi, pl.I, 13
Dibolisporites echinaceus (Eisenack) Richardson, 1965 1994 Wang Yi, pl.I, 23
Dibolisporites eifeliensis (Lanninger）McGregor 1968 1994 Wang Yi, pl.I, 21
Dictyotriletes emsiensis (Allen) McGregor, 1973 1994 Wang Yi, pl.II, 10-11
Dictyotriletes gorgoneus Cramer, 1967 1994 Wang Yi, pl.II, 9
Dictyotriletes subgranifer McGregor, 1973 1994 Wang Yi, pl.II, 7
Emphanisporites cf. *decoratus* Allen, 1965 1994 Wang Yi, pl.II, 23
Emphanisporites cf. *neglectus* Virgran, 19641994 Wang Yi, pl.II, 24
Lophotriletes sp. 1994 Wang Yi, pl.II, 12
Punctatisporites sp. 1994 Wang Yi, pl.I, 18
Raistrickia sp. 1994 Wang Yi, pl.I, 20
Retusotriletes cf. *triangulates* (Streel) Streel, 1967 1994 Wang Yi, pl.I, 9
Retusotriletes rotundus (Streel) Streel, 1967 1994 Wang Yi, pl.I, 3
Retusotriletes warringtonii Richardson et Lister, 1969 1994 Wang Yi, pl.I, 6-8
Verrucosisporites polygonalis Lanninger, 1968 1994 Wang Yi, pl.II, 5, 6

3. Qujing: Guijiatun Formation

Crissisporites guangxiensis Gao 1981 Gao Lianda, pl. II, 11
Emphanisporites neglectus Vigran. 1981 Gao Lianda, pl. II, 5

4. Cuifeng Mountain Section, Qujing City: Guijiatun Formation

?*Emphanisporites* sp. 1984 Gao Lianda, pl. II, 12
Acinosporites sp. 1984 Gao Lianda, pl. I, 27
Apiculiretusispora acuminate Gao, 1984 1984 Gao Lianda, pl. I, 24, 31
Apiculiretusispora cf. *plicata* (Allen) Streel 1984 Gao Lianda, pl. II, 1-2
Apiculiretusispora cf. *specula* Richardson et Lister 1984 Gao Lianda, pl. II, 8
Apiculiretusispora minuta Gao, 1984 Gao Lianda, pl. I, 21-22
Apiculiretusispora polygonalis McGregor 1984 Gao Lianda, pl. I, 23
Apiculiretusispora sp. A 1984 Gao Lianda, pl. I, 26, 28-80
Apiculiretusispora sp. B 1984 Gao Lianda, pl. II, 3-4
Apiculiretusispora sp. C 1984 Gao Lianda, pl. II, 5-7
Crissisporites guangxiensis Gao, 1984 Gao Lianda, pl. II, 20
Crissisporites minutus Gao, 1984 Gao Lianda, pl. II, 10
Cyclogranisporites qujingensis Gao, 1984 1984 Gao Lianda, pl. I, 18, 25
Dictyotidium sp. 1984 Gao Lianda, pl. II, 29
Emphanisporites minutus Allen 1984 Gao Lianda, pl. II,14
Emphanisporites neglectus Vigran 1984 Gao Lianda, pl. II, 13
Leiosphaeridium sp. 1984 Gao Lianda, pl. II, 22, 30
Leiotriletes pullatus Naum. 1984 Gao Lianda, pl. I, 1
Leiotriletes trivilis Naum. 1984 Gao Lianda, pl. I, 2
Lophosphaeridium pilosus Downie 1984 Gao Lianda, pl. II, 23
Lophosphaeridium retus Gao, 1984 1984 Gao Lianda, pl. II, 26
Lophosphaeridium sp. 1984 Gao Lianda, pl. II, 24
Micrhystidium cf. *raspa* (Cramer) Deflandre 1984 Gao Lianda, pl. II, 25
Punctatisporites minor (Naum.) Gao, 1984 Gao Lianda, pl. I, 17
Punctatisporites sp. 1984 Gao Lianda, pl. I, 16
Retusotriletes cf. *dubuis* (Eisenack) Richardson 1984 Gao Lianda, pl. I, 9, 13
Retusotriletes cf. *warringtonii* Richardson et Lister 1984 Gao Lianda, pl. I, 10
Retusotriletes communis Naum. 1984 Gao Lianda, pl. I, 5-6
Retusotriletes dittonensis Richardson et Lister 1984 Gao Lianda, pl. I, 7-8
Retusotriletes major (Streel) Lu et Ouyang 1984 Gao Lianda, pl. I, 14-15
Retusotriletes simplex Naum. 1984 Gao Lianda, pl. I, 3-4
Retusotriletes triangulates (Streel) Streel 1984 Gao Lianda, pl. I, 11
Retusotriletes yunnanensis Gao, 1984 1984 Gao Lianda, pl. I, 12
Stenozonotriletes insessus Allen 1984 Gao Lianda, pl. II, 15
Streelispora newportensis Richardson et Lister 1984 Gao Lianda, pl. I, 19-20
Tholisporites chulus (Cramer) McGregor var. *chulus* McGregor 1984 Gao Lianda, pl. II, 16-17
Tholisporites chulus (Cramer) McGregor var. *oanus* McGregor 1984 Gao Lianda, pl. II, 18-19
Tholisporites sp. 1984 Gao Lianda, pl. II, 9, 21
Trichysphaeridium sp. 1984 Gao Lianda, pl. II, 27-28

5. Xujiachong Section, Qujing City; Longhuashan Section, Zhanyi County: Xujiachong Formation

Anapiculatisporites minutus Lu et Ouyang, 1976 1976 Lu Lichang et Ou Yangshu, pl.3, 9-10; 1980 Lu Lichang, pl.4, 1-2; pl.11, 11
Apiculiretusispora brandtii Streel 2005 Tian Jiajie et Zhu Huaicheng, p.312
Apiculiretusispora cf. *granulata* Owens 1980 Lu Lichang, pl.11, 6-8

Apiculiretusispora cf. *gsapiensis* McGregor 2005 Tian Jiajie et Zhu Huaicheng, p.312
Apiculiretusispora conica Lu et Ouyang, 1976 1976 Lu Lichang et Ou Yangshu, pl.2, 25-27
Apiculiretusispora golatensis (Staplin) Lu et Ouyang, 1976 1976 Lu Lichang et Ou Yangshu, pl.2, 28-31; 2005 Tian Jiajie et Zhu Huaicheng, p.312
Apiculiretusispora granulata Owens 1980 Lu Lichang, pl.11, 1-5, 9
Apiculiretusispora minuta Lu et Ouyang, 1976 1976 Lu Lichang et Ou Yangshu, pl.2, 21-24
Apiculiretusispora nitida Owens 2005 Tian Jiajie et Zhu Huaicheng, p.312
Apiculiretusispora plicata (Allen) Streel 1976 Lu Lichang et Ou Yangshu, pl.2, 18-20; 2005 Tian Jiajie et Zhu Huaicheng, p.312
Apiculiretusispora sp.1 1983b Gao Lianda, pl.107, 11
Archaeozonotriletes chulus (Cramer) var. *chulus* Richardson et Lister 2005 Tian Jiajie et Zhu Huaicheng, p.312
Calamospora cf. *pannucea* Richardson 1976 Lu Lichang et Ou Yangshu, pl.1, 9, 10
Camarozonotriletes cf. *parvus* Owens 1976 Lu Lichang et Ou Yangshu, pl.3, 20-21
Cymbosporites dittonensis Richardson et Lister 1976 Lu Lichang et Ou Yangshu, pl.3, 17-19; 2005 Tian Jiajie et Zhu Huaicheng, p.312
Cymbosporites sp. 1980 Lu Lichang, pl.11, 19
Dibolisporites cf. *favosus* McGregor et Camfield 2005 Tian Jiajie et Zhu Huaicheng, p.312
Dibolisporites cf. *variegates* McGregor 2005 Tian Jiajie et Zhu Huaicheng, p.312
Dibolisporites quebecensis McGregor 2005 Tian Jiajie et Zhu Huaicheng, p.312
Dibolisporites wetteldorfensis Lanninger 2005 Tian Jiajie et Zhu Huaicheng, p.312
Dictyotriletes sp. 1976 Lu Lichang et Ou Yangshu, pl.2, 17
Emphanisporites epicautus Richardson et Lister 1980 Lu Lichang, pl.11, 21-22
Geminospora cf. *lasius* var. *minor* (Naumova, 1953) Lu et Ouyang, 1976 1976 Lu Lichang et Ou Yangshu, pl.3, 14
Granulatisporites planiusculus (Luber) Playford 1980 Lu Lichang, pl.11, 12-13
Granulatisporites planiusculus (Luber) Playford 2005 Tian Jiajie et Zhu Huaicheng, p.312
Granulatisporites sp.1 1983b Gao Lianda, pl.107, 6
Leiotriletes crassus Lu, 1980 1980 Lu Lichang, pl.10, 1
Leiotriletes flexuosus Lu, 1980 1980 Lu Lichang, pl.10, 2
Leiotriletes ornatus Ishchenko 1976 Lu Lichang et Ou Yangshu, pl.1, 3, 4; 2005 Tian Jiajie et Zhu Huaicheng, p.312
Leiotriletes rotundus Naumova 1976 Lu Lichang et Ou Yangshu, pl.1, 5
Perotrilites laevigatus Lu, 1980 1980 Lu Lichang, pl.11, 23-24
Punctatisporites divisus Gao et Hou 1976 1976 Lu Lichang et Ou Yangshu, pl.1, 1, 2
Punctatisporites glabrimarginatus Owens 2005 Tian Jiajie et Zhu Huaicheng, p.312
Punctatisporites obesus (Loose) Potonié et Kremp, 1955 1976 Lu Lichang et Ou Yangshu, pl.1, 6
Punctatisporites sp. 1976 Lu Lichang et Ou Yangshu, pl.1, 8
Punctatisporites sp.2 1983b Gao Lianda, pl.106, 12
Retusotriletes cf. *delicatus* Lu, 1980 Lu Lichang, pl.10, 12
Retusotriletes cf. *distinctus* Richardson 1976 Lu Lichang et Ou Yangshu, pl.1, 17
Retusotriletes cf. *rugulatus* Riegel 2005 Tian Jiajie et Zhu Huaicheng, p.312
Retusotriletes communis Naumova 1976 Lu Lichang et Ou Yangshu, pl.1, 16
Retusotriletes confossus (Richardson) Lu et Ouyang, 1976 1976 Lu Lichang et Ou Yangshu, pl.1, 20-21; 2005 Tian Jiajie et Zhu Huaicheng, p.312
Retusotriletes delicatus Lu, 1980 1980 Lu Lichang, pl.10, 13
Retusotriletes distinctus Richardson, 1965 1983b Gao Lianda, pl.107, 1
Retusotriletes intergranulatus Lu et Ouyang, 1976 1976 Lu Lichang et Ou Yangshu, pl.2, 12-14

Retusotriletes pychovii Naumova 1976 Lu Lichang et Ou Yangshu, pl.1, 13-15; 1980 Lu Lichang, pl.1, 14-15; pl.10, 5-6
Retusotriletes reculitus Lu et Ouyang, 1976 1976 Lu Lichang et Ou Yangshu, pl.1, 18-19
Retusotriletes rotundus (Streel) Streel 2005 Tian Jiajie et Zhu Huaicheng, p.312
Retusotriletes rugulatus Riegel 1980 Lu Lichang, pl.10, 10-11
Retusotriletes simplex Naumova 1976 Lu Lichang et Ou Yangshu, pl.1, 11, 12; 1980 Lu Lichang, pl.1, 12-13; pl.10, 3-4
Retusotriletes sp.1 1983b Gao Lianda, pl.106, 18
Retusotriletes triangulatus (Streel) Streel var. *major* var. nov. 1976 Lu Lichang et Ou Yangshu, pl.1, 7; pl.2, 11; 1980 Lu Lichang, pl.1, 10, 11; pl.10, 8-9
Retusotriletes triangulatus (Streel) Streel var. *microtriangulatus* var. nov. 1976 Lu Lichang et Ou Yangshu, pl.2, 5-8; 1980 Lu Lichang, pl.1, 19; pl.10, 7
Retusotriletes triangulatus (Streel) Streel var. *triangulates* 1976 Lu Lichang et Ou Yangshu, pl.2, 1-4
Retusotriletes? sp. 1976 Lu Lichang et Ou Yangshu, pl.2, 15-16
Samarisporites sp. 2 1980 Lu Lichang, pl.11, 18
Stenozonotriletes extensus var. *major* Naumova 1976 Lu Lichang et Ou Yangshu, pl.3, 9
Stenozonotriletes extensus var. *medius* Naumova 1976 Lu Lichang et Ou Yangshu, pl.3, 7, 8
Stenozonotriletes interbaculus Lu, 1980 1980 Lu Lichang, pl.11, 16-17
Stenozonotriletes conformis Naumova, 1953 1983b Gao Lianda, pl.110, 17
Streelispora zhanyiensis Lu, 1980 1980 Lu Lichang, pl.11, 20
Tholisporites chulus (Cramer) McGregor var. *chulus* (Richardson et Lister) McGregor 1976 Lu Lichang et Ou Yangshu, pl.3, 10-13, 15; 1980 Lu Lichang, pl.11, 14-15
Tholisporites sp. 1976 Lu Lichang et Ou Yangshu, pl.3, 16
Verruciretusispora megaplatyverruca Lu et Ouyang, 1976 1976 Lu Lichang et Ou Yangshu, pl.3, 3-6; 1980 Lu Lichang, pl.11, 10
Verruciretusispora platyverruca Lu et Ouyang, 1976 1976 Lu Lichang et Ou Yangshu, pl.3, 1, 2
Verrucosisporites cf. *polygonalis* Lanninger 2005 Tian Jiajie et Zhu Huaicheng, p.312

6. Xujiachong−Xichong Section, northwest of Qujing City: Xujiachong Formation

?*Brochosporites rarus* Arkhangelskaya, 1978 2012 Wellman et al., fig. 4M
?*Dictyotriletes favosus* McGregor et Camfield, 1976 2012 Wellman et al., fig. 4L, 9I
Ambitisporites avitus Hoffmeister, 1959 2012 Wellman et al., fig. 4K
Aneurospora conica (Lu and Ouyang, 1976) Wellman et al., 2012 2012 Wellman et al., fig. 5A-F, 9K-L
Aneurospora xujiachongensis Wellman et al., 2012 2012 Wellman et al., fig. 5H-L, 9E-G
Apiculiretusispora brandtii Streel, 1964 2012 Wellman et al., fig. 3E
Apiculiretusispora plicata (Allen) Streel, 1967 2012 Wellman et al., fig. 3F-I, K, 9A
Apiculiretusispora sp. 2012 Wellman et al., fig. 9B
Apiculiretusispora sp. 2012 Wellman et al., fig. 3J
Archaeozonotriletes chulus (Cramer) Richardson et Lister, 1969 2012 Wellman et al., fig. 8C
Camptozonotriletes? *luii* Wellman et al., 2012 2012 Wellman et al., fig. 7A-H, 9J, M-O
Cheilotetras caledonica Wellman et Richardson, 1993 2012 Wellman et al., fig. 8P
Chelinospora ouyangii Wellman et al., 2012 2012 Wellman et al., fig. 8D-H
Dibolisporites cf. *echinaceous* (Eisenack) Richardson, 1965 2012 Wellman et al., fig. 4A-C, 9C-D
Dibolisporites sp. 2012 Wellman et al., fig. 9H
Dibolisporites sp. 2012 Wellman et al., fig. 4D-E
Dictyotriletes emsiensis? (Allen) McGregor, 1973 2012 Wellman et al., fig. 4I

Dictyotriletes sp. 2012 Wellman et al., fig. 4F
Dictyotriletes sp. A 2012 Wellman et al., fig. 4J
Emphanisporites sp. 2012 Wellman et al., fig. 8A-B
Latosporites ovalis Breuer et al., 2007 2012 Wellman et al., fig. 8K-M
Leiozonospora xichongensis Wellman et al., 2012 2012 Wellman et al., fig. 7I-N
Pseudodyadospora petasus Wellman et Richardson, 1993 2012 Wellman et al., fig. 8O
Retusotriletes cf . *rotundus* (Streel) Streel, 1967 2012 Wellman et al., fig. 3D
Retusotriletes cf. *triangulatus* (Streel) Streel, 1967 2012 Wellman et al., fig. 3A, B
Retusotriletes sp. A 2012 Wellman et al., fig. 3C
Tetrahedraletes medinensis Strother et Traverse, 1979 emend. Wellman et Richardson, 1993 2012 Wellman et al., fig. 8Q
Verrucosisporites megaplatyverruca Lu et Ouyang, 1976 2012 Wellman et al., fig. 6E, G, 10A
Verrucosisporites polygonalis Lanninger 1968 2012 Wellman et al., fig. 6A-D, F, H, 10C
Verrucosisporites sp. 2012 Wellman et al., fig. 10D

Guizhou

Hou'ershan and Lishan Section, Dushan County: Lower Danlin Formation
Acanthotriletes pyriformis Gao et Hou, 1975 1975 Gao Lianda et Hou Jingpeng, pl.4, 14-15; 1983b Gao Lianda, pl.108, 2
Apiculatisporites eximius (Naum.) Gao et Hou, 1975 1975 Gao Lianda et Hou Jingpeng, pl.5, 3a-b
Apiculatisporites sp. 1975 Gao Lianda et Hou Jingpeng, pl.5, 2a-b
Apiculiretusispora microperforatus (Tschibr.) Gao et Hou, 1975 1975 Gao Lianda et Hou Jingpeng, pl.3, 7
Archaeozonotriletes semilucensis Naum. 1975 Gao Lianda et Hou Jingpeng, pl.9, 10
Camarozonotriletes obtusus Naum. 1975 Gao Lianda et Hou Jingpeng, pl.9, 17
Cyclobaculisporites sp. 1975 Gao Lianda et Hou Jingpeng, pl.4, 7
Cyclogranisporites retisimilis Riegel, 1968 1983b Gao Lianda, pl.107, 8
Cyclogranisporites sp. 1975 Gao Lianda et Hou Jingpeng, pl.4, 6
Cyclogranisporites vulgaris (Kedo) Gao et Hou, 1975 1975 Gao Lianda et Hou Jingpeng, pl.4, 2
Diatomozonotriletes sp. 1975 Gao Lianda et Hou Jingpeng, pl.9, 13
Dictyotriletes gorgoneus Cramer 1975 Gao Lianda et Hou Jingpeng, pl.7, 9
Dictyotriletes grandis Lanninger 1975 Gao Lianda et Hou Jingpeng, pl.7, 12
Dictyotriletes microreticulatus Gao, 1983 1983b Gao Lianda, pl.110, 7
Dictyotriletes nigratus Naumova, 1953 1983b Gao Lianda, pl.110, 9
Dictyotriletes sp. 1 1983b Gao Lianda, pl.110, 8
Emphanisporites annulatus McGregor, 1961 1975 Gao Lianda et Hou Jingpeng, pl.8, 5; 1983b Gao Lianda, pl.110, 12
Emphanisporites neglectus Vigran 19641975 Gao Lianda et Hou Jingpeng, pl.8, 9b; 1983b Gao Lianda, pl.110, 11
Emphanisporites sp. B 1975 Gao Lianda et Hou Jingpeng, pl.8, 8
Granulatisporites minusculus (Naum.) Gao et Hou, 1975 1975 Gao Lianda et Hou Jingpeng, pl.3, 14
Laevigatosporites antiqus Moreau-Benoit 1975 Gao Lianda et Hou Jingpeng, pl.13, 6
Laevigatosporites minutus Gao et Hou, 1975 1975 Gao Lianda et Hou Jingpeng, pl.13, 8
Laevigatosporites sp. A 1975 Gao Lianda et Hou Jingpeng, pl.13, 5a-b
Punctatisporites micropunctatus Gao et Hou, 1975 1975 Gao Lianda et Hou Jingpeng, pl.1, 22
Punctatisporites subminor (Naum.) Gao et Hou, 1975 1975 Gao Lianda et Hou Jingpeng, pl.1, 17
Reticulatisporites cf. *minor* Naum. 1975 Gao Lianda et Hou Jingpeng, pl.7, 5

Reticulatisporites emsiensis Allen, 1965 1983b Gao Lianda, pl.110, 6
Retusotriletes inimitabilis Tschibr. 1975 Gao Lianda et Hou Jingpeng, pl.2, 11
Retusotriletes radiatus Gao et Hou, 1975 1975 Gao Lianda et Hou Jingpeng, pl.2, 17
Retusotriletes sterlibaschevensis var. *denticulatus* Tschibr. 1975 Gao Lianda et Hou Jingpeng, pl.2, 12a-b
Retusotriletes translaticius Tschibr. 1975 Gao Lianda et Hou Jingpeng, pl.2, 14
Retusotriletes trilobatus Gao et Hou, 1975 1975 Gao Lianda et Hou Jingpeng, pl.3, 2
Stenozonotriletes excurreus Gao et Hou, 1975 1975 Gao Lianda et Hou Jingpeng, pl.8, 21
Verrucosisporites cf. *sifati* (Ibr.) S. W. et B. 1975 Gao Lianda et Hou Jingpeng, pl.5, 14
Verrucososporites intortus Gao et Hou, 1975 1975 Gao Lianda et Hou Jingpeng, pl.13, 13a-c
Verrucososporites sp. 1975 Gao Lianda et Hou Jingpeng, pl.13, 15

Guangxi

1. Hengxian County: Nagaoling Formation

Apiculiretusispora nagaolingensis Gao 1981 Gao Lianda, pl. I, 8
Synorisporites cf. *verrucatus* Richardson et Lister 1981 Gao Lianda, pl. II, 2

2. Liujing : Nagaoling Formation

Acanthotriletes cuspidatus Gao, 1978 1978 Gao, pl. 44, 13
Acanthotriletes exsertus Gao, 1978 1978 Gao, pl. 44, 9
Acanthotriletes guangxiensis Gao, 1978 1978 Gao, pl. 44, 18
Apiculiretusispora minuta Gao, 1978 1978 Gao, pl. 43, 17
Apiculiretusispora nagaolingensis Gao, 1978 1978 Gao, pl. 44, 1
Crissisporites guangxiensis Gao, 1978 1978 Gao, pl. 45, 16-19
Crissisporites minutus Gao, 1978 1978 Gao, pl. 45, 15
Hymenozonotriletes antuqus Gao, 1978 1978 Gao, pl. 45, 11
Hymenozonotriletes deltoideus Gao, 1978 1978 Gao, pl. 45, 12
Hymenozonotriletes serrulatus Gao, 1978 1978 Gao, pl. 45, 13
Pterospermopsis minus Gao, 1978 1978 Gao, pl. 48, 8
Retusotriletes dilicatus Gao, 1978 1978 Gao, pl. 43, 10
Stenozonotriletes angulatus Gao, 1978 1978 Gao, pl. 45, 5
Stenozonotriletes inspissatus Gao, 1978 1978 Gao, pl. 45, 6
Verrucosisporites multiformis Gao, 1978 1978 Gao, pl. 44, 21
Veryhachium liujingensis Gao, 1978 1978 Gao, pl. 47, 7

Emsian

Yunnan

1. Luquan; Gumu Section, Wenshan City: Pojiao Formation

Acanthotriletes loratus Gao, 1983 1983b Gao Lianda, pl.108, 4
Acinosporites acanthomammillatus Richardson, 1965 1983b Gao Lianda, pl.108, 13
Ancyrospora simplex Guennel, 1963 1983b Gao Lianda, pl.112, 6
Apiculatisporis longispinosus Gao, 1983 1983b Gao Lianda, pl.108, 5

Apiculatisporis sp. 1983b Gao Lianda, pl.109, 6
Apiculiretusispora plicata (Allen) Streel, 1967 1983b Gao Lianda, pl.107, 9; 1994 Wang Yi, p.321
Apiculiretusispora wenshanensis, 1994 1994 Wang Yi, pl.II, 16, 18
Apiculiretusispora pygmaea McGregor, 1973 1994 Wang Yi, p.321
Apiculiretusispora colliculosa (Tschibreikova) Gao, 1983 1983b Gao Lianda, pl.107, 12
Apiculiretusispora idimornphusa (Tschibreikova) Gao, 1983 1983b Gao Lianda, pl.107, 10
Apiculiretusispora sp.2 1983b Gao Lianda, pl.107, 13
Archaeozonotriletes liukongqiaoensis Gao, 1983 1983b Gao Lianda, pl.110, 21
Archaeozonotriletes variabilis variabilis Naumova, 1953 1983b Gao Lianda, pl.111, 1-2
Archaeozonotriletes variabilis gigantus Gao, 1983 1983b Gao Lianda, pl.110, 24
Archaeozonotriletes sp. 1983b Gao Lianda, pl.110, 22-23
Brochotriletes sp. B. 1994 Wang Yi, pl.I, 10
Brochotriletes foveolatus minor Naumova, 1953 1983b Gao Lianda, pl.109, 12
Brochotriletes globosus Gao, 1983 1983b Gao Lianda, pl.109, 11
Brochotriletes retusus Gao, 1983 1983b Gao Lianda, pl.109, 13
Calamospora cf. *atava* (Naumova) McGregor, 1973 1983b Gao Lianda, pl.106, 7
Calamospora cf. *microrugosa* (Ibrahim) Schopf, Wilson et Bentall, 1944 1983b Gao Lianda, pl.106, 9
Calamospora nigrata (Naumova) Allen, 1965 1983b Gao Lianda, pl.106, 8
Calyptosporites velatus (Eisenack) Richardson 1962 1983b Gao Lianda, pl.111, 13
Camarozonotriletes obtusus Naumova, 1953 1983b Gao Lianda, pl.111, 6
Camptozonotriletes sextantii McGregor, 1973 1994 Wang Yi, pl.II, 13
Camptozonotriletes sp. G 1994 Wang Yi, pl.I, 12
Chelinospora sp. 1983b Gao Lianda, pl.109, 9; pl.110, 1
Cymbosporites echinatus Richardson et Lister, 1969 1994 Wang Yi, pl.I, 24
Cymbosporites raistrickiaeformis (Schultz) Steemens, 1982 1994 Wang Yi, pl.I, 14
Densosporites cf. *formosus* Artuz, 1957 1994 Wang Yi, pl.II, 22
Dibolisporites parvispinosus Gao, 1983 1983b Gao Lianda, pl.108, 6
Dibolisporites eifeliensis (Lanninger) McGregor 1968 1994 Wang Yi, pl.I, 22
Dibolisporites echinaceus (Eisenack) Richardson, 1965 1994 Wang Yi, p.321
Dictyotriletes subgranifer McGregor, 1973 1994 Wang Yi, pl.II, 8
Dictyotriletes gorgoneus Cramer, 1967 1994 Wang Yi, p.321
Emphanisporites robustus McGregor, 1961 1983b Gao Lianda, pl.110, 13
Emphanisporites yunnanensis Gao, 1983 1983b Gao Lianda, pl.110, 14
Emphanisporites sp. 1983b Gao Lianda, pl.110, 15
Endosporites hyalinus hyalinus (Naumova) Gao, 1983 1983b Gao Lianda, pl.111, 7
Geminospora compactus (Naumova) Gao, 1983 1983b Gao Lianda, pl.111, 3
Geminospora extensus (Naumova) 1983b Gao Lianda, pl.111, 4
Geminospora sp. 1983b Gao Lianda, pl.111, 5
Grandispora haikaoensis Gao, 1983 1983b Gao Lianda, pl.113, 1
Grandispora spinosa (Naumova) Gao, 1983 1983b Gao Lianda, pl.113, 3
Grandispora vera (Naumova) Gao, 1983 1983b Gao Lianda, pl.113, 4
Grandispora sp.1 1983b Gao Lianda, pl.113, 2
Hymenozonotriletes commutatus Naumova, 1953 1983b Gao Lianda, pl.111, 9
Hymenozonotriletes maoshanensis Gao, 1983 1983b Gao Lianda, pl.113, 6

Hymenozonotriletes proteus Naumova, 1953 1983b Gao Lianda, pl.111, 10
Hymenozonotriletes rectiformis Naumova, 1953 1983b Gao Lianda, pl.111, 8
Hymenozonotriletes striphnos Gao, 1983 1983b Gao Lianda, pl.111, 12
Hymenozonotriletes varius Naumova, 1953 1983b Gao Lianda, pl.111, 11
Hystricosporites delectabilis McGregor, 1960 1983b Gao Lianda, pl.112, 7
Laevigatosporites rarus (Naumova) Gao et Hou, 1975 1983b Gao Lianda, pl.113, 9
Laevigatosporites sp.1 1983b Gao Lianda, pl.113, 8
Leiotriletes devonicus Naumova, 1953 1983b Gao Lianda, pl.106, 5
Leiotriletes laevis Naumova, 1953 1983b Gao Lianda, pl.106, 1
Leiotriletes pullatus Naumova, 1953 1983b Gao Lianda, pl.106, 6
Leiotriletes sp.1 1983b Gao Lianda, pl.106, 4
Lophozonotriletes contextus Gao, 1983 1983b Gao Lianda, pl.108, 9
Punctatisporites sp. 1994 Wang Yi, p.321
Punctatisporites sp.3 1983b Gao Lianda, pl.106, 14
Radiatispinospora longispinosa Gao, 1983 1983b Gao Lianda, pl.109, 7
Radiatispinospora cf. *radiata* Bharadwaj 1971 1983b Gao Lianda, pl.109, 10
Radiatispinospora yunnanensis Gao, 1983 1983b Gao Lianda, pl.109, 8
Raistrickia baculata Gao, 1983 1983b Gao Lianda, pl.109, 5
Raistrickia serrulata Gao, 1983 1983b Gao Lianda, pl.109, 4
Raistrickia sp. 1994 Wang Yi, p.321
Reticulatisporites devonicus (Naumova) Gao, 1983 1983b Gao Lianda, pl.110, 3
Retusotriletes warringtonii Richardson et Lister, 1969 1994 Wang Yi, p.321
Retusotriletes rotundus (Streel) Streel, 1967 1994 Wang Yi, p.321
Retusotriletes communis major Schultz 1968 1983b Gao Lianda, pl.106, 19
Retusotriletes luquanensis Gao, 1983 1983b Gao Lianda, pl.107, 5
Retusotriletes maximus Gao, 1983 1983b Gao Lianda, pl.106, 17
Retusotriletes nitidus Gao, 1983 1983b Gao Lianda, pl.107, 3
Retusotriletes triangulatus (Streel) Streel, 1967 1983b Gao Lianda, pl.107, 4
Retusotriletes sp.2 1983b Gao Lianda, pl.107, 2
Rhabdosporites langii (Eisenack) Richardson 1960 1983b Gao Lianda, pl.113, 7
Spinozonotriletes unguisus (Tschibrikova) Gao, 1983 1983b Gao Lianda, pl.112, 2
Stenozonotriletes laevigatus Naumova, 1953 1983b Gao Lianda, pl.110, 18-19
Thymospora intortus Gao et Hou, 1975 1983b Gao Lianda, pl.113, 14
Thymospora microtuberculata (Tschibrikova) Gao et Hou, 1975 1983b Gao Lianda, pl.113, 12-13
Thymospora minus Gao et Hou, 1975 1983b Gao Lianda, pl.113, 10-11
Verruciretusispora microtuberculata (Schultz) Gao, 1983 1983b Gao Lianda, pl.109, 2-3
Verruciretusispora verrucosa Gao, 1983 1983b Gao Lianda, pl.109, 1
Verrucosisporites paremecus Gao, 1983 1983b Gao Lianda, pl.108, 14
Verrucosisporites sp. 1983b Gao Lianda, pl.108, 12

2. Xujiachong−Xichong Section, northwest of Qujing City: Xujiachong Formation

?*Brochosporites rarus* Arkhangelskaya, 1978 2012 Wellman et al., fig. 4M
?*Dictyotriletes favosus* McGregor et Camfield, 1976 2012 Wellman et al., fig. 4L, 9I
Ambitisporites avitus Hoffmeister, 1959 2012 Wellman et al., fig. 4K

Aneurospora conica (Lu and Ouyang, 1976) Wellman et al., 2012 2012 Wellman et al., fig. 5A-F, 9K-L
Aneurospora xujiachongensis Wellman et al., 2012 2012 Wellman et al., fig. 5H-L, 9E-G
Apiculiretusispora brandtii Streel, 1964 2012 Wellman et al., fig. 3E
Apiculiretusispora plicata (Allen) Streel, 1967 2012 Wellman et al., fig. 3F-I, K, 9A
Apiculiretusispora sp. 2012 Wellman et al., fig. 9B
Apiculiretusispora sp. 2012 Wellman et al., fig. 3J
Archaeozonotriletes chulus (Cramer) Richardson et Lister, 1969 2012 Wellman et al., fig. 8C
Camptozonotriletes? *luii* Wellman et al., 2012 2012 Wellman et al., fig. 7A-H, 9J, M-O
Cheilotetras caledonica Wellman et Richardson, 1993 2012 Wellman et al., fig. 8P
Chelinospora ouyangii Wellman et al., 2012 2012 Wellman et al., fig. 8D-H
Dibolisporites cf. *echinaceous* (Eisenack) Richardson, 1965 2012 Wellman et al., fig. 4A-C, 9C-D
Dibolisporites sp. 2012 Wellman et al., fig. 9H
Dibolisporites sp. 2012 Wellman et al., fig. 4D-E
Dictyotriletes emsiensis? (Allen) McGregor, 1973 2012 Wellman et al., fig. 4I
Dictyotriletes sp. 2012 Wellman et al., fig. 4F
Dictyotriletes sp. A 2012 Wellman et al., fig. 4J
Emphanisporites sp. 2012 Wellman et al., fig. 8A-B
Latosporites ovalis Breuer et al., 2007 2012 Wellman et al., fig. 8K-M
Leiozonospora xichongensis Wellman et al., 2012 2012 Wellman et al., fig. 7I-N
Pseudodyadospora petasus Wellman et Richardson, 1993 2012 Wellman et al., fig. 8O
Retusotriletes cf . *rotundus* (Streel) Streel, 1967 2012 Wellman et al., fig. 3D
Retusotriletes cf. *triangulatus* (Streel) Streel, 1967 2012 Wellman et al., fig. 3A, B
Retusotriletes sp. A 2012 Wellman et al., fig. 3C
Tetrahedraletes medinensis Strother et Traverse, 1979 emend. Wellman et Richardson, 1993 2012 Wellman et al., fig. 8Q
Verrucosisporites megaplatyverruca Lu et Ouyang, 1976 2012 Wellman et al., fig. 6E, G, 10A
Verrucosisporites polygonalis Lanninger 1968 2012 Wellman et al., fig. 6A-D, F, H, 10C
Verrucosisporites sp. 2012 Wellman et al., fig. 10D

Guizhou

Hou’ershan Section and Lishan Section, Dushan County; Baoyang Section, Duyun City: Shujiaping Formation

Acanthotriletes longispinosus Moreau-Benoit 1975 Gao Lianda et Hou Jingpeng, pl.4, 8
Acanthotriletes perpusillus Naum. 1975 Gao Lianda et Hou Jingpeng, pl.4, 10-11
Acanthotriletes sp. 1975 Gao Lianda et Hou Jingpeng, pl.4, 12
Acanthotriletes tenuispinosus Naum. 1975 Gao Lianda et Hou Jingpeng, pl.4, 13
Acinosporites cf. *acanthomannillatus* Richardson 1975 Gao Lianda et Hou Jingpeng, pl.8, 11
Ancyrospora aduncus (Naum.) Gao et Hou, 1975 1975 Gao Lianda et Hou Jingpeng, pl.10, 10
Ancyrospora grandispinosa Richardson 1975 Gao Lianda et Hou Jingpeng, pl.11, 3-4
Ancyrospora larvatus (Naum.) Gao et Hou, 1975 1975 Gao Lianda et Hou Jingpeng, pl.11, 5
Ancyrospora simplex Guennnel 1975 Gao Lianda et Hou Jingpeng, pl.10, 12
Ancyrospora sp. A 1975 Gao Lianda et Hou Jingpeng, pl.10, 11
Ancyrospora sp. B 1975 Gao Lianda et Hou Jingpeng, pl.10, 13

Apiculatisporites eximius (Naum.) Gao et Hou, 1975 1975 Gao Lianda et Hou Jingpeng, pl.5, 3a-b

Apiculatisporites microaenus Richardson 1975 Gao Lianda et Hou Jingpeng, pl.5, 5

Apiculatisporites rigidispinus Gao et Hou, 1975 1975 Gao Lianda et Hou Jingpeng, pl.5, 4a-b

Apiculiretusispora subgibberosus Naum. var. *capitellatus* (Tschibr.) Gao et Hou, 1975 1975 Gao Lianda et Hou Jingpeng, pl.3, 11

Archaeozonotriletes sp. B 1975 Gao Lianda et Hou Jingpeng, pl.9, 20

Bullatisporites bullatus Richardson, 1965 1975 Gao Lianda et Hou Jingpeng, pl.6, 7; 1983b Gao Lianda, pl.108, 15

Calamospora divisa Gao et Hou, 1975 1975 Gao Lianda et Hou Jingpeng, pl.1, 11

Calamospora mitosobuobuta Gao et Hou, 1975 1975 Gao Lianda et Hou Jingpeng, pl.1, 15

Calamospora pannuces Richardson 1975 Gao Lianda et Hou Jingpeng, pl.1, 12

Calamospora plana Gao et Hou, 1975 1975 Gao Lianda et Hou Jingpeng, pl.1, 9a-b

Calyptosporites velatus (Eisenack) Richardson 1975 Gao Lianda et Hou Jingpeng, pl.12, 3

Camarazonotriletes obtusus Naumova 1981 Gao Lianda, pl. III, 13

Cirratriradites sp. B 1975 Gao Lianda et Hou Jingpeng, pl.9, 5a-b

Corystisporites sp. 1975 Gao Lianda et Hou Jingpeng, pl.11, 7

Cyclogranisporites retisimilis Riegel 1975 Gao Lianda et Hou Jingpeng, pl.4, 4

Cyclogranisporites rugosus (Naum.) Gao et Hou, 1975 1975 Gao Lianda et Hou Jingpeng, pl.4, 3

Diatomozonotriletes acuminatus Gao et Hou, 1975 1975 Gao Lianda et Hou Jingpeng, pl.9, 16

Diatomozonotriletes devonicus Naum. var. *azonatus* Tschibr. 1975 Gao Lianda et Hou Jingpeng, pl.9, 14a-c

Diatomozonotriletes oligodontus Tschibr. 1975 Gao Lianda et Hou Jingpeng, pl.9, 15

Dibolisporites eifeliensis (Lanninger) McGregor 1975 Gao Lianda et Hou Jingpeng, pl.4, 17

Dibolisporites sp. 1975 Gao Lianda et Hou Jingpeng, pl.4, 18

Dictyotriletes sp. 1975 Gao Lianda et Hou Jingpeng, pl.7, 16-17

Emphanisporites annulatus McGregor 1981 Gao Lianda, pl. II, 7

Emphanisporites minutus Allen 1975 Gao Lianda et Hou Jingpeng, pl.8, 9a

Emphanisporites sp. A 1975 Gao Lianda et Hou Jingpeng, pl.8, 6

Emphanisporites sp. C 1975 Gao Lianda et Hou Jingpeng, pl.8, 10

Grandispora sp. 1975 Gao Lianda et Hou Jingpeng, pl.12, 2

Granulatisporites magnus (Naum.) Gao et Hou, 1975 1975 Gao Lianda et Hou Jingpeng, pl.3, 16

Granulatisporites minusculus (Naum.) Gao et Hou, 1975 1975 Gao Lianda et Hou Jingpeng, pl.3, 14

Hymenozonotriletes cf. *varius* Naum. 1975 Gao Lianda et Hou Jingpeng, pl.10, 1

Hymenozonotriletes rarus Naum. 1975 Gao Lianda et Hou Jingpeng, pl.10, 9

Hymenozonotriletes spinulosus Naum. 1975 Gao Lianda et Hou Jingpeng, pl.10, 4-5

Laevigatosporites commutatus (Tschibr.) Gao et Hou, 1975 1975 Gao Lianda et Hou Jingpeng, pl.13, 4a-b

Leiotriletes cf. *priddyi* (Berry) Pot. et Kr. 1975 Gao Lianda et Hou Jingpeng, pl.1, 7

Leiotriletes confertus McGregor 1975 Gao Lianda et Hou Jingpeng, pl.1, 3

Leiotriletes sp. 1975 Gao Lianda et Hou Jingpeng, pl.1, 6

Lophotriletes sp. 1975 Gao Lianda et Hou Jingpeng, pl.4, 16

Lophozonotriletes sp. A 1975 Gao Lianda et Hou Jingpeng, pl.5, 6

Microreticulatisporites subtriangulatus Gao et Hou, 1975 1975 Gao Lianda et Hou Jingpeng, pl.7, 4

Punctatisporites nitidus Hoff. Stapl. et Mally. 1975 Gao Lianda et Hou Jingpeng, pl.1, 16

Punctatisporites solidus (Naumova) Gao et Hou, 1975 1975 Gao Lianda et Hou Jingpeng, pl.1, 18-19; 1983b Gao Lianda, pl.106, 13

Punctatisporites sp. B 1975 Gao Lianda et Hou Jingpeng, pl.2, 7

Punctatisporites sp. 3 1983b Gao Lianda, pl.106, 16
Retialetes sp. 1975 Gao Lianda et Hou Jingpeng, pl.8, 3
Retusotriletes biornatus Schutlz 1975 Gao Lianda et Hou Jingpeng, pl.2, 15
Retusotriletes diligens Tschibr. 1975 Gao Lianda et Hou Jingpeng, pl.2, 13
Retusotriletes laevigatus Naum. 1975 Gao Lianda et Hou Jingpeng, pl.2, 9
Retusotriletes parrimammatus Naum. 1975 Gao Lianda et Hou Jingpeng, pl.2, 8
Retusotriletes pychovii Naum. 1975 Gao Lianda et Hou Jingpeng, pl.2, 10
Retusotriletes simplex Naum. 1975 Gao Lianda et Hou Jingpeng, pl.3, 10
Spinozonotriletes maxinus Lanninger 1975 Gao Lianda et Hou Jingpeng, pl.12, 4-5
Spinozonotriletes sp. 1975 Gao Lianda et Hou Jingpeng, pl.12, 6
Stenozonotriletes extensus var. *medius* Naum. 1975 Gao Lianda et Hou Jingpeng, pl.8, 16a-b
Stenozonotriletes laratus Gao et Hou, 1975 1975 Gao Lianda et Hou Jingpeng, pl.8, 20a-b
Stenozonotriletes sp. A 1975 Gao Lianda et Hou Jingpeng, pl.8, 19a-b
Stenozonotriletes sp. B 1975 Gao Lianda et Hou Jingpeng, pl.9, 1
Stenozonotriletes zonalis Naum. 1975 Gao Lianda et Hou Jingpeng, pl.8, 17a-b
Verrucosisporites mediscris Gao et Hou, 1975 1975 Gao Lianda et Hou Jingpeng, pl.5, 11
Verrucosisporites papillosus Gao et Hou, 1975 1975 Gao Lianda et Hou Jingpeng, pl.5, 15-16
Verrucosisporites polygonalis Lanninger 1975 Gao Lianda et Hou Jingpeng, pl.6, 3a-b
Verrucosisporites semanticus (Tschibr.) Gao et Hou, 1975 1975 Gao Lianda et Hou Jingpeng, pl.6, 4
Verrucosisporites sp. A 1975 Gao Lianda et Hou Jingpeng, pl.5, 10
Verrucosisporites sp. B 1975 Gao Lianda et Hou Jingpeng, pl.5, 12
Verrucosisporites sp. C 1975 Gao Lianda et Hou Jingpeng, pl.6, 2
Verrucososporites intortus Gao et Hou, 1975 1975 Gao Lianda et Hou Jingpeng, pl.13, 13a-c
Verrucososporites minus Gao et Hou, 1975 1975 Gao Lianda et Hou Jingpeng, pl.13, 14a-b

Hou'ershan and Lishan Section, Dushan County: Upper Danlin Formation

Acanthotriletes perpusillus Naum. 1975 Gao Lianda et Hou Jingpeng, pl.4, 10-11
Acanthotriletes tenuispinosus Naum. 1975 Gao Lianda et Hou Jingpeng, pl.4, 13
Acinosporites shujiapinensis Gao et Hou, 1975 1975 Gao Lianda et Hou Jingpeng, pl.8, 13-14
Acinosporites sp. A 1975 Gao Lianda et Hou Jingpeng, pl.8, 12
Acinosporites sp. B 1975 Gao Lianda et Hou Jingpeng, pl.8, 15
Ancyrospora breviradius (Tschibr.) Gao et Hou, 1975 1975 Gao Lianda et Hou Jingpeng, pl.11, 1-2
Apiculatisporites eximius (Naum.) Gao et Hou, 1975 1975 Gao Lianda et Hou Jingpeng, pl.5, 3a-b
Apiculiretusispora greggsis (McGregor) Gao et Hou, 1975 1975 Gao Lianda et Hou Jingpeng, pl.3, 6
Apiculiretusispora homogranulatus Gao et Hou, 1975 1975 Gao Lianda et Hou Jingpeng, pl.3, 4-5
Apiculiretusispora microperforatus (Tschibr.) Gao et Hou, 1975 1975 Gao Lianda et Hou Jingpeng, pl.3, 7
Apiculiretusispora subgibberosus Naum. var. *capitellatus* (Tschibr.) Gao et Hou, 1975 1975 Gao Lianda et Hou Jingpeng, pl.3, 11
Archaeozonotriletes semilucensis Naum. 1975 Gao Lianda et Hou Jingpeng, pl.9, 10
Brochotriletes sp. 1975 Gao Lianda et Hou Jingpeng, pl.7, 2
Calamospora cf. *atava* (Naum.) McGregor 1975 Gao Lianda et Hou Jingpeng, pl.1, 13
Calamospora microrugosa (Ibr.) S. W. et B. 1975 Gao Lianda et Hou Jingpeng, pl.1, 14
Camptotriletes sp. 1975 Gao Lianda et Hou Jingpeng, pl.8, 4
Cirratriradites sp. A 1975 Gao Lianda et Hou Jingpeng, pl.9, 4

Cirratriradites sp. B 1975 Gao Lianda et Hou Jingpeng, pl.9, 5a-b
Cirratriradites sp. C 1975 Gao Lianda et Hou Jingpeng, pl.9, 6
Convolutispora sinuous Gao et Hou, 1975 1975 Gao Lianda et Hou Jingpeng, pl.6, 9
Convolutispora sp.A 1975 Gao Lianda et Hou Jingpeng, pl.6, 10
Convolutispora sp.B 1975 Gao Lianda et Hou Jingpeng, pl.6, 11a-b
Cyclogranisporites bonyangensis Gao et Hou, 1975 1975 Gao Lianda et Hou Jingpeng, pl.4, 5
Cyclogranisporites rugosus (Naum.) Gao et Hou, 1975 1975 Gao Lianda et Hou Jingpeng, pl.4, 3
Cyclogranisporites sp. 1975 Gao Lianda et Hou Jingpeng, pl.4, 6
Cyclogranisporites vulgaris (Kedo) Gao et Hou, 1975 1975 Gao Lianda et Hou Jingpeng, pl.4, 2
Densosporites cf. *formosus* Artuz 1975 Gao Lianda et Hou Jingpeng, pl.9, 2a-b
Densosporites sp. 1975 Gao Lianda et Hou Jingpeng, pl.9, 3
Diatomozonotriletes sp. 1975 Gao Lianda et Hou Jingpeng, pl.9, 13
Dibolisporites coniculus Gao et Hou, 1975 1975 Gao Lianda et Hou Jingpeng, pl.4, 19
Dibolisporites giganteus Gao et Hou, 1975 1975 Gao Lianda et Hou Jingpeng, pl.3, 8-9
Dibolisporites sp. 1975 Gao Lianda et Hou Jingpeng, pl.5, 1
Dibolisporites wetteldofensis Lanninger 1975 Gao Lianda et Hou Jingpeng, pl.4, 20-21
Dictyotriletes cf. *nigratus* Naum. 1975 Gao Lianda et Hou Jingpeng, pl.7, 10
Dictyotriletes famenensis Naum. 1975 Gao Lianda et Hou Jingpeng, pl.7, 13a-b
Dictyotriletes mutatus Naum. var. *atavus* (Tschibr) Gao et Hou, 1975 1975 Gao Lianda et Hou Jingpeng, pl.7, 11
Dictyotriletes serrulatus Gao et Hou, 1975 1975 Gao Lianda et Hou Jingpeng, pl.7, 15
Dictyotriletes triangularia Gao et Hou, 1975 1975 Gao Lianda et Hou Jingpeng, pl.7, 14a-b; 1983b Gao Lianda, pl.110, 10
Emphanisporites annulatus McGregor, 1961 1975 Gao Lianda et Hou Jingpeng, pl.8, 5; 1983b Gao Lianda, pl.110, 12
Emphanisporites cf. *obscurus* McGregor 1975 Gao Lianda et Hou Jingpeng, pl.8, 7
Grandispora douglostoworense McGregor 1975 Gao Lianda et Hou Jingpeng, pl.12, 1
Grandispora sp. A 1975 Gao Lianda et Hou Jingpeng, pl.11, 8
Granulatisporites kweichowensis Gao et Hou, 1975 1975 Gao Lianda et Hou Jingpeng, pl.4, 1
Granulatisporites labiatus Gao et Hou, 1975 1975 Gao Lianda et Hou Jingpeng, pl.3, 15
Granulatisporites magnus (Naum.) Gao et Hou, 1975 1975 Gao Lianda et Hou Jingpeng, pl.3, 16
Granulatisporites miliarius Gao et Hou, 1975 1975 Gao Lianda et Hou Jingpeng, pl.3, 13
Granulatisporites minusculus (Naum.) Gao et Hou, 1975 1975 Gao Lianda et Hou Jingpeng, pl.3, 14
Hymenozonotriletes sp. A 1975 Gao Lianda et Hou Jingpeng, pl.10, 2
Hymenozonotriletes varabilis Naum. 1975 Gao Lianda et Hou Jingpeng, pl.10, 3
Hystricosporites sp. 1975 Gao Lianda et Hou Jingpeng, pl.12, 7
Laevigatosporites commutatus (Tschibr.) Gao et Hou, 1975 1975 Gao Lianda et Hou Jingpeng, pl.13, 4a-b
Laevigatosporites microellipsoideus Gao et Hou, 1975 1975 Gao Lianda et Hou Jingpeng, pl.13, 2a-b
Laevigatosporites rarus (Naum.) Gao et Hou, 1975 1975 Gao Lianda et Hou Jingpeng, pl.13, 1
Laevigatosporites sp. B 1975 Gao Lianda et Hou Jingpeng, pl.13, 7
Laevigatosporites usitatus (Tschibr.) Gao et Hou, 1975 1975 Gao Lianda et Hou Jingpeng, pl.13, 3
Leiotriletes dissimilis McGregor 1975 Gao Lianda et Hou Jingpeng, pl.1, 2
Leiotriletes pullatus Naum. 1975 Gao Lianda et Hou Jingpeng, pl.1, 8
Lophozonotriletes cf. *concessus* Naum. 1975 Gao Lianda et Hou Jingpeng, pl.5, 7
Lophozonotriletes sp. 1975 Gao Lianda et Hou Jingpeng, pl.6, 8
Lophozonotriletes sp. B 1975 Gao Lianda et Hou Jingpeng, pl.5, 9
Lophozonotriletes tanlinensis Gao et Hou, 1975 1975 Gao Lianda et Hou Jingpeng, pl.5, 8a-b

Lycospora sp. 1975 Gao Lianda et Hou Jingpeng, pl.9, 7-8
Microreticulatisporites sp. 1975 Gao Lianda et Hou Jingpeng, pl.7, 3
Punctatisporites cricorugosus Gao et Hou, 1975 1975 Gao Lianda et Hou Jingpeng, pl.2, 5
Punctatisporites famenensis (Naum.) Gao et Hou, 1975 1975 Gao Lianda et Hou Jingpeng, pl.2, 4
Punctatisporites giganteus (Naum.) Gao et Hou, 1975 1975 Gao Lianda et Hou Jingpeng, pl.2, 1
Punctatisporites medius (Naum.) Gao et Hou, 1975 1975 Gao Lianda et Hou Jingpeng, pl.2, 3
Punctatisporites pyramidalis Gao et Hou, 1975 1975 Gao Lianda et Hou Jingpeng, pl.2, 2
Punctatisporites rugosus Gao et Hou, 1975 1975 Gao Lianda et Hou Jingpeng, pl.1, 23
Punctatisporites sp. A 1975 Gao Lianda et Hou Jingpeng, pl.2, 6
Punctatisporites subminor (Naum.) Gao et Hou, 1975 1975 Gao Lianda et Hou Jingpeng, pl.1, 17
Punctatisporites typicus (Naum.) Gao et Hou, 1975 1975 Gao Lianda et Hou Jingpeng, pl.1,21
Punctatosporites gangitus Gao et Hou, 1975 1975 Gao Lianda et Hou Jingpeng, pl.13, 9
Punctatosporites labiatus Gao et Hou, 1975 1975 Gao Lianda et Hou Jingpeng, pl.13, 11
Punctatosporites sp. 1975 Gao Lianda et Hou Jingpeng, pl.13, 10
Reticulatisporites cf. *emsiensis* Allen 1975 Gao Lianda et Hou Jingpeng, pl.7, 7
Reticulatisporites houershanensis Gao et Hou, 1975 1975 Gao Lianda et Hou Jingpeng, pl.7, 8
Reticulatisporites serratus Gao et Hou, 1975 1975 Gao Lianda et Hou Jingpeng, pl.7, 6a-d
Retusotriletes communis Naum. var. *modestus* Tschibr. 1975 Gao Lianda et Hou Jingpeng, pl.2, 19
Retusotriletes furcutus Gao et Hou, 1975 1975 Gao Lianda et Hou Jingpeng, pl.2, 20
Retusotriletes inimitabilis Tschibr. 1975 Gao Lianda et Hou Jingpeng, pl.2, 11
Retusotriletes radiatus Gao et Hou, 1975 1975 Gao Lianda et Hou Jingpeng, pl.2, 17
Retusotriletes raise Tschibr. 1975 Gao Lianda et Hou Jingpeng, pl.3, 1
Retusotriletes simplex Naum. 1975 Gao Lianda et Hou Jingpeng, pl.3, 10
Retusotriletes sterlibaschevensis var. *denticulatus* Tschibr. 1975 Gao Lianda et Hou Jingpeng, pl.2, 12a-b
Retusotriletes translaticius Tschibr. 1975 Gao Lianda et Hou Jingpeng, pl.2, 14
Retusotriletes triangulatus (Streel) Streel 1975 Gao Lianda et Hou Jingpeng, pl.2, 18
Stenozonotriletes extensus var. *major* Naum. 1975 Gao Lianda et Hou Jingpeng, pl.8, 18
Stenozonotriletes extensus var. *medius* Naum. 1975 Gao Lianda et Hou Jingpeng, pl.8, 16a-b
Stenozonotriletes flexuosus Gao et Hou, 1975 1975 Gao Lianda et Hou Jingpeng, pl.8, 22
Stenozonotriletes sp. A 1975 Gao Lianda et Hou Jingpeng, pl.8, 19a-b
Stenozonotriletes zonalis Naum. 1975 Gao Lianda et Hou Jingpeng, pl.8, 17a-b
Verrucosisporites cf. *sifati* (Ibr.) S. W. et B. 1975 Gao Lianda et Hou Jingpeng, pl.5, 14
Verrucosisporites evlanensis (Naum.) Gao et Hou, 1975 1975 Gao Lianda et Hou Jingpeng, pl.6, 1
Verrucosisporites krypsis Gao et Hou, 1975 1975 Gao Lianda et Hou Jingpeng, pl.6, 5
Verrucosisporites moniliformis Gao et Hou, 1975 1975 Gao Lianda et Hou Jingpeng, pl.6, 6a-b
Verrucosisporites polygonalis Lanninger 1975 Gao Lianda et Hou Jingpeng, pl.6, 3a-b
Verrucosisporites pseudoreticulatus Gao et Hou, 1975 1975 Gao Lianda et Hou Jingpeng, pl.5, 13
Verrucososporites microtuberculatus (Tschibr.) Gao et Hou, 1975 1975 Gao Lianda et Hou Jingpeng, pl.13, 12a-e
Verrucososporites minus Gao et Hou, 1975 1975 Gao Lianda et Hou Jingpeng, pl.13, 14a-b

Hunan

Yuankou Section and Shangzhai Section, Jiangyong County; Xiaowei Section, Jianghua County: Yuankou Formation

Acinosporites cf. *salaopiensis* Richardson et Lister, 1969 1990 Gao Lianda et Wang Genxian, pl.1, 34
Acinosporites sp. 1990 Gao Lianda et Wang Genxian, p.2
Acinosporites sp. Rodriguez 1990 Gao Lianda et Wang Genxian, p.3
Ambitisporites avitus Hoffmeister 1990 Gao Lianda et Wang Genxian, p.3
Ambitisporites dilutus (Hoffmeister) Richardson et Lister, 1969 1990 Gao Lianda et Wang Genxian, pl.1, 31
Amocosporites sp. 1990 Gao Lianda et Wang Genxian, pl.1, 26
Amocosporites miserabilis (Cramer) Cramer et Diez 1975 1990 Gao Lianda et Wang Genxian, pl.1, 30
Anapiculatisporites isidori Cramer et Diez 1990 Gao Lianda et Wang Genxian, p.2
Anapiculatisporites sp. 1990 Gao Lianda et Wang Genxian, pl.1, 23
Anapiculatisporites sp. B Streel et al., 1981 1990 Gao Lianda et Wang Genxian, pl.1, 24
Anapiculatisporites sp. C Le Herisse, 1982 1990 Gao Lianda et Wang Genxian, p.2
Anapiculatisporites sp. C Streel et al., 1981 1990 Gao Lianda et Wang Genxian, pl.1, 18
Apiculatasporites microconus (Richardson) McGregor et Camfield 1990 Gao Lianda et Wang Genxian, p.2
Apiculatasporites sp. 1990 Gao Lianda et Wang Genxian, pl.1, 15
Apiculiretusispora brandtii Streel 1990 Gao Lianda et Wang Genxian, p.2
Apiculiretusispora plicata (Allen) Streel 1990 Gao Lianda et Wang Genxian, p.2
Apiculiretusispora spicula Richardson et Lister 1990 Gao Lianda et Wang Genxian, p.3
Apiculiretusispora synorea Richardson et Lister 1990 Gao Lianda et Wang Genxian, p.3
Archaeozonotriletes chulus var. *chulus* Richardson et Lister, 1969 1990 Gao Lianda et Wang Genxian, pl.1, 21
Brochotriletes cf. *robustus* (Scott et Rouse) McGregor 1990 Gao Lianda et Wang Genxian, p.2
Brochotriletes foveolatus Naumova 1990 Gao Lianda et Wang Genxian, p.3
Brochotriletes rarus Arkhangelskaya 1978 1990 Gao Lianda et Wang Genxian, pl.1, 38
Brochotriletes sp. 1990 Gao Lianda et Wang Genxian, p.3
Camptozonotriletes cf. *caperatus* McGregor 1990 Gao Lianda et Wang Genxian, p.3
Chelinospora cassicula Richardson et Lister, 1969 1990 Gao Lianda et Wang Genxian, pl.1, 12
Chelinospora cf. *cassicula* Richardson et Lister 1990 Gao Lianda et Wang Genxian, p.2
Chelinospora sp. 1990 Gao Lianda et Wang Genxian, pl.1, 20
Coronaspora mariae Rodriguez, 1983 1990 Gao Lianda et Wang Genxian, pl.1, 29
Crissispora guangxiensis Gao, 1978 1990 Gao Lianda et Wang Genxian, pl.1, 27
Cymbosporites dittonensis Richardson et Lister, 1969 1990 Gao Lianda et Wang Genxian, pl.1, 13-14
Cymbosporites proteus McGregor et Camfield, 1976 1990 Gao Lianda et Wang Genxian, pl.1, 19
Cymbosporites sp. 1990 Gao Lianda et Wang Genxian, pl.1, 28
Dibolisporites cf. *echinaccus* Richardson 1990 Gao Lianda et Wang Genxian, p.3
Dibolisporites sp. 1990 Gao Lianda et Wang Genxian, p.2
Dictyotriletes fovosus McGregor et Camfield 1990 Gao Lianda et Wang Genxian, p.3
Dictyotriletes geriense Rodriguez, 1979 1990 Gao Lianda et Wang Genxian, pl.1, 17
Dictyotriletes gorgoensis Cramer 1990 Gao Lianda et Wang Genxian, p.3
Emphanisporites decoratus Allen, 1965 1990 Gao Lianda et Wang Genxian, pl.1, 37
Emphanisporites micrornatus Richardson et Lister, 1969 1990 Gao Lianda et Wang Genxian, pl.1, 36
Endosporites sp. 1990 Gao Lianda et Wang Genxian, pl.1, 22
Hymenozonotriletes antiguus Gao, 1978 1990 Gao Lianda et Wang Genxian, pl.1, 35
Iberospora guzmani Cramer et Diez 1975 1990 Gao Lianda et Wang Genxian, pl.1, 25
Perotriletes sp. 1990 Gao Lianda et Wang Genxian, p.3
Pustulatisporites sp. A 1990 Gao Lianda et Wang Genxian, pl.1, 16

Retusotriletes aureoladus Rodriguez 1990 Gao Lianda et Wang Genxian, p.2
Retusotriletes dittonensis Richardson et Lister 1990 Gao Lianda et Wang Genxian, p.2
Retusotriletes dubius (Eisenack) Richardson 1990 Gao Lianda et Wang Genxian, p.2
Retusotriletes minor Kedo 1990 Gao Lianda et Wang Genxian, p.2
Retusotriletes simplex Naumova 1990 Gao Lianda et Wang Genxian, p.2
Retusotriletes warringtonii Richardson et Lister 1990 Gao Lianda et Wang Genxian, p.2
Samarisporites sp. A Richardson et al., 1984 1990 Gao Lianda et Wang Genxian, p.3
Stenozonotriletes pumilus (Waltz) Naumova, 1953 1990 Gao Lianda et Wang Genxian, pl.1, 32-33
Streelispora cf. *granulata* Richardson et Lister, 1969 1990 Gao Lianda et Wang Genxian, pl.1, 9
Streelispora cf. *newportensis* (Chaloner et Streel) Richardson et Lister, 1969 1990 Gao Lianda et Wang Genxian, pl.1, 6-7
Streelispora newportensis (Chaloner et Streel) Richardson et Lister, 1969 1990 Gao Lianda et Wang Genxian, pl.1, 3-5
Streelispora richardsonii Rodriguez 1990 Gao Lianda et Wang Genxian, p.2
Streelispora sp. 1990 Gao Lianda et Wang Genxian, pl.1, 8
Streelospora granulata Richardson et Lister, 1969 1990 Gao Lianda et Wang Genxian, pl.1, 1
Synorispora verrucata Richardson et Lister, 1969 1990 Gao Lianda et Wang Genxian, pl.1, 10-11
Synorisporites cf. *tripapillatus* Richardson et Lister 1990 Gao Lianda et Wang Genxian, p.3
Synorisporites cf. *verrucata* Richardson et Lister, 1969 1990 Gao Lianda et Wang Genxian, pl.1, 2
Synorisporites verrucaus 1990 Gao Lianda et Wang Genxian, p.3
Verruciretusispora dubia (Eisenack) Richardson et Rasul 1990 Gao Lianda et Wang Genxian, p.3
Verrucosisporites sp. 1990 Gao Lianda et Wang Genxian, p.2

Sichuan

Ganxi Section, Beichuan County, Mianyang City: Guixi Formation; Mu’erchang Formation; Guanyinmiao Formation
Anapiculatisporites minutus Gao, 1988 1988 Gao, pl. 143, 17, 18
Archaeozonotriletes jiangyouensis Gao, 1988 1988 Gao, pl. 149, 24
Cymbosporites granulates Gao, 1988 1988 Gao, pls. 148, 24, 25; 152, 4, 5, 6, 7
Cymbosporites macrotuberculatus Gao, 1988 1988 Gao, pl. 148, 26
Cymbosporites minutus Gao, 1988 1988 Gao, pl. 141, 5
Cymbosporites spissatus Gao, 1988 1988 Gao, pl. 149, 1, 2
Dictyotriletes camutus Gao, 1988 1988 Gao, pl. 145, 15, 16, 18
Synorisporites similverrucatus Gao, 1988 1988 Gao, pl. 147, 13, 14
Verrucosisporites evlanensis (Naumova) Gao, 1988 1988 Gao, pl. 144, 2, 6

Eifelian

Yunnan

Xichong Section, Zhanyi County; Luquan: Chuandong Formation
Acinosporites acanthomammillatus Richardson 1991 Xu Ren et Gao Lianda, pl.I, 8; pl.II, 9
Acinosporites macrospinosus 1991 Xu Ren et Gao Lianda, pl.306
Ancyrospora ancyea var. *ancyrea* (Eisenack) Richardson 1994 Xu Ren et Gao Lianda, p.119

Ancyrospora angulata (Tiwari et Schaaschmidt) McGregor et Camfield 1994 Xu Ren et Gao Lianda, p.119
Ancyrospora gandispinosa Richardson 1994 Xu Ren et Gao Lianda, p.120
Apiculatasporites perpusillus (Naumova et Chibrikova) McGregor 1991 Xu Ren et Gao Lianda, pl.I, 17
Apiculiretusispora plicata 1991 Xu Ren et Gao Lianda, pl.307
Brochotriletes foveolatus Naumova 1991 Xu Ren et Gao Lianda, pl.I, 16
Calyptosporites velatus (Eisenack) Richardson 1991 Xu Ren et Gao Lianda, pl.II, 10
Camarozonotriletes pusillus Naumova ex Chibrikova 1991 Xu Ren et Gao Lianda, pl.I, 19
Corystisporites multispinosus var. *multispinosus* McGregor et Camfield 1991 Xu Ren et Gao Lianda, pl.I, 12
Dibolisporites bullatus (Allen) Richardson 1991 Xu Ren et Gao Lianda, pl.I, 9
Dibolisporites echinaceus (Eisenack) Richardson 1991 Xu Ren et Gao Lianda, pl.I, 2
Dictyotriletes gorgoneus 1991 Xu Ren et Gao Lianda, p.307
Dictyotriletes subgranifer McGregor 1991 Xu Ren et Gao Lianda, pl.I, 13-14
Emphanisporites annulatus 1991 Xu Ren et Gao Lianda, p.306
Geminospora micropaxilla 1991 Xu Ren et Gao Lianda, p.306
Grandispora douglastownense McGregor 1991 Xu Ren et Gao Lianda, pl.II, 8
Hystricosporites corystus Richardson 1994 Xu Ren et Gao Lianda, p.120
Hystricosporites grandis Owens 1994 Xu Ren et Gao Lianda, pl.2, 2
Hystricosporites reflexus Owens 1994 Xu Ren et Gao Lianda, p.120
Retusotriletes distinotus Richardson 1991 Xu Ren et Gao Lianda, p.306; 1994 Xu Ren et Gao Lianda, p.119
Retusotriletes rugulatus 1991 Xu Ren et Gao Lianda, p.306
Rhabdosporites langii (Eisenack) Richardson 1991 Xu Ren et Gao Lianda, pl.II, 14
Triletes langii Richardson, 1965 1994 Xu Ren et Gao Lianda, pl.1, 12
Verruciretusispora dubia (Eisenack) Richardson et Rasul 1991 Xu Ren et Gao Lianda, pl.I, 10
Verruciretusispora primus Chi et Hills 1994 Xu Ren et Gao Lianda, p.120
Verrucosisporites paremecus 1991 Xu Ren et Gao Lianda, p.307

Guizhou

Hou'ershan Section, Dushan County; Baoyang Section, Duyun City: Longdongshui Formation

Acanthotriletes minus Gao et Hou, 1975 1975 Gao Lianda et Hou Jingpeng, pl.4, 9; 1983b Gao Lianda, pl.108, 3
Ancyrospora ancyrea var. *ancyrea* Richardson 1975 Gao Lianda et Hou Jingpeng, pl.11, 6
Apiculiretusispora cf. *brandtii* Streel, 1964 1975 Gao Lianda et Hou Jingpeng, pl.3, 3; 1983b Gao Lianda, pl.108, 1
Archaeozonotriletes basilaris Naum. 1975 Gao Lianda et Hou Jingpeng, pl.9, 9
Archaeozonotriletes micromanifestus var. *minor* Naum. 1975 Gao Lianda et Hou Jingpeng, pl.9, 19
Archaeozonotriletes sp. A 1975 Gao Lianda et Hou Jingpeng, pl.9, 18
Brochotriletes foveolatus Naum. 1975 Gao Lianda et Hou Jingpeng, pl.7, 1
Diatomozonotriletes devonicus Naum. var. *azonatus* Tschibr. 1975 Gao Lianda et Hou Jingpeng, pl.9, 14a-c
Granulatisporites sp. 1975 Gao Lianda et Hou Jingpeng, pl.3, 12
Hymenozonotriletes mancus Naum. 1975 Gao Lianda et Hou Jingpeng, pl.10, 8
Hymenozonotriletes sp. B 1975 Gao Lianda et Hou Jingpeng, pl.10, 6
Hymenozonotriletes varius var. *minor* Naum. 1975 Gao Lianda et Hou Jingpeng, pl.10, 7
Hystricosporites corystus Richardson 1975 Gao Lianda et Hou Jingpeng, pl.12, 8
Leiotriletes laevis Naumova, 1953 1983b Gao Lianda, pl.106, 2; 1975 Gao Lianda et Hou Jingpeng, pl.1, 1
Leiotriletes trivialis McGregor 1975 Gao Lianda et Hou Jingpeng, pl.1, 4-5

Punctatisporites pusillus (Naum.) Gao et Hou, 1975 1975 Gao Lianda et Hou Jingpeng, pl.1, 20
Retusotriletes cf. *distinctus* Richardson 1975 Gao Lianda et Hou Jingpeng, pl.2, 16
Verrucososporites intortus Gao et Hou, 1975 1975 Gao Lianda et Hou Jingpeng, pl.13, 13a-c

Givetian

Hunan

Shanmen Reservoir, Li County; Tianmenya Village, Shimen County: Yuntaiguan Formation
Acanthotriletes serratus Naumova 1989 Gao Lianda, p.3
Acinosporites acanthomammillatus Richardson, 1965 1989 Gao Lianda, pl.1, 4
Acinosporites granulis (Chibrikova) Gao, 1989 1989 Gao Lianda, pl.1, 6
Ancyrospora ampulla Owens, 1971 1989 Gao Lianda, pl.2, 10
Ancyrospora ancyrea var. *ancyrea* Richardson 1989 Gao Lianda, p.3
Ancyrospora ancyrea var. *brevispinosa* Richardson, 1965 1989 Gao Lianda, pl.2, 7
Ancyrospora furcala Owens, 1971 1989 Gao Lianda, pl.1, 11
Ancyrospora involucra Owens, 1971 1989 Gao Lianda, pl.2, 5
Ancyrospora melvillensis Owens, 1971 1989 Gao Lianda, pl.2, 1-2
Aneurospora greggsii (McGregor) Streel 1989 Gao Lianda, p.2
Apiculiretusispora microspinosa Gao 1989 1989 Gao Lianda, pl.1, 3
Apiculiretusispora nitida Owens 1989 Gao Lianda, p.2
Apiculiretusispora plicata (Allen) Streel 1989 Gao Lianda, p.2
Archaeoperisaccus sp. 1989 Gao Lianda, p.3
Archaeozonotriletes polymorphus Naumova, 1953 1989 Gao Lianda, pl.1, 12
Archaeozonotriletes variabilis (Naumova) Allen 1989 Gao Lianda, p.2
Asperospora serena (Kedo) Gao 1989 Gao Lianda, p.3
Bullatisporites bullatus Allen 1989 Gao Lianda, p.2
Chelinospora concinna Allen, 1965 1989 Gao Lianda, pl.1, 7
Convolutispora spp. 1989 Gao Lianda, p.3
Corystisporites multispinosus Richardson, 1965 1989 Gao Lianda, pl.2, 9
Cristatisporites multispinosa Gao 1989 1989 Gao Lianda, pl.2, 15
Cristatisporites triangulatus (Allen) McGregor et Camfield 1989 Gao Lianda, p.3
Cymbosporites cyathus Allen, 1965 1989 Gao Lianda, pl.2, 13
Cymbosporites decorus Allen 1989 Gao Lianda, p.3
Cymbosporites magnificus (McGregor) McGregor et Camfield 1976 1989 Gao Lianda, pl.1, 8
Cymbosporites nanus (Naumova) Gao 1989 Gao Lianda, p.3
Dibolisporites echinaceus (Eisenack) Richardson, 1965 1989 Gao Lianda, pl.1, 5
Dictyotriletes nigratus Naumova 1989 Gao Lianda, p.2
Geminospora tuberculata var. *tuberculata* McGregor et Camfield, 1982 1989 Gao Lianda, pl.2, 12
Geminospora verrusta (Naumova) McGregor et Camfield 1989 Gao Lianda, p.3
Grandispora inculta Allen, 1965 1989 Gao Lianda, pl.2, 16
Grandispora megaformis (Richardson) McGregor, 1973 1989 Gao Lianda, pl.1, 16
Grandispora polymorphus (Naumova) Gao 1989 Gao Lianda, p.3
Hysticosporites delicabilis McGregor, 1961 1989 Gao Lianda, pl.1, 10

Hysticosporites spp. 1989 Gao Lianda, p.3
Leiofusa spp. 1989 Gao Lianda, p.3
Michystridium spp. 1989 Gao Lianda, p.3
Navifusa bacillata Playford 1989 Gao Lianda, p.3
Nikitinsporites sp. 1989 Gao Lianda, pl.2, 6
Retusotriletes communis Naumova 1989 Gao Lianda, pl.1, 1
Retusotriletes rotundus (Streel) Streel 1989 Gao Lianda, p.2
Retusotriletes triangulatus (Streel) Streel 1989 Gao Lianda, pl.1, 2
Rhabdosporites langii (Eisenaesk) Richardson, 1965 1989 Gao Lianda, pl.2, 11
Samarisporites concinnus Owens, 1971 1989 Gao Lianda, pl.1, 14
Samarisporites inaequus (McGregor) Owens 1989 Gao Lianda, p.3
Spinozonotriletes cassideus Owens 1989 Gao Lianda, p.3
Verrucosiretusispora robusta Owens, 1971 1989 Gao Lianda, pl.1, 9
Verrucosisporites premnus Richardson 1989 Gao Lianda, p.2
Verrucosisporites uncatus (Naumova) Richardson 1953 1989 Gao Lianda, pl.1, 13

Hubei

Liujiachanghuangkuang Section, Songzi County: Yuntaiguan Formation
Acanthotriletes serratus Naumova 1989 Gao Lianda, p.3
Acinosporites acanthomammillatus Richardson 1989 Gao Lianda, p.3
Ancyrospora sp. 1989 Gao Lianda, pl.2, 8
Aneurospora goensis Streel et Lele 1989 Gao Lianda, p.3
Aneurospora greggsii (McGregor) Streel 1989 Gao Lianda, p.3
Apiculiretusispora nitida Owens 1984 Feng Shaonan, pl.50, 6
Archaeozonotriletes variabilis (Naumova) Allen 1989 Gao Lianda, p.3
Calamospora atava (Naumova) McGregor 1989 Gao Lianda, p.3
Cymbosporites magnificus (McGregor) McGregor et Camfield 1989 Gao Lianda, p.3
Dictyotriletes minor Naumova 1989 Gao Lianda, p.3
Geminospora basilaris (Naumova) Gao 1989 Gao Lianda, p.3
Geminospora formosus (Naumova) Gao 1989 Gao Lianda, p.3
Geminospora parvibasilaria (Naumova) Gao et Zhong, 1984 1984 Feng Shaonan, pl.50, 29-30
Grandispora douglastownense McGregor 1989 Gao Lianda, p.3
Hymenozonotriletes denticulatus Naumova 1989 Gao Lianda, p.3
Retusotriletes simplex Naumova 1989 Gao Lianda, p.3

Yunnan

1. **Longhuashan Section and Xichong Section, Zhanyi County; Lunan County; Luquan County: Haikou Formation**

Acinosporites acanthomammillatus Richardson 1991 Xu Ren et Gao Lianda, pl.I, 8; pl.II, 9
Anapiculatisporites dilutus Lu, 1980 1980 Lu Lichang, pl.3, 23-27
Anapiculatisporites minutus Lu et Ouyang 1980 Lu Lichang, pl.4, 1-2; pl.11, 11
Ancyrospora acuminata Lu, 1980 1980 Lu Lichang, pl.7, 14-16

Ancyrospora aff. *incisa* (Naumova) Lu, 1980 1980 Lu Lichang, pl.7, 5-8
Ancyrospora ampulla 1991 Xu Ren et Gao Lianda, p.308
Ancyrospora ancyea var. *ancyrea* (Eisenack) Richardson, 1965 1994 Xu Ren et Gao Lianda, pl.2, 4
Ancyrospora ancyrea ancyrea Richardson 1962 1983b Gao Lianda, pl.112, 3-5
Ancyrospora angulata (Tiwari et Schaarschmidt) McGregor et Camfield, 1982 1994 Xu Ren et Gao Lianda, pl.3, 10
Ancyrospora arguta (Naumova) Lu, 1980 1980 Lu Lichang, pl.7, 12-13
Ancyrospora delicatus 1991 Xu Ren et Gao Lianda, p.308
Ancyrospora dentata (Naumova) Lu, 1980 1980 Lu Lichang, pl.7, 9-11
Ancyrospora involucra Owens, 1971 1994 Xu Ren et Gao Lianda, pl.2, 1
Ancyrospora longii (Taugourdeau et Lantz) Allen, 1965 1994 Xu Ren et Gao Lianda, pl.2, 5
Ancyrospora longispinosa Richardson 1962 1994 Xu Ren et Gao Lianda, pl.3, 12
Ancyrospora melvillensis Owens 1994 Xu Ren et Gao Lianda, p.120
Ancyrospora sp. 1994 Xu Ren et Gao Lianda, pl.2, 3
Ancyrospora subcircularis Lu, 1980 1980 Lu Lichang, pl.8, 1-4
Aneurospora greggsii 1991 Xu Ren et Gao Lianda, p.308
Apiculatasporites brevidenticulatus (Chibrikova) McGregor et Camfield 1991 Xu Ren et Gao Lianda, pl.I,18
Apiculatisporis aculeatus (Ibrahim) Potoni éet Kremp 1980 Lu Lichang, pl.3, 19-22
Apiculatisporis sp. 1980 Lu Lichang, pl.4, 3-4
Apiculiretusispora crassa Lu, 1980 1980 Lu Lichang, pl.11, 17-18
Apiculiretusispora microverrucosa (Bharadwaj, Tiwari et Venkatachala) Lu, 1980 1980 Lu Lichang, pl.3, 9-10, 14-18
Apiculiretusispora minuta Lu et Ouyang 1980 Lu Lichang, pl.2, 14
Apiculiretusispora pseudozonalis Lu, 1980 1980 Lu Lichang, pl.3, 11-13
Apiculiretusispora sp. 1980 Lu Lichang, pl.2, 15-16
Archaeoperisaccus cf. *scabratus* Owens 1980 Lu Lichang, pl.9, 8-9
Archaeoperisaccus elongatus Naumova 1991 Xu Ren et Gao Lianda, pl.II, 13
Archaeoperisaccus granulatus Gao 1991 Xu Ren et Gao Lianda, pl.II, 12
Archaeoperisaccus oviformis Lu, 1980 1980 Lu Lichang, pl.1, 8-10
Archaeoperisaccus xichongensis Lu, 1980 1980 Lu Lichang, pl.1, 6-7
Archaeozonotriletes auritus Lu, 1980 1980 Lu Lichang, pl.5, 7-8
Archaeozonotriletes lageniformis Lu, 1980 1980 Lu Lichang, pl.5, 5-6
Archaeozonotriletes variabilis (Naumova) Allen 1991 Xu Ren et Gao Lianda, pl.II, 1; 1980 Lu Lichang, pl.5, 1-4
Archaeozonotriletes variabilis var. *gigantea* 1991 Xu Ren et Gao Lianda, p.308
Auroraspora macromanifestus (Hacquebard) Richardson 1991 Xu Ren et Gao Lianda, pl.II, 15
Biharisporites microspinosus 1994 1994 Xu Ren et Gao Lianda, pl.1, 11; pl.3, 13-14
Biharisporites parviornatus Richardson 1994 Xu Ren et Gao Lianda, p.120
Brochotriletes foveolatus Naumova 1991 Xu Ren et Gao Lianda, pl.I, 16
Calamospora pannucea Richardson 1980 Lu Lichang, pl.1, 9
Calyptosporites proteus 1991 Xu Ren et Gao Lianda, p.306
Calyptosporites velatus (Eisenack) Richardson 1991 Xu Ren et Gao Lianda, pl.II, 10
Camarozonotriletes devonicus Naumova 1991 Xu Ren et Gao Lianda, pl.I, 11
Camarozonotriletes mosolovicus Naumova ex Kedo 1991 Xu Ren et Gao Lianda, pl.I, 20
Camarozonotriletes pusillus Naumova ex Chibrikova 1991 Xu Ren et Gao Lianda, pl.I, 19
Camarozonotriletes sp. 1980 Lu Lichang, pl.8, 11
Cereusisporites mirabilis Lu et Ouyang, 1978 1978 Lu Lichang et Ouyang Shu, pl.3, 1-4

Chelinospora cf. *multireticulata* Lu, 1980 Lu Lichang, pl.6, 16-17
Chelinospora cf. *ochyrosa* Lu, 1980 Lu Lichang, pl.6, 11
Chelinospora concinna 1991 Xu Ren et Gao Lianda, p.306
Chelinospora irregulata Lu, 1980 1980 Lu Lichang, pl.6, 7-8
Chelinospora ligurata Allen 1980 Lu Lichang, pl.6, 1-4
Chelinospora multireticulata Lu, 1980 1980 Lu Lichang, pl.6, 9-10
Chelinospora ochyrosa Lu, 1980 1980 Lu Lichang, pl.6, 12-13
Chelinospora rarireticulata Lu, 1980 1980 Lu Lichang, pl.6, 5-6
Chelinospora sp. 1980 Lu Lichang, pl.6, 14-15
Cirratriradites avicus 1991 Xu Ren et Gao Lianda, p.306
Contagisporites optivus var. *optivus* 1991 Xu Ren et Gao Lianda, p.307
Contagisporites poticus (Chibrikova) var. *opticus* Owens 1994 Xu Ren et Gao Lianda, p.120
Cornispora? *varicornata* Staplin et Jansonius 1991 Xu Ren et Gao Lianda, pl.II, 3
Coronispora sinica Lu et Ouyang, 1978 1978 Lu Lichang et Ouyang Shu, pl.3, 6-10
Corystisporites multispinosus var. *multispinosus* McGregor et Camfield 1991 Xu Ren et Gao Lianda, pl.I, 12
Cristatisporites menacanthus 1991 Xu Ren et Gao Lianda, p.308
Cristatisporites triangulates (Allen) McGregor et Camfield 1991 Xu Ren et Gao Lianda, pl.II, 5
Cyclogranisporites delicatus Lu, 1980 1980 Lu Lichang, pl.1, 7-8
Cymbosporites catillus 1991 Xu Ren et Gao Lianda, p.308
Cymbosporites cyathus Allen 1980 Lu Lichang, pl.5, 19-21; 1991 Xu Ren et Gao Lianda, pl.I, 27
Cymbosporites dentatus Lu, 1980 1980 Lu Lichang, pl.4, 19; pl.5, 22-24
Cymbosporites magnificus (McGregor) McGregor et Camfield 1991 Xu Ren et Gao Lianda, pl.I, 28
Densosporites sp. 1980 Lu Lichang, pl.7, 4
Dibolisporites echinaceus (Eisenack) Richardson 1991 Xu Ren et Gao Lianda, pl.I, 2
Dictyotriletes destudineus Lu, 1980 1980 Lu Lichang, pl.4, 5-6
Dictyotriletes subgranifer McGregor 1991 Xu Ren et Gao Lianda, pl.I, 13-14
Geminospora compacta 1991 Xu Ren et Gao Lianda, p.308
Geminospora lemurata (Balme) Playford 1991 Xu Ren et Gao Lianda, pl.I, 26
Geminospora micropaxilla 1991 Xu Ren et Gao Lianda, p.306
Geminospora punctata Owens 1980 Lu Lichang, pl.5, 12-16
Geminospora tuberculata (Kedo) Allen 1991 Xu Ren et Gao Lianda, pl.I, 24
Geminospora tuberculata var. *micrornata* 1991 Xu Ren et Gao Lianda, p.306
Geminospora venusta (Naumova) McGregor et Camfield 1991 Xu Ren et Gao Lianda, pl.I, 22-23
Geminospora verrucosa Owens 1980 Lu Lichang, pl.5, 17-18
Grandispora inculta 1991 Xu Ren et Gao Lianda, p.306
Grandispora spinosa 1991 Xu Ren et Gao Lianda, p.308
Granulatisporites triangularis Gao, 1983 1983b Gao Lianda, pl.107, 7
Hymenozonotriletes verrucosus Gao, 1983 1983b Gao Lianda, pl.113, 5
Hymenozonotriletes rarus 1991 Xu Ren et Gao Lianda, p.306
Hystrichosporites cf. *winslovii* 1991 Xu Ren et Gao Lianda, p.308
Hystrichosporites grandis 1991 Xu Ren et Gao Lianda, p.308
Hystrichosporites sp. 1991 Xu Ren et Gao Lianda, p.308
Hystricosporites cf. *microancyreus* Riegel 1980 Lu Lichang, pl.4, 14-15
Hystricosporites corystus Richardson 1994 Xu Ren et Gao Lianda, p.120

Hystricosporites delrctabilis McGregor 1994 Xu Ren et Gao Lianda, p.120

Hystricosporites expandus Chi et Hills 1994 Xu Ren et Gao Lianda, p.120

Hystricosporites furcatus Owens, 1971 1994 Xu Ren et Gao Lianda, pl.2, 12

Hystricosporites gravis Owens, 1971 1994 Xu Ren et Gao Lianda, pl.1, 15

Hystricosporites multifurcaus (Winslow) Mortimer et Chaloner 1994 Xu Ren et Gao Lianda, p.120

Hystricosporites sp. 1 1980 Lu Lichang, pl.4, 17

Hystricosporites sp. 2 1980 Lu Lichang, pl.4, 18

Hystricosporites sp. 3 1980 Lu Lichang, pl.4, 16

Lagenicula bulbosa Chi et Hills, 1976 1994 Xu Ren et Gao Lianda, pl.2, 8-9

Lagenicula bullosum 1991 Xu Ren et Gao Lianda, p.308

Lagenicula devonica var. *devonica* Chi et Hills 1994 Xu Ren et Gao Lianda, p.120; 1991 Xu Ren et Gao Lianda, p.307

Lagenicula expandus Chi et Hills, 1976 1994 Xu Ren et Gao Lianda, pl.2, 6

Lagenicula sp. 1994 Xu Ren et Gao Lianda, pl.2, 7

Lagenicula verrucosa Xu et Gao, 1994 1994 Xu Ren et Gao Lianda, pl.2, 10

Lagenoisporites sp. 1978 Lu Lichang et Ouyang Shu, pl.1, 8

Latosporites sp. 1980 Lu Lichang, pl.9, 10

Leiotriletes cf. *adnatoides* Potonié et Kremp 1980 Lu Lichang, pl.1, 1

Longhuashanispora commixta Lu et Ouyang, 1978 1978 Lu Lichang et Ouyang Shu, pl.2, 1-5; 2012 Xu Honghe et al., fig. 2Q-T, in press

Lophotriletes devonicus 1991 Xu Ren et Gao Lianda, p.306

Lophozonotriletes cf. *tylophorus* Naumova, 1953 1983b Gao Lianda, pl.108, 7-8

Lophozonotriletes grumosus Naumova, 1953 1983b Gao Lianda, pl.108, 10

Lophozonotriletes mucronatus Gao, 1983 1983b Gao Lianda, pl.108, 11

Lophozonotriletes grandis Naumova 1991 Xu Ren et Gao Lianda, pl.I, 4

Lophozonotriletes tylophorus 1991 Xu Ren et Gao Lianda, p.306

Nikitinsporites cathayensis 1978 1978 Lu Lichang et Ouyang Shu, pl.1, 1, 7

Nikitinsporites maximus 1994 1994 Xu Ren et Gao Lianda, pl.3, 9

Nikitinsporites pseudozonatus 1978 1978 Lu Lichang et Ouyang Shu, pl.1, 2-3

Nikitinsporites simplex Chi et Hills, 1976 1994 Xu Ren et Gao Lianda, pl.1, 14

Nikitinsporites sinensis Xu et Gao, 1994 1994 Xu Ren et Gao Lianda, pl.2, 11

Nikitinsporites sp. 1994 Xu Ren et Gao Lianda, pl.3, 6-8

Nikitinsporites spp. 1991 Xu Ren et Gao Lianda, p.308

Nikitinsporites striatus 1978 1978 Lu Lichang et Ouyang Shu, pl.1, 4-6

Nikitinsporites yunnanensis 1994 1994 Xu Ren et Gao Lianda, pl.1, 13

Nikitinsporites? sp. 1994 Xu Ren et Gao Lianda, pl.3, 1

Peroretisporites distalis Lu, 1980 1980 Lu Lichang, pl.9, 5-7; fig.1

Perotrilites conicus Lu, 1980 1980 Lu Lichang, pl.9, 1-3

Perotrilites sp. 1980 Lu Lichang, pl.9, 4

Punctatisporites laevigatus (Naumova) Lu, 1980 1980 Lu Lichang, pl.1, 2-3

Punctatisporites perforatus Lu, 1980 1980 Lu Lichang, pl.1, 4-6

Retizonospora punicoida Lu, 1980 1980 Lu Lichang, pl.6, 18-19

Retusotriletes confossus (Richardson) Lu et Ouyang 1980 Lu Lichang, pl.1, 16-18

Retusotriletes crassus Lu, 1980 1980 Lu Lichang, pl.2, 9-11

Retusotriletes distinctus 1991 Xu Ren et Gao Lianda, p.306

Retusotriletes glossatus Lu, 1980 1980 Lu Lichang, pl.2, 1-2

Retusotriletes impressus Lu, 1980 1980 Lu Lichang, pl.2, 12

Retusotriletes levidensus Lu, 1980 1980 Lu Lichang, pl.1, 22-23

Retusotriletes minor Lu, 1980 1980 Lu Lichang, pl.2, 13

Retusotriletes pychovii Naumova, 1953 1980 Lu Lichang, pl.1, 14-15; pl.10, 5-6; 1991 Xu Ren et Gao Lianda, p.308

Retusotriletes rugulatus 1991 Xu Ren et Gao Lianda, p.306

Retusotriletes scabratus Lu, 1980 1980 Lu Lichang, pl.2, 3-4

Retusotriletes semizonalis McGregor 1980 Lu Lichang, pl.1, 20-21

Retusotriletes simplex Naumova 1980 Lu Lichang, pl.1, 12-13; pl.10, 3-4

Retusotriletes stratus Lu, 1980 1980 Lu Lichang, pl.2, 5-8

Retusotriletes triangulatus (Streel) Streel cf. var. *major* Lu et Ouyang 1980 Lu Lichang, pl.1, 10, 11; pl.10, 8-9

Retusotriletes triangulatus (Streel) Streel var. *microtriangulatus* Lu et Ouyang 1980 Lu Lichang, pl.1, 19; pl.10, 7

Rhabdosporites cf. *cymatilus* Allen 1980 Lu Lichang, pl.4, 8-9

Rhabdosporites langii (Eisenack) Richardson 1991 Xu Ren et Gao Lianda, pl.II, 14

Rhabdosporites zonatus Lu, 1980 1980 Lu Lichang, pl.4, 10

Samarisporites concinnus Owens 1980 Lu Lichang, pl.7, 17

Samarisporites heteroverrucosus Lu, 1980 1980 Lu Lichang, pl.8, 6, 8-9

Samarisporites inaequus 1991 Xu Ren et Gao Lianda, p.307

Samarisporites sp.1 1980 Lu Lichang, pl.8, 7

Samarisporites triangulatus Allen 1980 Lu Lichang, pl.8, 5, 10

Sporogonites yunnanense Hsü 1966 1974 Gu et Zhi, pl.1, 1-3; fig.14; 1966 Hsü, pl.III, 5-6; pl.IV, 1; pl.VI, 1-4; fig.13-14

Stenozonotriletes clarus Ischenko 1980 Lu Lichang, pl.7, 1

Stenozonotriletes extensus Naumova 1991 Xu Ren et Gao Lianda, pl.I, 21

Stenozonotriletes extensus var. *media* 1991 Xu Ren et Gao Lianda, p.308

Stenozonotriletes inspissatus Owens 1980 Lu Lichang, pl.7, 2-3

Synorisporites verrucatus Richardson et Lister 1980 Lu Lichang, pl.4, 11-13

Tholisporites densus McGregor 1980 Lu Lichang, pl.5, 9-10

Tholisporites punctatus McGregor 1980 Lu Lichang, pl.5, 11

Thymospora sp. 1980 Lu Lichang, pl.9, 11

Triangulatisporites spp. 1991 Xu Ren et Gao Lianda, p.308

Trichodosporites delicatus Chi et Hills, 1976 1994 Xu Ren et Gao Lianda, pl.3, 4-5

Trichodosporites mirabilis 1994 1994 Xu Ren et Gao Lianda, pl.3, 2-3, 11

Verruciretusispora magnifica (McGregor) var. magnifica Owens 1980 Lu Lichang, pl.3, 1-3, 5-8

Verruciretusispora pallida (McGregor) Owens 1980 Lu Lichang, pl.2, 19-21; pl.3, 4

Verruciretusispora primus Chi et Hills, 1976 1994 Xu Ren et Gao Lianda, pl.1, 1

Verrucisporites arcticus var. *arcticus* Chi et Hills 1994 Xu Ren et Gao Lianda, p.120

Verrucisporites ellesmerensis (Choloner) var. *connactus* Chi et Hills, 1976 1994 Xu Ren et Gao Lianda, pl.1, 5

Verrucisporites luii (Lu et Ouyang) Xu Honghe et al. 2012 1978 Lu Lichang et Ouyang Shu, pl.2, 6-10; 1978 Lu Lichang et Ouyang Shu, pl.3, 5; 2012 Xu Honghe et al., fig. 2A-D

Verrucisporites lunanensis, 1994 1994 Xu Ren et Gao Lianda, pl.1, 6-7

Verrucisporites medius var. *magnus* Chi et Hills 1994 Xu Ren et Gao Lianda, p.120

Verrucisporites medius var. *medius* Chi et Hills, 1976 1994 Xu Ren et Gao Lianda, pl.1, 10

Verrucisporites medius var. *minutus* Chi et Hills, 1976 1994 Xu Ren et Gao Lianda, pl.1, 8-9

Verrucisporites reticuloides, 1994 1994 Xu Ren et Gao Lianda, pl.1, 4

Verrucisporites submamillaris (McGregor) Chi et Hills, 1976 1994 Xu Ren et Gao Lianda, pl.1, 2-3
Verrucosisporites confertus Owens 1980 Lu Lichang, pl.4, 7

2. Luquan: Xichong Formation

Acinosporites acanthomammillatus Richardson 1981 Gao Lianda, pl. I, 11
Ancyrospora imgnisa (Chibrikova) Gao 1981 Gao Lianda, pl. II, 23
Contagisporites optivus var. *vorobjevensis* (Chibrikova) Owens. 1981 Gao Lianda, pl. II, 13
Dibolisporites eifeliensis (Lanninger) McGregor 1981 Gao Lianda, pl. II, 21
Dictyotriletes subgranifer McGregor 1981 Gao Lianda, pl. I, 18
Hystrichosporites grandis Owens 1981 Gao Lianda, pl. III, 8
Lophozonotriletes tylophorus Naumova. 1981 Gao Lianda, pl. II, 14
Punctatisporites uniformis (Naumova) Gao 1981 Gao Lianda, pl. I, 4
Radiatisponospora langispinosa Gao 1981 Gao Lianda, pl. II, 19
Verruciretusispora macrotuberculata Schultz. 1981 Gao Lianda, pl. II, 10

3. Longhuashan Section, Zhanyi County: Upper Xichong Formation

Acinosporites acanthomammillatus Richardson 2005 Tian Jiajie et Zhu Huaicheng, p.312
Anapiculatisporites dilutus Lu 2005 Tian Jiajie et Zhu Huaicheng, p.312
Ancyrospora acuminata Lu 2005 Tian Jiajie et Zhu Huaicheng, p.312
Ancyrospora baccillaris Lu 2005 Tian Jiajie et Zhu Huaicheng, p.312
Ancyrospora subcircularis Lu 2005 Tian Jiajie et Zhu Huaicheng, p.312
Archaeoperisaccus indistinctus Lu 2005 Tian Jiajie et Zhu Huaicheng, p.312
Archaeozonotriletes timanicus Naumova 2005 Tian Jiajie et Zhu Huaicheng, p.312
Archaeozonotriletes variabilis (Naumova) Allen 2005 Tian Jiajie et Zhu Huaicheng, p.312
Auroraspora solisortus Hoffmeister, Staplin et Malloy 2005 Tian Jiajie et Zhu Huaicheng, p.312
Auroraspora torquata Higgs 2005 Tian Jiajie et Zhu Huaicheng, p.312
Camarozonotriletes convexus Lu 2005 Tian Jiajie et Zhu Huaicheng, p.312
Chelinospora concinna Allen 2005 Tian Jiajie et Zhu Huaicheng, p.312
Chelinospora ligurata Allen 2005 Tian Jiajie et Zhu Huaicheng, p.312
Corystisporites conicus Lu 2005 Tian Jiajie et Zhu Huaicheng, p.312
Corystisporites conoideus Lu 2005 Tian Jiajie et Zhu Huaicheng, p.312
Corystisporites multispinosus Richardson 2005 Tian Jiajie et Zhu Huaicheng, p.312
Cymbosporites catillus Allen 2005 Tian Jiajie et Zhu Huaicheng, p.312
Cymbosporites conatus Bharadwaj, Tiwari et Venkatachala 2005 Tian Jiajie et Zhu Huaicheng, p.312
Cymbosporites magnificus (McGregor) McGregor et Camfield 2005 Tian Jiajie et Zhu Huaicheng, p.312
Cyrtospora cristifer (Luber) van der Zwan 2005 Tian Jiajie et Zhu Huaicheng, p.312
Densosporites inaequus (McGregor) McGregor et Camfield 2005 Tian Jiajie et Zhu Huaicheng, p.312
Dibolisporites echinaceus (Eisenack) Richardson 2005 Tian Jiajie et Zhu Huaicheng, p.312
Grandispora cf. *inculta* Allen 2005 Tian Jiajie et Zhu Huaicheng, p.312
Grandispora distincta Lu 2005 Tian Jiajie et Zhu Huaicheng, p.312
Granulatisporites? *muninensis* Allen 2005 Tian Jiajie et Zhu Huaicheng, p.312
Hystricosporites microancyreus Riegel 2005 Tian Jiajie et Zhu Huaicheng, p.312
Lophotriletes minor Naumova 2005 Tian Jiajie et Zhu Huaicheng, p.312
Lophozonotriletes baculiformis Lu 2005 Tian Jiajie et Zhu Huaicheng, p.312

Lophozonotriletes polymorphus (Naumova) Lu 2005 Tian Jiajie et Zhu Huaicheng, p.312
Lophozonotriletes tylophorus Naumova 2005 Tian Jiajie et Zhu Huaicheng, p.312
Perotrilites conicus Lu 2005 Tian Jiajie et Zhu Huaicheng, p.312
Punctatisporites cf. *separatus* Lu 2005 Tian Jiajie et Zhu Huaicheng, p.312
Punctatisporites solidus Hacquebard 2005 Tian Jiajie et Zhu Huaicheng, p.312
Raistrickia clavata (Hacquebard) Playford 2005 Tian Jiajie et Zhu Huaicheng, p.312
Retusotriletes biarealis McGregor 2005 Tian Jiajie et Zhu Huaicheng, p.312
Rhabdosporites cf. *Parvulus* Richardson 2005 Tian Jiajie et Zhu Huaicheng, p.312
Samarisporites triangulatus Allen 2005 Tian Jiajie et Zhu Huaicheng, p.312
Stenozonotriletes inspissatus Owens 2005 Tian Jiajie et Zhu Huaicheng, p.312
Verrucosisporites (al. *Lophozonotriletes*) cf. *grandis* (Naumova) Richardson 2005 Tian Jiajie et Zhu Huaicheng, p.312
Verrucosisporites premnus Richardson 2005 Tian Jiajie et Zhu Huaicheng, p.312
Verrucosisporites scurrus (Naumova) McGregor et Camfield 2005 Tian Jiajie et Zhu Huaicheng, p.312

4. Shijiapo Section, Zhanyi County, Qujing City: Haikou Formation

Acinosporites acanthomammillatus Richardson, 1965 1988 Lu, pl. VII, 12, 13
Acinosporites pyramidatus Lu, 1981 1988 Lu, pl. VII, 15, 17-19
Acritosporites bilamellatus Lu, 1988 1988 Lu, pl. XXIV, 1, 2
Acritosporites singularis Lu, 1988 1988 Lu, pl. XXIV, 7-11
Acritosporites sp. 1988 Lu, pl. XXIV, 12
Anapiculatisporites dilutus Lu, 1980 1988 Lu, pl. II, 3-5
Anapiculatisporites minutus Lu et Ouyang, 1976 1988 Lu, pl. XXII, 16-19
Ancyrospora baccillaris Lu, 1988 1988 Lu, pl. XXV, 9, 10; fig. 17
Ancyrospora incisa (Naumova) Lu, 1988 1988 Lu, pl. XXII, 1, 7; fig. 16
Ancyrospora stellizonalis Lu, 1988 1988 Lu, pl. XXIX, 4, 5; fig. 18
Ancyrospora striata Lu, 1988 1988 Lu, pl. XXVIII, 1, 2; fig. 19
Ancyrospora tenuicaulis Lu, 1988 1988 Lu, pls. XXVI, 9; XXVII, 7, 8; XXIX, 3; fig. 20
Ancyrospora acuminata Lu, 1980 1988 Lu, pls. XXV, 1, 2; XXVII, 1; fig. 14
Ancyrospora arguta (Naumova) Lu, 1980 1988 Lu, pls. XXVI, 1; XXIX, 2; fig. 13
Ancyrospora cf. *subcircularis* Lu, 1980 1988 Lu, pl. XXVI, 4
Ancyrospora conjunctiva Lu, 1988 1988 Lu, pl. XXXII, 7-10; fig. 22
Ancyrospora dissecta Lu, 1988 1988 Lu, pls. XXXI, 1-3; 7-11; XXXIII, 12, 13; fig. 23
Ancyrospora distincta Lu, 1988 1988 Lu, pls. XI, 1; XXXII, 1-3; fig. 24
Ancyrospora irregularis Lu, 1988 1988 Lu, pl. XXXIII, 1-3; fig. 25
Ancyrospora melvillensis Owens, 1971 1988 Lu, pls. XXIV, 5; XXVII, 2; XXVIII, 3, 4; XXXI, 12, 13
Ancyrospora penicillata Lu, 1988 1988 Lu, pls. XXIII, 5; XXX, 1-4; fig. 26
Ancyrospora pulchra Owens, 1971
Ancyrospora simplex (Guennel) Urban 1969 1988 Lu, pls. XXIV, 3; XXVIII, 9, 10; XXXIII, 5
Ancyrospora subcircularis Lu, 1980 1988 Lu, pls. VIII, 6; XXII, 5, 6; XXIV, 4; XXV, 7, 8; fig. 15
Ancyrospora? majuscula Lu, 1988 1988 Lu, pl. XXIX, 1, 6; fig. 21
Apiculatasporites delicatus Lu, 1981 1988 Lu, pl. XXII, 8, 9
Apiculatisporis abditus (Loose) Potoni é et Kremp, 1955 1988 Lu, pls. X, 12, 13; XXVIII, 5
Apiculatisporis aculeatus (Ibr.) Potoni é et Kremp, 1955 1988 Lu, pls. VII, 6, 7; XX, 11
Apiculatisporis regularis Lu, 1988 1988 Lu, pl. XX, 13, 14

Apiculatisporis sp. 1988 Lu, pl. XI, 10
Apiculiretusispora crassa Lu, 1980 1988 Lu, pl. VI, 18, 19
Apiculiretusispora densa Lu, 1988 1988 Lu, pls. XXI, 17; XXVI, 6-8
Apiculiretusispora nitida Owens, 1971 1988 Lu, pls. VI, 1-4; X, 2
Archaeoperisaccus indistinctus Lu, 1988 1988 Lu, pl. XXXIV, 7, 8
Archaeoperisaccus scabratus Owens, 1971 1988 Lu, pl. XXXIV, 6
Archaeozonotriletes auritus Lu, 1980 1988 Lu, pl. XXV, 4, 11
Archaeozonotriletes dissectus Lu, 1988 1988 Lu, pl. XVIII, 12, 15
Archaeozonotriletes distinctus Lu, 1988 1988 Lu, pls. XVII, 12; XVIII, 11, 13, 14
Archaeozonotriletes incompletus Lu, 1988 1988 Lu, pls. XVII, 11; XVIII, 8
Archaeozonotriletes orbiculatus Lu, 1988 1988 Lu, pls. XVII, 9, 13; XVIII, 6, 7, 9, 10
Archaeozonotriletes splendidus Lu, 1981 1988 Lu, pl. XVII, 14
Archaeozonotriletes variabilis (Naumova) Allen, 1965 1988 Lu, pls. XVIII, 4; XXI, 5; XXXIII, 11
Brochotriletes sp. 1988 Lu, pl. XXXIII, 8
Calamospora intropunctata Lu, 1988 1988 Lu, pl. I, 7, 8, 14, 15
Calamospora microrugosa (Ibrahim) Schopf, Wilson et Bentall, 1944 1988 Lu, pl. II, 1, 2, 12
Calamospora normalis Lu, 1988 1988 Lu, pl. III, 1-4
Camarozonotriletes convexus Lu, 1988 1988 Lu, pl. XXII, 12-15
Camarozonotriletes microgranulatus Lu, 1981 1988 Lu, pl. XXI, 18, 19
Camarozonotriletes triangulatus Lu, 1988 1988 Lu, pl. XXIX, 9, 10
Camptotriletes rarus Lu, 1988 1988 Lu, pl. XXXIII, 9
Cereusisporites mirabilis Lu et Ouyang, 1978 1988 Lu, pls. X, 1; XVII, 8
cf. *Auroraspora* sp. 1 1988 Lu, pl. XXIX, 7, 8
cf. *Auroraspora* sp. 2 1988 Lu, pl. XXXIII, 7
Chelinospora irregulata Lu, 1980 1988 Lu, pl. VII, 1-4
Chelinospora larga Lu, 1988 1988 Lu, pl. XIX, 1-3
Chelinospora ligulata Allen, 1965 1988 Lu, pl. XXII, 3
Chelinospora multireticulata Lu, 1980 1988 Lu, pl. XIX, 7, 8, 15, 16
Chelinospora ochyrosa Lu, 1980 1988 Lu, pls. XIX, 11; XXII, 2
Chelinospora rarireticulata Lu, 1980 1988 Lu, pl. XIX, 9, 12
Chelinospora regularis Lu, 1988 1988 Lu, pls. XIX, 6, 10, 17, 18; XX, 10
Chelinospora robusta Lu, 1988 1988 Lu, pl. XIX, 4, 5, 14
Cingulatisporites? sp. 1988 Lu, pl. III, 12
Cingulizonates spongiformis Lu, 1988 1988 Lu, pls. XI, 7; XVI, 14-16
Convolutispora florida Hoffmeister et al. 1955 1988 Lu, pl. XXVI, 2, 3
Convolutispora tuberosa Winslow, 1962 1988 Lu, pl. XIV, 6, 7
Cornispora lagenifornis (Lu) Lu, 1988 1988 Lu, pls. II, 11; XI, 8; XXV, 12
Coronispora circularis Lu, 1988 1988 Lu, pls. XXIV, 6; XXVII, 3
Corystisporites conicus Lu, 1981 1988 Lu, pl. XV, 6
Corystisporites conoideus Lu, 1988 1988 Lu, pl. XV, 5, 9
Costazonotriletes latidentatus Lu, 1988 1988 Lu, pls. XII, 10, 11, 13, 14; XIII, 1, 2
Costazonotriletes navicularis Lu, 1988 1988 Lu, pls. XI, 4, 5; XIII, 3-6, 9, 10; XV, 1, 3, 4; fig. 6
Costazonotriletes verrucosus Lu, 1988 1988 Lu, pls. XI, 3; XII, 12; XIII, 7, 8; XV, 2
Crassispora cf. *imperfect* Lu, 1988 1988 Lu, pl. VIII, 3

Crassispora imperfecta Lu, 1988 1988 Lu, pl. III, 14-16
Crassispora kosankei (Pot. et Kr.) Smith et Butterworth, 1967 1988 Lu, pl. III, 13
Crassispora remota Lu, 1988 1988 Lu, pl. VI, 5, 6
Crassispora sp. 1988 Lu, pls. XXI, 9; XXXIII, 6
Cristatisporites sp. 1988 Lu, pl. XXVI, 5, 10
Cymbosporites arcuatus Bharadwaj et al., 1971 1988 Lu, pl. XVI, 11, 12
Cymbosporites conatus Bharadwaj et al., 1971 1988 Lu, pls. I, 21, 22; XXIII, 11-14
Cymbosporites cyathus Allen, 1965 1988 Lu, pl. XXX, 8, 9
Cymbosporites dentatus Lu 1990 1988 Lu, pl. XXXI, 4
Cymbosporites magnifica var. *endoformis* (Owens) Lu, 1988 1988 Lu, pl. VI, 15
Cymbosporites magnifica var. *magnifica* (Owens) Lu, 1988 1988 Lu, pl. VI, 9, 12-14
Cymbosporites microverrucosus Bharadwaj et al., 1971 1988 Lu, pls. V, 13-17; X, 9-11
Cymbosporites rhytideus Lu, 1988 1988 Lu, pls. X, 6-8; XXI, 11-13
Perotrilites conicus Lu, 1980 1988 Lu, pl. XII, 6, 7
Densosporites anulatus (Loose) Smith et Butterworth, 1967 1988 Lu, pl. XV, 13
Densosporites conicus Lu, 1988 1988 Lu, pls. V, 19, 20; XI, 9; XXVII, 10
Dibolisporites bifurcatus Lu, 1988 1988 Lu, pl. VII, 10, 11, 14, 16
Dibolisporites diaphanus Lu, 1988 1988 Lu, pl. VII, 8, 9
Dictyotriletes varius Naumova, 1953 1988 Lu, pl. VII, 5
Endosporites? sp. 1988 Lu, pl. XV, 7
Favispora conferta Lu, 1988 1988 Lu, pls. II, 8, 9; VIII, 1
Favispora langa Lu, 1988 1988 Lu, pl. IX, 1-4, 7-9
Favispora reticuloides (Lu et Ouyang) Lu, 1988 1988 Lu, pls. II, 6, 7; VIII, 11; X, 4, 5; fig. 4
Favispora rotunda Lu, 1988 1988 Lu, pl. IX, 5, 6, 10-12
Geminospora lemurata (Balme) Playford, 1983 1988 Lu, pl. XV, 12
Geminospora microdenta Lu, 1988 1988 Lu, pl. XVII, 5-7
Geminospora punctata Owens, 1971 1988 Lu, pl. XXVI, 11, 12
Grandispora distincta Lu, 1988 cf. *Auroraspora* sp.1 1988 Lu, pl. XII, 1-5
Grandispora sp. 1988 Lu, pl. XX, 9
Gulisporites sp. 1988 Lu, pl. I, 3
Hystricosporites cf. *gravis* Owens, 1971 1988 Lu, pl. XXIII, 7
Hystricosporites germinis Lu, 1981 1988 Lu, pl. VIII, 2, 7; fig. 9
Hystricosporites microancyreus Riegel 1973 1988 Lu, pl. XXVI, 13, 14
Hystricosporites sp. 1 1988 Lu, pl. XXIII, 6
Hystricosporites sp. 1 1988 Lu, pl. XXVI, 15
Hystricosporites sp. 2 1988 Lu, pl. XI, 15
Hystricosporites sp. 4 1988 Lu, pl. XXIII, 1; fig. 10
Hystricosporites triangulatus Tiwari et Schaarschmidt 1975 1988 Lu, pl. XXIII, 8-10
Leiotriletes dissimilis McGregor, 1960 1988 Lu, pl. I, 4-6
Leiotriletes sp. 1988 Lu, pl. I, 20
Longhuashanispora commixta Lu et Ouyang 1988 Lu, pl. X, 3
Lophotriletes incompletus Lu, 1988 1988 Lu, pl. XXII, 10, 11
Lophotriletes minor Naumova, 1953 1988 Lu, pls. XVI, 17-19; XVIII, 16-18
Lophozonotriletes baculiformis Lu, 1981 1988 Lu, pl. XX, 1, 2

Lophozonotriletes cristifer (Luber) Kedo, 1957 1988 Lu, pl. XXV, 5, 6
Lophozonotriletes irregularis Lu, 1988 1988 Lu, pls. II, 10; XVII, 1-3, 10
Lophozonotriletes mamillatus Lu, 1988 1988 Lu, pls. XXI, 6; XXV, 3
Lophozonotriletes polymorphus (Naumova) Lu, 1988 1988 Lu, pl. XX, 3, 4
Lophozonotriletes timanicus (Naumova) Lu, 1988 1988 Lu, pl. XX, 5, 6, 12
Lophozonotriletes verrucosus Lu, 1988 1988 Lu, pls. XIX, 13; XXI, 7, 10
Nikitinsporites brevicornis Lu, 1988 1988 Lu, pls. VIII, 4, 5; XI, 2; XXVII, 6; fig. 12
Nikitinsporites rhabdocladus Lu, 1988 1988 Lu, pls. XXIII, 2-4; XXXII, 4-6
Ocksisporites minisculus Lu, 1988 1988 Lu, pl. XVI, 8-10
Peltosporites imparilis Lu, 1988 1988 Lu, pl. XXVII, 4, 5
Peltosporites rotundus Lu, 1988 1988 Lu, pl. III, 10, 11
Peltosporites rugulosus Lu, 1988 1988 Lu, pl. III, 7, 8
Peltosporites sp. 1988 Lu, pl. VI, 7, 8
Punctatisporites glabrimarginatus Owens, 1971 1988 Lu, pls. I, 9, 11, 16
Punctatisporites planus Hacquebard, 1957 1988 Lu, pl. VI, 16, 17
Punctatisporites putaminis McGregor, 1960 1988 Lu, pl. I, 1, 2
Punctatisporites robustus Lu, 1988 1988 Lu, pl. XXX, 6, 7
Punctatisporites solidus Hacquebard, 1957 1988 Lu, pl. I, 12, 13
Punctatisporites sp. 1988 Lu, pl. I, 23, 24
Pustulatisporites paucispinus Lu, 1988 1988 Lu, pl. I, 17-19
Raistrickia crassa Lu, 1988 1988 Lu, pls. XIV, 8; XXI, 4, 8
Raistrickia incompleta Lu, 1981 1988 Lu, pl. XXVIII, 6, 7
Reticulatamonoletes angustus Lu, 1988 1988 Lu, pl. XXXIV, 13-17
Reticulatamonoletes robustus Lu, 1988 1988 Lu, pl. XXXIV, 9-12
Reticulatisporites sp. 1988 Lu, pl. XXX, 10, 11
Reticulatisporites translatus Lu, 1988 1988 Lu, pl. XVIII, 1-3, 5
Retusotriletes avonensis Playford, 1963 1988 Lu, pl. IV, 10
Retusotriletes communis Naumova, 1953 1988 Lu, pl. IV, 12
Retusotriletes confossus (Richardson) Lu et Ouyang, 1976 1988 Lu, pl. V, 1, 2
Retusotriletes densus Lu, 1988 1988 Lu, pl. III, 5, 6
Retusotriletes distinctus Richardson, 1965 1988 Lu, pls. IV, 7, 11; V, 12, 18
Retusotriletes dubiosus McGregor, 1973 1988 Lu, pl. IV, 8, 9
Retusotriletes impressus Lu, 1980 1988 Lu, pl. III, 9
Retusotriletes levidensus Lu, 1980 1988 Lu, pl. IV, 5, 6
Retusotriletes linearis Lu, 1988 1988 Lu, pl. XXI, 2, 3
Retusotriletes rugulatus Riegel 1973 1988 Lu, pl. V, 8, 9
Retusotriletes simplex Naumova, 1953 1988 Lu, pl. V, 6, 7
Retusotriletes spissus Lu, 1988 1988 Lu, pls. IV, 1-4; V, 10, 11
Retusotriletes triangulatus (Streel) Streel 1966 1988 Lu, pls. I, 10; V, 3-5
Rhabdosporites cf. *cymatilus* Allen, 1965 1988 Lu, pl. XX, 15
Rhabdosporites micropaxillus Owens, 1971 1988 Lu, pl. XV, 10, 11
Samarisporites heteroverrucosus Lu, 1980 1988 Lu, pl. XX, 7, 8
Samarisporites triangulatus Allen, 1965 1988 Lu, pl. VIII, 8-10
Stenozonotriletes clarus Ischenko 1988 Lu, pl. XVI, 1

Tholisporites densus McGregor, 1960 1988 Lu, pl. XVI, 2, 3
Tholisporites distalis Lu, 1981 1988 Lu, pl. XVI, 6, 7
Tholisporites interopunctatus Lu, 1988 1988 Lu, pl. XVI, 4, 5
Tholisporites sp. 1988 Lu, pl. XVI, 13
Thymospora imperfecta Lu, 1988 1988 Lu, pl. XXXIV, 1-3
Verruciretusispora platyverruca Lu et Ouyang, 1976 1988 Lu, pl. VI, 10, 11
Verrucizonotriletes distalis Lu, 1988 1988 Lu, pls. XII, 9; XIV, 1-3; fig. 7
Verrucizonotriletes triangulatus Lu, 1988 1988 Lu, pls. VII, 8, 15, 16; XIV, 4, 5; XV, 8
Verrucosisporites chilus Lu, 1988 1988 Lu, pl. XI, 13, 14
Verrucosisporites nitidus (Naumova) Playford, 1964 1988 Lu, pls. XXVIII, 8; XXXI, 6
Verrucosisporites sp. 1 1988 Lu, pl. XXIV, 13, 14
Verrucosisporites sp. 2 1988 Lu, pl. XXXI, 5

Guangxi

Donggangling Formation

Lagenicula sp. 1981 Gao Lianda, pl. III, 3
Punctatisporites gigantus (Naumova) Gao 1981 Gao Lianda, pl. I, 6
Retusotriletes triangulatus (Streel) Allen 1981 Gao Lianda, pl. I, 10
Rhabdosporites micropaxillus Owens 1981 Gao Lianda, pl. III, 4

Frasnian

Yunnan

1. Panxi, Huaning County

Ancyrospora ampulla Owens 1994 Xu Ren et Gao Lianda, p.120
Ancyrospora ancyrea 1991 Xu Ren et Gao Lianda, pl.309
Ancyrospora furcata 1991 Xu Ren et Gao Lianda, pl.309
Ancyrospora fuscula Owens 1994 Xu Ren et Gao Lianda, p.120
Ancyrospora involucra Owens 1994 Xu Ren et Gao Lianda, p.120
Ancyrospora melvillensis Owens 1994 Xu Ren et Gao Lianda, p.120
Aneurospora goensis 1991 Xu Ren et Gao Lianda, pl.309
Aneurospora greggsii 1991 Xu Ren et Gao Lianda, pl.309
Apiculiretusispora granulata 1991 Xu Ren et Gao Lianda, pl.308
Apiculiretusispora nitida 1991 Xu Ren et Gao Lianda, pl.308
Archaeoperisaccus ovalis Naumova 1991 Xu Ren et Gao Lianda, pl.II, 11
Auroraspora macromanifestus 1991 Xu Ren et Gao Lianda, pl.309
Camarozonotriletes devonicus Naumova 1991 Xu Ren et Gao Lianda, pl.I, 11
Contagisporites potivus var. *optivus* 1991 Xu Ren et Gao Lianda, p.307
Cristatisporites triangulates (Allen) McGregor et Camfield 1991 Xu Ren et Gao Lianda, pl.II, 5
Cymbosporites cyathus Allen 1991 Xu Ren et Gao Lianda, pl.I, 27
Cymbosporites magnificus (McGregor) McGregor et Camfield 1991 Xu Ren et Gao Lianda, pl.I, 28
Geminospora lemurata (Balme) Playford 1991 Xu Ren et Gao Lianda, pl.I, 26

Geminospora venusta (Naumova) McGregor et Camfield 1991 Xu Ren et Gao Lianda, pl.I, 22-23
Hymenozonotriletes argutus 1991 Xu Ren et Gao Lianda, pl.309
Hymenozonotriletes rarus 1991 Xu Ren et Gao Lianda, p.306
Hymenozonotriletes spp. 1991 Xu Ren et Gao Lianda, pl.309
Hystrichosporites multifurcatus 1991 Xu Ren et Gao Lianda, pl.309
Lagenicula bulbosa Chi et Hills 1994 Xu Ren et Gao Lianda, p.120
Lagenicula devonica var. *devonica* Chi et Hills 1994 Xu Ren et Gao Lianda, p.120; 1991 Xu Ren et Gao Lianda, p.307
Lagenicula spp. 1991 Xu Ren et Gao Lianda, pl.309
Lohpozonotriletes media 1991 Xu Ren et Gao Lianda, pl.309
Perotriletes aculeatus 1991 Xu Ren et Gao Lianda, pl.309
Pustulatisporites rugulatus 1991 Xu Ren et Gao Lianda, pl.309
Rhabdosporites parvus 1991 Xu Ren et Gao Lianda, pl.309
Samarisporites concinnus 1991 Xu Ren et Gao Lianda, pl.309
Samarisporites inaequus 1991 Xu Ren et Gao Lianda, p.307
Verrucisporites ellesmerensis var. *connatus* Chi et Hills 1994 Xu Ren et Gao Lianda, p.120
Verrucisporites medius var. *medius* Chi et Hills 1994 Xu Ren et Gao Lianda, p.120
Verrucisporites medius var. *minutus* Chi et Hills 1994 Xu Ren et Gao Lianda, p.120
Verrucisporites submamillaris (McGregor) Chi et Hills 1994 Xu Ren et Gao Lianda, p.120
Verrucosisporites bullatus 1991 Xu Ren et Gao Lianda, pl.309

2. Huaning: Yidade Formation

Ancyrospora ancyrea Richardson 1981 Gao Lianda, p. 21
Ancyrospora simplex Guennel 1981 Gao Lianda, p. 21
?*Archaeoperisaccus* sp. 1981 Gao Lianda, p. 21
Archaeotriletes sp. 1981 Gao Lianda, p. 21
Cymbosporites echinatus (Naumova) Gao 1981 Gao Lianda, p. 21
Geminospora sp. 1981 Gao Lianda, p. 21
Hymenozonotriletes argutus Naumova 1981 Gao Lianda, pl. III, 10
Hystricosporites sp. 1981 Gao Lianda, p. 21
Radiatispinospora radiata Bharadwaj 1981 Gao Lianda, p. 21

3. Xiaolengka Section, Huaping County

Acinosporites acanthomammillatus Rich. 1981 Lu, pl.3, 7-8
Acinosporites pyramidatus Lu, 1981 1981 Lu, pl.3, 9-11
Ancyrospora melvillensis Owens 1981 Lu, pl.8, 3-5
Ancyrospora aff. *incisa* (Naum.) Lu, 1981 Lu, pl.8, 6
Ancyrospora cf. *acuminata* Lu, 1981 Lu, pl.8, 1-2
Ancyrospora robusta Lu, 1981 1981 Lu, pl.9, 9-10
Apiculatisporis variabilis Lu, 1981 1981 Lu, pl.2, 9-10
Archaeozonotriletes variabilis (Naum.) Allen 1981 Lu, pl.9, 3-5
Archaeozonotriletes splendidus Lu, 1981 1981 Lu, pl.9, 6-8; pl.10, 4
Camarozonotriletes parvus Owens 1981 Lu, pl.7, 5-7
Convolutispora distincta Lu, 1981 1981 Lu, pl.3, 4-5
Convolutispora sp. 1981 Lu, pl.3, 6

Corystisporites conicus Lu, 1981 1981 Lu, pl.5, 4
Cymbosporites catillus Allen 1981 Lu, pl.10, 3
Densosporites crassus McGr. 1981 Lu, pl.6, 7-8
Diaphanospora crassa Lu, 1981 1981 Lu, pl.6, 5-6
Dissizonotriletes acutangulatus Lu, 1981 1981 Lu, pl.7, 11-12
Dissizonotriletes stenodes Lu, 1981 1981 Lu, pl.7, 13
Hystricosporites sp. 1981 Lu, pl.5, 7; pl.10, 13
Ocksisporites sp. 1981 Lu, pl.9, 11
Proprisporites reticulatus Lu, 1981 1981 Lu, pl.6, 3-4
Punctatisporites separatus Lu, 1981 1981 Lu, pl.1, 4
Punctatisporites sp. 1981 Lu, pl.1, 5
Retusotriletes imparilis Lu, 1981 1981 Lu, pl.2, 1-2
Rotaspora interornata Lu, 1981 1981 Lu, pl.7, 1-4
Verruciretusispora magnifica (McGr.) Owens var. *magnifica* Owens 1981 Lu, pl.4, 3-6
Verruciretusispora pallida (McGr.) Owens 1981 Lu, pl.4, 7-9
Verruciretusispora magnifica var. *endoformis* (McGr.) Owens 1981 Lu, pl.4, 10-11

Sichuan

Damaidi Section, Dukou City

Ancyrospora melvillensis Owens 1981 Lu, pl.8, 3-5
Apiculatasporites delicatus Lu, 1981 1981 Lu, pl.2, 3-4
Apiculatisporis aculeatus (Ibr.) Pot. et Kr. 1981 Lu, pl.2, 7-8
Apiculatisporis variabilis Lu, 1981 1981 Lu, pl.2, 9-10
Apiculiretusispora granulata Owens 1981 Lu, pl.4, 1
Apiculiretusispora crassa Lu, 1981 Lu, pl.4, 2
Apiculiretusispora microverrucosa (Bhard. et al.) Lu, 1981 Lu, pl.3, 12-13
Archaeoperisaccus microancyra Lu, 1981 1981 Lu, pl.10, 9-10
Archaeozonotriletes variabilis (Naum.) Allen 1981 Lu, pl.9, 3-5
Asperispora scabra Lu, 1981 1981 Lu, pl.7, 17-18
Asperispora verrucosa Lu, 1981 1981 Lu, pl.7, 20-21
Calamospora cf. *mutabilis* (Loose) Schopf et al. 1981 Lu, pl.1, 6-7
Calamospora cf. *microrugosa* (Ibr.) Schopf et al. 1981 Lu, pl.1, 8-9
Calamospora sp. 1981 Lu, pl.1, 10
Camarozonotriletes parvus Owens 1981 Lu, pl.7, 5-7
Camarozonotriletes microgranulatus Lu, 1981 1981 Lu, pl.7, 8-10
Canthospora patula Winslow 1981 Lu, pl.6, 15
Convolutispora distincta Lu, 1981 1981 Lu, pl.3, 4-5
Corystisporites longispinosus Lu, 1981 1981 Lu, pl.5, 3
Cycadopites sp. 1981 Lu, pl.10, 11
Cyclogranisporites dukouensis Lu, 1981 1981 Lu, pl.2, 5-6
Cymbosporites cyathus Allen 1981 Lu, pl.10, 3
Cymbosporites catillus Allen 1981 Lu, pl.10, 1-2
Densosporites rotundus Lu, 1981 1981 Lu, pl.6, 9-10

Densosporites cordatus Lu, 1981 1981 Lu, pl.6, 11-12
Densosporites trilamellatus Lu, 1981 1981 Lu, pl.6, 13-14
Diaphanospora crassa Lu, 1981 1981 Lu, pl.6, 5-6
Dibolisporites conoideus Lu, 1981 1981 Lu, pl.4, 13-14
Dibolisporites cf. *conoideus* Lu, 1981 1981 Lu, pl.5, 1-2
Galeatisporites laevigatus Lu, 1981 1981 Lu, pl.7, 19, 22, 23
Gulisporites intropunctatus Lu, 1981 1981 Lu, pl.1, 1
Hystricosporites germinis Lu, 1981 1981 Lu, pl.5, 5-6
Hystricosporites sp. 1981 Lu, pl.5, 7; pl.10, 13
Latosporites sp. A 1981 Lu, pl.10, 5-6
Latosporites sp.B 1981 Lu, pl.10, 7-8
Lophozonotriletes torosus Naum. 1981 Lu, pl.7, 14
Lophozonotriletes curvatus Naum. 1981 Lu, pl.5, 8
Lophozonotriletes baculiformis Lu, 1981 1981 Lu, pl.7, 15-16
Punctatisporites aerarius Butt. et Will. 1981 Lu, pl.1, 2-3
Pustulatisporites triangulatus Lu, 1981 1981 Lu, pl.2, 11-12
Pustulatisporites distalis Lu, 1981 1981 Lu, pl.2, 13-14; pl.10, 12
Raistrickia levis Lu, 1981 1981 Lu, pl.2, 15-16
Raistrickia incompleta Lu, 1981 1981 Lu, pl.3, 1-3
Rhabdosporites zonofossulatus Lu, 1981 1981 Lu, pl.6, 1-2
Retusotriletes simplex Naum. 1981 Lu, pl.1, 11
Retusotriletes communis Naum. 1981 Lu, pl.1, 12
Retusotriletes triangulatus (Str.) Str. var. *major* Lu et Ouyang 1981 Lu, pl.1, 13-15
Retusotriletes levidensus Lu, 1981 Lu, pl.1, 16-17
Rotaspora interornata Lu, 1981 1981 Lu, pl.7, 1-4
Tholisporites separatus Lu, 1981 1981 Lu, pl.9, 1-2
Tholisporites distalis Lu, 1981 1981 Lu, pl.8, 8-9
Tholisporites sp. 1981 Lu, pl.8, 7
Verrucisporites luii (Lu et Ouyang) Xu Honghe et al., 2012 1981 Lu, pl.4, 12; pl.10, 14

Hubei

1. Miliangshan Section, Wuhan City: Luojia Series, layer 1-5

Acinosporites pyramidatus 2000 Li Chengsen, p. 6
Anapiculatisporites juvongensis 2000 Li Chengsen, p. 5
Ancyrospora carnarvonensis 2000 Li Chengsen, p. 6
Ancyrospora langii 2000 Li Chengsen, p. 6
Ancyrospora melvillensis 2000 Li Chengsen, p. 6
Ancyrospora parva 2000 Li Chengsen, p. 6
Apiculiretusispora brandtii 2000 Li Chengsen, p. 5
Auroraspora hyaline 2000 Li Chengsen, p. 6
Auroraspora macra 2000 Li Chengsen, p. 6
Biharisporites sp. 2000 Li Chengsen, p. 6
Chelinospora irregulata 2000 Li Chengsen, p. 6

Chelinospora multireticulata 2000 Li Chengsen, p. 6
Chelinospora ochyrosa 2000 Li Chengsen, p. 6
Corystisporites multispinosus 2000 Li Chengsen, p. 6
Cyrtospora cristifer 2000 Li Chengsen, p. 6
Diducites mucronatus 2000 Li Chengsen, p. 6
Geminospora nanus 2000 Li Chengsen, p. 5
Hystricosporites sp. 2000 Li Chengsen, p. 6
Longhuashanispora commixta 2000 Li Chengsen, p. 6
Murospora dubiata 2000 Li Chengsen, p. 6
Punctatisporites anisoletus 2000 Li Chengsen, p. 5
Rhabdosporites zonofossulatus 2000 Li Chengsen, p. 6
Verruciretusispora magnifica var. *magnifica* 2000 Li Chengsen, p. 5
Verruciretusispora semilucensis 2000 Li Chengsen, p. 5
Verrucisporites luii (Lu et Ouyang) Xu Honghe et al., 2012 2000 Li Chengsen, p. 6

2. Liujiachanghuangkuang Section, Songzi County: Huangjiadeng Formation

Acinosporites acanthomannillatus Richardson 1984 Feng Shaonan, pl.50, 21
Ancyrospora simplex Guennel 1984 Feng Shaonan, pl.51, 23
Aneurospora greggsii (McGregor) Streel 1984 Feng Shaonan, pl.50, 27-28; pl.51, 6
Apiculiretusispora kurta Gao et Zhong, 1984 1984 Feng Shaonan, pl.50, 7
Archaeozonotriletes variabilis (Naumova) Allen 1984 Feng Shaonan, pl.51, 16
Ballatisporites ballatus Allen 1984 Feng Shaonan, pl.50, 19
Biornatispora dentate Lele et Streel 1984 Feng Shaonan, pl.50, 22-23
Calamospora microrugosa (Ibrahim) Schopf, Wilson et Bentall 1984 Feng Shaonan, pl.50, 1
Convolutispora cerrecta (Naumova) Gao et Zhong, 1984 1984 Feng Shaonan, pl.50, 15
Cristatisporites echinatus Playford 1984 Feng Shaonan, pl.51, 7
Cymbosporites cyathus Allen 1984 Feng Shaonan, pl.51, 3-5
Grandispora melianida (Naumova) Taugourdeau-Lantz 1984 Feng Shaonan, pl.51, 26
Hymenozonotriletes denticulatus Naumova 1984 Feng Shaonan, pl.51, 24
Laevigatosporites sp. 1984 Feng Shaonan, pl.51, 18
Raistrickia hathra Gao et Zhong, 1984 1984 Feng Shaonan, pl.50, 11
Raistrickia sp. 1984 Feng Shaonan, pl.50, 13
Retusotriletes communis Naumova 1984 Feng Shaonan, pl.50, 4
Retusotriletes hubaiensis Cao et Zhong, 1984 1984 Feng Shaonan, pl.50, 5
Samarisporites songzinensis Gao et Zhong, 1984 1984 Feng Shaonan, pl.51, 22
Samarisporites spinosus Gao et Zhong, 1984 1984 Feng Shaonan, pl.51, 19-20
Tholisporites sp. 1984 Feng Shaonan, pl.51, 15
Verruciretusispora livenensus (Naumova) Gao et Zhong, 1984 1984 Feng Shaonan, pl.50, 24
Verruciretusispora vermiculata Gao et Zhong, 1984 1984 Feng Shaonan, pl.51, 8

3. Luoyanshan Section, Changyang County: Huangjiadeng Formation

Ancyrospora melvillensis 1985 Liu Shuwen et Gao Lianda, p. 114
Apiculiretusispora granulata 1985 Liu Shuwen et Gao Lianda, p. 114
Archaeotriletes conicus Gao, 1985 Liu Shuwen et Gao Lianda, pl. II, 21

Archaeotriletes magnificus Gao, 1985 1985 Liu Shuwen et Gao Lianda, pl. II, 3
Cymbosporites canthoceus (Naum.) Gao, 1985 1985 Liu Shuwen et Gao Lianda, pl. II, 9
Discernisporites micromanifestus Gao, 1985 1985 Liu Shuwen et Gao Lianda, pl. II, 20
Gemnospora regularis (Naum.) Gao, 1985 1985 Liu Shuwen et Gao Lianda, pl. II, 11
Grandispora eximius (Naum.) Gao, 1985 1985 Liu Shuwen et Gao Lianda, pl. II, 15
Grandispora krestovinikovii (Naum.) Gao, 1985 1985 Liu Shuwen et Gao Lianda, pl. II, 16
Grandispora medius (Naum.) Gao, 1985 1985 Liu Shuwen et Gao Lianda, pl. II, 17
Grandispora mesodevonicus (Naum.) Gao, 1985 1985 Liu Shuwen et Gao Lianda, pl. II, 22
Hymenozonotriletes turbinatus (Naum.) Gao, 1985 1985 Liu Shuwen et Gao Lianda, pl. II, 19
Hystricosporites devonicus 1985 Liu Shuwen et Gao Lianda, p. 114
Perotriletes aculeatus 1985 Liu Shuwen et Gao Lianda, p. 114
Perotriletes minor 1985 Liu Shuwen et Gao Lianda, p. 114
Retusotriletes triverrucosus Gao, 1985 1985 Liu Shuwen et Gao Lianda, pl. II, 1
Samarisporites concinnus 1985 Liu Shuwen et Gao Lianda, p. 114
Samarisporites granulatus Gao, 1985 1985 Liu Shuwen et Gao Lianda, pl. II, 13
Samarisporites minutus Gao, 1985 1985 Liu Shuwen et Gao Lianda, pl. II, 14
Samarisporites tozeri 1985 Liu Shuwen et Gao Lianda, p. 114
Samarisporites triangulatus 1985 Liu Shuwen et Gao Lianda, p. 114
Verruciretusispora magnifica 1985 Liu Shuwen et Gao Lianda, p. 114

Late Famennian

Jiangxi

Xiaomu Section, Chengxiang Town, Quannan County: Sanmentan Formation

Acanthotriletes denticulatus Naumova 1993 Wen Zicai et Lu Lichang, pl.1, 20-22
Ancyrospora sp. 1993 Wen Zicai et Lu Lichang, pl.3, 10
Aneurospora rarispinosa Wen et Lu, 1993 1993 Wen Zicai et Lu Lichang, pl.3, 9, 20
Apiculiretusispora gannanensis Wen et Lu, 1993 1993 Wen Zicai et Lu Lichang, pl.2, 1-6
Apiculiretusispora rarissima Wen et Lu, 1993 1993 Wen Zicai et Lu Lichang, pl.2, 7-10
Auroraspora poljessica (Kedo) Streel 1993 Wen Zicai et Lu Lichang, pl.3, 17
Bascaudaspora sp.1 1993 Wen Zicai et Lu Lichang, pl.3, 6
Cymbosporites circinatus Ouyang et Chen 1993 Wen Zicai et Lu Lichang, pl.3, 13-14
Diaphanospora depressa (Balme et Hassell) Evans 1993 Wen Zicai et Lu Lichang, pl.3, 18
Grandispora macrospinosa (Jush.) var. *punctata* (Jush.) Wen et Lu, 1993 1993 Wen Zicai et Lu Lichang, pl.2, 25
Grandispora echinata Hacquebard 1993 Wen Zicai et Lu Lichang, pl.3, 19
Grandispora sp. 1993 Wen Zicai et Lu Lichang, pl.4, 2
Granulatisporites minimus Wen et Lu, 1993 1993 Wen Zicai et Lu Lichang, pl.1, 28-29
Leiotriletes laevis Naumova 1993 Wen Zicai et Lu Lichang, pl.1, 4-5
Leiotriletes microrugosus (Ibr.) Naumova 1993 Wen Zicai et Lu Lichang, pl.1, 2-3
Retispora? cf. *lepidophyta* (Kedo) Playford 1993 Wen Zicai et Lu Lichang, pl.4, 13-15
Retusotriletes crassus Clayton et al. 1993 Wen Zicai et Lu Lichang, pl.1, 18
Retusotriletes minor Kedo 1993 Wen Zicai et Lu Lichang, pl.1, 19
Retusotriletes simplex Naumova 1993 Wen Zicai et Lu Lichang, pl.1, 24-25

Spelaeotriletes resolutus Higgs 1993 Wen Zicai et Lu Lichang, pl.4, 3-6

Hunan

1. Xinhua: Xikuangshan Formation

Cymbosporites parvibasilaris (Naumova) Gao 1981 Gao Lianda, pl. II, 22

2. Tianmenya Village, Shimen County: Tizikou Formation (Strunian)

Aneurospora greggsii (McGregor) Streel 1984 Feng Shaonan, pl.50, 27-28; pl.51, 6
Archaeoperisaccus scabratus Owens 1984 Feng Shaonan, pl.51, 28-29
Calamospora atava (Naumova) McGregor 1984 Feng Shaonan, pl.50, 2
Clivosisporites variabilis Staplin et Jansonius 1984 Feng Shaonan, pl.51, 9
Cristatisporites cf. *orcadensis* Richardson, 1965 1992 Gao Lianda, pl. III, 21
Densosporites spitsbergensis Playford, 1963 1992 Gao Lianda, pl. III, 8-9
Dibolisporites sp. 1992 Gao Lianda, pl. I, 16
Dictyotriletes submarginatus Playford, 1964 1990 Gao Lianda, pl. I, 16
Dictyotriletes trivialis Naumova in Kedo 1984 Feng Shaonan, pl.50, 18
Discernisporites micromanifestus (Hacquebard) Sabry et Neves, 1971 1990 Gao Lianda, pl. II, 13
Geminospora sp. 1992 Gao Lianda, pl. II, 14
Hymenozonotriletes explanatus (Luber) Kedo, 1963 1992 Gao Lianda, pl. IV, 15-16
Knoxisporites literatus (Waltz) Playford, 1963 1992 Gao Lianda, pl. I, 32
Knoxisporites literatus (Waltz) Playford var. *triangulatus* (Kedo) Clayton, 1970 1992 Gao Lianda, pl. II, 1
Pustulatisporites gibberosus (Hacquebard) Playford, 1964 1990 Gao Lianda, pl. I, 12
Raistrickia macrura (Luber) Dolby et Neves 1984 Feng Shaonan, pl.50, 12
Retispora lepidophyta (Kedo) Playford 1984 Feng Shaonan, pl.51, 17
Retusotriletes incohatus Sullivan, 1964 1992 Gao Lianda, pl. I, 18
Retusotriletes simplex Naumova 1984 Feng Shaonan, pl.50, 3
Samarisporites concinnus Owens, 1971 1990 Gao Lianda, pl. II, 4
Samarisporites hunanensis Gao, 1992 1992 Gao Lianda, pl. III, 19
Samarisporites inaequus (McGregor) Owens, 1971 1990 Gao Lianda, pl. II, 3
Spelaeotriletes arenaceus Neves et Ioannides, 1974 1992 Gao Lianda, pl. III, 20
Tumulispora malevkensis (Naumova) Turnau 1984 Feng Shaonan, pl.51, 10
Tumulispora variverrucata (Playford) Staplin et Jansonius, 1964 1992 Gao Lianda, pl. III, 7
Tumulispora variverrucata (Playford) Staplin et Jansonius, 1964 1990 Gao Lianda, pl. I, 23
Vallatisporites pusillites (Kedo) Dolby et Neves, 1970 1990 Gao Lianda, pl. II, 14-15
Vallatisporites pusillites (Kedo) Dolby et Neves, 1971 1992 Gao Lianda, pl. IV, 8
Vallatisporites pusillites (Kedo) Dolby et Neves 1984 Feng Shaonan, pl.51, 14
Vallatisporites sp. 1992 Gao Lianda, pl. IV, 14
Vallatisporites vallatus Hacquebard, 1957 1990 Gao Lianda, pl. II, 16-17
Vallatisporites vallatus Hacquebard, 1957 1992 Gao Lianda, pl. IV, 9-12
Vallatisporites vallatus Hacquebard 1984 Feng Shaonan, pl.51, 13
Vallatisporites verrucosus Hacquebard 1984 Feng Shaonan, pl.51, 12
Verruciretusispora magnifica (McGregor) var. *magnifica* Owens 1984 Feng Shaonan, pl.50, 25
Verrucosisporites aepressus Winlsow, 1962 1992 Gao Lianda, pl. I, 12

3. Oujiachong Section, Lengshuijiang City: Magunao Formation and Oujiachong Formation

Acanthotriletes cf. *tenuispinosus* 1982 Hou Jingpeng, pl.1, 16
Acanthotriletes crenatus 1982 Hou Jingpeng, p.84
Apiculiretusispora plicata Allen (Streel) 1982 Hou Jingpeng, pl.1, 8
Cyclogranisporites sp. 1982 Hou Jingpeng, p.84
Cymbosporites parvibasilaris (Naumova) Gao 1982 Hou Jingpeng, pl.2, 22
Dictyotriletes sp. 1982 Hou Jingpeng, pl.1, 18
Granulatisporites hunanensis Hou, 1982 1982 Hou Jingpeng, pl.1, 12-13
Hymenozonotriletes sp. 1982 Hou Jingpeng, pl.2, 23
Leiotriletes ornatus Ischenko 1982 Hou Jingpeng, pl.1, 2
Lophotriletes sp. 1982 Hou Jingpeng, p.84
Punctatisporites debilis Hacquebard 1982 Hou Jingpeng, pl.1, 3
Punctatisporites galber 1982 Hou Jingpeng, pl.1, 4
Punctatisporites planus 1982 Hou Jingpeng, p.84
Punctatisporites sp. 1982 Hou Jingpeng, pl.2, 24
Retusotriletes cf. *avonensis* Playford 1982 Hou Jingpeng, pl.1, 22
Retusotriletes incohatus 1982 Hou Jingpeng, p.84
Retusotriletes sp. 1982 Hou Jingpeng, pl.1, 6
Spelaeotriletes lepidophytus (Kedo) Streel 1982 Hou Jingpeng, pl.2, 13
Spinozonotriletes cf. *conspicuus* 1982 Hou Jingpeng, pl.2, 11
Spinozonotriletes uncatus (Hacquebard) Playford 1982 Hou Jingpeng, pl.2, 12
Verrucosisporites cf. *grumosus* (Naumova) Sullivan 1982 Hou Jingpeng, pl.1, 17
Verrucosisporites sp. 1982 Hou Jingpeng, pl.1, 23

4. Oujiachong Section, Lengshuijiang City: Lower Shaodong Formation

Acanthotriletes sp. 1982 Hou Jingpeng, pl.1, 15
Apiculiretusispora plicata (Allen) Streel 1982 Hou Jingpeng, pl.1, 8
Cristatisporites sp. 1982 Hou Jingpeng, pl.2, 10
Cyclogranisporites sp. 1982 Hou Jingpeng, p.84
Densosporites sp. 1982 Hou Jingpeng, pl.2, 7
Granulatisporites hunanensis Hou, 1982 1982 Hou Jingpeng, pl.1, 12-13
Leiotriletes inermis Ischenko 1982 Hou Jingpeng, pl.1, 1
Leiotriletes ornatus Ischenko 1982 Hou Jingpeng, pl.1, 2
Lophotriletes sp. 1982 Hou Jingpeng, p.84
Punctatosporites sp. 1982 Hou Jingpeng, p.84
Punctatisporites debilis Hacquebard 1982 Hou Jingpeng, pl.1, 3
Punctatisporites galber 1982 Hou Jingpeng, pl.1, 4
Punctatisporites planus 1982 Hou Jingpeng, p.84
Punctatisporites sp. 1982 Hou Jingpeng, pl.2, 24
Retusotriletes incohatus 1982 Hou Jingpeng, p.84
Retusotriletes sp. 1982 Hou Jingpeng, pl.1, 6
Spelaeotriletes lepidophytus (Kedo) Streel 1982 Hou Jingpeng, pl.2, 13
Verrucosisporites sp. 1981 Hou Jingpeng, pl.1, 23

Oujiachong Section, Lengshuijiang City: Upper Shaodong Formation

Acanthotriletes sp. 1982 Hou Jingpeng, pl.1, 15
Aneurospora greggsii 1987 Wang Genxian et al., p. 300-301
Apiculatisporis sp. 1982 Hou Jingpeng, pl.1, 14
Apiculiretusispora flexuosa Hou, 1982 1982 Hou Jingpeng, pl.1, 11
Apiculiretusispora oujiachongensis Hou, 1982 1982 Hou Jingpeng, pl.1, 9-10
Apiculiretusispora plicata 1987 Wang Genxian et al., p. 300-301
Apiculiretusispora plicata (Allen) Streel 1982 Hou Jingpeng, pl.1, 8
Asperispora rispora 1987 Wang Genxian et al., p. 300-301
Convalatispora sp. 1982 Hou Jingpeng, p.84
Cyclogranisporites sp. 1982 Hou Jingpeng, p.84
Cymbosporites spp. 1987 Wang Genxian et al., p. 300-301
Densosporites cf. *anulatus* (Loose) Smith et Butterworth 1982 Hou Jingpeng, pl.2, 6
Densosporites runcinatus 1987 Wang Genxian et al., p. 300-301
Densosporites sp. 1982 Hou Jingpeng, pl.2, 7
Densosporites xinhuanensis Hou, 1982 1982 Hou Jingpeng, pl.2, 5
Dibolisporites sp. 1982 Hou Jingpeng, pl.1, 20
Dictyotriletes cf. *trivialis* (Naumova) Kedo 1982 Hou Jingpeng, pl.1, 19
Discernisporites micromanifestus Hacquebard 1982 Hou Jingpeng, pl.2, 19
Grandispora cornuta 1987 Wang Genxian et al., p. 300-301
Grandispora gracilis 1987 Wang Genxian et al., p. 300-301
Grandispora sp.1 1982 Hou Jingpeng, pl.2, 14
Grandispora tenuispinosa 1987 Wang Genxian et al., p. 300-301
Grandispora uniformis Hou, 1982 1982 Hou Jingpeng, pl.2, 15-16
Granulatisporites hunanensis 1987 Wang Genxian et al., p. 300-301
Hymenozonotriletes explanatus 1987 Wang Genxian et al., p. 300-301
Hymenozonotriletes sp. 1982 Hou Jingpeng, pl.2, 23
Hymenozonotriletes sp. 1987 Wang Genxian et al., p. 300-301
Knoxisporites literatus (Waltz) Playford 1982 Hou Jingpeng, pl.1, 21
Knoxisporites literatus 1987 Wang Genxian et al., p. 300-301
Leiotriletes inermis Ischenko 1982 Hou Jingpeng, pl.1, 1
Loiotriletes ornatus 1987 Wang Genxian et al., p. 300-301
Lophotriletes gramosus 1987 Wang Genxian et al., p. 300-301
Lophotriletes sp. 1982 Hou Jingpeng, p.84
Lophozonotriletes curratus 1987 Wang Genxian et al., p. 300-301
Lophozonotriletes curvatus Naumova 1982 Hou Jingpeng, pl.2, 20
Lophozonotriletes cyclophymatus Hou, 1982 1982 Hou Jingpeng, pl.2, 3
Lophozonotriletes gramosus 1987 Wang Genxian et al., p. 300-301
Lophozonotriletes malevkensis (Naum.) Kedo 1982 Hou Jingpeng, pl.2, 1
Lophozonotriletes rarituberculatus (Luber) Kedo 1982 Hou Jingpeng, pl.2, 4
Lophozonotriletes sp. 1982 Hou Jingpeng, p.84
Lophozonotriletes zhushanensis Hou, 1982 1982 Hou Jingpeng, pl.2, 2
Punctatisporites glaber 1987 Wang Genxian et al., p. 300-301
Punctatisporites solidus 1987 Wang Genxian et al., p. 300-301

Punctatisporites sp. 1982 Hou Jingpeng, pl.2, 24
Punctatosporites sp. 1982 Hou Jingpeng, p.84
Punctatisporites debilis Hacquebard 1982 Hou Jingpeng, pl.1, 3
Punctatisporites galber 1982 Hou Jingpeng, pl.1, 4
Punctatisporites planus 1982 Hou Jingpeng, p.84
Retispora lepidophyta 1987 Wang Genxian et al., p. 300-301
Retusotriletes asthenolabratus Hou, 1982 1982 Hou Jingpeng, pl.1, 5, 7
Retusotriletes communis 1987 Wang Genxian et al., p. 300-301
Retusotriletes incohatus 1987 Wang Genxian et al., p. 300-301
Retusotriletes sp. 1987 Wang Genxian et al., p. 300-301
Tumulisporites varituberculatus 1987 Wang Genxian et al., p. 300-301
Vallatisporites batiambes Hou, 1982 1982 Hou Jingpeng, pl.2, 8
Vallatisporites pusillites 1987 Wang Genxian et al., p. 300-301
Vallatisporites sp.1 1982 Hou Jingpeng, pl.2, 9
Vallatisporites sp.2 1982 Hou Jingpeng, pl.2, 18
Vallatisporites vallatus 1987 Wang Genxian et al., p. 300-301
Verrucosisporites cf. *grumosus* 1987 Wang Genxian et al., p. 300-301
Verrucosisporites nitidus 1987 Wang Genxian et al., p. 300-301
Verrucosisporites sp. 1982 Hou Jingpeng, pl.1, 23
Verrucosisporites sp. 1987 Wang Genxian et al., p. 300-301

5. Xinhua: Lower Shaodong Formation

Grandispora cornuta Higgs, 1975 1990 Gao Lianda, pl. I, 13
Grandispora cornuta Higgs, 1975 1990 Gao Lianda, pl. II, 20
Grandispora echinata Hacquebard, 1957 1990 Gao Lianda, pl. I, 25
Retispora lepidophyta (Kedo) Playford var. *tener* Kedo et Golubtsov 1971 1990 Gao Lianda, pl. I, II, 9
Retusotriletes crassus Clayton, 1970 1990 Gao Lianda, pl. I, 2
Verrucosisporites cf. *nitidus* (Naumova) Playford, 1964 1990 Gao Lianda, pl. I, 6

6. Simaowan Section, Xinhua County: Lower Shaodong Formation

Geminospora nanus (Naumova) Gao, 1983 1990 Gao Lianda, pl. II, 5
Grandispora cornuta Higgs, 1975 1990 Gao Lianda, pl. II, 20
Retispora lepidophyta (Kedo) Playford var. *tener* Kedo et Golubtsov 1971 1990 Gao Lianda, pl. II, 9

Simaowan Section, Xinhua County: Upper Shaodong Formation

Acanthotriletes sp. 1987 Wang Genxian et al., p. 301-302
Acanthotriletes sp. 1987 Wang Genxian et al., p. 301-302
Acinosporites acanthomammillatus Richardson, 1965 1990 Gao Lianda, pl. I, 14
Calamospora sp. 1987 Wang Genxian et al., p. 301-302
Cymbosporites formusos 1987 Wang Genxian et al., p. 301-302
Cymbosporites sp. 1987 Wang Genxian et al., p. 301-302
Densosporites sp. 1987 Wang Genxian et al., p. 301-302
Densosporites spitsbergensis 1987 Wang Genxian et al., p. 301-302
Geminospora echinata 1987 Wang Genxian et al., p. 301-302

Geminospora parvibilarisa 1987 Wang Genxian et al., p. 301-302
Geminospora serenusa 1987 Wang Genxian et al., p. 301-302
Grandispora echinata 1987 Wang Genxian et al., p. 301-302
Hymenozonotriletes commutatus Naumova, 1953 1990 Gao Lianda, pl. II, 19
Hymenozonotriletes explanatus (Luber) Kedo, 1963 1987 Wang Genxian et al., p. 301-302; 1990 Gao Lianda, pl. II, 7
Hymenozonotriletes sp. 1987 Wang Genxian et al., p. 301-302
Lophozonotriletes sp. 1990 Gao Lianda, pl. I, 27; 1987 Wang Genxian et al., p. 301-302
Retispora lepidophyta (Kedo) Playford, 1976 1987 Wang Genxian et al., p. 301-302; 1990 Gao Lianda, pl. II, 10-12
Retispora sp. 1987 Wang Genxian et al., p. 301-302
Retispora vervucosus 1987 Wang Genxian et al., p. 301-302
Retusotriletes sp. 1987 Wang Genxian et al., p. 301-302
Rugospora flexuosa (Juschko) Streel in B. B. S. T., 1974 1990 Gao Lianda, pl. I, 15
Samarisporites sp. 1987 Wang Genxian et al., p. 301-302
Spelaeotriletes crustatus Higgs, 1975 1990 Gao Lianda, pl. I, 28
Tumulispora rarituberculata 1987 Wang Genxian et al., p. 301-302
Vallatisporites pusillites (Kedo) Dolby et Neves, 1970 1987 Wang Genxian et al., p. 301-302; 1990 Gao Lianda, pl. II, 14-15
Vallatisporites verrucosus Hacquebard, 1957 1990 Gao Lianda, pl. II, 18
Verrucosisporites sp. 1987 Wang Genxian et al., p. 301-302

Xinhua: Menggong'ao Formation

Anapiculatisporites delicatus Neves et Ioannides, 1974 1990 Gao Lianda, pl. I, 19
Convolutispora venusta Hoffmeister, Staplin et Malloy 1953 1990 Gao Lianda, pl. I, 6
Convolutispora vermiformis Hughes et Playford, 1961 1990 Gao Lianda, pl. I, 11
Densosporites spitsbergensis Playford, 1963 1990 Gao Lianda, pl. I, 20
Knoxisporites literatus (Waltz) Playford, 1963 1990 Gao Lianda, pl. I, 17
Punctatisporites irrasus Hacquebard, 1957 1990 Gao Lianda, pl. I, 1
Retusotriletes planus Dolby et Neves, 1970 1990 Gao Lianda, pl. I, 3
Tumulispora variverrucata (Playford) Staplin et Jansonius, 1964 1990 Gao Lianda, pl. I, 23
Vallatisporites cf. *pusillites* (Kedo) Dolby ex Neves, 1970 1990 Gao Lianda, pl. II, 22
Verrucosisporites cf. *nitidus* (Naumova) Playford, 1964 1990 Gao Lianda, pl. I, 6

7. Malanbian Section, Xinshao County: Oujiachong Formation

Anapiculatisporites minutus Lu et Ouyang 1987 Yang Yuncheng, pl. 27, 16
Apiculiretusispora kurta Gao 1987 Yang Yuncheng, pl. 27, 15
Apiculiretusispora plicata (Allen) Streel 1987 Yang Yuncheng, pl. 27, 14
Cymbosporites formosus (Naumova) Gao 1987 Yang Yuncheng, pl. 27, 27-28
Retispora lepidophyta (Kedo) Playford 1987 Yang Yuncheng, pl. 28, 1-5
Spelaeotriletes hunanensis (Fang et al.) comb. nov., emend. 1993 Fang Xiaosi et al., pl. I.

Malanbian Section, Xinshao County: Upper Shaodong Formation

Spelaeotriletes hunanensis (Fang et al.) comb. nov., emend. 1993 Fang Xiaosi et al., pl. I.
Spelaeotriletes resolutus Higgs, 1975 1990 Gao Lianda, pl. I, 29

Malanbian Section, Xinshao County: Menggong'ao Formation

Acanthotriletes sp. 1 1987 Yang Yuncheng, pl. 27, 18
Acanthotriletes sp. 2 1987 Yang Yuncheng, pl. 27, 21
Apiculiretusispora minuta Lu et Ouyang 1987 Yang Yuncheng, pl. 27, 11-12
Apiculiretusispora oujiachongensis Hou 1987 Yang Yuncheng, pl. 27, 13
Archaeozonotriletes famenensis Naumova 1987 Yang Yuncheng, pl. 27, 24
Archaeozonotriletes variabilis (Naumova) Allen 1987 Yang Yuncheng, pl. 27, 29
Auroraspora hyalina (Naumova) Streel in Beker et al. 1987 Yang Yuncheng, pl. 28, 24
Auroraspora macra Sullivan 1987 Yang Yuncheng, pl. 28, 23
Calamospora atava (Naumova) McGregor 1987 Yang Yuncheng, pl. 27, 2
Calamospora divisa Gao 1987 Yang Yuncheng, pl. 27, 3
Calamospora nigrata (Naumova) Allen 1987 Yang Yuncheng, pl. 27, 1
Convolutispora implicata (Kedo) Gao 1987 Yang Yuncheng, pl. 27, 20
Densosporites spitsbergensis Playford 1987 Yang Yuncheng, pl. 27, 30-31
Densosporites xinhuanensis Hou 1987 Yang Yuncheng, pl. 27, 25-26
Dibolisporites sp. 1987 Yang Yuncheng, pl. 27, 17
Discernisporites micromanifestus (Hacquebard) Sabry et Neves 1987 Yang Yuncheng, pl. 28, 26
Geminospora nanus (Naumova) Gao 1987 Yang Yuncheng, pl. 27, 23
Grandispora famenensis (Naumova) Streel 1987 Yang Yuncheng, pl. 28, 21
Grandispora meonacanthus Naumova 1987 Yang Yuncheng, pl. 28, 19
Grandispora multispinosa Gao 1987 Yang Yuncheng, pl. 28, 20
Granulatisporites hunanensis Hou 1987 Yang Yuncheng, pl. 27, 8-10
Granulatisporites rotundus (Naumova) Gao 1987 Yang Yuncheng, pl. 27, 7
Hymenozonotriletes explanatus (Luber) Kedo 1987 Yang Yuncheng, pl. 28, 18
Laevigatosporites sp. 1 1987 Yang Yuncheng, pl. 28, 28
Lophozonotriletes grumosus Naumova 1987 Yang Yuncheng, pl. 27, 22
Retispora lepidophyta (Kedo) Playford 1987 Yang Yuncheng, pl. 28, 1-5
Retusotriletes asthenolabratus Hou 1987 Yang Yuncheng, pl. 27, 5-6
Retusotriletes simplex Naumova 1987 Yang Yuncheng, pl. 27, 4
Spelaeotriletes hunanensis (Fang et al.) comb. nov., emend. 1993 Fang Xiaosi et al., pl. I.
Spelaeotriletes resolatus Higgs 1987 Yang Yuncheng, pl. 28, 22
Tumulispora rarituberculata (Luber) Potoni é1987 Yang Yuncheng, pl. 27, 32-33
Vallatisporites pusillites (Kedo) Dolby et Neves 1987 Yang Yuncheng, pl. 28, 8-11
Vallatisporites vallatus Hacquebard 1987 Yang Yuncheng, pl. 28, 14-17
Vallatisporites verrucosus Hacquebard 1987 Yang Yuncheng, pl. 28, 12-13
Verrucosisporites nitidus (Naumova) Playford 1987 Yang Yuncheng, pl. 27, 19

8. Liujiatang Section, near Jieling Town, Shaodong County: Shaodong Formation

Acanthotriletes (Lophotriletes) fastuosus (Naumova) Lu, 1995 1995 Lu Lichang, p. 41
Acanthotriletes denticulatus Naumova, 1953 1995 Lu Lichang, p. 41
Acanthotriletes hirtus Naumova, 1953 1995 Lu Lichang, p. 41
Acanthotriletes impolitus 1997 Lu Lichang, p. 187
Anapiculatisporites hystricosus Playford, 1964 1995 Lu Lichang, p. 41
Anaplanisporites globutus (Butterworth et Williams) Streel et Butterworth, 1967 1995 Lu Lichang, p. 41
Ancyrospora cf. *furcula* Owens, 1971 1995 Lu Lichang, p. 41

Aneurospora asthenolabrata (Hou) Lu, 1994 1995 Lu Lichang, p. 41
Aneurospora spinulifer Wen et Lu, 1993 1995 Lu Lichang, p. 41
Apiculatisporis morbosus Balme et Hassell, 1962 1995 Lu Lichang, p. 41
Apiculiretusispora flexuosa Hou, 1982 1995 Lu Lichang, p. 41
Apiculiretusispora gannanensis Wen et Lu, 1993 1995 Lu Lichang, p. 41
Apiculiretusispora leberidos McGregor et Camfield, 1982 1995 Lu Lichang, p. 41
Apiculiretusispora plicata (Allen) Streel, 1967 1995 Lu Lichang, p. 41
Apiculiretusispora pseudozonalis Lu, 1980 1995 Lu Lichang, p. 41
Archaeozonotriletes aconthaceus 1997 Lu Lichang, p. 187
Auroraspora asperella (Kedo) Van der Zwan, 1980 1995 Lu Lichang, p. 41
Auroraspora macra Sullivan, 1964 1995 Lu Lichang, p. 41
Baculatisporites fusticulus 1997 Lu Lichang, p. 187
Baculatisporites villosus 1997 Lu Lichang, p. 187
Brochotriletes foveolatus Naumova, 1953 1995 Lu Lichang, p. 41
Camptoriletes triangulatus Lu, 1997 1997 Lu Lichang, p. 187
Camptozonotriletes proximalis Lu, 1997 1997 Lu Lichang, p. 187
Cirratriradites veeversi 1997 Lu Lichang, p. 187
Clivosispora verrucata McGregor var. *verrucata* McGregor 1976 1995 Lu Lichang, p. 41
Convolutispora ampla Hoffmeister, Staplin et Malloy, 1955 1995 Lu Lichang, p. 41
Convolutispora balmei Playford, 1971 1995 Lu Lichang, p. 41
Convolutispora crassa Playford, 1962 1995 Lu Lichang, p. 41
Convolutispora major (Kedo) Turnau, 1978 1995 Lu Lichang, p. 41
Convolutispora subtilis 1997 Lu Lichang, p. 187
Convolutispora vermiformis Hughes et Playford, 1961 1995 Lu Lichang, p. 41
Cordylosporites papillatus (Naumova) Playford et Satterthwait, 1985 1995 Lu Lichang, p. 41
Crassispora imperfecta Lu, 1988 1995 Lu Lichang, p. 41
Cristatisporites connexus 1997 Lu Lichang, p. 187
Cristatisporites digitatus Lu, 1997 1997 Lu Lichang, p. 187
Cristatisporites limitatus Ouyang et Chen, 1987 1995 Lu Lichang, p. 41
Cyclogranisporites baoyingensis Ouyang et Chen, 1987 1995 Lu Lichang, p. 41
Cyclogranisporites pisticus Playford, 1978 1995 Lu Lichang, p. 41
Cycloverrutriletes sp. 1997 Lu Lichang, p. 187
Cymbosporites cf. *formosus* 1997 Lu Lichang, p. 187
Cymbosporites cyathus Allen, 1965 1995 Lu Lichang, p. 41
Cymbosporites microgranulatus Lu, 1997 1997 Lu Lichang, p. 187
Densosporites capistratus Hoffmeister, Staplin et Malloy, 1955 1995 Lu Lichang, p. 41
Densosporites crassus McGregor, 1960 1995 Lu Lichang, p. 41
Densosporites rariabilis 1997 Lu Lichang, p. 187
Densosporites rarispinosus 1997 Lu Lichang, p. 187
Densosporites secundus Playford et Satterthwait 1988 1995 Lu Lichang, p. 41
Densosporites sp. 1997 Lu Lichang, p. 187
Densosporites spinifer Hoffmeister, Staplin et Malloy, 1955 1995 Lu Lichang, p. 41
Densosporites variomarginatus Playford, 1963 1995 Lu Lichang, p. 41
Densosporites xinhuanensis Hou, 1982 1995 Lu Lichang, p. 41

Diaphanospora (Hymenozonotriletes) submirabilis (Jush.) Lu, 1995 1995 Lu Lichang, p. 41
Diaphanospora depressa (Balme et Hassell) Evans 1970 1995 Lu Lichang, p. 41
Dictyotriletes famenensis Naumova, 1953 1995 Lu Lichang, p. 41
Discernisporites deminutus Lu, 1997 1997 Lu Lichang, p. 187
Discernisporites macromanifestus (Hacquebard) Higgs, Clayton et Keegan, 1988 1995 Lu Lichang, p. 41
Discernisporites micromanifestus (Hacquebard) Sabry et Nevens, 1971 1995 Lu Lichang, p. 41
Discernisporites papillatus Lu, 1993 1995 Lu Lichang, p. 41
Discernisporites suspictus Lu, 1997 1997 Lu Lichang, p. 187
Discernisporites usitatus Lu, 1997 1997 Lu Lichang, p. 187
Discernisporites varius Lu, 1993 1995 Lu Lichang, p. 41
Foveosporites insculptus Playford, 1962 1995 Lu Lichang, p. 41
Foveosporites pellucidus Playford et Helby 1968 1995 Lu Lichang, p. 41
Geminospora (Archaeozonotriletes) lasius (Naumova) var. *minor* (Naumova) Lu 1995 1995 Lu Lichang, p. 41
Geminospora lemurata 1997 Lu Lichang, p. 187
Geminospora micropaxilla 1997 Lu Lichang, p. 187
Geminospora multiramis Lu, 1997 1997 Lu Lichang, p. 187
Geminospora spongiata Higgs, Clayton et Keegan 1988 1995 Lu Lichang, p. 41
Geminospora venusta 1997 Lu Lichang, p. 187
Grandispora (Hymenozonotriletes) eximia (Naumova) Lu 1995 1995 Lu Lichang, p. 41
Grandispora (Spelaeotriletes?) cumula (Higgs et Streel) Lu 1995 1995 Lu Lichang, p. 41
Grandispora cornuta Higgs, 1975 1995 Lu Lichang, p. 41
Grandispora echinata Hacquebard, 1957 1995 Lu Lichang, p. 41
Grandispora furcata Lu, 1997 1997 Lu Lichang, p. 187
Grandispora gracilis (Kedo) Streel, 1974 1995 Lu Lichang, p. 41
Grandispora saurota (Higgs, Clayton et Keegan) Playford et McGregor 1993 1995 Lu Lichang, p. 41
Grandispora serena (Kedo) Lu, 1997 1997 Lu Lichang, p. 187
Granulatisporites (Spinozonotriletes) atratus (Naumova) Lu 1995 1995 Lu Lichang, p. 41
Granulatisporites humerus Staplin, 1960 1995 Lu Lichang, p. 41
Gulisporites hiatus Lu, 1997 1997 Lu Lichang, p. 187
Hymenospora sp. 1997 Lu Lichang, p. 187
Hymenozonotriletes angulatus Naumova, 1953 1995 Lu Lichang, p. 41
Hymenozonotriletes elegans (Waltz) Naumova, 1953 1995 Lu Lichang, p. 41
Hymenozonotriletes granulatus (Naumova) Jushen in Kedo, 1963 1995 Lu Lichang, p. 41
Hymenozonotriletes spicatus Lu, 1994 1995 Lu Lichang, p. 41
Knoxisporites dedaleus (Naumova) Lu, 1994 1995 Lu Lichang, p. 41
Laevigatosporites vulgaris (Ibrahim) Alpern et Doubinger, 1973 1995 Lu Lichang, p. 41
Leiotriletes cf. *subintertus* (Waltz) Ischenko var. *rotundatus* Waltz, 1941 1995 Lu Lichang, p. 41
Leiotriletes crassus Lu, 1994 1995 Lu Lichang, p. 41
Leiotriletes macrothelis Wen et Lu, 1993 1995 Lu Lichang, p. 41
Leiotriletes microthelis Wen et Lu, 1993 1995 Lu Lichang, p. 41
Leiotriletes ornatus Ischenko, 1956 1995 Lu Lichang, p. 41
Leiotriletes pyramidatus Sullivan, 1964 1995 Lu Lichang, p. 41
Leiotriletes sp. 1997 Lu Lichang, p. 187
Leiotriletes velatus (Caro-Monieg) Streel, 1974 1995 Lu Lichang, p. 41

Lophotriletes atratus Naumova, 1953 1995 Lu Lichang, p. 41
Lophotriletes magnus (Naumova) Lanniger 1968 1995 Lu Lichang, p. 41
Lophozonotriletes torosus Naum. var. *famenensis* Naumova, 1953 1995 Lu Lichang, p. 41
Lycospora uber 1997 Lu Lichang, p. 187
Microreticulatisporites distinctus (Naumova, in Kedo) Lu, 1997 1997 Lu Lichang, p. 187
Microreticulatisporites reticuloides 1997 Lu Lichang, p. 187
Phyllothecotriletes rigidus Playford, 1962 1995 Lu Lichang, p. 41
Planisporites magnus (Naumova) Lu, 1994 1995 Lu Lichang, p. 41
Punctatisporites cornatus 1997 Lu Lichang, p. 187
Punctatisporites debilis Hacquebard, 1957 1995 Lu Lichang, p. 41
Punctatisporites irrasus Hacquebard, 1957 1995 Lu Lichang, p. 41
Punctatisporites limbatus 1997 Lu Lichang, p. 187
Punctatisporites planus Hacquebard, 1957 1995 Lu Lichang, p. 42
Punctatisporites subtritus Playford et Helby, 1968 1995 Lu Lichang, p. 42
Pustulatisporites distalis Lu, 1981 1995 Lu Lichang, p. 42
Raistrickia sp. 1997 Lu Lichang, p. 187
Reticulatisporites (*Azonotriletes*) *cancellothyris* (Waltz) Lu, 1995 1995 Lu Lichang, p. 42
Reticulatisporites amplectus (Naum.) Lu, 1997 1997 Lu Lichang, p. 187
Reticulatisporites ancoralis 1997 Lu Lichang, p. 187
Reticulatisporites minor (Naumova) Gao et Ye, 1987 1995 Lu Lichang, p. 42
Retispora cassicula (Higgs) Higgs et Russell, 1981 1995 Lu Lichang, p. 42
Retispora lepidophyta (Kedo) Playford, 1976 1995 Lu Lichang, p. 42
Retusotriletes avonensis Playford, 1963 1995 Lu Lichang, p. 42
Retusotriletes crassus Clayton in Clayton et al., 1980 1995 Lu Lichang, p. 42
Retusotriletes rotundus (Streel) Lele et Streel, 1969 1995 Lu Lichang, p. 42
Retusotriletes triangulatus (Streel) Streel, 1967 1995 Lu Lichang, p. 42
Schopfites cf. *claviger* 1997 Lu Lichang, p. 187
Spelaeotriletes crenulatus (Playford) Higgs, Clayton et Keegan 1988 1995 Lu Lichang, p. 42
Spelaeotriletes crustatus Higgs, 1975 1995 Lu Lichang, p. 42
Spelaeotriletes fanxiaensis Lu, 1997 1995 Lu Lichang, p. 42; 1997 Lu Lichang, p. 187
Spelaeotriletes hetermorphus Lu, 1997 1997 Lu Lichang, p. 187
Spelaeotriletes hunanensis (Fang, Steemans et Streel) Lu, 1994 1994 Lu Lichang, pls. I, 1-15; II, 1-15; 1995 Lu Lichang, p. 42
Spelaeotriletes microgranulatus var. *minor* 1997 Lu Lichang, p. 187
Spelaeotriletes microspinosus 1995 Lu Lichang, p. 42; 1996 Lu Lichang, p. 187
Spelaeotriletes obtusus Higgs, 1975 1995 Lu Lichang, p. 42
Spelaeotriletes pretiosus (Playford) Neves et Belt, 1970 1995 Lu Lichang, p. 42
Spelaeotriletes rarus Lu, 1997 1997 Lu Lichang, p. 187
Spelaeotriletes setosus (Kedo) Lu, 1994 1995 Lu Lichang, p. 42
Spelaeotriletes triangulatus Neves et Owens, 1966 1995 Lu Lichang, p. 42
Stenozonotriletes extensus var. *major* 1997 Lu Lichang, p. 187
Tumulispora sp. 1997 Lu Lichang, p. 187
Tumulispora ordinaria 1997 Lu Lichang, p. 187
Velamisporites irrugatus Playford, 1978 1995 Lu Lichang, p. 42

Velamisporites perinatus Knox (Hughes et Playford) Playford, 1971 1995 Lu Lichang, p. 42
Velamisporites sp. 1997 Lu Lichang, p. 187
Verruciretusispora megaplatyverruca Lu et Ouyang, 1976 1995 Lu Lichang, p. 42
Verrucosisporites mesogrumosus (Kedo) Byvscheva, 1985 1995 Lu Lichang, p. 42
Verrucosisporites morulatus (Knox) Potonié et Kremp, 1954 1995 Lu Lichang, p. 42
Verrucosisporites papulosus Hacquebard, 1957 1995 Lu Lichang, p. 42
Videospora? *glabrimarginata* (Owens) Higgs et Russell, 1981 1995 Lu Lichang, p. 42

9. Oujiachong Section, Lengshuijiang City: Shaodong Formation

Acanthotriletes cf. *denticulatus* Naumova 1996 Wang, pl.1, 21
Acanthotriletes similis Naumova 1996 Wang, pl.2, 4-5
Acanthotriletes tenuispinosus Waltz 1996 Wang, pl.1, 19
Acanthotriletes intonsus Playford 1996 Wang, pl.1, 16-17
Anapiculatisporites concinnus Playford 1996 Wang, pl.1, 20
Anapiculatisporites juyongensis Ouyang et Chen 1996 Wang, pl.2, 1
Anapiculatisporites hystricosus Playford 1996 Wang, pl.1, 13
Aneurospora erinacesis Wang, 1996 1996 Wang, pl.3, 18-19
Aneurospora greggsii (McGregor) Streel 1996 Wang, pl.3, 16-17
Apiculiretusispora hunanensis (Hou) Ouyang et Chen 1996 Wang, pl.2, 8-9
Apiculiretusispora microverrucosa (Bharadwaj et al.) Lu 1996 Wang, pl.2, 6-7
Apiculiretusispora fructiosa Higgs 1996 Wang, pl.2, 10-11
Auroraspora macra Sullivan 1996 Wang, pl.6, 6
Calamospora divisa Gao et Hou 1996 Wang, pl.1, 1-2
Calamospora cf. *microrugosa* Schopf, Wilson et Bentall 1996 Wang, pl.1, 3
Camptotriletes cf. *certus* Venkatachala et Bharadwaj 1996 Wang, pl.3, 7
Convolutispora tesselata Hoffmeister, Staplin et Malloy 1996 Wang, pl.2, 19-20
Cordylosporites papillatus (Naumova) Playford et Satterthwait 1996 Wang, pl.3, 4
Corystisporites multispinosus Richardson 1996 Wang, pl.4, 6-7
Crassispora spinogranulata Wang, 1996 1996 Wang, pl.2, 26-28
Crissisporites guangxinensis Wang, 1996 1996 Wang, pl.3, 8-9
Cristatisporites acutus Wang, 1996 1996 Wang, pl.3, 22-23
Cristatisporites reticulatus Wang, 1996 1996 Wang, pl.5, 16-17
Cristatisporites limitatus Ouyang et Chen 1996 Wang, pl.5, 18-19
Cymbosporites chinensis Ouyang et Chen 1996 Wang, pl.2, 14-15
Densosporites rarispinosus Playford 1996 Wang, pl.5, 5-7
Densosporites gracilis Smith et Butterworth 1996 Wang, pl.4, 1-2
Densosporites tenuis Hoffmeister, Staplin et Malloy 1996 Wang, pl.5, 1-3
Dictyotriletes distinctus Naumova in Kedo 1996 Wang, pl.3, 6
Dictyotriletes trivialis Naumova in Kedo 1996 Wang, pl.3, 3
Diducites versabilis (Kedo) Van Veen 1996 Wang, pl.4, 12
Endosporites velatus Wang, 1996 1996 Wang, pl.6, 16
Endosporites micromanifestus Hacquebard 1996 Wang, pl.6, 19-20
Geminospora lemurata Balme 1996 Wang, pl.5, 4
Grandispora echinata Hacquebard 1996 Wang, pl.4, 14-15

Granulatisporites frustulentus (Balme et Hassell) Playford 1996 Wang, pl.2, 2-3
Granulatisporites crenulatus Playford 1996 Wang, pl.2, 12-13
Hymenozonotriletes rarispinosus Lu 1996 Wang, pl.5, 8-12
Hymenozonotriletes tenellus Naumova 1996 Wang, pl.5, 13
Knoxisporites literatus (Waltz) Playford 1996 Wang, pl.6, 10
Kraeuselisporites cf. *echinatus* Owens, Mishell et Marshall 1996 Wang, pl.6, 12
Laevigatosporites minor Loose 1996 Wang, pl.6, 4-5
Leiotriletes confertus McGregor 1996 Wang, pl.1, 8
Leiotriletes simplex Naumova 1996 Wang, pl.1, 5
Lophotriletes erinaceus (Waltz et Naumova) Zhang 1996 Wang, pl.1, 22-23
Lophozonotriletes excisus Naumova 1996 Wang, pl.2, 16-17
Microreticulatisporites regulatus Wang, 1996 1996 Wang, pl.3, 5
Punctatisporites recavus Ouyang et Chen 1996 Wang, pl.1, 6
Punctatisporites camaratus Ouyang et Chen 1996 Wang, pl.1, 7
Punctatisporites glaber (Naumova) Playford 1996 Wang, pl.1, 10
Radiizonates camarosus Wang, 1996 1996 Wang, pl.6, 7-9
Radiizonates irregulatus Wang, 1996 1996 Wang, pl.6, 13-14
Radiizonates radianus Wang, 1996 1996 Wang, pl.5, 14
Radiizonates regulatus Wang, 1996 1996 Wang, pl.5, 15
Raistrickia variabilis Dolby et Neves 1996 Wang, pl.2, 21
Raistrickia clavata (Hacquebard) Playford 1996 Wang, pl.2, 25
Reticulatisporites cancellatus (Waltz) Playford 1996 Wang, pl.3, 1
Reticulatisporites rudis Staplin 1996 Wang, pl.3, 2
Reticulatisporites perlotus (Naumova) Ouyang et Chen 1996 Wang, pl.3, 10
Retispora lepidophyta (Kedo) Playford 1996 Wang, pl.3, 11-15
Retusisporites communis Naumova 1996 Wang, pl.1, 18
Retusisporites digressus Playford 1996 Wang, pl.1, 24
Retusisporites simplex Naumova 1996 Wang, pl.1, 9
Retusisporites asthenolabratus Hou 1996 Wang, pl.1, 14-15
Rugospora cf. *flexuosa* (Jusch.) Streel in B. B. S. T. 1996 Wang, pl.6, 11
Spelaeotriletes echinatus (Luber) Ouyang et Chen 1996 Wang, pl.1, 11-12
Spelaeotriletes pretiosus (Playford) Neves et Belt 1996 Wang, pl.2, 24
Spinozonotriletes saurotus Higgs, Clayton et Keegan 1996 Wang, pl.2, 22-23
Stenozonotriletes facilis var. *paraecrassus* Ischenko 1996 Wang, pl.4, 13
Tumulispora rarituberculata (Luber) Playford 1996 Wang, pl.4, 5
Vallatisporites sp. 1996 Wang, pl.4, 8-10
Vallatisporites cf. *pusillites* (Kedo) Dolby et Neves 1996 Wang, pl.4, 4
Vallatisporites cf. *vallatus* Hacquebard 1996 Wang, pl.4, 3
Velamisporites circulus Wang, 1996 1996 Wang, pl.6, 3
Velamisporites perinatus (Hughes et Playford) Playford 1996 Wang, pl.6, 1-2
Verrucosisporites cf. *dejerseyi* Playford 1996 Wang, pl.2, 18

Oujiachong Section, Lengshuijiang City: Lower–Middle Menggong'ao Formation

Acanthotriletes cf. *dentoculatus* Naumova 1996 Wang, pl.1, 21

Acanthotriletes intonsus Playford 1996 Wang, pl.1, 16-17
Acanthotriletes similis Naumova 1996 Wang, pl.2, 4-5
Aneurospora greggsii (McGregor) Streel 1996 Wang, pl.3, 16-17
Apiculiretusispora fructiosa 1996 Wang, p.16
Apiculiretusispora fructiosa Higgs 1996 Wang, pl.2, 10-11
Apiculiretusispora hunanensis (Hou) Ouyang et Chen 1996 Wang, pl.2, 8-9
Auroraspora macra Sullivan 1996 Wang, pl.6, 6
Calamospora cf. *microrugosa* Schopf, Wilson et Bentall 1996 Wang, pl.1, 3
Calamospora divisa Gao et Hou 1996 Wang, pl.1, 1-2
Calyptosporites minor Wang, 1996 1996 Wang, pl.4, 11
Camptotriletes cf. *certus* Venkatachala et Bharadwaj 1996 Wang, pl.3, 7
Convolutispora sp. 1987 Wang Genxian et al. et al., p. 299
Convolutispora tesselata Hoffmeister, Staplin et Malloy 1996 Wang, pl.2, 19-20
Cordylosporites papillatus (Naumova) Playford et Satterthwait 1996 Wang, pl.3, 4
Corystisporites multispinosus Richardson 1996 Wang, pl. 4, 6-7
Crissisporites guangxinensis Wang, 1996 1996 Wang, pl.3, 8-9
Cristatisporites acutus Wang, 1996 1996 Wang, pl.3, 22-23
Cristatisporites limitatus Ouyang et Chen 1996 Wang, pl.5, 18-19
Cymbosporites chinensis Ouyang et Chen 1996 Wang, pl.2, 14-15
Densosporites gracilis Smith et Butterworth 1996 Wang, pl.4, 1-2
Densosporites rarispinosus Playford 1996 Wang, pl.5, 5-7
Densosporites spitsbergensis 1987 Wang Genxian et al. et al., p. 299
Densosporites tenuis Hoffmeister, Staplin et Malloy 1996 Wang, pl.5, 1-3
Dictyotriletes distinctus Naumova in Kedo 1996 Wang, pl.3, 6
Dictyotriletes rotundus 1987 Wang Genxian et al. et al., p. 299
Dictyotriletes trivialis Naumova in Kedo 1996 Wang, pl.3, 3
Endosporites micromanifestus Hacquebard 1996 Wang, pl.6, 19-20
Endosporites velatus Wang, 1996 1996 Wang, pl.6, 16
Grandispora echinata Hacquebard 1996 Wang, pl.4, 14-15
Grandispora gracilis (Kedo) Streel 1996 Wang, pl.4, 16-17
Granulatisporites crenulatus Playford 1996 Wang, pl.2, 12-13
Granulatisporites frustulentus (Balme et Hassell) Playford 1996 Wang, pl.2, 2-3
Hymenozonotriletes explanatus 1987 Wang Genxian et al. et al., p. 299
Hymenozonotriletes rarispinosus Lu 1996 Wang, pl.5, 8-12
Hymenozonotriletes sp. 1987 Wang Genxian et al. et al., p. 299
Ilymenozonoptriletes explanatus 1987 Wang Genxian et al. et al., p. 299
Knoxisporites literatus (Waltz) Playford 1996 Wang, pl.6, 10
Knoxisporites literatus 1987 Wang Genxian et al. et al., p. 299
Kraeuselisporites cf. *echinatus* Owens, Mishell et Marshall 1996 Wang, pl.6, 12
Laevigatosporites minor Loose 1996 Wang, pl.6, 4-5
Laevigatosporites rarispinosus 1996 Wang, p. 17
Leiotriletes cf. *prominulus* Ouyang et Chen 1996 Wang, pl.1, 4
Leiotriletes confertus McGregor 1996 Wang, pl.1, 8
Leiotriletes simplex Naumova 1996 Wang, pl.1, 5

Lophotriletes erinaceus (Waltz et Naumova) Zhang 1996 Wang, pl.1, 22-23
Lophozonotriletes excisus Naumova 1996 Wang, pl.2, 16-17
Lycospora brevis Bharadwaj 1996 Wang, pl.6, 17
Lycospora denticulata Bharadwaj 1996 Wang, pl.6, 18
Punctatisporites camaratus Ouyang et Chen 1996 Wang, pl.1, 7
Punctatisporites glaber (Naumova) Playford 1996 Wang, pl.1, 10
Punctatisporites recavus Ouyang et Chen 1996 Wang, pl.1, 6
Radiizonates camarosus Wang, 1996 1996 Wang, pl.6, 7-9
Radiizonates irregulatus Wang, 1996 1996 Wang, pl.6, 13-14
Radiizonates regulatus Wang, 1996 1996 Wang, pl.5, 15
Raistrickia variabilis Dolby et Neves 1996 Wang, pl.2, 21
Reticulatisporites cancellatus (Waltz) Playford 1996 Wang, pl.3, 1
Reticulatisporites perlotus (Naumova) Ouyang et Chen 1996 Wang, pl.3, 10
Reticulatisporites rudis Staplin 1996 Wang, pl.3, 2
Retispora lepidophyta (Kedo) Playford 1996 Wang, pl.3, 11-15
Retusisporites asthenolabratus Hou 1996 Wang, pl.1, 14-15
Retusisporites communis Naumova 1996 Wang, pl.1, 18
Retusisporites simplex Naumova 1996 Wang, pl.1, 9
Retusotriletes incohatus 1987 Wang Genxian et al. et al., p. 299
Retusotriletes incohatus 1987 Wang Genxian et al. et al., p. 299
Rugospora cf. *flexuosa* (Jusch.) Streel in B. B. S. T. 1996 Wang, pl.6, 11
Spelaeotriletes echinatus (Luber) Ouyang et Chen 1996 Wang, pl.1, 11-12
Spelaeotriletes pretiosus (Playford) Neves et Belt 1996 Wang, pl.2, 24
Spinozonotriletes saurotus Higgs, Clayton et Keegan 1996 Wang, pl.2, 22-23
Stenozonotriletes facilis var. *paraecrassus* Ischenko 1996 Wang, pl.4, 13
Tumulispora rarituberculata (Luber) Playford 1996 Wang, pl.4, 5
Tumulisporites varituberculatus 1987 Wang Genxian et al. et al., p. 299
Tumulisporites variverrucata 1987 Wang Genxian et al. et al., p. 299
Vallatisporites cf. *pusillites* (Kedo) Dolby et Neves 1996 Wang, pl.4, 4
Vallatisporites cf. *vallatus* Hacquebard 1987 Wang Genxian et al. et al., p. 299; 1996 Wang, pl.4, 3
Vallatisporites vallatus 1987 Wang Genxian et al. et al., p. 299
Vallatisporites verrucosus 1987 Wang Genxian et al. et al., p. 299
Velamisporites perinatus (Hughes et Playford) Playford 1996 Wang, pl.6, 1-2
Verrucosisporites cf. *dejerseyi* Playford 1996 Wang, pl.2, 18
Verrucosisporites nitidus 1987 Wang Genxian et al. et al., p. 299

Oujiachong Section, Lengshuijiang City: Upper Menggong'ao Formation

Acanthotriletes similis Naumova 1996 Wang, pl.2, 4-5
Aneurospora greggsii (McGregor) Streel 1996 Wang, pl.3, 16-17
Apiculiretusispora microverrucosa (Bharadwaj et al.) Lu 1996 Wang, pl.2, 6-7
Calamospora divisa Gao et Hou 1996 Wang, pl.1, 1-2
Calyptosporites minor Wang, 1996 1996 Wang, pl.4, 11
Crassispora cf. *kosankei* (Potoni é et Kremp) Smith et Butterworth 1996 Wang, pl.3, 20-21
Crassispora spinogranulata Wang, 1996 1996 Wang, pl.2, 26-28

Cristatisporites limitatus Ouyang et Chen 1996 Wang, pl.5, 18-19
Cymbosporites chinensis Ouyang et Chen 1996 Wang, pl.2, 14-15
Densosporites rarispinosus Playford 1996 Wang, pl.5, 5-7
Grandispora gracilis (Kedo) Streel 1996 Wang, pl.4, 16-17
Hymenozonotriletes rarispinosus Lu Wang, pl.5, 8-12
Leiotriletes simplex Naumova 1996 Wang, pl.1, 5
Lophotriletes erinaceus (Waltz et Naumova) Zhang 1996 Wang, pl.1, 22-23
Lophozonotriletes excisus Naumova 1996 Wang, pl.2, 16-17
Lycospora denticulata Bharadwaj 1996 Wang, pl.6, 18
Punctatisporites camaratus Ouyang et Chen 1996 Wang, pl.1, 7
Punctatisporites glaber (Naumova) Playford 1996 Wang, pl.1, 10
Raistrickia clavata (Hacquebard) Playford 1996 Wang, pl.2, 25
Rugospora cf. *flexuosa* (Jusch.) Streel in B. B. S. T. 1996 Wang, pl.6, 11
Spelaeotriletes pretiosus (Playford) Neves et Belt 1996 Wang, pl.2, 24
Tumulispora rarituberculata (Luber) Playford 1996 Wang, pl.4, 5
Vallatisporites cf. *vallatus* Hacquebard 1996 Wang, pl.4, 3
Velamisporites perinatus (Hughes et Playford) Playford 1996 Wang, pl.6, 1-2

10. Leimingqiao, Lianyuan City: Shaodong Formation
Aneurospora greggsii (McGregor) Streel in B. B. S. T., 1974 1990 Gao Lianda, pl. II, 8
Aneurospora incohatus (Sullivan) Streel in B. B. S. T., 1974 1990 Gao Lianda, pl. I, 4
Cristatisporites echinatus Playford, 1963 1990 Gao Lianda, pl. II, 2
Cymbosporites formosus (Naumova) Gao, 1983 1990 Gao Lianda, pl. II, 7
Dibolisporites sp. 1990 Gao Lianda, pl. I, 18
Lophozonotriletes sp. 1990 Gao Lianda, pl. I, 26
Vallatisporites vallatus Hacquebard, 1957 1990 Gao Lianda, pl. II, 17

Hubei

1. Liujiachanghuangkuang Section, Songzi County: Tizikou Formation
Acinosporites macrospinosus Rhichardson 1965 1992 Gao Lianda, pl. I, 24-26
Ancyrospora furcula Owens, 1971 1992 Gao Lianda, pl. I, 28
Aneurospora greggsii (McGregor) Streel in Becker et al., 1974 1992 Gao Lianda, pl. II, 18
Apiculatisporis microspinosus Gao et Zhong, 1984 1984 Feng Shaonan, pl.50, 9
Apiculiretusispora fructiosa Higgs 1984 Feng Shaonan, pl.50, 8
Apiculiretusispora nitida Owens, 1971 1992 Gao Lianda, pl. I, 11
Archaeoperisaccus scabratus Owens, 1971 1984 Feng Shaonan, pl.51, 28-2; 1992 Gao Lianda, pl. IV, 30
Archaeozonotriletes sp. 1992 Gao Lianda, pl. III, 13-14
Archaeozonotriletes variabilis (Naumova) Allen, 1965 1992 Gao Lianda, pl. III, 12
Chelinospora? sp. 1992 Gao Lianda, pl. II, 3
Clivosisporites variabilis Staplin et Jansonius 1984 Feng Shaonan, pl.51, 9
Convolutispora implicata (Kedo) Gao et Zhong, 1984 1984 Feng Shaonan, pl.50, 16
Convolutispora micatia Gao et Zhong, 1984 1984 Feng Shaonan, pl.50, 14
Convolutispora sp. 1992 Gao Lianda, pl. I, 15

Cristatisporites sp. 1992 Gao Lianda, pl. IV, 28
Cymbosporites cyathus Allen, 1965 1992 Gao Lianda, pl. II, 24
Cymbosporites echinatus (Naumova) Gao et Zhong, 1984 1984 Feng Shaonan, pl.50, 26
Cymbosporites sp. 1992 Gao Lianda, pl. II, 20-22
Cymbosporites vulgatus (Naumova) Gao et Zhong, 1984 1984 Feng Shaonan, pl.51, 2
Dictyotriletes minor Naumova 1984 Feng Shaonan, pl.50, 17
Dictyotriletes submarginatus Playford, 1964 1992 Gao Lianda, pl. II, 4
Dictyotriletes trivialis Naumova in Kedo 1984 Feng Shaonan, pl.50, 18; 1992 Gao Lianda, pl. II, 2
Discernisporites micromanifestus (Hacquebard) Sabry et Neves, 1971 1992 Gao Lianda, pl. IV, 26
Geminospora decora (Naumova) Gao et Zhong, 1984 1984 Feng Shaonan, pl.51, 1
Geminospora lemurata (Balme) Playford, 1983 1992 Gao Lianda, pl. II, 16
Geminospora nanus (Naumova) Gao, 1983 1992 Gao Lianda, pl. II, 17
Geminospora parvibasilaria (Naumova) Gao et Zhong, 1984 1984 Feng Shaonan, pl.50, 29-30; 1992 Gao Lianda, pl. II, 14
Geminospora sp. 1992 Gao Lianda, pl. II, 13
Grandispora cf. *echinata* Hacquebard, 1957 1992 Gao Lianda, pl. IV, 21
Grandispora cornuta Higgs, 19751992 Gao Lianda, pl. IV, 23-24
Grandispora delicates (Naumova) Gao, 1984 1992 Gao Lianda, pl. III, 23
Grandispora deliquescens Naumova var. *cinctus* (Chibrikova) Gao, 1992 1992 Gao Lianda, pl. III, 15
Grandispora douglastowense McGregor 1984 Feng Shaonan, pl.51, 27
Granulatisporites normalis (Naumova) Gao, 1992 1992 Gao Lianda, pl. I, 9
Granulatisporites sp. 1984 Feng Shaonan, pl.50, 10
Hymenozonotriletes? sp. 1992 Gao Lianda, pl. IV, 28
Indosporites sp. 1992 Gao Lianda, pl. IV, 25
Laevigatosporites sp. 1992 Gao Lianda, pl. IV, 31
Lophozonotriletes bellus Kedo, 1963 1992 Gao Lianda, pl. III, 22
Lophozonotriletes consimilis (Kedo) Gao, 1992 1992 Gao Lianda, pl. I, 29
Lophozonotriletes gracilis (Kedo) Gao, 1992 1992 Gao Lianda, pl. II, 7
Pustulatisporites rugulatus (Taugourdeau et Loboziak) Loboziak et Streel 1981 1992 Gao Lianda, pl. I, 29
Raistrickia cf. *variabilis* Dolby et Neves, 1970 1992 Gao Lianda, pl. I, 27
Reticulatisporites fimriatus Winlsow, 1962 1992 Gao Lianda, pl. II, 5
Retispora lepidophyta (Kedo) Playford, 1976 1984 Feng Shaonan, pl.51, 17; 1992 Gao Lianda, pl. IV, 1-3
Retispora lepidophyta (Kedo) Playford var. *minor* Kedo et Golubtova, 1971 1992 Gao Lianda, pl. IV, 6
Retispora lepidophyta (Kedo) Playford var. *tener* Kedo et Golubtova, 1971 1992 Gao Lianda, pl. IV, 4-5
Retispora sp. 1992 Gao Lianda, pl. IV, 7
Retusotriletes communis Naumova, 1970 1992 Gao Lianda, pl. I, 5
Retusotriletes planus Dolby et Neves, 1970 1992 Gao Lianda, pl. I, 4
Retusotriletes sp. 1992 Gao Lianda, pl. I, 6
Rugospora flexuosa (Juschko) Streel in Becker et al., 1974 1992 Gao Lianda, pl. II, 9
Samarisporites concinnus Owens 1984 Feng Shaonan, pl.51, 21
Samarisporites spinosus Gao et Zhong, 1984 1984 Feng Shaonan, pl.51, 19-20
Spelaeotriletes balteatus (Playford) Higgs, 1975 1992 Gao Lianda, pl. IV, 17-18
Spelaeotriletes crustatus Higgs, 1975 1992 Gao Lianda, pl. II, 25
Spelaeotriletes sp. 1992 Gao Lianda, pl. III, 1
Tumulispora macrogrumosa (Kedo) Gao et Zhong, 1984 1984 Feng Shaonan, pl.51, 11

Tumulispora malevkensis (Naumova) Turnau 1984 Feng Shaonan, pl.51, 10
Tumulispora sp. 1992 Gao Lianda, pl. III, 5
Tumulispora rarituberculata (Luber) Turnau, 1975 1992 Gao Lianda, pl. III, 3
Vallatisporites verrucosus Hacquebard, 1957 1992 Gao Lianda, pl. IV, 13
Verrucosisporites grandis McGregor 1984 Feng Shaonan, pl.50, 20
Verrucosisporites nitidus (Naumova) Playford, 1964 1992 Gao Lianda, pl. I, 13

2. Changyang: Tizikou Formation
Cymbosporites formosus (Naumova) Gao, 1983 1992 Gao Lianda, pl. II, 23
Diducites poljessicus (Kedo) Van Veen 1980 1992 Gao Lianda, pl. IV, 19
Grandispora meonacanthus (Naumova) Gao, 1983 1992 Gao Lianda, pl. IV, 20
Hymenozonotriletes explanatus (Luber) Kedo, 1963 1992 Gao Lianda, pl. IV, 15-16
Retispora sp. 1992 Gao Lianda, pl. IV, 7
Retusotriletes crassus Clayton, 1970 1992 Gao Lianda, pl. I, 3
Samarisporites concinnus Owens, 1971 1992 Gao Lianda, pl. III, 18
Samarisporites inaequus (McGregor) Owens, 1971 1992 Gao Lianda, pl. III, 17
Spelaeotriletes resolutus Higgs, 1975 1992 Gao Lianda, pl. III, 2

Yunnan

Nongba Section, Sipaishan Town and Mengshengqiao Section, Gengma County: Lower Nongba Formation
Apiculatisporis morbosus 1995 Yang Weiping et Jiajinhua, p.66
Auroraspora macra Sullivan, 1968 1995 Yang Weiping et Jiajinhua, pl.I, 7, 8
Bascaudaspora collicula (Playford) Higgs et al., 1988 1995 Yang Weiping et Jiajinhua, pl.I, 5
Grandispora clandestina 1995 Yang Weiping et Jiajinhua, p.66
Grandispora echinata 1995 Yang Weiping et Jiajinhua, p.66
Grandispora notensis 1995 Yang Weiping et Jiajinhua, p.66
Grandispora praecipua Playford, 1976 1995 Yang Weiping et Jiajinhua, pl.I, 9, 10
Hymenozonotriletes explanatus 1995 Yang Weiping et Jiajinhua, p.66
Microreticulatisporites araneum 1995 Yang Weiping et Jiajinhua, p.66
Retispora lepidophyta (Kedo) Playford, 1976 1995 Yang Weiping et Jiajinhua, pl.I, 2, 3, 6
Retusotriletes incohatus Sullivan, 1964 1995 Yang Weiping et Jiajinhua, pl.I, 1, 4
Retusotriletes triangulatus 1995 Yang Weiping et Jiajinhua, p.66

Zhejiang

Xindian Section, Fuyang City: Lower Xihu Formation
Acanthotriletes sp. 1992 Yan Tiezeng, p. 113
Acanthotriletes cf. *ignotus* Kedo, 1957 1993 He Shengce et Ouyang Shu, pl.3, 1, 5
Ancyrospora pallid Naum. 1992 Yan Tiezeng, p. 113
Aneurospora sp. 1993 He Shengce et Ouyang Shu, p.34
Apiculiretusispora granulata 1992 Yan Tiezeng, p. 113
Apiculiretusispora nitida Owens, 1971 1993 He Shengce et Ouyang Shu, pl.1, 17

Apiculiretusispora hunanensis (Hou) Ouyang et Chen, 1987 1993 He Shengce et Ouyang Shu, pl.1, 7
Auroraspora macra Sullivan, 1968 1993 He Shengce et Ouyang Shu, pl.2, 5
Auroraspora pallid Naum. 1992 Yan Tiezeng, p. 113
Auroraspora macra 1992 Yan Tiezeng, p. 113
Auroraspora cf. *poljessica* (Kedo) Streel in B. B. S. T. 1974 1993 He Shengce et Ouyang Shu, pl.4, 3-4
Auroraspora cf. *pseudocrista* Ahmed, 1980 1993 He Shengce et Ouyang Shu, pl.3, 9
Brochotriletes foveolatus 1992 Yan Tiezeng, p. 113
Calamospora unisofissus 1992 Yan Tiezeng, p. 113
Convolutispora cancellothyra 1992 Yan Tiezeng, p. 113
Cordylosporites papillatus (Naumova) Playford, 1985 1993 He Shengce et Ouyang Shu, pl.4, 5, 7-8
Cristatisporites cf. *mitratus* 1992 Yan Tiezeng, p. 113
Cyclogranisporites delicates 1992 Yan Tiezeng, p. 113
Cyclogranisporites baoyingensis 1992 Yan Tiezeng, p. 113
Cymbosporites? sp. 1993 He Shengce et Ouyang Shu, pl.2, 13
Cymbosporites promiscuous 1992 Yan Tiezeng, p. 113
Cymbosporites chinensis 1992 Yan Tiezeng, p. 113
Cymbosporites circinatus 1992 Yan Tiezeng, p. 113
Densosporites cf. *tersus* Waltz, 1993 1993 He Shengce et Ouyang Shu, pl.1, 16, 18
Densosporites? sp. 1993 He Shengce et Ouyang Shu, pl.3, 11
Densosporites xinhuanensis Hou, 1982 1993 He Shengce et Ouyang Shu, pl.2, 9-10
Dictyotriletes cf. *crassipterus* Naumova ex Kedo, 1963 1993 He Shengce et Ouyang Shu, pl.2, 4
Dictyotriletes sp. 1992 Yan Tiezeng, p. 113
Dictyotriletes sp. A 1993 He Shengce et Ouyang Shu, pl.2, 3
Dictyotriletes sp. B 1993 He Shengce et Ouyang Shu, pl.2, 8
Discernisporites micromanifestus (Hacquebard) Sabry et Neves, 1971 1993 He Shengce et Ouyang Shu, pl.4, 1
Emphanisporites sp. 1992 Yan Tiezeng, p. 113
Foveosporites cf. *pellucidus* Playford et Helby 1968 1993 He Shengce et Ouyang Shu, pl.2, 1
Grandispora sp. A 1993 He Shengce et Ouyang Shu, pl.1, 4, 11
Hymenozonotriletes sp. 1993 He Shengce et Ouyang Shu, pl.4, 2, 6, 9-12
cf. *Hymenozonotriletes explanatus* (Luber) Kedo, 1963 1993 He Shengce et Ouyang Shu, pl.3, 12
Indeterminable spora 1993 He Shengce et Ouyang Shu, pl.2, 6
Knoxisporites literatus (Waltz) Playford, 1963 1992 Yan Tiezeng, p. 113; 1993 He Shengce et Ouyang Shu, pl.1, 12, 15; pl.2, 7; pl.3, 2-3
Leiotriletes cf. *dissimilis* McGregor, 1960 1992 Yan Tiezeng, p. 113; 1993 He Shengce et Ouyang Shu, pl.2, 2
Leiotriletes sp. 1993 He Shengce et Ouyang Shu, pl.1, 13
Leiotriletes simplex 1992 Yan Tiezeng, p. 113
Lophotriletes uncatus (Naumova) Kedo, 1963 1993 He Shengce et Ouyang Shu, pl.2, 11
Lophozonotriletes rarituberculatus (Luber) Kedo, 1963 1993 He Shengce et Ouyang Shu, pl.1, 8
Lycospora tenuispinosa 1992 Yan Tiezeng, p. 113
Peritrirhiospora punctata 1992 Yan Tiezeng, p. 113
Punctatisporites sp. 1993 He Shengce et Ouyang Shu, pl.1, 3
Punctatisporites anisoletus 1992 Yan Tiezeng, p. 113
Punctatisporites jiangsuensis 1992 Yan Tiezeng, p. 113
Reticulatisporites sp. 1993 He Shengce et Ouyang Shu, pl.3, 6

Reticulatisporites poltatus 1992 Yan Tiezeng, p. 113
Reticulatisporites emsiensis 1992 Yan Tiezeng, p. 113
Retispora lepidophyta (Kedo) Playford, 1976 1993 He Shengce et Ouyang Shu, pl.1, 5
Retusotriletes sp. 1993 He Shengce et Ouyang Shu, pl.1, 1
Retusotriletes? sp. 1993 He Shengce et Ouyang Shu, pl.1, 6
Rhabdosporites porvulus 1992 Yan Tiezeng, p. 113
Samarisporites sp. 1992 Yan Tiezeng, p. 113
cf. *Simozonotriletes duploides* Ouyang et Chen, 1987 1993 He Shengce et Ouyang Shu, pl.1, 2
Stenozonotriletes rasilis 1992 Yan Tiezeng, p. 113
Stenozonotriletes conformis 1992 Yan Tiezeng, p. 113
Stenozonotriletes pumilus Waltz 1992 Yan Tiezeng, p. 113
Stenozonotriletes cf. *extensis* 1992 Yan Tiezeng, p. 113
Trihiospora subracemis 1992 Yan Tiezeng, p. 113
Verruciretusisporites magnifioa (MGG) var. *minor* 1992 Yan Tiezeng, p. 113

Jiangxi

Xiaomu Section, Chengxiang Town, Quannan County: Fanxia Formation
Acanthotriletes denticulatus Naumova 1993 Wen Zicai et Lu Lichang, pl.1, 20-22
Acanthotriletes retispinus (Luber) Ischenko 1993 Wen Zicai et Lu Lichang, pl.1, 23
Aneurospora chinensis (Ouyang et Chen) Wen et Lu, 1993 1993 Wen Zicai et Lu Lichang, pl.2, 23-24
Aneurospora greggsii (McGregor) Streel in Becker, Bless, Streel et Threz 1993 Wen Zicai et Lu Lichang, pl.2, 21-22
Aneurospora spinulifer Wen et Lu, 1993 1993 Wen Zicai et Lu Lichang, pl.3, 1-5
Apiculiretusispora gannanensis Wen et Lu, 1993 1993 Wen Zicai et Lu Lichang, pl.2, 1-6
Apiculiretusispora rarissima Wen et Lu, 1993 1993 Wen Zicai et Lu Lichang, pl.2, 7-10
Asperispora acuta (Kedo) Van der Zwan 1993 Wen Zicai et Lu Lichang, pl.2, 26-29
Auroraspora macra Sullivan 1993 Wen Zicai et Lu Lichang, pl.3, 15-16
Auroraspora poljessica (Kedo) Streel 1993 Wen Zicai et Lu Lichang, pl.3, 17
Bascaudaspora sp.1 1993 Wen Zicai et Lu Lichang, pl.3, 6
Camptozonotriletes cf. *vermiculatus* Staplin 1993 Wen Zicai et Lu Lichang, pl.3, 28
Cingulizonates bialatus (Waltz) Smith et Butterworth 1993 Wen Zicai et Lu Lichang, pl.4, 16-17
Cyclogranisporites cf. *minutus* Bharadwaj 1993 Wen Zicai et Lu Lichang, pl.1, 27
Cymbosporites circinatus Ouyang et Chen 1993 Wen Zicai et Lu Lichang, pl.3, 13-14
Densosporites rarispinosus Playford 1993 Wen Zicai et Lu Lichang, pl.4, 23
Densosporites variabilis (Waltz) Pot. et Kr. 1993 Wen Zicai et Lu Lichang, pl.4, 7-8
Densosporites? sp. cf. *Archaeozonotriletes consimilis* Kedo 1993 Wen Zicai et Lu Lichang, pl.4, 21-22
Dictyotriletes? *rotundatus* Naumova 1993 Wen Zicai et Lu Lichang, pl.2, 17
Discernisporites micromanifestus (Hacq.) Sabry et Neves 1993 Wen Zicai et Lu Lichang, pl.4, 18-19
Emphanisporites sp. 1993 Wen Zicai et Lu Lichang, pl.2, 20
Foveosporites sp. 1993 Wen Zicai et Lu Lichang, pl.2, 18
Grandispora conspicua (Playford) Playford 1993 Wen Zicai et Lu Lichang, pl.4, 20
Grandispora echinata Hacquebard 1993 Wen Zicai et Lu Lichang, pl.3, 19
Grandispora gracilis (Kedo) Streel 1993 Wen Zicai et Lu Lichang, pl.4, 1

Grandispora xiaomuensis Wen et Lu, 1993 1993 Wen Zicai et Lu Lichang, pl.3, 21-24
Granulatisporites minimus Wen et Lu, 1993 1993 Wen Zicai et Lu Lichang, pl.1, 28-29
Gulisporites torpidus Playford 1993 Wen Zicai et Lu Lichang, pl.1, 1
Laevigatosporites vulgaris (Ibr.) Alpern et Doubinger 1993 Wen Zicai et Lu Lichang, pl.4, 24
Leiotriletes laevis Naumova 1993 Wen Zicai et Lu Lichang, pl.1, 4-5
Leiotriletes macrothelis Wen et Lu, 1993 1993 Wen Zicai et Lu Lichang, pl.1, 6-9
Leiotriletes microrugosus (Ibr.) Naumova 1993 Wen Zicai et Lu Lichang, pl.1, 2-3
Leiotriletes microthelis Wen et Lu, 1993 1993 Wen Zicai et Lu Lichang, pl.1, 10-12
Leiotriletes simplex Naumova 1993 Wen Zicai et Lu Lichang, pl.1, 15
Leiotriletes sp. 1993 Wen Zicai et Lu Lichang, pl.1, 13
Microreticulatisporites sp. 1993 Wen Zicai et Lu Lichang, pl.2, 19
Punctatisporites cf. *minutus* Kosankei 1993 Wen Zicai et Lu Lichang, pl.1, 26
Punctatisporites jiangsuensis Ouyang et Chen 1993 Wen Zicai et Lu Lichang, pl.1, 14
Retusotriletes asthenolabratus Hou 1993 Wen Zicai et Lu Lichang, pl.1, 16-17
Retusotriletes minor Kedo 1993 Wen Zicai et Lu Lichang, pl.1, 19
Retusotriletes simplex Naumova 1993 Wen Zicai et Lu Lichang, pl.1, 24-25
Spelaeotriletes vulgaris Wen et Lu, 1993 1993 Wen Zicai et Lu Lichang, p.309
Tumulispora cf. *variverrucata* (Playford) Staplin et Jansonius 1993 Wen Zicai et Lu Lichang, pl.3, 25
Tumulispora malvekensis (Kedo) Turnau 1993 Wen Zicai et Lu Lichang, pl.3, 27
Tumulispora zhushanensis (Hou) Wen et Lu, 1993 1993 Wen Zicai et Lu Lichang, pl.3, 26

Jiangsu

1. Cishan, Nanjing City: Leigutai Member of Wutung Formation

Aneurospora greggsii 1987 Li Hanmin et al., p. 117-118
Aneurospora incohatus 1987 Li Hanmin et al., p. 117-118
Archaeoponotriletes sp. 1987 Li Hanmin et al., p. 117-118
Cymbosporites formosus 1987 Li Hanmin et al., p. 117-118
Discernisporites micromanifestus 1987 Li Hanmin et al., p. 117-118
Grandispora sp. 1987 Li Hanmin et al., p. 117-118
Hymenosonotriletes explanatus 1987 Li Hanmin et al., p. 117-118
Knoxisporites literatus 1987 Li Hanmin et al., p. 117-118
Retispora lepidophyta 1987 Li Hanmin et al., p. 117-118
Retispora lepidophyta var. *tener* 1987 Li Hanmin et al., p. 117-118
Vallatisporites pusillites 1987 Li Hanmin et al., p. 117-118
Vallatisporites vallatus 1987 Li Hanmin et al., p. 117-118
Vallatisporites verrucosus 1987 Li Hanmin et al., p. 117-118

2. Baojia Village, Jurong County: Lower Leigutai Member

Acanthotriletes edurus Ouyang et Chen, 1987 1987a Ouyang Shu et Chen Yongxiang, pl.16, 14
Acanthotriletes liratus Ouyang et Chen, 1987 1987a Ouyang Shu et Chen Yongxiang, pl.5, 21
Acanthotriletes mirus var. *trigonalis* Isch., 1958 1987a Ouyang Shu et Chen Yongxiang, pl.5, 4-9
Acanthotriletes rarus Ouyang et Chen, 1987 1987a Ouyang Shu et Chen Yongxiang, pl.5, 3
Acanthotriletes stiphros Ouyang et Chen, 1987 1987a Ouyang Shu et Chen Yongxiang, pl.5, 1-2

Acanthotriletes sunanensis Ouyang et Chen, 1987 1987a Ouyang Shu et Chen Yongxiang, pl.6, 1
Anapiculatisporites famenensis (Naumova) Ouyang et Chen, 1987 1987a Ouyang Shu et Chen Yongxiang, pl.5, 19
Anapiculatisporites hystricosus Playford, 1963 1987a Ouyang Shu et Chen Yongxiang, pl.9, 6-9
Anapiculatisporites mucronata Ouyang et Chen, 1987 1987a Ouyang Shu et Chen Yongxiang, pl.9, 1-5
Apiculiretusispora conflecta Ouyang et Chen, 1987 1987a Ouyang Shu et Chen Yongxiang, pl.8, 5-6
Apiculiretusispora granulata Owens, 1971 1987a Ouyang Shu et Chen Yongxiang, pl.8, 3-4
Apiculiretusispora hunanensis (Hou) Ouyang et Chen, 1987 1987a Ouyang Shu et Chen Yongxiang, pl.8, 15-19
Apiculiretusispora nitida Owens, 1971 1987a Ouyang Shu et Chen Yongxiang, pl.8, 1
Archaeozonotriletes cf. *variabilis* Naumova 1987a Ouyang Shu et Chen Yongxiang, pl.15, 40
Auroraspora cf. *hyaline* (Naumova) Streel in Becker et al., 1974 1987a Ouyang Shu et Chen Yongxiang, pl.13, 8
Biornatispora compactilis Ouyang et Chen, 1987 1987a Ouyang Shu et Chen Yongxiang, pl.5, 12-13
Calamospora cf. *pedata* Kosanke, 1950 1985 Chen Yongxiang et Ouyangshu, p. 268; 1987a Ouyang Shu et Chen Yongxiang, pl.2, 22, 24
Calamospora parva Guennel, 1958 1987a Ouyang Shu et Chen Yongxiang, pl.2, 14-15
Calamospora unisofissus Ouyang et Chen, 1987 1987a Ouyang Shu et Chen Yongxiang, pl.2, 21, 23
Chomotriletes rarivittatus Ouyang et Chen, 1987 1987a Ouyang Shu et Chen Yongxiang, pl.18, 13-15
Colatisporites expansus Ouyang et Chen, 1987 1987a Ouyang Shu et Chen Yongxiang, pl.17, 13
Colatisporites reticuloides Ouyang et Chen, 1987 1987a Ouyang Shu et Chen Yongxiang, pl.12, 5; pl.16, 15; pl.17, 4, 6, 9-11
Colatisporites spiculifer Ouyang et Chen, 1987 1987a Ouyang Shu et Chen Yongxiang, pl.17, 1-3
Convolutispora composita Ouyang et Chen, 1987 1987a Ouyang Shu et Chen Yongxiang, pl.6, 6-7
Cristatisporites limitatus Ouyang et Chen, 1987 1987a Ouyang Shu et Chen Yongxiang, pl.16, 10-11
Cyclogranisporites areolatus Ouyang et Chen, 1987 1987a Ouyang Shu et Chen Yongxiang, pl.4, 6-7
Cyclogranisporites cf. *aureus* (Loose) Potonié et Kremp, 1955 1987a Ouyang Shu et Chen Yongxiang, pl.3, 27
Cyclogranisporites commodus Playford, 1964 1985 Chen Yongxiang et Ouyangshu, p. 268; 1987a Ouyang Shu et Chen Yongxiang, pl.4, 9
Cyclogranisporites microgranus Bharadwaj, 1957 1987a Ouyang Shu et Chen Yongxiang, pl.3, 19, 22, 23, 24
Cymbosporites chinensis Ouyang et Chen, 1987 1987a Ouyang Shu et Chen Yongxiang, pl.15, 20-24
Cymbosporites circinatus Ouyang et Chen, 1987 1987a Ouyang Shu et Chen Yongxiang, pl.15, 25-28
Cymbosporites cordylatus Ouyang et Chen, 1987 1987a Ouyang Shu et Chen Yongxiang, pl.15, 12-15
Cymbosporites densus Ouyang et Chen, 1987 1987a Ouyang Shu et Chen Yongxiang, pl.15, 16-19
Cymbosporites dimerus Ouyang et Chen, 1987 1987a Ouyang Shu et Chen Yongxiang, pl.15, 29-33
Cymbosporites minutus Ouyang et Chen, 1987 1987a Ouyang Shu et Chen Yongxiang, pl.16, 12-13
Cymbosporites promiscuus Ouyang et Chen, 1987 1987a Ouyang Shu et Chen Yongxiang, pl.15, 5-7
Dibolisporites coalitus Ouyang et Chen, 1987 1987a Ouyang Shu et Chen Yongxiang, pl.9, 10-11
Dibolisporites mucronatus Ouyang et Chen, 1987 1987a Ouyang Shu et Chen Yongxiang, pl.8, 8-14
Dibolisporites orientalis Ouyang et Chen, 1987 1987a Ouyang Shu et Chen Yongxiang, pl.8, 7
Dibolisporites spiculatus Ouyang et Chen, 1987 1987a Ouyang Shu et Chen Yongxiang, pl.7, 1-5
Dibolisporites uncinulus Ouyang et Chen, 1987 1987a Ouyang Shu et Chen Yongxiang, pl.7, 9
Dibolisporites upensis Jush. in Kedo 1987 1987a Ouyang Shu et Chen Yongxiang, pl.7, 10-12
Endosporites elegans Ouyang et Chen, 1987 1987a Ouyang Shu et Chen Yongxiang, pl.17, 5
Grandispora apicilaris Ouyang et Chen, 1987 1987a Ouyang Shu et Chen Yongxiang, pl.16, 5-9
Grandispora cf. *echinata* Hacq., 1957 1987a Ouyang Shu et Chen Yongxiang, pl.13, 1-3; pl.15, 38
Grandispora gracilis (Kedo) Streel, 1974 1987a Ouyang Shu et Chen Yongxiang, pl.13, 4, 5, 7, 10-12
Grandispora wutongiana Ouyang et Chen, 1987 1987a Ouyang Shu et Chen Yongxiang, pl.5, 24; pl.7, 13-14

Granulatisporites cf. *rudigranulatus* Staplin, 1960 1987a Ouyang Shu et Chen Yongxiang, pl.3, 7
Granulatisporites crassus Ouyang et Chen, 1987 1987a Ouyang Shu et Chen Yongxiang, pl.3, 8
Hymenozonotriletes caveatus Ouyang et Chen, 1987 1987a Ouyang Shu et Chen Yongxiang, pl.15, 39
Hystricosporites sp. 1987a Ouyang Shu et Chen Yongxiang, pl.7, 7-8
Leiotriletes cf. *dissimilis* McGregor, 1960 1987a Ouyang Shu et Chen Yongxiang, pl.1, 31-32
Leiotriletes cf. *subintortus* (Waltz) Ischenko var. *rotundatus* Waltz, 1941 1987a Ouyang Shu et Chen Yongxiang, pl.1, 28-30
Leiotriletes involuta Ouyang et Chen, 1987 1987a Ouyang Shu et Chen Yongxiang, pl.1, 24-27
Leiotriletes labiatus Ouyang et Chen, 1987 1987a Ouyang Shu et Chen Yongxiang, pl.1, 3-6, 9
Leiotriletes laevis Naumova, 1953 1985 Chen Yongxiang et Ouyangshu, p. 268; 1987a Ouyang Shu et Chen Yongxiang, pl.1, 1, 2
Leiotriletes scabratus Ouyang et Chen, 1987 1987a Ouyang Shu et Chen Yongxiang, pl.3, 11-13
Leiotriletes simplex Naumova, 19531985 Chen Yongxiang et Ouyangshu, p. 268; 1987a Ouyang Shu et Chen Yongxiang, pl.1, 11, 12
Leiotriletes trivialis Naumova, 1953 1985 Chen Yongxiang et Ouyangshu, p. 268; 1987a Ouyang Shu et Chen Yongxiang, pl.1, 7
Leiozonotriletes leigutaiensis Ouyang et Chen, 1987 1987a Ouyang Shu et Chen Yongxiang, pl.12, 13
Lophozonotriletes cf. *curvatus* Naumova, 1953 1987a Ouyang Shu et Chen Yongxiang, pl.5, 23
Microreticulatisporites cf. *verus* Potonié et Kremp, 1955 1987a Ouyang Shu et Chen Yongxiang, pl.3, 26
Monilospora limbata Ouyang et Chen, 1987 1987a Ouyang Shu et Chen Yongxiang, pl.15, 42
Peritrirhiospora laevigata Ouyang et Chen, 1987 1987a Ouyang Shu et Chen Yongxiang, pl.10, 8-10
Peritrirhiospora magna Ouyang et Chen, 1987 1987a Ouyang Shu et Chen Yongxiang, pl.10, 13; pl.11, 1-3
Peritrirhiospora punctata Ouyang et Chen, 1987 1987a Ouyang Shu et Chen Yongxiang, pl.10, 4-7
Pulvinispora? *spinulosa* Ouyang et Chen, 1987 1987a Ouyang Shu et Chen Yongxiang, pl.4, 2, 11
Punctatisporites anisoletus Ouyang et Chen, 1987 1987a Ouyang Shu et Chen Yongxiang, pl.2, 6-8; pl.4, 1
Punctatisporites camaratus Ouyang et Chen, 1987 1987a Ouyang Shu et Chen Yongxiang, pl.1, 13-14; pl.2, 4
Punctatisporites jiangsuensis Ouyang et Chen, 1987 1987a Ouyang Shu et Chen Yongxiang, pl.1, 17-19
Punctatisporites recavus Ouyang et Chen, 1987 1987a Ouyang Shu et Chen Yongxiang, pl.1, 15-16
Punctatisporites rotundatus (Naum.) 1985 Chen Yongxiang et Ouyangshu, p. 268
Punctatisporites rotundus (Naumova) Ouyang et Chen, 1987 1987a Ouyang Shu et Chen Yongxiang, pl.2, 1-3
Raistrickia nigra Love 1960 1987a Ouyang Shu et Chen Yongxiang, pl.4, 12-13
Reticulatisporites cf. *mediareticulatus* (Ibr.) Potonié et Kremp, 1955 1987a Ouyang Shu et Chen Yongxiang, pl.6, 16-17
Reticulatisporites perlotus (Naumova) Ouyang et Chen, 1987 1987a Ouyang Shu et Chen Yongxiang, pl.6, 14
Retispora lepidophyta (Kedo) Playford var. *minor* Kedo et Golub., 1971 1987a Ouyang Shu et Chen Yongxiang, pl.13, 9, 13
Retusotriletes ? *mirificus* Ouyang et Chen, 1987 1987a Ouyang Shu et Chen Yongxiang, pl.3, 9
Retusotriletes communis Naumova, 1953 1985 Chen Yongxiang et Ouyangshu, p. 268; 1987a Ouyang Shu et Chen Yongxiang, pl.3, 4-6
Retusotriletes pychovii var. *major* Naumova, 1953 1987a Ouyang Shu et Chen Yongxiang, pl.3, 10
Retusotriletes rotundus (Streel) Streel, 1967 1987a Ouyang Shu et Chen Yongxiang, pl.3, 1-3
Retusotriletes triangulatus (Streel) Streel, 1967 1985 Chen Yongxiang et Ouyangshu, p. 268; 1987a Ouyang Shu et Chen Yongxiang, pl.2, 25-26
Samarisporites microspinosus Ouyang et Chen, 1987 1987a Ouyang Shu et Chen Yongxiang, pl.13, 21
Samarisporites spiculatus Ouyang et Chen, 1987 1987a Ouyang Shu et Chen Yongxiang, pl.15, 41
Simozonotriletes duploides Ouyang et Chen, 1987 1987a Ouyang Shu et Chen Yongxiang, pl.14, 3-6
Stenozonotriletes cf. *extensus* Naumova, 1953 1987a Ouyang Shu et Chen Yongxiang, pl.15, 35-37
Stenozonotriletes cf. *simplex* Naumova, 1953 1987a Ouyang Shu et Chen Yongxiang, pl.13, 20; pl.15, 34

Stenozonotriletes conformis Naumova, 1953 1987a Ouyang Shu et Chen Yongxiang, pl.13, 18-19
Stenozonotriletes pumilus (Waltz) Naumova, 1953 1985 Chen Yongxiang et Ouyangshu, p. 268; 1987a Ouyang Shu et Chen Yongxiang, pl.14, 14-18
Stenozonotriletes solidus Ouyang et Chen, 1987 1987a Ouyang Shu et Chen Yongxiang, pl.14, 10-11
Sublagenicula acifera Chen et Ou, 1987 1987 Chen Yongxiang et Ouyang Shu, pl.2, 3, 3a, 4, 4a, 5; pl.4, 12, 12a
Sublagenicula jiangsuensis Chen et Ou, 1987 1987 Chen Yongxiang et Ouyang Shu, pl.4, 11, 11a
Sublagenicula reticuloides Chen et Ou, 1987 1987 Chen Yongxiang et Ouyang Shu, pl.1, 6, 6a; pl.2, 1, 2, 2a; pl.4, 13
Synorisporites minor Ouyang et Chen, 1987 1987a Ouyang Shu et Chen Yongxiang, pl.4, 20-21
Synorisporites varius Ouyang et Chen, 1987 1987a Ouyang Shu et Chen Yongxiang, pl.4, 14-19
Trimontisporites flexuosus Ouyang et Chen, 1987 1987a Ouyang Shu et Chen Yongxiang, pl.2, 5, 9
Trimontisporites minor Ouyang et Chen, 1987 1987a Ouyang Shu et Chen Yongxiang, pl.1, 8
Trirhiospora furva Ouyang et Chen, 1987 1987a Ouyang Shu et Chen Yongxiang, pl.9, 19; pl.10, 11
Trirhiospora plicata Ouyang et Chen, 1987 1987a Ouyang Shu et Chen Yongxiang, pl.9, 20-21
Trirhiospora strigata Ouyang et Chen, 1987 1987a Ouyang Shu et Chen Yongxiang, pl.9, 22
Trirhiospora subracemis Ouyang et Chen, 1987 1987a Ouyang Shu et Chen Yongxiang, pl.10, 1-3
Velamisporites simplex Ouyang et Chen, 1987 1987a Ouyang Shu et Chen Yongxiang, pl.13, 23
Velamisporites vincinus Ouyang et Chen, 1987 1987a Ouyang Shu et Chen Yongxiang, pl.12, 10
Velamisporites? *segregus* Ouyang et Chen, 1987 1987a Ouyang Shu et Chen Yongxiang, pl.12, 6, 11
Verruciretusispora magnifica (McGregor) var. *magnifica* Owens, 1971 1987a Ouyang Shu et Chen Yongxiang, pl.8, 2

3. Chengjiao Section, Baoying County, Yangzhou City: Lower-Middle Leigutai Member

Acanthotriletes cf. *liratus* 1987b Ouyang Shu and Chen Yongxiang, p. 199-200
Acanthotriletes simplex 1987b Ouyang Shu and Chen Yongxiang, p. 199-200
Acanthotriletes? *subulatus* 1987b Ouyang Shu and Chen Yongxiang, p. 199-200
Aneurospora cf. *semizonalis* 1987b Ouyang Shu and Chen Yongxiang, p. 199-200
Aneurospora greggsii 1987b Ouyang Shu and Chen Yongxiang, p. 199-200
Aneurospora jiangsuensis 1987b Ouyang Shu and Chen Yongxiang, p. 199-200
Apiculiretusispora acuta 1987b Ouyang Shu and Chen Yongxiang, p. 199-200
Apiculiretusispora hunanensis 1987b Ouyang Shu and Chen Yongxiang, p. 199-200
Auroraspora? *macra* 1987b Ouyang Shu and Chen Yongxiang, p. 199-200
Crassispora hystricosa 1987b Ouyang Shu and Chen Yongxiang, p. 199-200
Cristatisporites mitratus 1987b Ouyang Shu and Chen Yongxiang, p. 199-200
Cyclogranisporites baoyingensis 1987b Ouyang Shu and Chen Yongxiang, p. 199-200
Cymbosporites minutus 1987b Ouyang Shu and Chen Yongxiang, p. 199-200
Densosporites penitus 1987b Ouyang Shu and Chen Yongxiang, p. 199-200
Dibolisporites microspicatus 1987b Ouyang Shu and Chen Yongxiang, p. 199-200
Dibolisporites sp. 1987b Ouyang Shu and Chen Yongxiang, p. 199-200
Dibolisporites upensis 1987b Ouyang Shu and Chen Yongxiang, p. 199-200
Emphanisporites euryzonatus 1987b Ouyang Shu and Chen Yongxiang, p. 199-200
Endosporites parvus 1987b Ouyang Shu and Chen Yongxiang, p. 199-200
Grandispora apicilaris 1987b Ouyang Shu and Chen Yongxiang, p. 199-200
Grandispora cf. *echinata* 1987b Ouyang Shu and Chen Yongxiang, p. 199-200
Knoxisporites literatus 1987b Ouyang Shu and Chen Yongxiang, p. 199-200
Lophotriletes uncatus 1987b Ouyang Shu and Chen Yongxiang, p. 199-200

Lycospora cf. *pusilla* 1987b Ouyang Shu and Chen Yongxiang, p. 199-200
Reticulatisporites dedaleus 1987b Ouyang Shu and Chen Yongxiang, p. 199-200
Reticulatisporites serratus 1987b Ouyang Shu and Chen Yongxiang, p. 199-200
Retispora lepidophyta 1987b Ouyang Shu and Chen Yongxiang, p. 199-200
Retispora lepidophyta var. *minor* 1987b Ouyang Shu and Chen Yongxiang, p. 199-200
Stenozonotriletes rasilis 1987b Ouyang Shu and Chen Yongxiang, p. 199-200
Vallatisporites cf. *pusillites* 1987b Ouyang Shu and Chen Yongxiang, p. 199-200
Verruciretusispora semilucensis 1987b Ouyang Shu and Chen Yongxiang, p. 199-200

4. Longtan Section, Nanjing City: Guanshan Member and Lower-Middle Leigutai Member

Acanthotriletes denticulatus Naumova, 1994 Lu Lichang, pl.1, 37, 38
Aneurospora asthenolabrata Hou 1994 1994 Lu Lichang, pl.5, 34-39
Apiculiretusispora conica Lu et Ouyang 1994 Lu Lichang, pl.2, 8-9
Apiculiretusispora flexuosa Hou 1994 Lu Lichang, pl.2, 11-12
Apiculiretusispora gannanensis Wen et Lu, 1994 Lu Lichang, pl.2, 1-3
Apiculiretusispora granulata Owens 1994 Lu Lichang, pl.2, 10
Asperispora acuta (Kedo) Van der Zwan 1994 Lu Lichang, pl.5, 5-7
Auroraspora macra Sullivan 1994 Lu Lichang, pl.3, 21
Baculatisporites sp. 1988 Cai Chongyang et al., p. 173
Camptosporites sp. 1994 Lu Lichang, pl.2, 14
Converrucosisporites parvinodosus Playford 1994 Lu Lichang, pl.3, 8-9
Crassispora parva 1988 Cai Chongyang et al., p. 173
Cristatisporites connexus Pot. et Kr. 1994 Lu Lichang, pl.4, 14
Crossispora spitzbergense 1988 Cai Chongyang et al., p. 173
Cyclogranisporites baoyingensis Ouyang et Chen 1994 Lu Lichang, pl.1, 39, 40
Cymbosporites conatus Bharadwaj et al. 1994 Lu Lichang, pl.5, 8-9
Cymbosporites famensis (Naumova) Lu, 1994 1994 Lu Lichang, pl.5, 17-18
Cymbosporites magnificus var. *magnificus* (Owens) Lu, 1994 Lu Lichang, pl.5, 19
Cymbosporites microverrucosus Bharadwaj et al. 1994 Lu Lichang, pl.4, 7
Cymbosporites minutus Ouyang et Chen 1994 Lu Lichang, pl.5, 1-2
Cymbosporites septalis (Jush.) var. *minor* (Kedo) Lu, 1994 1994 Lu Lichang, pl.5, 24-25
Cymbosporites zonalis 1994 1994 Lu Lichang, pl.3, 31-34
Dictyotriletes rotundatus 1988 Cai Chongyang et al., p. 173
Dictyotriletes submarginatus 1988 Cai Chongyang et al., p. 173
Discernisporites micromanifestus (Hacquebard) Sabry et Neves 1994 Lu Lichang, pl.6, 9
Gorgonispora convoluta 1988 Cai Chongyang et al., p. 173
Grandispora echinata Hacquebard 1994 Lu Lichang, pl.5, 27
Grandispora upensis (Kedo) Byvscheva 1994 Lu Lichang, pl.5, 28
Granulatisporites rugosus var. *minor* Naumova, 1994 1994 Lu Lichang, pl.4, 1-2
Granulatisporites sp. 1988 Cai Chongyang et al., p. 173
Knoxisporites literatus (Waltz) Playford 1994 Lu Lichang, pl.4, 24-25
Leiotriletes labiatus Ouyang et Chen 1994 Lu Lichang, pl.1, 2
Leiotriletes laevis Naumova, 1994 Lu Lichang, pl.1, 3
Leiotriletes ornatus Ishchenko 1994 Lu Lichang, pl.1, 35

Lophotriletes minor Naumova, 1994 Lu Lichang, pl.3, 29-30
Lophotriletes perpusillus Naumova, 1994 Lu Lichang, pl.3, 7
Lycospora tenuispinosa Ouyang et Chen 1994 Lu Lichang, pl.4, 5-6
Punctatisporites debilis Hacquebard 1994 Lu Lichang, pl.1, 8
Punctatisporites involutus 1994 1994 Lu Lichang, pl.1, 4, 5, 23
Punctatisporites planus Hacquebard 1994 Lu Lichang, pl.1, 9, 10
Punctatisporites rotundus (Naumova) Ouyang et Chen 1994 Lu Lichang, pl.1, 11
Radiizonates longtanensis Lu, 1994 1994 Lu Lichang, pl.5, 10-16
Reticulatisporites sp. 1988 Cai Chongyang et al., p. 173
Retispora? cf. *lepidophyta* (Kedo) Playford 1994 Lu Lichang, pl.6, 10-12
Retusotriletes crassus Clayton in Clayton et al. 1994 Lu Lichang, pl.1, 17
Retusotriletes minor Kedo 1994 Lu Lichang, pl.1, 36
Retusotriletes rotundus (Streel) Streel 1994 Lu Lichang, pl.1, 19
Retusotriletes simplex Naumova, 1994 Lu Lichang, pl.1,1, 21
Retusotriletes triangulates 1988 Cai Chongyang et al., p. 173
Rugospora flexuosa 1988 Cai Chongyang et al., p. 173
Simozonotriletes conduplicatus (Andrejeva) Ishchenko 1994 Lu Lichang, pl.5, 26
Spelaeotriletes cf. *minutus* Butterworth et Mahdi 1994 Lu Lichang, pl.3, 1-2
Spelaeotriletes crenulatus (Playford) Higgs et al. 1994 Lu Lichang, pl.3, 11-12
Spelaeotriletes crustatus Higgs 1994 Lu Lichang, pl.6, 18
Spelaeotriletes granulates Lu, 1994 1994 Lu Lichang, pl.6, 13-15
Spelaeotriletes inaequiformis Lu, 1994 1994 Lu Lichang, pl.6, 33-35
Spelaeotriletes resolutus Higgs 1994 Lu Lichang, pl.6, 38-39
Spelaeotriletes setosus (Kedo) Lu, 1994 1994 Lu Lichang, pl.6, 28-29
Spelaeotriletes subulatus (Ouyang et Chen) Lu, 1994 1994 Lu Lichang, pl.6, 25-27
Stenozonotriletes solidus Ouyang et Chen 1994 Lu Lichang, pl.1, 24, 25
Synorisporites minor Ouyang et Chen 1994 Lu Lichang, pl.5, 3-4
Triquitrites leiotritus Bharadwaj 1994 Lu Lichang, pl.1, 13, 14
Vallatisporites cf. *vallatus* Hacquebard 1994 Lu Lichang, pl.6, 16-17
Velamisporites lacertosus Playford 1994 Lu Lichang, pl.6, 40
Velamisporites laevigatus (Lu) Lu, 1994 1994 Lu Lichang, pl.6, 5-6
Velamisporites perinatus (Heghes et Playford) Playford 1994 Lu Lichang, pl.6, 7-8
Velamisporites sp. 1994 Lu Lichang, pl.3, 22

Guanshan Section, Longtan Town, Nanjing City: Guanshan Member of Wutung Formation

Cymbosporites conatus 1988 Cai Chongyang et al., p. 174
Discernisporites micromanifestus 1988 Cai Chongyang et al., p. 174
Spelaeotriletes exiguous 1988 Cai Chongyang et al., p. 174
Vallatisporites vallatus 1988 Cai Chongyang et al., p. 174

5. Kongshan, Nanjing City: Wutung Formation

Archaeozonotriletes variabilis 1988 Cai Chongyang et al., p. 178
Calamospora sp. 1988 Cai Chongyang et al., p. 178
Dictyotriletes sp. 1988 Cai Chongyang et al., p. 178

Grandispora echinata 1988 Cai Chongyang et al., p. 178
Knoxisporites literatus 1988 Cai Chongyang et al., p. 178
Retispora lepidophyta 1988 Cai Chongyang et al., p. 178
Retispora lepidophyta var. *minor* 1988 Cai Chongyang et al., p. 178
Retusotriletes triangulates 1988 Cai Chongyang et al., p. 178
Stenozonotriletes sp. 1988 Chongyang et al., p. 178
Vallatisporites cf. *pusillites* 1988 Cai Chongyang et al., p. 178

6. Nanshan, Dingshu Town, Yixing City: Wutung Formation
cf. *Archaeozonotriletes acutus* 1988 Cai Chongyang et al., p. 182
Acanthotriletes simplex 1988 Cai Chongyang et al., p. 181
Crassispora parva 1988 Cai Chongyang et al., p. 181
Grandispora echinata 1988 Cai Chongyang et al., p. 182
Lophotriletes minutissimus 1988 Cai Chongyang et al., p. 182
Punctatisporites irrasus 1988 Cai Chongyang et al., p. 181
Spelaeotriletes cf. *minutus* 1988 Cai Chongyang et al., p. 181

Tibet
Yalicun Formation, Nyalam County: Upper Boqu Group
Acanthotriletes spinellosus (Naumova, 1953) Gao, 1983 1983a Gao Lianda, pl. II, 1
Acinosporites acanthomammillatus Richardson, 1965 1983a Gao Lianda, pl. II, 4
Acinosporites macrospinosus Richardson, 1965 1983a Gao Lianda, pl. II, 17
Anapiculatisporites sp. 1983a Gao Lianda, pl. IV, 3-4
Ancyrospora sp. 1983a Gao Lianda, pl. II, 19
Aneurospora asperella (Kedo) Van Der Zwen, 1980 1983a Gao Lianda, pl. III, 12-13
Aneurospora (Retusotriletes) incohatus (Sullivan) Streel in Becker et al., 1974 1983a Gao Lianda, pl. III, 11
Aneurospora greggsii (McGregor) Streel in Becker et al., 1974 1983a Gao Lianda, pl. III, 8-9
Apiculatisporis sp. 1983a Gao Lianda, pl. II, 3, 18
Apiculatisporis xizangensis Gao, 1983 1983a Gao Lianda, pl. I, 21-23
Apiculiretusispora granulata Owens, 1971 1983a Gao Lianda, pl. I, 11
Apiculiretusispora kurta Gao, 1983 1983a Gao Lianda, pl. I, 12-13
Apiculiretusispora nitida Owens, 1971 1983a Gao Lianda, pl. I, 14-15
Apiculiretusispora plicata (Allen) Streel, 1967 1983a Gao Lianda, pl. I, 18
Apiculiretusispora septalata (Kedo) Gao, 1983 1983a Gao Lianda, pl. I, 16
Archaeoperisaccus sp. 1983a Gao Lianda, pl. VIII, 3
Archaeozonotriletes antiquus Naumova, 1953 1983a Gao Lianda, pl. IV, 7-8
Archaeozonotriletes dilatatus Gao, 1983 1983a Gao Lianda, pl. IV, 11
Archaeozonotriletes variabilis (Naumova) Allen, 1965 1983a Gao Lianda, pl. IV, 9-10
Asperispora acuta (Kedo) Van Der Zwan, 1980 1983a Gao Lianda, pl. IV, 13
Auroraspora macra Sullivan, 1964 1983a Gao Lianda, pl. VII, 19, 21
Auroraspora sp. 1983a Gao Lianda, pl. VIII, 7
Calamospora atava (Naumova) McGregor 1964 1983a Gao Lianda, pl. I, 4
Calamospora nigrata (Naumova) Allen, 1965 1983a Gao Lianda, pl. I, 5
Calyptosporites sp.1 1983a Gao Lianda, pl. VII, 4-5

Calyptosporites sp.2 1983a Gao Lianda, pl. VII, 6
Cirratriradites sp. 1983a Gao Lianda, pl. V, 15
Contagisporites optivus var. vorobjebensis (Chibrikova) Owens, 1971 1983a Gao Lianda, pl. III, 10
Convolutispora amplecta (Naumova) Gao, 1983 1983a Gao Lianda, pl. II, 9
Convolutispora cancellothyra (Waltz) Gao, 1983 1983a Gao Lianda, pl. II, 8
Convolutispora implicata (Kedo) Gao, 1983 1983a Gao Lianda, pl. II, 10, 14
Convolutispora major (Kedo) Turnau, 1978 1983a Gao Lianda, pl. II, 13
Convolutispora subamplecta (Kedo) Gao, 1983 1983a Gao Lianda, pl. II, 11
Corystisporites multispinosus Richardson, 1965 1983a Gao Lianda, pls. VI, 20; VIII, 8-9
Crissisporites nidus Gao, 1983 1983a Gao Lianda, pl. II, 15-16
Cristatisporites hacquebardii Playford, 1964 1983a Gao Lianda, pl. VII, 10
Cyclogranisporites magnus (Naumova) Gao, 1983 1983a Gao Lianda, pl. I, 19
Cymbosporites formosus (Naumova) Gao, 1983 1983a Gao Lianda, pl. III, 16-18
Densosporites runcinatus Gao, 1983 1983a Gao Lianda, pl. IV, 14-15
Dibolisporites sp. 1983a Gao Lianda, pl. I, 20
Dictyotriletes submarginatus Playford, 1971 1983a Gao Lianda, pl. II, 12
Discernisporites micromanifestus (Hacquebard) Sabry et Neves, 1971 1983a Gao Lianda, pl. VII, 1-3, 7-8
Discernisporites sp. 1983a Gao Lianda, pl. VII, 9
Emphanisporites annulatus McGregor, 1961 1983a Gao Lianda, pl. III, 7
Emphanisporites cerchnus Gao, 1983 1983a Gao Lianda, pl. III, 6
Emphanisporites hibernicus Clayton, Higgs et Keegan 1977 1983a Gao Lianda, pl. III, 1-4
Emphanisporites rotatus McGregor, 1961 1983a Gao Lianda, pl. III, 5
Geminospora lemurata Balme, 1962 1983a Gao Lianda, pl. IV, 1-2
Geminospora nanus (Naumova) Gao, 1983 1983a Gao Lianda, pl. III, 20-23
Geminospora parvibasilaria (Naumova) Gao, 1983 1983a Gao Lianda, pl. III, 19, 25
Geminospora sp. 1983a Gao Lianda, pl. III, 24
Grandispora bellus (Naumova) Gao, 1983 1983a Gao Lianda, pl. VI, 4-5
Grandispora cornuta Higgs, 1975 1983a Gao Lianda, pl. V, 11
Grandispora crassis (Kedo) Gao, 1983 1983a Gao Lianda, pl. V, 9, 12
Grandispora dentata (Naumova) Gao, 1983 1983a Gao Lianda, pl. VI, 9-10
Grandispora dilectus (Naumova) Gao, 1983 1983a Gao Lianda, pl. VI, 3
Grandispora echinata Hacquebard, 1957 1983a Gao Lianda, pl. V, 10
Grandispora famenensis (Naumova) Streel, 1974 1983a Gao Lianda, pl. VI, 8
Grandispora gracilis (Kedo) Streel, 1974 1983a Gao Lianda, pl. V, 17
Grandispora meonacanthus (Naumova) Gao, 1983 1983a Gao Lianda, pl. V, 13
Grandispora multispinosa Gao, 1983 1983a Gao Lianda, pls. VI, 1-2, 12-13; VIII, 6
Grandispora sernusa (Kedo) Gao, 1983 1983a Gao Lianda, pl. VI, 11
Grandispora sp. 1983a Gao Lianda, pls. V, 16; VIII, 5
Grandispora spinulosa (Naumova) Gao, 1983 1983a Gao Lianda, pl. VI, 6
Grandispora storthyna Gao, 1983 1983a Gao Lianda, pl. VI, 7
Grandispora tener Gao, 1983 1983a Gao Lianda, pl. V, 18
Grandispora tenuispinosa (Hacquebard) Playford var. *punctata* Streel, 1974 1983a Gao Lianda, pl. V, 14
Granulatisporites rotundus (Naumova) Gao, 1983 1983a Gao Lianda, pl. I, 10
Hymenozonotriletes explanatus (Luber) Kedo, 1963 1983a Gao Lianda, pl. VI, 14-19

Laevigatosporites rarus (Naumova) Gao et Hou, 1975 1983a Gao Lianda, pl. VIII, 10-11
Leiotriletes devonicus Naumova, 1953 1983a Gao Lianda, pl. I, 1
Lophotriletes gramosus Naumova, 1953 1983a Gao Lianda, pl. II, 2
Lophozonotriletes grandis Naumova, 1953 1983a Gao Lianda, pl. IV, 6
Lophozonotriletes grumosus Naumova, 1953 1983a Gao Lianda, pl. III, 15
Lophozonotriletes sp. 1983a Gao Lianda, pls. III, 14; IV, 5
Perisaccus devonicus Naumova, 1953 1983a Gao Lianda, pl. VIII, 4
Perotriletes aculeatus Owens, 1971 1983a Gao Lianda, pl. VII, 22
Perotriletes pallidus Gao, 1983 1983a Gao Lianda, pl. VII, 20
Perotriletes sp. cf. *Diaphanospora ricinata* Balme et Hassell, 1962 1983a Gao Lianda, pl. VIII, 1
Perotriletes xizangensis Gao, 1983 1983a Gao Lianda, pl. VIII, 2
Pulvinispora depressa (Balme et Hassell) Evens, 1970 1983a Gao Lianda, pl. I, 17
Punctatisporites lasius (Waltz) Gao, 1983 1983a Gao Lianda, pl. I, 3
Punctatisporites solidus (Naumova) Gao et Hou, 1975 1983a Gao Lianda, pl. I, 2
Retispora lepidophyta (Kedo) Playford, 1976 1983a Gao Lianda, pls. IV, 12, 16-17; V, 1, 4-5, 6-8
Retispora lepidophyta var. *minor* (Kedo) Gao, 1983 1983a Gao Lianda, pl. V, 2-3
Retusotriletes communis Naumova, 1953 1983a Gao Lianda, pl. I, 9
Retusotriletes planus Dolby et Neves, 1970 1983a Gao Lianda, pl. I,7
Retusotriletes simplex Naumova, 1953 1983a Gao Lianda, pl. I, 8
Retusotriletes sp. 1983a Gao Lianda, pl. I, 6
Samarisporites concinnus Owens, 1971 1983a Gao Lianda, pl. VII, 12
Samarisporites plicatus Gao, 1983 1983a Gao Lianda, pl. VII, 11
Stenozonotriletes conformis Naumova, 1953 1983a Gao Lianda, pl. II, 20
Vallatisporites pusillites (Kedo) Dolby et Neves, 1970 1983a Gao Lianda, pl. VII, 13-15
Vallatisporites vallatus Hacquebard, 1957 1983a Gao Lianda, pl. VII, 17
Vallatisporites verrucosus Hacquebard, 1957 1983a Gao Lianda, pl. VII, 16, 18
Verruciretusispora magnifica (MeGregor) *magnifica* Owesn 1971 1983a Gao Lianda, pl. II, 6-7
Verrucosisporites omalus Gao, 1983 1983a Gao Lianda, pl. I, 24-25
Verrucosisporites sp. 1983a Gao Lianda, pl. II, 5

Guizhou

1. **Baihupo Section, Baiguoping Section, Jialaohe Section, and Wuliqiao Section, Dushan County: Zhewang Formation and Lower Gelaohe Formation**

Abundant Genera: *Geminospora*, *Cymbosporites*, *Aneurospora*, *Apiculiratusispora*, *Retusotriletes*
Aneurospora greggsii 1991 Gao Lianda, p.60
Archaeozonotriletes minutus 1991 Gao Lianda, p.60
Cristatisporites echinatus 1991 Gao Lianda, p.60
Cymbosporites cyathus 1991 Gao Lianda, p.60
Cymbosporites formosus 1991 Gao Lianda, p.60
Densosporites spitsbergensis 1991 Gao Lianda, p.60
Dictyotriletes submarginatus 1991 Gao Lianda, p.60
Discernisporites micromanifestus 1991 Gao Lianda, p.60
Dorbulispora subulaeolaris 1991 Gao Lianda, p.60

Grandispora echinata 1991 Gao Lianda, p.60
Hymenozonotriletes explanatus 1991 Gao Lianda, p.60
Raistrickia spathulata Higgs 1991 Gao Lianda, p.60
Reticulatisporites fimbriatus 1991 Gao Lianda, p.60
Rugospora felxuosa 1991 Gao Lianda, p.60
Tumulispora dentata 1991 Gao Lianda, p.60
Tumulispora rarituberculuta 1991 Gao Lianda, p.60
Tumulispora variverrucata 1991 Gao Lianda, p.60
Vallatisporites pusillites 1991 Gao Lianda, p.60
Vallatisporites vullatus 1991 Gao Lianda, p.60
Verrucosisporites mtutus 1991 Gao Lianda, p.60

2. Baihupo Section, Jialaohe Section, and Wuliqiao Section, Dushan County: Upper Gelaohe Formation

Anapiculatisporites delicatus 1991 Gao Lianda, p.60
Anaplanisporites atheticus 1991 Gao Lianda, p.60
Auroraspora macra 1991 Gao Lianda, p.60
Corbulispora subalaeolaris 1991 Gao Lianda, p.60
Densosporites spitsbergensis 1991 Gao Lianda, p.60
Dictyotriletes submarginatus 1991 Gao Lianda, p.60
Grandispora echinata 1991 Gao Lianda, p.60
Hymenozonotriletes explanatus 1991 Gao Lianda, p.60
Knoxisporites literatus 1991 Gao Lianda, p.60
Retusotriletes incohatus 1991 Gao Lianda, p.60
Spelaeotriletes crustatus 1991 Gao Lianda, p.60
Spelaeotriletes resolatus 1991 Gao Lianda, p.60
Tumulispora dentata 1991 Gao Lianda, p.60
Tumulispora dentata 1991 Gao Lianda, p.60
Tumulispora ordinaria 1991 Gao Lianda, p.60
Tumulispora rarituberculata 1991 Gao Lianda, p.60
Tumulispora rarituberculata 1991 Gao Lianda, p.60
Umbonatisporites distinctus 1991 Gao Lianda, p.60
Vallatisporites verrucosus 1991 Gao Lianda, p.60
Verrucosisporites nitidus 1991 Gao Lianda, p.60

3. Muhua Section: Lower Gedongguan Member of Wangyou Formation

Acanthotriletes serratus Naumova, 1953 1985 Gao Lianda, pl. III, 21
Acinosporites sp. 1985 Gao Lianda, pls. IV, 1; VI, 1
Aneurospora goensis Streel, 1964 1985 Gao Lianda, pl. VI, 13
Aneurospora greggsii (McGregor) Streel in Becker et al., 1974 1985 Gao Lianda, pl. VI, 11-12
Aneurospora incohatus (Sullivan) Streel in Becker et al., 1974 1985 Gao Lianda, pl. VI, 9-10
Apiculatisporis sp. 1985 Gao Lianda, pl. III, 14
Apiculiretusispora kurta Gao, 1983 1985 Gao Lianda, pl. III, 11
Archaeozonotriletes variabilis (Naumova) Allen, 1965 1985 Gao Lianda, pl. VI, 28-31

Archaeozonotriletes famenensis Naumova, 1953 1985 Gao Lianda, pl. VI, 23-25
Archaeozonotriletes fruncatus Naumova, 1953 1985 Gao Lianda, pl. VI, 26
Archaeozonotriletes pustulatus Naumova, 1953 1985 Gao Lianda, pl. VI, 27
Baculatisporites fusticulus Sullivan, 1968 1985 Gao Lianda, pl. III, 15
Convolutispora ampla Hoffmeister, Staplin et Malloy, 1955 1985 Gao Lianda, pl. IV, 24
Corbulispora subalaeolaris (Luber) Sulliban, 1964 1985 Gao Lianda, pl. V, 4, 7
Cristatisporites? sp. 1985 Gao Lianda, pl. IX, 5
Cyclogranisporites rugosus (Naumova) Gao et Hou, 1975 1985 Gao Lianda, pl. III, 13
Cymbosporites basilaris (Naumova) Gao, 1985 1985 Gao Lianda, pl. VII, 4
Dictyotriletes distinctus Naumova in Kedo, 1963 1985 Gao Lianda, pl.V, 8
Dictyotriletes trivialis Naumova in Kedo, 1963 1985 Gao Lianda, pl. V, 6
Emphanisporites rotatus McGregor, 1961 1985 Gao Lianda, pl. V, 17
Grandispora echinata Hacquebard, 1957 1985 Gao Lianda, pl. IX, 1
Grandispora polyacanthus (Naumova) Gao, 1985 1985 Gao Lianda, pl. VIII, 9
Hymenozonotriletes explanatus (Luber) Kedo, 1963 1985 Gao Lianda, pl. VIII, 7
Hymenozonotriletes sp. 1985 Gao Lianda, pl. VIII, 4
Hymenozonotriletes tenellus Naumova, 1953 1985 Gao Lianda, pl. VIII, 5
Hymenozonotriletes varius Naumova, 1953 1985 Gao Lianda, pl. VIII, 6
Laevigatosporites sp. 1985 Gao Lianda, pl. IX, 18
Lophotriletes atratus Naumova, 1953 1985 Gao Lianda, pl. IV, 4
Lophozonotriletes curvatus Naumova, 1953 1985 Gao Lianda, pl. V, 19
Lophozonotriletes excisus Naumova, 1953 1985 Gao Lianda, pl. V, 20
Punctatisporites lasius (Waltz) Gao, 1983 1985 Gao Lianda, pl. III, 6
Punctatisporites subminor (Naumova) Gao et Hou, 1975 1985 Gao Lianda, pl. III, 5
Pustulatisporites gibberosus (Hacquebard) Playford, 1964 1985 Gao Lianda, pl. IV, 18
Raistrickia macrura (Luber) Dolby et Neves, 1970 1985 Gao Lianda, pl. III, 25
Raistrickia tonsilisa (Ischenko) Gao, 1985 1985 Gao Lianda, pl. IV, 3
Retispora? sp. 1985 Gao Lianda, pl. IX, 10
Retusotriletes communis Naumova, 1953 1985 Gao Lianda, pl. III, 8
Retusotriletes dubius (Eisenack) Richardson, 1965 1985 Gao Lianda, pl. III, 9
Samarisporites concinnus Owens, 1971 1985 Gao Lianda, pl. VIII, 2
Samarisporites sp. 1985 Gao Lianda, pl. VIII, 3
Stenozonotriletes stenomarginatus Naumova in Kedo, 1963 1985 Gao Lianda, pl. VI, 5
Tumulispora dentata (Hughes et Playford) Turnau, 1975 1985 Gao Lianda, pls. VII, 11-14; X, 9-11
Tumulispora macrotuberculata 1985 1985 Gao Lianda, pl. VII, 16-18
Tumulispora major (Kedo) Gao, 1985 1985 Gao Lianda, pl. VII, 19
Tumulispora turgiduta Gao, 1985 1985 Gao Lianda, pl. VII, 5-6
Vallatisporites pusillites (Kedo) Dolby et Neves, 1970 1985 Gao Lianda, pl. IX, 11-16
Vallatisporites sp. 1985 Gao Lianda, pl. IX, 4
Vallatisporites sp.2 1985 Gao Lianda, pl. IX, 6-7
Vallatisporites verrucosus Hacquebard, 1957 1985 Gao Lianda, pl. IX, 8-10
Verrucosisporites depressus Winslow, 1962 1985 Gao Lianda, pl. IV, 9-10
Verrucosisporites nitidus (Naumova) Playford, 1964 1985 Gao Lianda, pl. IV, 11, 14

Late Famennian to Early Tournaisian

Zhejiang

Niutoushan, Changxing County: Upper Wutung Formation

Acanthotriletes cf. *tennispinosus* Naumova 1987 Yan Youyin, p. 8
Acanthotriletes sp. 1987 Yan Youyin, p. 8
Anapiculatisporites cf. *tarsus* Playford 1987 Yan Youyin, p. 8
Apiculatasporites sp. 1987 Yan Youyin, p. 8
Apiculiretusispora flexuosa Hacquebard 1987 Yan Youyin, p. 8
Auroraspora macra Sullivan 1987 Yan Youyin, p. 8
Baculatisporites fusticulus Sullivan 1987 Yan Youyin, p. 8
Calamospora cf. *microrugosa* (Ibrahim) Schopf, Wilson et Bentall 1987 Yan Youyin, p. 8
Calamospora sp. 1987 Yan Youyin, p. 8
Convolutispora sp. 1987 Yan Youyin, p. 8
Dibolisporites sp. 1987 Yan Youyin, p. 8
Discernisporites crenulatus (Playford) Clayton 1987 Yan Youyin, p. 8
Endosporites sp. 1987 Yan Youyin, p. 8
Grandispora echinata Hacquebord 1987 Yan Youyin, p. 8
Granulatisporites cf. *crenulatus* Playford 1987 Yan Youyin, p. 8
Hymenozonotriletes cassiculus Higgs 1987 Yan Youyin, p. 8
Knoxisporites sp. 1987 Yan Youyin, p. 8
Lycospora cf. *magnifica* McGregor 1987 Yan Youyin, p. 8
Perotriletes sp. 1987 Yan Youyin, p. 8
Punctatisporites cf. *glaber* (Naum.) Playford 1987 Yan Youyin, p. 8
Punctatisporites cf. *irrasus* Hacquebord 1987 Yan Youyin, p. 8
Punctatisporites sp. 1987 Yan Youyin, p. 8
Raistrickia cf. *clavata* (Hacquebard) Playford 1987 Yan Youyin, p. 8
Remysporites cf. *magnificus* (Horst) Butterworth et Williams 1987 Yan Youyin, p. 8
Retusotriletes incohatus Sullivan 1987 Yan Youyin, p. 8
Rugospora versabilis (Kedo) Streel 1987 Yan Youyin, p. 8
Spelaeotriletes pretiosus (Playford) Neves et Belt 1987 Yan Youyin, p. 8
Vallatisporites cf. *vallatus* Hacquebard 1987 Yan Youyin, p. 8
Vallatisporites ciliaris (Luber) Sullivan 1987 Yan Youyin, p. 8
Verrucosisporites nitidus (Naum.) Playford 1987 Yan Youyin, p. 8

Early Tournaisian

Yunnan

Nongba Section, Sipaishan Town, and Mengshengqiao Section, Gengma County: Middle Nongba Formation

Anapiculatisporites hystricosus Playford, 1971 1995 Yang Weiping et Jiajinhua, pl.II, 8
Apiculiretusispora granulata Owens, 1971 1995 Yang Weiping et Jiajinhua, pl.II, 10
Auroraspora macra 1995 Yang Weiping et Jiajinhua, p.66
Baculatisporites fusticulus 1995 Yang Weiping et Jiajinhua, p.66

Grandispora cornuta Higgs, 1975 1995 Yang Weiping et Jiajinhua, pl.II, 1-2
Grandispora echinata Hacquebard, 1957 1995 Yang Weiping et Jiajinhua, pl.II, 5
Grandispora notensis Playford, 1971 1995 Yang Weiping et Jiajinhua, pl.II, 3
Grandispora spiculifera Playford, 1976 1995 Yang Weiping et Jiajinhua, pl.II, 4
Granulatisporites frustulentus 1995 Yang Weiping et Jiajinhua, p.66
Latospora sp. 1995 Yang Weiping et Jiajinhua, pl.II, 11
Neoraistrickia sp. 1995 Yang Weiping et Jiajinhua, pl.II, 12
Punctatisporites irrasus 1995 Yang Weiping et Jiajinhua, p.66
Raistrickia cf. *condylosa* 1995 Yang Weiping et Jiajinhua, p.66
Retusotriletes incohatus 1995 Yang Weiping et Jiajinhua, p.66
Schopfites claviger Sullivan emend. Higgs et al., 1988 1995 Yang Weiping et Jiajinhua, pl.II, 6
Spelaeotriletes balteatus 1995 Yang Weiping et Jiajinhua, p.66
Spelaeotriletes crustatus 1995 Yang Weiping et Jiajinhua, p.66
Spelaeotriletes obtusus 1995 Yang Weiping et Jiajinhua, p.66
Spinozonotriletes uncatus Hacquebard, 1957 1995 Yang Weiping et Jiajinhua, pl.II, 9
Vallatisporites verrucosus 1995 Yang Weiping et Jiajinhua, p.66
Velamisporites caperatus 1995 Yang Weiping et Jiajinhua, p.66
Verrucosisporites cf. *V. scoticus* Sullivan, 1968 1995 Yang Weiping et Jiajinhua, pl.II, 7

Guangdong

Dasaiba Section, Changlai Town, Lechang City: Lower Dasaiba Formation
Acanthotriletes pusillus Ishcheko 1997 Zhao Ruxuan et Qin Guorong, pl. I, 4, 5
Anapiculatisporites hystricosus Playford 1997 Zhao Ruxuan et Qin Guorong, pl. I, 26
Anapiculatisporites rotundus Zhao et Qin 1997 Zhao Ruxuan et Qin Guorong, pl. I, 7
Aneurospora cf. *greggsii* McGregor 1997 Zhao Ruxuan et Qin Guorong, pl. I, 6
Apiculatisporites baccatus var. *densatus* Staplin 1997 Zhao Ruxuan et Qin Guorong, pl. I, 21
Calamospora nigrata Naumora 1997 Zhao Ruxuan et Qin Guorong, pl. I, 12
Cyclogranisporites dasaibaensis Zhao et Qin 1997 Zhao Ruxuan et Qin Guorong, pl. I, 16-18
Densosporites anulatus Loose 1997 Zhao Ruxuan et Qin Guorong, pl. I, 10
Densosporites spitsbergensis Playford 1997 Zhao Ruxuan et Qin Guorong, pl. I, 13
Diatsmozonotriletes sp. 1997 Zhao Ruxuan et Qin Guorong, pl. I, 15
Dictyotriletes minor Zhao et Qin 1997 Zhao Ruxuan et Qin Guorong, pl. I, 1
Dictyotriletes submarginatus Playford 1997 Zhao Ruxuan et Qin Guorong, pl. I, 28
Granulatisporites granulatus Ibrahim 1997 Zhao Ruxuan et Qin Guorong, pl. I, 2
Granulatisporites minutus Potunié et Kremp 1997 Zhao Ruxuan et Qin Guorong, pl. I, 20
Knoxisporites literatus Waltz 1997 Zhao Ruxuan et Qin Guorong, pl. I, 27
Knoxisporites minutus Zhao et Qin 1997 Zhao Ruxuan et Qin Guorong, pl. I, 3
Lycospora granianellatus Staplin 1997 Zhao Ruxuan et Qin Guorong, pl. I, 14
Lycospora granulatus Kosanke 1997 Zhao Ruxuan et Qin Guorong, pl. I, 25
Lycospora orbicula Pot. et Kr. 1997 Zhao Ruxuan et Qin Guorong, pl. I, 22
Lycospora pusilla Ibrahim 1997 Zhao Ruxuan et Qin Guorong, pl. I, 24
Lycospora verrucosus Kos. 1997 Zhao Ruxuan et Qin Guorong, pl. I, 8
Murospora minutus Gao 1997 Zhao Ruxuan et Qin Guorong, pl. I, 23

Punctatisporites debilis Hacg. 1997 Zhao Ruxuan et Qin Guorong, pl. I, 11
Reticulatisporites macroreticulatus Naurmova 1997 Zhao Ruxuan et Qin Guorong, pl. I, 9
Retusotriletes communis Zhao et Qin 1997 Zhao Ruxuan et Qin Guorong, pl. I, 19
Verrucosisporites nitidus (Naum.) Playford 1997 Zhao Ruxuan et Qin Guorong, pl. I, 29

Hukeng, Renhua County, Shaoguan City: Maozifeng Formation
Acanthotriletes parispinosus Luber 1997 Zhao Ruxuan et Qin Guorong, pl. II, 41-43
Acanthotriletes sp. 1990 Zhao Ruxuan et Qin Guorong, fig. 6
Auroraspora sp. 1990 Zhao Ruxuan et Qin Guorong, fig. 6
Calamospora cf. *nigrata* Naumova 1997 Zhao Ruxuan et Qin Guorong, pl. II, 29
Calamospora pusilla Pepper 1997 Zhao Ruxuan et Qin Guorong, pl. II, 7, 34
Calamospora sp. 1990 Zhao Ruxuan et Qin Guorong, fig. 6
Cyclogranisporites sp. 1990 Zhao Ruxuan et Qin Guorong, fig. 6; 1997 Zhao Ruxuan et Qin Guorong, pl. II, 39
Densosporites anulatus 1997 Zhao Ruxuan et Qin Guorong, pl. II, 27, 28, 33, 49
Densosporites spitsbergensis Playford 1997 Zhao Ruxuan et Qin Guorong, pl. II, 50
Densosporites sp. 1990 Zhao Ruxuan et Qin Guorong, fig. 6
Dictyotriletes sp. 1990 Zhao Ruxuan et Qin Guorong, fig. 6
Granulatisporites minutus Pot. et Kr. 1997 Zhao Ruxuan et Qin Guorong, pl. II, 24, 25
Granulatisporites muninensis Allen 1997 Zhao Ruxuan et Qin Guorong, pl. II, 17, 18
Granulatisporites orbiculus Zhao et Qin 1997 Zhao Ruxuan et Qin Guorong, pl. II, 45
Granulatisporites tenuis Pepper 1997 Zhao Ruxuan et Qin Guorong, pl. II, 15, 16
Granulatisporites sp. 1990 Zhao Ruxuan et Qin Guorong, fig. 6; 1997 Zhao Ruxuan et Qin Guorong, pl. II, 14
Knoxisporites pristinus Sullivan 1997 Zhao Ruxuan et Qin Guorong, pl. II, 37
Knoxisporites sp. 1990 Zhao Ruxuan et Qin Guorong, fig. 6; 1997 Zhao Ruxuan et Qin Guorong, pl. II, 10-12, 38, 51
Laevigatosporites sp. 1990 Zhao Ruxuan et Qin Guorong, fig. 6; 1997 Zhao Ruxuan et Qin Guorong, pl. II, 6
Leiotriletes sp. 1990 Zhao Ruxuan et Qin Guorong, fig. 6; 1997 Zhao Ruxuan et Qin Guorong, pl. II, 22
Lophozonotriletes sp. 1990 Zhao Ruxuan et Qin Guorong, fig. 6
Lycospora microgranulata Bhardwaj 1997 Zhao Ruxuan et Qin Guorong, pl. II, 35
Lycospora orbicula 1997 Zhao Ruxuan et Qin Guorong, pl. II, 1, 2, 4, 13, 19, 20, 31
Lycospora pusilla Jbrahim 1997 Zhao Ruxuan et Qin Guorong, pl. II, 3, 26, 36
Lycospora sp. 1990 Zhao Ruxuan et Qin Guorong, fig. 6
Punctatosporites minutus Ibrahim 1997 Zhao Ruxuan et Qin Guorong, pl. II, 5
Punctatosporites sp. 1990 Zhao Ruxuan et Qin Guorong, fig. 6
Raistrickia sp. 1990 Zhao Ruxuan et Qin Guorong, fig. 6; 1997 Zhao Ruxuan et Qin Guorong, pl. II, 21
Retusotriletes communis Nanmova 1997 Zhao Ruxuan et Qin Guorong, pl. II, 8, 9
Retusotriletes sp. 1990 Zhao Ruxuan et Qin Guorong, fig. 6
Sporites sp. 1990 Zhao Ruxuan et Qin Guorong, fig. 6
Stenozonotriletes rasilis Kedo 1997 Zhao Ruxuan et Qin Guorong, pl. II, 46, 47
Stenozonotriletes stenomarginatus Naumova et Kedo 1997 Zhao Ruxuan et Qin Guorong, pl. II, 40
Stenozonotriletes sp. 1990 Zhao Ruxuan et Qin Guorong, fig. 6; 1997 Zhao Ruxuan et Qin Guorong, pl. II, 23, 44, 48
Triletes sp. 1990 Zhao Ruxuan et Qin Guorong, fig. 6

Hunan

Shimen Section: Lower Changyang Formation

Apiculatisporis sp. 1990 Gao Lianda, pl. I, 5

Auroraspora macra Sullivan, 1968 1990 Gao Lianda, pl. I, 21

Crassispora trychera Neves et Ioannides, 1974 1990 Gao Lianda, pl. I, 24

Perotriletes sp. 1990 Gao Lianda, pl. II, 21

Tumulispora raritubercalata (Luber) Potonié, 1966 1990 Gao Lianda, pl. II, 1

Zhejiang

Xindian Section, Fuyang City: Upper Xihu Formation

Acanthotriletes cf. *ignotus* Kedo, 1957 1993 He Shengce et Ouyang Shu, pl.3, 1, 5

Acanthotriletes simpex 1992 Yan Tiezeng, p. 112

Acanthotriletes sp. 1992 Yan Tiezeng, p. 112

Ancyrospora sp. 1992 Yan Tiezeng, p. 112

Apiculiretusispora granulata 1992 Yan Tiezeng, p. 112

Apiculiretusispora hunanensis (Hou) Ouyang et Chen, 1987 1992 Yan Tiezeng, p. 112; 1993 He Shengce et Ouyang Shu, pl.1, 7

Apiculiretusispora nitida Owens, 1971 1993 He Shengce et Ouyang Shu, pl.1, 17

Auroraspora cf. *poljessica* (Kedo) Streel in B. B. S. T., 1974 1993 He Shengce et Ouyang Shu, pl.4, 3-4

Auroraspora cf. *pseudocrista* Ahmed, 1980 1993 He Shengce et Ouyang Shu, pl.3, 9

Auroraspora macra Sullivan, 1968 1992 Yan Tiezeng, p. 112; 1993 He Shengce et Ouyang Shu, pl.2, 5

Calamospora sp. 1992 Yan Tiezeng, p. 112

cf. *Hymenozonotriletes explanatus* (Luber) Kedo, 1963 1993 He Shengce et Ouyang Shu, pl.3, 12

Cirratiradites sp. 1992 Yan Tiezeng, p. 112

Cordylosporites papillatus (Naumova) Playford, 1985 1993 He Shengce et Ouyang Shu, pl.4, 5, 7-8

Cyclogranisporites baoyingensis Ouyang et Chen, 1987 1993 He Shengce et Ouyang Shu, pl.1, 9

Cymbosporites promiscuous 1992 Yan Tiezeng, p. 112

Densosporites cf. *tersus* Waltz, 1993 1993 He Shengce et Ouyang Shu, pl.1, 16, 18

Densosporites xinhuanensis Hou, 1982 1993 He Shengce et Ouyang Shu, pl.2, 9-10

Dibolisporites distinctus (Clayton) Playford, 1976 1993 He Shengce et Ouyang Shu, pl.3, 4, 10

Dictyotriletes cf. *crassipterus* Naumova ex Kedo, 1963 1993 He Shengce et Ouyang Shu, pl.2, 4

Dictyotriletes sp. 1992 Yan Tiezeng, p. 112

Dictyotriletes sp. A 1993 He Shengce et Ouyang Shu, pl.2, 3

Dictyotriletes sp. B 1993 He Shengce et Ouyang Shu, pl.2, 8

Discernisporites micromanifestus (Hacquebard) Sabry et Neves, 1971 1993 He Shengce et Ouyang Shu, pl.4, 1

Emphanisporites sp. 1992 Yan Tiezeng, p. 112

Endosporites cf. *gilmorensis* 1992 Yan Tiezeng, p. 112

Foveosporites cf. *pellucidus* Playford et Helby 1968 1993 He Shengce et Ouyang Shu, pl.2, 1

Grandispora sp. 1992 Yan Tiezeng, p. 112

Grandispora sp. A 1993 He Shengce et Ouyang Shu, pl.1, 4, 11

Grandispora sp. B 1993 He Shengce et Ouyang Shu, pl.1, 10; pl.2, 12

Hymenozonotriletes explanatus (Luber) Kedo, 1963 1992 Yan Tiezeng, p. 112

Hymenozonotriletes sp. 1993 He Shengce et Ouyang Shu, pl.4, 2, 6, 9-12

Knoxisporites cf. *literatus* (Waltz) Playford, 1963 1993 He Shengce et Ouyang Shu, pl.3, 7-8

Knoxisporites literatus (Waltz) Playford, 1963 1993 He Shengce et Ouyang Shu, pl.1, 12, 15; pl.2, 7; pl.3, 2-3
Leiotriletes cf. *dissimilis* McGregor, 1960 1993 He Shengce et Ouyang Shu, pl.2, 2
Leiotriletes simplex 1992 Yan Tiezeng, p. 112
Leiotriletes sp. 1992 Yan Tiezeng, p. 112
Lycospora denticulata 1992 Yan Tiezeng, p. 112
Punctatisporites sp. 1993 He Shengce et Ouyang Shu, pl.1, 3
Reticulatisporites cf. *mediareticulatus* Ibr. 1992 Yan Tiezeng, p. 112
Reticulatisporites poltatus 1992 Yan Tiezeng, p. 112
Reticulatisporites sp. 1993 He Shengce et Ouyang Shu, pl.3, 6
Retusotriletes goensis 1992 Yan Tiezeng, p. 112
Rhabdosporites langi 1992 Yan Tiezeng, p. 112
Rhabdosporites porvulus 1992 Yan Tiezeng, p. 112
Spinozonotriletes sp. 1992 Yan Tiezeng, p. 112
Stenozonotriletes sp. 1992 Yan Tiezeng, p. 112
Verrucosisporites sp. 1993 He Shengce et Ouyang Shu, pl.1, 14

Jiangxi

Xiaomu Section, Chengxiang Town, Quannan County: Liujiatang Formation
Aneurospora chinensis (Ouyang et Chen) Wen et Lu, 1993 1993 Wen Zicai et Lu Lichang, pl.2, 23-24
Apiculiretusispora tenera Wen et Lu, 1993 1993 Wen Zicai et Lu Lichang, pl.2, 11-16
Crassispora parva Butterworth et Mahdi 1993 Wen Zicai et Lu Lichang, pl.3, 11-12
Granulatisporites minimus Wen et Lu, 1993 1993 Wen Zicai et Lu Lichang, pl.1, 28-29
Leiotriletes laevis Naumova 1993 Wen Zicai et Lu Lichang, pl.1, 4-5
Leiotriletes macrothelis Wen et Lu, 1993 1993 Wen Zicai et Lu Lichang, pl.1, 6-9
Lycospora cf. *tenuispinosa* Ouyang et Chen 1993 Wen Zicai et Lu Lichang, pl.4, 25-26
Retusotriletes asthenolabratus Hou 1993 Wen Zicai et Lu Lichang, pl.1, 16-17
Retusotriletes simplex Naumova 1993 Wen Zicai et Lu Lichang, pl.1, 24-25

Jiangsu

1. Baojia Village, Jurong County: Upper Leigutai Member
Anapiculatisporites cf. *reductus* Playford, 1985 Chen Yongxiang et Ouyangshu, p. 268
Anapiculatisporites cf. *reductus* Playford, 1978 1987a Ouyang Shu et Chen Yongxiang, pl.5, 20
Calamospora cf. *pedata* Kos. 1985 Chen Yongxiang et Ouyangshu, p. 268
Calamospora membrane Bharad. 1985 Chen Yongxiang et Ouyangshu, p. 268
Calamospora pallid (Loosa) S., W. et B. 1985 Chen Yongxiang et Ouyangshu, p. 268
Chomotriletes rarivittatus Ouyang et Chen, 1987 1987a Ouyang Shu et Chen Yongxiang, pl.18, 13-15
Convolutispora mellita H., S. et M. 1985 Chen Yongxiang et Ouyangshu, p. 268
Convolutispora mellita H., S. et M., 1955 1987a Ouyang Shu et Chen Yongxiang, pl.6, 18-19
Convolutispora planus Hughes et Playford, 1985 Chen Yongxiang et Ouyangshu, p. 268
Convolutispora planus Hughes et Playford, 1961 1987a Ouyang Shu et Chen Yongxiang, pl.6, 20-21
Crassilagenicula cf. *baccaefera* (Dijk.) Dybova-Jachowicz et al. 1979 1985 Chen Yongxiang et Ouyangshu, pl. II, 6
Crassilagenicula simplex (Zerndt) Dybova-Jachowicz et al. forma *canaliculata* f. nov. 1985 Chen Yongxiang et Ouyangshu, pl. II, 3-5

Cyclogranisporites commodus Playf. 1985 Chen Yongxiang et Ouyangshu, p. 268
Cyclogranisporites delicatus Ouyang et Chen, 1987 1987a Ouyang Shu et Chen Yongxiang, pl.6, 5
Cyclogranisporites microgranus Bharad 1985 Chen Yongxiang et Ouyangshu, p. 268
Cyclogranisporites pisticus Playford, 1985 Chen Yongxiang et Ouyangshu, p. 268
Cyclogranisporites pisticus Playford, 1978 1987a Ouyang Shu et Chen Yongxiang, pl.3, 15-17
Cyclogranisporites pseudozonatus Ouyang et Chen, 1987 1987a Ouyang Shu et Chen Yongxiang, pl.4, 3-5, 8
Cystosporites sp. 1985 Chen Yongxiang et Ouyangshu, pl. II, 7
Dictyotriletes cf. *falsus* Potonié et Kremp, 1955 1987a Ouyang Shu et Chen Yongxiang, pl.6, 11
Dictyotriletes cf. *varius* Naum. 1985 Chen Yongxiang et Ouyangshu, p. 268
Dictyotriletes cf. *varius* Naumova, 1953 1987a Ouyang Shu et Chen Yongxiang, pl.6, 10
Endosporites cf. *micromanifestus* Hacq. 1985 Chen Yongxiang et Ouyangshu, p. 268
Endosporites cf. *micromanifestus* Hacq. 1957 1987a Ouyang Shu et Chen Yongxiang, pl.14, 9
Granulatisporites unpromptus Ouyang et Chen, 1987 1987a Ouyang Shu et Chen Yongxiang, pl.4, 10
Hymenospora cf. *H.* caperata Felix et Burb. 1985 Chen Yongxiang et Ouyangshu, p. 268
Knoxisporites literatus (Waltz) Playford, 1985 Chen Yongxiang et Ouyangshu, p. 268
Knoxisporites literatus (Waltz) Playford, 1963 1987a Ouyang Shu et Chen Yongxiang, pl.6, 13
Lagenicula cf. *horrida* Zerndt, 1934 1985 Chen Yongxiang et Ouyangshu, pls. I, 1-6; II, 1, 2
Lagenicula sp. 1985 Chen Yongxiang et Ouyangshu, pl. II, 9
Lagenicula sp.B 1987 Chen Yongxiang et Ouyang Shu, pl.1, 7 7a
Lagenicula wutungiana Chen et Ouyang, 1985 1985 Chen Yongxiang et Ouyangshu, pls. I, 7-12; II, 8
Lagenoisporites sp. 1987 Chen Yongxiang et Ouyang Shu, pl.1, 4
Leiotriletes laevis Naum. 1985 Chen Yongxiang et Ouyangshu, p. 268
Leiotriletes simplex Naum. 1985 Chen Yongxiang et Ouyangshu, p. 268
Leiotriletes trivialis Naum. 1985 Chen Yongxiang et Ouyangshu, p. 268
Leiozonotriletes extensus Ouyang et Chen, 1987 1987a Ouyang Shu et Chen Yongxiang, pl.14, 8
Lycospora denticulate Bharad. 1985 Chen Yongxiang et Ouyangshu, p. 268
Pterospermella mirabilis Ouyang et Chen, 1987 1987a Ouyang Shu et Chen Yongxiang, pl.19, 1-3
Punctatisporites densipunctatus Ouyang et Chen, 1987 1987a Ouyang Shu et Chen Yongxiang, pl.3, 20-21
Punctatisporites rotundatus Naum. 1985 Chen Yongxiang et Ouyangshu, p. 268
Reticulatisporites cancellatus (Waltz) Bharad. et Venkat. 1985 Chen Yongxiang et Ouyangshu, p. 268
Reticulatisporites cancellatus (Waltz) Playford, 1962 1987a Ouyang Shu et Chen Yongxiang, pl.6, 22-23
Reticulatisporites cf. *mediareticulatus* (Ibr.) Pot. et Kr. 1985 Chen Yongxiang et Ouyangshu, p. 268
Reticulatisporites cf. *mediareticulatus* (Ibr.) Potonié et Kremp, 1955 1987a Ouyang Shu et Chen Yongxiang, pl.6, 16-17
Reticulatisporites verrucilabiatus Ouyang et Chen, 1987 1987a Ouyang Shu et Chen Yongxiang, pl.6, 24-25
Retusotriletes communis Naumova 1985 Chen Yongxiang et Ouyangshu, p. 268
Retusotriletes communis Naumova, 1953 1987a Ouyang Shu et Chen Yongxiang, pl.3, 4-6
Retusotriletes rotundus (Streel) Streel, 1967 1987a Ouyang Shu et Chen Yongxiang, pl.3, 1-3
Retusotriletes triangulates (Str.) Str. 1985 Chen Yongxiang et Ouyangshu, p. 268
Rugospora acutiplicata Ouyang et Chen, 1987 1987a Ouyang Shu et Chen Yongxiang, pl.12, 7-9
Stenozonotriletes cf. *extensus* Naumova 1985 Chen Yongxiang et Ouyangshu, p. 268
Stenozonotriletes pumilus (Waltz) Naumova 1985 Chen Yongxiang et Ouyangshu, p. 268
Sublagenicula nudoides Chen et Ou, 1987 1987 Chen Yongxiang et Ouyang Shu, pl.1, 1-3
Velamisporites perinatus (Hughes et Playf.) Playford, 1985 Chen Yongxiang et Ouyangshu, p. 268
Velamisporites pulchellus Ouyang et Chen, 1987 1987a Ouyang Shu et Chen Yongxiang, pl.9, 12-18

2. Chengjiao Section, Baoying County, Yangzhou City: Upper Leigutai Member

Aneurospora cf. *semizonalis* 1987b Ouyang Shu et Chen Yongxiang, p. 200
Apiculiretusispora setosa 1987b Ouyang Shu et Chen Yongxiang, p. 200
Auroraspora macra 1987b Ouyang Shu et Chen Yongxiang, p. 200
Auroraspora pallid 1987b Ouyang Shu et Chen Yongxiang, p. 200
Auroraspora tenuis 1987b Ouyang Shu et Chen Yongxiang, p. 200
Calamospora pedata 1987b Ouyang Shu et Chen Yongxiang, p. 200
Camptotriletes cf. *certus* 1987b Ouyang Shu et Chen Yongxiang, p. 200
Crassispora cf. *kosankei* 1987b Ouyang Shu et Chen Yongxiang, p. 200
Cyclogranisporites baoyingensis 1987b Ouyang Shu et Chen Yongxiang, p. 200
Densosporites penitus 1987b Ouyang Shu et Chen Yongxiang, p. 200
Dibolisporites distinctus 1987b Ouyang Shu et Chen Yongxiang, p. 200
Dibolisporites microspicatus 1987b Ouyang Shu et Chen Yongxiang, p. 200
Discernisporites? sp. 1987b Ouyang Shu et Chen Yongxiang, p. 200
Endosporites micromanifestus 1987b Ouyang Shu et Chen Yongxiang, p. 200
Endosporites parvus 1987b Ouyang Shu et Chen Yongxiang, p. 200
Grandispora sp. 1987b Ouyang Shu et Chen Yongxiang, p. 200
Granulatisporites sp. 1987b Ouyang Shu et Chen Yongxiang, p. 200
Gravisporites minutes 1987b Ouyang Shu et Chen Yongxiang, p. 200
Hymenozonotriletes explanatus 1987b Ouyang Shu et Chen Yongxiang, p. 200
Lophotriletes uncatus 1987b Ouyang Shu et Chen Yongxiang, p. 200
Monilospora sp. 1987b Ouyang Shu et Chen Yongxiang, p. 200
Punctatisporites debilis 1987b Ouyang Shu et Chen Yongxiang, p. 200
Punctatisporites nitidus 1987b Ouyang Shu et Chen Yongxiang, p. 200
Reticulatisporites cancellatus 1987b Ouyang Shu et Chen Yongxiang, p. 200
Retusotriletes cf. *simplex* 1987b Ouyang Shu et Chen Yongxiang, p. 200
Schopfites claviger 1987b Ouyang Shu et Chen Yongxiang, p. 200
Spelaeotriletes echinatus 1987b Ouyang Shu et Chen Yongxiang, p. 200
Velamisporites cf. *vermiculatus* 1987b Ouyang Shu et Chen Yongxiang, p. 200
Verruciretusispora semilucensis 1987b Ouyang Shu et Chen Yongxiang, p. 200

3. Chenjiabian, Jiangning District, Nanjing City: Chenjiabian Formation

Apiculatisporites cf. *maculosus* (Knox) Pot. et Kr. 1987 Yan Youyin, p. 4-5
Cyclobaculisporites cf. *grandiverrucosus* (Kos.) Bhaid. 1987 Yan Youyin, p. 4-5
Densosporites spinosus Dybova et Jachowicy 1987 Yan Youyin, p. 4-5
Discernisporites crenulatus (Playford) Clayton 1987 Yan Youyin, p. 4-5
Granulatisporites microgranifer (Ibrahim) Pat. et Kr. 1987 Yan Youyin, p. 4-5
Hymenozonotriletes explanatus (Luber) Kedo 1987 Yan Youyin, p. 4-5
Pulvinispora scolecophora Neves et Ioannides 1987 Yan Youyin, p. 4-5
Spelaeotriletes cf. *haltcatus* (Playford) Higgs 1987 Yan Youyin, p. 4-5
Spelaeotriletes pretiosus (Playford) Neves et Belt 1987 Yan Youyin, p. 4-5
Verrucosisporites nitidus (Naum.) Playford 1987 Yan Youyin, p. 4-5
Aneurospora macra Sullivan 1987 Yan Youyin, p. 4-5

Aneurospora incohatus (Sullivan) Streel 1987 Yan Youyin, p. 4-5
Angulisporites sp. 1987 Yan Youyin, p. 4-5
Apiculatisporis sp. 1987 Yan Youyin, p. 4-5
Apiculiretusispora sp. 1987 Yan Youyin, p. 4-5
Auroraspora sp. 1987 Yan Youyin, p. 4-5
Auroraspora macra Sullivan 1987 Yan Youyin, p. 4-5
Baculatisporites fusticulus Sullivan 1987 Yan Youyin, p. 4-5
Calamospora cf. *parva* Guennel 1987 Yan Youyin, p. 4-5
Cictyotriletes submarginatus Playford 1987 Yan Youyin, p. 4-5
Convolutispora circumvallata Clayton 1987 Yan Youyin, p. 4-5
Crassispora trychera Neves et Ioannides 1987 Yan Youyin, p. 4-5
Densosporites spitsbergensis Playford 1987 Yan Youyin, p. 4-5
Dictyotriletes submarginatus Playford 1987 Yan Youyin, p. 4-5
Foveolatisporites sp. 1987 Yan Youyin, p. 4-5
Grandispora sp. 1987 Yan Youyin, p. 4-5
Grandispora echinata Hacquebord 1987 Yan Youyin, p. 4-5
Grandispora microseta (Kedo) Streel 1987 Yan Youyin, p. 4-5
Grandispora trychera Neves et Ioannides 1987 Yan Youyin, p. 4-5
Knoxisporites literatus (Walty) Playford 1987 Yan Youyin, p. 4-5
Latosporites sp. 1987 Yan Youyin, p. 4-5
Lycospora pusilla (Ibrahim) Somers 1987 Yan Youyin, p. 4-5
Lycospora cf. *pusilla* (Ibrahim) Somers 1987 Yan Youyin, p. 4-5
Punctatisporites irrasus Hacquebard 1987 Yan Youyin, p. 4-5
Punctatisporites sp. 1987 Yan Youyin, p. 4-5
Raistrickia sp. 1987 Yan Youyin, p. 4-5
Remysporites sp. 1987 Yan Youyin, p. 4-5
Retusotriletes sp. 1987 Yan Youyin, p. 4-5
Retusotriletes incohatus Sullivan 1987 Yan Youyin, p. 4-5
Retusotriletes planus Dolby et Neves 1987 Yan Youyin, p. 4-5
Rugospora flexuosa (Jusch) Streel 1987 Yan Youyin, p. 4-5
Rugospora minuta Neves et Ioannides 1987 Yan Youyin, p. 4-5
Schopfites claviger Sullivan 1987 Yan Youyin, p. 4-5
Stenozonotriletes sp. 1987 Yan Youyin, p. 4-5
Vallatisporites sp. 1987 Yan Youyin, p. 4-5
Vallatisporites vallatus Hacquebard 1987 Yan Youyin, p. 4-5
Vallatisporites verrucosus Hacquebord 1987 Yan Youyin, p. 4-5
Verrucosisporites sp. 1987 Yan Youyin, p. 4-5

4. Kongshan, Nanjing City: Wutung Formation

Acanthotriletes sp. 1988 Cai Chongyang et al., p. 178
Aneurospora gregsii 1988 Cai Chongyang et al., p. 178
Auroraspora macra 1988 Cai Chongyang et al., p. 178
Cyclogranisporites microgranus 1988 Cai Chongyang et al., p. 178
Cymbosporites sp. 1988 Cai Chongyang et al., p. 178

Dibolisporites distinctus 1988 Cai Chongyang et al., p. 178
Dibolisporites upensis 1988 Cai Chongyang et al., p. 178
Grandispora apicilaris 1988 Cai Chongyang et al., p. 178
Grandispora echinata 1988 Cai Chongyang et al., p. 178
Knoxisporites literatus 1988 Cai Chongyang et al., p. 178
Retusotriletes simplex 1988 Cai Chongyang et al., p. 178
Spelaeotriletes echinatus 1988 Cai Chongyang et al., p. 178
Velamisporites cf. *vermiculatus* 1988 Cai Chongyang et al., p. 178
Verrucosisporites nitidus 1988 Cai Chongyang et al., p. 178

5. Cishan, Nanjing City: Cishan Formation

Aneurospora incohatus 1987 Li Hanmin et al., p. 117-118
Apiculiretusispora nitida 1987 Li Hanmin et al., p. 117-118
Auroraspora macra 1987 Li Hanmin et al., p. 117-118
Baculatisporites fusticulus 1987 Li Hanmin et al., p. 117-118
Baculatisporites sp. 1987 Li Hanmin et al., p. 117-118
Calamospora sp. 1987 Li Hanmin et al., p. 117-118
Colatisporites descotus 1987 Li Hanmin et al., p. 117-118
Colatisporites sp. 1987 Li Hanmin et al., p. 117-118
Convolutispora sp. 1987 Li Hanmin et al., p. 117-118
Crassispora catenata 1987 Li Hanmin et al., p. 117-118
Crassispora trychera 1987 Li Hanmin et al., p. 117-118
Cristatisporites sp. 1987 Li Hanmin et al., p. 117-118
Dictyotriletes sp. 1987 Li Hanmin et al., p. 117-118
Dictyotriletes submarginatus 1987 Li Hanmin et al., p. 117-118
Dictyotriletes trivialis 1987 Li Hanmin et al., p. 117-118
Foveolatisporites sp. 1987 Li Hanmin et al., p. 117-118
Grandispora echinata 1987 Li Hanmin et al., p. 117-118
Grandispora lepata 1987 Li Hanmin et al., p. 117-118
Grandispora sp. 1987 Li Hanmin et al., p. 117-118
Hymenospora sp. 1987 Li Hanmin et al., p. 117-118
Knoxisporites literatus 1987 Li Hanmin et al., p. 117-118
Knoxisporites sp. 1987 Li Hanmin et al., p. 117-118
Krauselisporites? *hibernicus* 1987 Li Hanmin et al., p. 117-118
Leiotriletes sp. 1987 Li Hanmin et al., p. 117-118
Punctatisporites irrasus 1987 Li Hanmin et al., p. 117-118
Punctatisporites sp. 1987 Li Hanmin et al., p. 117-118
Raistrickia clavata 1987 Li Hanmin et al., p. 117-118
Raistrickia corynoges 1987 Li Hanmin et al., p. 117-118
Raistrickia sp. 1987 Li Hanmin et al., p. 117-118
Rugospora cf. *flecuosa* 1987 Li Hanmin et al., p. 117-118
Rugospora polyptycha 1987 Li Hanmin et al., p. 117-118
Rugospora sp. 1987 Li Hanmin et al., p. 117-118
Spelaeotriletes pretiosus 1987 Li Hanmin et al., p. 117-118

Spelaeotriletes resolutus 1987 Li Hanmin et al., p. 117-118
Tumulispora raritUberculata 1987 Li Hanmin et al., p. 117-118
Umbonatisporites distinctus 1987 Li Hanmin et al., p. 117-118
Vallatisporites vallatus 1987 Li Hanmin et al., p. 117-118
Verrucosisporites sp. 1987 Li Hanmin et al., p. 117-118

6. Leigutai Section, Longtan Town, Nanjing City: Upper Leigutai Member of Wutung Formation

Corbulispora cancellata 1988 Cai Chongyang et al., p. 175
Crassispora sp.A 1988 Cai Chongyang et al., p. 175
Crassispora sp.B 1988 Cai Chongyang et al., p. 175
Crassispora trychera 1988 Cai Chongyang et al., p. 175
Cymbosporites conatus 1988 Cai Chongyang et al., p. 175
Densosporites gracilis 1988 Cai Chongyang et al., p. 175
Dictyotriletes trivialis 1988 Cai Chongyang et al., p. 175
Foveosporites appositus 1988 Cai Chongyang et al., p. 175
Hymenozonotriletes sp. 1988 Cai Chongyang et al., p. 175
Knoxisporites literatus 1988 Cai Chongyang et al., p. 175
Laevigatosporites vulgaris 1988 Cai Chongyang et al., p. 175
Lophotriletes minutissimus 1988 Cai Chongyang et al., p. 175
Lycospora flexuosa 1988 Cai Chongyang et al., p. 175
Lycospora noctuina 1988 Cai Chongyang et al., p. 175
Retusotriletes avonensis 1988 Cai Chongyang et al., p. 175
Retusotriletes incohatus 1988 Cai Chongyang et al., p. 175

7. A cement factory, Dingshu Town, Yixing City: Wutung Formation

Anaplanisporites atheticus 1988 Cai Chongyang et al., p. 183
Apiciliretusispora multiseta 1988 Cai Chongyang et al., p. 183
Auroraspora macra 1988 Cai Chongyang et al., p. 183
Calamospora cf. *nigrata* 1988 Cai Chongyang et al., p. 183
Colatisporites decorus 1988 Cai Chongyang et al., p. 183
Convolutispora oppressa 1988 Cai Chongyang et al., p. 183
Corbulispora cancellata 1988 Cai Chongyang et al., p. 183
Crassispora parva 1988 Cai Chongyang et al., p. 183
Densosporites spinifer 1988 Cai Chongyang et al., p. 183
Dibolisporites distinctus 1988 Cai Chongyang et al., p. 183
Dictyotriletes rotundatus 1988 Cai Chongyang et al., p. 183
Grandispora echineta 1988 Cai Chongyang et al., p. 183
Knoxisporites literatus 1988 Cai Chongyang et al., p. 183
Punctatisporites cf. *pseudopunctatus* 1988 Cai Chongyang et al., p. 183
Punctatisporites irrasus 1988 Cai Chongyang et al., p. 183
Spelaeotriletes cf. *minutus* 1988 Cai Chongyang et al., p. 183
Spelaeotriletes exiguus 1988 Cai Chongyang et al., p. 183

Hubei

Changyang: Changyang Formation

Ancyrospora macra Sullivan, 1964 1992 Gao Lianda, pl. III, 10-11
Baculatisporites fusticulus Sullivan, 1964 1992 Gao Lianda, pl. I, 14
Crassispora trychera Neves et Ioannides, 1974 1992 Gao Lianda, pl. I, 10
Tumulispora dentate (Hughus et Playford) Turnua 1975 1992 Gao Lianda, pl. III, 3

Guizhou

Baihupo Section, Jialaohe Section, Qilinzhai Reservior Section, and Wuliqiao Section, Dushan County; Ganzhai Section, Pingtang County: Tangbagou Formation

Anaplanisporites atheticus 1991 Gao Lianda, p.62
Auroraspora macra 1991 Gao Lianda, p.62
Colatisporites decorus 1991 Gao Lianda, p.62
Colatisporites denticulatus 1991 Gao Lianda, p.62
Crassispora trychera 1991 Gao Lianda, p.62
Dictyotriletes trivialis 1991 Gao Lianda, p.62
Foveolatisporites guizhouensis 1991 Gao Lianda, p.62
Knoxisporites literatus 1991 Gao Lianda, p.62
Lycospora pusilla 1991 Gao Lianda, p.62
Raistrickia variabilis 1991 Gao Lianda, p.62
Rotaspora knox 1991 Gao Lianda, p.62
Spelaeotriletes crustatus 1991 Gao Lianda, p.62
Spelaeotriletes obtusus 1991 Gao Lianda, p.62
Spelaeotriletes pretiosus (Playford) Neves et Beit 1991 Gao Lianda, p.62
Tricidarisporites fasciculatus 1991 Gao Lianda, p.62
Tumulispora ordinaria 1991 Gao Lianda, p.62
Tumulispora rarituberculata 1991 Gao Lianda, p.62
Tumulispora rariverruata 1991 Gao Lianda, p.62
Umbonatisporites distinctus 1991 Gao Lianda, p.62

Late Tournaisian

Guizhou

1. **Baihupo Section, Jialaohe Section, Qilinzhai Reservior Section, and Wuliqiao Section, Dushan County; Ganzhai Section, Pingtang County: Tangbagou Formation**

Anaplanisporites atheticus 1991 Gao Lianda, p.62
Auroraspora macra 1991 Gao Lianda, p.62
Colatisporites decorus 1991 Gao Lianda, p.62
Colatisporites denticulatus 1991 Gao Lianda, p.62
Crassispora trychera 1991 Gao Lianda, p.62
Dictyotriletes trivialis 1991 Gao Lianda, p.62

Foveolatisporites guizhouensis 1991 Gao Lianda, p.62
Knoxisporites literatus 1991 Gao Lianda, p.62
Lycospora pusilla 1991 Gao Lianda, p.62
Raistrickia variabilis 1991 Gao Lianda, p.62
Rotaspora knox 1991 Gao Lianda, p.62
Spelaeotriletes crustatus 1991 Gao Lianda, p.62
Spelaeotriletes obtusus 1991 Gao Lianda, p.62
Spelaeotriletes pretiosus (Playford) Neves et Beit 1991 Gao Lianda, p.62
Tricidarisporites fasciculatus 1991 Gao Lianda, p.62
Tumulispora ordinaria 1991 Gao Lianda, p.62
Tumulispora barituberculata 1991 Gao Lianda, p.62
Tumulispora rariverruata 1991 Gao Lianda, p.62
Umbonatisporites distinctus 1991 Gao Lianda, p.62

2. Muhua Section: Lower Dawuba Formation

Acanthotriletes parispinosus (Luber) Gao, 1985 1985 Gao Lianda, pl. III, 19
Acanthotriletes socraticus Neves et Ioannides, 1974 1985 Gao Lianda, pl. III, 20
Anapiculatisporites concinnus Playford, 1963 1985 Gao Lianda, pl. III, 16
Anapiculatisporites hystricosus Playford, 1964 1985 Gao Lianda, pl. III, 18
Anapiculatisporites sp. 1985 Gao Lianda, pl. III, 17
Apiculiretusispora granulata Owens, 1971 1985 Gao Lianda, pl. III, 10
Archaeozonotriletes polymorphus Naumova, 1953 1985 Gao Lianda, pl. VII, 1
Auroraspora macra Sullivan, 1968 1985 Gao Lianda, pl. VI, 19-20
Calamospora nigrata (Naumova) Allen, 1965 1985 Gao Lianda, pl. III, 7
Convolutispora mellita Hoffmeister, Staplin et Malloy, 1955 1985 Gao Lianda, pls. IV, 26; X, 3
Convolutispora venusta Hoffmeister, Staplin et Malloy, 1955 1985 Gao Lianda, pls. IV, 21-23; 10, 4-5
Convolutispora verriformis Hughes et Playford, 1961 1985 Gao Lianda, pl. IV, 25
Corbulispora cancellata (Waltz) Bharadwaj et Butterworth 1961 1985 Gao Lianda, pl. V, 5
Crassispora maculosa (Knox) Sullivan, 1964 1985 Gao Lianda, pl. VI, 6
Crassispora trychera Neves et Ioannides, 1974 1985 Gao Lianda, pl. VI, 7-8
Cristatisporites echinatus Playford, 1963 1985 Gao Lianda, pl. VIII, 13
Cristatisporites sp. 1985 Gao Lianda, pl. VIII, 12
Cyclogranisporites palaeophytus Neves et Ioannides, 1974 1985 Gao Lianda, pl. III, 12
Densosporites anulatus (Loose) Smith et Butterworth, 1967 1985 Gao Lianda, pl. VII, 3
Densosporites spitsbergensis Playford, 1963 1985 Gao Lianda, pl. VII, 2
Diatomozonotriletes pecticatus Gao, 1983 1985 Gao Lianda, pl. VI, 18
Dictyotriletes crassipterus Naumova in Kedo, 1963 1985 Gao Lianda, pl. V, 9
Dictyotriletes submarginatus Playford, 1963 1985 Gao Lianda, pls. V, 10-11; 10, 7
Diducites poljessicus (Kedo) Vanveen 1980 1985 Gao Lianda, pl. VIII, 15-16
Discernisporites micromanifestus (Hacquebard) Sabry et Neves, 1971 1985 Gao Lianda, pl. VIII, 17-19
Endoculeospora grazinskii Turnau, 1975 1985 Gao Lianda, pl. VIII, 14
Geminospora sp. 1985 Gao Lianda, pl. VI, 22
Grandispora microgranulata Gao, 1985 1985 Gao Lianda, pl. IX, 2
Grandispora sp. 1985 Gao Lianda, pl. VIII, 11

Grandispora trichacanthusa (Luber) Gao, 1985 1985 Gao Lianda, pl. IX, 3
Knoxisporites hederatus (Ischenko) Playford, 1963 1985 Gao Lianda, pl. V, 14
Knoxisporites literatus (Waltz) Playford, 1963 1985 Gao Lianda, pl. V, 15-16
Knoxisporites pristicus Sullivan, 1968 1985 Gao Lianda, pl. V, 12
Knoxisporites sp. 1985 Gao Lianda, pl. V, 13
Laevigatosporites vulgaris (Ibrahim) Alpern et Doubinger, 1973 1985 Gao Lianda, pl. IX, 17
Leiotriletes notus Ischenko 1952 1985 Gao Lianda, pl. III, 1
Lophozonotriletes circumcriptus Ischenko 1956 1985 Gao Lianda, pl. V, 22
Lophozonotriletes obsoletus Kedo, 1963 1985 Gao Lianda, pl. V, 21
Lycospora pusilla (Ibrahim) Somers 1972 1985 Gao Lianda, pls. VI, 2; X, 8
Monilospora triungensis Playford, 1963 1985 Gao Lianda, pl. VII, 20
Murospora conduplicata (Andrejeva) Playford, 1963 1985 Gao Lianda, pl. VI, 14
Murospora minuta Gao, 1985 1985 Gao Lianda, pl. VI, 16-17
Murospora sp. 1985 Gao Lianda, pl. VI, 15
Orbisporis muricatus Bharadwaj et Venkatachala 1961 1985 Gao Lianda, pl. V, 18
Punctatisporites pseudolevatus Hoffmeister, Staplin et Malloy, 1955 1985 Gao Lianda, pl. III, 3
Punctatisporites punctulus (Kedo) Gao, 1985 1985 Gao Lianda, pl. III, 2
Punctatisporites sp. 1985 Gao Lianda, pl. III, 4
Raistrickia condycosa Higgs, 1975 1985 Gao Lianda, pl. III, 24
Raistrickia multipertica Hoffmeister, Staplin et Malloy, 1955 1985 Gao Lianda, pl. IV, 2
Raistrickia sp. 1985 Gao Lianda, pl. III, 22
Raistrickia variabilis Dolby et Naves, 1970 1985 Gao Lianda, pl. III, 23
Reticulatisporites macroreticulatus (Naumova) Gao, 1985 1985 Gao Lianda, pl. V, 3
Reticulatisporites similis (Kedo) Gao, 1985 1985 Gao Lianda, pls. IV, 27; V, 1-2
Rotaspora sp. 1985 Gao Lianda, pl. VI, 21
Spelaeotriletes arenaceus Neves et Owens, 1966 1985 Gao Lianda, pl. VIII, 8
Stenozonotriletes conformis Naumova, 1953 1985 Gao Lianda, pl. VI, 4
Stenozonotriletes rasilis Kedo, 1963 1985 Gao Lianda, pl. VI, 3
Tricidarisporites fasciculatus (Love) Sullivan et Marshall, 1966 1985 Gao Lianda, pl. IV, 19-20
Tumulispora malevkensis (Kedo) Turnau, 1978 1985 Gao Lianda, pls. VII, 15; VIII, 1
Tumulispora rarituberculata (Luber) Potonié, 1966 1985 Gao Lianda, pl. VII, 8-10
Tumulispora variverrucata (Playford) Staplin et Jansonius, 1964 1985 Gao Lianda, pl. VII, 7
Umbonatisporites distinctus Clayton, 1970 1985 Gao Lianda, pl. IV, 5-7
Umbonatisporites sp. 1985 Gao Lianda, pl. IV, 8
Verrucosisporites cerosus (Hoffmeister, Staplin et Malloy) Butterworth et Williams, 1958 1985 Gao Lianda, pl. IV, 17
Verrucosisporites macrotuberculatus (Kedo) Gao, 1985 1985 Gao Lianda, pl. IV, 13
Verrucosisporites microtuberosus (Loose) Smith et Butterworth, 1967 1985 Gao Lianda, pl. IV, 16
Verrucosisporites nitidus (Naumova) Playford, 1964 1985 Gao Lianda, pl. IV, 11, 14
Verrucosisporites rufus Butterworth et Williams, 1958 1985 Gao Lianda, pl. IV, 12, 15

Visean

Guangdong

Shuangxia Section, Xingning City: Dahu Formation

Acanthotriletes cf. *infonsus* 2002 Li Wenhui, tab. 1
Acanthotriletes intonsus 2002 Li Wenhui, tab. 1
Acanthotriletes sp. 2002 Li Wenhui, tab. 1
Anapiculatisporites cf. *concinnus* 2002 Li Wenhui, tab. 1
Anapiculatisporites minor 2002 Li Wenhui, tab. 1
Convolutispora cf. *venusta* 2002 Li Wenhui, tab. 1
Convolutispora venusta 2002 Li Wenhui, tab. 1
Cyclogranisporites cf. *orbiculus* 2002 Li Wenhui, tab. 1
Cyclogranisporites desus 2002 Li Wenhui, tab. 1
Cyclogranisporites orbiculus 2002 Li Wenhui, tab. 1
Dictyotriletes cf. *clatriformis* 2002 Li Wenhui, tab. 1
Dictyotriletes cf. *distortus* 2002 Li Wenhui, tab. 1
Dictyotriletes cf. *pactilis* 2002 Li Wenhui, tab. 1
Dictyotriletes cf. *submarginatus* 2002 Li Wenhui, tab. 1
Dictyotriletes clatriformis 2002 Li Wenhui, tab. 1
Dictyotriletes distortus 2002 Li Wenhui, tab. 1
Dictyotriletes pactilis 2002 Li Wenhui, tab. 1
Dictyotriletes submarginatus 2002 Li Wenhui, tab. 1
Granulatisporites minutus 2002 Li Wenhui, tab. 1
Leiotriletes cf. *notius* 2002 Li Wenhui, tab. 1
Leiotriletes cf. *tumidus* 2002 Li Wenhui, tab. 1
Leiotriletes notius 2002 Li Wenhui, tab. 1
Leiotriletes sp. 2002 Li Wenhui, tab. 1
Leiotriletes tumidus 2002 Li Wenhui, tab. 1
Lycospora noctuina 2002 Li Wenhui, tab. 1
Lycospora rotunda 2002 Li Wenhui, tab. 1
Procoronaspora ambigua 2002 Li Wenhui, tab. 1
Punctatisporites planus 2002 Li Wenhui, tab. 1
Punctatisporites sp. 2002 Li Wenhui, tab. 1
Stenozonotriletes sp. 2002 Li Wenhui, tab. 1
Strumiantrospora cf. *shuangfengensis* 2002 Li Wenhui, tab. 1
Strumiantrospora shuangfengensis 2002 Li Wenhui, tab. 1
Strumiantrospora tribulla 2002 Li Wenhui, tab. 1
Triguitrites auricukatus 2002 Li Wenhui, tab. 1
Triguitrites cf. *auricukatus* 2002 Li Wenhui, tab. 1
Triportites margintus 2002 Li Wenhui, tab. 1
Triguitrites cf. *tricuspis* 2002 Li Wenhui, tab. 1
Triguitrites tricuspis 2002 Li Wenhui, tab. 1
Triportites cf. *distinetus* 2002 Li Wenhui, tab. 1
Triportites distinetus 2002 Li Wenhui, tab. 1
Triportites sp. 2002 Li Wenhui, tab. 1
Verrucosisporites compactus 2002 Li Wenhui, tab. 1

Verrucosisporites sp. 2002 Li Wenhui, tab. 1

Shiduan Section, Meizhou City: Zhongxin Formation

Acanthotriletes intonsus 2002 Li Wenhui, tab. 1
Anapiculatisporites concinnus 2002 Li Wenhui, tab. 1
Anapiculatisporites minor 2002 Li Wenhui, tab. 1
Calamospora sp. 2002 Li Wenhui, tab. 1
Convolutispora cf. *venusta* 2002 Li Wenhui, tab. 1
Convolutispora flexuosa 2002 Li Wenhui, tab. 1
Convolutispora sp. 2002 Li Wenhui, tab. 1
Cyclogranisporites desus 2002 Li Wenhui, tab. 1
Dictyotriletes cf. *distortus* 2002 Li Wenhui, tab. 1
Dictyotriletes clatriformis 2002 Li Wenhui, tab. 1
Dictyotriletes pactilis 2002 Li Wenhui, tab. 1
Dictyotriletes submarginatus 2002 Li Wenhui, tab. 1
Granulatisporites minutus 2002 Li Wenhui, tab. 1
Leiotriletes cf. *notius* 2002 Li Wenhui, tab. 1
Leiotriletes cf. *tumidus* 2002 Li Wenhui, tab. 1
Leiotriletes notius 2002 Li Wenhui, tab. 1
Leiotriletes sp. 2002 Li Wenhui, tab. 1
Leiotriletes tumidus 2002 Li Wenhui, tab. 1
Lycospora cf. *noctuina* 2002 Li Wenhui, tab. 1
Lycospora pusilla 2002 Li Wenhui, tab. 1
Lycospora rotunda 2002 Li Wenhui, tab. 1
Punctatisporites planus 2002 Li Wenhui, tab. 1
Punctatisporites pseudolevatus 2002 Li Wenhui, tab. 1
Punctatisporites sp. 2002 Li Wenhui, tab. 1
Strumiantrospora sp. 2002 Li Wenhui, tab. 1
Strumiantrospora tribulla 2002 Li Wenhui, tab. 1
Triguitrites auricukatus 2002 Li Wenhui, tab. 1
Triguitrites tricuspis 2002 Li Wenhui, tab. 1
Triportites cf. *distinetus* 2002 Li Wenhui, tab. 1
Triportites distinetus 2002 Li Wenhui, tab. 1
Verrucosisporites compactus 2002 Li Wenhui, tab. 1

Hunan

1. **Ceshui, Yongfeng Town, Shuangfeng County; Chaoguang Coal Mine, Xiandong Village, Lianyuan City; Zhujiadang Section, Wentang Town, Xinhua County: Lower Ceshui Formation**

Acanthotriletes intonsus Playford 1986 Tang Shanyuan, pl.1, 12
Anapiculatisporites concinnus Playford 1986 Tang Shanyuan, pl.1, 15-17
Anapiculatisporites minor (B. et W.) S. et B. 1986 Tang Shanyuan, pl.1, 14
Apiculiretusispora setosa (Kedo) Gao 1986 Tang Shanyuan, pl.1, 34-35
Auroraspora macra Sullivan 1986 Tang Shanyuan, pl.2, 34-35

Calamospora sp. 1986 Tang Shanyuan, pl.1, 6
Converrucosisporites sp. 1986 Tang Shanyuan, pl.1, 10
Convolutispora flexuosa f. *minor* Hacquebard 1986 Tang Shanyuan, pl.1, 31
Convolutispora sp. 1986 Tang Shanyuan, pl.1, 29
Convolutispora venusta H. S. et M. 1986 Tang Shanyuan, pl.1, 30
Cyclogranisporites desus Bharadwaj 1986 Tang Shanyuan, pl.1, 8
Cyclogranisporites orbiculus Potonié et Kremp 1986 Tang Shanyuan, pl.1, 9
Densosporites braziliensis Pant et Sriv 1986 Tang Shanyuan, pl.2, 27
Dictyotriletes clatriformis (Artuez) Sullivan 1986 Tang Shanyuan, pl.1, 18
Dictyotriletes distortus Peppers 1986 Tang Shanyuan, pl.1, 19
Dictyotriletes pactilis Sullivan et Marshall 1986 Tang Shanyuan, pl.1, 20-21
Dictyotriletes submarginatus Playford 1986 Tang Shanyuan, pl.1, 22
Discernisporites micromanifestus (Hacquebard) Sabry et Neves 1986 Tang Shanyuan, pl.2, 31-32
Endosporites parvus Guennel 1986 Tang Shanyuan, pl.2, 30
Endosporites plicatus Kosanke 1986 Tang Shanyuan, pl.2, 29
Grandispora sp. 1986 Tang Shanyuan, pl.2, 36
Granulatisporites minutus Potonié et Kremp 1986 Tang Shanyuan, pl.1, 7
Heteroporispora arcus Tang, 1986 1986 Tang Shanyuan, pl.1, 47-48
Heteroporispora circinata Tang, 1986 1986 Tang Shanyuan, pl.1, 49-50
Heteroporispora compta Tang, 1986 1986 Tang Shanyuan, pl.2, 1
Heteroporispora deformis Tang, 1986 1986 Tang Shanyuan, pl.1, 45-46
Heteroporispora foveota Jiang, Hu et Tang 1986 Tang Shanyuan, pl.1, 52-54, 56-57
Heteroporispora ningyuanensis Jiang, Hu et Tang 1986 Tang Shanyuan, pl.1, 51, 55
Heteroporispora rectangularis Tang, 1986 1986 Tang Shanyuan, pl.1, 58-59
Heteroporispora? sp. 1986 Tang Shanyuan, pl.2, 2-4
Knoxisporites cf. *ruhlandi* Doubinger et Rausher 1986 Tang Shanyuan, pl.1, 44
Leiotriletes notius Hacquebard 1986 Tang Shanyuan, pl.1, 1
Leiotriletes tumidus Butterworth et Williams 1986 Tang Shanyuan, pl.1, 2
Lycospora noctuina Betterworth et Williams 1986 Tang Shanyuan, pl.1, 41
Lycospora pusilla (Ibr.) Somers 1986 Tang Shanyuan, pl.1, 42-43
Lycospora rotunda (Bharadwaj) Somers 1986 Tang Shanyuan, pl.1, 39-40
Microreticulatisporites lunatus Knox 1986 Tang Shanyuan, pl.1, 23-24
Murospora aurita (Waltz) Playford 1986 Tang Shanyuan, pl.2, 18-19
Murospora cf. *conduplicata* (Andr.) Playford 1986 Tang Shanyuan, pl.2, 5-6
Murospora cf. *intorta* (Waltz) Playford 1986 Tang Shanyuan, pl.2, 20
Murospora hunanensis Tang, 1986 1986 Tang Shanyuan, pl.2, 9-10
Murospora margodentata Beju 1986 Tang Shanyuan, pl.2, 7-8
Murospora sp. 2 1986 Tang Shanyuan, pl.2, 15
Murospora sp. 3 1986 Tang Shanyuan, pl.2, 16
Murospora sp. 4 1986 Tang Shanyuan, pl.2, 17
Murospora sp. 1 1986 Tang Shanyuan, pl.2, 11-14
Perotriletes sp. 1986 Tang Shanyuan, pl.2, 28
Planisporites sp. 1986 Tang Shanyuan, pl.1, 13
Procoronaspora ambigua (Butterworth et Williams) Smith et Butterworth 1986 Tang Shanyuan, pl.1, 32

Punctatisporites cf. *pseudolevatus* H. S. et M. 1986 Tang Shanyuan, pl.1, 4
Punctatisporites planus Hacquebard 1986 Tang Shanyuan, pl.1, 3
Punctatisporites sp. 1986 Tang Shanyuan, pl.1, 5
Rotaspora knoxi Butterworth et Williams 1986 Tang Shanyuan, pl.1, 38
Rotaspora sp. 1986 Tang Shanyuan, pl.1, 37
Spelaeotriletes cf. *balteatus* (Playford) Higgs 1986 Tang Shanyuan, pl.2, 33
Stenozonotriletes sp. 1986 Tang Shanyuan, pl.1, 36
Strumiantrospora shuangfengensis Tang, 1986 1986 Tang Shanyuan, pl.1, 27-28
Strumiantrospora tribulla Tang, 1986 1986 Tang Shanyuan, pl.1, 25-26
Tripartites distinctus Williams 1986 Tang Shanyuan, pl.2, 21
Tripartites sp. 1986 Tang Shanyuan, pl.2, 22
Triquitrites duriculatus Bharadwaj 1986 Tang Shanyuan, pl.2, 25
Triquitrites marginatus Hoffmeiste, Staplin et Malloy 1986 Tang Shanyuan, pl.2, 24
Triquitrites tendoris Hacquebard et Barss 1986 Tang Shanyuan, pl.2, 26
Triquitrites tricuspis (Horst) Potonié et Kremp 1986 Tang Shanyuan, pl.2, 23
Verrucosisporites compactus Habib 1986 Tang Shanyuan, pl.1, 11
Waltzispora? sp. 1986 Tang Shanyuan, pl.1, 33

2. Shetianqiao, Shaodong County; Lengshuipu Coal Mine, Lengshui Town, Ningyuan County; Daquan Village, Shaodong County: Shidengzi Member of Datang Stage

Acanthotriletes sp. 1984 Jiang et Hu, p.43
Apiculatasporites sp. 1984 Jiang et Hu, p.43
Bellispores sp. 1982 Jiang et Hu, pl.396, 17, 23-26
Camptotriletes cf. *corrugatus* (Ibrahim) Potonié et Kremp 1982 Jiang et Hu, pl.396, 19
Cyclogranisporites sp. 1984 Jiang et Hu, p.43
Densosporites lori Bharadwaj 1982 Jiang et Hu, pl.396, 14-16
Dictyotriletes minor Jiang et Hu 1984 Jiang et Hu, p.43
Granulatisporites minutus Potonié et Kremp 1984 Jiang et Hu, p.43
Granulatisporites ningyuanensis Jiang et Hu 1984 Jiang et Hu, p.43
Heteroporispora foveota Jiang et Hu et Tang, 1982 1982 Jiang et Hu, pl.396, 27-31
Lepidozonotriletes tersus Jiang et Hu, 1982 1982 Jiang et Hu, pl.396, 18, 20-22
Lophozonotriletes sp. 1984 Jiang et Hu, p.43
Lycospora brevis Bharadwaj 1982 Jiang et Hu, pl.396, 10
Lycospora granulata Ibrahim 1984 Jiang et Hu, p.43
Murospora sp. 1984 Jiang et Hu, p.43
Punctatisporites inconspieus Jiang et Hu, 1982 1982 Jiang et Hu, pl.395, 25-27
Savitrisporites minor Jiang et Hu, 1982 1982 Jiang et Hu, pl.396, 5-9
Savitrisporites sp. 1982 Jiang et Hu, pl.396, 11
Simozonotriletes robustus Jiang et Hu, 1982 1982 Jiang et Hu, pl.396, 32
Stenozonotriletes minor Jiang et Hu, 1982 1982 Jiang et Hu, pl.396, 12-13
Stenozonotriletes sp. 1984 Jiang et Hu, p.43
Tripartites hunanensis Jiang et Hu, 1982 1982 Jiang et Hu, pl.396, 33, 35-38
Tripartites sp. 1 1982 Jiang et Hu, pl.396, 34
Tripartites sp. 2 1982 Jiang et Hu, pl.396, 39

3. Lengshuipu Coal Mine, Lengshui Town, Ningyuan County; Daquan Village, Shaodong County: Ceshui Formation of Datang Stage

Acanthotriletes sp. 1984 Jiang et Hu, p.43
Apiculatasporites sp. 1984 Jiang et Hu, p.43
Camptotriletes cf. *corrugatus* (Ibrahim) Potonié et Kremp 1982 Jiang et Hu, pl.395, 9-10
Camptotriletes corrugatus Potonié et Kremp 1984 Jiang et Hu, p.43
Cyclogranisporites sp. 1984 Jiang et Hu, p.43
Densosporites sp. 1984 Jiang et Hu, p.43
Diatomozonotriletes ningyuanensis Jiang et Hu 1984 Jiang et Hu, p.43
Dictyotriletes minor Jiang et Hu, 1982 1982 Jiang et Hu, pl.395, 11-12
Foveosporites sp. 1984 Jiang et Hu, p.43
Granulatisporites minutus Potonié et Kremp 1982 Jiang et Hu, pl.396, 1-4
Granulatisporites ningyuanensis Jiang et Hu, 1982 1982 Jiang et Hu, pl.395, 2-5
Heteroporispora foveota Jiang et Hu et Tang, 1982 1982 Jiang et Hu, pl.395, 13, 20-24
Heteroporispora ningyuanensis Jiang, Hu et Tang, 1982 1982 Jiang et Hu, pl.395, 15-19
Lepidozonotriletes tersus Jiang et Hu, 1982 1982 Jiang et Hu, pl.395, 6-8
Lophozonotriletes sp. 1984 Jiang et Hu, p.43
Lycospora brevis Bharadwaj 1982 Jiang et Hu, pl.395, 1
Lycospora granulata Ibrahim 1984 Jiang et Hu, p.43
Murospora sp. 1984 Jiang et Hu, p.43
Punctatisporites inconspieus Jiang et Hu 1984 Jiang et Hu, p.43
Remysporites sp. 1984 Jiang et Hu, p.43
Savitrisporites minor Jiang et Hu 1984 Jiang et Hu, p.43
Simozonotriletes robustus Jiang et Hu, 1982 1982 Jiang et Hu, pl.395, 35-37
Simozonotriletes sp.1 1982 Jiang et Hu, pl.395, 28
Simozonotriletes sp.2 1982 Jiang et Hu, pl.395, 29
Sporopollenites sp. 1984 Jiang et Hu, p.43
Stenozonotriletes minor Jiang et Hu 1984 Jiang et Hu, p.43
Stenozonotriletes sp.1 1982 Jiang et Hu, pl.395, 14
Tripartites hunanensis Jiang et Hu, 1982 1982 Jiang et Hu, pl.395, 31-34
Triquitrites sp.3 1982 Jiang et Hu, pl.395, 30

Jiangsu

Baojia Village, Jurong County: Gaolishan Formation

Anapiculatisporites epicharis Ouyang et Chen, 1987 1987a Ouyang Shu et Chen Yongxiang, pl.8, 20
Anapiculatisporites juyongensis Ouyang et Chen, 1987 1987a Ouyang Shu et Chen Yongxiang, pl.6, 2-4
Apiculatisporis pineatus H., S. et M., 1955 1987a Ouyang Shu et Chen Yongxiang, pl.5, 14-18
Calamospora cf. *membrana* Bharadwaj, 1957 1987a Ouyang Shu et Chen Yongxiang, pl.2, 19-20
Calamospora cf. *pedata* Kosanke, 1950 1987a Ouyang Shu et Chen Yongxiang, pl.2, 22, 24
Calamospora exigua Staplin, 1960 1987a Ouyang Shu et Chen Yongxiang, pl.2, 16-18
Calamospora pallida (Loose) S., W. et B., 1944 1987a Ouyang Shu et Chen Yongxiang, pl.2, 11-12
Calamospora parva Guennel, 1958 1987a Ouyang Shu et Chen Yongxiang, pl.2, 14-15

Calamospora unisofissus Ouyang et Chen, 1987 1987a Ouyang Shu et Chen Yongxiang, pl.2, 21, 23
Colatisporites subgranulatus Ouyang et Chen, 1987 1987a Ouyang Shu et Chen Yongxiang, pl.16, 17, 20
Crassispora tuberculiformis Ouyang et Chen, 1987 1987a Ouyang Shu et Chen Yongxiang, pl.16, 1-3; pl.13, 6
Cyclogranisporites areolatus Ouyang et Chen, 1987 1987a Ouyang Shu et Chen Yongxiang, pl.4, 6-7
Cyclogranisporites cf. *micaceus* (Imgr.) Potonié et Kremp, 1955 1987a Ouyang Shu et Chen Yongxiang, pl.3, 18
Cyclogranisporites microgranus Bharadwaj, 1957 1987a Ouyang Shu et Chen Yongxiang, pl.3, 19, 22, 23, 24
Cyclogranisporites pisticus Playford, 1978 1987a Ouyang Shu et Chen Yongxiang, pl.3, 15-17
Dibolisporites distinctus (Clayton) Playford, 1976 1987a Ouyang Shu et Chen Yongxiang, pl.7, 6a-b
Hymenospora cf. *H. caperata* Felix et Burbridge, 1967 1987a Ouyang Shu et Chen Yongxiang, pl.13, 14, 15, 22
Lagenicula applicita Chen et Ouyang, 1987 1987 Chen Yongxiang et Ouyang Shu, pl.1, 8; pl.2, 7
Leiotriletes prominulus Ouyang et Chen, 1987 1987a Ouyang Shu et Chen Yongxiang, pl.1, 21-22
Lycospora denticulata Bharadwaj, 1957 1987a Ouyang Shu et Chen Yongxiang, pl.15, 8-11
Lycospora tenuispinosa Ouyang et Chen, 1987 1987a Ouyang Shu et Chen Yongxiang, pl.15, 1-4
Phyllothecotriletes rigidus Playford, 1962 1987a Ouyang Shu et Chen Yongxiang, pl.2, 10, 13
Punctatisporites anisoletus Ouyang et Chen, 1987 1987a Ouyang Shu et Chen Yongxiang, pl.2, 6-8; pl.4, 1
Rugospora arenacea Ouyang et Chen, 1987 1987a Ouyang Shu et Chen Yongxiang, pl.17, 12
Sublagenicula nudoides Chen et Ou, 1987 1987 Chen Yongxiang et Ouyang Shu, pl.1, 1-3
Velamisporites cf. *vermiculatus* Felix et Burbridge, 1967 1987a Ouyang Shu et Chen Yongxiang, pl.11, 4-5; pl.12, 1
Velamisporites perinatus (Hughes et Playford) Playford, 1971 1987a Ouyang Shu et Chen Yongxiang, pl.12, 12
Velamisporites rugosus Bharad. et Venkat., 1962 1987a Ouyang Shu et Chen Yongxiang, pl.11, 6; pl.12, 2-4
Velamisporites verrucosus Ouyang et Chen, 1987 1987a Ouyang Shu et Chen Yongxiang, pl.14, 19-20

Fujian

1. Niukenggou Section, Pojiao Village, Changting County: Lower Zishan Formation

Acanthotriletes multisetus (Luber) Pot. et Kr. 1982 Huang Xinyu, pl.1, 9
Acanthotriletes sp. 1982 Huang Xinyu, pl.1, 10-11
Anapiculatisporites dumosus (Staplin) Huang, 1982 1982 Huang Xinyu, pl.1, 8
Calamospora exigua Staplin 1982 Huang Xinyu, pl.1, 4
Changtingispora pulchra Huang, 1982 1982 Huang Xinyu, pl.2, 3-4
Changtingispora simplex Huang, 1982 1982 Huang Xinyu, pl.2, 1-2
Cirratriradites sp. 1982 Huang Xinyu, pl.2, 34
Convolutispora sp. 1982 Huang Xinyu, pl.1, 7
Densosporites dentatus (Waltz) Pot. et Kr. 1982 Huang Xinyu, pl.2, 30
Densosporites loricatus (Loose) S. W. et B. 1982 Huang Xinyu, pl.2, 33
Densosporites sp. 1982 Huang Xinyu, pl.2, 31-32
Diatomozonotriletes? sp. 1982 Huang Xinyu, pl.2, 29
Dictyotriletes minutus Huang, 1982 1982 Huang Xinyu, pl.1, 16-17
Distalanulisporites subtriangulus Huang, 1982 1982 Huang Xinyu, pl.2, 14-15
Endosporites? sp. 1982 Huang Xinyu, pl.2, 35
Foveolatisporites sp. 1982 Huang Xinyu, pl.1, 19
Granulatisporites granulatus Ibr. 1982 Huang Xinyu, pl.1, 6
Granulatisporites parvigranulatus Staplin 1982 Huang Xinyu, pl.1, 5
Knoxisporites sp. 1982 Huang Xinyu, pl.1, 20

Laevigatosporites sp. 1982 Huang Xinyu, pl.2, 36-37
Leiotriletes sp. 1982 Huang Xinyu, pl.1, 1
Leiotriletes sphaerotriangulus (Loose) Pot. et Kr. 1982 Huang Xinyu, pl.1, 2
Lophozonotriletes raritiberculatus (Luber) Kedo 1982 Huang Xinyu, pl.2, 17
Lophozonotriletes? *appendices* (Hacquebard et Barss) Playford 1982 Huang Xinyu, pl.2, 16
Lycospora granulata Kos. 1982 Huang Xinyu, pl.2, 7-8
Lycospora lobulata Staplin 1982 Huang Xinyu, pl.2, 6
Lycospora micrograndulata Bhardwaj. 1982 Huang Xinyu, pl.2, 5
Lycospora orbicula (Pot. et Kr.) Smith. et Butt. 1982 Huang Xinyu, pl.2, 12-13
Lycospora pallucida (Wich.) S. W. et B. 1982 Huang Xinyu, pl.2, 11
Lycospora verrucosa Kos. 1982 Huang Xinyu, pl.2, 9-10
Microreticulatisporites fistulosus (Ibr.) K. MS. 1982 Huang Xinyu, pl.1, 15
Microreticulatisporites parvirugosus Staplin 1982 Huang Xinyu, pl.1, 14
Murospora sp. 1982 Huang Xinyu, pl.2, 26-27
Murospora varia Staplin 1982 Huang Xinyu, pl.2, 24-25
Punctatisporites sp. 1982 Huang Xinyu, pl.1, 3
Raistrickia bacilla Huang, 1982 1982 Huang Xinyu, pl.1, 12-13
Reticulatisporites sp. 1982 Huang Xinyu, pl.1, 18
Simozonotriletes intortus (Waltz) Pot. et Kr. 1982 Huang Xinyu, pl.2, 23
Simozonotriletes pijiaoensis Huang, 1982 1982 Huang Xinyu, pl.2, 18-22
Stenozonotriletes clarus Ischenko 1982 Huang Xinyu, pl.2, 28
Tripartites complanatus Staplin 1982 Huang Xinyu, pl.1, 35
Tripartites incisotrilobus (Naumova) R. Pot. 1982 Huang Xinyu, pl.1, 38
Tripartites paradoxus Huang, 1982 1982 Huang Xinyu, pl.1, 36-37
Tripartites serratus Staplin 1982 Huang Xinyu, pl.1, 32-33
Tripartites trilinguis (Horst.) Pot. et Kremp 1982 Huang Xinyu, pl.1, 31
Tripartites vetustus Schemel 1982 Huang Xinyu, pl.1, 34
Triquitrites cf. *simplex* Dybova. et Jach. 1982 Huang Xinyu, pl.1, 26
Triquitrites ornatus Dybova. et Jach 1982 Huang Xinyu, pl.1, 23
Triquitrites tendoris Hacq. et Barss 1982 Huang Xinyu, pl.1, 25
Triquitrites tribullatus (Ibr.) S. W. et B. 1982 Huang Xinyu, pl.1, 27
Waltzispora albertensis Staplin 1982 Huang Xinyu, pl.1, 28-29
Waltzispora sagittata Playford 1982 Huang Xinyu, pl.1, 30

2. Xiangliao Village, Chishui Town, Zhangping City: Lower–Middle Lindi Group

Acanthotriletes sp. 1982 Wu et Zhao, p. 143
Densosporites sp. 1982 Wu et Zhao, p. 143
Granulatisporites sp. 1982 Wu et Zhao, p. 143
Lycospora sp. 1982 Wu et Zhao, p. 143
Murospora sp. 1982 Wu et Zhao, p. 143
Triquitrites sp. 1982 Wu et Zhao, p. 143

Guizhou

1. **Wudang Section, Guiyang City; Jialaohe Section, Qilinzhai Reservior Section, Dushan County; Xiguan Section, Pingtang County: Jiusi Formation**

Acanthotriletes tenuispinosus Kedo, 1963 1983b Gao Lianda, pl.114, 19
Anapiculatisporites minor Butterworth et Williams, 1958 1983b Gao Lianda, pl.114, 22, 24
Anaplanisporites atheticus Neves et Ioannides, 1974 1983b Gao Lianda, pl.114, 23, 25
Apiculiretusispora microgranulata Gao, 1983 1983b Gao Lianda, pl.114, 17
Apiculiretusispora setosa (Kedo) Gao, 1983 1983b Gao Lianda, pl.114, 14-16
Auroraspora solisortus Hoffmeister, Staplin et Malloy, 1955 1983b Gao Lianda, pl.116, 23
Calamospora breviradiata Kosanke, 1950 1983b Gao Lianda, pl.114, 4; pl.117, 1
Calamospora flexilis Kosanke, 1950 1983b Gao Lianda, pl.114, 3
Cirratriradites cf. *saturni* (Ibrahim) Schopf, Wilson et Bentall, 1944 1983b Gao Lianda, pl.116, 4
Cirratriradites saturni 1991 Gao Lianda, p.62
Colatisporites denticulatus Neville, 1974 1983b Gao Lianda, pl.116, 1-2
Convolutispora venusta Hoffmeister, Staplin et Malloy, 1955 1984 Gao Lianda, pl.136, 14-15; 1983b Gao Lianda, pl.114, 29-30
Crassispora maculosa 1991 Gao Lianda, p.62
Cyclogranisporites lasius (Waltz) Playford, 1962 1983b Gao Lianda, pl.114, 12
Densosporites anulatus (Loose) Schopf, Wilson et Bentall, 1944 1983b Gao Lianda, pl.115, 31
Densosporites parvus Hoffmeister, Staplin et Malloy, 1955 1983b Gao Lianda, pl.115, 30
Diatomozonotriletes jubatus (Staplin) Gao, 1983 1983b Gao Lianda, pl.115, 25
Diatomozonotriletes minutus Gao, 1983 1983b Gao Lianda, pl.115, 22
Diatomozonotriletes papillatus Gao, 1983 1983b Gao Lianda, pl.115, 26-27
Diatomozonotriletes pectinatus Gao, 1983 1983b Gao Lianda, pl.115, 21
Diatomozonotriletes sp. 1 1983b Gao Lianda, pl.115, 28
Diatomozonotriletes sp. 2 1983b Gao Lianda, pl.115, 29
Diatomozonotriletes subspeciosus Gao, 1983 1983b Gao Lianda, pl.115, 20, 24
Diatomozonotriletes ubertus Ischenko, 1958 1983b Gao Lianda, pl.115, 23
Diatomozonotriletes cf. *rarus* 1991 Gao Lianda, p.62
Dictyotriletes sp.2 1983b Gao Lianda, pl.114, 31
Discernisporites micromanifestus 1991 Gao Lianda, p.62
Discernisporites cf. *micromanifestus* (Hacquebard) Sabry et Neves, 1967 1983b Gao Lianda, pl.116, 21
Endosporites hyalinus hyalinus (Naumova) Gao, 1983 1983b Gao Lianda, pl.116, 18-19
Endosporites hyalinus tournensis (Kedo) Gao, 1983 1983b Gao Lianda, pl.116, 16-17
Foveolatisporites sp. 1983b Gao Lianda, pl.114, 32
Foveolatisporites triangulatus Gao, 1983 1983b Gao Lianda, pl.114, 33-34
Grandispora sp.2 1983b Gao Lianda, pl.116, 20
Granulatisporites granulatus Ibrahim, 1933 1983b Gao Lianda, pl.114, 21
Granulatisporites normalis (Naumova) Gao, 1983 1983b Gao Lianda, pl.114, 18
Hadrohercos minutus Gao, 1983 1983b Gao Lianda, pl.115, 32
Knoxisporites pristinus 1991 Gao Lianda, p.62
Knoxisporites seniradiatus Neves 1964 1983b Gao Lianda, pl.114, 11
Laevigatosporites vulgaris (Ibrahim) Alpern et Doubinger, 1973 1983b Gao Lianda, pl.116, 28-29
Leiotriletes sp.2 1983b Gao Lianda, pl.114, 2
Leiotriletes sphaerotriangularis (Loose) Potonié et Kremp, 1954 1983b Gao Lianda, pl.114, 1

Lycospora granianellatus Staplin, 1960 1983b Gao Lianda, pl.115, 5-6
Lycospora granulata Kosanke, 1950 1983b Gao Lianda, pl.115, 7-8
Lycospora pusilla (Ibrahim) Schopf, Wilson et Bentall, 1944 1983b Gao Lianda, pl.115, 3-4
Lycospora sp. 1983b Gao Lianda, pl.115, 2
Monilospora mutabilis Staplin, 1960 1983b Gao Lianda, pl.116, 5-8
Murospora canduplicata (Andre) Playford 1991 Gao Lianda, p.62
Murospora canduplicata (Andrejeva) Playford, 1963 1983b Gao Lianda, pl.116, 9-10, 12
Murospora mutabilis Staplin 1991 Gao Lianda, p.62
Orbisporis sp. 1983b Gao Lianda, pl.115, 11
Punctatisporites lacunosus maximus Gao, 1983 1983b Gao Lianda, pl.114, 5-6
Punctatisporites punctatus Ibrahim, 1933 1983b Gao Lianda, pl.114, 8
Punctatisporites punctulus (Kedo) Gao, 1983 1983b Gao Lianda, pl.114, 9
Punctatisporites solidus (Naumova) Gao et Hou, 1975 1983b Gao Lianda, pl.114, 7
Punctatosporites sp. 1983b Gao Lianda, pl.116, 30
Raistrickia subrotundata (Kedo) Gao, 1983 1983b Gao Lianda, pl.114, 20
Reinschospora speciosa 1991 Gao Lianda, p.62
Reticulatisporites polygonalis (Ibrahim) Smith et Butterworth, 1967 1983b Gao Lianda, pl.115, 9-10; pl.116, 3
Reticulatisporites reticulatus Ibrahim, 1933 1983b Gao Lianda, pl.115, 1
Reticulatisporites serratus Gao et Hou, 1975 1983b Gao Lianda, pl.110, 4-5
Retusotriletes communis communis Naumova, 1953 1983b Gao Lianda, pl.114, 13
Rotaspora fracta (Shemel) Smith et Butter 1991 Gao Lianda, p.62
Rotaspora knoxi Butterworth et Williams, 1958 1983b Gao Lianda, pl.114, 26-27
Simozonotriletes arcuatus paputus Gao, 1983 1983b Gao Lianda, pl.115, 19
Spelaeotriletes cf. *microspinosus* Neves et Ioannides, 1974 1983b Gao Lianda, pl.116, 22
Stenozonotriletes pumilus (Waltz) Naumova, 1953 1983b Gao Lianda, pl.114, 10
Tripartites tripertitus (Horst) Potoni ée t Kremp 1956 1983b Gao Lianda, pl.115, 12
Tripartites verrucosus Gao, 1983 1983b Gao Lianda, pl.115, 16-17
Tripartites vetustus Schemel, 1950 1983b Gao Lianda, pl.115, 13-15
Triquitrites mirabilis Gao, 1983 1983b Gao Lianda, pl.115, 18
Vallatisporites sp.1 1983b Gao Lianda, pl.116, 11
Vallatisporites sp.2 1983b Gao Lianda, pl.116, 24
Vesicatispora circumligus (Staplin) Gao, 1983 1983b Gao Lianda, pl.116, 13-14
Vesicatispora guizhouensis Gao, 1983 1983b Gao Lianda, pl.116, 15
Vesticatisporites circuneligus Gao 1991 Gao Lianda, p.62
Vesticatisporites guizhouensis 1991 Gao Lianda, p.62
Waltzispora sp. 1983b Gao Lianda, pl.114, 28

2. Ganzhai Section, Pingtang County; Qilinzhai Reservior, Dushan County; and Wudang Section, Guiyang City: Xiangbai Formation

Acanthotriletes socraticun 1991 Gao Lianda, p.62
Anapiculatisporites minor 1991 Gao Lianda, p.62
Anaplanisporites atheticus 1991 Gao Lianda, p.62
Auroraspora asperella (Kedo) Van der Zwan 1991 Gao Lianda, p.62
Auroraspora macra 1991 Gao Lianda, p.62

Crassispora trychera 1991 Gao Lianda, p.62
Densosporites anulatus 1991 Gao Lianda, p.62
Diatomozonotriletes curiosu 1991 Gao Lianda, p.62
Diatomozonotriletes ubertus 1991 Gao Lianda, p.62
Foveolatisporites triangulatus 1991 Gao Lianda, p.62
Granulatisporites microgranifer (Ibrahim) Pot. et Kremp 1991 Gao Lianda, p.62
Knoxisporites pristinus 1991 Gao Lianda, p.62
Knoxisporites seniradiatus 1991 Gao Lianda, p.62
Lycospora noctuina 1991 Gao Lianda, p.62
Lycospora pusilla 1991 Gao Lianda, p.62
Lycospora rotunda 1991 Gao Lianda, p.62
Murospora aurita 1991 Gao Lianda, p.62
Pulvinisporites scolecophora 1991 Gao Lianda, p.62
Raistrickia subrotundata (Kedo) Gao 1991 Gao Lianda, p.62
Reinschospora speciosa 1991 Gao Lianda, p.62
Schopfites claviger 1991 Gao Lianda, p.62
Spelaeotriletes crustatus 1991 Gao Lianda, p.62
Tumulispora ordinaria 1991 Gao Lianda, p.62
Umbonatisporites distinctus 1991 Gao Lianda, p.62
Velamisporites sp. 1991 Gao Lianda, p.62

3. Xiguan Section, Pingtang County; Jialaohe Section, Dushan County: Shangsi Formation

Anapiculatisporites minor 1991 Gao Lianda, p.63
Apiculatisporites sp. 1991 Gao Lianda, p.63
Apiculiretusispora fructicusa Higgs 1991 Gao Lianda, p.63
Cingulizonates bialatus 1991 Gao Lianda, p.63
Cirratriradites saturni 1991 Gao Lianda, p.63
Convolutispora mallita 1991 Gao Lianda, p.63
Convolutispora mallita 1991 Gao Lianda, p.63
Convolutispora venusta 1991 Gao Lianda, p.63
Convolutispora vermoformis 1991 Gao Lianda, p.63
Crassispora kosankei 1991 Gao Lianda, p.63
Crassispora trychera 1991 Gao Lianda, p.63
Hymenospora sp. 1991 Gao Lianda, p.63
Lycospora pusilla 1991 Gao Lianda, p.63
Lycospora rotunda 1991 Gao Lianda, p.63
Mooreisporites sp. 1991 Gao Lianda, p.63
Murospora kosankei 1991 Gao Lianda, p.63
Murospora mutabilis 1991 Gao Lianda, p.63
Neostrickia sp. 1991 Gao Lianda, p.63
Raistrickia clavata 1991 Gao Lianda, p.63
Rotaspora knoxi 1991 Gao Lianda, p.63
Rugospora minuta 1991 Gao Lianda, p.63
Tripartites vetustus 1991 Gao Lianda, p.63

Umbonatisporites distinctus 1991 Gao Lianda, p.63
Waltzispora planiangulata 1991 Gao Lianda, p.63

Hubei

1. Changyang: Ziqiu Formation
Anapiculatisporites microspinosus Gao, 1992 1992 Gao Lianda, pl. I, 19
Apiculatisporis aculeatus (Ibrahim) Smith et Butterworth, 1967 1992 Gao Lianda, pl. I, 17
Cyclogranisporites palaeophytus Neves et Ioannides 1992 Gao Lianda, pl. I, 8
Cyclogranisporites rotundus (Naumova) Gao, 1992 1992 Gao Lianda, pl. I, 7
Granulatisporites sp. 1992 Gao Lianda, pl. I, 10
Leiotriletes microgranifer (Ibrahim) Potonié et Kremp, 1955 1992 Gao Lianda, pl. I, 1
Schulzospora eamphloptera (Waltz) H. S. et M., 1955 1992 Gao Lianda, pl. IV, 27
Umbonatisporites distinctus Clayton, 1970 1992 Gao Lianda, pl. I, 22
Waltzispora planiangulata Sullivan, 1964 1992 Gao Lianda, pl. I, 20
Waltzispora verrucosa Gao, 1992 1992 Gao Lianda, pl. I, 21

Changyang: Hezhou Formation
Leiotriletes notatus Hacquebard, 1957 1992 Gao Lianda, pl. I, 2

2. Songzi: Hezhou Formation
Lycospora pusilla (Ibrahim) Somers, 1973 1992 Gao Lianda, pl. II, 11
Triquitrites marginatus Hoffmeister, Staplin et Malloy, 1955 1992 Gao Lianda, pl. II, 12
Songzi: Gaolishan Formation
Cingulizonates capistratus (H. S. et M.) Staplin et Jansonius, 1964 1992 Gao Lianda, pl. II, 19
Foveolatisporites triangulatus Gao, 1984 1992 Gao Lianda, pl. I, 31
Spelaeotriletes pretiosus (Playford) Neves et Helt, 1970 1992 Gao Lianda, pl. III, 24
Triquitrites marginatus Hoffmeister, Staplin et Malloy, 1955 1992 Gao Lianda, pl. II, 12

References (Pp: 178–192):

Ao, Z. K., 1956, New materials of fossil plants from the Tseshui Coal Series in Shuangfeng County, Hunan: Journal of Central–South Institute of Mining and Metallurgy, vol. 1, no. 1, p. 33–36. (in Chinese)

Ao, Z. K., 1963, On a new species of ?*Sphenophyllum changshaense* from the Lower Carboniferous of Changsha, Hunan: Acta Palaeontologica Sinica, v. 11, no. 4, p. 610. (in Chinese)

Berry, C. M., and Wang, Y., 2006a, *Eocladoxylon* (*Protopteridium*) *minutum* (Halle) Koidzumi from the Middle Devonian of Yunnan, China: An Early *Rhacophyton*-like Plant?: International Journal of Plant Sciences, v. 167, no. 3, p. 551–566.

Berry, C. M., and Wang, Y., 2006b, A new plant attributed to Cladoxylopsida from the Middle Devonian of Yunnan Province, China: Review of Palaeobotany and Palynology, v. 142, no. 3, p. 63–78.

Berry, C. M., Wang, Y., and Cai, C. Y., 2003, A lycopsid with novel reproductive structures from the Upper Devonian of Jiangsu, China: International Journal of Plant Sciences, v. 164, no. 2, p. 263–273.

Cai, C. Y., 1981, On the occurrence of *Archaeopteris* in China: Acta Palaeontologica Sinica, v. 20, no. 1, p. 75–80. (in Chinese with English summary)

Cai, C. Y., and Cheng, L. Z., 1996, On a Chinese Givetian lycopod, *Longostachys latisporophyllus* Zhu, Hu et Feng, emend. Its morphology, anaomy and reconstruction: Palaeontographica Abteilung B, v. 238, no. 1–3, p. 1–43.

Cai, C. Y., and Li, X. X., 1982, Subdivision and correlation of the Devonian continental strata in China, p. 109–123. In: Nanjing Institute of Geology and Palaeontology, Academia Sinica, ed. Stratigraphic correlation chart in China with explanatory text: Beijing, Science Press. (in Chinese)

Cai, C. Y., and Li, X. X., 1995, A review of Silurain and Devonian macrofloras in China: Palaeontologia Cathayana, v. 6, p. 167–214.

Cai, C. Y., Lu, L. C., Wu, X. Y., and Zhang, G. F., 1988, The Devonian biostratigraphy in Jiangsu, the Lower Yangtze Platform. p. 169–217. In: Institute of Geology, Jiangsu Petroleum and Nanjing Institute of Geology and Palaeontology, Academia Sinica eds., The Stratigraphy and Paleontology of Jiangsu Province, 1. Sinian–Triassic biostratigraphy of the Lower Yangtze Peneplatform in Jiangsu Region: Nanjing, Nanjing University Press. (in Chinese)

Cai, C. Y., and Wang, Y., 1995, Devonian floras, p. 28–77. In: Li, X. X. ed. Fossil floras of China through the geological ages: Guangzhou, Guangdong Science and Technology Press.

Cai, C. Y., and Wu, X. Y., 1994, First discovery of *Cyclostigma*-like stem with ligular pit from Upper Devonian of Chaohu City, Anhui: Acta Palaeontologica Sinica, v. 33, no. 1, p. 75–84. (in Chinese with English summary)

Cai, C. Y., Ouyang, S., and Wang, Y., 1995, Silurian floras. p. 3–21. In: Li, X. X. ed. Fossil floras of China through the geological ages: Guangzhou, Guangdong Science and Technology Press.

Cai, C. Y., Ouyang, S., Wang, Y., Fang, Z. J., Rong, J. Y., Geng, L. Y., and Li, X. X., 1996, An Early Silurian vascular plant: Nature, v. 379, no. 6566, p. 592.

Cai, C. Y., Wen, Y. G., and Chen, P. Q., 1987, *Archaeopteris* florule from Upper Devonian of Xinhui County, central Guangdong and its stratigraphical significance: Acta Palaeontologica Sinica, v. 26, no. 1, p. 55–64. (in Chinese with English summary)

Chen, F., and Sun, K. Q., 1996, Early Carboniferous flora in southeastern Henan: Acta Botanica Sinica, v. 38, no. 4, p. 312–317. (in Chinese with English summary)

Chen, G. X., 1984, Pteridophyta, Spermatophyta. p. 560–615. In: Regional Geological Surveying Team of Hubei, ed., The palaeontological atlas of Hubei province: Wuhan, Hubei Science and Technology

Press. (in Chinese)

Chen, Q. S., 1976, The discovery of *Cardiopteris* Schimper in China: Chinese Journal of Geology, no. 2, p. 192. (in Chinese with English title)

Chen, Q. S., 1987, Early Carboniferous flora and stratigraphy in Qiantang area: Bulletin of the Nanjing Institute of Geology and Mineral Rescources, Chinese Academy of Geological Sciences, v. 8, no. 2, p. 71–84. (in Chinese with English summary)

Chen, Q. S., 1988, Fossil plants Sphenophyllales from Late Devonian Xihu Formation in Xiaoshan, Zhejiang: Acta Palaeontologica Sinica, v. 27, no. 4, p. 404–415. (in Chinese with English summary)

Chen, Q. S., 1999, Fossil plants Lycopsidales from Late Devonian Xihu Formation and Wutong Formation in Xiaoshan and Changxing, Zhejiang: Geology of Zhejiang, v. 15, no. 2, p. 15–23. (in Chinese with English summary)

Chen, Q. S., 2001, Fossil Plants Sphenophyllales From Late Devonian Wutung Formation in Changxing, Zhejiang: Geology of Zheijang, v. 17, no. 1, p. 19–26. (in Chinese with English summary)

Chen, Y. X., and Ouyang, S., 1985, Discovery of megaspores from the upper part of the Leigutai Formation in Juyong of Jiangsu and its stratigraphical significance: Acta Palaeontologica Sinica, v. 24, no. 3, p. 267–274. (in Chinese with English summary)

Cheng, L. Z., 1982, Palaeozoic plants. In: Geological Bureau of Hunan eds. The palaeontological atlas of Hunan. People’s Republic of China, Ministry of Geology and Mineral Resources, Geological Memoirs, Series 2 (1). p. 506–519. (in Chinese)

Deng, B., 1978, On the discovery of Early Carboniferous flora from Shanyang, S. Shanxi and its stratigraphic significance: Acta Geological Sinica, v. 52, no. 1, p. 15–21. (in Chinese with English summary)

Deng, L. H., 1978, On a new species of Early Carboniferous *Lepidodendron* of Xizang: Acta Palaeontologica Sinica, v. 17, no. 2, p. 230–231. (in Chinese with English summary)

Du, M. L., 1996, Cuticle characteristics of *Sublepidodendron* cf. *xinjiangense* Sun: Acta Botanica Sinica, v. 38, no. 3, p. 223–226. (in Chinese with English summary)

Fang, R. L., 1965, The plant fossils of Upper Devonian age in the northwest vicinity of Canton: Acta Scientiarum Naturalium Universitatis Sunyatseni, v. 7, no. 3, p. 383–394. (in Chinese with English summary)

Fang, X. S., Steemens, P., and Streel, M., 1993, New advancement on determination of the boundary between Devonian and Carboniferous, central Hunan: Chinese Science Bulletin, v. 38, no. 8, p. 732–736. (in Chinese)

Fang, Z. J., Cai, C. Y., Wang, Y., Li, X. X., Gao, L. D., Wang, C. Y., Geng, L. Y., Wang, S. Q., Wang, N. Z., and Li, D. Y., 1994, New advance in the study of the Silurian–Devonian boundary in Qujing, East Yunnan: Journal of Stratigraphy, v. 18, no. 2, p. 81–90. (in Chinese with English summary)

Feng, S. N., 1984, Devonian system and plant kingdom. p. 303–305. In: Institute of Geology and Mineral Resources of Yichang ed., Biostratigraphy of the Yangtze Gorge area, 3. Late Paleozoicera: Beijing, Geological Publishing House. (in Chinese)

Feng, S. N., 1985, The sequence of plant assemblage of Devonian in the boundary between Hunan and Hubei: Hunan Geology, v. 4, no. 4, p. 39–44. (in Chinese with English summary)

Feng, S. N., 1992, Carboniferous plants in Hainan Island. In: Wang, X. F., Ma, D. Q. and Jiang, D. H. eds., Geology of Hainan Island (1) Stratigraphy and Palaeontology. Beijing: Geological Publishing House, p. 125, 126. (in Chinese)

Feng, S. N., Chen, G. X., Xi, Y. H., and Zhang, C. F., 1977, Plants. p. 622–674. In: Hupei Institute of Geological Sciense et al. eds. Fossil atlas of Central–South China, II: Beijing, Geological Publishing House. (in Chinese)

Feng, S. N., Hu, Y. F., and Zhu, J. N., 1982, Fossil plants and their assemblages from the Early Carboniferous in Guangdong: Acta Botanica Sinica, v. 24, no. 4, p. 374–382. (in Chinese with English summary)

Feng, S. N., and Ma, J., 1988, On occurring of sporangia of *Protolepidodendron* Krejci and its significance: Acta Botanica Sinica, v. 30, no. 3, p. 333–337. (in Chinese with English abstract)

Feng, S. N., and Meng, F. S., 1975, Plant fossils from Hsiehchingsu Formation with special reference to the geologic age of siderite ore beds: Professional Papers of Stratigraphy and Palaeontology, v. 2, p. 122–131. (in Chinese)

Feng, S. N., and Zhang, R. J., 1999, Discovery of fossil plants in Yuntaiguan Formation from Zhouping area in Zigui and its significance: Geology and Mineral Resources of South China, no. 3, p. 35–44. (in Chinese with English summary)

Feng, S. N., and Zhang, R. J., 2000, The delimitation between Middle Devonian and Upper Devonian in Yangtze Gorges: Geology and Mineral Resources of South China, no. 4, p. 36–39. (in Chinese with English summary)

Gao, L. D., 1978, The Early Devonian spores and acritarchs in Liujing, Guangxi. p. 346–358. In: The Institute of Geology, Chinese Academy of Geological Sciences ed., Proceedings of the National Conferences on Devonian of South China: Beijing, Geological Publishing House. (in Chinese)

Gao, L. D., 1981, Devonian spore assemblages of China: Review of Palaeobotany and Palynology, v. 34, no. 1, p. 11–23.

Gao, L. D., 1983a, Devonian and Carboniferous spores. p. 481–520. In: Chengdu Institute of Geology and Mineral Resources eds. Paleontological atlas of southwestern China (Microfossils): Beijing, Geological Publishing House. (in Chinese)

Gao, L. D., 1983b, Discovery of Late Devonian spore assemblage from Nyalam County, Xizang (Tibet) and their stratigraphic significance: Contribution to the Geology of the Qinghai–Xizang (Tibet) Plateau, v. 8, p. 183–217. (in Chinese)

Gao, L. D., 1984, Early Devonian spores and acritarches from the Guijiatun Formation of Qujing, Yunnan: Bulletin of the Institute of Geology, Chinese Academy of Geological Sciences, v. 9, p. 125–136. (in Chinese with English summary)

Gao, L. D., 1985, Systematic Palaeontology: Spores, p. 50–85. In: Muhua Sections of Devonian–Carboniferours boundary beds: Beijing, Geological Publishing House. (in Chinese)

Gao, L. D., 1988a, The Early Carboniferous spore zones of Chouniugou Formation, Jingyuan County, Gansu Province: Professional Papers of Stratigraphy and Palaeontology, v. 22, p. 181–212. (in Chinese)

Gao, L. D., 1988b, Spores. p. 345–356. In: Chengdu Institute of Geology and Mineral Resources, eds., Devonian stratigraphy, paleontology and sedimentary facies of Longmenshan. Beijing: Geological Publishing House. (in Chinese)

Gao, L. D., 1989, The miospore assemblage from Yuntaiguan Formation in northwestern Hunan and western Hubei and its stratigraphical significance: Hunan Geology, v. 8, no. 1, p. 1–12. (in Chinese with English abstract)

Gao, L. D., 1990, Miospore zones in the Devonian–Carboniferous boundary beds in Hunan and their stratigraphical significance: Geological Review, v. 36, no. 1, p. 58–68. (in Chinese with English

abstract)

Gao, L. D., 1991, Late Devonian and Early Carboniferous miospore zones from southeastern Guizhou and the boundary of the Devonian And Carboniferous: Guizhou Geology, v. 8, no. 1, p. 59–69. (in Chinese with English abstract)

Gao, L. D., 1992, Palynostratigraphy at the Devonian–Carboniferous boundary in West Hubei and northwest Hunan Provinces: Bulletin of the Institute of Geology, Chinese Academy of Geological Sciences, v. 23, p. 171–192. (in Chinese)

Gao, L. D., 1993, Palynomorphs from upper parts of the Wengxiang Gruop in Dushan, Guizhou and its age: Guizhou Geology, v. 10, no. 1, p. 45–53. (in Chinese with English abstract)

Gao, L. D., 1996, Late Silurian–Early Devonian spores, acritarchs from Shidian, West Yunnan: Acta Geoscientia Sinica (Bulletin of the Chinese Academy of Geological Sciences), v. 17, no. 1, p. 105–114. (in Chinese)

Gao, L. D., and Hou, J. P., 1975, The Early–Middle Dovenian spores and their stratigraphical significance in Dushan and Duyun, Guizhou.: Professional Papers of Stratigraphy and Palaeontology, v. 1, p. 170–232. (in Chinese)

Gao, L. D., and Wang, G. X., 1990, Early Devonian miospore assemblage from the Yuankou Formation in southern Hunan and its stratigraphical significance: Hunan Geology, v. 9, no. 3, p. 1–9. (in Chinese with English abstract)

Geng, B. Y., 1983, *Stachyophyton* gen. nov., discovers from Lower Devonian of Yunnan and its significance: Acta Botanica Sinica, v. 25, no. 6, p. 574–579. (in Chinese with English abstract)

Geng, B. Y., 1985, *Huia recurvata*, a new plant from Lower Devonian of Southeastern Yunnan, China: Acta Botanica Sinica, v. 27, no. 4, p. 419–426. (in Chinese with English abstract)

Geng, B. Y., 1986, Anatomy and morphology of *Pinnatiramosus*, a new plant from the Middle Silurian (Wenlockian) of China: Acta Botanica Sinica, v. 28, no. 6, p. 664–670. (in Chinese with English abstract)

Geng, B. Y., 1992a, *Amplectosporangium*-A new genus of plant from the Lower Devonian of Sichuan, China: Acta Botanica Sinica, v. 34, no. 6, p. 450–454. (in Chinese with English abstract)

Geng, B. Y., 1992b, Studies on Early Devonian flora of Sichuan: Acta Phytotaxonomica Sinica, v. 30, no. 3, p. 197–211. (in Chinese with English abstract)

Geng, B. Y., and Zhu, W. Q., 1994, New observations on *Drepanophycus spinaeformis* from the Lower Devonian of Guizhou, China: Acta Phytotaxonomica Sinica, v. 32, no. 4, p. 345–348. (in Chinese with English abstract)

Gonez, P., Hung, N. H., Phuong, T. H., Clément, G., and Janvier, P., 2012. The oldest flora of the South China Block, and the stratigraphic bearings of the plant remains from the Ngoc Vung Series, northern Vietnam. Journal of Asian Earth Sciences, v. 43, no.1, p. 51–63.

Guo, Y., and Wang, D. M., 2009, *Archaeopteris halliana* from the Late Devonian (Famennian) of Anhui Province, China: Acta Geologica Sinica (English Edition), v. 83, no. 3, p. 479–491.

Guo, Y., and Wang, D. M., 2011, Anatomical reinvestigation of *Archaeopteris macilenta* from the Upper Devonian (Frasnian) of South China: Journal of Systematics and Evolution, v. 49, no. 6, p. 590–597.

Halle, T. G., 1927, Fossil plants from southwestern China: Palaeontologia Sinica, Series A, v. 1, no. 2, p. 1–26.

Hao, S. G., 1988, A new Lower Devonian genus from Yunnan, with notes on the origin of leaves: Acta Botanica Sinica, v. 30, no. 4, p. 441–448. (in Chinese with English abstract)

Hao, S. G., 1989a, *Gumuia Zyzzata*, a new plant from the Lower Devonian of Yunnan, China: Acta Botanica Sinica, v. 12, p. 954–961. (in Chinese with English abstract)

Hao, S. G., 1989b, A new zosterophyll from the Lower Denovian (Siegenian) of Yunnan, China: Review of Palaeobotany and Palynology, v. 57, no. 3, p. 155–171.

Hao, S. G., 1992, Some observations on *Zosterophyllum australianum* Lang & Cookson from the Lower Devonian of Yunnan, China: Botanical Journal of the Linnean Society, v. 109, no. 2, p. 189–202.

Hao, S. G., and Beck, C. B., 1991a, *Catenalis digitata*, gen. et sp. nov., a plant from the Lower Devonian (Siegenian) of Yunnan, China: Canadian Journal of Botany, v. 69, no. 4, p. 873–882.

Hao, S. G., and Beck, C. B., 1991b, *Yunia dichotoma*, a Lower Devonian plant from Yunnan, China: Review of Palaeobotany and Palynology, v. 68, no. 3, p. 181–195.

Hao, S. G., and Beck, C. B., 1993, Further observations on *Eophyllophyton bellum* from the Lower Devonian (Siegenian) of Yunnan, China: Palaeontographica Abteilung B, v. 230, no. 1–6, p. 27–41.

Hao, S. G., and Gensel, P. G., 1995, A new genus and species, *Celatheca beckii*, from the Siegenian (Early Devonian) of southeastern Yunnan, China: International Journal of Plant Sciences, v. 156, no. 6, p. 896–909.

Hao, S. G., and Gensel, P. G., 1998, Some new plant finds from the Posongchong Formation of Yunnan, and consideration of a phytogeographic similarity between South China and Australia during the Early Devonian: Science in China Series D: Earth Sciences, v. 41, no. 1, p. 1–13.

Hao, S. G., Gensel, P. G., and Wang, D. M., 2001, *Polythecophyton demissum*, gen. et sp. nov., a new plant from the Lower Devonian (Pragian) of Yunnan, China and its phytogeographic significance: Review of Palaeobotany and Palynology, v. 116, no. 1, p. 55–71.

Hao, S. G., and Mei, S., 1987, Further observations on *Archaeopteris macilenta* Lesquereux from western Hubei: Acta Palaeontologica Sinica, v. 26, p. 555–562. (in Chinese with English summary)

Hao, S. G., and Wang, D. M., 2000, Two Species of *Zosterophyllum* Penhallow (*Z. australianum* Lang and Cookson, *Z. ramosum* sp. nov.) from the Lower Devonian (Pragian) of Southeastern Yunnan, China: Acta Palaeontologica Sinica, v. 39 (Suppl.), p. 26–41.

Hao, S. G, Wang, D. M., and Beck, C. B., 2003, Observations on anatomy of *Adoketophyton subverticillatum* from the Posongchong Formation (Pragian, Lower Devonian) of Yunnan, China: Review of Palaeobotany and Palynology, v. 127, no. 3, p. 175–186.

Hao, S. G., Wang, D. M., and Wang, Q., 2004, A new species of *Estinnophyton* from the Lower Devonian Posongchong Formation, Yunnan, China: its phylogenetic and palaeophytogeographical significance: Botanical Journal of the Linnean Society, v. 146, no. 2, p. 201–216.

Hao, S. G., Wang, D. M., Wang, Q., and Xue, J. Z., 2006, A new lycopsid, *Zhenglia radiata* gen. et sp. nov., from the Lower Devonian Posongchong Formation of Southeastern Yunnan, China, and its evolutionary significance: Acta Geologica Sinica (English Edition), v. 80, no. 1, p. 11–19.

Hao, S. G., and Xue, J. Z., 2011, A new zosterophyll Plant, *Ramoferis* gen. nov., from the Posongchong Formation of Lower Devonian (Pragian) of southeastern Yunnan, China: Acta Geologica Sinica (English Edition), v. 85, no. 4, p. 765–776.

Hao, S. G., and Xue, J. Z., 2013, The Early Devonian Posongchong flora of Yunnan—A Contribution to an understanding of the evolution and early diversification of vascular plants. Beijing: Science Press, p. 1-366.

Hao, S. G., Xue, J. Z., Guo, D. L., and Wang, D. M., 2010, Earliest rooting system and root: shoot ratio from a new *Zosterophyllum* plant: New Phytologist, v. 185, no. 1, p. 217–225.

Hao, S. G., Xue, J. Z., Liu, Z. F., and Wang, D. M., 2007a, *Zosterophyllum* Penhallow around the

Silurian–Devonian boundary of northeastern Yunnan, China: International Journal of Plant Sciences, v. 168, no. 4, p. 477–489.

Hao, S. G., Xue, J. Z., Wang, Q., and Liu, Z. F., 2007b, *Yuguangia ordinata* gen. et sp. nov., a new lycopsid from the Middle Devonian (late Givetian) of Yunnan, China, and its phylogenetic implications: International Journal of Plant Sciences, v. 168, no. 8, p. 1161–1175.

Hao, S. G., Xue, J. Z., Zhu, X., and Wang, D. M., 2012, A new genus of Early Devonian plants with novel strobilar structures and vegetative appendages from the Posongchong Formation of Yunnan, China: Review of Palaeobotany and Palynology, v. 171, p. 73–82.

He, S. C., and Ouyang, S., 1993, Spore assemblages from Devonian–Carboniferous transitional beds of Hsihu Formation, Fuyang, W. Zhejiang: Acta Palaeontologica Sinica, v. 32, no. 1, p. 31–48. (in Chinese with English summary)

He, X. L., 1959, Some new materials of Lower Carboniferous fossils from Lungtan of Nanking with a discussion on the age of Wutung Series: Acta Palaeontologica Sinica, v. 7, no. 4, p. 319–328. (in Chinese with English summary)

Hilton, J., Geng, B. Y., and Kenrick, P., 2003, A novel Late Devonian (Frasnian) woody cladoxylopsid from China: International Journal of Plant Sciences, v. 164, no. 5, p. 793–805.

Hilton, J., and Li, C. S., 2000, Novel branching structures from the Lower Devonian and a note of caution: Acta Palaeobotanica, v. 40, no. 1, p. 9–16.

Hou, J. P., 1982a, Microfossils from the Maoshan Group of Jiangsu and Anhui, with a discussion on its geological age: Selected Papers from the First Symposium of the Palynological Society of China (1979). Beijing: Science Press., p. 167–172. (in Chinese)

Hou, J. P., 1982b, Some spore assemblages of the Devonian–Carboniferous transition from Xikuangshan District, central Hunan: Bulletin of the Institute of Geology, Chinese Academy of Geological Sciences, v. 5, p. 81–92. (in Chinese)

Hsü, J., 1966, On plant-remains from the Devonian of Yunnan and their significance in the identification of the stratigraphical sequence of this region: Acta Botanica Sinica, v. 14, no. 1, p. 50–69. (in Chinese with English abstract)

Huang, X. Y., 1982, Spores from the Zishan Formation of Pijiao, Fujian.: Selected Papers from the First Symposium of the Palynological Society of China (1979). Beijing: Science Press., p. 151–158. (in Chinese)

Jiang, Q. M., and Hu, J. M., 1982, Paleozoic spores and pollen. p. 595–635. In: Geological Bureau of Hunan ed., The palaeontological atlas of Hunan. People's Republic of China, Ministry of Geology and Mineral Resources, Geological Memoirs, Series 2, (1). Beijing: Geological Publishing House. (in Chinese)

Jiang, Q. M., and Hu, J. M., 1984, The Early Carboniferous spores and pollen and their stratigraphical significance in central Hunan: Hunan Geology, v. 3, no. 2, p. 41–44. (in Chinese with English abstract)

Jin, J. H., and Wu, Q. J., 2001, Late Devonian to Early Carboniferous strata and floral assemblages of the Guangzhou area: Journal of Stratigraphy, v. 25, no. 3, p. 166–173, 206. (in Chinese with English abstract)

Jin, J. H., Wu, Q. J., and Liao, W. B., 2000, Procathaysian flora of Guangzhou area: Acta Scientiarum Naturalium Universitatis Sunyatseni, v. 39, no. 4, p. 114–118. (in Chinese with English abstract)

Laveine, J. P., Lemoigne, Y., Zhang, S. Z., and Deng, G. G., 1992, The Carboniferous flora of the Huaxian area near Guangzhou, Guangdong Province, South China: Revue de Paleobiologie, Volume Special,

no. 6, p. 113–148.

Lee, H. H., Li, P. J., Chow, T. Y., and Guo, S. X., 1964, Plants. p. 73, 74, 80–82, 87, 88, 91, 114–117, 123–125, 128–131, 134–136, 139, 140. In: Wang Y ed., Handbook of index fossils of South China: Beijing, Science Press. (in Chinese)

Li, C. S., 1990, *Minarodendron cathaysiense* (gen. et comb. nov.), a lycopod from the late Middle Devonian of Yunnan, China: Palaeontographica Abteilung B, v. 220, no. 5–6, p. 97–117.

Li, C. S., 1992, *Hsüa robusta*, an Early Devonian plant from Yunnan Province, China and its bearing on some structures of early land plants: Review of Palaeobotany and Palynology, v. 71, p. 121–147.

Li, C. S., 2000, Devonian plants and stratum in Wuhan, Hubei Province, China.: Chinese Bulletin of Botany, v. 17 (Suppl.), p. 1–10. (in Chinese with English abstract)

Li, C. S., and Edwards, D., 1992, A new genus of early land plants with novel strobilar construction from the Lower Devonian Posongchong Formation, Yunnan Province, China: Palaeontology, v. 35, no. 2, p. 257–272.

Li, C. S., and Edwards, D., 1995, A reinvestigation of Halle's *Drepanophycus spinaeformis* Göpp. from the Lower Devonian of Yunnan Province, southern China: Botanical Journal of the Linnean Society, v. 118, no. 3, p. 163–192.

Li, C. S., and Edwards, D., 1996, *Demersatheca* Li et Edwards, gen. nov., a new genus of early land plants from the Lower Devonian, Yunnan Province, China: Review of Palaeobotany and Palynology, v. 93, no. 1, p. 77–88.

Li, C. S., and Edwards, D., 1997, A new microphyllous plant from the Lower Devonian of Yunnan Province, China: American Journal of Botany, v. 84, no. 10, p. 1441–1448.

Li, C. S., and Hsü, J., 1987, Studies on a new Devonian plant *Protopteridophyton devonicum* assigned to primitive fern from South China: Palaeontographica Abteilung B, v. 207, no. 111–131.

Li, C. S., and Hueber, F. M., 2000, *Cervicornus wenshanensis*, gen. et sp. nov, a Pragian (Early Devonian) plant with forked leaves from Yunnan, China: Review of Palaeobotany and Palynology, v. 109, no. 2, p. 113–119.

Li, C. S., Hilton, J., and Hemsley, A. R., 1997, Frasnian (Upper Devonian) evidence for multiple origins of seed-like structures: Botanical Journal of the Linnean Society, v. 123, no. 2, p. 133–146.

Li, D. Y., 1985, Plants. p. 118–126. In: Fang, R. S., Jiang, N. R., Fan, J. C., Cao, R. G., Li, D. Y., eds., The Middle Silurian–Early Devonian stratigraphy and fossils: Kunming, The People's Publishing House of Yunnan. (in Chinese)

Li, D. Y., and Ge, H., R., 2001, Early land plants and environment in Yunnan, China: Kunming, Yunnan Science and Technology Press, p. 1–166. (in Chinese)

Li, D. Y., and Yang, J. W., 1983, The discovery of Lower Devonian strata in Changning area, Yunnan: Contribution to the Geology of the Qinghai–Xizang (Tibet) Plateau, v. 11, p. 37–41. (in Chinese)

Li, H. M., 1981, New data on the flora of Zhishan Formation, Jiangxi: Bulletin of the Nanjing Institute of Geology and Mineral Rescources, Chinese Academy of Geological Sciences, v. 2, no. 2, p. 56–61. (in Chinese)

Li, H. M., HU, F. R., and Wu, H. Y., 1984, On the geological age and flora of clastic rocks beneath Huanglung Formation in Tungling area of Anhui Province: Bulletin of the Nanjing Institute of Geology and Mineral Resources, Chinese Academy of Geological Sciences, v. 5, no. 1, p. 152–162. (in Chinese)

Li, H. M., and Lan, S. X., 1984, The fossil plants from the upper part of the Wutong Formation in Chaoxian District, Anhui Province: Acta Botanica Sinica, v. 26, no. 2, p. 223–225. (in Chinese with English

summary)

Li, H. M., Lan, S. X., Li, X. X., Cai, C. Y., Wu, X. Y., Mo, Z. G., Chen, Q. S., and Wang, G. P., 1982, Plants. p. 336–378. In: Nanjing Institute of Geology and Mineral Resources ed., Palaeontological atlas of East China (2), Late Palaeozoic: Beijing, Geological Publishing House. (in Chinese)

Li, H. M., Zhang, Y., Ying, Z. E., Zhang, X. D., and Liu, W. H., 1987, Cishan Formation–Early Aikuanian strata in the Nanjing region: Journal of Stratigraphy, v. 11, no. 2. p. 116–119. (in Chinese with English abstract)

Li, W. H., 2002, Characteristics of Early Carboniferous spore assemblage in the Xingmei area: Guangdong Geology, v. 17, no. 1, p. 44–50. (in Chinese with English abstract)

Li, X. X., Cai, C.Y., 1977, Early Devonian *Zosterophyllum*-remains from Southwest China: Acta Palaeontologica Sinica, v. 16, no. 1, p. 12–34. (in Chinese with English summary)

Li, X. X., Cai, C. Y., 1978, A type-section of Lower Devonian strata in Southwest China with brief notes on the succession and correlation of its plant assemblages: Acta Geologica Sinica, v. 52, no. 1, p. 1–12. (in Chinese with English abstract)

Li, X. X., Cai, C. Y., and Wang, Y., 1995, *Hamatophyton verticillatum* (Gu & Zhi) emend. a primitive plant of Sphenopsida from the Upper Devonian–Lower Carboniferous in China: Palaeontographica Abteilung B, v. 235, p. 1–22.

Li, X. X., and Deng, L. H., 1974, Devonian plants. p. 248. In: Nanjing Institute of Geology and Palaeontology, Academia Sinica ed., Handbook of Stratigraphy and Palaeontology in Southwest China: Beijing, Science Press. (in Chinese)

Li, X. X., Shen, G. L., and Wu, X. Y., 1992, First appearance of Parispermae and their migration and dispersion: Acta Palaeontologica Sinica, v. 31, no. 1, p. 1–16. (in Chinese with English summary)

Li, X. X., and Wang, H. F., 1982, On the occurrence of Late Devonian plants from Mt. Longmenshan, North Sichuan: Acta Palaeontologica Sinica, v. 21, no. 1, p. 87–95. (in Chinese with English summary)

Liu, S. W., and Gao, L. D., 1985, Conchostracans and spores of Upper Devonian Huangjiadeng Formation in Hubei Province: Bulletin of the Chinese Academy of Geological Sciences, v. 11, p. 113–125. (in Chinese)

Liu, Z. J., and Shen, G. L., 1983, Plant Kingdom. In: Xi'an Institute of Geology and Mineral Resources ed. Palaeontological atlas of Northwest China, Shaanxi–Gansu–Ningxia (2). Beijing: Geological Publishing House., p. 480–506. (in Chinese)

Lu, L. C., 1980, On the occurrence of *Archaeoperisaccus* in E. Yunnan: Acta Palaeontologica Sinica, v. 19, no. 6, p. 500–505. (in Chinese with English summary)

Lu, L. C., 1981, Upper Devonian (Frasnian) sporo-pollen assemblage from the Damaidi area of Dukou, Sichuan Province.: Bulletin of Nanjing Institute of Geology and Palaeontology, v. 3, p. 91–130. (in Chinese)

Lu, L. C., 1988, Middle Devonian microflora from Haikou Formation at Shijiaopo in Zhanyi of Yunnan, China.: Memoirs of Nanjing Institute of Geology and Palaeontology, Academia Sinica, v. 24, p. 109–222. (in Chinese)

Lu, L. C., 1994, A restudy of "*Retispora lepidophyta*" in China: Acta Micropalaeontologica Sinica, v. 11, no. 4, p. 469–478. (in Chinese with English summary)

Lu, L. C., 1995, Miospores from Shaodong Member at Jieling Section of Hunan, China and their geological age: Acta Palaeontologica Sinica, v. 34, no. 1, p. 40–52. (in Chinese with English summary)

Lu, L. C., and Ouyang, S., 1976, The Early Devonian spore assemblage from the Xujiachong Formation at

Cuifengshan, in Qujing of Yunnan: Acta Palaeontologica Sinica, v. 15, no. 1, p. 21–40. (in Chinese with English summary)

Lu, L. C., and Ouyang, S., 1978, Devonian megaspores from the Zhanyi District, E. Yunnan: Acta Palaeontologica Sinica, v. 17, no. 1, p. 69–80. (in Chinese with English summary)

Meng, M. C., Wang, D. M., Xue, J. Z., and Zhu, X., New insights and evolutionary significance of the megasporangiate strobilus of *Minostrobus chaohuensis* (Lycopsida) from the Upper Devonian of South China. Review of Palaeobotany and Palynology, v. 190, 20–40.

Ouyang, S., and Chen, Y. X., 1987a, Miospore assemblages from the Devonian–Carboniferous transition in Jurong of southern Jiangsu with special reference to the geological age of the Wutung Group: Memoirs of Nanjing Institute of Geology and Palaeontology, Academia Sinica, v. 23, p. 1–92. (in Chinese)

Ouyang, S., and Chen, Y. X., 1987b, Miospores of the Famennian and Tournaisian deposits from a borehole in the Baoying District, central Jiangsu: Acta Micropalaeontologica Sinica, v. 4, no. 2, p. 195–215. (in Chinese with English summary)

"*Palaeozoic plants from China*" Writing Group of Nanjing Institute of Geology and Palaeontology, Institute of Botony, Academia Sinica (Gu et Zhi), 1974, Palaeozoic plants from China: Beijing, Science Press. (in Chinese)

Peng, Z. Q., Li, Z. H., Meng, F. S., and Wei, Y. X., 2010, New material of Late Devonian plants fossils from Huangjiadeng Formation in Yichang area, Hubei, China and its significance: Geological Bulletin of China, v. 29, no. 7, p. 980–987. (in Chinese with English summary)

Quan, B., and Han, D. X., 1997, Coal Forming Plants in Middle Devonian from South China: Journal of China University of Mining & Technology, v. 26, no. 3, p. 91–94. (in Chinese with English abstract)

Schweitzer, H. J., and Cai, C. Y., 1987, Beiträge zur Mitteldevon-flora Südchinas: Palaeontographica Abteilung B, v. 207, p. 1–109.

Schweitzer, H. J., and Li, C. S., 1996, *Chamaedendron* nov. gen., eine multisporangiate Lycophyte aus dem Frasnium Südchinas: Palaeontographica Abteilung B, v. 238, no. 1–3, p. 45–69.

Sun, D. W., Wang, Y, Xu, H. H., Fu, Q. , 2007, Restudy on the cuticles of late Middle Devonian coal from Luquan,Yunnan,China: Acta Palaeontologica Sinica, v. 46, no. 3, p. 355–364. (in Chinese with English summary)

Sze, H. C., 1937, On some *Psilophyton*-like remains from the Tiaomachien Series in central Hunan: Bulletin of the Geological Society of China, v. 17, no. 2, p. 245–254.

Sze, H. C., 1943, On the occurrence of *Sublepidodendron*, a lepidodendroid plant from Wutung Formation: Bulletin of the Geological Society of China, v. 23, p. 61–68.

Sze, H. C., 1952, Upper Devonian plants from China: Palaeontologia Sinica, Whole Number 136, New Series A, v. 4, p. 166–192.

Sze, H. C., 1953, Atlas of the Palaeozoic plants from China: Beijing, Chinese Academy of Sciences, p. 1–148. (in Chinese)

Sze, H. C., 1956, On some specimens of *Lepidodendropsis hirmeri* Lutz from the Wutung Series of Kiangsu: Scientia Sinica, v. 4, no. 1, p. 45–50. (in Chinese with English abstract)

Sze, H. C., 1958, On a Westphalian flora of the Tzushan Coal Series in Loping district, northeastern Kiangsi: Acta Palaeontologica Sinica, v. 6, no. 4, p. 375–388. (in Chinese with English summary)

Sze, H. C., and Chen, K. T., 1942, On the occurrence of *Neuropteris gigantea* Sternb. in Kiangsi: Bulletin of the Geological Society of China, v. 22, no. 3–4, p. 195–200.

Sze, H. C., and Hsü, J., 1954, Index fossils of China-plants: Beijing, Geological Publishing House, p. 1–83. (in Chinese)

Tang, S. Y., 1986, Spore assemblage from the Ceshui Formation of Lower Carboniferous in central Hunan: Professional Papers of Stratigraphy and Palaeontology, v. 16, p. 193–206. (in Chinese)

Tian, J. J., and Zhu, H. C., 2005, Devonian miospore biostratigraphy of the Longhuashan and Xichong Formations in Zhanyi of Yunnan: Journal of Stratigraphy, v. 29, no. 4, p. 311–312. (in Chinese with English abstract)

Wang, D. M., 2007a, *Tenuisa frasniana* gen. et sp. nov., a plant of euphyllophyte affinity from the Late Devonian of China: International Journal of Plant Sciences, v. 168, no. 9, p. 1341–1349.

Wang, D. M., 2007b, Two Species of *Zosterophyllum* from South China and dating of the Xujiachong Formation with a biostratigraphic method: Acta Geologica Sinica (English Edition), v. 81, no. 4, p. 525–538.

Wang, D. M., and Guo, Y., 2009, *Hamatophyton* from the Late Devonian of Anhui Province, South China and evolution of Sphenophyllales: Acta Geologica Sinica (English Edition), v. 83, no. 3, p. 492–503.

Wang, D. M., and Hao, S. G., 2001, A new species of vascular plants from the Xujiachong Formation (Lower Devonian) of Yunnan Province, China: Review of Palaeobotany and Palynology, v. 114, no. 3, p. 157–174.

Wang, D. M., and Hao, S. G., 2002, *Guangnania cuneata* gen. et sp. nov. from the Lower Devonian of Yunnan Province, China: Review of Palaeobotany and Palynology, v. 122, no. 1, p. 13–27.

Wang, D. M., and Hao, S. G., 2004, *Bracteophyton variatum* gen. et sp. nov., an Early Devonian Plant from the Xujiachong Formation of Yunnan, China: International Journal of Plant Sciences, v. 165, no. 2, p. 337–345.

Wang, D. M., Hao, S. G., and Wang, Q., 2003, Tracheid ultrastructure of *Hsüa deflexa* from the Lower Devonian Xujiachong Formation of Yunnan, China: International Journal of Plant Sciences, v. 164, no. 3, p. 415–427.

Wang, D. M., Hao, S. G., Tian, L., and Xue, J. Z., 2006a, Further study of the Late Devonian sphenopsid *Hamatophyton verticillatum* from China: International Journal of Plant Sciences, v. 167, no. 4, p. 885–896.

Wang, D. M., Hao, S. G., and Wang, Q., 2003, *Hsüa deflexa* sp. nov. from the Xujiachong Formation (Lower Devonian) of eastern Yunnan, China: Botanical Journal of the Linnean Society, v. 142, no. 3, p. 255–271.

Wang, D. M., Hao, S. G., and Wang, Q., 2005, *Rotafolia songziensis* gen. et comb. nov., a sphenopsid from the Late Devonian of Hubei, China: Botanical Journal of the Linnean Society, v. 148, no. 1, p. 21–37.

Wang, D. M., Hao, S. G., Wang, Q., and Xue, J. Z., 2006b, Anatomy of the Late Devonian sphenopsid *Rotafolia songziensis*, with a discussion of stelar architecture of the Sphenophyllales: International Journal of Plant Sciences, v. 167, no. 2, p. 373–383.

Wang, D. M., and Lin, Y. J., 2007, A new species of *Metacladophyton* from the Late Devonian of China: International Journal of Plant Sciences, v. 168, no. 7, p. 1067–1084.

Wang, D. M., Wan, Z. Z., and Cui, L., 2008, Further study of S*phenophyllum lungtanense*, with a discussion on the evolution of the Late Devonian Sphenophyllales in South China: Acta Scientiarum Naturalium Universitatis Pekinensis, v. 44, no. 2, p. 177–184. (in Chinese with English abstract)

Wang, D. W., 1956, On the occurrence of a Westphalian plant index fossil in the Tsaoliangyi Coal Series of the Tsinling Range: Scientia (Kexue Tongbao), v. 12, p. 39. (in Chinese)

Wang, G. X., Jing, Y. J., Zhuang, J. L., and Zhang, C. F., 1987, The Devonian–Carboniferous boundary of benthonic facies in central Hunan: Geological Review, v. 33, no. 4, p. 299–309. (in Chinese with English abstract)

Wang, H. F., 2003, The Middle Devonian Jinbaoshi flora from Longmen Mountains, northwest of Sichuan: Journal of Chengdu University of Technology (Science & Technology Edition), v. 30, no. 5, p. 474–484. (in Chinese with English abstract)

Wang, Q., Hao, S. G., Wang, D. M., and Dilcher, D. L., 2002, An anatomically preserved arborescent lycopsid, *Sublepidodendron songziense* (sublepidodendraceae), from the Late Devonian of Hubei, China: American Journal of Botany, v. 89, no. 9, p. 1468–1477.

Wang, Q., Hao S. G., Wang, D. M., Wang, Y., and Denk, T., 2003, A Late Devonian arborescent lycopsid *Sublepidodendron songziense* Chen emend. (Sublepidodendraceae Kräusel et Weyland 1949) from China, with a revision of the genus *Sublepidodendron* (Nathorst) Hirmer 1927: Review of Palaeobotany and Palynology, v. 127, no. 3–4, p. 269–305.

Wang, Q., Xue, J. Z., and Prestianni, C., 2007, *Sphinxiocarpon*, a new name for *Sphinxia* Li, Hilton & Hemsley, 1997–not Reid & Chandler, 1933: Lethaia, v. 40, no. 4, p. 393–393.

Wang, Q., Xue, J., and Prestianni, C., 2008, Erratum to the article: *Sphinxiocarpon*, a new name for *Sphinxia* Li, Hilton & Hemsley, 1997–not Reid & Chandler, 1933: Lethaia, v. 41, no. 3, p. 301–301.

Wang, Y., 1993, First discovery of *Eviostachya hoegii* Stockmans from Wutung Formation in China: Acta Palaeontologica Sinica, v. 32, no. 4, p. 430–441. (in Chinese with English summary)

Wang, Y., 1994, Lower Devonian miospores from Gumu in the Wenshan District, southeastern Yunnan: Acta Micropalaeontologica Sinica, v. 11, no. 3, p. 319–332. (in Chinese with English summary)

Wang, Y., 1996, Miospore assemblages from the Shaodong and Mengkung'ao Formations at Xikuangshan, central Hunan with discussion on Devonian–Carboniferous boundary: Acta Micropalaeontologica Sinica, v. 13, no. 1, p. 13–42. (in Chinese with English summary)

Wang, Y., 2000, *Kongshania* gen. nov. a new plant from the Wutung Formation (Upper Devonian) of Jiangning County, Jiangsu, China: Acta Palaeontologica Sinica, v. 39, no. (Suppl.), p. 42–56.

Wang, Y., 2001, A new lycopsid megaspore cone from the Upper Devonian of Chaohu, China: Botanical Journal of the Linnean Society, v. 136, p. 439–448.

Wang, Y., 2003, A new plant from the earliest Carboniferous of Jiangsu, China: Alcheringa, v. 27, no. 1, p. 51–61.

Wang, Y., and Berry, C., 2001a, A new small plant from the Xichong Formation of Yunnan, and discussion on the floral assemblage of late Middle Devonian in South China: Acta Palaeontologica Sinica, v. 40, no. 4, p. 424–432.

Wang, Y., and Berry, C. M., 2001b, A new plant from the Xichong Formation (Middle Devonian), South China: Review of Palaeobotany and Palynology, v. 116, no. 1, p. 73–85.

Wang, Y., and Berry, C. M., 2003, A reconsideration of *Dimeripteris cornuta* Schweitzer and Cai, a diminutive fossil plant from the Middle Devonian of Yunnan, China: Geobios, v. 36, no. 4, p. 437–446.

Wang, Y., and Berry, C. M., 2006, Morphology of a non-pseudosporochnalean cladoxylopsid from the Middle Devonian of Yunnan, South China: Palaeoworld, v. 15, no. 1, p. 54–67.

Wang, Y., Berry, C., Hao, S. G., Xu, H. H., and Fu, Q., 2007b, The Xichong flora of Yunnan, China:

diversity in late Mid Devonian plant assemblages: Geological Journal, v. 42, no. 3–4, p. 339–350.

Wang, Y., and Cai, C. Y., 1996, Further observation on *Stachyophyton yunnanense* Geng from Posongchong Formation (Siegenian) of SE Yunnan, China: Acta Palaeontologica Sinica, v. 35, p. 99–108. (in Chinese with English summary)

Wang, Y., and Cai, C. Y., 2007, A review of the study of a late Llandovery plant, *Pinnatiramosus qianensis* Geng, from Fenggang, Guizhou, sw China: Acta Palaeontologica Sinica, v. 46, no. 3, p. 269–277. (in Chinese with English summary)

Wang, Y., Edwards, D., Bassett, M., Xu, H. H., Xiao, J. F., Jiang, Q., and Zhang, X. L., 2013, Enigmatic occurrence of Permian plant roots in Lower Silurian rocks, Guizhou Province, China: Palaeontology, v. 56, p. 1–5.

Wang, Y., and Ouyang, S., 1997, Discovery of Early Silurian spores from Fenggang, northern Guizhou, and its Palaeobotanical significance: Acta Palaeontologica Sinica, v. 36, no. 2, p. 217–237. (in Chinese with English summary)

Wang, Yi, Rong, J. Y., Xu, H. H., Wang, C. Y., Wang, G. X., 2010, On the Late Silurian stratigraphy of the Zhangjiajie area, Hunan Province, with a discussion on age of the Xiaoxi formation: Journal of Stratigraphy, no. 2, p. 113–126. (in Chinese with English summary)

Wang, Y., and Xu, H. H., 2002, A new fossil plant from the earliest Carboniferous of China: International Journal of Plant Sciences, v. 163, no. 3, p. 475–483.

Wang, Y., and Xu, H. H., 2003, Studies on a new earliest Carboniferous plant: *Coenosophyton tristichus* gen. et sp. nov. from China: International Journal of Plant Sciences, v. 164, no. 1, p. 77–87.

Wang, Y., and Xu, H. H., 2005, *Sublepidodendron grabaui* comb. nov., a lycopsid from the Upper Devonian of China: Botanical Journal of the Linnean Society, v. 149, p. 299–311.

Wang, Y., Xu, H. H., and Wang, Q., 2012, Restudy of *Minostrobus chaohuensis* Wang (Lycopsida) from the Upper Devonian of Anhui, South China: Palaeoworld, v. 21, no. 1, p. 20–28.

Wang, Y., Zhu, H. C., and Li, J., 2005, Late Silurian plant microfossil assemblage from Guangyuan, Sichuan, China: Review of Palaeobotany and Palynology, v. 133, no. 3, p. 153–168.

Wang, Yi, Zhu, H. C., and Li, J., 2004, the plant-derived fragments of the Late Silurian from Guangyuan, Sichuan, South China: Acta Micropalaeontologica Sinica, v. 21, no. 1, p. 25–31.

Wang, Z., and Geng, B. Y., 1997, A new Middle Devonian plant: *Metacladophyton tetraxylum* gen. et sp. nov: Palaeontographica Abteilung B, v. 243, no. 4–6, p. 85–102.

Wellman, C. H., Zhu, H. C., Marshall, J. E. A., Wang, Y., Berry, C. M., and Xu, H. H., 2012, Spore assemblages from the Lower Devonian Xujiachong Formation from Qujing, Yunnan, China: Palaeontology, v. 55, p. 583–611.

Wen, Z. C., and Lu, L. C., 1993, Devonian–Carboniferous miospore assemblages from Xiaomu Section of Quannan, Jiangxi, China: Acta Palaeontologica Sinica, v. 32, no. 3, p. 295–327. (in Chinese with English summary)

Wu, C. Z., Zhao, L. C., and Deng, S. C., 1979, On the occurrence of *Sphenophyllum megalofolium* sp. nov. from the Wutung Group in Yixing of Jiangsu: Geological Review, v. 25, no. 1, p. 53–55. (in Chinese)

Wu, C. Z., Zhao, L. C., and Deng, S. C., 1982, Discovery and significance of *Archaeopteris* in the Wutong Formation near Nanjing Kongshan: Acta Botanica Sinica , v. 24, no. 3, p. 292–294. (in Chinese with English abstract)

Wu, Q. J., and Dong, L. L., 1993, The Devonian–Carboniferous boundary of continental facies in Guangzhou area: Acta Scientiarum Naturalium Universitatis Sunyatseni, v. 32, no. 1, p. 111–116.

(in Chinese with English abstract)

Wu, X. Y., 1992, Fossil plants from Yangshan Formation (Early Carboniferous) in Gushi, Henan: Acta Palaeontologica Sinica, v. 31, no. 5, p. 564–584. (in Chinese with English summary)

Wu, X. Y., and Deng, B., 1983, Fossil plants from the Caoliangyi Formation of Fengxian, Shaanxi: Acta Palaeontologica Sinica, v. 22, no. 2, p. 183–192. (in Chinese with English summary)

Wu, X. Y., and Liu, L. J., 1997, Feature of Namurian flora: Palaeoworld, v. 7, p. 159–175.

Wu, X. Y., Lu, T. D., and Yang, L. X., 1986, New material of fossil plants from the Tseshui Formation of Lianyuan–Lengshuijiang area, central Hunan: Acta Palaeontologica Sinica, v. 25, no. 4, p. 406–416. (in Chinese with English summary)

Wu, X. Y., and Zhao, X. H., 1981, Fossil plants from the Kaolishan Formation (Lower Carboniferous) in Jurong, southern Jiangsu: Acta Palaeontologica Sinica, v. 20, no. 1, p. 50–59. (in Chinese with English summary)

Wu, X. Y., and Zhao, X. H., 1982, Subdivision and correlation of the Carboniferous continental strata in China, p. 137–152. In: Nanjing Institute of Geology and Palaeontology, Academia Sinica, ed., Stratigraphical correlation chart in China with explanatory text: Beijing: Science Press. (in Chinese)

Xiong, C. H., Wang, D. M., Wang, Q., and Meng, M. C., 2012, A new euphyllophyte *Kunia venusta* gen. et sp. nov. from the Middle Devonian of Yunnan, South China: Journal of Systematics and Evolution, v. 50, no. 6, p. 540–549.

Xu, H. H., Marshall, J. E. A., Berry, C. M., Wang, Y., Zhu, H. C., and Wellman, C. H., 2012, Mid Devonian megaspores from Yunnan and North Xinjiang, China: Their palaeogeographical and palaeoenvironmental significances: Palaeoworld, v. 21, no. 1, p. 11–19.

Xu, H. H., and Wang, Q., 2009, Reconsideration of a specimen attributed to *Leclercqia complexa* (Lycopsida) from the Lower Devonian of Sichuan, South China: Review of Palaeobotany and Palynology, v. 153, no. 3, p. 331–335.

Xu, H. H., Zong, R. W., and Wang, Q., 2012, New materials of *Sphinxiocarpon*, a seed-like organ of putative lycopsid affinity, from the Late Devonian of Hubei, China: Palaeoworld, v. 21, no. 2, p. 131–136.

Xu, R., and Gao, L. D., 1991, Middle and early Upper Devonian miospore zonations in eastern Yunnan and the significance their stratigraphy: Acta Botanica Sinica , v. 33, no. 4, p. 304–313. (in Chinese with English abstract)

Xu, R., and Gao, L. D., 1994, Middle and early Late Devonian megaspores from eastern Yunnan: Bulletin of the Chinese Academy of Geological Sciences, v. 29, p. 119–132. (in Chinese)

Xue, J. Z., 2009, Two zosterophyll plants from the Lower Devonian (Lochkovian) Xitun Formation of northeastern Yunnan, China: Acta Geologica Sinica (English Edition), v. 83, no. 3, p. 504–512.

Xue, J. Z., 2010, Morphological observations of the seed-like structure *sphinxiocarpon wuhanium* from the Upper Devonian of Hubei, China: Acta Palaeontologica Sinica, v. 49, no. 4, p. 461–471. (in Chinese with English summary)

Xue, J. Z., and Hao, S. G., 2007, A lycopsid plant from the Upper Devonian of Changyang, Hubei Province, China: Acta Palaeontologica Sinica, v. 46, no. 2, p. 183–194. (in Chinese with English summary)

Xue, J. Z., and Hao, S. G., 2008, *Denglongia hubeiensis* gen. et sp. nov., a new plant attributed to Cladoxylopsida from the Upper Devonian (Frasnian) of South China: International Journal of Plant Sciences, v. 169, no. 9, p. 1314–1331.

Xue, J. Z., Hao, S. G., and Basinger, J. F., 2010, Anatomy of the Late Devonian *Denglongia hubeiensis*,

with a discussion of the phylogeny of the Cladoxylopsida: International Journal of Plant Sciences, v. 171, no. 1, p. 107–120.

Xue, J. Z., Hao, S. G., Wang, D. M., and Liu, Z. F., 2005, A new lycopsid from the Upper Devonian of Hubei Province, China: International Journal of Plant Sciences, v. 166, no. 3, p. 519–531.

Xue, J. Z., Hao, S. G., Zhu, X., and Wang, D. M., 2012, A new basal euphyllophyte, *Pauthecophyton* gen. nov., from the Lower Devonian (Pragian) of Yunnan, China: Review of Palaeobotany and Palynology, v. 183, p. 9–20.

Yan, T. Z., 1992, The microflora of the Tangjiawu and Xihu Formations in Zhejiang and their stratigraphical division and correlation: Regional Geology of China, v. 2, p. 111–117. (in Chinese with English abstract)

Yan, Y. Y., 1987, Lower Carboniferous in the Lower Yangtze Region: Bulletin of the Nanjing Institute of Geology and Mineral Rescources, Chinese Academy of Geological Sciences, v. 8, no. 2, p. 3–26. (in Chinese)

Yang, C. R., 1987, The discovery of *Archaeopteris* and age assignment of Wutong Formation: Regional Geology of China, no. 3, p. 285–286. (in Chinese with English title)

Yang, N., Li, C. S., and Edwards, D., 2009, *Hueberia zhichangensis* gen. et sp. nov, an Early Devonian (Pragian) Plant from Yunnan, China: Palynology, v. 33, no. 1, p. 113–124.

Yang, S. S., 2005, *Archaeopteris* and its plant fossil assemblage in the upper part of Wutong Group: Jiangsu Geology, v. 29, no. 2, p. 78–81. (in Chinese with English abstract)

Yang, W. P., and Jia, J. H., 1995, The new knowledges about the geology in Gengma, W. Yunnan: Journal of Stratigraphy, v. 19, no. 1, p. 62–71. (in Chinese with English abstract)

Yang, Y. C., 1987, Spores. In: Regional Geological Surveying Party, Bureau of Geology and Mineral Resources of Hunan Province ed. The Late Devonian and Early Carboniferous strata and palaeobiocoenosis of Hunan. Beijing: Geological Publishing House., p. 147–156. (in Chinese)

Zhang, C. F., and Liu, Y., G., 1964, On the geological age of the Tsushan Coal Series, Kiangsi: Acta Geologica Sinica, v. 44, no. 4, p. 371–381. (in Chinese with English abstract)

Zhang, J. H., 1978, Plants, p. 458–491. In: Stratigraphical Geological Working Team, Guizhou Province ed., Fossil atlas of Southwest China. Guizhou Volume, II. Geological Publishing House, Beijing. (in Chinese)

Zhang, L. J., and Gong, Y. M., 2009, New discovery of Late Devonian plant and trace fossils in Hougaoping, Guangyuan, Sichuan: Journal of Stratigraphy, v. 33, no. 2, p. 138–146. (in Chinese with English abstract)

Zhang, Q. S., 1965, New material of *Lepidodendron*-like plants from the Wutung Series of Kiangsu: Acta Palaeontologica Sinica, v. 13, no. 4, p. 610–616. (in Chinese with English summary)

Zhang, S. Z., Zhao, X. H., and Wu, X. Y., 1980, A culm florule from the Tseshui Series of Shuangfeng Xian, central Hunan: Acta Palaeontologica Sinica, v. 19, no. 3, p. 220–227. (in Chinese with English summary)

Zhang, Z. Y., 1978, On the age of the flora from the Chongyi Formation of southern Kiangsi: Journal of Nanjing University (Natural Sciences), no. 2, p. 71–80. (in Chinese with English abstract)

Zhao, R. X., and Qin, G. R., 1990, Remarks on the “Maotzefeng Shale” of northern Guangdong: Journal of Stratigraphy, v. 14, no. 1, p. 1–14. (in Chinese with English abstract)

Zhao, R. X., and Qin, G. R., 1997, Characteristics and correlation of the spore assemblage zone of Lower Carboniferous in northern Guangdong: Guangdong Geology, v. 12, no. 1, p. 19–26. (in Chinese with English abstract)

Zhao, R. X., Zhang, C. F., Xia, Z. F., Zuo, Z. B., and Liu, Z. H., 1978, The Devonian of Hunan Province, p. 68–89. In: The Institute of Geology, Chinese Academy of Geological Sciences ed., Proceedings of the National Conferences on Devonian of South China. Beijing: Geological Publishing House. (in Chinese)

Zhao, X. H., and Wu, X. Y., 1982a, Early Carboniferous flora and coal-bearing deposits of Hunan and Guangdong: Bulletin of Nanjing Institute of Geology and Palaeontology, Academia Sinica, v. 5, p. 1–40. (in Chinese with English summary)

Zhao, X. H., and Wu, X. Y., 1982b, Fossil plants from the Tzushan Series in Yudu of southern Jiangxi: Acta Palaeontologica Sinica, v. 21, no. 6, p. 699–708. (in Chinese with English summary)

Zhao, X. H., Wu, X. Y., and Chen, Q. S., 1986, Carboniferous flora in western Zhejiang: Memoirs of Nanjing Institute of Geology and Palaeontology, Academia Sinica, v. 22, p. 1–70. (in Chinese with English summary)

Zhou, D. C., and Zhu, Z. G., 1978, Brief introduction on Devonian of Jiangxi Province. p. 184–188. In: The Institute of Geology, Chinese Academy of Geological Sciences ed., Proceedings of the National Conferences on Devonian of South China: Beijing, Geological Publishing House. (in Chinese)

Zhou, H. Q., 1963, Plant fossils, p. 159–176. In: Chinese Academy of Geological Science eds. Handbook of Nanling fossils: Beijing, China Architecture & Building Press. (in Chinese)

Zhu, J. N., Hu, Y. F., and Feng, S. N., 1983, On occurrence and significance about the fossil plants from the Yuntaiguan Formation located between Hunan and Hubei: Acta Botanica Sinica, v. 25, no. 1, p. 75–81. (in Chinese with English abstract)

Zhu, W. Q., and Kenrick, P., 1999, A *Zosterophyllum*-like plant from the Lower Devonian of Yunnan Province, China: Review of Palaeobotany and Palynology, v. 105, no. 1, p. 111–118.

Zhu, X., Xue, J. Z., Hao, S. G., and Wang, D. M., 2011, A new species of *Adoketophyton* from the Lower Devonian (Pragian) Posongchong Formation of Yunnan, China: Review of Palaeobotany and Palynology, v. 164, no. 3, p. 238–246.
